# Isoselenourea‐Catalyzed Enantioselective Pyrazolo‐Heterocycle Synthesis Enabled by Self‐Correcting Amide and Ester Acylation

**DOI:** 10.1002/anie.202425305

**Published:** 2025-03-31

**Authors:** Martha I. Prindl, Matthew T. Westwood, Alister S. Goodfellow, Aidan P. McKay, David B. Cordes, Michael Bühl, Andrew D. Smith

**Affiliations:** ^1^ EaStCHEM School of Chemistry University of St Andrews St Andrews Fife KY16 9ST UK

**Keywords:** Enantioselective Michael addition, Isoselenourea, Pyrazole, Self‐correcting amide acylation

## Abstract

Pyrazole heterocycles are prevalent in a wide range of medicinal and agrochemical compounds, and as such, the development of methods for their enantioselective incorporation into molecular scaffolds is highly desirable. This manuscript describes the effective formation of fused pyrazolo‐pyridones and ‐pyranones in high enantioselectivity (up to >99:1 er) via an isoselenourea (HyperSe) catalyzed enantioselective [3 + 3]‐Michael addition‐cyclization process using readily available pyrazolylsulfonamides or pyrazolones as pronucleophiles and α,β‐unsaturated anhydrides as starting materials. Mechanistic analysis indicates an unusual self‐correcting reaction pathway involving preferential [1,2]‐addition of the pronucleophile to initially generate an intermediate amide or ester that can be intercepted by isoselenourea acylation, leading to productive formation of the fused heterocyclic products with high enantiocontrol. The scope and limitations of this process are developed across a range of examples, with insight into the factors leading to the observed enantioselectivity provided by density functional theory (DFT) analysis.

## Graphical abstract

A highly enantioselective isoselenourea catalyzed route to fused pyrazolo‐pyridone and ‐pyranone heterocycles has been developed. Mechanistic and computational studies highlight the significance of an intermediate amide or ester product derived from 1,2‐addition that can be diverted to the desired pathway through isoselenourea acylation.

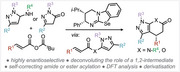

## Introduction

**Scheme 1:**
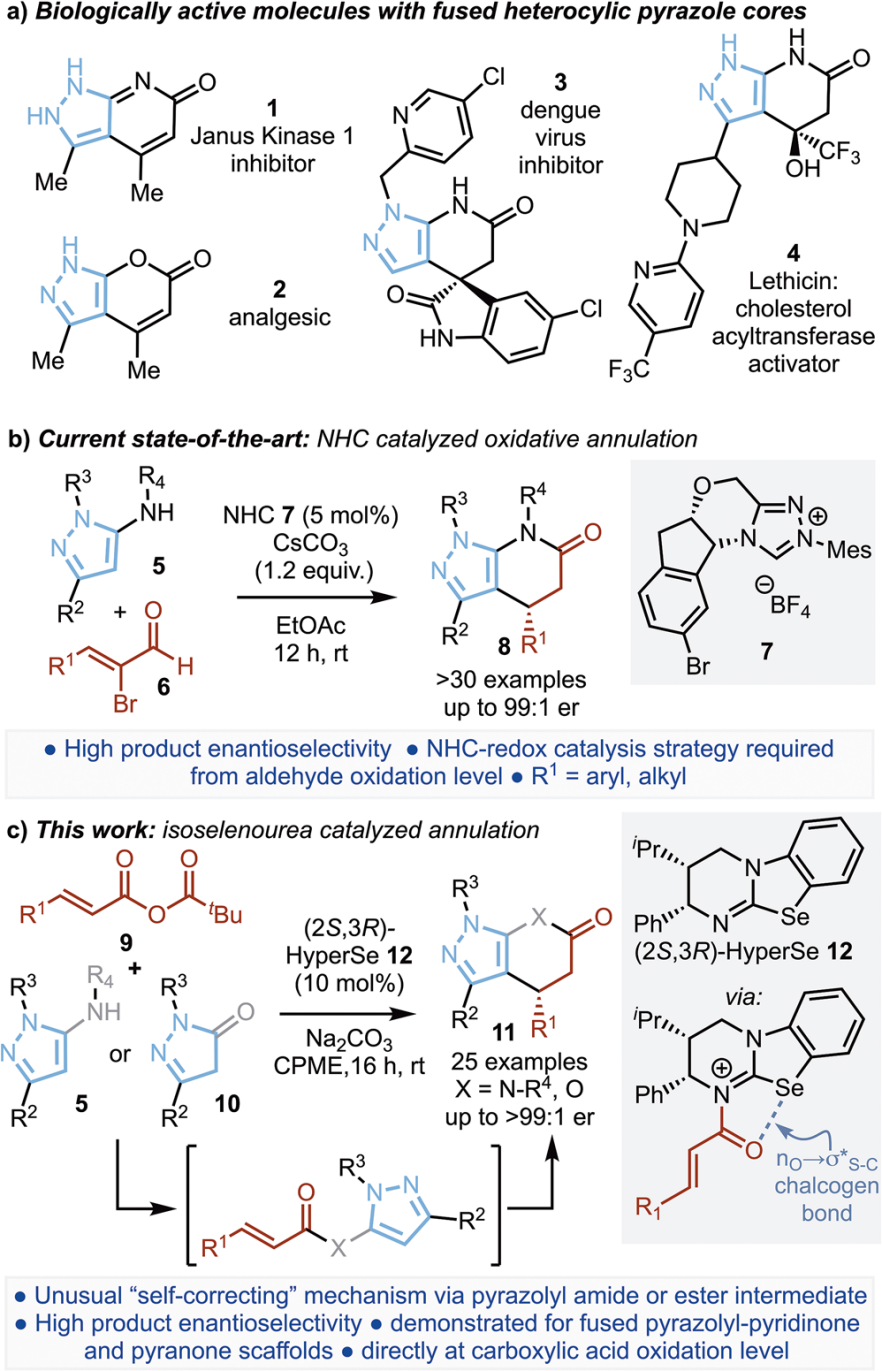
a) 5‐aminopyrazole and pyrazolone bioactive derivatives. b) Current NHC catalyzed routes to enantioenriched pyrazolopyridinones. c) This work: isoselenourea catalyzed generation of fused pyrazolo‐pyridinones and pyrazolones.

Nitrogen‐containing heterocycles are ubiquitous within pharmaceutical and agrochemical compounds, with 82% of US FDA‐approved drugs containing at least one nitrogen heterocycle.^[^ ^1^, ^2^, ^3^, ^4^ ^]^ Among the heterocyclic scaffolds incorporated within this classification, fused polycyclic structures that contain the pyrazole motif are prevalent within a number of biologically active drugs. Representative examples of these structures include the immune modulating Janus Kinase I inhibitor **1**,^[^ ^5^ ^]^ the dengue virus inhibitor **3** ^[^ ^6^ ^]^ and the lecithin cholesterol acyltransferase (LCAT) activator **4** ^[^ ^7^ ^]^ (Scheme 1). Given the significant interest in these scaffolds a variety of synthetic methods for their preparation have been developed.^[^ ^8^, ^9^, ^10^, ^11^ ^]^ For example, the synthesis of dihydropyrazolo[3,4‐*b*]pyridinones in racemic form has been achieved effectively through a three‐component coupling process involving Meldrum's acid, an aldehyde, and the corresponding 5‐aminopyrazole.^[^ ^5^, ^12^ ^]^ Catalytic enantioselective methods to generate such products in enantiopure form have also been demonstrated, with the most powerful approach developed to date (established during the completion of this manuscript) employing N‐heterocyclic carbene (NHC) catalysts.^[^ ^6^, ^7^, ^13^, ^14^ ^]^ As a representative example, Wang and co‐workers,^[^ ^7^ ^]^ alongside Chi and co‐workers,^[^ ^13^ ^]^ have demonstrated that treatment of α‐bromoenals **6** with the chiral NHC derived from **7** and 5‐aminopyrazole **5** allows the efficient synthesis of pyrazolopyridinones **8** with excellent levels of enantiocontrol (>95:5 er). These NHC‐catalyzed reaction processes all proceed via the in situ formation of the corresponding α,β‐unsaturated acyl azolium intermediate, which requires either stoichiometric in situ oxidation or highly functionalized α‐bromoenal starting materials for its formation.^[^ ^6^ ^]^ Complementary to this approach, Pericàs and co‐workers recently employed immobilized isothioureas in a formal [4 + 2] cycloaddition to generate highly enantioenriched fused‐pyrazolones **11** via the corresponding C(1)‐ammonium enolate.^[^ ^15^ ^]^

Isothioureas have been widely used as enantioselective Lewis base organocatalysts over the last two decades since being established for the kinetic resolution of secondary alcohols by Birman and Li.^[^ ^16^ ^]^ The use of these Lewis basic tertiary amines has been applied to a range of reactivity modes employing acyl ammonium,^[^ ^17^, ^18^, ^19^ ^]^ C(1)‐ammonium enolate,^[^ ^20^ ^]^ α,β‐unsaturated acyl ammonium^[^ ^21^, ^22^ ^]^ and silyl ammonium intermediates,^[^ ^23^ ^]^ as well as allenoate activation.^[^ ^24^, ^25^ ^]^ Recent work has demonstrated that enhancement in catalytic activity can be achieved through variation of the chalcogen (Ch) substituent within the catalyst structure that modulates the key chalcogen bonding interaction (*n*_O_→σ*_C‐Ch_) in catalytically competent intermediates. For example, the incorporation of selenium (to give an isoselenourea) rather than sulfur typically leads to optimum reaction rates in a range of catalytic processes with equivalent levels of stereocontrol.^[^ ^26^ ^]^ In this context, this manuscript describes a rare example where an isoselenourea catalyst leads to enhanced reactivity and enantioselectivity over the equivalent isothiourea in a Michael addition‐cyclization between pyrazolylsulfonamides and pyrazolone pronucleophiles with α,β‐unsaturated anhydrides. Under optimized reaction conditions, this leads to fused heterocycles with excellent enantiocontrol (up to >99:1 er). Mechanistic investigations indicate this preference derives from interception of an intermediate amide or ester derived from initial 1,2‐addition of the pronucleophile by isoselenourea acylation, diverting this to a productive catalytic pathway.

## Results and Discussion

### Optimization and Discovery of “Self‐Correcting” Mechanism

Initial optimization utilized 4‐nitrophenyl‐4,4,4‐trifluorobut‐2‐enoate **13** and *N*‐methanesulfonyl (Ms) pyrazoleamine **15** (Table 1) as model substrates for optimization. Screening of various isothiourea and isoselenourea catalysts, solvents, bases, and temperatures was investigated; however, in all cases an inseparable mixture of the desired fused heterocyclic product **19** and the corresponding acyclic ester **20** was observed (see Section S2.1 for full details). For example, using HyperBTM **17** and HyperSe **12** as catalysts in MeCN led to an approximate 60:40 ratio of the desired heterocyclic product **19**: acyclic ester **20** (entries 1 and 2), with higher enantioenrichment for both **19** and **20** observed using HyperSe (92:8 er) than HyperBTM (88:12 er). The formation of ester **20** is consistent with trapping of a post‐conjugate addition α,β‐unsaturated acyl isoselenouronium ion by 4‐nitrophenolate rather than intramolecular lactamization to give **19**. Use of an alternative α,β‐unsaturated pivaloyl mixed anhydride **14** was predicted to minimize competitive formation of any undesired acyclic products analogous to **20** by reducing the nucleophilicity of the acyl isoselenouronium counterion. Pleasingly, use of **14** afforded exclusively the desired heterocyclic lactam product **19** in 75% yield and 91:9 er (entry 3). Various solvents were subsequently screened (entries 4–6), with cyclopentyl methyl ether (CPME) determined to be optimal, providing **19** in excellent yield and enantioselectivity (90% yield, 99:1 er). Notably, the use of an auxiliary base was not necessary in the reaction using mixed anhydride **14**, consistent with the pivalate anion acting as an in situ generated base. Further optimization (entries 7–9) varied reactant stoichiometry and concentration; using 1.5 equivalents of the anhydride **14** with 1.0 equivalent of N‐Ms aminopyrazole **15** in CPME (0.1 m) using (2*S*,3*R*)‐HyperSe **12** (10 mol%) at room temperature showed effective catalysis, giving **19** in 95% yield and 99:1 er (entry 8). Reduced catalyst loading of HyperSe **12** (5 mol%) was also tolerated but resulted in reduced product yield (70%) while maintaining high product enantioselectivity (entry 9). HyperBTM **17** afforded **19** with a reduced yield of 64% with maintained enantioenrichment (entry 11). Interestingly, monitoring the reaction using HyperBTM but stopping the reaction after 3.5 h gave ∼20% conversion to **19**, but gave preferential formation of 1,2‐product amide **21** (entry 12). Use of the alternative isothiourea BTM **18** afforded no product, instead affording ∼75% of **21**, with the remaining yields attributed to starting material (entry 10). A control experiment using unprotected 5‐aminopyrazole derivative **16** (entry 13) gave exclusive formation of 1,2‐addition product **22** that was generated in quantitative yield, indicating the crucial role of the electron withdrawing N‐Ms substituent on the 5‐aminopyrazole in facilitating product formation. No reaction was observed with N‐Ms pyrazoleamine **15** without catalyst (entry 14).

**Table 1:** Optimization of the reaction conditions. Yields are isolated. Enantiomeric ratio measured by HPLC analysis on a chiral stationary phase.

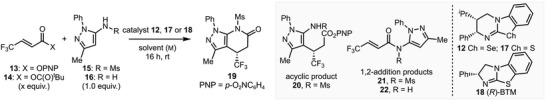							
Entry	Acyl Donor (Equiv.)	Catalyst (mol%)	Solvent (0.1 m)	19 (%)	20 (%)	21/22 (%)	er
1	**13** (1.2)	**17** (10)	MeCN	59	36	−	88:12
2	**13** (1.2)	**12** (10)	MeCN	61	39	−	92:8
3	**14** (1.2)	**12** (10)	MeCN	75	−	−	91:9
4	**14** (1.2)	**12** (10)	Dioxane	65	−	−	98:2
5	**14** (1.2)	**12** (10)	Acetone	50	−	−	97:3
6	**14** (1.2)	**12** (10)	CPME	90	−	−	99:1
7^a)^	**14** (1.2)	**12** (10)	CPME	85	−	−	98:2
**8**	**14 (1.5)**	**12 (10)**	**CPME**	**95**	−	−	**99:1**
9	**14** (1.5)	**12** (5)	CPME	70	−	−	99:1
10	**14**(1.5)	**18** (10)	CPME	0	−	75	−
11	**14** (1.5)	**17** (10)	CPME	64	−	−	98:2
12^b)^	**14** (1.5)	**17** (10)	CPME	22	−	74	−
13^c)^	**14** (1.5)	**12** (10)	CPME	−	−	100	−
14	**14** (1.5)	None	CPME	−	−	−	−

^a)^ 0.2 m concentration.

^b)^ Reaction time 3.5 h; yields indicated by ^1^H NMR analysis with 1,3,5‐trimethoxybenzene as internal standard.

^c)^ Using **16** (R = H).

**Scheme 2:**
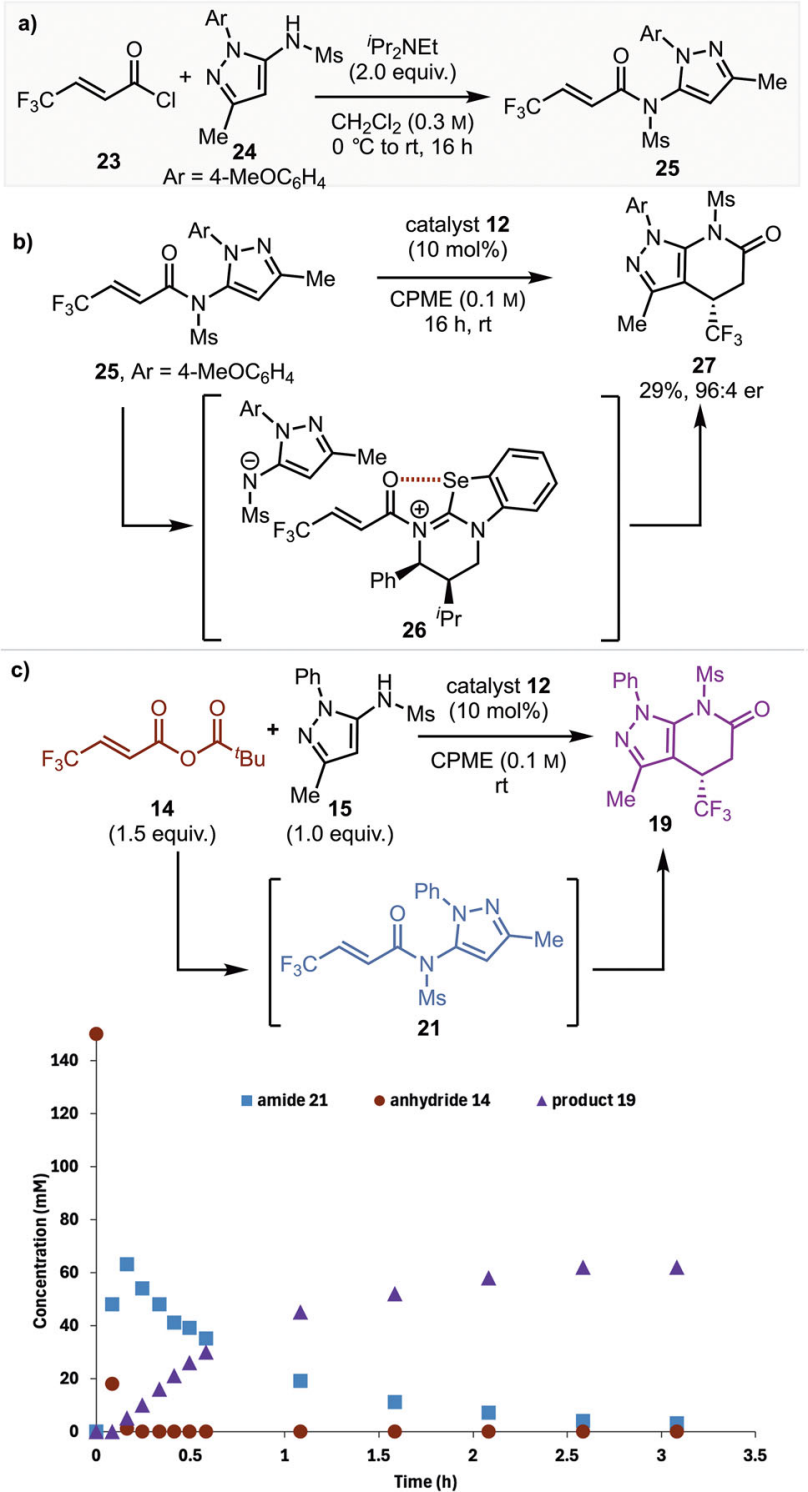
Yields are isolated. Enantiomeric ratio measured by HPLC analysis on a chiral stationary phase. a) Synthesis of a 1,2‐addition product. b) Demonstration of amide acylation. Note: The opposite enantiomer of catalyst **12** was used. c) Reaction profile analysis.

Given the unusual preference for the isoselenourea HyperSe catalyst to deliver maximum product enantioselectivity and reactivity, plus the observation of competitive 1,2‐addition products, mechanistic investigations into the developed process were followed. The N‐4‐OMeC_6_H_4_ substituted amide 1,2‐addition product **25** was synthesized from the corresponding acid chloride **23** and aminopyrazole **24**, with its constitution assigned unambiguously using ^1^H‐^15^N HMBC analysis. The amide **25** was then treated with (2*R*,3*S*)‐HyperSe **12** (10 mol%) in CPME, giving the desired product **27** in 20% yield and 99:1 er (Scheme 2b). The conversion of the 1,2‐addition product **25** into enantioenriched product **27** indicates that the formation of **25** within the Michael addition–annulation is reversible. Control reactions indicated that no background reaction between the anhydride **14** and aminopyrazole **24** is observed in CPME, indicating that **27** must form via reversible 1,2‐addition with the catalyst generating the α,β‐unsaturated acyl isoselenouronium ion pair **26**. While unusual for amides to undergo Lewis base acylation, an explanation for this pathway relates to a distorted or twisted amide within **25**. The reactivity of related “twisted” amides has been recognized in the literature^[^ ^27^, ^28^, ^29^, ^30^ ^]^ and exploited extensively in both transition metal coupling or transition metal‐free amide functionalization,^[^ ^31^ ^]^ but to the best of our knowledge has not been exploited in an organocatalytic transformation. In such cases, distortion of planarity disrupts the usual n_N_→π*_C═O_ conjugation, leading to a weakening of the C─N bond, facilitating cleavage. Unfortunately, despite repeated attempts, X‐ray crystallographic analysis of **25** could not be obtained to confirm the proposed distortion from planarity in the solid state, although density functional theory (DFT) calculations predict a torsion of *τ* = 10.5° around the N─C(═O) bond (*τ* corresponds to the Winkler–Dunitz twist angle describing an out‐of‐plane rotation around the N–C(═O) bond; see Section S6 for further details).^[^ ^32^, ^33^ ^]^

A series of further control reactions were performed to monitor the evolution of product formation with time (Scheme 2c). Treatment of pyrazolylsulfonamide **15** and mixed anhydride **14** with (2*S*,3*R*)‐HyperSe **12** under the optimized conditions led to rapid consumption of the anhydride **14** and conversion to the 1,2‐addition product **21** (maximum concentration ∼65 mM) within 12 min. Onward formation of amide heterocyclic product **19** correlates to a reduction in concentration of the amide 1,2‐intermediate **21**. Full consumption of the 1,2‐addition product **21** is observed within 8 h to give product **19**. Using 4‐OMeC_6_H_4_ substituted aminopyrazole **24**, comparative reactions using HyperSe **12** and HyperBTM **17** were investigated, with similar reaction profiles observed involving rapid formation of the 1,2‐product **25** from the anhydride. However, onward product formation is significantly faster with HyperSe **12** than with HyperBTM **17** (see Section S5.8), consistent with previously observed enhanced stabilization of acylated isoselenourea intermediates and associated transition states.^[^ ^26^ ^]^

### Scope and Limitations

**Scheme 3:**
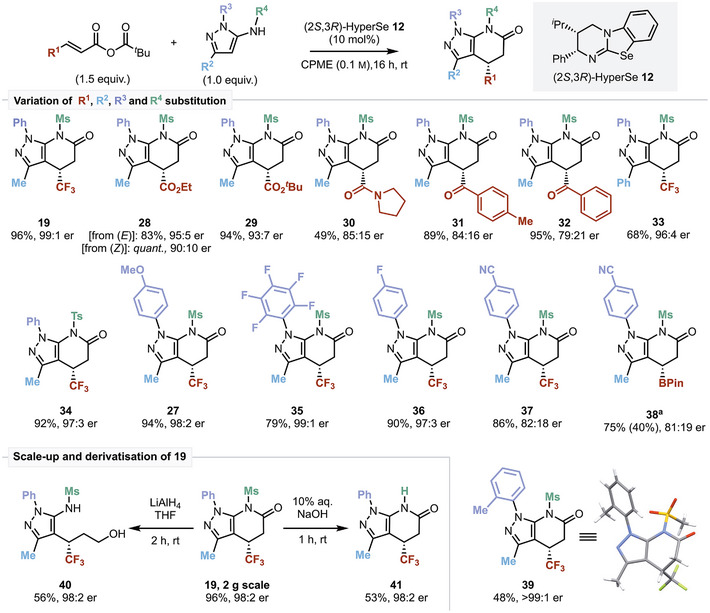
Scope of the [3 + 3]‐Michael addition‐cyclization approach to pyrazolopyridinones. Yields are isolated (for **38** isolated yields in parentheses). Enantiomeric ratio measured by HPLC analysis on a chiral stationary phase. ^a^ Dioxane (0.1 m), K_2_CO_3_ (1.0 equiv.), 20 mol% (2*S*,3*R*)‐HyperSe **12** (see Section S2.3).

The scope and limitations of this process were then explored under the developed conditions using pyrazolylsulfonamide pronucleophiles (Scheme 3). First, variation of the α,β‐unsaturated anhydride reaction component (R^1^ substituent) was investigated. Consistent with the electron withdrawing trifluoromethyl substituent, structural variation to encompass ester, amide, and ketone functionality at the β‐position of the mixed anhydride was successful, giving products **28**–**32** in good to excellent yields (up to 95%) and good to high product enantioselectivity (up to 95:5 er). Notably, both (*E*)‐ and (*Z*)‐anhydride configurations of the ethyl ester gave the same product enantiomers, **28**, in 95:5 er and 90:10 er, respectively, in excellent yields. This is consistent with previous work that implicated rapid Lewis base promoted (*Z*)‐ to (*E*)‐isomerization under the reaction conditions.^[^ ^34^ ^]^

A *tert*‐butyl ester functionality was readily tolerated, giving **29** in 94% yield and 93:7 er. Amide and aryl ketone substituents were also tolerated, albeit giving products with reduced enantioselectivity (up to 85:15 er). An improved enantiomeric ratio of **32** (from 79:21 er to 85:15 er) was achieved through a shorter reaction time of 2 h, to the detriment of a reduced yield (61%). Disappointingly, the use of β‐aryl and β‐alkyl substituents at R^1^ was not successful in this series using **15** as a pronucleophile, instead resulting in preferential 1,2‐addition in varying yields (see Section S4 for further information). This limitation highlights the requirement of electron withdrawing β‐substituents within the anhydride for the desired Michael addition to occur in this series.

Substituent variation within the aminopyrazole functionality was then explored, with consistently high enantioselectivity observed. For example, incorporation of an R^2^ aryl substituent was readily tolerated, giving **33** in 68% yield and 96:4 er. R^3^ variation indicated that both electron‐donating (4‐MeOC_6_H_4_), halogen (4‐FC_6_H_4_), and electron‐withdrawing (C_6_F_5_) substituents gave products with high yields and enantioselectivities. The addition of a 4‐NCC_6_H_4_ group gave reduced product enantioselectivity (82:18 er), while the introduction of the 2‐MeC_6_H_4_ group on **15** afforded **39** as a rotameric mixture in >99:1 er, albeit with lower yield. The absolute configuration within **39** was unambiguously determined by X‐ray crystal diffraction analysis, and all subsequent product configurations were assigned by analogy.^[^ ^35^ ^]^ Subsequent investigations probed the inclusion of synthetically useful β‐BPin substituted anhydrides as enantioselective organocatalyzed addition to β‐boryl compounds, remains relatively underdeveloped in the literature. Interestingly, successful reactivity leading to enantioenriched BPin substituted **38** required re‐optimization, and was only observed with the electron withdrawing 4‐NCC_6_H_4_ group as the R^3^ substituent of the aminopyrazole (see Section S2.3). Heterocycle **38** suffered significant instability during chromatographic purification with moderate yields relative to the observed NMR yields (75% NMR, 40% isolated) and gave promising but moderate enantioselectivity (81:19 er).

The developed process could be readily performed on a 2‐g scale using 5 mol% (2*S*,3*R*)‐HyperSe **12** over 24 h to obtain **19** in 96% yield and 98:2 er. Notably, at this scale, aqueous acidic extraction was employed to facilitate 84% catalyst recovery. From **19**, derivatization showed that N‐Ms deprotection was readily achieved by treatment with aqueous sodium hydroxide, giving **41** in moderate yield (53%) with no erosion of enantioselectivity observed. Furthermore, reduction with LiAlH_4_ provided the enantioenriched γ‐trifluoromethylated alcohol **40** in moderate yield (56%) whilst maintaining stereochemical fidelity.

**Scheme 4:**
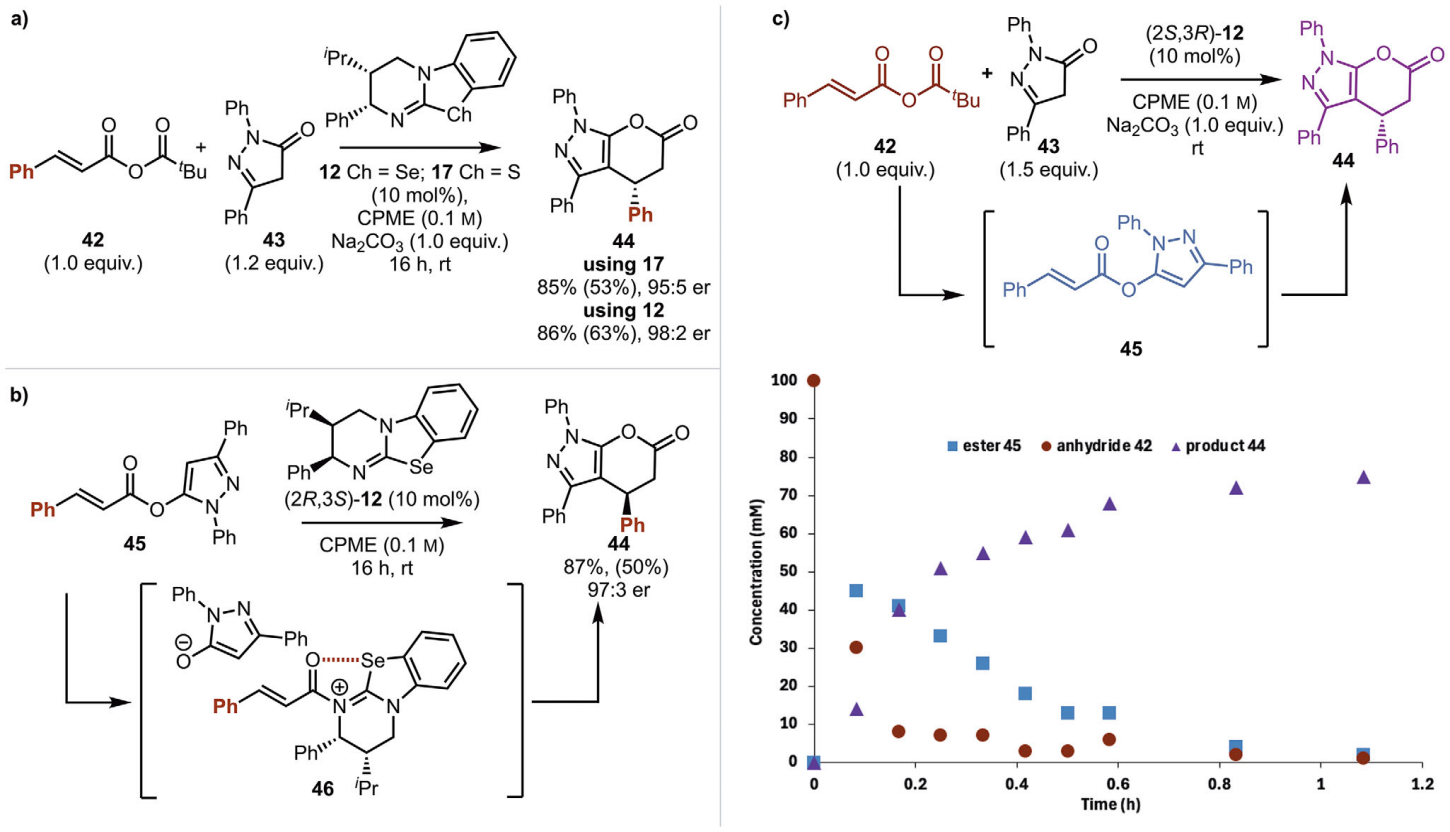
Yields are shown as NMR yields, calculated using 1,3,5‐trimethoxybenzene as an internal standard, with isolated yields in parentheses. Enantiomeric ratio measured by HPLC analysis on a chiral stationary phase. a) Optimization of pyrazolone lactone products. b) Demonstration of a productive reaction pathway from an ester intermediate. Note: The opposite enantiomer of catalyst **12** was used. c) Reaction profile analysis.

**Scheme 5:**
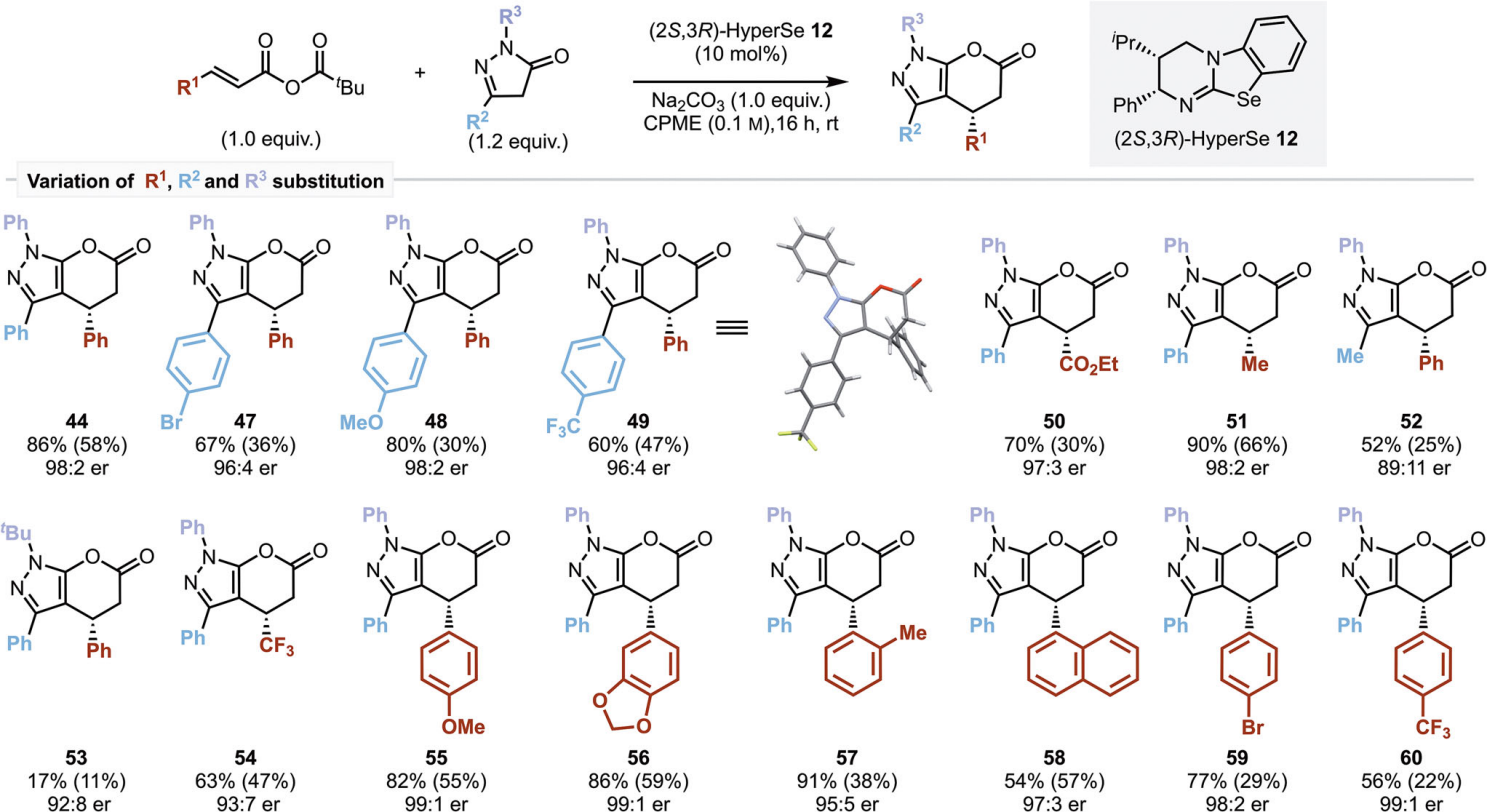
Scope of the [3 + 3]‐Michael addition cyclization approach to pyrazolopyranones. Yields are shown as NMR yields, calculated using 1,3,5‐trimethoxybenzene as an internal standard, with isolated yields in parentheses; enantiomeric ratios are measured by HPLC analysis on a chiral stationary phase.

The extension of this protocol toward the generation of pyrazolone lactone products was next investigated.^[^ ^36^ ^]^ Optimization (see Section S2.4–S2.5 for full details) using pyrazolone **43** as a pronucleophile showed that, unlike in the previous series, cinnamic anhydrides proved optimal. Comparative use of 10 mol% (2*S*,3*R*)‐HyperSe **12** gave product **44** in slightly higher conversion and enantioselectivity than using (2*R*,3*S*)‐HyperBTM **17**, with the use of Na_2_CO_3_ as a base giving optimal product conversion in CPME (0.1 m). However, a significant inherent limitation in this series was the instability of the lactone products to chromatographic purification on silica, leading to significant product loss and reduced isolated yields compared to the product yield indicated by ^1^H NMR spectroscopic analysis of the crude product mixture. Attempts to derivatize these products directly by ring‐opening with methanol prior to their isolation proved problematic, leading to a complex mixture of product tautomers that could not be fully characterized. Therefore, the optimized conditions treated cinnamic anhydride **42** with an excess of the pyrazolone **43** to afford **44** in 86% NMR yield (63% isolated yield) with 98:2 er (Scheme 4a). Notably, pyrazolyl ester **45** could be readily prepared, and its constitution was confirmed unambiguously by X‐ray crystallographic analysis (see Section S8 for details).^[^ ^35^ ^]^ Upon treatment of ester **45** with 10 mol% (2*R*,3*S*)‐HyperSe **12** in CPME, the heterocyclic product **44** was generated in 87% NMR yield (50% isolated) and 97:3 er (Scheme 4b). Consistent with the observations in the amide series, the conversion of the ester product **45** into the corresponding enantioenriched product **44** indicates that the formation of **45** within the Michael addition‐annulation must be reversible. Furthermore, in situ monitoring of the reaction of anhydride **42** and pyrazolone **43** with 10 mol% (2*S*,3*R*)‐**12** showed initial rapid anhydride consumption that corresponded to the formation of the 1,2‐addition ester **45** (maximum concentration of ∼45 mM) and its subsequent consumption to give heterocycle **44**, with high product conversion observed after 3 h (∼77 mM) (Scheme 4, 5).

The generality of the developed process was next investigated, with initial studies focused upon variation of the pyrazolone R^2^ and R^3^ substituents. R^2^‐aryl substituent variation showed that the incorporation of halogen (4‐BrC_6_H_4_), electron‐donating (4‐MeOC_6_H_4_) and electron‐withdrawing (4‐F_3_CC_6_H_4_) substituents were tolerated, giving products **47**–**49** with high enantioselectivity (up to 98:2 er) but significantly reduced isolated yield (30%–47%). With methyl substitution at R^2^, reduced conversion and product enantioselectivity were observed, giving **52** in 89:11 er and 52% NMR yield (25% isolated). A limitation of this process involved the incorporation of a sterically encumbered *tert*‐butyl substituent at R^3^, leading to significantly reduced reactivity, giving **53** in 17% NMR yield and 92:8 er. The incorporation of alkyl as well as aryl substituents at R^1^ was also tolerated, giving methyl‐substituted **51** in 98:2 er. Electron‐donating, 2‐substituted, extended aromatic, and electron‐withdrawing substituents were all tolerated, giving **54**–**60** with excellent enantioselectivity (>93:7 er).

**Scheme 6:**
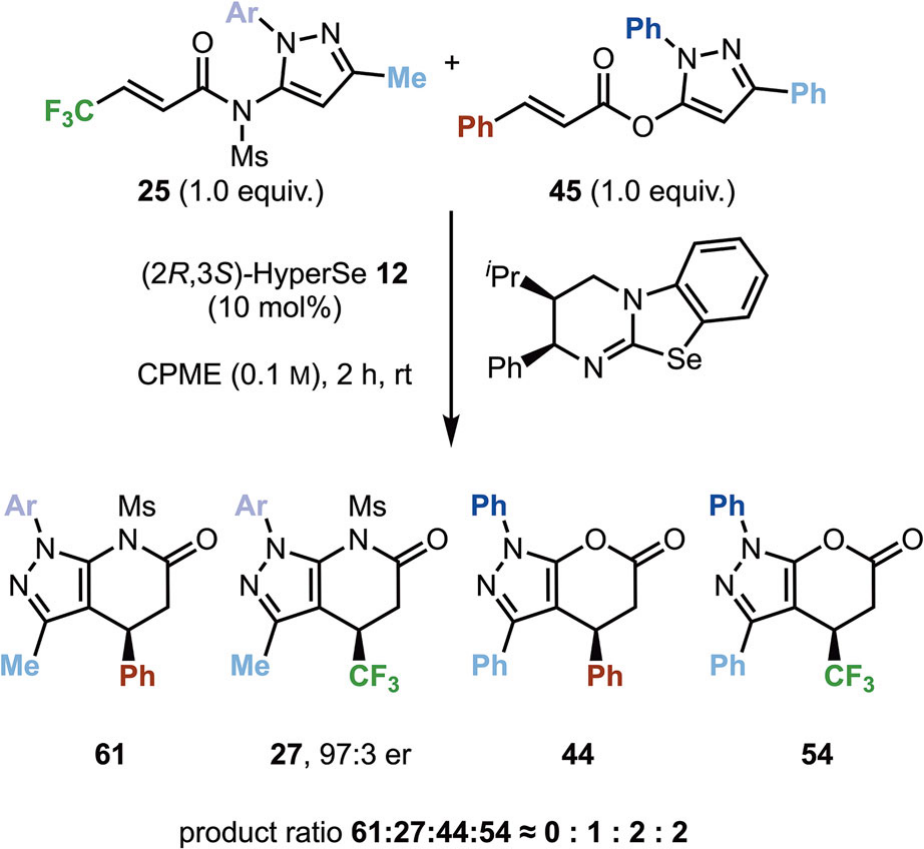
Crossover experiment between **25** and **45** with (2*R*,3*S*)‐HyperSe **12**. Note: The opposite enantiomer of catalyst **12** was used. Ar = −MeOC_6_H_4_. Ratio determined by ^1^H NMR using 1,3,5‐trimethoxybenzene as internal standard. Remaining yields are attributed to starting materials.

To further probe the mechanism of this process, a crossover experiment was performed utilizing ester **45** (1.0 equiv.) and amide **25** (1.0 equiv.) with the Lewis base (2*R*,3*S*)‐HyperSe **12** (10 mol %) (Scheme 6). After 2 h, the formation of three of the four possible cyclized heterocyclic products (**27**, **44**, and **54**) was identified by ^1^H NMR spectroscopic analysis in a ≈1:2:2 ratio. In line with observations from defining the scope and limitations of this methodology, where β‐aryl substituted anhydrides were unsuccessful in generating fused heterocyclic products with pyrazolylsulfonamides, the formation of product **61** was not observed. Due to poor HPLC separation of the mixture, only the enantioselectivity of **27** (97:3 er) could be readily identified but is indicative of the expected enantiocontrol in this process. The formation of these three products from two starting materials is indicative of significant crossover in the reaction process.

**Scheme 7:**
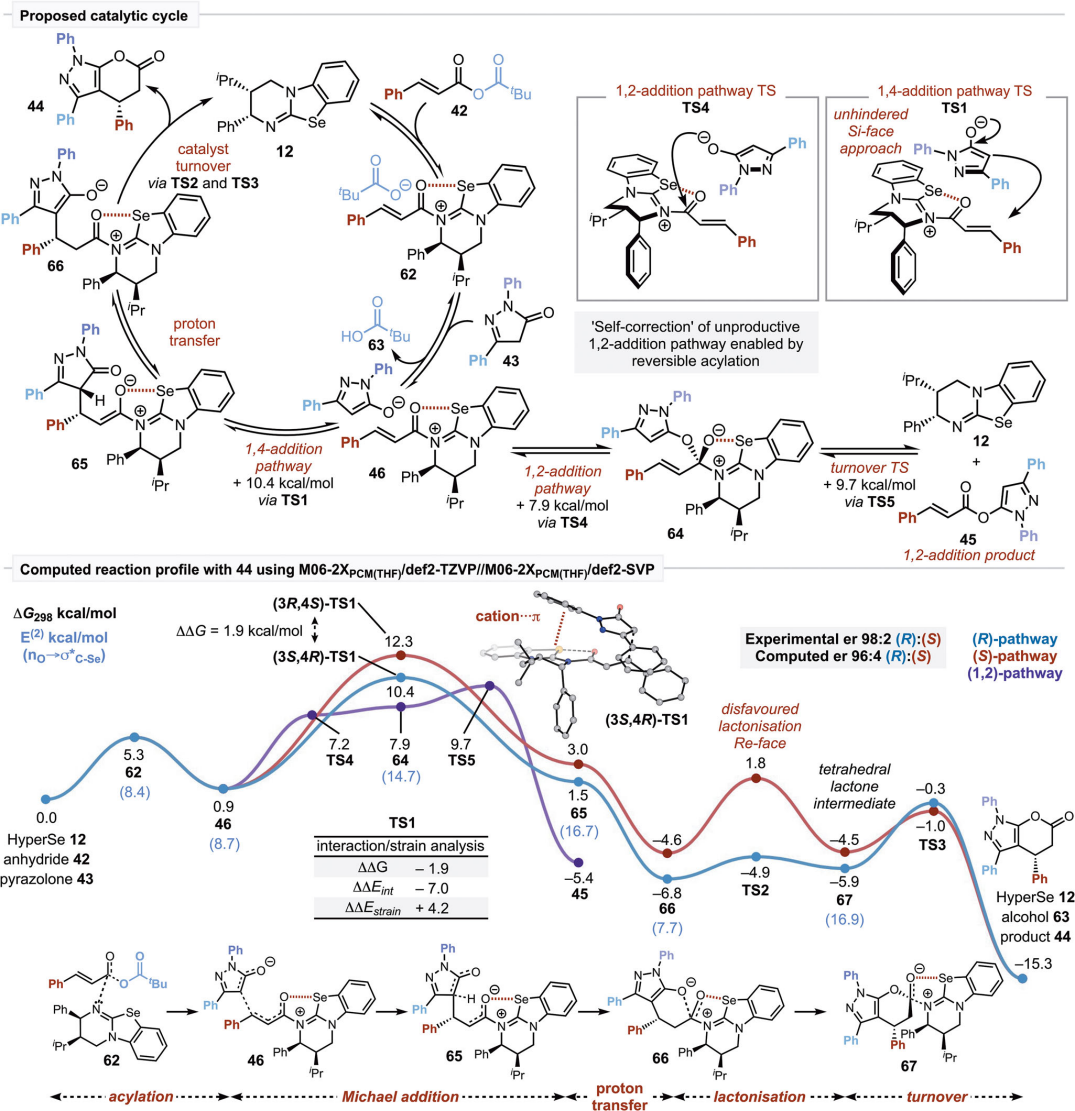
Proposed catalytic cycle DFT analysis of the pathways leading to enantiomeric products. Key transition states are highlighted with interaction/strain analysis on the stereodetermining transition states (see Section S6.3 for details). The chalcogen interaction strength is described by NBO second‐order stabilization energies (*E* ^2^) shown in lilac in kcal mol^−1^. M06‐2X_PCM(THF)_/def2‐TZVP//M06‐2X_PCM(THF)_/def2‐SVP Gibbs free energies (ΔG_298_) are shown in kcal mol^−1^.

Building upon these synthetic and mechanistic observations, DFT computations at the M06‐2X_PCM(THF)_/def2‐TZVP//M06‐2X_PCM(THF)_/def2‐SVP level of theory with Gaussian16 were performed using the pyrazolone substrate **43** to correlate with observed intermediates and understand the factors leading to the observed high levels of enantioselectivity.^[^ ^37^, ^38^, ^39^, ^40^, ^41^ ^]^ THF was used to model solvation instead of CPME, which was used experimentally. Both are ethereal solvents with similarly low dielectric constants, and PCM parameters were not available for CPME. Initially, reversible *N‐*acylation of the isoselenourea **12** by the pivaloyl mixed anhydride **42** affords the α,β‐unsaturated acyl isoselenouronium carboxylate ion pair **62**. Proton exchange with the pyrazolone pronucleophile **43** generates pivalic acid **63** and the isoselenouronium pyrazole ion pair **46**, from which several reaction pathways are available. Reversible 1,2‐addition with the nucleophilic *O*‐heteroatom via **TS4** leads to the observable amide or ester intermediate, with isoselenourea acylation capable of regenerating the reactive α,β‐unsaturated acyl isoselenouronium ion pair **46**.^[^ ^42^ ^]^ Alternatively, productive stereoselective 1,4‐addition via the nucleophilic carbon of the pyrazolone (X = OH) pronucleophile **43** provides the corresponding C(1)‐isoselenouronium enolate **65** (via **TS1**). Proton transfer leads to destruction of the newly formed pyrazolone‐based stereocenter to generate zwitterionic intermediate **66**, with subsequent 1,2‐addition (via **TS2**) enabling catalyst turnover to afford the corresponding enantioenriched lactone product **44**. After *N*‐acylation, **62** is formed in an *s*‐*cis* conformation, stabilized by an intramolecular 1,5‐chalcogen interaction (*n*_O_→σ*_C‐Se_, *E* ^(2)^ = 8.4 kcal mol^−1^, obtained from second‐order perturbation analysis). Deprotonation of the pronucleophile by the carboxylate is exergonic by 4.4 kcal mol^−1^, leading to a more stabilized and reactive acyl ammonium nucleophile ion‐pair (**46**, Scheme 7). From this species, unproductive, direct 1,2‐addition is favored over Michael addition, with an overall barrier of 9.7 kcal mol^−1^ (via **TS4**) and a driving force of −5.4 kcal mol^−1^, initially populating the reaction with the 1,2‐product (**45**). Due to the low driving force and barrier height, the formation of the 1,2‐product can be considered reversible, and the subsequent productive formation of the Michael adduct proceeds with a computed barrier of 15.8 kcal mol^−1^, (via **TS1** from **45** through **46**).

**Figure 1:**
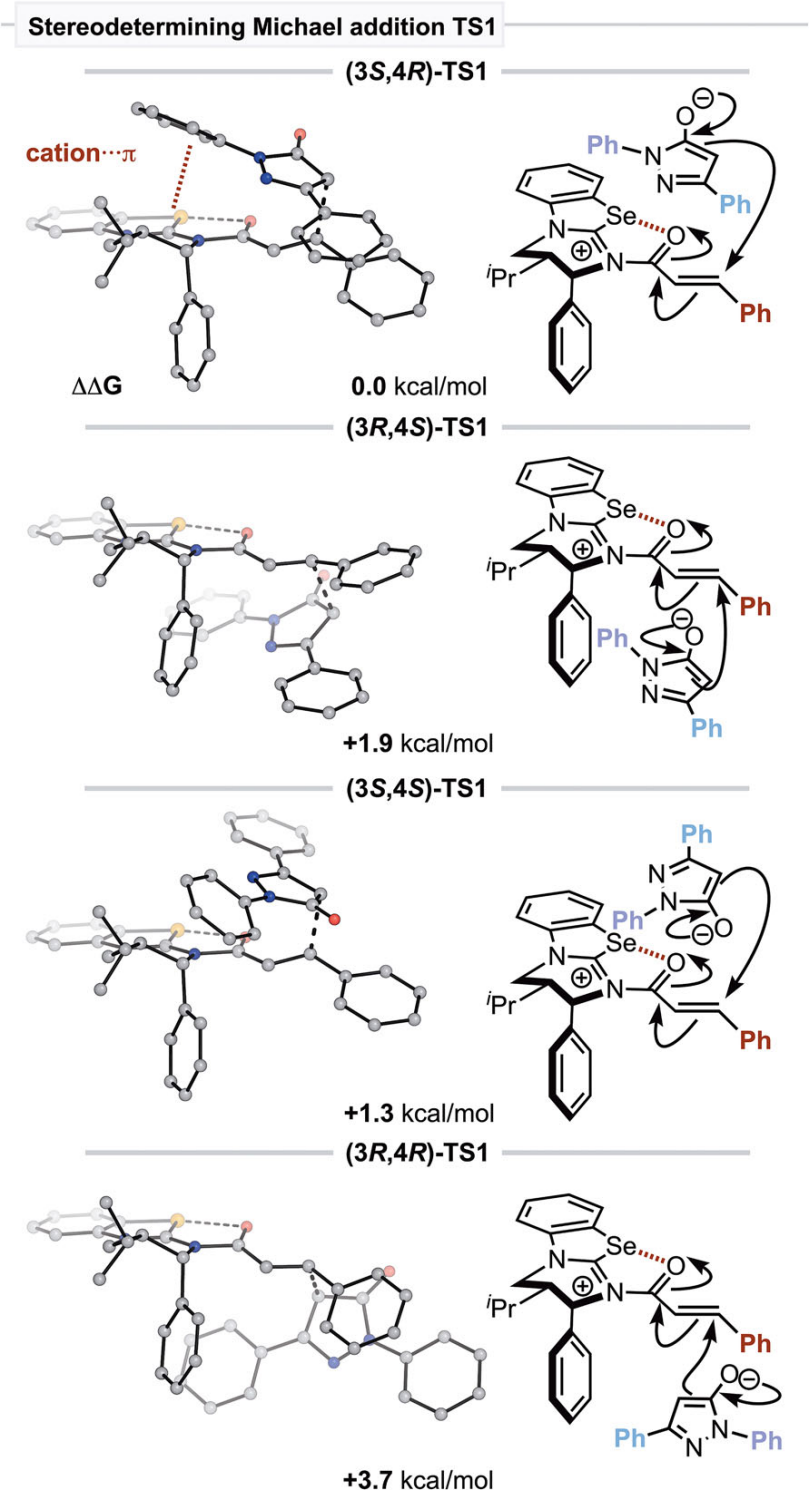
Comparison of the diastereomeric transition states in the stereodetermining Michael addition transition state. Relative free energies (ΔΔ^‡^ *G*) in kcal mol^−1^.

The diastereoselectivity of the Michael addition into the α,β‐unsaturated acyl isoselenouronium intermediate **46** was probed by considering the possible staggered conformations of this bond, forming Michael addition reaction (Figure 1, see Section S6.3). Similar to work by Wei and co‐workers,^[^ ^43^ ^]^ computation indicated that addition to the relatively unhindered *Si*‐face of the α,β‐unsaturated acyl isoselenouronium ion is preferred, with diastereoselective formation of (3*S*,4*R*)‐**TS1** favored over (3*S*,4*S*)‐**TS1** by ΔΔ^‡^ *G* = 1.3 kcal mol^−1^ (Figure 1). Preferential orientation of the pronucleophile in (3*S*,4*R*)‐**TS1** leads to stabilization via a cation‐π‐type interaction unlike (3*S*,4*S*)‐**TS1**. Michael addition upon the more hindered *Re*‐face of the α,β‐unsaturated acyl isoselenouronium intermediate is relatively disfavored with ΔΔ^‡^ *G* = 1.9 and 3.7 kcal mol^−1^ for the formation of (3*R*,4*S*)‐**TS1** and (3*R*,4*R*)‐**TS1**, respectively. After the Michael addition, proton transfer is calculated to be exergonic by 7.8 kcal mol^−1^, leading to two diastereoisomeric intermediates after the destruction of the transient pyrazolone stereocenter (for clarity, only the lowest diastereomeric transition states for each approach toward the catalyst are shown in Scheme 7). Following the formation of **66**, ring closing via lactonization leads to a tetrahedral lactone‐containing intermediate **67**, where the catalyst remains bound to the ring‐closed product. This may be stabilized by a strengthened chalcogen interaction relative to **66** as the charged oxygen species can act as a stronger donor toward the σ*_C‐Se_ acceptor orbital (*E* ^(2)^ = 16.9 kcal mol^−1^). Upon reformation of the carbonyl component of the product, the isoselenourea catalyst is regenerated. Enantioselectivity is governed by the stereodetermining Michael addition TS, with (3*S*,4*R*)‐**TS1** favored by ΔΔ^‡^ *G* = 1.9 kcal mol^−1^ relative to (3*R*,4*S*)‐**TS1**, in excellent agreement with the experimentally observed 98:2 er (computed 96:4 er). Through the activation strain model,^[^ ^44^ ^]^ calculations indicate that the selectivity arises due to favorable interactions between the substrate and catalyst in the (3*S*,4*R*)‐**TS1** (ΔΔ^‡^ *E*_int_ = −7.0 kcal mol^−1^), though this is a more strained orientation than the (3*R*,4*S*)‐**TS1** approach (ΔΔ^‡^ *E*_strain_ = +4.2 kcal mol^−1^). This implies that in this step, the stereodirecting phenyl substituent is limiting the geometry of the substrate such that it cannot benefit from stabilizing interactions with the isoselenouronium ion. Contrastingly, the lactonization TS (**TS2**) is strongly disfavored for the (*S*)‐pathway (ΔΔ^‡^ *G*  =  6.7 kcal/mol), due to the less favorable angle of attack (100.1° compared to 108.0°) imposed by the stereodirecting phenyl substituent. As a result, the orbital overlap is reduced, and the interaction in **TS2** is significantly lower (ΔΔ^‡^ *E*_int_ = +21.9 kcal mol^−1^).

## Conclusion

In conclusion, an operationally simple isoselenourea catalyzed formal [3 + 3] Michael addition‐cyclization has been described, giving access to a structurally diverse array of pyrazolo‐pyridinones and ‐pyranones in moderate to high yields and generally excellent enantioselectivity. Both series are tolerant to electronic and steric variation of the nucleophilic and electrophilic reaction components, with the pyranone series tolerant of β‐aryl and β‐alkyl anhydride substitution. The reaction can be readily carried out on a 2‐g scale with high catalyst recovery, with subsequent product derivatizations maintaining the high enantioselectivity. Mechanistic investigations indicate the isoselenourea preference derives from interception of an intermediate amide or ester derived from initial 1,2‐addition of the pronucleophile that diverts these products to a productive catalytic pathway. Computational studies in the pyrazolone series found that the stereodetermining Michael addition step occurs via preferential *Re*‐addition of the nucleophile to the α,β‐unsaturated acyl isoselenouronium ion that is favored by stabilizing cation‐π type interactions that are not available with Michael addition toward the *Si*‐face.

## Supporting Information

The authors have cited additional references within the Supporting Information.^[^ ^14^, ^45^, ^46^, ^47^, ^48^, ^49^, ^50^, ^51^, ^52^, ^53^, ^54^, ^55^, ^56^, ^57^, ^58^, ^59^, ^60^, ^61^, ^62^, ^63^, ^64^, ^65^, ^66^, ^67^, ^68^, ^69^, ^70^, ^71^, ^72^, ^73^, ^74^ ^]^

## Author Contributions

M.I.P. and M.T.W. performed the synthetic work, A.S.G. carried out the computational studies, A.P.M. and D.B.C. performed the X‐ray crystallography work. M.I.P., M.T.W., and A.S.G. co‐wrote the manuscript and E.S.I., A.D.S. oversaw the project and wrote the manuscript.

## Conflict of Interests

The authors declare no conflict of interest.

## Supporting information



Supporting Information

## Data Availability

All of the experimental procedures, characterization, NMR, HPLC spectra, details of the computational studies and crystallography data can be found in the ESI. The research data supporting this publication can be accessed from “Isoselenourea‐Catalysed Enantioselective Pyrazolo‐heterocycle Synthesis Enabled By Self‐correcting Amide and Ester Acylation”, University of St Andrews Research Portal, https://doi.org/10.17630/d5ff6e0d‐b94b‐4c7b‐bf77‐b5ecbbc5c195.
