# Supplementary material for: Isoselenourea‐Catalyzed Enantioselective Pyrazolo‐Heterocycle Synthesis Enabled by Self‐Correcting Amide and Ester Acylation
Source: Angew Chem Int Ed Engl. 2025 Mar 31;64(19):e202425305. doi: 10.1002/anie.202425305 (PMC12051791; doi:10.1002/anie.202425305)
Supplement: Supplementary file 1 — Supporting Information [file ANIE-64-e202425305-s001.pdf]

Isoselenourea-Catalyzed Enantioselective Pyrazolo-Heterocycle Synthesis Enabled By Self-Correcting Amide and Ester Acylation

Martha I. Prindl, Matthew T. Westwood, Alister S. Goodfellow, Aidan P. McKay, David B. Cordes, Michael Bühl, Andrew D. Smith

Table of Contents

1.General Experimental 4

2.Optimisation........ 6

2.1.Pyrazole Amine Aryl Ester Conjugate Addition Optimisation 6

2.2.Pyrazole Amine Mixed Anhydride Optimisation........ 7

2.3.Pyrazole Amine Mixed Anhydride Optimisation with β-boryl substitution 8

2.4.Pyrazolone Conjugate Addition Optimisation 9

2.5.Pyrazolone Conjugate Addition 10

3.General Procedures 11

3.1.General Procedure 1 - Ethyl Ester Hydrolysis........ 11

3.2.General Procedure 2 – Anhydride Formation 11

3.3.General Procedure 3 – Pyrazole Formation 11

3.4.General procedure 4 – Pyrazole Dimesylation 12

3.5.General procedure 5 – Pyrazole Demesylation........ 12

3.6.General Procedure 6 – Pyrazolone Formation 12

3.7.General procedure 7 - Pyrazole Amine Conjugate Addition........ 13

3.8.General procedure 8 – Pyrazolone Conjugate Addition........ 13

3.9.General procedure 9 –1,2-Addition Products 13

4.Unsuccessful substrates within the aminopyrazole approach to pyrazolopyridinones 14

5.Compound Synthesis 15

5.1.Mixed Anhydride Synthesis........ 15

5.2.Pyrazole Synthesis 24

5.3.Pyrazolone Synthesis 33

5.4.Pyrazole Conjugate Addition Products 36

5.5.Product Derivatizations........ 44

5.6.Pyrazolone Conjugate Addition Products 47

5.7.1,2-Addition Product Synthesis 56

5.8.Mechanistic investigation of 1,2-addition substrate........ 58

5.9.Cross-over Experiment ... 59
6.Computational Supporting Information ... 60
6.1.Computational Details ... 60
6.2.Interaction and Reorganisation Energies ... 60
6.3.Staggered Conformations of TS-MA ... 61
6.4.Formation of 1,2-Addition Product ... 61
7.Computational Data ... 63
8.Crystallographic Details ... 97
9.References ... 99
10.NMR Spectra ... 100
11.HPLC data ... 290

1. General Experimental

Reactions involving moisture sensitive reagents were carried out in flame-dried glassware under an inert atmosphere (N_2) using standard vacuum line techniques. Anhydrous solvents (tetrahydrofuran and toluene) were obtained after passing through an alumina column (Mbraun SPS-800). Petrol is defined as petroleum ether 40–60 °C. All other solvents and commercial reagents were used as received without further purification.

Room temperature (rt) refers to 15–20 °C. Temperatures of 0 °C were obtained using ice/water bath. Reaction involving heating were performed using DrySyn blocks and a contact thermocouple. Under reduced pressure refers to the use of either a Büchi Rotavapor R-200 with a Büchi V-491 heating bath and Büchi V-800 vacuum controller, a Büchi Rotavapor R-210 with a Büchi V-491 heating bath and Büchi V-850 vacuum controller, a Heidolph Laborota 4001 with vacuum controller, an IKA RV10 rotary evaporator with a IKA HB10 heating bath and ILMVAC vacuum controller, or an IKA RV10 rotary evaporator with a IKA HB10 heating bath and Vacuubrand CVC3000 vacuum controller. Rotary evaporator condensers are fitted to Julabo FL601 Recirculating Coolers filled with ethylene glycol and set to –6 °C.

Analytical thin layer chromatography was performed on pre-coated aluminium plates (Kieselgel 60 F254 silica) purchased from Merck and visualisation was achieved using ultraviolet light (254 nm) and/or staining with aqueous $KMnO_4$ solution followed by heating. Manual column chromatography was performed in glass columns fitted with porosity 3 sintered discs over Kieselgel 60 silica using the solvent system stated. Automated chromatography was performed on a Biotage Selekt Four running Biotage OS578 with a UV/Vis detector using the method stated and cartridges filled with Kieselgel 60 silica.

Melting points were recorded on an Electrothermal 9100 melting point apparatus and are not corrected, (*dec*) refers to decomposition.

Optical rotations were measured on a Perkin Elmer Precisely/Model-341 polarimeter operating at the sodium D line with a 100 mm path cell at 20 °C.

HPLC analyses were obtained on either a Shimadzu HPLC consisting of a DGU-20A_5 degassing unit, LC-20AT liquid chromatography pump, SIL-20AHT autosampler, CMB-20A communications bus module, SPD-M20A diode array detector and a CTO-20A column oven or a Shimadzu HPLC consisting of a DGU-20A_{5R} degassing unit, LC-20AD liquid chromatography pump, SIL-20AHT autosampler, SPD-20A UV/Vis detector and a CTO-20A column oven. Separation was achieved using either DAICEL CHIRALCEL OD-H columns or DAICEL CHIRALPAK AD-H, IA, IB, and IC columns using the method stated. HPLC traces of enantiomerically enriched compounds were compared with spectra

of an authentic racemic sample prepared from a corresponding reaction using either DHPB (10 mol%) or (±)-HyperBTM (10 mol%).

Infrared spectra were recorded on a Shimadzu IRAffinity-1 Fourier transform IR spectrophotometer fitted with a Specac Quest ATR accessory (diamond puck). Spectra were recorded of either thin films or solids, with characteristic absorption wavenumbers (ν_{max}) reported in cm^{-1}.

^{1}H, $^{13}C\{^{1}H\}$4, $^{11}B\{^{1}H\}$ and $^{19}F\{^{1}H\}$ NMR spectra were acquired on either a Bruker AV300 with a BBFO probe, a Bruker AV400 with a BBFO probe, a Bruker AVII 400 with a BBFO probe, a Bruker AVIII-HD 500 with a SmartProbe BBFO+ probe, a Bruker AVIII 500 with a CryoProbe Prodigy BBO probe in the deuterated solvent stated. Deuterated solvents were purchased and used as received. All chemical shifts are quoted in parts per million (ppm) relative to the residual solvent peak. All coupling constants *J* are quoted in Hz. Multiplicities are indicated as s (singlet), d (doublet), t (triplet), q (quartet), m (multiplet), and multiples thereof. The abbreviation Ar denotes aromatic and app denotes apparent. NMR peak assignments were confirmed using 2D ^{1}H correlated spectroscopy (COSY), 2D $^{1}H–^{13}C$ heteronuclear multiple-bond correlation spectroscopy (HMBC), and 2D $^{1}H–^{13}C$ heteronuclear single quantum coherence (HSQC) where necessary.

Mass spectrometry (*m/z*) data were acquired by electrospray ionisation (ESI) either at the University of St Andrews Mass Spectrometry Facility or at the University of Edinburgh Mass Spectrometry Facility.

2. Optimisation

2.1. Pyrazole Amine Aryl Ester Conjugate Addition Optimisation

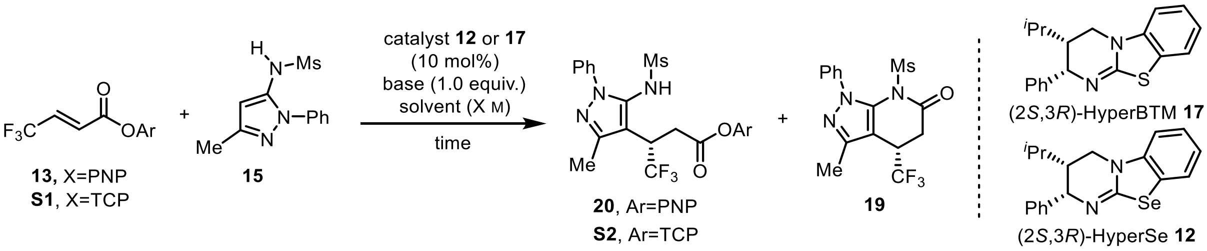

Entry	13 or S1:15 (equiv.)	OAr	Base	Catalyst	Solvent	Time	20 or S2 (%)	19 (%)	19 er
1	1.2:1	PNP	*i*-Pr_2NEt	HyperBTM **17**	MeCN	16	16	66	80:20
2	1.2:1	PNP	$NaHCO_3$	HyperBTM **17**	MeCN	16	28	56	85:15
3	1.2:1	TCP	$NaHCO_3$	HyperBTM **17**	MeCN	16	25	55	87:13
4	1.2:1	PNP	$NaHCO_3$	HyperBTM **17**	THF	16	21	39	87:13
5	1.2:1	PNP	$NaHCO_3$	HyperBTM **17**	EtOAc	16	20	41	88:12
6	1.2:1	PNP	None	HyperBTM **17**	MeCN	16	36	59	88:12
7	1.2:1	PNP	None	HyperSe **12**	MeCN	16	39	61	92:8
8	1.2:1	PNP	None	HyperSe **12**	Acetone	16	30	70	92:8
9	1.2:1	PNP	None	HyperSe **12**	Dioxane	16	14	48	98:2
10[a]	1.2:1	PNP	None	HyperSe **12**	Dioxane	24	17	53	98:2
11	1.2:1	PNP	None	HyperSe **12**	Dioxane	24	9	43	98:2
12	1.2:1	PNP	none	HyperSe **12**	Dioxane	48	10	45	98:2

Table 1: Optimisation of pyrazole amine conjugate addition. Yields were determined using 1,3,5-trimethoxybenzene as the internal standard. Enantiomeric ratios were measured by HPLC analysis using a chiral stationary phase; a) Reaction conducted at 0.2 M concentration. PNP = p-$O_2NC_6H_4$. TCP = 2,4,6-$Cl_3C_6H_2$

2.2. Pyrazole Amine Mixed Anhydride Optimisation

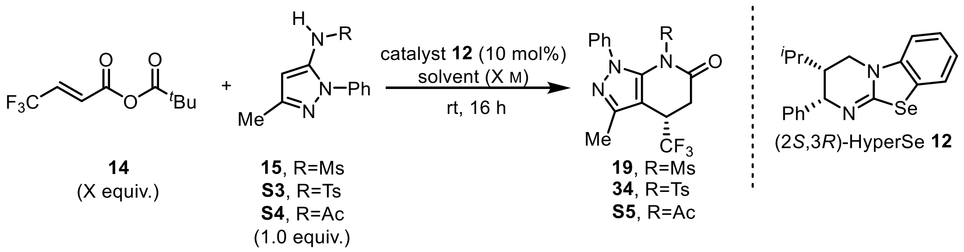

Entry	R =	14 (equiv.)	Solvent (M)	Catalyst (mol%)	Yield of 19. 34 or S5 (%)	19, 34 or S5 er
1	Ms	1.2	MeCN (0.1)	HyperSe **12** (10)	75	91:9
2	Ms	1.2	Dioxane (0.1)	HyperSe **12** (10)	65	98:2
3	Ms	1.2	MeCN + Dioxane (1:1, 0.1)	HyperSe **12** (10)	73	92:8
4	Ms	1.2	Acetone (0.1)	HyperSe **12** (10)	50	97:3
5	Ms	1.2	CPME (0.1)	HyperSe **12** (10)	90	99:1
6	Ms	1.2	CPME (0.2)	HyperSe **12** (10)	85	98:2
7	Ms	1.0	CPME (0.1)	HyperSe **12** (10)	70	-
**8**	**Ms**	**1.5**	**CPME (0.1)**	**HyperSe 12 (10)**	**95**	**99:1**
9	Ts	1.5	CPME (0.1)	HyperSe **12** (10)	92	97:3
10	Ac	1.5	CPME (0.1)	HyperSe **12** (10)	N/A	N/A
11	Ms	1.5	CPME (0.1)	HyperSe **12** (5)	70	99:1
13[a]	Ms	1.5	CPME (0.1)	HyperSe **12** (5)	99	99:1
12	Ms	1.5	CPME (0.1)	None	N/A	N/A

Table 2: Optimisation of the pyrazole amine reaction. a) Reacted for 24 hours. The yields stated are isolated. Enantiomeric ratios measured by HPLC analysis using a chiral stationary phase.

2.3. Pyrazole Amine Mixed Anhydride Optimisation with β-boryl substitution

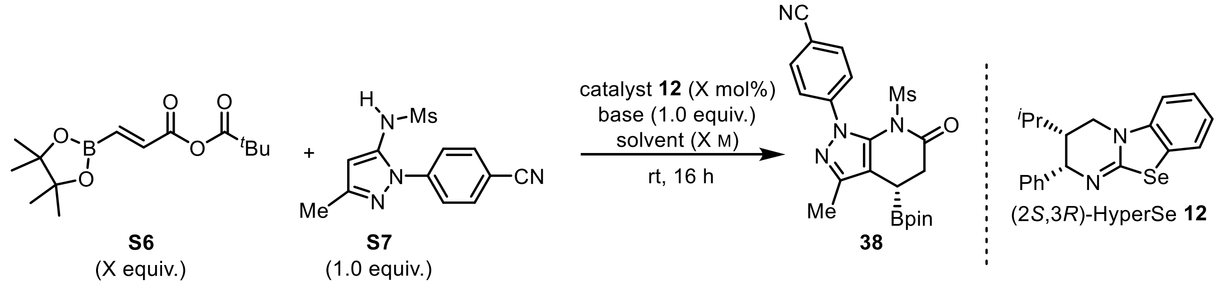

Entry	S6 (equiv.)	S7 (equiv.)	Base	Solvent (M)	Catalyst (mol%)	Yield of 38 (%)	38 er
1	1.5	1.0	Na_2CO_3	CPME (0.1)	HyperSe **12** (10)	Trace	N/A
2	1.5	1.0	Na_2CO_3	MeCN (0.1)	HyperSe **12** (10)	63 (10)	61:39
3	1.5	1.0	Na_2CO_3	1,4-dioxane (0.1)	HyperSe **12** (10)	44 (22)	81:29
4	1.5	1.0	K_2CO_3	1,4-dioxane (0.1)	HyperSe **12** (10)	40 (11)	85:15
5	1.5	1.0	K_2CO_3	1,4-dioxane (0.1)	HyperSe **12** (20)	75 (30)	81:19
6	1.0	2.0	K_2CO_3	1,4-dioxane (0.1)	HyperSe **12** (10)	44 (16)	89:11

Table 3:Optimisation of the pyrazole amine conjugate addition with boron substitution. Yields were determined using 1,3,5-triemthoxy benzene as internal standard with isolated yields in brackets. Enantiomeric ratios were measured by HPLC analysis using a chiral stationary phase

2.4. Pyrazolone Conjugate Addition Optimisation

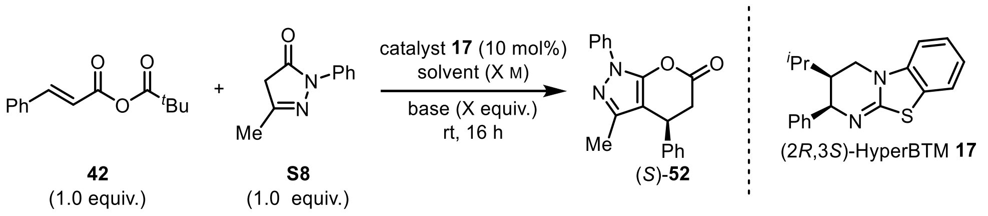

Entry	Base (equiv.)	Solvent (M)	Catalyst (mol%)	NMR yield (%)	Yield of 52 (%)	52 er
1	Et_3N (1.0)	MeCN (0.1)	HyperBTM **17** (10)	-	25	66:34
2	$^iPr_2NEt$ (1.0)	MeCN (0.1)	HyperBTM **17** (10)	-	34	66:34
3	Na_2CO_3 (1.0)	MeCN (0.1)	HyperBTM **17** (10)	-	23	68:32
4	Na_2CO_3 (1.0)	MeCN (0.1)	None	N/A	N/A	N/A
5	Et_3N (1.0)	CH_2Cl_2 (0.1)	HyperBTM **17** (10)	N/A	N/A	N/A
6	Et_3N (1.0)	PhMe (0.1)	HyperBTM **17** (10)	62	13	67:33
7	Et_3N (1.0)	THF (0.1)	HyperBTM **17** (10)	73	30	62:38
8	Et_3N (1.0)	DMF (0.1)	HyperBTM **17** (10)	42	22	56:44
9	Et_3N (1.0)	EtOAc (0.1)	HyperBTM **17** (10)	60	17	61:39
10	Et_3N (1.0)	Et_2O (0.1)	HyperBTM **17** (10)	13	N/A	N/A
11	Et_3N (1.0)	CPME (0.1)	HyperBTM **17** (10)	54	18	72:28

Table 4: Initial optimisation of the pyrazolone conjugate addition reaction. NMR yields were determined using 1,3,5-trimethoxy benzene as the internal standard. Enantiomeric ratios measured by HPLC analysis using a chiral stationary phase.

2.5. Pyrazolone Conjugate Addition

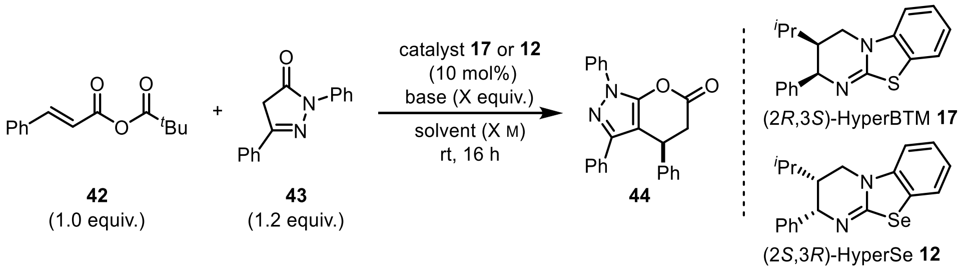

Entry	Base (equiv.)	Solvent (M)	Catalyst (mol%)	NMR yield (%)	Yield of 44 (%)	44 er
12	Et_3N (1.0)	CPME (0.1)	HyperBTM **17** (10)	50	43	95:5
14	Et_3N (1.0)	MeCN (0.1)	HyperBTM **17** (10)	79	50	85:15
15	Et_3N (1.0)	PhMe (0.1)	HyperBTM **17** (10)	40	21	92:8
17	Et_3N (1.0)	TBME (0.1)	HyperBTM **17** (10)	37	24	93:7
18	Et_3N (1.0)	DME (0.1)	HyperBTM **17** (10)	47	17	92:8
19	Et_3N (1.0)	2-Methyl THF	HyperBTM **17** (10)	46	29	93:7
20	Et_3N (1.0)	Dioxane (0.1)	HyperBTM **17** (10)	N/A	-	-
21[c]	Et_3N (1.0)	CPME (0.1)	HyperBTM **17** (10)	47	34	96:4
22	Et_3N (2.0)	CPME (0.1)	HyperBTM **17** (10)	45	28	94:6
23	Na_2CO_3 (1.0)	CPME (0.1)	HyperBTM **17** (10)	85	53	95:5
24	K_2CO_3 (1.0)	CPME (0.1)	HyperBTM **17** (10)	34	21	85:15
25[b]	Na_2CO_3 (1.0)	CPME (0.1)	HyperBTM **17** (10)	71	52	95:5
26[a]	Na_2CO_3 (1.0)	CPME (0.1)	HyperBTM **17** (10)	75	46	93:7
27	Na_2CO_3 (1.0)	CPME (0.1)	HyperBTM **17** (5)	50	43	96:4
**28**	**Na_2CO_3 (1.0)**	**CPME (0.1)**	**HyperSe 12 (10)**	**93**	**52**	**98:2**
29	Na_2CO_3 (1.0)	CPME (0.2)	HyperSe **12** (10)	69	40	93:7
30	Na_2CO_3 (1.0)	CPME (0.05)	HypeSe **12** (10)	79	46	96:4

*Table 5: Optimisation of the pyrazolone conjugate addition reaction. a) 24-hour reaction; b) Reaction at 0 °C; c) 1.0 equivalents of pyrazolone **43**. NMR yields were determined using 1,3,5-trimethoxy benzene as the internal standard. Enantiomeric ratio measured by HPLC analysis using a chiral stationary phase.*

3. General Procedures

3.1. General Procedure 1 - Ethyl Ester Hydrolysis

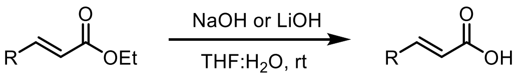

To a solution of the appropriate ester (1.0 equiv.) in THF (0.75 M), NaOH (1.2 equiv.) or LiOH (3.0 equiv.) and H_2O (0.75 M) were added. The mixture was stirred at room temperature until starting material consumption was observed *via* TLC, and then the mixture was concentrated under reduced pressure to remove THF. The solution was acidified with 1 M aq. HCl and extracted with Et_2O (3 ×), the organic phases were combined, washed (brine), dried (Na_2SO_4) and then concentrated under reduced pressure to afford the crude product which was used without further purification.

3.2. General Procedure 2 – Anhydride Formation

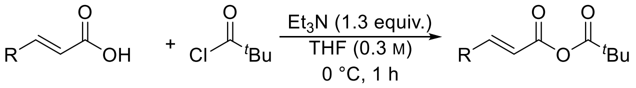

Pivaloyl chloride (1.2 equiv.) was added dropwise to a solution of the appropriate carboxylic acid (1.0 equiv.) and Et_3N (1.3 equiv.) in anhydrous THF (0.3 M) at 0 °C under N_2. The mixture was stirred at 0 °C for 1 h, filtered with Et_2O, and the filtrate was concentrated under reduced pressure to give the product, which was used without further purification.

3.3. General Procedure 3 – Pyrazole Formation

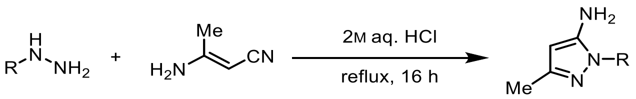

The appropriate hydrazine (1.0 equiv.), 3-aminocrotonitrile (1.0 equiv.) and 2 M aq. HCl (0.5 M) were heated at reflux for 16 h. The reaction was cooled to room temperature and basified with Na_2CO_3. The mixture was then extracted with EtOAc (3 ×), the organic phases were then combined, washed (brine), dried (Na_2SO_4) and concentrated under reduced pressure to afford the crude product which was purified as specified .

3.4. General procedure 4 – Pyrazole Dimesylation

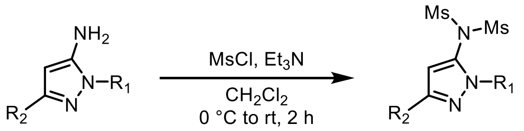

Methanesulfonyl chloride (3.0 equiv.) was added dropwise to a solution of the appropriate pyrazole (1.0 equiv.) and Et_3N (3.0 equiv.) in anhydrous CH_2Cl_2 (0.1 M) at 0 °C. The reaction mixture was allowed to warm to room temperature and stir for 2 hours. The mixture was then diluted with H_2O, extracted with CH_2Cl_2 (3 ×). The organic phases were then combined, washed (brine), dried (Na_2SO_4) and concentrated under reduced pressure to afford the crude product which was purified as specified.

3.5. General procedure 5 – Pyrazole Demesylation

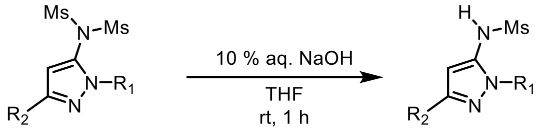

Following an adapted literature procedure, [45] aq. 10% NaOH (1.0 equiv.) was added portionwise to a solution of the appropriate dimesylated pyrazole (1.0 equiv.) in THF (0.3 M) and the resulting mixture was stirred at room temperature for 1 hour. The solution was then acidified with 1 M aq. HCl and concentrated under reduced pressure to remove THF. The mixture was then extracted with CH_2Cl_2 (3 ×), the organic phases were then combined, washed (brine), dried (Na_2SO_4) and concentrated under reduced pressure to afford the crude product which was used without further purification.

3.6. General Procedure 6 – Pyrazolone Formation

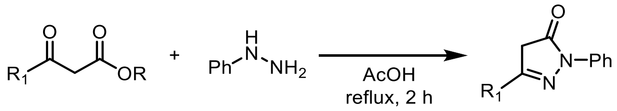

Following an adapted literature procedure, [46] the appropriate *β*-keto ester (1.0 equiv.) and phenyl hydrazine (1.0 equiv.) in glacial acetic acid (5.0 M) were heated at reflux (130 °C) for 2 h. The solution was diluted with Et_2O (1.0 M) and stirred for 30 min at 0 °C. The precipitate was washed with Et_2O and collected *via* vacuum filtration to afford the product which was used without further purification.

3.7. General procedure 7 - Pyrazole Amine Conjugate Addition

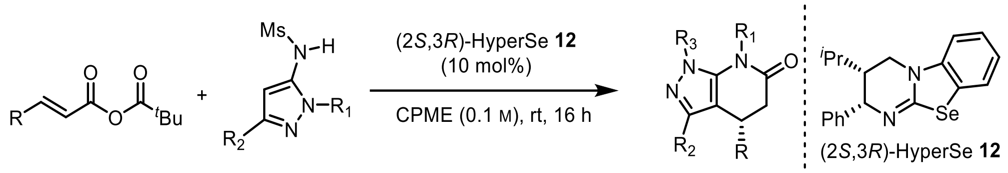

The appropriate anhydride (1.5 equiv.), (2*S*,3*R*)-HyperSe **12** (0.01 equiv.) and CPME (0.1 M) were combined in a vial. The appropriate aminopyrazole (1.0 equiv.) was then added and the reaction mixture stirred at room temperature for 16 h. The mixture was concentrated under reduced pressure and the crude product was purified as specified. The internal standard 1,3,5-trimethoxy benzene (0.33 equiv.) was used to determine the conversion to product by ^{1}H NMR.

3.8. General procedure 8 – Pyrazolone Conjugate Addition

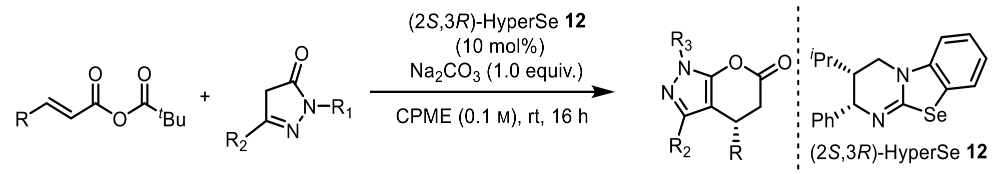

The appropriate pyrazolone (1.2 equiv.), anhydride (1.0 equiv.), (2*S*,3*R*)-HyperSe **12** (10 mol%) and CPME (0.1 M) were combined in a vial. Na_2CO_3 (1.0 equiv.) was added and the reaction mixture stirred at room temperature for 16 h. The mixture was concentrated under reduced pressure and purified as specified. The internal standard 1,3,5-trimethoxy benzene (0.33 equiv.) was used to determine the conversion to product by ^{1}H NMR.

3.9. General procedure 9 –1,2-Addition Products

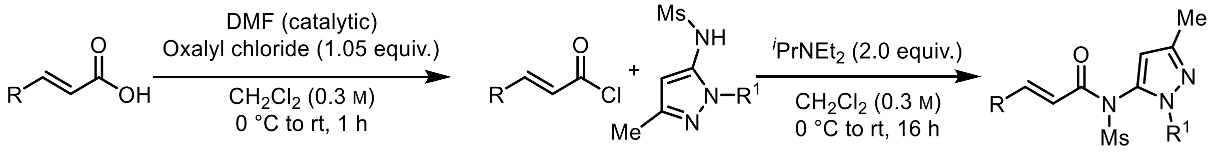

Following literature [47]. and under N_2, the appropriate acid (1.0 equiv.) and DMF (catalytic amount) were dissolved in CH_2Cl_2 (0.3 M) and set to 0 °C. Oxalyl chloride (1.05 equiv.) was added dropwise and the mixture was stirred at rt for 1 h until gas evolution stopped. The acyl chloride solution was then added dropwise to a solution of the appropriate aminopyrazole (1.0 equiv.) and $^{i}Pr_2NEt$ (2.0 equiv.) in CH_2Cl_2 (0.3 M) at 0 °C. The mixture was stirred at rt for 16 h and then concentrated under reduced pressure to afford the crude product which was purified by Biotage® Selekt™.

4. Unsuccessful substrates within the aminopyrazole approach to pyrazolopyridinones

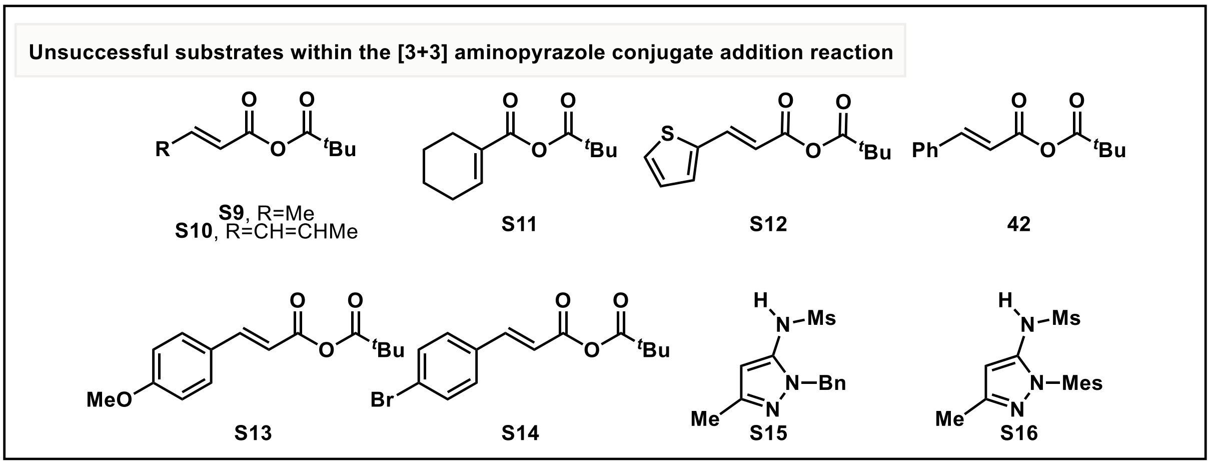

5. Compound Synthesis

5.1. Mixed Anhydride Synthesis

(*E*)-4,4,4-trifluorobut-2-enoic acid **S17**

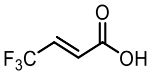

S17

Following general procedure **1**, ethyl (*E*)-4,4,4-trifluorobut-2-enoate (4.48 mL, 30 mmol), THF (40 mL), NaOH (1.42 g, 36 mmol) and H_2O (30 mL) for 3 h, gave the title compound **S17** (4.79 g, *quant.*) as a colourless solid with all spectroscopic data in accordance to literature. [34] **mp** 56–58 °C {Lit. [34] 52-55 °C}; **IR** ν_{max} (solid) 3088, 2992, 1699, 1126, 914; **^{1}H NMR** (400 MHz, $CDCl_3$): δ 6.89 (dq, 1H, J = 15.8, 6.4 Hz), 6.52 (dq, 1H, J = 15.8, 1.9 Hz); **^{19}F{^{1}H} NMR** (377 MHz, $CDCl_3$): δ –65.83 (s, 3F).

(*E*)-(*E*)-4,4,4-trifluorobut-2-enoic pivalic anhydride **14**

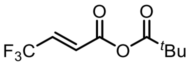

14

Following general procedure **2**, (*E*)-4,4,4-trifluorobut-2-enoic acid **S17** (4.20 g, 30 mmol), Et_3N (5.64 mL, 39 mmol), THF (100 mL) and pivaloyl chloride (4.43 mL, 36 mmol) for 1 h, gave the title compound **14** (3.31 g, 49%) as a colourless oil. **IR** ν_{max} (film) 2980, 1809, 1740, 1001; **^{1}H NMR** (400 MHz, $CDCl_3$): δ 6.85 (dq, 1H, J = 15.8, 6.4 Hz), 6.52 (dq, 1H, J = 15.8, 1.9 Hz), 1.30 (s, 9H); **^{13}C{^{1}H} NMR** (126 MHz, $CDCl_3$): δ 172.8, 159.8, 134.0 (q, $^2J_{CF}$ = 36.1 Hz), 128.0 (q, $^3J_{CF}$ = 6.1 Hz), 122.7 (q, $^1J_{CF}$ = 270.8 Hz), 40.4, 26.5; **^{19}F{^{1}H} NMR** (377 MHz, $CDCl_3$): δ –65.7 (s); **HRMS** (ESI+) $C_9H_{11}F_3O_3Na$ $[M+Na]^+$ found 247.0544, requires 247.0558 (–5.7 ppm).

(*E*)-4-ethoxy-4-oxobut-2-enoic pivalic anhydride (*E*)-**S18**

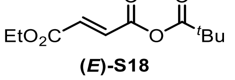

(*E*)-S18

Following general procedure **2**, (*E*)-4-ethoxy-4-oxobut-2-enoic acid (0.72 g, 5 mmol), THF (17 mL), Et_3N (0.94 mL, 6.5 mmol) and pivaloyl chloride (0.74 mL, 6.0 mmol), for 1h gave the title compound **S18** (1.0 g, 88%) as a pale-yellow oil. **IR** ν_{max} (film) 2980, 1807, 1726, 1207, 1045, 1009; **^{1}H NMR** (500 MHz, $CDCl_3$): δ 6.91 (d, 1H, J = 15.8 Hz), 6.84 (d, 1H, J = 15.8 Hz), 4.29 (q, 2H, J = 7.1 Hz), 1.34 (t, 3H, J = 7.1 Hz), 1.30 (s, 9H); **^{13}C{^{1}H} NMR** (101 MHz, $CDCl_3$): δ 173.2, 164.5, 160.9, 136.4, 132.3, 61.9, 40.3, 26.6, 14.2; **HRMS** (ESI^+) $C_{11}H_{16}O_5Na$ $[M+Na]^+$ found 251.0885, requires 251.0895 (–2.1 ppm).

(*Z*)-4-ethoxy-4-oxobut-2-enoic pivalic anhydride (*Z*)-**S18**

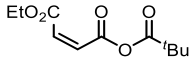

(*Z*)-S18

Following general procedure **2**, ethyl hydrogen maleate (0.72 g, 5.0 mmol), Et_3N (0.94 mL, 6.5 mmol), THF (17 mL) and pivaloyl chloride (0.74 mL, 6.0 mmol) for 1 h, gave the title compound **S18** (0.87 g, 77%) as a pale pink oil. **IR** ν_{max} (film) 2980, 1807, 1726, 1638, 1221, 1043, 1001; **^{1}H NMR** (400 MHz, $CDCl_3$): δ 6.31 (s, 2H), 4.26 (q, 2H, J = 7.2 Hz), 1.31 (t, 3H, J = 7.2 Hz), 1.26 (s, 9H); **^{13}C{^{1}H} NMR** (126 MHz, $CDCl_3$): δ 173.5, 164.6, 161.2, 130.8, 129.1, 61.8, 40.2, 26.5, 14.1; **HRMS** (ESI$^+$) $C_{11}H_{16}O_5Na$ $[M+Na]^+$ found 251.0886, requires 251.0895 (−1.5 ppm).

Tert-butyl ethyl fumarate **S19**

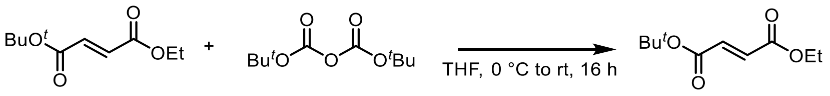

S19

Following a literature procedure [34], di-*tert*-butyl dicarbonate (4.36 g, 20 mmol, 1.25 equiv.) and DMAP (0.39 g, 3.2 mmol, 0.2 equiv.) were added to a solution of monoethyl fumarate (2.29 g, 16 mmol, 1.0 equiv.) in THF (24 mL) at 0 °C. The mixture was stirred at rt for 16 h, diluted with EtOAc (25 mL) and washed with 10% sulfuric acid (50 mL), 1 M NaOH (50 mL) and brine. The organic layer was dried over Na_2SO_4 and concentrated under reduced pressure. The crude product was then columned using Biotage® Selekt™ [100 g, 120 mL.min^{-1}, Petrol : EtOAc (100:0 to 60:40 20CV)] to afford the title compound **S19** as a colourless oil (1.21 g, 38%) with all spectroscopic data in accordance to literature. **IR** ν_{max} (film) 2980, 1715, 1294, 1258, 1142; **^{1}H NMR** (400 MHz, $CDCl_3$): δ 6.78 (d, 1H, J = 15.8 Hz), 6.74 (d, 1H, J = 15.8 Hz), 4.25 (q, 2H, J = 7.2 Hz), 1.50 (s, 9H), 1.31 (t, 3H, J = 7.2 Hz).

(*E*)-4-(*tert*-butoxy)-4-oxobut-2-enoic acid **S20**

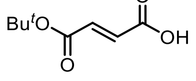

S20

Following general procedure **1**, *tert*-butyl ethyl fumarate **S19** (1.0 g, 5 mmol), THF (2.5 mL), LiOH (0.23 g, 5.5 mmol) and water (2.5 mL) for 16 h, gave the title compound **S20** (0.17 g, 20%) as a white solid with all spectroscopic data in accordance to literature. [34] **mp** 59–62 °C{Lit. [34] 61–64 °C (CH_2Cl_2)}; **IR** ν_{max} (film) 3074, 2982, 1682, 1269, 1159 (C-O);**^{1}H NMR** (400 MHz, $CDCl_3$): δ 6.87 (d, 1H, J =15.8 Hz), 6.75 (d, 1H, J = 15.8 Hz), 1.52 (9H, s).

(E)-(E)-4-(*tert*-butoxy)-4-oxobut-2-enoic pivalic anhydride **S21**

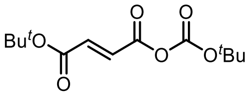

S21

Following general procedure **2**, (E)-4-(*tert*-butoxy)-4-oxobut-2-enoic acid **S20** (0.34 g, 1.97 mmol), Et_3N (0.37 mL, 2.36 mmol), THF (6.5 mL) and pivaloyl chloride (0.29 mL, 2.56 mmol) for 1 h, gave the title compound **S21** (0.22 g, 44%) as a yellow oil. **IR** ν_{max} (film) 2978, 1807, 1717, 1043, 1001, 976; **^{1}H NMR** (400 MHz, $CDCl_3$): δ 6.83 (d, 1H, J = 15.7 Hz), 6.75 (d, 1H, J = 15.8 Hz), 1.52 (s, 9H), 1.29 (s, 9H); **^{13}C{^{1}H} NMR** (126 MHz, $CDCl_3$): δ 173.3, 163.6, 161.1, 138.5, 131.4, 82.7, 40.3, 28.1, 26.6; **HRMS** (ESI$^+$) $C_{13}H_{20}O_5Na$ [M+Na]$^+$ found 279.1202, requires 279.1203 (–0.2 ppm).

Ethyl (E)-4-oxo-4-(pyrrolidin-1-yl)but-2-enoate **S22**

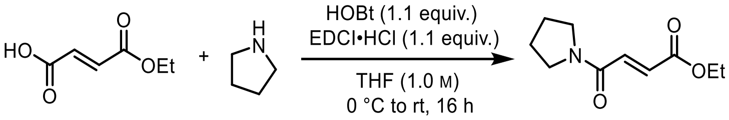

S22

Following literature [48], (E)-4-ethoxy-4-oxobut-2-enoic acid (1.59 g, 11 mmol, 1.1 equiv.), pyrrolidine (0.83 mL, 10 mmol, 1.0 equiv.), THF (10 mL) and HOBt (1.49 g, 11 mmol, 1.1 equiv.) were combined in a round bottom flask. The reaction mixture was set to 0 °C and EDCI (2.11 g, 11 mmol, 1.1 equiv.) was added in portions. The reaction was left to stir over 16 h at rt and then concentrated under reduced pressure to remove THF. The solution was diluted with EtOAc (50 mL) and the organic layer was washed with ammonium chloride (3 × 50 mL), $NaHCO_3$ (3 × 50 mL) and brine (50 mL). The organic layers were combined, dried over $MgSO_4$, filtered, and the filtrate was concentrated under reduced pressure to afford the crude product which was purified on Biotage® Selekt™ [Sfär HC 25 g, 80 mL.min^{-1}, Petrol : EtOAc (60:40 to 20:80 18 CV, 20:80 to 10:90 10 CV)] to afford the title compound **S22** (0.77 g, 39%) as a colourless oil with all spectroscopic data in accordance to literature. **IR** ν_{max} (film) 2978, 1721, 1651, 1620, 1269, 1161; **^{1}H NMR** (400 MHz, $CDCl_3$): δ 7.24 (d, 1H, J = 15.3 Hz), 6.85 (d, 1H, J = 15.3 Hz), 4.25 (d, 2H, J = 7.2 Hz), 3.61–3.54 (m, 4H), 2.03–1.96 (m, 2H), 1.94–1.87 (m, 2H), 1.31 (t, 3H, J = 7.2 Hz).

(E)-4-oxo-4-(pyrrolidine-1-yl)but-2-enoic acid **S23**

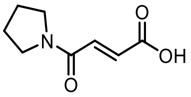

S23

Following general procedure **1**, ethyl (E)-4-oxo-(pyrrolidin-1-yl)but-2-enoate **S22** (0.73 g, 3.68 mmol) in THF (6 mL), aq. NaOH (176.6 mg , 5.00 mmol) and H_2O (4 mL) for 4 h, gave the title compound

S23 (0.33 g, 53%) as a white solid with all spectroscopic data in accordance to literature. [48] **mp** 177–179 °C{Lit. [48] 162–164 °C (MeOH)}; **IR** ν_{max} (film) 1716, 1662, 1589, 1260; **^{1}H NMR** (500 MHz, $CDCl_3$): δ 7.31 (d, 1H, J = 15.3 Hz), 6.89 (d, 1H, J = 15.3 Hz), 3.60 (dt, 4H, J = 14.2, 6.9 Hz), 2.01 (p, 2H, J = 6.8 Hz), 1.92 (p, 2H, J = 6.9 Hz).

(*E*)-(*E*)-4-oxo-4-(pyrrolidin-1-yl)but-2-enoic pivalic anhydride **S24**

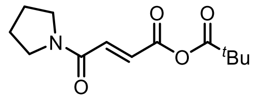

S24

Following general procedure **2**, (*E*)-4-oxo-4-(pyrrolidin-1-yl)but-2-enoic acid **S23** (0.33 g, 1.96 mmol), THF (6.5 mL), Et_3N (0.36 mL, 2.55 mmol) and pivaloyl chloride (0.29 mL, 2.35 mmol), for 1 h gave the title compound **S24** (0.37 g, 76%) as a pale brown solid. **mp** 47–48 °C; **IR** ν_{max} (film) 2974, 1801, 1732, 1654, 1620, 1047, 1015; **^{1}H NMR** (400 MHz, $CDCl_3$): δ 7.34 (d, 1H, J = 15.2 Hz), 6.86 (d, 1H, J = 15.3 Hz), 3.62–3.56 (m, 4H), 2.06–1.99 (m, 2H), 1.96–1.89 (m, 2H), 1.29 (s, 9H); **^{13}C{^{1}H} NMR** (101 MHz, $CDCl_3$): δ 173.5, 161.9, 161.8, 137.7, 129.5, 47.0, 46.5, 40.2, 26.6, 26.2, 24.4; **HRMS** (ESI^+) $C_{11}H_{19}NO_4Na$ $[M+Na]^+$ found 276.1206, requires 276.1212 (–1.6 ppm).

(*E*)-(*E*)-4-oxo-4-(*p*-tolyl)but-2-enoic pivalic anhydride **S25**

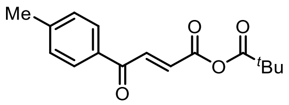

S25

Following general procedure **2**, (*E*)-4-oxo-4-(*p*-tolyl)but-2-enoic acid (0.95 g, 5.0 mmol), Et_3N (0.94 mL, 6.5 mmol), THF (17 mL) and pivaloyl chloride (0.74 mL, 6.0 mmol) for 1 h, gave the title compound **S25** (0.80 g, 72%) as a yellow solid. **mp** 40–42 °C; **IR** ν_{max} (film) 2978, 1803, 1734, 1670, 1045, 1007; **^{1}H NMR** (400 MHz, $CDCl_3$): δ 7.98 (d, 1H, J = 15.5 Hz), 7.90 (d, 2H, J = 8.2 Hz), 7.33 (d, 2H, J = 8.0 Hz), 6.87 (d, 1H, J = 15.5 Hz), 2.45 (s, 3H), 1.32 (s, 9H); **^{13}C{^{1}H} NMR** (126 MHz, $CDCl_3$): δ 188.5, 173.5, 161.7, 145.6, 139.5, 134.0, 130.7, 129.9, 129.2, 40.3, 26.6, 22.0; **HRMS** (ESI^+) $C_{16}H_{18}O_4Na$ $[M+Na]^+$ found 297.1097, requires 297.1103 (–0.04 ppm).

(*E*)-(*E*)-4-oxo-4-phenylbut-2-enoic pivalic anhydride **S26**

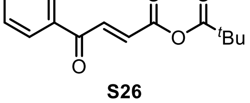

S26

Following general procedure **2**, (*E*)-4-oxo-4-phenylbut-2-enoic acid (0.79 g, 4.5 mmol), Et_3N (0.85 mL, 5.85 mmol), THF (15 mL) and pivaloyl chloride (0.66 mL, 5.4 mmol) for 1 h, gave the title compound **S26** (0.83 g, 71%) as an orange solid. **mp** 45–46 °C; **IR** ν_{max} (film) 2976, 1805, 1734, 1042, 995; **^{1}H**

NMR (400 MHz, $CDCl_3$): δ 8.01–7.97 (m, 3H), 7.66 (app t, 1H, J =7.4 Hz), 7.54 (app t, 2H, J = 7.7 Hz), 6.88 (d, 1H, J = 15.5 Hz), 1.32 (s, 9H); **^{13}C{^{1}H} NMR** (126 MHz, $CDCl_3$): δ 189.0, 173.4, 161.6, 139.2, 136.4, 134.4, 131.1, 129.2, 129.1, 40.3, 26.6; **HRMS** (ESI$^+$) $C_{15}H_{16}O_4Na$ $[M+Na]^+$ found 283.0944, requires 283.0941 (+1.0 ppm).

Ethyl (E)-3-(4,4,5,5-tetramethyl-1,3,2-dioxaborolan-2-yl)acrylate **S27**

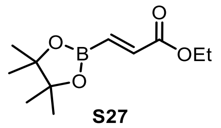

S27

Following literature [49]. CuCl (118.8 mg, 1.2 mmol, 0.03 equiv.), NaO^{t}Bu (230.6 mg, 2.4 mmol, 0.06 equiv.) and Xantphos (694.3 mg, 1.2 mmol, 0.03 equiv.) were added to a 2-neck round bottom flask and backfilled with nitrogen. THF (40 mL) was added and the reaction was stirred under N_2 for 1 h. Bis(pinacolato)diborane (11.2 g, 44 mmol, 1.1 equiv.) in THF (28 mL) was introduced to the reaction and stirred for 10 mins after which ethyl propiolate (4.1 mL, 40 mmol, 1.0 equiv.) and MeOH (3.2 mL) were added. The orange-brown reaction mixture was then stirred at rt for 20 h after which it was washed and filtered through a celite pad with ether. The crude product was filtered with petrol and concentrated under reduced pressure. The product was then purified via Biotage® Selekt™ [Sfär HC 50g, 120 mL.min^{-1}, Petrol : Et_2O (100:0 to 50:50 10 CV, 50:50 10 CV)] to afford the title compound **S27** (4.6 g, 51%) as a colourless oil with all spectroscopic data in accordance to literature. **IR** ν_{max} (film); 2980, 1724, 1348, 1142; **^{1}H NMR** (500 MHz, $CDCl_3$): δ 6.77 (d, 1H, J = 18.2 Hz), 6.62 (d, 1H, J = 18.2 Hz), 4.21 (q, 2H, J = 7.1 Hz), 1.30−1.26 (m, 15H); **^{11}B{^{1}H} NMR** (128 MHz, $CDCl_3$): δ 29.91 (s).

(E)-3-(4,4,5,5-tetramethyl-1,3,2-dioxaborolan-2-yl)acrylic acid **S28**

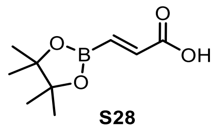

S28

Following general procedure **1**, ethyl (*E*)-3-(4,4,5,5-tetramethyl-1,3,2-dioxaborolan-2-yl)acrylate **S27** (2.87 g, 12.7 mmol), THF (20 mL), LiOH (0.91 g, 38.1 mmol) and H_2O (10 mL) for 16 h, gave the title compound **S28** (1.96 g, 78%) as a white solid. **mp** 79–83 °C; **IR** ν_{max} (film) 2978, 1694, 1333; **^{1}H NMR** (400 MHz, $CDCl_3$): δ 6.88 (d, 1H, J = 18.2 Hz), 6.62 (d, 1H, J = 18.2 Hz), 1.29 (s, 12H); **^{13}C{^{1}H} NMR** (126 MHz, $CDCl_3$): δ 171.0, 137.9, 84.4, 24.9; **^{11}B{^{1}H} NMR** (128 MHz, $CDCl_3$): δ 30.38 (s); **HRMS** (ESI$^-$) $C_9H_{14}O_4{}^{11}B$ $[M-H]^-$ found 197.0994, requires 197.0991 (+1.5 ppm).

(E)-(E)-3-(4,4,5,5-tetramethyl-1,3,2-dioxaborolan-2-yl)acrylic pivalic anhydride **S6**

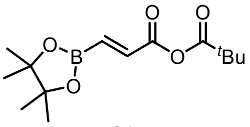

S6

Following general procedure **2**, (*E*)-3-(4,4,5,5-tetramethyl-1,3,2-dioxaborolan-2-yl)acrylic acid **S28** (0.99 g, 5.0 mmol), Et_3N (0.94 mL, 6.5 mmol), THF (17 mL) and pivaloyl chloride (0.74 mL, 6.0 mmol), for 1 h, gave the title compound **S6** (1.31 g, 93%) as a colourless oil. **IR** ν_{max} (film) 2980, 1803, 1734, 1344, 1047, 1007; **^{1}H NMR** (400 MHz, $CDCl_3$): δ 6.87 (d, 1H, J = 18.1 Hz), 6.61 (d, 1H, J = 18.1 Hz), 1.30 (s, 12H), 1.28 (s, 9H); **^{13}C{^{1}H} NMR** (126 MHz, $CDCl_3$): δ 173.9, 161.6, 137.3, 84.5, 46.0, 26.7, 24.9; **^{11}B{^{1}H} NMR** (128 MHz, $CDCl_3$): δ 29.41 (s); **HRMS** (ESI^+) $C_{14}H_{23}BO_3Na$ $[M+Na]^+$ found 305.1525, requires 305.1531 (−1.9 ppm).

(E)-Cinnamic pivalic anhydride **42**

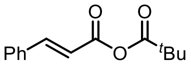

42

Following general procedure **2**, cinnamic acid (1.48 g, 10.0 mmol), THF (33 mL), Et_3N (1.88 mL, 13.0 mmol) and pivaloyl chloride (1.48 mL, 12.0 mmol) for 1 h, gave the title compound **42** (2.34 g, *quant.*) as a colourless oil with all the spectroscopic data in accordance with literature. [50] **IR** ν_{max} (film) 2974, 1792, 1722, 1043; **^{1}H NMR** (300 MHz, $CDCl_3$): δ 7.77 (d, 1H, J = 15.9 Hz), 7.57–7.54 (m, 2H), 7.47–7.38 (m, 3H), 6.45 (d, 1H, J = 15.9 Hz), 1.33 (s, 9H).

(*E*)-(*E*)-but-2-enoic pivalic anhydride **S9**

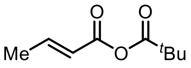

S9

Following general procedure **2**, crotonic acid (0.43 g, 5 mmol), THF (17 mL), Et_3N (0.94 mL, 6.5 mmol) and pivaloyl chloride (0.74 mL, 6.0 mmol) for 1 h, gave the title compound **S9** (0.67 g, 78%) as a brown oil with all the spectroscopic data in accordance to literature. [51] **IR** ν_{max} (film) 2976, 1798, 1730, 1651, 1051, 1016; **^{1}H NMR** (500 MHz, $CDCl_3$): δ 7.10 (dq, 1H, J = 14.1, 6.9 Hz), 5.89 (d, 1H, J = 15.5 Hz), 1.95 (d, 3H, J = 6.9 Hz), 1.28 (s, 9H).

(*E*)-4-Methoxycinnamic pivalic anhydride **S13**

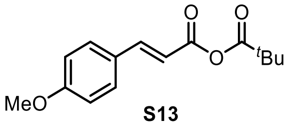

S13

Following general procedure **2**, 4-methoxycinnamic acid (1.78 g, 10.0 mmol), THF (33 mL), Et_3N (1.88 mL, 13.0 mmol) and pivaloyl chloride (1.48 mL, 12.0 mmol) for 1 h, gave the title compound **S13** (2.70 g, *quant.*) as an orange solid with all the spectroscopic data in accordance with the literature. [51] **mp** 56–58 °C; **IR** ν_{max} (solid) 1786, 1263, 1020; **^{1}H NMR** (400 MHz, $CDCl_3$): δ 7.72 (d, 1H, J = 15.6 Hz), 7.53–7.49 (m, 2H), 6.94–6.91 (m, 2H), 6.31 (d, 1H, J = 15.6 Hz), 3.86 (s, 3H), 1.32 (2, 9H).

(*E*)-3,4-Methylenedioxycinnamic pivalic anhydride **S29**

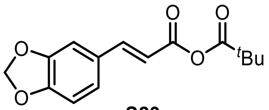

S29

Following general procedure **2**, 3,4-methylenedioxycinnamic acid (1.92 g, 10 mmol), THF (33 mL), Et_3N (1.88 mL, 13.0 mmol) and pivaloyl chloride (1.48 mL, 12.0 mmol) for 1 h, gave the title compound **S29** (2.83 g, *quant.*) as an orange solid. **mp** 86−88 °C; **IR** ν_{max} (solid) 1788, 1719, 1618, 1254, 1045, 1015, 984; **^{1}H NMR** (500 MHz, $CDCl_3$): δ 7.66 (d, 1H, J = 15.8 Hz), 7.05−7.03 (m, 2H), 6.83 (d, 1H, J = 8.2 Hz), 6.25 (d, 1H, J = 15.8 Hz), 6.03 (s, 2H), 1.32 (s, 9H); **^{13}C{^{1}H} NMR** (126 MHz, $CDCl_3$): δ 174.3, 163.0, 150.6, 148.7, 148.3, 128.2, 125.7, 114.7, 108.8, 106.8, 101.9, 40.1, 26.7; **HRMS** (ESI^+) $C_{15}H_{16}NaO_6$ $[M+Na]^+$ found 299.0884, requires 299.0890 (−2.0 ppm).

(*E*)-2-Methylcinnamic pivalic anhydride **S30**

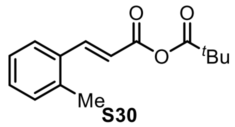

S30

Following general procedure **2**, 2-methycinnamic acid (0.24 g, 1.46 mmol), THF (5.0 mL), Et_3N (0.27 mL, 1.86 mmol) and pivaloyl chloride (0.21 mL, 1.71 mmol) for 1 h, gave the title compound **S30** (0.22 g, 60%) as a yellow oil. **IR** ν_{max} (film) 1724, 1793, 1011, 1045; **^{1}H NMR** (400 MHz, $CDCl_3$): δ 8.09 (d, 1H, J = 15.9 Hz), 7.60–7.57 (m, 1H), 7.35–7.31 (m, 1H), 7.26–7.22 (m, 2H), 6.37 (d, 1H, J = 15.9 Hz), 2.45 (s, 3H), 1.33 (s, 9H); **^{13}C{^{1}H} NMR** (101 MHz, $CDCl_3$): δ 174.2, 162.8, 146.2, 138.3, 132.7, 131.1, 126.8, 126.7, 117.8, 40.2, 26.8, 19.8; **HRMS** (ESI^+) $C_{15}H_{18}NaO_3$ $[M+Na]^+$ found 269.1139, requires 269.1148 (–3.4 ppm).

(*E*)-3-(naphthalen-1-yl)acrylic pivalic anhydride **S31**

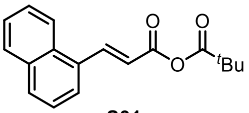

S31

Following general procedure **2**, (*E*)-3-(naphthalen-1-yl)acrylic acid (1.98 g, 10.0 mmol), THF (33 mL), Et_3N (1.88 mL, 13.0 mmol) and pivaloyl chloride (1.48 mL, 12.0 mmol) for 1 h, gave the title compound **S31** as an off-white solid (2.68 g, 95%). **mp** 52–53 °C; **IR** ν_{max} (solid) 2972, 1786, 1721, 1614, 1570, 1474; **^{1}H NMR** (400 MHz, $CDCl_3$): δ 8.64 (d, 1H J = 15.7 Hz), 8.20 – 8.13 (m, 1H), 7.99 – 7.92 (m, 1H), 7.93 – 7.88 (m, 1H), 7.86 – 7.78 (m, 1H), 7.65 – 7.48 (m, 3H), 6.56 (d, J = 15.7 Hz, 1H), 1.37 (s, 9H); **^{13}C{^{1}H} NMR** (101 MHz, $CDCl_3$): δ 174.3, 162.7, 145.5, 133.8, 131.7, 131.5, 131.0, 129.0, 127.4, 126.6, 125.8, 125.6, 123.1, 119.4, 40.2, 26.8; **HRMS** (ESI$^+$) $C_{18}H_{18}O_3Na$ $[M+Na]^+$found 305.1152, requires 305.1148 (+1.4 ppm).

(*E*)-4-Bromocinnamic pivalic anhydride **S14**

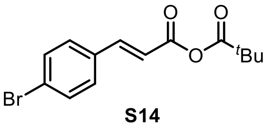

S14

Following general procedure **2**, 4-bromocinnamic acid (2.27 g, 10.0 mmol), THF (33 mL), Et_3N (1.88 mL, 13.0 mmol) and pivaloyl chloride (1.48 mL, 12.0 mmol), for 1 h, gave the title compound **S14** (2.99 g, 96%) as a yellow solid with all spectroscopic data in accordance to literature. [52] **mp** 54–56 °C; **IR** ν_{max} (film) 1794, 1007, 1045; **^{1}H NMR** (500 MHz, $CDCl_3$): δ 7.69 (d, 1H, J = 16.0 Hz), 7.55 (app d, 2H, J = 8.5 Hz), 7.42 (app d, 2H, J = 8.5 Hz), 6.43 (d, 1H, J = 16.0 Hz), 1.32 (s, 9H).

(*E*)-3-(4-(trifluoromethyl)phenyl)acrylic pivalic anhydride **S32**

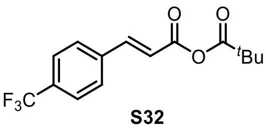

S32

Following general procedure **2**, (E)-3-(4-(trifluoromethyl)phenyl)acrylic acid (2.16 g, 10.0 mmol), THF (33 mL), Et_3N (1.88 mL, 13.0 mmol) and pivaloyl chloride (1.48 mL, 13.0 mmol) for 1 h, gave the title compound **S32** (2.79 g, 93%) as an off-white solid with all spectroscopic data in accordance with the literature. [51] **mp** 62–63 °C; **IR** ν_{max} (solid) 2982, 1780, 1717, 1632, 1323; **^{1}H NMR** (400 MHz, $CDCl_3$): δ 7.77 (d, 1H, J = 16.0 Hz), 7.71 – 7.63 (m, 4H), 6.52 (d, 1H, J = 16.0 Hz), 1.33 (s, 9H); **^{19}F{^{1}H} NMR** (376 MHz, $CDCl_3$): δ -62.96 (s).

(2*E*,4*E*)-hexa-2,4-dienoic pivalic anhydride **S10**

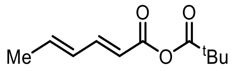

S10

Following general procedure **2**, sorbic acid (0.56 g, 5.0 mmol), Et_3N (0.94 mL, 6.5 mmol), THF (17 mL) and pivaloyl chloride (0.74 mL, 6.0 mmol) for 1 h, gave the title compound **S10** (0.98 g, 94%) as a light brown oil. **IR** ν_{max} (film) 2976, 1794, 1724, 1641, 1606, 1045, 1001; ^{1}**H NMR** (500 MHz, $CDCl_3$): δ 7.34–7.29 (m, 1H) 6.30–6.21 (m, 2H), 5.79 (d, 1H, J = 15.4 Hz), 1.89 (d, 3H, J = 5.5 Hz), 1.29 (s 9H); ^{13}**C{^{1}H} NMR** (126 MHz, $CDCl_3$): δ 174.4, 163.1, 148.9, 142.6, 129.7, 117.8, 40.1, 26.7, 19.0; **HRMS** (ESI^{+}) $C_{11}H_{16}O_3Na$ $[M+Na]^+$ found 219.0986, requires 219.0997 (–2.5 ppm).

Cyclohex-1-ene-1-carboxylic pivalic anhydride **S11**

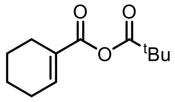

S11

Following general procedure **2**, cyclohex-1-ene-1-carboxylic acid (0.57 mL, 5 mmol), THF (17 mL), Et_3N (0.94 mL, 6.5 mmol) and pivaloyl chloride (0.74 mL, 6.0 mmol) for 1 h, gave the title compound **S11** (0.87 g, 81%) as a pink oil with all spectroscopic data in accordance to literature. [51] **IR** ν_{max} (film) 2938, 1794, 1722, 1068, 995; ^{1}**H NMR** (500 MHz, $CDCl_3$): δ 7.08 (sept, 1H, J = 1.6 Hz), 2.31–2.23 (m, 4H), 1.70–1.60 (m, 4H), 1.29 (s 9H).

(*E*)-pivalic (*E*)-3-(thiophen-2-yl)acrylic anhydride **S12**

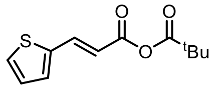

S12

Following general procedure **2**, (*E*)-3-(thiophen-2-yl)acrylic acid (0.77 g, 5 mmol), THF (17 mL), Et_3N (0.94 mL, 6.5 mmol) and pivaloyl chloride (0.74 mL, 6.0 mmol) for 1 h, gave the title compound **S12** (0.87 g, 73%) as a pale brown solid with all spectroscopic data in accordance to literature. [51] **mp** 36–38 °C; **IR** ν_{max} (film) 1790, 1721, 1612, 1042, 1007; ^{1}**H NMR** (500 MHz, $CDCl_3$): δ 7.86 (d, 1H, J = 15.6 Hz), 7.47 (app d, 1H, J = 5.1 Hz), 7.34 (app d, 1H, J = 3.6 Hz), 7.10 (dd, 1H, J = 5.1, 3.7 Hz), 6.23 (d, 1H, J = 15.6), 1.32 (s, 9H).

5.2. Pyrazole Synthesis

N-(3-methyl-1-phenyl-1*H*-pyrazol-5-yl)-*N*-(methylsulfonyl)methanesulfonamide **S33**

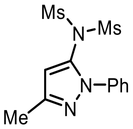

S33

Following general procedure **4**, 3-methyl-1-phenyl-1*H*-pyrazol-5-amine (1.73 g, 10 mmol), Et_3N (4.18 mL, 30 mmol), CH_2Cl_2 (30 mL) and methanesulfonyl chloride (2.32 mL, 30 mmol) for 2 h gave, after purification by Biotage® Selekt™ [Sfär HC 50 g, 65 mL Petrol : EtOAc 100:0 to 50:50 20 CV)], the title compound **S33** (2.78 g, 84%) as a white solid. **mp** 153–155 °C; **IR** ν_{max} (film) 2916, 1676, 1547, 1362, 1163; **^{1}H NMR** (400 MHz, $CDCl_3$): δ 7.63–7.61 (m, 2H), 7.53–7.44 (m, 3H), 6.37 (s, 1H), 3.06 (s, 6H), 2.37 (s, 3H); **^{13}C{^{1}H} NMR** (126 MHz, $CDCl_3$): δ 149.7, 138.1, 131.3, 129.5, 129.4, 126.7, 107.0, 42.8, 14.4; **HRMS** (ESI$^+$) $C_{12}H_{15}N_3O_4S_2Na$ $[M+Na]^+$ found 352.0387, requires 352.0396 (–2.6 ppm).

N-(3-methyl-1-phenyl-1*H*-pyrazol-5-yl)methanesulfonamide **15**

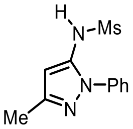

15

Following general procedure **5**, *N*-(3-methyl-1-phenyl-1*H*-pyrazol-5-yl)-*N*-(methylsulfonyl)methanesulfonamide **S33** (2.78 g, 8.5 mmol), THF (21 mL) and aq. 10 % NaOH (21 mL, 8.5 mmol) for 1 h, gave the title compound **15** (1.86 g, 87%) as a white solid. **mp** 163−164 °C; **IR** ν_{max} (film) 3011, 2793, 1321, 1149; **^{1}H NMR** (400 MHz, $CDCl_3$): δ 7.53−7.41 (m, 5H), 6.30 (br s, 1H), 6.22 (s, 1H), 2.93 (s, 3H), 2.32 (s, 3H); **^{13}C{^{1}H} NMR** (126 MHz, $CDCl_3$): δ 149.6, 137.7, 134.4, 129.7, 128.6, 125.2, 101.4, 40.3, 14.1; **HRMS** (ESI$^+$) $C_{11}H_{13}O_2N_3SNa$ $[M+Na]^+$ found 274.0622, requires 274.0621 (+0.7 ppm).

N-(1,3-diphenyl-1*H*-pyrazol-5-yl)-*N*-(methylsulfonyl)methanesulfonamide **S34**

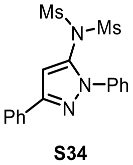

S34

Following general procedure **4**, 1,3-diphenyl-1*H*-pyrazol-5-amine (2.35 g, 10.0 mmol), Et_3N (30 mmol) methanesulfonyl chloride (2.32 mL, 30 mmol, 3.0 equiv), Et_3N (4.18 mL, 30 mmol, 3.0 equiv) and

CH_2Cl_2 (30 mL) gave, after purification via column chromatography (R_f = 0.30, Petrol:EtOAc (3:1)). the title compound **S34** (3.60 g, 92%) as an off-white solid with all the spectroscopic data in accordance to literature.[45] **mp** 158 °C; **IR** ν_{max} (solid) 3044, 1593, 1547, 1499, 1460, 1352, 1315, 1157. **^{1}H NMR** (500 MHz, $CDCl_3$): δ 7.93 – 7.86 (m, 2H), 7.78 – 7.71 (m, 2H), 7.60 – 7.55 (m, 2H), 7.55 – 7.50 (m, 1H), 7.48 – 7.42 (m, 2H), 7.41 – 7.36 (m, 1H), 6.90 (s, 1H), 3.13 (s, 6H); **^{13}C{^{1}H} NMR** (126 MHz, $CDCl_3$): δ 152.0, 138.0, 132.3, 132.1, 129.62, 129.55, 128.9, 128.7, 126.9, 125.8, 104.6, 42.9.

N-(1,3-diphenyl-1*H*-pyrazol-5-yl)methanesulfonamide **S35**

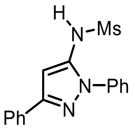

S35

Following general procedure **5**, *N*-(1,3-diphenyl-1*H*-pyrazol-5-yl)-*N*-(methylsulfonyl)methanesulfonamide **S34** (1.96 g, 5.0 mmol, 1.0 equiv.) in 10% aq. NaOH (13 mL) and THF (13 mL) gave the title compound **S35** (1.02 g, 65%) as a pale brown solid. **mp** 191 °C; **IR** ν_{max} (solid) 3061, 2789, 1595, 1553, 1501, 1458, 1425, 1364, 1329, 1148; **^{1}H NMR** (400 MHz, $CDCl_3$) δ: 7.87 – 7.80 (m, 2H), 7.56 – 7.46 (m, 4H), 7.46 – 7.38 (m, 3H), 7.38 – 7.32 (m, 1H), 6.66 (s, 1H), 5.78 (br s, 1H), 2.90 (s, 3H); **^{13}C{^{1}H} NMR** (101 MHz, $CDCl_3$) δ: 151.9, 137.8, 135.3, 132.6, 129.8, 128.9, 128.8, 128.6, 125.8, 125.3, 98.8, 40.3; **HRMS** $(ESI)^+$ $C_{16}H_{15}O_2N_3SNa$ $[M+Na]^+$ found 336.0777, requires 336.0777 (− 0.2 ppm).

N-(1-(4-methoxyphenyl)-3-methyl-1*H*-pyrazol-5-yl)-*N*-(methylsulfonyl)methanesulfonamide **S36**

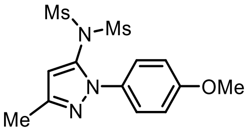

S36

Following general procedure **4**, 1-(4-methoxyphenyl)-3-methyl-1*H*-pyrazol-5-amine (2.03 g, 10.0 mmol, 1.0 equiv), methanesulfonyl chloride (2.32 mL, 30.0 mmol, 3.0 equiv), Et_3N (4.18 mL, 30.0 mmol, 3.0 equiv) and CH_2Cl_2 (30 mL) gave, after purification via column chromatography (R_f = 0.19, Petrol:EtOAc (3:1)), the title compound **S36** (3.24 g, 90%) as an off-white solid **mp** 171 °C. **IR** ν_{max} (solid) 3011, 2930, 1547, 1514, 1366, 1354, 1248, 1161. **^{1}H NMR** (400 MHz, $CDCl_3$): δ 7.56 – 7.47 (m, 2H), 7.02 – 6.97 (m, 2H), 6.336 – 6.335 (app m, 1H), 3.85 (s, 3H), 3.08 (s, 6H), 2.36 (s, 3H). **^{13}C{^{1}H} NMR** (101 MHz, $CDCl_3$): δ 160.2, 149.3, 131.3, 130.9, 128.2, 114.5, 106.6, 55.7, 42.9, 14.4; **HRMS** $(ESI)^+$ $C_{13}H_{17}O_5N_3S_2Na$ $[M+Na]^+$ found 382.0501, requires 382.0502 (−0.1 ppm).

N-(1-(4-methoxyphenyl)-3-methyl-1*H*-pyrazol-5-yl)methanesulfonamide **24**

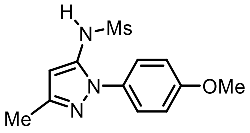

24

Following general procedure **5**, *N*-(1-(4-methoxyphenyl)-3-methyl-1*H*-pyrazol-5-yl)-*N*-(methylsulfonyl)methanesulfonamide **S36** (1.80 g, 5.0 mmol, 1.0 equiv.) in 10% aq. NaOH (13 mL) and THF (13 mL) gave the title compound **24** (1.23 g, 87%). as a pale brown solid **mp** 163 °C. **IR** 3003, 1553, 1518, 1406, 1335, 1258, 1155. **^{1}H NMR** (400 MHz, $CDCl_3$) δ: 7.38 – 7.30 (m, 2H), 7.03 – 6.93 (m, 2H), 6.49 (br s, 1H), 6.15 (s, 1H), 3.85 (s, 3H), 2.91 (s, 3H), 2.30 (s, 3H). **^{13}C{^{1}H} NMR** (101 MHz, $CDCl_3$) δ: 159.9, 149.4, 134.7, 130.6, 127.0, 115.0, 100.4, 55.8, 40.4, 14.2. **HRMS** $C_{12}H_{15}O_3N_3SNa$ $[M+Na]^+$ found 304.0729, requires 304.0726 (+1.0 ppm).

3-Methyl-1-(perfluorophenyl)-1*H*-pyrazol-5-amine **S37**

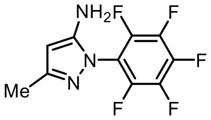

S37

Following general procedure **3**, (perfluorophenyl)hydrazine (1.98 g, 10 mmol), 3-aminocrotonitrile (0.82 g, 10 mmol) and 2 M HCl (20 mL) for 16 h gave, after purification by Biotage® Selekt™ [Sfär HC 100g, 120 mL.min^{-1}, Petrol : EtOAc (100:0 to 50:50 20 CV)], the title compound **S37** (1.79 g, 69%) as an orange oil. **IR** ν_{max} (film) 3312, 3183, 1634, 1510, 993; **^{1}H NMR** (500 MHz, $CDCl_3$): δ 5.55 (s, 1H), 3.55 (br s, 2H), 2.23 (s, 3H); **^{13}C{^{1}H} NMR** (126 MHz, $CDCl_3$): δ 152.5, 147.3, 145.3–145.1 (m), 143.2–143.1 (m), 142.9–142.7 (m), 140.8–140.7 (m), 139.2–139.0 (m), 137.2–137.0 (m), 92.2, 14.2; **^{19}F{^{1}H} NMR** (376 MHz, $CDCl_3$): δ −144.49- −144.58 (m, 2F), -152.35 (t, 1F, J = 21.3 Hz), −160.90- −161.06 (m, 2F); **HRMS** (ESI$^+$) $C_{10}H_7F_5N_3$ $[M+H]^+$ found 264.0555, requires 264.0555 (+0.31 ppm).

N-(3-methyl-1-(perfluorophenyl)-1*H*-pyrazol-5-yl)-*N*-(methylsulfonyl)methanesulfonamide **S38**

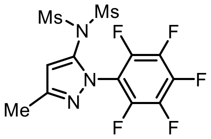

S38

Following general procedure **4**, 3-methyl-1-(perfluorophenyl)-1*H*-pyrazol-5-amine **S37** (0.79 g, 3.0 mmol), Et_3N (1.25 mL, 9 mmol), CH_2Cl_2 (10 mL) and methanesulfonyl chloride (0.7 mL, 9 mmol) for 2 h gave, after purification by Biotage® Selekt™ [Sfär HC 50 g, 65 mL.min^{-1}, Petrol : EtOAc (100:0 to 60:40 20 CV, 60:40 to 50:50 10 CV)], the title compound **S38** (0.70 g, 56%) as a pale yellow solid.

mp 221–222 °C (*dec.*); **IR** ν_{max} (film) 2926, 1533, 1371, 1161; **^{1}H NMR** (400 MHz, $CDCl_3$): δ 6.48 (s, 1H), 3.39 (2, 6H), 2.39 (s, 3H); **^{13}C{^{1}H} NMR** (126 MHz, $CDCl_3$): δ 152.1, 145.3–145.1 (m), 143.6–143.1 (m), 141.5–141.3 (m), 139.0–138.7 (m), 137.0–136.7 (m), 132.6, 113.7, 108.5, 41.7, 14.4; **^{19}F{^{1}H} NMR** (376 MHz, $CDCl_3$): δ –143.5 - –143.6 (app m, 2F), -150.3 (tt, 1F, J = 21.2, 2.2 Hz), -160.91 (td, 2F, J = 21.1, 5.2 Hz); **HRMS** (ESI$^+$) $C_{12}H_{10}F_5N_3O_4S_2Na$ [M+Na]$^+$ found 441.99231, requires 441.9925 (+1.3 ppm).

N-(3-methyl-1-(perfluorophenyl)-1H-pyrazol-5-yl)methanesulfonamide **S39**

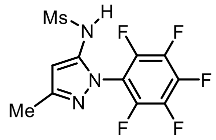

S39

Following general procedure **5**, *N*-(3-methyl-1-(perfluorophenyl)-1*H*-pyrazol-5-yl)-*N*-(methylsulfonyl)methanesulfonamide **S38** (0.54 g, 1.3 mmol), THF (3.3 mL), 10% aq. NaOH (3.3 mL, 1.3 mmol) for 1 h, gave the title compound **S39** (0.21 g, 46%) as a pale yellow solid. **mp** 224–225 °C; **IR** ν_{max} (film) 2992, 1514, 1335, 1152, 993; **^{1}H NMR** (400 MHz, DMSO-d_6): δ 10.22 (br s, 1H), 6.31 (s, 1H), 3.04 (s, 3H), 2.22 (s, 3H); **^{13}C{^{1}H} NMR** (126 MHz, DMSO-d_6): δ 151.3, 144.8–144.7 (m), 142.8–142.7 (m), 140.7–140.4 (m), 138.9, 138.4–138.2 (m), 136.4–136.2 (m), 113.6, 100.5, 40.0, 13.9; **^{19}F{^{1}H} NMR** (377 MHz, $CDCl_3$): δ –144.93 - –145.02 (m, 2F), –150.87 (tt, 1F, J = 21.5, 2.2 Hz), –160.45 - –160.59 (m, 2F); **HRMS** (ESI$^+$) $C_{11}H_8F_5N_3O_2SNa$ [M+Na]$^+$ found 364.0146, requires 364.0150 (–1.0 ppm).

N-(1-(4-fluorophenyl)-3-methyl-1*H*-pyrazol-5-yl)-*N*-(methylsulfonyl)methanesulfonamide **S40**

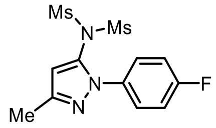

S40

Following general procedure **4**, 1-(4-fluorophenyl)-3-methyl-1*H*-pyrazol-5-amine (1.91 g, 10 mmol, 1.0 equiv.), methanesulfonyl chloride (2.32 mL, 30 mmol, 3.0 equiv.), Et_3N (4.18 mL, 30 mmol) and CH_2Cl_2 (30 mL) gave, after purification *via* column chromatography (R_f = 0.26, Petrol:EtOAc (3:1)), the title compound **S40** (3.04 g, 87%) as an off-white solid. **mp** 166 °C. **IR** ν_{max} (solid) 2929, 1553, 1510, 1418, 1354, 1325, 1227. **^{1}H NMR** (400 MHz, $CDCl_3$): δ 7.67 – 7.60 (m, 2H), 7.27 – 7.18 (m, 2H), 6.391–6.390 (app m, 1H), 3.14 (s, 6H), 2.39 (s, 3H); **^{13}C{^{1}H} NMR** (101 MHz, $CDCl_3$): δ 162.8 (d, J = 250.3 Hz), 149.8, 134.1, 131.3, 128.8 (d, J = 8.8 Hz), 116.4 (d, J = 22.8 Hz), 107.1, 42.8, 14.3; **^{19}F{^{1}H} NMR** (377 MHz, $CDCl_3$): δ -110.9 (S); **HRMS** (ESI)$^+$ $C_{12}H_{14}O_4N_3FS_2Na$ [M+Na]$^+$ found 370.0304, requires 370.0302 (+0.5 ppm).

N-(1-(4-fluorophenyl)-3-methyl-1*H*-pyrazol-5-yl)methanesulfonamide **S41**

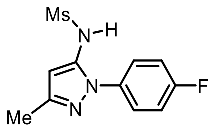

S41

Following general procedure **5**, *N*-(1-(4-fluorophenyl)-3-methyl-1*H*-pyrazol-5-yl)-*N*-(methylsulfonyl)methanesulfonamide **S40** (1.74 g, 5 mmol, 1.0 equiv.) in 10% aq. NaOH (13 ml and THF (13 mL) gave the title compound **S41** as a pale-yellow solid (0.99 g, 3.69 mmol, 74%). **mp** 165 °C. **IR** ν_{max} (solid) 3021, 2797, 1558, 1508, 1325, 1231, 1150. **^{1}H NMR** (400 MHz, $CDCl_3$): δ 7.49 – 7.42 (m, 2H), 7.20 – 7.12 (m, 2H), 6.47 (br s, 1H), 6.18 (s, 1H), 2.95 (s, 3H), 2.30 (s, 3H); **^{13}C{^{1}H} NMR** (101 MHz, $CDCl_3$): δ 162.3 (d, J = 249.1 Hz), 149.7, 134.3, 133.9, 127.2 (d, J = 8.7 Hz), 116.6 (d, J = 22.9 Hz), 101.8, 40.4, 14.1; **^{19}F{^{1}H} NMR** (377 MHz, $CDCl_3$): δ -112.2; **HRMS** $C_{11}H_{12}O_2N_3FSNa$ $[M+Na]^+$ found 292.0528, requires 292.0527 (+0.5 ppm).

4-(5-amino-3-methyl-1*H*-pyrazol-1-yl)benzonitrile **S42**

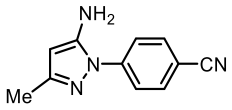

S42

Following general procedure **3**, 4-cyanophenylhydrazine hydrochloride (3.39 g, 20 mmol), 3-aminocrotonitrile (1.56 g, 20 mmol) and 2 M HCl (40 mL) for 16 h gave, after purification by Biotage® SelektTM [Sfär HC 100g, 120 mL.min^{-1}, Petrol : EtOAc (90:10 to 20:80 15 CV, 20:80 to 10:90 10 CV)], the title compound **S42** (2.30 g, 58%) as an orange solid with all spectroscopic data in accordance to literature.[53] **mp** 157–159 °C{Lit.[53] 155–157 °C); **IR** ν_{max} (film) 3399, 3184, 2922, 2226, 1647, 1520; **^{1}H NMR** (400 MHz, $CDCl_3$): δ 7.80 (d, 2H, J = 8.7 Hz), 7.73 (d, 2H, J = 8.8 Hz), 5.52 (s, 1H), 3.80 (br s, 2H), 2.23 (s, 3H).

N-(1-(4-cyanophenyl)-3-methyl-1*H*-pyrazol-5-yl)-*N*-(methylsulfonyl)methanesulfonamide **S43**

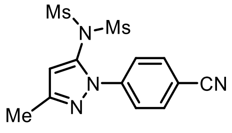

S43

Following general procedure **4** 4-(5-amino-3-methyl-1*H*-pyrazol-1-yl)benzonitrile **S42** (2.30 g, 11.6 mmol), Et_3N (4.85 mL, 34.8 mmol), CH_2Cl_2 (34 mL) and methanesulfonyl chloride (2.75 mL, 35 mmol) for 2 h gave, after recrystallisation from EtOH, the title compound **S43** (2.98 g, 72%) as a pale yellow solid. **mp** 211 °C (*dec*, EtOH); **IR** ν_{max} (film) 3012, 2234, 1607, 1523, 1369, 1161; **^{1}H NMR** (500 MHz, $CDCl_3$): δ 7.81 (s, 4H), 6.44 (s, 1H), 3.14 (s, 6H), 2.38 (s, 3H); **^{13}C{^{1}H} NMR** (126 MHz, $CDCl_3$): δ

151.0, 141.5, 133.4, 131.1, 126.3, 117.9, 112.7, 108.5, 42.8, 14.4 ppm; **HRMS** (ESI$^+$) $C_{13}H_{14}N_4O_4S_2Na$ [M+Na]$^+$ found 377.0345, requires 377.0349 (–1.0 ppm).

N-(1-(4-cyanophenyl)-3-methyl-1*H*-pyrazol-5-yl)methanesulfonamide **S7**

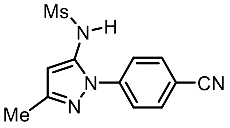

S7

Following general procedure **5**, *N*-(1-(4-cyanophenyl)-3-methyl-1*H*-pyrazol-5-yl)-*N* (methylsulfonyl) methanesulfonamide **S43** (2.98 g, 8.41 mmol), THF (21 mL) and 10% aq. NaOH (21 mL, 8.41 mmol) for 1 h, gave the title compound **S7** (1.57 g, 68%) as a pale yellow solid. **mp** 171−172 °C; **IR** ν_{max} (film) 3264, 3021, 2228, 1607, 1512, 1329, 1152; **^{1}H NMR** (500 MHz, $CDCl_3$): δ 7.76 (app s, 4H), 6.54 (br s, 1H), 6.26 (s, 1H), 3.04 (s, 3H), 2.33 (s, 3H); **^{13}C{^{1}H} NMR** (126 MHz, $CDCl_3$): δ 150.8, 141.8, 134.1, 133.5, 124.5, 118.3, 111.3, 104.6, 40.4, 14.2 ppm; **HRMS** (ESI$^+$) $C_{12}H_{12}O_2N_4SNa$ [M+Na]$^+$ found 299.0567, requires 299.0573 (–2.1 ppm).

3-Methyl-1-(*o*-tolyl)-1*H*-pyrazol-5-amine **S44**

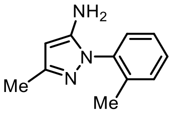

S44

Following general procedure **3**, *o*-tolylhydrazine hydrochloride (1.59 g, 10 mmol), 3-aminocrotonitrile (0.82 g, 10 mmol) and 2 M aq. HCl (20 mL) for 16 h gave, after purification by Biotage® SelektTM [Sfär HC 100g, 120 mL.min^{-1}, Petrol : EtOAc (85:15 to 20:80 20 CV)], the title compound **S44** (1.30 g, 70%) as an orange solid with all spectroscopic data in accordance to literature. [54] **mp** 87–89 °C {Lit. [54] 89–92 °C}; **IR** ν_{max} (film) 3302, 3171, 1622, 1558, 1460; **^{1}H NMR** (500 MHz, $CDCl_3$): δ 7.34–7.25 (m, 4H), 5.41 (s, 1H), 3.49 (br s, 2H), 2.22 (s, 3H), 2.16 (s, 3H).

N-(3-methyl-1-(*o*-tolyl)-1*H*-pyrazol-5-yl)-*N*-(methylsulfonyl)methanesulfonamide **S45**

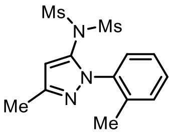

S45

Following general procedure **4**, 3-methyl-1-(*o*-tolyl)-1*H*-pyrazol-5-amine **S44** (1.12 g, 6 mmol), Et_3N (2.5 mL, 18 mmol), CH_2Cl_2 (20 mL) and methanesulfonyl chloride (1.4 mL, 18 mmol) for 2 h gave, after purification by Biotage® Selekt^{TM} [Sfär HC 50 g, 65 mL.min^{-1}, Petrol : EtOAc (100:0 to 40:60 20 CV, 40:60 to 50:50 10 CV)], the title compound **S45** (1.29 g, 63%) as a pale yellow solid. **mp** 165–166

°C (*dec*); **IR** ν_{max} (film) 3017, 1547, 1429, 1367, 1358, 1163; **^{1}H NMR** (500 MHz, $CDCl_3$): δ 7.57 (dd, 1H, J = 7.6, 1.5 Hz), 7.39–7.30 (m, 3H), 6.35 (s, 1H), 3.03 (br s, 6H), 2.37 (s, 3H), 2.16 (s, 3H); **^{13}C{^{1}H} NMR** (126 MHz, $CDCl_3$): δ 149.1, 137.8, 136.6, 131.6, 131.5, 130.1, 128.3, 126.5, 106.0, 42.7, 17.7, 14.4; **HRMS** (ESI$^+$) $C_{13}H_{17}N_3O_4S_2Na$ $[M+Na]^+$ found 366.0552, requires 366.0558 (–0.1 ppm).

N-(3-methyl-1-(*o*-tolyl)-1*H*-pyrazol-5-yl)methanesulfonamide **S46**

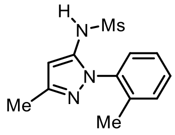

S46

Following general procedure **5**, *N*-(3-methyl-1-(*o*-tolyl)-1*H*-pyrazol-5-yl)-*N*-(methylsulfonyl)methanesulfonamide **S45** (1.02 g, 3.0 mmol), THF (7.5 mL), 10% aq. NaOH (7.5 mL, 3.0 mmol) for 1 h, gave the title compound **S46** (0.60 g, 75%) as a pale orange solid. **mp** 180 °C; **IR** ν_{max} (film) 2997, 1559, 1462, 1333, 1159; **^{1}H NMR** (500 MHz, $CDCl_3$): δ 7.40 (td, 1H, J = 7.4, 1.4 Hz), 7.35 (app d, 1H, J = 7.5 Hz), 7.31 (td, 1H, J = 7.5, 1.7 Hz), 7.23 (dd, 1H, J = 7.7, 1.4 Hz), 6.20 (br s, 1H), 6.15 (s, 1H), 2.91 (s, 3H), 2.31 (s, 3H), 2.11 (s, 3H); **^{13}C{^{1}H} NMR** (126 MHz, $CDCl_3$): δ 149.5, 136.8, 136.0, 135.7, 131.7, 130.3, 128.1, 127.2, 97.9, 40.2, 17.4, 14.2; **HRMS** (ESI$^+$) $C_{12}H_{15}N_3O_2SNa$ $[M+Na]^+$ found 288.0776, requires 288.0777 (–0.5 ppm).

N-(1-benzyl-3-methyl-1*H*-pyrazol-5-yl)-*N*-(methylsulfonyl)methanesulfonamide **S47**

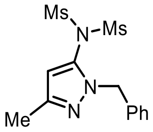

S47

Following general procedure **4**, 1-benzyl-3-methyl-1*H*-pyrazol-5-amine (1.87 g, 10.0 mmol), methanesulfonyl chloride (2.32 mL, 30.0 mmol), NEt_3 (4.18 mL, 30.0 mmol) in CH_2Cl_2 (30 mL) gave, after purification via column chromatography (Petrol:EtOAc), the title compound **S47** (2.22 g, 65%) as an off-white solid **mp** 171 °C. **IR** ν_{max} (solid) 3013, 2930, 1541, 1456, 1356, 1315, 1159. **^{1}H NMR** (400 MHz, $CDCl_3$): δ 7.36 – 7.31 (m, 2H), 7.30 – 7.26 (m, 1H), 7.18 – 7.10 (m, 2H), 6.27 – 6.27 (m, 1H), 5.42 (s, 2H), 3.01 (s, 6H), 2.34 (s, 3H); **^{13}C{^{1}H} NMR** (101 MHz, $CDCl_3$)**:**δ 148.2, 136.8, 130.0, 129.0, 128.1, 127.0, 107.1, 52.8, 41.8, 14.3; **HRMS** (ESI$^+$) $C_{13}H_{17}O_4N_3S_2Na$ $[M+Na]^+$ found 366.0554, requires 366.0553 (+0.4 ppm).

N-(1-benzyl-3-methyl-1*H*-pyrazol-5-yl)methanesulfonamide **S15**

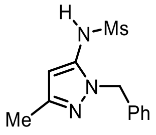

S15

Following general procedure **5**, *N*-(1-benzyl-3-methyl-1*H*-pyrazol-5-yl)-*N*-(methylsulfonyl)methanesulfonamide **S47** (1.72 g, 5.0 mmol, 1.0 equiv) in 10% aq. NaOH (13 mL and THF (13 mL) gave the title compound **S15** (0.92 g, 70%) as an off-white solid **mp** 141 °C. **IR** ν_{max} (solid) 2965, 2718, 1553, 1447, 1333, 1153. **^{1}H NMR** (500 MHz, $CDCl_3$):δ 7.34 – 7.29 (m, 2H), 7.28 – 7.24 (m, 1H), 7.18 – 7.14 (m, 2H), 6.06 (s, 1H), 5.31 (s, 2H), 2.90 (s, 3H), 2.26 (s, 3H); **^{13}C{^{1}H} NMR** (126 MHz, $CDCl_3$):δ 148.1, 136.7, 133.6, 129.0, 128.1, 127.3, 102.6, 52.3, 39.8, 14.2;. **HRMS** (ESI$^+$) $C_{12}H_{15}N_3O_2SNa$ [M+Na]$^+$ found 288.0778, requires 288.0777 (+0.2 ppm).

1-Mesityl-3-methyl-1*H*-pyrazol-5-amine **S48**

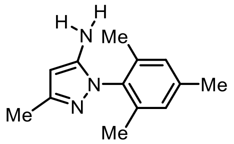

S48

Following general procedure **3**, mesitylene hydrazine hydrochloride (1.86 g, 10 mmol), 3-aminocrotonitrile (0.82 g, 10 mmol) and 2 M HCl (20 mL) for 16 h gave, after purification by Biotage® Selekt™ [Sfär HC 100g, 120 mL.min^{-1}, Petrol : EtOAc (65:35 to 0:100 20 CV)], the title compound **S48** (1.17 g, 54%) as an orange solid. **mp** 131–132 °C; **IR** ν_{max} (film) 3148, 2918, 1630, 1558, 1381; **^{1}H NMR** (400 MHz, $CDCl_3$): δ 6.93 (s, 2H), 5.41 (s, 1H), 3.39 (br s, 2H), 2.31 (s, 3H), 2.22 (s, 3H), 2.02 (s, 6H); **^{13}C{^{1}H} NMR** (126 MHz, $CDCl_3$): δ 149.0, 145.8, 139.1, 137.5, 133.1, 129.1, 88.4, 21.3, 17.5, 14.3; **HRMS** (ESI$^+$) $C_{13}H_{18}N_3$ [M+H]$^+$ found 216.1496, requires 216.1495 (+0.5 ppm).

N-(1-mesityl-3-methyl-1*H*-pyrazol-5-yl)-*N*-(methylsulfonyl)methanesulfonamide **S49**

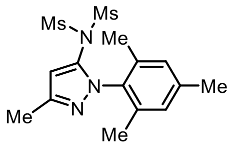

S49

Following general procedure **4**, 1-mesityl-3-methyl-1*H*-pyrazol-5-amine **S48** (1.11 g, 5.2 mmol), Et_3N (2.2 mL, 15.5 mmol), CH_2Cl_2 (20 mL) and methanesulfonyl chloride (1.2 mL, 15.5 mmol) for 2 h gave, after purification Biotage® Selekt™ [Sfär HC 100 g, 120 mL.min^{-1}, Petrol : EtOAc (100:0 to 60:40 20 CV, 60:40 to 40:60 10 CV)], the title compound **S49** (1.49 g, 78%) as a white solid. **mp** 202–205 °C; **IR** ν_{max} (solid) 3009, 1544, 1368, 1352, 1165; **^{1}H NMR** (400 MHz, $CDCl_3$): δ 6.93 (s, 2H), 6.32 (s, 1H), 3.05 (s, 6H), 2.36 (s, 3H), 2.30 (s, 3H), 2.06 (s, 6H); **^{13}C{^{1}H} NMR** (126 MHz, $CDCl_3$): δ 149.0,

139.9, 138.3, 133.4, 133.1, 129.2, 106.0, 43.1, 21.9, 18.1, 14.4; **HRMS** (ESI^+) $C_{15}H_{21}N_3O_4S_2Na$ $[M+Na]^+$ found 394.0871, requires 394.0866 (+1.5 ppm).

N-(1-mesityl-3-methyl-1*H*-pyrazol-5-yl)methanesulfonamide **S16**

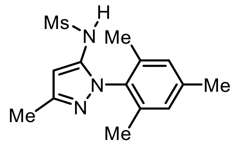

S16

Following general procedure **5**, *N*-(1-mesityl-3-methyl-1*H*-pyrazol-5-yl)-*N*-(methylsulfonyl)methanesulfonamide **S49** (1.4 g, 3.8 mmol), THF (10 mL) and 10% aq. NaOH (10 mL, 3.8 mmol) for 1 h, gave the title compound **S16** (0.91 g, 82%) as a white solid. **mp** 212–214 °C; **IR** ν_{max} (solid) 3017, 2926, 1564, 1327, 1152; **^{1}H NMR** (400 MHz, $CDCl_3$): δ 6.96 (s, 2H), 6.14 (br s, 1H), 6.10 (s, 1H), 2.96 (s, 3H), 2.33 (s, 3H), 2.30 (s, 3H), 1.97 (s, 6H); **^{13}C{^{1}H} NMR** (126 MHz, $CDCl_3$): δ 149.6, 140.3, 137.1, 136.1, 132.0, 129.5, 95.4, 40.2, 21.3, 17.4, 14.3; **HRMS** (ESI^+) $C_{14}H_{19}O_2N_3SNa$ $[M+Na]^+$ found 316.1094, requires 316.1090 (+1.2 ppm).

5.3. Pyrazolone Synthesis

2,5-Diphenyl-2,4-dihydro-3*H*-pyrazol-3-one **43**

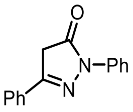

43

Following general procedure **6**, ethyl benzoylacetate (3.5 mL, 20 mmol), phenyl hydrazine (2.0 mL, 20 mmol) and glacial acetic acid (4.0 mL) for 3 h, gave the title compound **43** (2.7 g, 57%) as an orange solid with all the spectroscopic data in accordance with the literature. [55] **mp** 135–138 °C{Lit. [56] 139–141 °C}; **IR** ν_{max} (solid) 1697, 1591, 1119; **^{1}H NMR** (400 MHz, $CDCl_3$): δ 8.00–7.97 (m, 2H), 7.80–7.78 (m, 2H), 7.48–7.42 (m, 5H), 7.25–7.21 (m, 1H), 3.87 (s, 2H); **^{13}C{^{1}H} NMR** (101 MHz, $CDCl_3$): δ 170.4, 154.8, 138.3, 131.0, 130.9, 129.1, 129.0, 126.1, 125.5, 119.2, 39.8.

5-(4-Bromophenyl)-2-phenyl-2,4-dihydro-3*H*-pyrazol-3-one **S50**

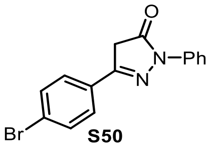

S50

Following general procedure **6**, methyl 3-(4-bromophenyl)-3-oxopropanoate (2.6 mL, 10 mmol), phenyl hydrazine (1.0 mL, 10 mmol) and glacial acetic acid (2.0 mL) for 1 h, gave the title compound **S50** (2.5 g, 78%) as an off-white solid with all the spectroscopic data in accordance with the literature. [57] **mp** 171–174 °C {Lit. [57] 171–172 °C}; **IR** ν_{max} (solid) 1713, 1595, 1492, 1071; **^{1}H NMR** (300 MHz, $CDCl_3$): δ 7.97–7.93 (m, 2H), 7.66–7.56 (m, 4H), 7.46–7.40 (m, 2H), 7.23 (app t, 1H, J = 7.4 Hz), 3.82 (s, 2H).

5-(4-Methoxyphenyl)-2-phenyl-2,4-dihydro-3*H*-pyrazol-3-one **S51**

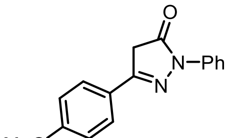

S51

Following general procedure **6**, methyl 3-(4-methoxyphenyl)-3-oxopropanoate (1.3 mL, 6 mmol), phenyl hydrazine (0.6 mL, 6 mmol) and glacial acetic acid (1.2 mL) for 1 h, gave the title compound **S51** (1.3 g, 79%) as an orange solid with all the spectroscopic data in accordance with the literature. [58] **mp** 137–139 °C {Lit. [59] 141–142 °C (EtOH)}; **IR** ν_{max} (solid) 1713, 1499, 1253, 1022; **^{1}H NMR** (300 MHz, $CDCl_3$): δ 8.00–7.96 (m, 2H), 7.72 (app dt, 2H, J = 9.0, 2.1 Hz), 7.45–7.30 (m, 2H), 7.24–7.19 (m, 1H), 6.97 (app dt, 2H, J = 8.7, 2.1 Hz), 3.87 (s, 3H), 3.83 (s, 2H).

Methyl 3-oxo-3-(4-(trifluoromethyl)phenyl)propanoate **S52**

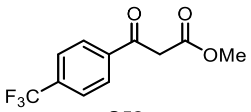

S52

Under nitrogen, a solution of 1-(4-(trifluoromethyl)phenyl)ethan-1-one (7.5 g, 40.0 mmol, 1.0 equiv.) in toluene (40 mL) was added dropwise to a refluxing mixture of sodium hydride (4.5 g, 0.11 mol, 2.8 equiv.) and dimethyl carbonate (6.7 mL, 80.0 mmol, 2.0 equiv.) in toluene (20 mL). The mixture was heated to reflux for 30 min before being cooled to rt. Acetic acid (12 mL) and ice-cold water (20 mL) were added and the organic layer was extracted with diethyl ether and brine. The organic layer was dried (Na_2SO_4) and concentrated under reduced pressure. The crude product was purified by column chromatography (7:1 Petrol : EtOAc, R_f 0.65) to give the title compound **S52** (7.4 g, 75%) as a yellow solid with all data in accordance with the literature. [60] **mp** 72–74 °C; **IR** ν_{max} (solid) 2965, 1653, 1111;**^{1}H NMR** (300 MHz, $CDCl_3$): δ [keto, major] 8.06 (app d, 2H, J = 8.1 Hz), 7.76 (app d, 2H, J = 8.1 Hz), 4.04 (s, 2H), 3.77 (s, 3H); [enol, minor] 12.49 (s, 1H), 7.89 (app d, 2H, J = 8.1 Hz), 7.68 (app d, 2H, J = 8.3 Hz), 5.73 (s, 1H), 3.83 (s, 3H); **^{19}F{^{1}H} NMR** (376 MHz, $CDCl_3$): δ [keto, major] –63.22 (s, 3F); [enol, minor] –62.95 (s, 3F).

2-Phenyl-5-(4-(trifluoromethyl)phenyl)-2,4-dihydro-3*H*-pyrazol-3-one **S53**

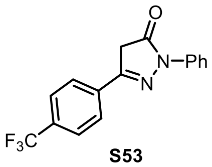

S53

Following general procedure **6**, methyl 3-oxo-3-(4-(trifluoromethyl)phenyl)propanoate **S52** (2.5 g, 10 mmol), phenyl hydrazine (1.0 mL, 10 mmol) and glacial acetic acid (5.5 mL) for 1 h, gave the title compound **S53** (2.1 g, 67%) as a white solid with the spectroscopic data in accordance with the literature. [61] **mp** 173–174 °C {Lit. [61] 166 °C}; **IR** ν_{max} (solid) 1605, 1312, 1111; **^{1}H NMR** (300 MHz, $CDCl_3$) : δ 7.98–7.94 (m, 2H), 7.89 (app d, 2H, J = 8.1 Hz), 7.72 (app d, 2H, J = 8.4 Hz), 7.48–7.42 (m, 2H), 7.28–7.22 (m, 1H), 3.88 (s, 2H); **^{19}F{^{1}H} NMR** (471 MHz, $CDCl_3$) : δ –62.89 (s, CF_3).

5-Methyl-2-phenyl-2,4-dihydro-3*H*-pyrazol-3-one **S8**

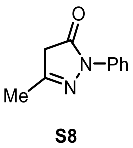

S8

Ethyl acetoacetate (1.3 mL, 10 mmol, 1.0 equiv.) and phenyl hydrazine (0.98 mL, 10 mmol, 1.0 equiv.) were heated under reflux (140 °C) for 2 h. Diethyl ether (5 mL) and hexane (2 mL) were added, and the

mixture was sonicated for *ca.* 15 minutes. The resulting precipitate was filtered to give the title compound **S8** (1.6 g, 91%) as a light orange solid with all the spectroscopic data in accordance with the literature. [46] **mp** 107–111 °C {Lit. [62] 123–124 °C}; **IR** ν_{max} (solid) 1769, 1591, 1034; **^{1}H NMR** (300 MHz, $CDCl_3$): δ 7.88–7.84 (m, 2H), 7.42–7.36 (m, 2H), 7.21–7.15 (m, 1H), 3.43 (s, 2H), 2.20 (s, 3H).

2-(*tert*-butyl)-5-phenyl-2,4-dihydro-3*H*-pyrazol-3-one **S54**

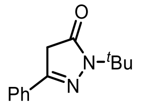

S54

Following a literature procedure, [63] *tert*-butyl hydrazine hydrochloride (2.5 g, 20 mmol, 2.0 equiv.), sodium acetate (1.6 g, 20 mmol, 2.0 equiv.), ethyl acetoacetate (1.9 g, 10 mmol, 1.0 equiv.) and ethanol (11 mL) were heated under reflux (110 °C) for 24 h. The solution was diluted with CH_2Cl_2 (15 mL) and washed with water (3 × 15 mL) and brine (15 mL). The organic layer was dried (Na_2SO_4), filtered, and concentrated under reduced pressure. The crude product was then purified by column chromatography (3:1 Petrol : EtOAc, R_f 0.57) to give the title compound **S54** (1.0 g, 44%) as an off-white solid with all spectroscopic data in accordance with the literature. [64] **mp** 154–155 °C; **IR** ν_{max} (solid) 2978, 1674, 1556, 1231; **^{1}H NMR** (300 MHz, $CDCl_3$): δ 7.66–7.63 (m, 2H), 7.40–7.38 (m, 3H), 3.59 (s, 2H), 1.57 (s, 9H).

5.4. Pyrazole Conjugate Addition Products

(*S*)-3-methyl-7-(methylsulfonyl)-1-phenyl-4-(trifluoromethyl)-1,4,5,7-tetrahydro-6*H*-pyrazolo[3,4-*b*]pyridin-6-one **19**

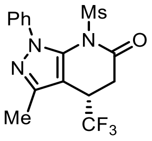

19

Following general procedure **7**, (*E*)-4,4,4-trifluorobut-2-enoic pivalic anhydride **14** (67.3 mg, 0.30 mmol), (2*S*,3*R*)-HyperSe **12** (7.1 mg, 0.02 mmol), CPME (2 mL) and N-(3-methyl-1-phenyl-1H-pyrazol-5-yl)methanesulfonamide **15** (50.3 mg, 0.20 mmol) for 16 h gave, after purification by Biotage® Selekt™ [Sfär HC 25 g, 80 mL.min^{-1}, Petrol : EtOAc (100:0 to 60:40 20 CV, 60:40 to 50:50 10 CV)] the title compound **19** (71.7 mg, 96%) as a white solid. **mp** 117–119 °C; $[\alpha]_D^{20}$ −139 (*c* 1.5, $CHCl_3$); **Chiral HPLC analysis**, Chiralpak AD-H (90:10 hexane : IPA, flow rate 1mL.min^{-1}, 211 nm, 30 °C) t_R (4*S*): 18.1 min, t_R (4*R*): 22.5 min, 99:1 er; **IR** ν_{max} (film) 2926, 1732, 1504, 1364, 1155, 1126; **^{1}H NMR** (400 MHz, $CDCl_3$): δ 7.50–7.44 (m, 4H), 7.41–7.37 (m, 1H), 3.62–3.53 (m, 1H), 3.21–3.17 (m, 4H), 3.05 (dd, 1H, J = 17.3, 7.4 Hz), 2.33 (s, 3H); **^{13}C{^{1}H} NMR** (126 MHz, $CDCl_3$): δ 170.4, 146.9, 140.5, 136.3, 129.4, 128.4, 125.6 (q, $^1J_{CF}$ = 279.8 Hz), 124.0, 102.4, 42.8, 35.8, 34.4 (q, $^2J_{CF}$ = 31.3 Hz), 12.0; **^{19}F{^{1}H} NMR** (377 MHz, $CDCl_3$): δ −71.84 (s, 3F); **HRMS** (ESI$^+$) $C_{15}H_{15}F_3N_3O_3S$ $[M+H]^+$ found 374.0768, requires 374.0781 (−3.4 ppm).

Ethyl (*S*)-3-methyl-7-(methylsulfonyl)-6-oxo-1-phenyl-4,5,6,7-tetrahydro-1*H*-pyrazolo[3,4-*b*]pyridine-4-carboxylate **28**

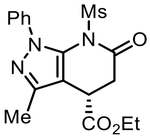

28

Following general procedure **7**, (*E*)-4-ethoxy-4-oxobut-2-enoic pivalic anhydride **S18** (68.4 mg, 0.30 mmol), (2*S*,3*R*)-HyperSe **12** (7.1 mg, 0.02 mmol), CPME (2 mL) and N-(3-methyl-1-phenyl-1H-pyrazol-5-yl)methanesulfonamide **15** (50.3 mg, 0.20 mmol) for 16 h gave, after purification by Biotage® Selekt™ [Sfär HC 25 g, 80 mL.min^{-1}, Petrol : EtOAc (95:5 to 60:40 18 CV, 60:40 to 40:60 10 CV)], the title compound **28** (62.4 mg, 83%) as a colourless oil. $[\alpha]_D^{20}$ −100 (*c* 1.2, $CHCl_3$); **Chiral HPLC analysis**, Chiralpak AD-H (90:10 hexane : IPA, flow rate 1mL.min^{-1}, 211 nm, 30 °C) t_R(4*S*): 24.7 min, t_R(4*R*): 30.1 min, 95:5 er; **IR** ν_{max} (film) 2984, 1728, 1505, 1364, 1151; **^{1}H NMR** (500 MHz, $CDCl_3$): δ 7.49–7.44 (m, 4H), 7.37–7.34 (m,1H), 4.25–4.15 (app m, 2H), 3.78 (dd, 1H, J = 6.1, 1.9 Hz), 3.30 (s, 3H), 3.12 (dd, 1H, J = 16.3, 2.0 Hz), 2.89 (dd, 1H, J = 16.3, 6.1 Hz), 2.35 (s, 3H), 1.29 (t, 3H,

J = 7.1 Hz); **^{13}C{^{1}H} NMR** (126 MHz, $CDCl_3$): δ 171.9, 171.5, 145.7, 140.6, 135.4, 129.4, 128.0, 123.5, 106.7, 62.2, 42.3, 38.6, 34.9, 14.2, 12.1; **HRMS** (ESI$^+$) $C_{17}H_{19}N_3O_5SNa$ [M+Na]$^+$ found 400.0925, requires 400.0938 (−3.2 ppm).

From (Z)-**S18**: $[\alpha]_D^{20}$ −130 (*c* 0.7, $CHCl_3$); **Chiral HPLC analysis**, Chiralpak AD-H (90:10 hexane : IPA, flow rate 1mL.min^{-1}, 211 nm, 30 °C) t_R(4*S*): 25.2 min, t_R(4*R*): 30.6 min, 90:10 er.

Tert-butyl (*S*)-3-methyl-7-(methylsulfonyl)-6-oxo-1-phenyl-4,5,6,7-tetrahydro-1*H*-pyrazolo[3,4-*b*]pyridine-4-carboxylate **29**

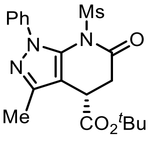

29

Following general procedure **7**, (*E*)-4-(*tert*-butoxy)-4-oxobut-2-enoic pivalic anhydride **S21** (76.8 mg, 0.30 mmol), (2*S*,3*R*)-HyperSe **12** (7.1 mg, 0.02 mmol), CPME (2 mL) and *N*-(3-methyl-1-phenyl-1*H*-pyrazol-5-yl)methanesulfonamide **15** (50.3 mg, 0.20 mmol) for 16 h gave, after purification by Biotage® Selekt™ [Sfär HC 25 g, 80 mL.min^{-1}, Petrol : EtOAc (100:0 to 60:40 20 CV, 60:40 to 50:50 10 CV)], the title compound B29 (76.4 mg, 94 %) as a colourless oil. $[\alpha]_D^{20}$ −97 (*c* 1.4, $CHCl_3$); **Chiral HPLC analysis**, Chiralpak AD-H (90:10 hexane : IPA, flow rate 1mL.min^{-1}, 211 nm, 30 °C) t_R (4*S*): 14.9 min, t_R (4*R*): 18.8 min, 93:7 er; **IR** ν_{max} (film); 2980, 1728, 1506, 1368, 1143; **^{1}H NMR** (400 MHz, $CDCl_3$): δ 7.50–7.42 (m, 4H), 7.35–7.31 (m, 1H), 3.70 (dd, 1H, J = 6.1, 1.9 Hz), 3.27 (s, 3H), 3.09 (dd, 1H, J = 16.2, 2.0 Hz), 2.81 (dd, 1H, J = 16.2, 6.1 Hz), 2.34 (s, 3H), 1.45 (s, 9H); **^{13}C{^{1}H} NMR** (126 MHz, $CDCl_3$): δ 171.7, 171.2, 145.7, 140.6, 135.2, 129.3, 127.9, 123.4, 107.2, 83.0, 42.1, 38.6, 35.9, 28.1, 12.0; **HRMS** (ESI$^+$) $C_{19}H_{23}O_5N_3SNa$ [M+Na]$^+$ found 428.1256, requires 428.1251 (+1.2 ppm).

(*S*)-3-methyl-7-(methylsulfonyl)-1-phenyl-4-(pyrrolidine-1-carbonyl)-1,4,5,7-tetrahydro-6*H*-pyrazolo[3,4-*b*]pyridin-6-one **30**

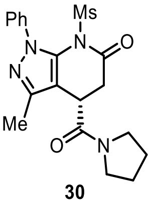

30

Following general procedure **7**, (*E*)-4-oxo-4-(pyrrolidin-1-yl)but-2-enoic pivalic anhydride **S24** (75.9 mg, 0.30 mmol), (2*S*,3*R*)-HyperSe **12** (7.1 mg, 0.02 mmol), CPME (2 mL) and N-(3-methyl-1-phenyl-1H-pyrazol-5-yl)methanesulfonamide **15** (50.3 mg, 0.20 mmol) for 16 h gave, after purification by Biotage® Selekt™ [Sfär HC 25 g, 80 mL.min^{-1}, Petrol : EtOAc (60:40 to 8:92 20 CV, 8:92 to 0:100 10 CV)], the title compound **30** (39.5 mg, 49%) as a white solid. **mp** 234 °C (*dec*); $[\alpha]_D^{20}$ −124 (*c* 0.3,

$CHCl_3$); **Chiral HPLC analysis**, Chiralpak IC (75:25 hexane : IPA, flow rate 1mL.min^{-1}, 211 nm, 30 °C) t_R (4*S*): 12.4 min, t_R (4*R*): 19.6 min, 85:15 er; **IR** ν_{max} (film) 2974, 1724, 1632, 1360, 1161; **^{1}H NMR** (400 MHz, $CDCl_3$): δ 7.50–7.43 (m, 4H), 7.34 (t, 1H, J = 7.0 Hz), 3.90 (dd, 1H, J = 5.2, 3.2 Hz), 3.68 (t, 2H, J = 6.8 Hz), 3.49–3.46 (m, 5H), 3.01 (dd, 1H, J = 15.8, 3.1 Hz), 2.83 (dd, 1H, J =15.8, 5.4 Hz), 2.32 (s, 3H), 2.09–2.02 (m, 2H), 1.97–1.86 (m, 2H); **^{13}C{^{1}H} NMR** (126 MHz, $CDCl_3$): δ 171.9, 169.2, 144.3, 140.7, 136.6, 129.3, 127.9, 123.7, 107.6, 47.2, 46.5, 41.8, 40.2, 34.4, 26.4, 24.3, 13.1; **HRMS** (ESI$^+$) $C_{19}H_{22}N_4O_4SNa$ [M+Na]$^+$ found 425.1247, requires 425.1254 (−1.6 ppm).

(*S*)-3-methyl-4-(4-methylbenzoyl)-7-(methylsulfonyl)-1-phenyl-1,4,5,7-tetrahydro-6*H*-pyrazolo[3,4-*b*]pyridin-6-one **31**

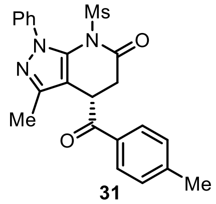

31

Following general procedure **7**, (*E*)-4-oxo-4-(*p*-tolyl)but-2-enoic pivalic anhydride **S25** (81.6 mg, 0.30 mmol), (2*S*,3*R*)-HyperSe **12** (7.1 mg, 0.02 mmol), CPME (2 mL) and N-(3-methyl-1-phenyl-1H-pyrazol-5-yl)methanesulfonamide **15** (50.3 mg, 0.20 mmol) for 16 h gave, after purification by Biotage® Selekt™ [Sfär HC 25 g, 80 mL.min^{-1}, Petrol : EtOAc (95:5 to 40:60 18 CV, 40:60 to 30:70 10 CV)], the title compound **31** (75.2 mg, 89 %) as a pale yellow oil. $[\alpha]_D^{20}$ −224 (*c* 1.2, $CHCl_3$); **Chiral HPLC analysis**, Chiralpak AD-H (85:15 hexane : IPA, flow rate 1mL.min^{-1}, 211 nm, 30 °C) t_R (4*S*): 23.0 min, t_R (4*R*): 45.3 min, 84:16 er; **IR** ν_{max} (film) 3021, 1726, 1672, 1605, 1364, 1155, 1034; **^{1}H NMR** (400 MHz, $CDCl_3$): δ 7.96 (d, 2H, J = 8.2 Hz), 7.48–7.42 (m, 4H), 7.36–7.32 (m, 3H), 4.79 (dd, 1H, J = 5.5, 2.4 Hz), 3.46 (s, 3H), 3.11 (dd, 1H, J = 16.0, 2.4 Hz), 2.98 (dd, 1H, J = 16.0, 5.4 Hz), 2.46 (s, 3H), 2.26 (s, 3H); **^{13}C{^{1}H} NMR** (126 MHz, $CDCl_3$): δ 197.6, 171.9, 145.8, 145.1, 140.6, 136.3, 132.6, 129.9, 129.3, 129.1, 128.0, 123.6, 106.6, 42.1, 39.2, 36.6, 21.9, 13.1; **HRMS** (ESI$^+$) $C_{22}H_{21}N_3O_3SNa$ [M+Na]$^+$ found 446.1139, requires 446.1145 (−1.3 ppm).

(*S*)-4-benzoyl-3-methyl-7-(methylsulfonyl)-1-phenyl-1,4,5,7-tetrahydro-6*H*-pyrazolo[3,4-*b*]pyridin-6-one **32**

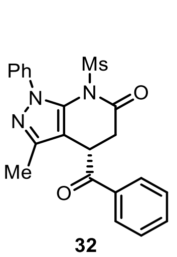

32

Following general procedure **7**, (*E*)-4-oxo-4-phenylbut-2-enoic pivalic anhydride **S26** (78.0 mg, 0.30 mmol), (2*S*,3*R*)-HyperSe **12** (7.1 mg, 0.02 mmol), CPME (2 mL) and *N*-(3-methyl-1-phenyl-1*H*-

pyrazol-5-yl)methanesulfonamide **15** (50.3 mg, 0.20 mmol) for 16 h gave, after purification by Biotage® Selekt™ [Sfär HC 25 g, 80 mL.min^{-1}, Petrol : EtOAc (90:10 to 40:60 18 CV, 40:60 to 30:70 10 CV)], the title compound **32** (76.4 mg, 94 %) as a yellow oil. $[\alpha]_D^{20}$ − 82 (*c* 3.0, $CHCl_3$); **Chiral HPLC analysis**, Chiralpak AD-H (75:25 hexane : IPA, flow rate 1mL.min^{-1}, 211 nm, 30 °C) t_R (4*S*): 12.3 min, t_R (4*R*): 19.1 min, 79:21 er; **IR** ν_{max} (film); 3059, 1728, 1678, 1595, 1364; **^{1}H NMR** (400 MHz, $CDCl_3$): δ 8.06–8.04 (app m, 2H), 7.68 (t, 1H, J = 7.4 Hz), 7.55 (t, 2H, J = 7.7 Hz), 7.48–7.42 (m, 4H), 7.36–7.32 (m, 1H), 4.81 (dd, 1H, J = 5.2, 2.4 Hz), 3.44 (s, 3H), 3.12 (dd, 1H, J = 16.0, 2.4 Hz), 2.99 (dd, 1H, J = 16.0, 5.4 Hz), 2.25 (s, 3H); **^{13}C{^{1}H} NMR** (126 MHz, $CDCl_3$): δ 198.1, 171.8, 145.1, 140.6, 136.3, 135.1, 134.6, 129.3, 129.2, 128.9, 128.0, 123.7, 106.4, 42.2, 39.1, 36.8, 13.1; **HRMS** (ESI$^+$) $C_{21}H_{19}N_3O_4SNa$ $[M+Na]^+$ found 432.0984, requires 432.0989 (−1.0 ppm).

(*S*)-7-(methylsulfonyl)-1,3-diphenyl-4-(trifluoromethyl)-1,4,5,7-tetrahydro-6*H*-pyrazolo[3,4-*b*]pyridin-6-one **33**

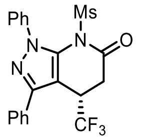

33

Following general procedure **7**, (*E*)-4,4,4-trifluorobut-2-enoic pivalic anhydride **14** (67.3 mg, 0.30 mmol), (2*S*, 3*R*)-HyperSe **12** (7.1 mg, 0.02 mmol), CPME (2 mL) and *N*-(1,3-diphenyl-1*H*-pyrazol-5-yl)methanesulfonamide **S35** (62.6 mg, 0.20 mmol) for 16 h gave, after purification by Biotage® Selekt™ [Sfär HC 25 g, 80 mL.min^{-1}, Petrol : EtOAc (95:5 to 40:60 18 CV, 40:60 to 30:70 10 CV)], the title compound **33** (59.3 mg, 68%) as a colourless oil. $[\alpha]_D^{20}$ −66 (*c* 1.2 $CHCl_3$); **Chiral HPLC analysis**, Chiralpak ID (88:12 hexane : IPA, flow rate 1mL.min^{-1}, 211 nm, 30 °C) t_R (4*R*): 16.4 min, t_R (4*S*): 19.7 min, 4:96 er; **IR** ν_{max} (film) 3063, 1732, 1597, 1368, 1163, 1136; **^{1}H NMR** (500 MHz, $CDCl_3$): δ 7.77–7.76 (m, 2H), 7.56–7.50 (m, 4H), 7.48–7.41 (m, 4H), 3.83 (app p, 1H, J = 8.5 Hz), 3.22–3.19 (m, 4H), 3.05 (dd, 1H, J = 17.1, 6.9 Hz); **^{13}C{^{1}H} NMR** (126 MHz, $CDCl_3$): δ 170.4, 149.6, 140.4, 137.3, 131.4, 129.4, 129.1, 129.0, 128.9, 127.6, 125.7 (q, $^1J_{CF}$ = 280.2 Hz), 124.6, 102.0, 42.9, 36.7, 35.2 (d, $^2J_{CF}$ = 30.5 Hz); **^{19}F{^{1}H} NMR** (377 MHz, $CDCl_3$): δ −69.80 (s, 3F); **HRMS** (ESI$^+$) $C_{20}H_{16}F_3N_3O_3SNa$ $[M+Na]^+$ found 458.0753, requires 458.0757 (−0.8 ppm).

(*S*)-3-methyl-1-phenyl-7-tosyl-4-(trifluoromethyl)-1,4,5,7-tetrahydro-6*H*-pyrazolo[3,4-*b*]pyridin-6-one **34**

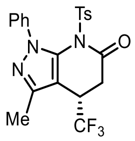

34

Following general procedure **7**, (*E*)-4,4,4-trifluorobut-2-enoic pivalic anhydride **14** (67.3 mg, 0.30 mmol), (2*S*,3*R*)-HyperSe **12** (7.1 mg, 0.02 mmol), CPME (2 mL) and 4-methyl-*N*-(3-methyl-1-phenyl-1*H*-pyrazol-5-yl)benzenesulfonamide **S3** (65.4 mg, 0.20 mmol) for 16 h gave, after purification by Biotage® Selekt^{TM} [Sfär HC 25 g, 80 mL.min^{-1}, Petrol : EtOAc 100:0 to 50:50 20 CV, 50:50 to 40:60 10 CV)], the title compound **34** (82.2 mg, 92 %) as a colourless oil. $[\alpha]_D^{20}$ −257 (*c* 0.8, $CHCl_3$); **Chiral HPLC analysis**, Chiralpak ID (90:10 hexane : IPA, flow rate 1mL.min^{-1}, 254 nm, 30 °C) t_R (4*R*): 23.2 min, t_R (4*S*): 39.8 min, 3:97 er; **IR** ν_{max} (film) 3071, 1730, 1506, 1373, 1163, 1126; **^{1}H NMR** (500 MHz, $CDCl_3$): δ 7.62 (d, 2H, J = 8.2 Hz), 7.50 (d, 2H, J = 7.8 Hz), 7.45 (t, 2H, J = 7.7 Hz), 7.38 (t, 1H, J = 7.3 Hz), 7.21 (d, 2H, J 8.3 Hz), 3.45 (app td, 1H, J = 7.7, 6.3, 2.8 Hz), 2.96–2.94 (m, 2H), 2.41 (s, 3H), 2.32 (s, 2H); **^{13}C{^{1}H} NMR** (126 MHz, $CDCl_3$): δ 168.5, 147.0, 146.1, 140.9, 137.2, 134.1, 130.3, 129.4, 129.1, 128.1, 125.2 (q $^1J_{CF}$ = 285.8 Hz), 124.2, 102.0, 35.6, 34.3 (q, $^2J_{CF}$ = 31.3 Hz), 21.9, 12.0; **^{19}F{^{1}H} NMR** (377 MHz, $CDCl_3$): δ −71.86 (s, 3F); **HRMS** (ESI$^+$) $C_{21}H_{19}F_3N_3O_3S$ $[M+H]^+$ found 450.1095, requires 450.1094 (+0.3 ppm).

(*S*)-1-(4-methoxyphenyl)-3-methyl-7-(methylsulfonyl)-4-(trifluoromethyl)-1,4,5,7-tetrahydro-6*H*-pyrazolo[3,4-*b*]pyridin-6-one **27**

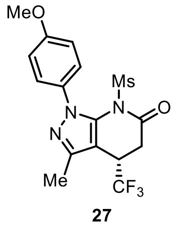

27

Following general procedure **7**, (*E*)-4,4,4-trifluorobut-2-enoic pivalic anhydride **14** (67.3 mg, 0.30 mmol), (2*S*,3*R*)-HyperSe **12** (7.1 mg, 0.02 mmol), CPME (2 mL) and *N*-(1-(4-methoxyphenyl)-3-methyl-1*H*-pyrazol-5-yl)methanesulfonamide **24** (56.2 mg, 0.20 mmol) for 16 h gave, after purification by Biotage® Selekt^{TM} [Sfär HC 25 g, 80 mL.min^{-1}, Petrol : EtOAc (90:10 to 30:70 18 CV, 30:70 to 20:80 10 CV)], the title compound **27** (75.5 mg, 94%) as a colourless oil. $[\alpha]_D^{20}$ −170 (*c* 1.2, $CHCl_3$); **Chiral HPLC analysis**, Chiralpak AD-H (88:12 hexane : IPA, flow rate 1mL.min^{-1}, 254 nm, 30 °C) t_R (4*R*): 21.6 min, t_R (4*S*): 29.5 min, 2:98 er; **IR** ν_{max} (film) 2982, 1732, 1518, 1366, 1157, 1115; **^{1}H NMR**

(500 MHz, $CDCl_3$): δ 7.36–7.34 (m, 2H), 6.97–6.96 (m, 2H), 3.83 (s, 3H), 3.56 (app p, 1H, J = 8.7 Hz), 3.19–3.15 (m, 4H), 3.03 (dd, 1H, J = 17.3, 7.4 Hz), 2.31 (s, 3H); **$^{13}C\{^1H\}$ NMR** (126 MHz, $CDCl_3$): δ 170.5, 159.5, 146.6, 136.4, 133.5, 125.7, 125.6 (q, $^1J_{CF}$ = 281.7 Hz), 114.4, 102.0, 55.6, 43.0, 35.9, 34.4 (q, $^2J_{CF}$ = 31.3 Hz), 12.0; **$^{19}F\{^1H\}$ NMR** (471 MHz, $CDCl_3$): δ −71.87 (s, 3F); **HRMS** (ESI^+) $C_{16}H_{16}F_3N_3O_4SNa$ $[M+Na]^+$ found 426.0701, requires 426.0706 (−1.1 ppm).

(*S*)-3-methyl-7-(methylsulfonyl)-1-(perfluorophenyl)-4-(trifluoromethyl)-1,4,5,7-tetrahydro-6*H*-pyrazolo[3,4-*b*]pyridin-6-one **35**

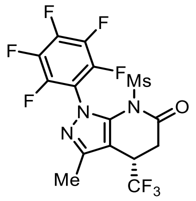

35

Following general procedure **7**, (*E*)-4,4,4-trifluorobut-2-enoic pivalic anhydride **14** (67.3 mg, 0.30 mmol (2*S*,3*R*)-HyperSe **12** (7.1 mg, 0.02 mmol), CPME (2 mL) and *N*-(3-methyl-1-(perfluorophenyl)-1*H*-pyrazol-5-yl)methanesulfonamide **S39** (68.2 mg, 0.20 mmol) for 16 h gave, after purification by Biotage® Selekt™ [Sfär HC 25 g, 80 mL.min^{-1}, Petrol : EtOAc (100:0 to 60:40 18 CV, 60:40 to 50:50 10 CV)], the title compound **35** (73.6 mg, 72 %) as a colourless oil. $[\alpha]_D^{20}$ −26 (*c* 0.6, $CHCl_3$); **Chiral HPLC analysis**, Chiralpak ID (99:1 hexane : IPA, flow rate 1mL.min^{-1}, 211 nm, 30 °C) t_R (4*R*): 14.9 min, t_R (4*S*): 18.1 min, 1:99 er; **IR** ν_{max} (film) 1736, 1518, 1369, 1169, 1125; **^1H NMR** (500 MHz, $CDCl_3$): δ 3.58 (qdd, 1H, J = 8.9, 7.3, 1.5 Hz), 3.29 (s, 3H), 3.17 (dd, 1H, J = 17.1, 1.5 Hz), 3.04 (dd, 1H, J = 17.1, 7.3 Hz), 2.33 (s, 3H); **$^{13}C\{^1H\}$ NMR** (126 MHz, $CDCl_3$): δ 168.9, 149.5, 145.2–143.0 (m), 143.7–141.5 (m), 143.2–140.9 (m), 139.7, 139.5–137.4 (m), 138.5–136.3 (m), 125.5 (q, $^1J_{CF}$ = 279.9 Hz), 122.2, 116.2 (app t, $^2J_{CF}$ = 13.2 Hz), 102.1, 35.3, 34.1 (q, $^2J_{CF}$ = 31.4 Hz), 12.0; **$^{19}F\{^1H\}$ NMR** (377 MHz, $CDCl_3$): δ −72.00 (s, 3F), −142.45 (dtd, 1F, J = 22.2, 6.5, 2.8 Hz), −149.32 (dt, 1F, J = 22.4, 6.0 Hz), −151.52 (t, 1F, J = 21.5 Hz), −160.24 (td, 1F, J = 21.8, 5.7 Hz), −161.58 (td, 1F, J = 21.7, 6.8 Hz); **HRMS** (ESI^+) $C_{15}H_9F_8N_3O_3SNa$ $[M+Na]^+$ found 486.0126, requires 486.0129 (−0.2 ppm).

(S)-1-(4-fluorophenyl)-3-methyl-7-(methylsulfonyl)-4-(trifluoromethyl)-1,4,5,7-tetrahydro-6*H*-pyrazolo[3,4-*b*]pyridin-6-one **36**

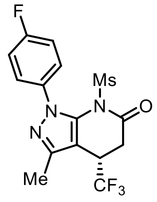

36

Following general procedure **7**, (*E*)-4,4,4-trifluorobut-2-enoic pivalic anhydride **14** (67.3 mg, 0.30 mmol), (2*S*,3*R*)-HyperSe **12** (7.1 mg, 0.02 mmol), CPME (2 mL) and N-(1-(4-fluorophenyl)-3-methyl-1H-pyrazol-5-yl)methanesulfonamide **S41** (53.8 mg, 0.20 mmol) for 16 h gave, after purification by Biotage® Selekt™ [Sfär HC 25 g, 80 mL.min^{-1}, Petrol : EtOAc (95:5 to 40:60 18 CV, 40:60 to 30:70 10 CV)] the title compound **36** (79.0 mg, 90%) as a colourless oil. $[\alpha]_D^{20}$ −153 (*c* 0.8, $CHCl_3$); **Chiral HPLC analysis**, Chiralpak AD-H (90:10 hexane : IPA, flow rate 1mL.min^{-1}, 254 nm, 30 °C) t_R (4*R*): 20.3 min, t_R (*4S*): 26.2 min, 3:97 er; **IR** ν_{max} (film) 3019, 1746, 1518, 1360, 1161, 1113; **^{1}H NMR** (500 MHz, $CDCl_3$): δ 7.43 (dd, 2H, J = 8.8, 4.7 Hz), 7.16 (app t, 2H, J = 8.4 Hz), 3.57 (app p, 1H, J = 8.6 Hz), 3.20–3.17 (m, 4H), 3.04 (dd, 1H, J = 17.2, 7.4 Hz), 2.32 (s, 3H); **^{13}C{^{1}H} NMR** (126 MHz, $CDCl_3$): δ 170.2, 163.1, 161.1, 147.0, 136.7 (d, $^1J_{CF}$ = 35.7 Hz), 125.9 (d, $^2J_{CF}$ = 8.6 Hz), 125.6 (q, $^1J_{CF}$ = 280.1 Hz), 116.4 (d, $^3J_{CF}$ = 22.9 Hz), 102.3, 42.7, 35.7, 34.3 (q, $^2J_{CF}$ = 31.3 Hz), 12.0; **^{19}F{^{1}H} NMR** (377 MHz, $CDCl_3$): δ −71.87 (s, 3F), −112.51 (s, 1F); **HRMS** (ESI$^+$) $C_{15}H_{13}F_4N_3O_3SNa$ [M+Na]$^+$ found 414.0500, requires 414.0506 (−1.4 ppm).

(S)-4-(3-methyl-7-(methylsulfonyl)-6-oxo-4-(trifluoromethyl)-4,5,6,7-tetrahydro-1*H*-pyrazolo[3,4-*b*]pyridin-1-yl)benzonitrile **37**

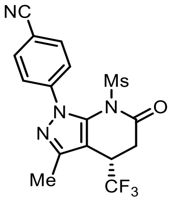

37

Following general procedure **7**, (*E*)-(*E*)-4,4,4-trifluorobut-2-enoic pivalic anhydride **14** (67.3 mg, 0.30 mmol), (2*S*,3*R*)-HyperSe **12** (7.1 mg, 0.02 mmol), CPME (2 mL) and *N*-(1-(4-cyanophenyl)-3-methyl-1*H*-pyrazol-5-yl)methanesulfonamide **S7** (55.3 mg, 0.20 mmol) for 16 h gave, after purification by Biotage® Selekt™ [Sfär HC 25 g, 60 mL.min^{-1}, Petrol : EtOAc (80:20 to 20:80 17 CV, 20:80 to 0:100 10 CV)], the title compound **37** (68.6 mg, 86%) as a white solid. **mp** 213–215 °C; $[\alpha]_D^{20}$ −76 (*c* 1.4, $CHCl_3$); **Chiral HPLC analysis**, Chiralpak IC (85:15 hexane : IPA, flow rate 1mL.min^{-1}, 211 nm, 40 °C) t_R (4*S*): 10.8 min, t_R (4*R*): 15.9 min, 82:18 er; **IR** ν_{max} (film) 2934, 2230, 1736, 1607, 1518, 1368,

1159, 1126; **^{1}H NMR** (400 MHz, $CDCl_3$): δ 7.76 (dt, 2H, J = 8.7, 2.1 Hz), 7.63 (dt, 2H, J = 8.7, 2.1 Hz), 3.62–3.57 (app m, 1H), 3.27 (s, 3H), 3.21 (dd, 1H, J = 17.0, 1.4 Hz), 3.08 (dd, 1H, J = 17.0, 7.4 Hz), 2.34 (s, 3H); **^{13}C{^{1}H} NMR** (126 MHz, $CDCl_3$): δ 169.8, 148.1, 144.1, 136.6, 133.5, 125.5 (q, $^1J_{CF}$ = 279.7 Hz), 123.8, 118.2, 111.6, 103.3, 42.1, 35.5, 34.3 (q, $^2J_{CF}$ = 31.4 Hz), 12.0; **^{19}F{^{1}H} NMR** (377 MHz, $CDCl_3$): δ – 71.76 (s, 3F); **HRMS** (ESI)$^+$ $C_{16}H_{13}F_3N_4O_3SNa$ [M+Na]$^+$ found 421.0553, requires 421.0558 (–2.8 ppm).

(*S*)-4-(3-methyl-7-(methylsulfonyl)-6-oxo-4-(4,4,5,5-tetramethyl-1,3,2-dioxaborolan-2-yl)-4,5,6,7-tetrahydro-1*H*-pyrazolo[3,4-*b*]pyridin-1-yl)benzonitrile **38**

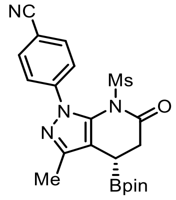

38

Following general procedure **7**, (*E*)-3-(4,4,5,5-tetramethyl-1,3,2-dioxaborolan-2-yl)acrylic pivalic anhydride **S6** (42.3 mg, 0.15 mmol), (2*S*,3*R*)-HyperSe **12** (7.1 mg, 0.02 mmol), K_2CO_3 (13.8 mg, 0.1 mmol), dioxane (1 mL) and *N*-(1-(4-cyanophenyl)-3-methyl-1*H*-pyrazol-5-yl)methanesulfonamide **S7** (27.7 mg, 0.1 mmol) for 16 h gave, after purification by Biotage® Selekt™ [Sfär HC 25 g, 90 mL.min^{-1}, CH_2Cl_2 : Et_2O (95:5 to 60:40 20 CV, 60:40 to 40:60 10 CV)] the title compound **38** (13.9 mg, (75 % NMRY) 30%) as a white solid. **mp** 199 – 201 °C; $[\alpha]_D^{20}$ –6 (*c* 0.4 $CHCl_3$); **Chiral HPLC analysis**, Chiralpak AD-H (90:10 hexane: IPA, flow rate 1mL.min^{-1}, 211 nm, 30 °C) t_R (4*S*): 34.8 min, t_R (4*R*): 47.3 min, 81:19 er; **IR** ν_{max} (film) 2980, 2228, 1740, 1607, 1360, 1348, 1163, 1138; **^{1}H NMR** (400 MHz, $CDCl_3$): δ 7.74–7.71 (m, 2H), 7.66–7.62 (m, 2H), 3.38 (s, 3H), 2.88 (dd, 2H, J = 5.1, 2.6 Hz), 2.55 (dd, 1H, J = 6.1, 4.1 Hz), 2.29 (s, 3H), 1.23 (app s, 12H); **^{13}C{^{1}H} NMR** (101 MHz, $CDCl_3$): δ 173.1, 146.7, 144.3, 134.6, 133.4, 123.1, 118.5, 111.6, 110.7, 84.8, 42.3, 37.7, 25.1, 24.6, 12.4; **^{11}B{^{1}H} NMR** (128 MHz, $CDCl_3$): δ 33.00; **HRMS** (ESI$^+$) $C_{21}H_{26}BN_4O_5S$ [M+H]$^+$ found 457.1702, requires 457.1712 (–2.08 ppm).

(*S*)-3-methyl-7-(methylsulfonyl)-1-(*o*-tolyl)-4-(trifluoromethyl)-1,4,5,7-tetrahydro-6*H*-pyrazolo[3,4-*b*]pyridin-6-one **39**

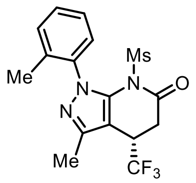

39

Following general procedure **7**, (*E*)-4,4,4-trifluorobut-2-enoic pivalic anhydride **14** (67.3 mg, 0.30 mmol), (2*S*,3*R*)-HyperSe **12** (7.1 mg, 0.02 mmol), CPME (2 mL) and *N*-(3-methyl-1-(*o*-tolyl)-1*H*-pyrazol-5-yl)methanesulfonamide **S46** (53.0 mg, 0.20 mmol) for 16 h gave, after purification by Biotage® Selekt™ [Sfär HC 25 g, 80 mL.min^{-1}, Petrol : EtOAc (95:5 to 60:40 20 CV, 60:40 to 40:60 10 CV)], the title compound **39** (37.5 mg, 48%) as a white solid. **mp** 214–215 °C (*dec.*); $[\alpha]_D^{20}$ –94 (*c* 0.4, $CHCl_3$); **Chiral HPLC analysis**, Chiralpak AD-H (92:8 hexane : IPA, flow rate 1mL.min^{-1}, 211 nm, 30 °C) t_R (4*S*): 15.3 min, t_R (4*R*): 20.3 min, >99:1 er; **IR** ν_{max} (film) 3032, 1734, 1581, 1364, 1159, 1126; **^{1}H NMR** (500 MHz, DMSO-d_6): δ 7.38–7.29 (m, 4H), 4.09 (dp, 1H, J = 17.9, 8.6 Hz), 3.45 (dd, 1H, J = 17.7, 7.5 Hz), 2.98–2.93 (m, 4H), 2.25 (s, 3H), 2.20 (br s, 3H); **^{13}C{^{1}H} NMR** (126 MHz, DMSO-d_6): δ 170.9, 146.0, 138.6, 137.3, 135.5, 131.5, 128.8, 126.3 (q, $^1J_{CF}$ = 280.0 Hz), 126.0, 101.7, 43.5, 35.4, 32.5 (q, $^2J_{CF}$ = 29.9 Hz), 18.1, 11.6; **^{19}F{^{1}H} NMR** (470 MHz, DMSO-d_6) δ –70.63 (s, 3F); **HRMS** (ESI$^+$) $C_{16}H_{16}F_3N_3O_3SNa$ $[M+Na]^+$ found 410.0761, requires 410.0762 (–0.2 ppm).

5.5. Product Derivatizations

(*S*)-3-methyl-1-phenyl-4-(trifluoromethyl)-1,4,5,7-tetrahydro-6H-pyrazolo[3,4-b]pyridin-6-one **41**

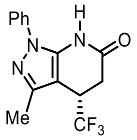

41

Following general procedure **1**, (*S*)-3-methyl-7-(methylsulfonyl)-1-phenyl-4-(trifluoromethyl)-1,4,5,7-tetrahydro-6*H*-pyrazolo[3,4-*b*]pyridin-6-one **19** (150 mg, 0.4 mmol), THF (1 mL), and 10% aq NaOH (1 mL, 0.4 mmol) for 1 h gave, after purification by Biotage® Selekt™ [Sfär HC 25 g, 80 mL.min^{-1} Petrol : EtOAc (95:5 to 50:50 20 CV, 50:50 to 30: 70 10 CV)], the title compound **41** (62.0 mg, 53%) as a white solid. **mp** 76–78 °C; $[\alpha]_D^{20}$ – 19 (1.4 *c*, $CHCl_3$); **Chiral HPLC analysis**, Chiralpak AD-H (92:8 hexane : IPA, flow rate 1mL.min^{-1}, 211 nm, 30 °C) t_R (4*S*): 10.7 min, t_R (4*R*): 13.8 min, 98:2 er; **IR** ν_{max} (solid) 3173, 2930, 1694, 160, 1553, 1159, 1119**; ^{1}H NMR** (400 MHz, $CDCl_3$): δ 8.13 (s, 1H), 7.50–7.46 (m, 2H), 7.44–7.37 (m, 3H), 3.58 (td, 1H, J = 8.8, 4.9, 4.8 Hz), 2.92–2.91 (app m, 2H), 2.27

(s, 3H); **^{13}C{^{1}H} NMR** (126 MHz, $CDCl_3$): δ 167.8, 147.6, 139.0, 137.1, 129.8, 127.6, 126.5 (q, $^{1}J_{CF}$ = 280.5 Hz), 123.2, 93.7, 34.6 (q, $^{2}J_{CF}$ = 30.5 Hz), 31.5, 12.2; **^{19}F{^{1}H} NMR** (377 MHz, $CDCl_3$): δ –73.54 (s, 3F); **HRMS** (ESI^+) $C_{14}H_{12}F_3ON_3Na$ $[M+Na]^+$ found 318.0829, requires 318.0825 (+1.3 ppm).

(*S*)-*N*-(3-methyl-1-phenyl-4-(1,1,1-trifluoro-4-hydroxybutan-2-yl)-1*H*-pyrazol-5 yl)methanesulfonamide **40**

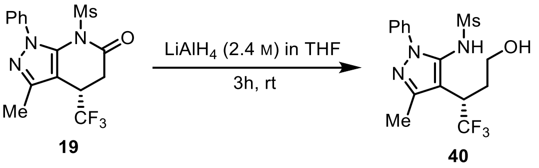

Following literature, [14] a suspension of $LiAlH_4$ (2.4 M) in THF (0.34 mL, 0.8 mmol, 2.0 equiv.) was added dropwise to a mixture of (*S*)-3-methyl-7-(methylsulfonyl)-1-phenyl-4-(trifluoromethyl)-1,4,5,7-tetrahydro-6*H*-pyrazolo[3,4-*b*]pyridin-6-one **19** (150 mg, 0.4 mmol, 1.0 equiv.) in THF (0.8 mL). The solution was stirred under nitrogen for 3 h and then the $LiAlH_4$ was quenched by slowly adding water. The mixture was concentrated under reduced pressure to remove THF and then extracted with EtOAc and brine. The organic layer was dried (Na_2SO_4), filtered, and concentrated under reduced pressure. The crude was then purified by Biotage® Selekt™ [Sfär HC 25 g, 80 mL.min^{-1}, Petrol : EtOAc (90:10 to 50:50 CV, 50:50 to 0:100 10 CV)] to afford the title compound **40** (79.8 mg, 56%) as a white solid. **mp** 129–133 °C; $[\alpha]_D^{20}$ +10.6 (*c* 1.7, $CHCl_3$); **Chiral HPLC analysis**, Chiralpak ID (90:10 hexane : IPA, flow rate 1mL.min^{-1}, 211 nm, 30 °C) t_R (4*S*): 14.6 min, t_R (4*R*): 17.9 min, 98:2 er; **IR** ν_{max} (film) 3250, 1599, 1503, 1325, 1152, 1117; **^{1}H NMR** (500 MHz, $CDCl_3$): δ 7.54–7.50 (m, 4H), 7.47–7.42 (m, 1H), 6.43 (br s, 1H), 3.81–3.72 (m, 2H), 3.53 (td, 1H, J = 11.0, 3.3 Hz), 2.44 (s, 3H), 2.36 (s, 3H), 2.30–2.17 (m, 2H); **^{13}C{^{1}H} NMR** (126 MHz, $CDCl_3$): δ 148.4, 138.3, 133.4, 129.6, 128.8, 127.4 (q, $^{1}J_{CF}$ = 279.5 Hz), 125.4, 111.1, 58.7, 41.8, 37.1 (q, $^{2}J_{CF}$ = 28.4 Hz), 29.0, 14.4; **^{19}F{^{1}H} NMR** (376 MHz, $CDCl_3$): δ –68.91 (s, 3F); **HRMS** (ESI^+) $C_{15}H_{19}F_3N_3O_3S$ $[M+H]^+$ found 378.1097, requires 378.1094 (+0.9 ppm).

1,2-addition product: (2*E*,4*E*)-*N*-(3-methyl-1-phenyl-1*H*-pyrazol-5-yl)-*N*-(methylsulfonyl)hexa-2,4-dienamide **S55**

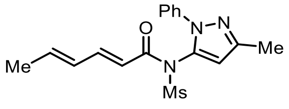

S55

Following general procedure **7**, (2*E*,4*E*)-hexa-2,4-dienoic pivalic anhydride **S10** (58.8 mg, 0.30 mmol), (2*S*,3*R*)-HyperSe **12** (7.1 mg, 0.02 mmol), CPME (2 mL) and *N*-(3-methyl-1-phenyl-1*H*-pyrazol-5-yl)methanesulfonamide **15** (50.3 mg, 0.20 mmol) for 16 h gave, after purification by Biotage® Selekt™ [Sfär HC 25 g, 80 mL.min^{-1}, Petrol : EtOAc (100:0 to 60:40 20 CV, 60:40 to 50:50 10 CV)], the title compound **S55** (94.2 mg, *quant.*) as a colourless oil. **IR** ν_{max} (film) 3013, 1680, 1634, 1599, 1358, 1128;

^{1}**H NMR** (400 MHz, $CDCl_3$): δ 7.46–7.38 (m, 6H), 6.36 (s, 1H), 6.33–6.25 (m, 1H), 6.21–6.17 (m, 1H), 5.82 (d, 1H, J = 15.4 Hz), 2.96 (s, 3H), 2.39 (s, 3H), 1.87 (d, 3H, J = 6.7 Hz); ^{13}**C{^{1}H} NMR** (126 MHz, $CDCl_3$): δ 166.2, 149.9, 149.0, 143.5, 138.0, 133.1, 129.8, 129.5, 129.0, 125.5, 116.3, 108.4, 42.2, 19.0, 14.3; **HRMS** (ESI$^+$) $C_{17}H_{19}N_3O_3SNa$ $[M+Na]^+$ found 368.1044, requires 368.1039 (+1.3 ppm).

5.6. Pyrazolone Conjugate Addition Products

(*R*)-1,3,4-Triphenyl-4,5-dihydropyrano[2,3-*c*]pyrazol-6(1*H*)-one **44**

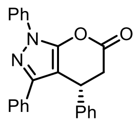

44

Following general procedure **8**, 2,5-Diphenyl-2,4-dihydro-3*H*-pyrazol-3-one **43** (56.7 mg, 0.24 mmol), (*E*)-cinnamic pivalic anhydride **42** (46.2 mg, 0.20 mmol), (2*S*,3*R*)-HyperSe **12** (7.1 mg, 0.02 mmol), CPME (2.0 mL) and Na_2CO_3 (21.2 mg, 0.20 mmol) for 16 h gave, after purification by flash column chromatography (90:10 Petrol : EtOAc, R_f 0.28), the title compound **44** (37.7 mg, 86% (NMR); 58% (isolated)) as a yellow solid. **mp** 133−136 °C; $[\alpha]_D^{20}$–48 (*c* 1.8, $CHCl_3$); **Chiral HPLC analysis:** Chiralpak IA (98:2 hexane : IPA, flow rate 1.0 mL·min^{-1}, 254 nm, 30 °C) t_R(4*S*): 14.3 min, t_R(4*R*): 16.9 min, 2:98 er; **IR** ν_{max} (solid) 1771, 1508, 1107; **^{1}H NMR** (300 MHz, $CDCl_3$): δ 7.93–7.90 (m, 2H), 7.59–7.56 (m, 2H), 7.51 (app t, 2H, J = 8.0 Hz), 7.38–7.22 (m, 10H), 4.53 (dd, 1H, J = 7.3, 2.4 Hz), 3.25 (dd, 1H, J = 15.7, 7.3 Hz), 3.07 (dd, 1H, J = 15.7, 2.4 Hz); **^{13}C{^{1}H} NMR** (126 MHz, $CDCl_3$): δ 165.4, 148.2, 147.7, 141.1, 137.8, 132.6, 129.5, 129.4, 128.8, 128.5, 127.9, 127.1, 127.0, 126.9, 121.4, 98.6, 38.8, 35.2; **HRMS** (ESI$^+$) $C_{24}H_{19}N_2O_2$ [M+H]$^+$ found 367.1441, requires 367.1441 (–0.1 ppm).

Note that recrystallization was attempted on the optimised conditions with various solvents including pentane, ethyl acetate, acetonitrile, diethyl ether and ethanol, however all attempts were unsuccessful in being able to generate crystalline material from the reaction mixture.

(*R*)-3-(4-Bromophenyl)-1,4-diphenyl-4,5-dihydropyrano[2,3-*c*]pyrazol-6(1*H*)-one **47**

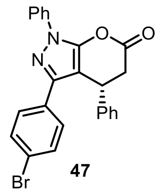

47

Following general procedure **8**, 5-(4-bromophenyl)-2-phenyl-2,4-dihydro-3*H*-pyrazol-3-one **S50** (75.6 mg, 0.24 mmol), (*E*)-cinnamic pivalic anhydride **42** (46.4 mg, 0.20 mmol), (2*S*,3*R*)-HyperSe **12** (7.1 mg, 0.02 mmol), CPME (2.0 mL) and Na_2CO_3 (21.2 mg, 0.200 mmol) for 16 h gave, after purification by flash column chromatography (13:1 Petrol : EtOAc, R_f 0.31), the title compound **47** (32.0 mg, 67% (NMR); 36% (isolated)) as a yellow solid. **mp** 181–183 °C; $[\alpha]_D^{20}$–5 (*c* 0.3, $CHCl_3$); **Chiral HPLC analysis:** Chiralpak IB (99:1 hexane : IPA, flow rate 1.0 mL·min^{-1}, 220 nm, 30 °C) t_R(4*S*): 19.4 min,

$t_R(4R)$: 39.1 min, 4:96 er; **IR** ν_{max} (film) 1796, 1599, 1096; **^{1}H NMR** (400 MHz, $CDCl_3$): δ 7.90–7.88 (m, 2H), 7.53–7.49 (m, 2H), 7.44 (s, 4H), 7.38–7.33 (m, 1H), 7.31–7.27 (m, 1H), 7.22–7.20 (m, 2H), 4.49 (dd, 1H, J = 7.4, 2.4 Hz), 3.26 (dd, 1H, J 15.7, 7.4 Hz), 3.07 (dd, 1H, J = 15.7, 2.5 Hz); **^{13}C{^{1}H} NMR** (126 MHz, $CDCl_3$): δ 165.1, 148.3, 146.6, 140.9, 137.7, 131.9, 131.5, 129.6, 129.5, 128.4, 128.1, 127.3, 126.9, 122.7, 121.4, 98.6, 38.7, 35.2; **HRMS** (ESI^+) $C_{24}H_{18}{}^{79}BrN_2O_2$ $[M+H]^+$ found 445.0542, requires 445.0546 (–0.9 ppm).

(*R*)-3-(4-Methoxyphenyl)-1,4-diphenyl-4,5-dihydropyrano[2,3-*c*]pyrazol-6(1*H*)-one **48**

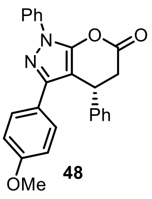

48

Following general procedure **8**, 5-(4-methoxyphenyl)-2-phenyl-2,4-dihydro-3*H*-pyrazol-3-one **S51** (63.9 mg, 0.24 mmol), (*E*)-cinnamic pivalic anhydride **42** (46.4 mg, 0.20 mmol), (2*S*,3*R*)-HyperSe **12** (7.1 mg, 0.02 mmol), CPME (2.0 mL) and Na_2CO_3 (21.2 mg, 0.20 mmol) for 16 h gave, after purification by flash column chromatography (90:10 Petrol : EtOAc, R_f 0.24), the title compound **48** (23.8 mg, 93% (NMR); 30% (isolated)) as a white solid. **mp** 147−149 °C; $[\alpha]_D^{20}$ –31 (*c* 0.9, $CHCl_3$); **Chiral HPLC analysis:** Chiralpak IA (95:5 hexane : IPA, flow rate 1.0 mL·min^{-1}, 270 nm, 30 °C) $t_R(4S)$: 16.3 min, $t_R(4R)$: 19.1 min, 2:98 er; **IR** ν_{max} (film) 1790, 1597, 1245, 1096; **^{1}H NMR** (500 MHz, $CDCl_3$): δ 7.91–7.89 (m, 2H), 7.52–7.49 (m, 4H), 7.37–7.32 (m, 3H), 7.30–7.27 (m, 1H), 7.24 – 7.23 (m, 2H), 7.91–7.89 (m, 2H), 4.49 (dd, 1H, J = 7.4, 2.1 Hz), 3.79 (s, 3H), 3.24 (dd, 1H, J = 15.7, 7.5 Hz), 3.06 (dd, 1H, J = 15.7, 2.3 Hz); **^{13}C{^{1}H} NMR** (126 MHz, $CDCl_3$): δ 165.4, 159.9, 148.1, 147.5, 141.2, 137.9, 129.5, 129.4, 128.2, 127.9, 126.99, 126.97, 125.2, 121.3, 114.2, 98.1, 55.4, 38.8, 35.1; **HRMS** (ESI^+) $C_{25}H_{21}O_3N_2$ $[M+H]^+$ found 397.1547, requires 397.1544 (+1.8 ppm).

(*R*)-1,4-Diphenyl-3-(4-(trifluoromethyl)phenyl)-4,5-dihydropyrano[2,3-*c*]pyrazol-6(1*H*)-one **49**

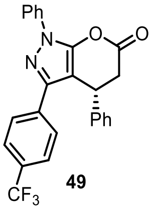

49

Following general procedure **8**, 2-phenyl-5-(4-(trifluoromethyl)phenyl)-2,4-dihydro-3*H*-pyrazol-3-one **S53** (73.0 mg, 0.24 mmol), (*E*)-cinnamic pivalic anhydride **42** (46.4 mg, 0.20 mmol), (2*S*,3*R*)-HyperSe **12** (7.1 mg, 0.02 mmol), CPME (2.0 mL) and Na_2CO_3 (21.2 mg, 0.20 mmol) for 16 h gave, after purification by Biotage® Selekt™ [Sfär HC 10 g, 50 mL·min^{-1}, Petrol : EtOAc (95:5 to 75:25 10 CV,

75:25 to 50:50 15 CV)], the title compound **49** (40.5 mg, 60 % (NMR), 47% (isolated)) as a white solid. **mp** 220–223 °C; $[\alpha]_D^{20}$ –34 (*c* 0.5, $CHCl_3$); **Chiral HPLC analysis:** Chiralpak IB (98:2 hexane : IPA, flow rate 1.0 mL·min^{-1}, 211 nm, 30 °C) t_R(4*S*): 13.4 min, t_R(4*R*): 22.2 min 4:96 er; **IR** ν_{max} (film) 1796, 1599, 1109; **^{1}H NMR** (500 MHz, $CDCl_3$): δ 7.90 (m, 2H, dd J = 8.6, 1.1 Hz), 7.68 (d, 2H, J = 8.1 Hz), 7.57–7.51 (m, 4H), 7.39–7.35 (m, 3H), 7.32–7.28 (m, 1H), 7.24–7.22 (m, 2H), 4.53 (dd, 1H, J = 7.4, 2.4 Hz), 3.27 (dd, 1H, J = 15.7, 7.4 Hz), 3.08 (dd, 1H, J = 15.7, 2.5 Hz); **^{13}C{^{1}H} NMR** (126 MHz, $CDCl_3$): δ 165.0, 148.5, 146.2, 140.8, 137.6, 136.0, 130.2 (q, $^2J_{CF}$ = 32.9 Hz), 129.7, 129.5, 128.2**,** 127.5, 127.0, 126.9, 125.7 (q, $^3J_{CF}$ = 3.9 Hz), 125.3 (q, $^1J_{CF}$ = 274.8 Hz), 121.5, 99.0, 38.7, 35.2; **^{19}F{^{1}H} NMR** (470 MHz, $CDCl_3$): δ –62.65 (s, 3F); **HRMS** (ESI$^+$) $C_{25}H_{18}F_3N_2O_2$ [M+H]$^+$ found 435.1305, requires 435.1315 (–2.3 ppm).

Ethyl (*S*)-6-oxo-1,3-diphenyl-1,4,5,6-tetrahydropyrano[2,3-*c*]pyrazole-4-carboxylate **50**

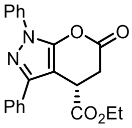

50

Following general procedure **8**, (*E*)-(*E*)-4-ethoxy-4-oxobut-2-enoic pivalic anhydride **S18** (45.6 mg, 0.2 mmol), (2*S*,3*R*)-HyperSe **12** (7.1 mg, 0.01 mmol), CPME (2 mL), 2,5-diphenyl-2,4-dihydro-3H-pyrazol-3-one **43** (50.3 mg, 0.24 mmol) and Na_2CO_3 (21.2 mg, 0.2 mmol) for 16 h gave, after purification by Biotage® Selekt™ [25 g, 100 mL.min^{-1}, Petrol : EtOAc (100:0 to 75:25 10 CV, 75:25 to 50:50 10 CV)], the title compound **50** (21.4 mg, 70% (NMR), 30% isolated) as a white solid. **mp** 113–115 °C; $[\alpha]_D^{20}$ – 85 (*c* 0.1, $CHCl_3$); **Chiral HPLC analysis**, Chiralpak IA (98:2 hexane : IPA, flow rate 1mL.min^{-1}, 220 nm, 30 °C) t_R (4*R*): 23.1 min, t_R (4*S*): 25.9 min, 3:97 er; **IR** ν_{max} (film); 2984, 1802, 1732, 1599, 1134; **^{1}H NMR** (400 MHz, $CDCl_3$): δ 7.94 (app d, 2H, J = 7.1 Hz), 7.85 (app d, 2H, J = 7.6 Hz), 7.48 (app q, 4H, J = 7.5 Hz), 7.42–7.38 (m, 1H), 7.34 (t, 1H, J = 7.4 Hz), 4.27–4.13 (m, 3H, m), 3.17 (dd, 1H, J = 16.1, 2.0 Hz), 2.92 (dd, 1H, J = 16.1, 6.6 Hz), 1.22 (t, 3H, J = 7.1 Hz); **^{13}C{^{1}H} NMR** (126 MHz, $CDCl_3$): δ 171.4, 164.5, 148.3, 147.5, 137.6, 132.4, 129.4, 128.9, 128.8, 127.3, 127.2, 121.5, 94.4, 62.3, 35.9, 32.5, 14.0; **HRMS** (ESI$^+$) $C_{21}H_{18}N_2O_4$ [M+Na]$^+$ found 385.1161, requires 385.1159 (+0.6 ppm).

(*S*)-4-Methyl-1,3-diphenyl-4,5-dihydropyrano[2,3-*c*]pyrazol-6(1*H*)-one **51**

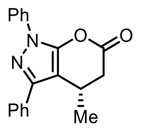

51

Following general procedure **8**, 2,5-Diphenyl-2,4-dihydro-3*H*-pyrazol-3-one **43** (56.7 mg, 0.24 mmol), (*E*)-crotonic pivalic anhydride **S9** (34.0 mg, 0.20 mmol), (2*S*,3*R*)-HyperSe **12** (7.1 mg, 0.02 mmol), CPME (2.0 mL) and Na_2CO_3 (21.2 mg, 0.20 mmol) for 16 h gave, after purification by Biotage® Selekt™ [Sfär HC 10 g, 40 mL·min^{-1}, Petrol : EtOAc (95:5 to 90:10 200 mL)], the title compound **51** (22.1 mg, 41% (NMR), 36% (isolated)) as a yellow solid. **mp** 127–129 °C; $[\alpha]_D^{20}$ +3 (*c* 0.6, $CHCl_3$); **Chiral HPLC analysis:** Chiralpak IA (99:1 hexane : IPA, flow rate 1.0 mL·min^{-1}, 220 nm, 30 °C) t_R(4*R*): 15.5 min, t_R(4*S*): 17.9 min, 1:91 er; **IR** ν_{max} (film) 2961, 1790, 1597, 1119; **^{1}H NMR** (400 MHz, $CDCl_3$): δ 7.85–7.80 (m, 4H), 7.50–7.44 (m, 4H), 7.40–7.37 (m, 1H), 7.34–7.30 (m, 1H,), 3.49 (pd, 1H, J = 6.9, 2.7 Hz), 3.01 (dd, 1H, J = 15.8, 6.7 Hz), 2.79 (dd, 1H, J = 15.8, 2.7 Hz), 1.38 (d, 3H, J = 7.0 Hz); **^{13}C{^{1}H} NMR** (126 MHz, $CDCl_3$): δ 166.2, 147.0, 146.8, 137.8, 133.2, 129.4, 128.9, 128.5, 127.0, 126.8, 121.3, 101.7, 37.4, 24.6, 21.6; **HRMS** (ESI$^+$) $C_{25}H_{17}O_2N_2$ [M+H]$^+$ found 305.1276, requires 305.1285 (–2.8 ppm).

(*R*)-3-Methyl-1,4-diphenyl-4,5-dihydropyrano[2,3-*c*]pyrazol-6(1*H*)-one **52**

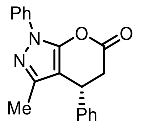

52

Following general procedure **8**, 5-methyl-2-phenyl-2,4-dihydro-3*H*-pyrazol-3-one **S8** (41.8 mg, 0.24 mmol), (*E*)-cinnamic pivalic anhydride **42** (46.4 mg, 0.20 mmol) and (2*S*,3*R*)-HyperSe **12** (7.1 mg, 0.02 mmol), CPME (2.0 mL) and Na_2CO_3 (21.2 mg, 0.20 mmol) for 16 h gave, after purification by Biotage® Selekt™ [Sfär HC 10 g, 50 mL.min^{-1}, Petrol : EtOAc (90:10 to 60:40 500 mL)], the title compound **52** (15.5 mg, 52% (NMR), 25% (isolated)) as an off-white oil. $[\alpha]_D^{20}$ –75 (*c* 0.4, $CHCl_3$); **Chiral HPLC analysis:** Chiralcel OD-H (90:10 hexane : IPA, flow rate 1.0 mL·min^{-1}, 254 nm, 30 °C) t_R(4*S*): 14.5 min, t_R(4*R*): 17.0 min, 11:89 er; **IR** ν_{max} (film) 1794, 1512, 1217; **^{1}H NMR** (500 MHz, $CDCl_3$): δ 7.76–7.74 (m, 2H), 7.47 – 7.44 (m, 2H), 7.38–7.35 (m, 2H), 7.31–7.28 (m, 2H), 7.23–7.21 (m, 2H), 4.26 (app t, 1H, J = 6.6 Hz), 3.20 (dd, 1H, J = 16.0, 7.0 Hz), 2.99 (dd, 1H, J = 16.0, 6.2 Hz), 1.97 (s, 3H); **^{13}C{^{1}H} NMR** (126 MHz, $CDCl_3$): δ 165.8, 147.1, 146.1, 141.0, 137.7, 129.4, 129.3, 127.9, 127.2, 126.7, 121.1, 99.5, 38.2, 34.8, 13.1; **HRMS** (ESI$^+$) $C_{19}H_{17}N_2O_2$ [M+H]$^+$ found 305.1286, requires 305.1285 (+0.5 ppm).

(*R*)-1-(*Tert*-butyl)-3,4-diphenyl-4,5-dihydropyrano[2,3-*c*]pyrazol-6(1*H*)-one **53**

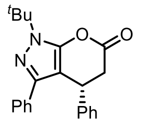

52

Following general procedure **8**, 2-(*tert*-butyl)-5-phenyl-2,4-dihydro-3*H*-pyrazol-3-one **S54** (51.9 mg, 0.24 mmol), (*E*)-cinnamic pivalic anhydride **42** (46.4 mg, 0.20 mmol,), (2*S,*3*R*)-HyperSe **12** (7.1 mg, 0.02 mmol), CPME (2.0 mL) and Na_2CO_3 (21.2 mg, 0.20 mmol) for 16 h gave, after purification by Biotage® Selekt™ [Sfär HC 10 g, 40 mL·min^{-1}, Petrol : EtOAc (95:5 to 90:10 400 mL)], the title compound **53** (7.40 mg, 19 % (NMR), 11% (isolated)) as a clear oil with spectroscopic data in accordance with the literature. [36] $[\alpha]_D^{20}$ +1 (*c* 0.4, $CHCl_3$) {Lit. [36] $[\alpha]_D^{20}$ +19 (*c* 0.1, $CHCl_3$)}; **Chiral HPLC analysis:** Chiralpak IC (99.9:0.1 hexane : IPA, flow rate 1.0 mL·min^{-1}, 254 nm, 30 °C) t_R(4*S*): 11.3 min, t_R(4*R*): 12.8 min, 8:92 er; **IR** ν_{max} (film) 1790, 1597, 1103; **^{1}H NMR** (500 MHz, $CDCl_3$): δ 7.49–7.47 (m, 2H), 7.33–7.30 (m, 2H), 7.28-7.21 (m, 4H), 7.16–7.14 (m, 2H), 4.43 (dd, 1H, J = 7.4, 2.2 Hz), 3.16 (dd, 1H, J = 15.6, 7.4 Hz), 2.99 (dd, 1H, J = 15.6, 2.3 Hz), 1.73 (s, 9H).

(*S*)-1,3-diphenyl-4-(trifluoromethyl)-4,5-dihydropyrano[2,3-*c*]pyrazol-6(1*H*)-one **54**

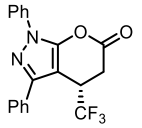

54

Following general procedure **8**, 2,5-diphenyl-2,4-dihydro-3*H*-pyrazol-3-one **43** (56.7 mg, 0.24 mmol), (2*S*,3*R*)-HyperSe **12** (7.1 mg, 0.02 mmol), (*E*)-4,4,4-trifluorobut-2-enoic pivalic anhydride **14** (44.8 mg, 0.20 mmol), CPME (2mL) and Na_2CO_3 (21.2 mg, 0.20 mmol) for 16 h gave, after purification by Biotage® Selekt^{TM} [SfärHC 10 g, 50 mL.min^{-1}, Petrol : EtOAc (95:5 to 80:20 10 CV, 80:20 to 50:50 10 CV)], the title compound **54** (30.8 mg, 63% (NMR), 43% (isolated)) as a white solid. **mp** 151−152 °C; $[\alpha]_D^{20}$+ 6 (*c* 1.2, $CHCl_3$); **Chiral HPLC analysis**, Chiralpak IA (95:5 hexane : IPA, flow rate 1mL.min^{-1}, 220 nm, 30 °C) t_R (4*R*): 11.2 min, t_R (4*S*): 18.3 min, 7:93 er; **IR** ν_{max} (film); 2926, 1802, 1601, 1512, 1121; **^{1}H NMR** (500 MHz, $CDCl_3$): δ 7.81 (dd, 4H, J = 15.8, 7.5 Hz), 7.51–7.40 (m, 5H), 7.36 (t, 1H, J = 7.4 Hz), 4.05 (pd, 1H, J = 7.8, 1.5 Hz), 3.23 (dd, 1H, J = 17.0, 2.5 Hz), 3.07 (dd, 1H, J = 16.9, 7.3 Hz); **^{13}C{^{1}H} NMR** (126 MHz, $CDCl_3$): δ 163.1, 148.8, 148.2, 137.3, 132.2, 129.5, 129.04, 128.96, 127.6, 127.0, 125.9 (q, $^1J_{CF}$ = 278.6 Hz), 121.7, 90.3, 35.1 (q, $^2J_{CF}$ = 30.8 Hz), 29.6; **^{19}F{^{1}H} NMR** (377 MHz, $CDCl_3$): δ −72.44 (s, 3F); **HRMS** (ESI$^+$) $C_{19}H_{13}F_3N_2O_2Na$ [M+Na]$^+$ found 381.0822, requires 381.0821 (+0.2 ppm).

(R)-4-(4-Methoxyphenyl)-1,3-diphenyl-4,5-dihydropyrano[2,3-*c*]pyrazol-6(1*H*)-one **55**

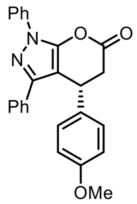

55

Following general procedure **8**, 2,5-Diphenyl-2,4-dihydro-3*H*-pyrazol-3-one **43** (56.7 mg, 0.24 mmol), (*E*)-4-Methoxycinnamic pivalic anhydride **S13** (52.5 mg, 0.20 mmol), (2*S*,3*R*)-HyperSe **12** (7.1 mg, 0.02 mmol), CPME (2.0 mL) and Na_2CO_3 (21.2 mg, 0.20 mmol) for 16 h gave, after purification by flash column chromatography (90:10 Petrol : EtOAc, R_f 0.21), the title compound **55** (43.6 mg, 82% (NMR); 55% (isolated)) as a yellow solid. **mp** 189−192 °C; $[\alpha]_D^{20}$ −64 (*c* 0.5, $CHCl_3$); **Chiral HPLC analysis:** Chiralpak IA (97:3 hexane : IPA, flow rate 1.0 mL·min^{-1}, 254 nm, 30 °C) t_R(4*S*): 17.2 min, t_R(4*R*): 22.5 min, 1:99 er; **IR** ν_{max} (film) 1796, 1510, 1096; **^{1}H NMR** (500 MHz, $CDCl_3$): δ 7.92-7.90 (m, 2H), 7.60–7.58 (m, 2H), 7.53–7.49 (m, 2H), 7.37–7.28 (m, 4H), 7.16–7.13 (m, 2H), 6.89–6.86 (m, 2H), 4.49 (dd, 1H, J = 7.2, 2.1 Hz), 3.79 (s, 3H), 3.22 (dd, 1H, J = 15.6, 7.3 Hz), 3.04 (dd, 1H, J = 15.6, 2.2 Hz); **^{13}C{^{1}H} NMR** (126 MHz, $CDCl_3$): δ 165.5, 159.2, 148.1, 147.6, 137.8, 133.1, 132.6, 129.4, 128.8, 128.5, 128.1, 127.1, 126.9, 121.3, 114.8, 99.0, 55.4, 39.1, 34.4; **HRMS** (ESI$^+$) $C_{25}H_{21}O_3N_2$ $[M+H]^+$ found 397.1547, requires 397.1547 (+0.1 ppm).

(R)-4-(Benzo[*d*][1,3]dioxol-5-yl)-1,3-diphenyl-4,5-dihydropyrano[2,3-*c*]pyrazol-6(1*H*)-one **56**

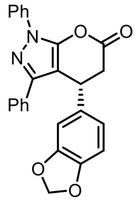

56

Following general procedure **8**, 2,5-diphenyl-2,4-dihydro-3*H*-pyrazol-3-one **43** (56.7 mg, 0.24 mmol), (*E*)-3,4-methylenedioxycinnamic pivalic anhydride **S29** (55.3 mg, 0.20 mmol), (2*S*,3*R*)-HyperSe **12** (7.1 mg, 0.02 mmol), CPME (2.0 mL) and Na_2CO_3 (21.2 mg, 0.20 mmol) for 16 h gave, after purification by Biotage® Selekt™ [SNAP KP-Sil 10 g, 36 mL·min^{-1}, Petrol : EtOAc (90:10 to 88:12 200 mL)], the title compound **56** (24.2 mg, 86 % (NMR), 59% (isolated)) as a white solid. **mp** 165–168 °C (*dec.*); $[\alpha]_D^{20}$–31 (*c* 0.3, $CHCl_3$); **Chiral HPLC analysis:** Chiralpak IC (99:1 hexane : IPA, flow rate 1.0 mL·min^{-1}, 211 nm, 30 °C) t_R(4*S*): 37.9 min, t_R(4*R*): 46.5 min, 1:99 er; **IR** ν_{max} (film) 1794, 1597, 1236; **^{1}H NMR** (500 MHz, $CDCl_3$): δ 7.91–7.89 (m, 2H), 7.61–7.59 (m, 2H), 7.53–7.49 (m, 2H), 7.37–7.30 (m, 4H), 6.78–6.76 (app m, 1H), 6.70–6.68 (m, 2H), 5.97 (d, 1H, J = 1.4 Hz), 5.95 (d, 1H, J = 1.4 Hz), 4.45 (dd, 1H, J = 7.3, 2.1 Hz), 3.21 (dd, 1H, J = 15.6, 7.3 Hz), 3.02 (dd, 1H, J = 15.6, 2.2 Hz);

$^{13}C\{^1H\}$ **NMR** (126 MHz, $CDCl_3$): δ 165.3, 148.6, 148.2, 147.6, 147.3, 137.8, 135.0, 132.5, 129.4, 128.8, 128.5, 127.1, 126.9, 121.4, 120.2, 109.0, 107.3, 101.4, 98.7, 39.1, 34.9; **HRMS** (ESI^+) $C_{25}H_{19}N_2O_4$ $[M+H]^+$ found 411.1335, requires 411.1339 (–1.0 ppm).

(*R*)-1,3-Diphenyl-4-(*o*-tolyl)-4,5-dihydropyrano[2,3-*c*]pyrazol-6(1*H*)-one **57**

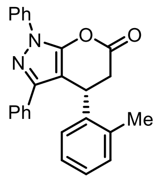

57

Following general procedure **8**, 2,5-diphenyl-2,4-dihydro-3*H*-pyrazol-3-one **43** (56.7 mg, 0.24 mmol), (*E*)-2-methylcinnamic pivalic anhydride **S30** (49.2 mg, 0.20 mmol), (2*S*,3*R*)-HyperSe **12** (7.1 mg, 0.02 mmol), CPME (2.0 mL) and Na_2CO_3 (21.2 mg, 0.20 mmol) for 16 h gave, after purification by flash column chromatography (90:10 Petrol : EtOAc, R_f 0.36), the title compound **57** (29.7 mg, 91% (NMR); 38% (isolated)) as a yellow solid. **mp** 189−191 °C; $[\alpha]_D^{20}$ –73 (*c* 1.5, $CHCl_3$); **Chiral HPLC analysis:** Chiralpak IA (97:3 hexane : IPA, flow rate 1.0 mL·min^{-1}, 220 nm, 30 °C) t_R(4*R*): 11.3 min, t_R(4*S*): 14.1 min, 95:5 er; **IR** ν_{max} (film) 1794, 1599, 1088; ^{1}**H NMR** (500 MHz, $CDCl_3$): δ 7.93 (app d, 2H, J = 7.7 Hz), 7.52 (app t, 2H, J = 8.0 Hz), 7.47–7.45 (m, 2H), 7.36 (app t, 1H, J = 7.4 Hz), 7.31–7.27 (m, 4H), 7.19 (t, 1H, J = 7.9 Hz), 7.13 (t, 1H, J = 7.4 Hz), 7.03 (d, 1H, J = 7.6 Hz), 4.70 (dd, 1H, J = 7.6, 2.0 Hz), 3.20 (dd, 1H, J = 15.6, 7.7 Hz), 2.94 (dd, 1H, J = 15.4, 2.1 Hz), 2.48 (s, 3H); $^{13}C\{^1H\}$ **NMR** (126 MHz, $CDCl_3$): δ 165.4, 148.7, 147.6, 138.9, 137.9, 134.5, 132.6, 131.5, 129.5, 128.8, 128.5, 127.9, 127.3, 127.1, 126.7, 126.6, 121.3, 98.8, 37.3, 31.3, 19.5; **HRMS** (ESI^+) $C_{25}H_{21}N_2O_2$ $[M+H]^+$ found 381.1591, requires 381.1598 (–1.7 ppm).

(*R*)-4-(Naphthalen-1-yl)-1,3-diphenyl-4,5-dihydropyrano[2,3-*c*]pyrazol-6(1*H*)-one **58**

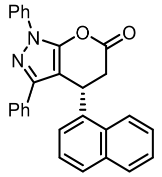

58

Following general procedure **8**, 2,5-diphenyl-2,4-dihydro-3*H*-pyrazol-3-one **43** (56.7 mg, 0.24 mmol), (*E*)-3-(naphth-1-yl)acrylic pivalic anhydride **S31** (56.5 mg, 0.20 mmol), (2*S*,3*R*)-HyperSe **12** (7.1 mg, 0.02 mmol), CPME (2.0 mL) and Na_2CO_3 (21.2 mg, 0.20 mmol) for 16 h gave, after purification by Biotage® Selekt™ [SNAP KP-Sil 10 g, 36 mL.min^{-1}, Petrol : EtOAc (100:0 to 79:21 200 mL)], the title compound **58** (47.6 mg, 54% (NMR); 57% (isolated)) as a white solid. **mp** 192−193 °C; $[\alpha]_D^{20}$–57 (*c* 0.4, $CHCl_3$); **Chiral HPLC analysis:** Chiralpak IA (90:10 hexane : IPA, flow rate 1.0 mL·min^{-1}, 270

nm, 30 °C) $t_R(4R)$: 8.8 min, $t_R(4S)$: 15.3 min, 97:3 er; **IR** ν_{max} (film) 1794, 1599, 1105; **^{1}H NMR** (500 MHz, $CDCl_3$): δ 8.10 (d, 1H, J = 8.3 Hz), 8.01–7.93 (m, 3H), 7.83 (d, 1H, J = 8.2 Hz), 7.67–7.64 (m, 1H), 7.62–7.59 (m, 1H), 7.54 (app t, 2H, J = 8.0 Hz), 7.51–5.49 (m, 2H), 7.40–7.36 (m, 2H), 7.27–7.26 (app m, 1H), 7.23–7.18 (m, 3H), 5.33 (dd, 1H, J = 7.5, 1.9 Hz), 3.36 (dd, 1H, J = 15.6, 7.5 Hz), 3.26 (dd, 1H, J = 15.6, 1.8 Hz); **^{13}C{^{1}H} NMR** (126 MHz, $CDCl_3$): δ 165.2, 148.9, 147.6, 137.9, 135.9, 134.7, 132.5, 130.3, 129.8, 129.5, 128.90, 128.85, 128.5, 127.2, 127.1, 126.7, 126.2, 125.9, 124.9, 122.2, 121.3, 98.1, 37.6, 30.9; **HRMS** (ESI^+) $C_{28}H_{21}O_2N_2$ $[M+H]^+$ found 417.1586, requires 417.1598 (–2.9 ppm).

(*R*)-4-(4-Bromophenyl)-1,3-diphenyl-4,5-dihydropyrano[2,3-*c*]pyrazol-6(1*H*)-one **59**

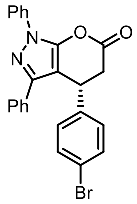

59

Following general procedure **8**, 2,5-diphenyl-2,4-dihydro-3*H*-pyrazol-3-one **43** (56.7 mg, 0.24 mmol), (*E*)-4-bromocinnamic pivalic anhydride **S14** (62.2 mg, 0.20 mmol), (2*S*,3*R*)-HyperSe **12** (7.1 mg, 0.02 mmol), CPME (2.0 mL) and Na_2CO_3 (21.2 mg, 0.20 mmol) for 16 h gave, after purification by flash column chromatography (90:10 Petrol : EtOAc, R_f 0.25), the title compound **59** (25.8 mg, 77% (NMR); 29% (isolated)) as a yellow solid. **mp** 162−164°C; $[\alpha]_D^{20}$ −14 (*c* 0.7, $CHCl_3$); **Chiral HPLC analysis:** Chiralpak IA (97:3 hexane : IPA, flow rate 1.0 mL·min^{-1}, 220 nm, 30 °C) $t_R(4S)$: 14.4 min, $t_R(4R)$: 21.3 min, 2:98 er; **IR** ν_{max} (film) 1788, 1601, 1096; **^{1}H NMR** (300 MHz, $CDCl_3$): δ 7.91–7.88 (m, 2H), 7.71–7.64 (m, 1H), 7.56–7.46 (m, 6H, m), 7.38–7.31 (m, 3H), 7.13–7.10 (m, 2H), 4.50 (dd, 1H, J = 7.3, 2.3 Hz), 3.25 (dd, 1H, J = 15.7, 7.4 Hz), 3.03 (dd, 1H, J = 15.7, 2.3 Hz); **^{13}C{^{1}H} NMR** (126 MHz, $CDCl_3$): δ 165.0, 148.2, 147.6, 140.2, 137.7, 132.7, 132.4, 132.2, 129.5, 128.83, 128.77, 127.3, 126.8, 121.9, 121.4, 98.0, 38.6, 34.7; **HRMS** (ESI^+) $C_{24}H_{18}BrN_2O_2$ $[M+H]^+$ found 445.0536, requires 445.0546 (–2.3 ppm).

(*R*)-1,3-Diphenyl-4-(4-(trifluoromethyl)phenyl)-4,5-dihydropyrano[2,3-*c*]pyrazol-6(1*H*)-one **60**

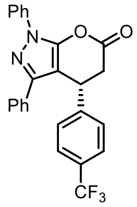

60

Following general procedure **8**, 2,5-diphenyl-2,4-dihydro-3*H*-pyrazol-3-one **43** (56.7 mg, 0.24 mmol), (E)-4-(trifluoromethyl)cinnamic pivalic anhydride **S32** (60.1 mg, 0.20 mmol), (2*S*,3*R*)-HyperSe **12** (7.1

mg, 0.02 mmol), CPME (2.0 mL) and Na_2CO_3 (21.2 mg, 0.20 mmol) for 16 h gave, after purification by flash column chromatography (90:10 Petrol : EtOAc, R_f 0.23), the title compound **60** (19.0 mg, 56% (NMR); 22% (isolated)) as a white solid. **mp** 172−174 °C; $[\alpha]_D^{20}$ –35 (*c* 0.2, $CHCl_3$); **Chiral HPLC analysis:** Chiralpak IA (97:3 hexane : IPA, flow rate 1.0 mL·min^{-1}, 254 nm, 30 °C) t_R(4*S*): 12.4 min, t_R(4*R*): 17.9 min, 1:99 er; **IR** ν_{max} (film) 1792, 1599, 1098; **^{1}H NMR** (300 MHz, $CDCl_3$): δ 7.92–7.88 (m, 2H), 7.62 (d, 2H, J = 8.0 Hz), 7.55–7.49 (m, 4H), 7.39–7.31 (m, 6H), 4.61 (dd, 1H, J =7.3, 2.3 Hz), 3.30 (dd, 1H, J = 15.8, 7.4 Hz), 3.06 (dd, 1H, J = 15.8, 2.5 Hz); **^{13}C{^{1}H} NMR** (126 MHz, $CDCl_3$): δ 164.8, 148.2, 147.6, 145.1, 137.7, 130.4 (q, $^2J_{CF}$ = 30.1 Hz), 130.2, 129.5, 128.9, 128.7, 127.5, 127.3, 126.8, 126.6 (q, $^3J_{CF}$ = 10.6 Hz), 124.0 (q, $^1J_{CF}$ = 271.7 Hz), 121.4, 97.7, 38.5, 35.0; **^{19}F{^{1}H} NMR** (470 MHz, $CDCl_3$): δ –62.62 (s, 3F); **HRMS** (ESI$^+$) $C_{25}H_{18}F_3O_2N_2$ $[M+H]^+$ found 435.1305, requires 435.1315 (–2.3 ppm).

5.7. 1,2-Addition Product Synthesis

(*E*)-4,4,4-trifluoro-N-(3-methyl-1-phenyl-1H-pyrazol-5-yl)-N-(methylsulfonyl)but-2-enamide **21**

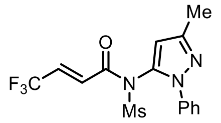

21

Following general procedure **9**, (*E*)-4,4,4-trifluorobut-2-enoic acid **S17** (83.9 mg, 0.6 mmol), DMF (2 drops), CH_2Cl_2 (1.9 mL), oxalyl chloride (54.5 μL, 0.63 mmol) for 1 h, followed by *N*-(3-methyl-1-phenyl-1*H*-pyrazol-5-yl)methanesulfonamide **15** (150.0 mg, 0.6 mmol) and $^{i}Pr_2NEt$ (0.21 mL, 1.2 mmol) and CH_2Cl_2 (1.9 mL) for 16 h gave, after purification by Biotage® Selekt^{TM} [Sfär HC 50 g, 150 mL.min^{-1}, Petrol : EtOAc (100:0 to 50:50 20 CV, 50:50 to 20:80 10 CV)], the title compound **21** (77.8 mg, 35%) as a milky white oil. **IR** ν_{max} (film) 2936, 1709, 1366, 1304, 1136; **^{1}H NMR** (400 MHz, $CDCl_3$): δ 7.51–7.44 (m, 3H), 7.37–7.34 (m, 2H), 6.88 (dq, 1H, J = 15.3, 6.5 Hz), 6.47 (dq, 1H, J = 15.3, 1.9 Hz), 6.40 (s, 1H), 3.08 (s, 3H), 2.41 (s, 3H); **^{13}C{^{1}H} NMR** (126 MHz, $CDCl_3$): δ 162.9, 150.4, 137.6, 134.2 (q, $^2J_{CF}$ = 36.0 Hz), 131.7, 129.7, 129.5, 126.4 (q, $^3J_{CF}$ = 5.8 Hz), 125.6, 121.6 q, $^1J_{CF}$ = 270.7), 108.5, 42.1, 14.3; **^{19}F{^{1}H} NMR** (377 MHz, $CDCl_3$): δ –65.42 (s, 3F); **HRMS** (ESI)$^+$ $C_{15}H_{14}F_3N_3O_3SNa$ [M+Na]$^+$ found 396.0598, requires 396.0600 (–0.5 ppm).

(E)-4,4,4-trifluoro-N-(1-(4-methoxyphenyl)-3-methyl-1H-pyrazol-5-yl)-N-(methylsulfonyl)but-2-enamide **25**

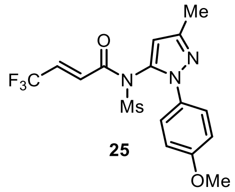

25

Following general procedure **9**, (*E*)-4,4,4-trifluorobut-2-enoic acid **S17** (74.23 mg, 0.53 mmol), DMF (2 drops), CH_2Cl_2 (1.7 mL), oxalyl chloride (48 μL, 0.56 mmol) for 1 h followed by *N*-(1-(4-methoxyphenyl)-3-methyl-1*H*-pyrazol-5-yl)methanesulfonamide **24** (150 mg, 0.53 mmol), $^{i}Pr_2NEt$ (0.18 mL, 1.1 mmol, 2.0 equiv.) and CH_2Cl_2 (1.7 mL) for 16 h gave, after purification by Biotage® Selekt^{TM} [Sfär HC 50 g, 150 mL.min^{-1}, Petrol : EtOAc (95:5 to 50:50 20 CV, 50:50 to 20:80 10 CV)] give the title compound **25** (24.4 mg, 11%) as a pale yellow oil. **IR** ν_{max} (film) 2932, 1707, 1364, 1302, 1130; **^{1}H NMR** (400 MHz, $CDCl_3$): δ 7.28–7.24 (app m, 2H), 6.98–6.94 (app m, 2H), 6.88 (dq, 1H, J = 15.3, 6.5 Hz), 6.44 (dq, 1H, J = 15.3, 1.9 Hz), 6.38 (s, 1H), 3.84 (s, 3H), 3.11 (s, 3H), 2.39 (s, 3H);

^{13}C{^{1}H} NMR (126 MHz, $CDCl_3$): δ 162.9, 160.3, 150.0, 134.1 (q, $^2J_{CF}$ = 36.0 Hz), 131.9, 130.3, 127.3, 126.5 (q, $^3J_{CF}$ = 6.0 Hz), 121.7 (q, $^1J_{CF}$ = 270.6 Hz), 114.8, 108.8, 55.7, 42.2, 14.3; **^{19}F{^{1}H} NMR** (377 MHz, $CDCl_3$): δ −65.39; **HRMS** (ESI$^+$) $C_{16}H_{16}F_3N_3O_4Na$ $[M+Na]^+$ found 426.0705, required 426.0706 (−0.2 ppm).

1,3-diphenyl-1H-pyrazol-5-yl cinnamate **45**

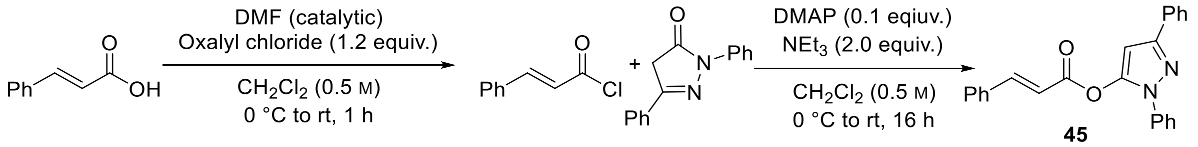

Following literature [47], under N_2, cinnamic acid (0.31 g, 2.12 mmol, 1.0 equiv.), DMF (2 drops) and CH_2Cl_2 (4.2 mL) were combined and set to 0 °C. Oxalyl chloride (0.21 mL, 2.54 mmol, 1.2 equiv.) was added dropwise and the solution was stirred until gas evolution stopped. The mixture was concentrated under reduced pressure and then used directly in the next step. 2,5-diphenyl-2,4-dihydro-3*H*-pyrazol-3-one **43** (0.5 g, 2.12 mmol, 1.0 equiv.) and DMAP (25.9 mg, 0.21 mmol, 0.1 equiv.) were combined and backfilled with N_2. CH_2Cl_2 (4.2 mL) followed by Et_3N (0.6 mL) were added and the reaction was set to 0 °C. The acyl chloride prepared in the previous step in CH_2Cl_2 (2.1 mL) was added dropwise and the mixture was stirred at rt overnight. The mixture was then diluted with CH_2Cl_2 and the organic layer was washed sequentially with 1M HCl and brine, dried (Na_2SO_4) and concentrated under reduced pressure to afford the crude product which was purified via Biotage® Selekt™ [Sfär HC 50 g, 150 mL.min^{-1}, Petrol : EtOAc (100:0 to 70:30 15 CV, 70:30 to 50:50 10 CV)] to afford the title compound **45** (0.23 g, 28%) as a yellow oil. **IR** ν_{max} (film) 3963, 1746, 1632, 1503, 1113; **^{1}H NMR** (400 MHz, $CDCl_3$): δ 7.92–7.85 (m, 3H), 7.72–7.69 (m, 2H), 7.58–7.56 (m, 2H), 7.52–7.48 (m, 2H), 7.46–7.41 (m, 5H), 7.39–7.30 (m, 2H), 6.77 (s, 1H), 6.55 (d, 1H, J = 16.0 Hz); **^{13}C{^{1}H} NMR** (126 MHz, $CDCl_3$): δ 162.0, 151.2, 148.8, 145.3, 138.4, 133.8, 133.3, 131.5, 129.3, 129.2, 128.73, 128.69, 128.3, 127.6, 125.8, 123.5, 115.5, 93.5; **HRMS** (ESI$^+$) $C_{24}H_{19}N_2O_2$ $[M+H]^+$ found 367.1441, requires 367.1441 (± 0.1 ppm).

5.8. Mechanistic investigation of 1,2-addition substrate

Following general procedure 7, anhydride **14**, aminopyrazole **24**, with catalyst **12** or **17** in CPME with 1,3,5-trimethoxybenzene as the internal standard were stirred in a round bottom flask. 100 μL aliquots of the reaction mixture were taken, concentrated, diluted with $CDCl_3$ and ^1H and ^{19}F NMR were taken. From this data the relative concentrations of **14**, **24**, **25** and **27** (in mM) were determined.

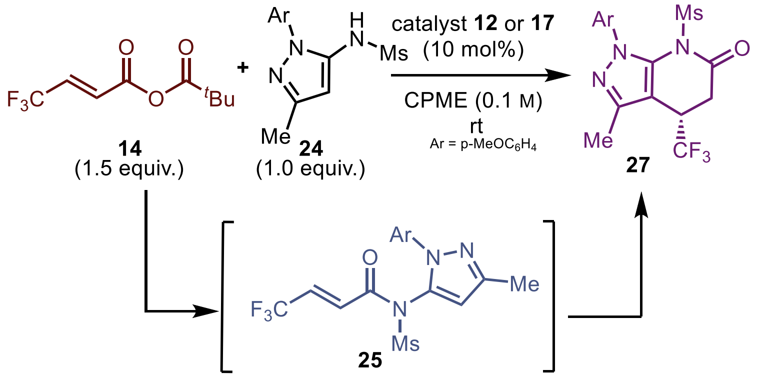

*Figure 5.1: NMR reaction profiles comparing isoselenourea catalyst HyperSe **12** and isothiourea catalyst HyperBTM **17** in the reaction between anhydride **14** and aminopyrazole **24**.*

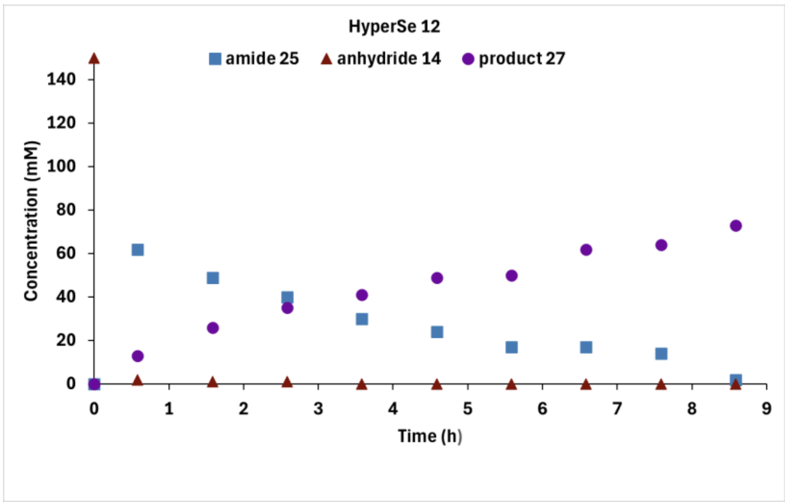

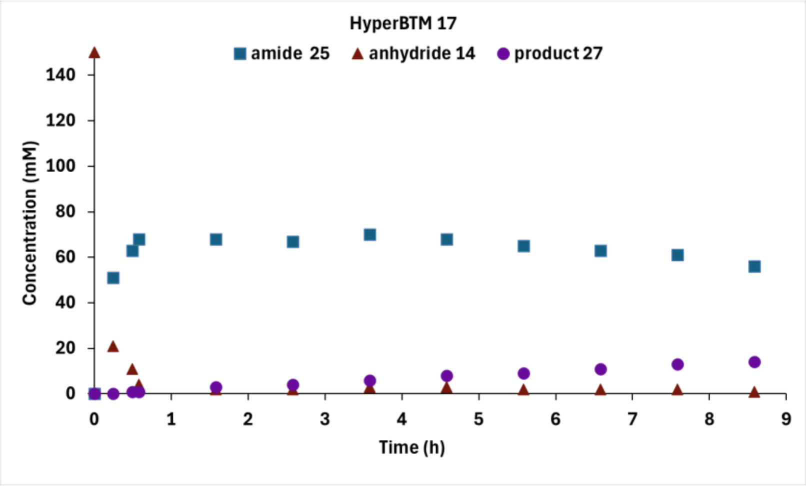

Following general procedure 7, anhydride **42**, pyrazolone **43**, with catalyst **12** in CPME with 1,3,5-trimethoxybenzene as the internal standard were stirred in a round bottom flask. 100 µL aliquots of the reaction mixture were taken, concentrated, diluted with $CDCl_3$ and ^{1}H were taken. From this data the relative concentrations of **42**, **43**, **45** and **44** (in mM) were determined.

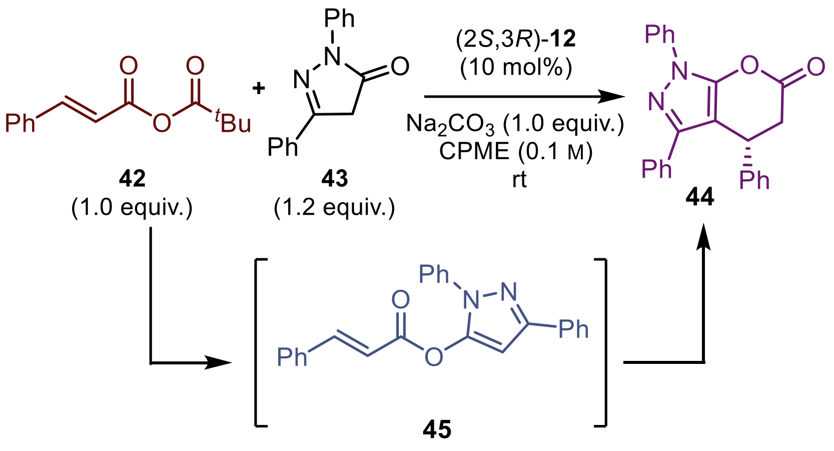

*Figure 5.2:NMR reaction profile of HyperSe **12** with anhydride **42** and pyrazolone **43**.*

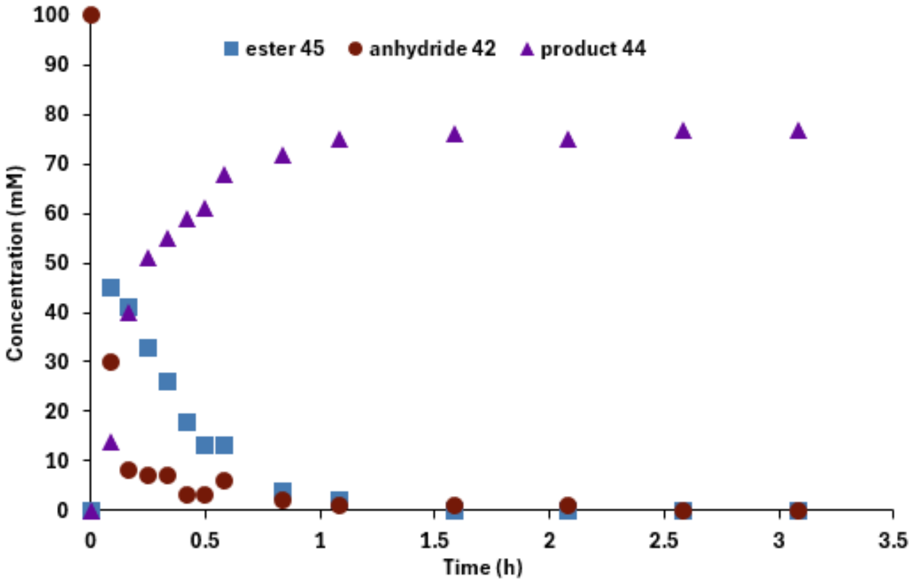

5.9. Cross-over Experiment

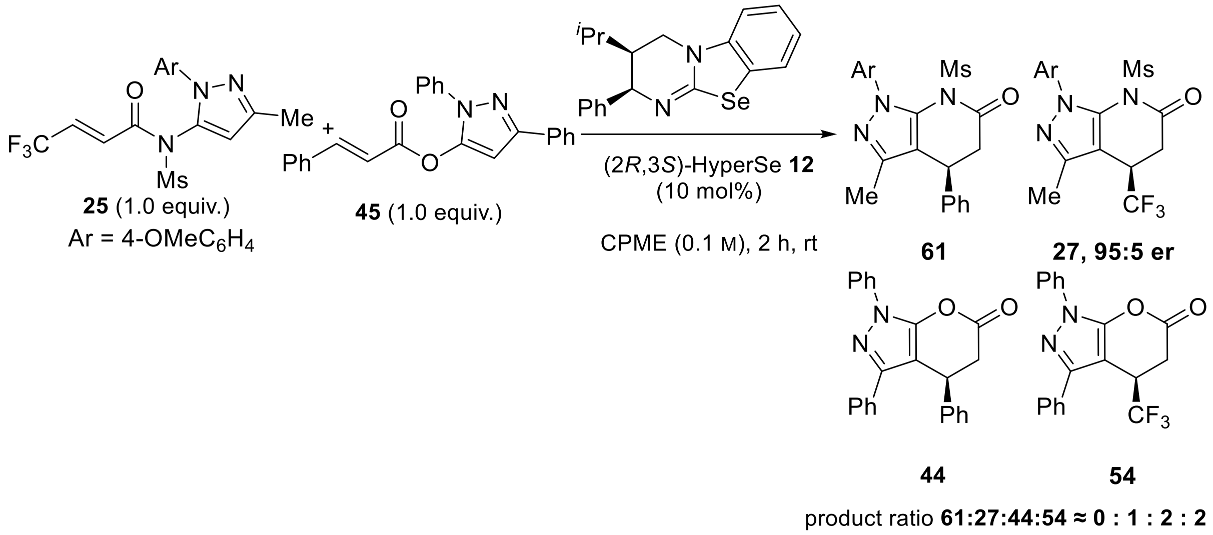

Following general procedure **3**, (*E*)-4,4,4-trifluoro-*N*-(1-(4-methoxyphenyl)-3-methyl-1*H*-pyrazol-5-yl)-*N*-(methylsulfonyl)but-2-enamide **25** (22.25 mg, 0.06 mmol), 1,3-diphenyl-1*H*-pyrazol-5-yl cinnamate **45** (20.15 mg, 0.06 mmol) and (2*R*,3*S*)-HyperSe **12** (1.96 mg, 0.006 mmol) in CPME (0.6 mL) for 2 h gave the crude product mixture which was analysed without purification *via* ^{1}H NMR using 1,3,5-trimethoxybenzene as the internal standard.

5.10. α,β-disubstituted anhydride substitution

(*E*)-2,3-diphenylacrylic pivalic anhydride **S56**

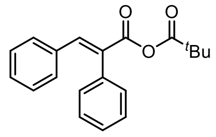

S56

Following general procedure **2**, (*E*)-2,3-diphenylacrylic acid (1.0 g, 4.5 mmol), THF (15 mL), Et_3N (0.81 mL, 5.8 mmol) and pivaloyl chloride (0.67 mL, 5.3 mmol), for 1 h gave the title compound **S56** (1.5 g, *quant.*) as a colourless oil. **IR** ν_{max} (film) 2976, 1794, 1721, 1204, 1047, 1013, 999; **^{1}H NMR** (400 MHz, $CDCl_3$): δ 7.91 (s, 1H), 7.41−7.36 (m, 3H), 7.27−7.23 (m, 3H), 7.18 (t, 2H, J = 7.6 Hz), 7.08 (d, 2H, J = 7.5 Hz), 1.12 (s, 9H); **^{13}C{^{1}H} NMR** (126 MHz, $CDCl_3$): δ 174.1, 164.1, 144.1, 135.1, 134.1, 131.9, 131.2, 130.1, 129.8, 129.0, 128.5, 128.4, 39.9, 26.5; **HRMS** (ESI$^+$) $C_{20}H_{20}O_3Na$ [M+Na]$^+$ found 331.1305, requires 331.1305 (+0.03 ppm).

(*E*)-*N*-(3-methyl-1-phenyl-1*H*-pyrazol-5-yl)-*N*-(methylsulfonyl)-2,3-diphenylacrylamide **S57**

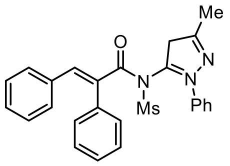

S57

Following general procedure **7**, (*E*)-2,3-diphenylacrylic pivalic anhydride **S56** (92.5 mg, 0.30 mmol), (2*S*,3*R*)-HyperSe **12** (7.1 mg, 0.02 mmol), CPME (2 mL) and N-(3-methyl-1-phenyl-1H-pyrazol-5-yl)methanesulfonamide **15** (50.3 mg, 0.20 mmol) for 16 h gave, after purification by Biotage® Selekt™ [Sfär HC 25 g, 80 mL.min^{-1}, Petrol : EtOAc (100:0 to 90:10 10 CV, 90:10 to 75:25 10 CV, 75:25 to 50:50 10 CV)] the title compound **S57** (43.7 mg, 48%) as a white solid. **mp** 62–65 ºC; **IR** ν_{max} (film) 3049, 2928, 1690, 1499, 1360, 1171, 1138, 962; **^{1}H NMR** (400 MHz, $CDCl_3$): δ 7.49–7.42 (m, 5H), 7.28 (t, 1H, J = 7.4 Hz), 7.22 (t, 2H, J = 7.5 Hz), 7.18 (d, 1H, J = 7.4 Hz), 7.11 (t, 2H, J = 7.6 Hz), 6.89 (d, 2H, J = 8.4 Hz), 6.83 (d, 2H, J = 7.5 Hz), 6.57 (s, 1H), 3.38 (s, 3H), 2.10 (s, 3H); **^{13}C{^{1}H} NMR** (126 MHz, $CDCl_3$): δ 170.8, 149.2, 138.5, 138.0, 135.6, 133.9, 133.6, 131.2, 130.2, 129.5, 129.2, 129.0, 128.69, 128.67, 128.28, 128.26, 124.4, 108.8, 42.2, 14.0; **HRMS** (ESI$^+$) $C_{26}H_{23}N_3O_3SNa$ $[M+Na]^+$ found 480.1352, requires 480.1352 (±0.0 ppm).

Note that attempts at forming the corresponding lactone (or 1,2-addition product) from **43** and **S56** under the optimised pyrazolone catalysis conditions were unsuccessful with unreacted starting materials remaining.

6. Computational Supporting Information

6.1. Computational Details

Geometry optimisations were performed with the *meta*-hybrid M06-2X functional [37] using the double-ζ, def2-SVP basis set from the redefinition of the Ahlrichs family of basis sets. [38,39,65,66] Implicit solvation was considered through the use of the IEFPCM model employing the parameters of THF (ε = 7.4257). [40] THF was used to model solvation instead of CPME which was used experimentally. Both are ethereal solvents with similarly low dielectric constants and PCM parameters were not available for CPME. An ultrafine integration grid (99 radial shells with 590 angular points per shell) was used for all calculations and all species were formally treated as closed-shell systems with restricted Kohn-Sham DFT used throughout. The nature of minima and transition states located were verified by the computation of harmonic frequencies at the same level of theory. Single-point energies (E_{sp}) were also evaluated using the M06-2X functional [37] with a larger, triple-ζ, def2-TZVP basis. Implicit solvation was also included at this level of theory using the same ultrafine integration grid (99,590). Additional empirical dispersion corrections were not included as the functional implicitly accounts for dispersion due to the nature of its construction. Thermochemistry was evaluated at 1 atm and 298.15 K using thermodynamic calculations at the level of geometry optimisation (thermal corrections to enthalpy, $\delta H_{298.15}$, and entropies $S_{298.15}$) in combination with energetics obtained from single-point calculations. Gibbs free energy was calculated at 298 K using Equation **1**, with additional Martin Hay Pratt empirical entropic corrections included (S_{MHP} = 3.38 kcal/mol per particle, evaluated at 302 atm to mimic bulk THF). [67] All computations were performed using the Gaussian16, C.01 programme [41] with visualisation of structures using PyMol. [68] Conformational flexibility of the key enantiodetermining transition state was evaluated by modelling each staggered conformation of the forming C-C bond (Figure 6.1). The reaction profile was then constructed by following the reactivity in each direction using the lowest energy conformations from the key transition state. The approach should allow for an accurate measure of the enantioselectivity and interactions in the key Michael addition transition state alongside a balance of computation cost in generating the remaining reaction profile. This and similar levels of DFT have previously been used successfully to rationalise reactivities and selectivities of organocatalytic reactions with isothioureas. [69.70]

$$G_{298.15} = E_{sp} + \delta H_{298.15} - \mathrm{T}.S_{298.15} + S_{MHP} \qquad (1)$$

6.2. Interaction and Reorganisation Energies

Following the activation-strain model, [44] both interaction and reorganisation energies were calculated from the respective geometry by fragmenting into the pyrazolone nucleophile and *N*-acylated isothiourea electrophile. Single-point energies of the TS and each fragment (in the geometry of the TS) were computed in the gas-phase and the interaction energy is given by Equation **2**. Reorganisation energies are calculated by the gas-phase single-point of the relaxed geometry of each fragment (*eg.* minima of each reactant) and this is given by Equation **3**.

$$\Delta E_{interaction} = E_{complex} - \Sigma E_{rigid\_fragments} \quad (2)$$

$$\Delta E_{reorg} = \Sigma(E_{rigid\_fragment} - E_{relaxed\_fragment}) \quad (3)$$

6.3. Staggered Conformations of TS-MA

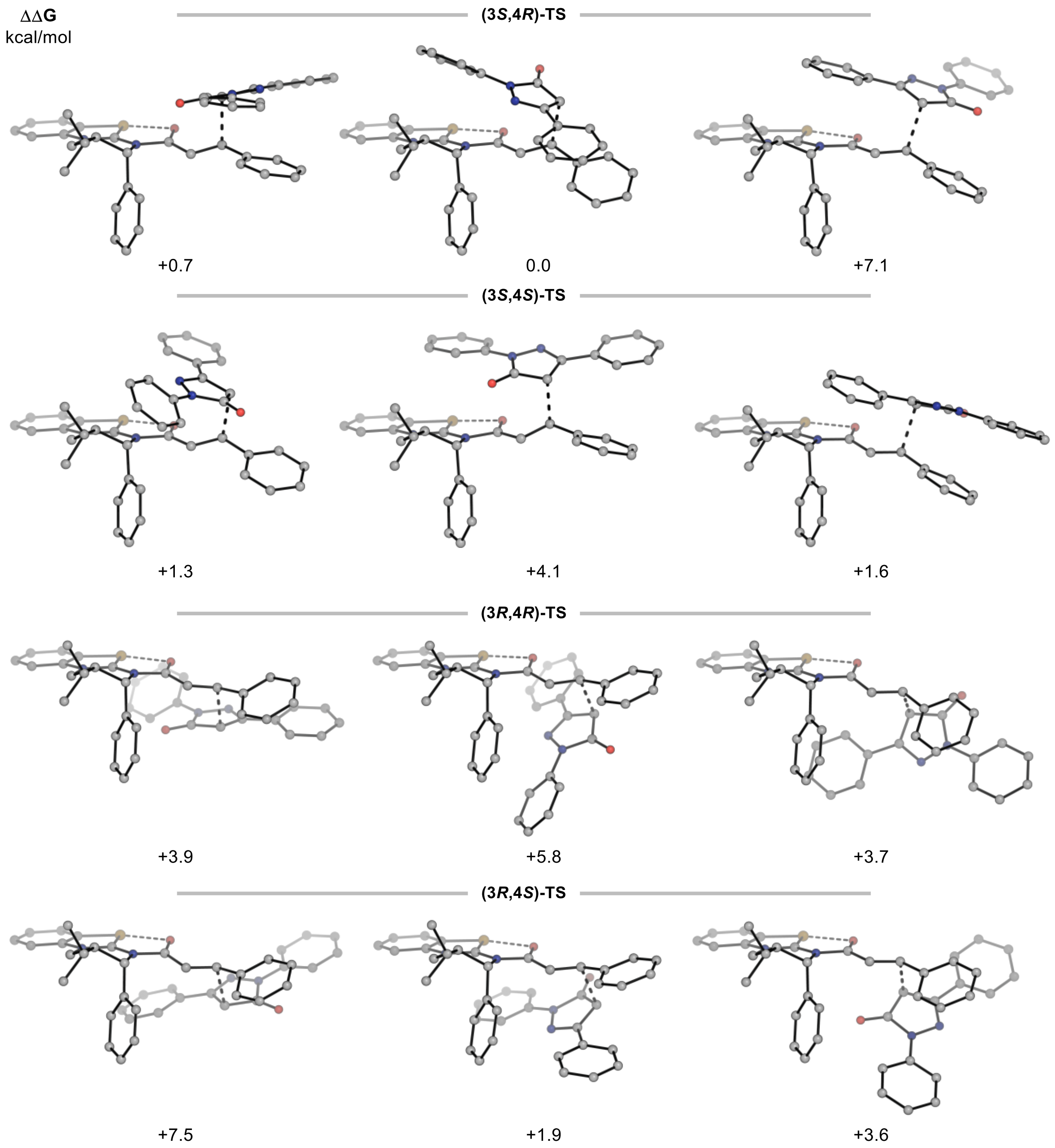

Figure 6.1. Conformational analysis of Michael addition transition state. Free energies (ΔG) in kcal/mol.

6.4. Formation of 1,2-Addition Product

The rapid formation of a 1,2-addition product has been observed with NMR monitoring. This is supported computationally by the barrier for the formation of this product ($\Delta^{\ddagger}G = 9.7$ kcal/mol), which is slightly lower than the computed barrier for Michael addition ($\Delta\Delta G = 0.7$ kcal/mol). Due to the driving force for the reaction ($\Delta_r G = -5.4$ kcal/mol), the reverse reaction has a higher barrier,

$\Delta^{\ddagger}G$ = 15.1 kcal/mol, reflecting the slower conversion from the 1,2-product back to the acyl ammonium intermediate.

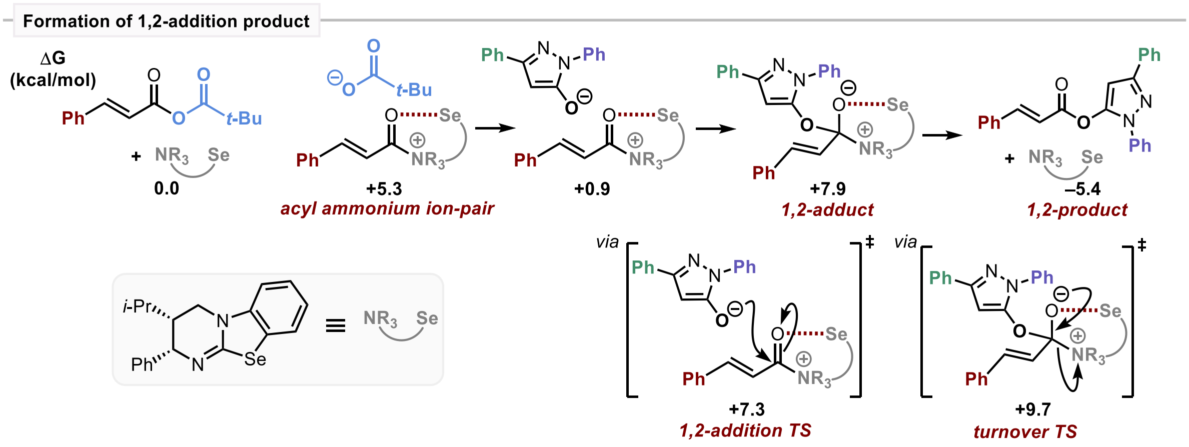

Figure 6.2. Proposed mechanism for the formation of the direct 1,2-addition product. Free energies (ΔG) in kcal/mol.

7. Computational Data

Raw data and cartesian coordinates obtained from geometry optimisation and frequency calculations with subsequent single-point energy calculations.

----
alcohol
Frequencies, energies and thermodynamic properties:
Lowest Vibrational Mode (1/cm) = 49.8334
2nd Lowest Vibrational Mode (1/cm) = 203.4457
E(RM062X) (a.u.) = -346.615809840
Thermal correction to Enthalpy (a.u.) = 0.156460
Thermal correction to Gibbs Free Energy (a.u.) = 0.115224
Total Entropy (cal/Kmol) = 86.790
Esp(RM062X) (a.u.) = -347.018522702
Optimised cartesian coordinates (Angstrom):
O -1.603299 0.979686 0.000064
C -0.938906 -0.183054 0.000034
O -1.508353 -1.243706 0.000043
C 0.571219 0.010863 0.000016
C 0.961414 0.804177 1.256228
C 1.243646 -1.359500 0.001184
C 0.961446 0.802051 -1.257533
H 0.482423 1.792300 1.263014
H 0.668968 0.266419 2.170160
H 2.052133 0.943660 1.274702
H 0.955177 -1.938941 -0.886617
H 2.335993 -1.235945 0.000952
H 0.955418 -1.937341 0.890119
H 2.052098 0.942092 -1.275973
H 0.669543 0.262474 -2.170568
H 0.481994 1.789939 -1.266308
H -2.553445 0.780285 0.000092
----
anhydride
Frequencies, energies and thermodynamic properties:
Lowest Vibrational Mode (1/cm) = 21.4201
2nd Lowest Vibrational Mode (1/cm) = 23.8713
E(RM062X) (a.u.) = -767.921246827
Thermal correction to Enthalpy (a.u.) = 0.290039
Thermal correction to Gibbs Free Energy (a.u.) = 0.225134
Total Entropy (cal/Kmol) = 136.604
Esp(RM062X) (a.u.) = -768.787472503
Optimised cartesian coordinates (Angstrom):
C -4.749827 1.657463 0.233556
C -3.447950 1.173286 0.180926
C -3.205079 -0.193787 -0.038366
C -4.299461 -1.056897 -0.201159
C -5.604367 -0.570985 -0.148753
C -5.831703 0.787212 0.068640
H -4.925777 2.720260 0.404693
H -2.613392 1.863478 0.312395
H -4.118641 -2.120291 -0.370672
H -6.444715 -1.254251 -0.277609
H -6.851915 1.171426 0.110665
C -1.852459 -0.755259 -0.103717
C -0.693004 -0.087780 0.017402
H -1.785237 -1.835732 -0.269239
H -0.629350 0.988160 0.180786
C 0.579036 -0.828900 -0.068920
O 1.644322 0.039555 0.012782
O 0.713810 -2.005929 -0.232420
C 2.892813 -0.377184 0.419818
O 3.047144 -1.316697 1.137329
C 3.977836 0.534477 -0.128048
C 5.325376 0.065482 0.415915
C 3.949403 0.439869 -1.661469
C 3.690463 1.977850 0.312827
H 5.342407 0.108348 1.513697
H 5.533613 -0.969770 0.112865
H 6.124157 0.712631 0.027345
H 2.985860 0.783970 -2.060901
H 4.745253 1.074590 -2.076970
H 4.122023 -0.592744 -1.998381
H 4.482893 2.635265 -0.072942
H 2.726274 2.327750 -0.078470
H 3.677891 2.062389 1.409292
----
back_face_R_1-TS1
Frequencies, energies and thermodynamic properties:
Lowest Vibrational Mode (1/cm) = -354.5536
2nd Lowest Vibrational Mode (1/cm) = 15.7736
E(RM062X) (a.u.) = -4429.88727281
Thermal correction to Enthalpy (a.u.) = 0.760821
Thermal correction to Gibbs Free Energy (a.u.) = 0.642829
Total Entropy (cal/Kmol) = 248.334
Esp(RM062X) (a.u.) = -4432.39798095

Optimised cartesian coordinates (Angstrom):

C	-6.644562	-1.893836	0.125233
C	-6.064310	-3.145192	-0.115854
C	-4.718500	-3.245544	-0.459121
C	-3.962531	-2.077930	-0.564066
C	-4.549682	-0.830775	-0.330289
H	-6.668394	-4.048729	-0.029219
H	-4.259217	-4.218940	-0.640074
C	-2.385456	-0.072433	-0.742431
C	-1.693798	2.271089	-0.597576
C	-3.140672	2.551429	-1.044813
C	-4.096577	1.632143	-0.304614
H	-5.106188	1.715423	-0.727536
N	-3.659246	0.246480	-0.466815
N	-1.411959	0.837739	-0.803240
C	-3.555601	4.038496	-0.956776
C	-4.181108	4.443880	0.381703
C	-4.508655	4.382759	-2.103329
H	-2.638551	4.633164	-1.099023
H	-5.177048	3.989560	0.502589
H	-3.564613	4.159363	1.245508
H	-4.317223	5.534154	0.409054
H	-4.039080	4.208211	-3.081638
H	-5.423511	3.771476	-2.050614
H	-4.811664	5.437943	-2.051272
H	-3.174255	2.262251	-2.108310
H	-1.038564	2.807399	-1.296994
C	-1.378915	2.726816	0.812740
C	-0.842550	4.004118	1.011068
C	-1.663614	1.926822	1.921834
C	-0.635184	4.492904	2.299484
H	-0.584120	4.623385	0.148287
C	-1.458283	2.415651	3.211801
H	-2.009503	0.900266	1.785385
C	-0.953646	3.701503	3.404002
H	-0.219391	5.491468	2.441119
H	-1.680780	1.781025	4.070634
H	-0.791505	4.080465	4.414072
C	1.007386	1.286344	-0.800099
H	0.835740	2.297931	-0.444429
C	2.306622	0.760894	-0.870560
O	0.075833	-0.807985	-1.337775
C	-0.048251	0.373050	-1.000143
H	-4.148159	1.868791	0.769236
C	-5.897347	-0.723697	0.023940
H	-6.356803	0.242198	0.230744
H	-7.697015	-1.829369	0.402723
H	2.394328	-0.187373	-1.407413
C	1.490963	-0.766130	1.265240
O	0.423970	-0.272174	1.618126
C	3.484871	1.654023	-0.999492
C	3.594316	2.847656	-0.269584
C	4.518889	1.303923	-1.875874
C	4.714024	3.663032	-0.410453
H	2.802286	3.128183	0.429689
C	5.634508	2.126832	-2.028148
H	4.449227	0.368775	-2.436080
C	5.738323	3.305990	-1.292411
H	4.790814	4.583286	0.171094
H	6.431802	1.836690	-2.714386
H	6.614710	3.946734	-1.401783
C	2.774776	-0.150036	0.992973
C	3.638664	-1.253474	0.654608
H	3.076818	0.769652	1.486909
N	1.685216	-2.108968	0.899197
N	2.966846	-2.373368	0.531282
C	5.092294	-1.210107	0.415782
C	5.759428	-2.322764	-0.120640
C	5.835629	-0.064873	0.731835
C	7.133284	-2.286079	-0.338891
H	5.179747	-3.214229	-0.362884
C	7.212456	-0.031267	0.516138
H	5.330980	0.816000	1.134319
C	7.866624	-1.139589	-0.020988
H	7.637939	-3.157228	-0.760289
H	7.774733	0.870887	0.763370
H	8.943861	-1.112009	-0.192593
C	0.693675	-3.097562	0.818414
C	0.750055	-4.056253	-0.201131
C	-0.354337	-3.127707	1.748241
C	-0.245952	-5.024358	-0.298007
H	1.575155	-4.018274	-0.911717
C	-1.345965	-4.100699	1.639180
H	-0.389584	-2.377550	2.535667
C	-1.302963	-5.049303	0.616074
H	-0.198242	-5.764518	-1.098693
H	-2.162060	-4.116445	2.364098
H	-2.080154	-5.811315	0.537923
Se	-2.119909	-1.919973	-0.984213

----

back_face_R_2-TS1

Frequencies, energies and thermodynamic properties:

Lowest Vibrational Mode (1/cm) =	-248.3356
2nd Lowest Vibrational Mode (1/cm) =	12.7428
E(RM062X) (a.u.) =	-4429.88372064

Thermal correction to Enthalpy (a.u.) = 0.760911
Thermal correction to Gibbs Free Energy (a.u.) = 0.643903
Total Entropy (cal/Kmol) = 246.265
Esp(RM062X) (a.u.) = -4432.39604700
Optimised cartesian coordinates (Angstrom):
C 6.729249 -0.307533 0.733556
C 6.698950 1.082216 0.900116
C 5.552859 1.807423 0.585158
C 4.438461 1.124099 0.099115
C 4.478356 -0.262121 -0.075753
H 7.577365 1.601780 1.283653
H 5.525140 2.889817 0.719156
C 2.274780 0.058041 -0.760264
C 0.748565 -1.750081 -1.380843
C 2.033407 -2.438484 -1.880284
C 3.156359 -2.231600 -0.879024
H 4.110774 -2.564405 -1.307228
N 3.282390 -0.805393 -0.574833
N 1.049998 -0.328883 -1.120976
C 1.843182 -3.924436 -2.265208
C 2.072886 -4.913309 -1.117681
C 2.747234 -4.278252 -3.448038
H 0.799222 -4.032112 -2.602396
H 3.138316 -4.954622 -0.841965
H 1.491666 -4.668864 -0.217645
H 1.783611 -5.923137 -1.440941
H 2.523924 -3.654357 -4.324910
H 3.808481 -4.135832 -3.189337
H 2.614836 -5.331226 -3.733667
H 2.313761 -1.893971 -2.797189
H 0.041488 -1.754498 -2.220262
C 0.105663 -2.434442 -0.195052
C -0.909701 -3.370081 -0.422862
C 0.567440 -2.230456 1.108129
C -1.411652 -4.136538 0.626537
H -1.300422 -3.509053 -1.434396
C 0.064918 -2.998615 2.158432
H 1.320460 -1.465798 1.315861
C -0.910771 -3.964721 1.917127
H -2.199552 -4.867055 0.436948
H 0.430613 -2.834433 3.172623
H -1.302495 -4.561295 2.741414
C -1.319198 0.251730 -1.403328
H -1.588105 -0.786282 -1.233057
C -2.290199 1.226128 -1.653597
O 0.417621 1.850455 -1.334744
C 0.019040 0.687321 -1.287954
H 2.984232 -2.783338 0.057394
C 5.622708 -0.996546 0.245908
H 5.654122 -2.079441 0.133054
H 7.630143 -0.864520 0.992050
H -1.899867 2.198821 -1.960755
C -3.539423 1.052031 0.796951
O -4.680684 0.625222 0.663162
C -3.635524 0.906660 -2.166719
C -4.195499 -0.374424 -2.057475
C -4.383393 1.913028 -2.796097
C -5.456772 -0.644382 -2.582781
H -3.642292 -1.169364 -1.554042
C -5.646153 1.646697 -3.318049
H -3.959803 2.917233 -2.877996
C -6.186173 0.363377 -3.214407
H -5.877393 -1.646989 -2.491275
H -6.210443 2.440942 -3.809169
H -7.175249 0.150338 -3.622996
C -2.879010 2.221228 0.258060
C -1.622277 2.284385 0.940231
H -3.430333 3.055861 -0.167603
N -2.516981 0.418900 1.534895
N -1.391727 1.192447 1.630052
C -0.646689 3.389964 0.923620
C 0.411265 3.416401 1.841567
C -0.772281 4.442043 0.007141
C 1.330139 4.463808 1.833694
H 0.503576 2.599523 2.558528
C 0.147930 5.487329 -0.003253
H -1.589189 4.431753 -0.717140
C 1.205356 5.502035 0.908939
H 2.149755 4.470438 2.554966
H 0.041065 6.295681 -0.728569
H 1.924162 6.323058 0.902659
C -2.694324 -0.666392 2.409633
C -1.941690 -0.744548 3.590878
C -3.651345 -1.654285 2.132927
C -2.158293 -1.787361 4.487531
H -1.198625 0.025711 3.791015
C -3.861702 -2.686153 3.045179
H -4.233753 -1.588700 1.216973
C -3.124305 -2.760099 4.227055
H -1.569417 -1.832964 5.405840
H -4.613107 -3.446292 2.822606
H -3.297931 -3.570425 4.936755
Se 2.758405 1.845488 -0.408622
----
back_face_R_3-TS1

Frequencies, energies and thermodynamic properties:

Lowest Vibrational Mode (1/cm) =	-249.6306
2nd Lowest Vibrational Mode (1/cm) =	18.1155
E(RM062X) (a.u.) =	-4429.88822756
Thermal correction to Enthalpy (a.u.) =	0.761190
Thermal correction to Gibbs Free Energy (a.u.) =	0.644650
Total Entropy (cal/Kmol) =	245.278
Esp(RM062X) (a.u.) =	-4432.40007135

Optimised cartesian coordinates (Angstrom):

C	-7.180096	-1.763845	0.678463
C	-6.788263	-3.087504	0.445850
C	-5.522470	-3.372893	-0.059089
C	-4.656645	-2.314098	-0.330096
C	-5.056190	-0.993518	-0.106704
H	-7.476779	-3.903662	0.665615
H	-5.213094	-4.403642	-0.236368
C	-2.893330	-0.521494	-0.834378
C	-1.941471	1.731193	-0.945168
C	-3.389150	2.139831	-1.271162
C	-4.352321	1.397676	-0.360624
H	-5.385821	1.549135	-0.698441
N	-4.075190	-0.039548	-0.428838
N	-1.834938	0.258480	-1.056508
C	-3.623046	3.668860	-1.275962
C	-4.033564	4.254821	0.078670
C	-4.660365	4.035145	-2.339432
H	-2.667455	4.132594	-1.571289
H	-5.049881	3.932887	0.354962
H	-3.349037	3.976442	0.891831
H	-4.049032	5.351729	0.013183
H	-4.335847	3.721864	-3.341623
H	-5.628179	3.553694	-2.127769
H	-4.827733	5.121125	-2.357095
H	-3.568756	1.775993	-2.296361
H	-1.306858	2.128486	-1.748193
C	-1.430855	2.263336	0.377517
C	-0.669365	3.437691	0.383756
C	-1.744894	1.647225	1.591387
C	-0.258539	4.007863	1.586903
H	-0.391325	3.908184	-0.562806
C	-1.336220	2.219178	2.796573
H	-2.298210	0.705234	1.609245
C	-0.599831	3.403432	2.797564
H	0.333966	4.923689	1.578305
H	-1.588574	1.728438	3.737028
H	-0.280088	3.847972	3.740935
C	0.586364	0.429184	-1.282088
H	0.535913	1.360910	-0.728349
C	1.829986	-0.067478	-1.674259
O	-0.625744	-1.523419	-1.839389
C	-0.571652	-0.356311	-1.450323
H	-4.275889	1.730209	0.685352
C	-6.322173	-0.702071	0.407119
H	-6.633304	0.322746	0.605920
H	-8.170988	-1.555757	1.082685
H	1.870386	-0.815024	-2.470632
C	3.640855	-1.996490	-0.816025
O	4.138384	-2.384412	-1.875540
C	3.044664	0.740886	-1.484334
C	3.173491	1.614414	-0.389996
C	4.105646	0.637652	-2.398635
C	4.326702	2.377896	-0.230406
H	2.372975	1.677628	0.350292
C	5.255492	1.406543	-2.236941
H	4.024939	-0.065302	-3.228207
C	5.367762	2.280926	-1.155678
H	4.418734	3.045752	0.627785
H	6.073096	1.315724	-2.953895
H	6.272917	2.876491	-1.025107
C	2.269704	-1.918308	-0.396200
C	2.307996	-1.399593	0.931733
H	1.454560	-2.456529	-0.872116
N	4.344263	-1.400753	0.254472
N	3.523611	-1.057701	1.290418
C	1.165038	-1.245195	1.848104
C	-0.098474	-1.738911	1.491196
C	1.328659	-0.660942	3.113869
C	-1.164222	-1.680494	2.389994
H	-0.239143	-2.198494	0.511408
C	0.263019	-0.599278	4.006640
H	2.311795	-0.277759	3.389844
C	-0.986443	-1.116878	3.653115
H	-2.136517	-2.089013	2.102012
H	0.407634	-0.149759	4.990862
H	-1.816994	-1.078609	4.360416
C	5.694977	-1.020706	0.289840
C	6.146019	-0.176284	1.317608
C	6.603525	-1.458590	-0.688072
C	7.475374	0.232394	1.354088
H	5.435475	0.160337	2.069320
C	7.929875	-1.033007	-0.637769
H	6.252561	-2.113021	-1.480472
C	8.378325	-0.185200	0.374739
H	7.807289	0.892396	2.158003
H	8.623657	-1.377012	-1.407464

H 9.418860 0.140950 0.404872
Se -2.894529 -2.398826 -1.024394
----
back_face_S_1-TS1
Frequencies, energies and thermodynamic properties:
Lowest Vibrational Mode (1/cm) = -281.6039
2nd Lowest Vibrational Mode (1/cm) = 14.5254
E(RM062X) (a.u.) = -4429.87819415
Thermal correction to Enthalpy (a.u.) = 0.760923
Thermal correction to Gibbs Free Energy (a.u.) = 0.641696
Total Entropy (cal/Kmol) = 250.933
Esp(RM062X) (a.u.) = -4432.39102193
Optimised cartesian coordinates (Angstrom):
C -6.324424 -3.090353 -0.279649
C -5.509299 -4.225821 -0.363423
C -4.137294 -4.098985 -0.563513
C -3.591858 -2.820649 -0.681486
C -4.411699 -1.691071 -0.603122
H -5.950665 -5.218122 -0.267625
H -3.499768 -4.982199 -0.624858
C -2.384180 -0.573427 -0.847497
C -2.142202 1.859708 -0.764468
C -3.539786 1.857472 -1.409292
C -4.416538 0.809147 -0.745807
H -5.344826 0.675066 -1.316890
N -3.716299 -0.476975 -0.732267
N -1.582708 0.494945 -0.848685
C -4.203471 3.253818 -1.460662
C -5.051580 3.600338 -0.233045
C -5.044092 3.388168 -2.732121
H -3.384510 3.989371 -1.526053
H -5.961001 2.980664 -0.195916
H -4.508166 3.472958 0.713430
H -5.376335 4.648301 -0.295984
H -4.430994 3.254391 -3.634395
H -5.850349 2.637925 -2.753161
H -5.513872 4.380471 -2.780780
H -3.376373 1.530799 -2.449403
H -1.501520 2.483184 -1.401434
C -2.107579 2.406570 0.647572
C -1.738316 3.738418 0.862522
C -2.486638 1.620450 1.739068
C -1.769716 4.282328 2.145792
H -1.419707 4.355909 0.018620
C -2.515550 2.161607 3.024273
H -2.748139 0.570066 1.592121
C -2.162755 3.495690 3.229159
H -1.479758 5.322368 2.300669
H -2.812503 1.538741 3.869691
H -2.184068 3.918817 4.234179
C 0.685948 1.373915 -0.566006
H 0.268771 2.320944 -0.240464
C 2.071377 1.194650 -0.615106
O 0.225853 -0.848151 -1.218969
C -0.142453 0.280683 -0.900596
H -4.682610 1.083206 0.286294
C -5.788381 -1.811400 -0.396794
H -6.429903 -0.934685 -0.316199
H -7.396240 -3.203974 -0.115450
H 2.423027 0.291527 -1.123167
C 4.141354 0.267439 1.052689
O 5.009327 1.125783 1.184407
C 2.986215 2.348610 -0.645572
C 4.181927 2.253823 -1.368588
C 2.693918 3.546963 0.023966
C 5.049147 3.341212 -1.456235
H 4.432399 1.311870 -1.862952
C 3.567342 4.626857 -0.048404
H 1.781561 3.628903 0.619495
C 4.744509 4.530052 -0.796391
H 5.972767 3.253432 -2.030470
H 3.332749 5.550882 0.482404
H 5.426436 5.379893 -0.854415
C 2.729009 0.283190 1.346758
C 2.300738 -1.076346 1.171752
H 2.273030 1.034451 1.985836
N 4.332962 -1.011060 0.475743
N 3.232868 -1.810507 0.614693
C 5.498300 -1.532732 -0.108650
C 5.523710 -2.879313 -0.509716
C 6.635042 -0.732305 -0.319434
C 6.663661 -3.410630 -1.105467
H 4.638395 -3.490420 -0.349232
C 7.766189 -1.284752 -0.918438
H 6.617615 0.307497 -0.005187
C 7.795332 -2.621280 -1.314999
H 6.663975 -4.458995 -1.409861
H 8.641044 -0.650780 -1.075154
H 8.686656 -3.042573 -1.781564
C 1.026884 -1.680612 1.608839
C 0.861526 -3.074186 1.569284
C -0.005130 -0.895675 2.142055
C -0.293973 -3.669076 2.066723
H 1.668504 -3.682469 1.159865
C -1.161206 -1.497176 2.645647

H 0.103184 0.191359 2.180106
C -1.309994 -2.883257 2.615632
H -0.399258 -4.755223 2.036586
H -1.943320 -0.879198 3.090598
H -2.210186 -3.348538 3.020581
Se -1.775863 -2.349826 -0.949802
----
back_face_S_2-lact
Frequencies, energies and thermodynamic properties:
Lowest Vibrational Mode (1/cm) = 14.4502
2nd Lowest Vibrational Mode (1/cm) = 15.6483
E(RM062X) (a.u.) = -4429.92420980
Thermal correction to Enthalpy (a.u.) = 0.763951
Thermal correction to Gibbs Free Energy (a.u.) = 0.646717
Total Entropy (cal/Kmol) = 246.739
Esp(RM062X) (a.u.) = -4432.43176005
Optimised cartesian coordinates (Angstrom):
C 7.125258 0.451653 -1.306207
C 6.802724 0.858362 -2.604606
C 5.499939 0.723496 -3.082651
C 4.526597 0.174008 -2.250553
C 4.859918 -0.242513 -0.958469
H 7.572225 1.288386 -3.246451
H 5.245079 1.046798 -4.093387
C 2.586124 -0.775676 -0.863278
C 1.438820 -1.644282 1.094689
C 2.812469 -2.246928 1.445119
C 3.920495 -1.253811 1.136101
H 4.898531 -1.745261 1.221321
N 3.776455 -0.775598 -0.237811
N 1.467449 -1.175774 -0.299072
C 2.895978 -2.820429 2.878265
C 3.330446 -1.810232 3.944717
C 3.822362 -4.038015 2.904744
H 1.881963 -3.168992 3.136466
H 4.387054 -1.528508 3.813417
H 2.724723 -0.893687 3.935983
H 3.238975 -2.265836 4.940832
H 3.469655 -4.826255 2.224732
H 4.845327 -3.761308 2.603913
H 3.878631 -4.459492 3.918298
H 2.938714 -3.090306 0.746028
H 0.729772 -2.481519 1.126618
C 0.946515 -0.575129 2.052216
C 0.021790 -0.918711 3.042280
C 1.410435 0.743490 1.984523
C -0.412593 0.030069 3.969494
H -0.368104 -1.939203 3.087958
C 0.972572 1.694806 2.902750
H 2.082911 1.048888 1.178882
C 0.064668 1.338078 3.902351
H -1.134512 -0.252585 4.737006
H 1.319482 2.727061 2.818724
H -0.283049 2.086452 4.616169
C -0.992126 -1.712521 -0.555146
H -1.090482 -1.646340 0.537503
C -2.319540 -1.230828 -1.175015
O 0.357278 -0.857633 -2.324449
C 0.171619 -0.857158 -1.075087
H 3.906421 -0.392345 1.821975
C 6.159456 -0.101038 -0.467263
H 6.420894 -0.402166 0.546506
H 8.144702 0.569494 -0.937605
H -2.238438 -1.370183 -2.265484
C -1.290881 0.954328 -0.526383
O -0.033218 0.561878 -0.527284
C -3.467877 -2.080500 -0.666656
C -4.020880 -3.075366 -1.477512
C -3.987641 -1.895515 0.620136
C -5.086654 -3.855401 -1.025730
H -3.623078 -3.226723 -2.484204
C -5.053302 -2.669759 1.074260
H -3.568401 -1.113879 1.259650
C -5.610682 -3.649651 0.249823
H -5.513749 -4.620330 -1.676562
H -5.457568 -2.502810 2.074155
H -6.451020 -4.250010 0.601981
C -2.428775 0.235816 -0.841403
C -3.482817 1.167167 -0.598120
N -1.671307 2.201719 -0.129909
N -3.015895 2.329437 -0.163835
C -0.847974 3.238562 0.367361
C -1.351552 4.090247 1.354675
C 0.455127 3.404015 -0.114063
C -0.542927 5.101555 1.868057
H -2.371938 3.943952 1.706505
C 1.255851 4.414027 0.416041
H 0.834155 2.740971 -0.890395
C 0.764724 5.264814 1.407537
H -0.938382 5.764370 2.639303
H 2.272278 4.541617 0.040101
H 1.395860 6.055671 1.814905
C -4.936403 0.965818 -0.757071
C -5.837035 1.610677 0.101097
C -5.436386 0.118150 -1.753931

C -7.208151 1.396105 -0.023884
H -5.445021 2.272518 0.874637
C -6.806723 -0.103803 -1.872332
H -4.742830 -0.370609 -2.440222
C -7.696782 0.531746 -1.005097
H -7.899624 1.898637 0.654603
H -7.181637 -0.773025 -2.648587
H -8.769913 0.356206 -1.095942
Se 2.677540 -0.114561 -2.638992
H -0.792475 -2.755345 -0.840047
----
back_face_S_2-tet-pt
Frequencies, energies and thermodynamic properties:
Lowest Vibrational Mode (1/cm) = 7.3761
2nd Lowest Vibrational Mode (1/cm) = 14.3581
E(RM062X) (a.u.) = -4429.91977290
Thermal correction to Enthalpy (a.u.) = 0.764078
Thermal correction to Gibbs Free Energy (a.u.) = 0.644262
Total Entropy (cal/Kmol) = 252.174
Esp(RM062X) (a.u.) = -4432.42954307
Optimised cartesian coordinates (Angstrom):
C 6.695506 1.873989 -0.967236
C 6.154692 2.822244 -1.844861
C 4.846998 2.698660 -2.304242
C 4.088537 1.610077 -1.873442
C 4.636313 0.659524 -1.007437
H 6.760622 3.668812 -2.168338
H 4.422273 3.439801 -2.982288
C 2.515166 -0.280065 -1.169157
C 1.770346 -2.184821 0.174936
C 3.226630 -2.661519 0.021440
C 4.172868 -1.485970 0.191903
H 5.190091 -1.768584 -0.109719
N 3.750613 -0.377664 -0.669328
N 1.534842 -1.131199 -0.837723
C 3.594869 -3.860257 0.927656
C 4.142501 -3.474944 2.305381
C 4.589461 -4.772566 0.206630
H 2.666509 -4.435041 1.080367
H 5.145269 -3.027881 2.219291
H 3.493109 -2.769623 2.842090
H 4.243450 -4.377470 2.924183
H 4.173604 -5.154949 -0.736135
H 5.521029 -4.232523 -0.025545
H 4.853469 -5.631927 0.838693
H 3.310910 -3.000183 -1.024360
H 1.132284 -3.029359 -0.113837
C 1.402847 -1.759888 1.581818
C 0.767228 -2.684562 2.416466
C 1.739316 -0.501242 2.089187
C 0.506207 -2.373846 3.750472
H 0.472727 -3.659551 2.019691
C 1.479234 -0.189447 3.422107
H 2.173694 0.259207 1.438308
C 0.870878 -1.127126 4.257678
H 0.011520 -3.104935 4.391178
H 1.738639 0.798351 3.805449
H 0.665234 -0.878450 5.299729
C -0.910657 -1.680287 -0.785863
H -0.775205 -1.769662 0.298557
C -2.270471 -1.024839 -1.097554
O 0.129161 -0.239590 -2.387731
C 0.230028 -0.913531 -1.392669
H 4.203982 -1.127913 1.231691
C 5.946459 0.783753 -0.537129
H 6.373341 0.059455 0.155391
H 7.718640 1.989792 -0.609135
H -2.327988 -0.950561 -2.197860
C -1.318442 1.042846 0.151223
O -0.090288 0.776116 0.247508
C -3.353250 -2.001089 -0.654639
C -3.897145 -2.907989 -1.569630
C -3.790797 -2.044286 0.673333
C -4.866366 -3.830504 -1.174692
H -3.566395 -2.878880 -2.611740
C -4.758161 -2.965662 1.073272
H -3.377917 -1.330936 1.390630
C -5.302278 -3.860192 0.149963
H -5.288480 -4.522315 -1.905854
H -5.094883 -2.981182 2.111394
H -6.066124 -4.574597 0.461259
C -2.381837 0.338197 -0.462337
C -3.528710 1.150065 -0.251059
N -1.918090 2.198717 0.681130
N -3.253623 2.246965 0.434487
C -1.313153 3.229486 1.416492
C -2.123021 4.225275 1.990404
C 0.080571 3.298197 1.593270
C -1.551070 5.258130 2.726753
H -3.199193 4.167855 1.843447
C 0.633324 4.340220 2.336758
H 0.705248 2.531904 1.142894
C -0.168981 5.325922 2.910529
H -2.198948 6.020322 3.164114
H 1.717304 4.379367 2.463542

H 0.274846 6.136534 3.489975
C -4.922328 0.915894 -0.686724
C -5.989579 1.377418 0.097844
C -5.210383 0.255703 -1.888718
C -7.307808 1.175031 -0.304165
H -5.763670 1.893752 1.031739
C -6.529510 0.048732 -2.289641
H -4.391709 -0.089472 -2.522061
C -7.583678 0.505989 -1.498402
H -8.126686 1.536398 0.320637
H -6.734675 -0.468151 -3.228850
H -8.616320 0.342487 -1.811088
Se 2.302258 1.184752 -2.325670
H -0.897676 -2.695739 -1.218332

----

back_face_S_2-tet
Frequencies, energies and thermodynamic properties:
Lowest Vibrational Mode (1/cm) = 21.1679
2nd Lowest Vibrational Mode (1/cm) = 26.1381
E(RM062X) (a.u.) = -4429.91026433
Thermal correction to Enthalpy (a.u.) = 0.763084
Thermal correction to Gibbs Free Energy (a.u.) = 0.646830
Total Entropy (cal/Kmol) = 244.677
Esp(RM062X) (a.u.) = -4432.42003536
Optimised cartesian coordinates (Angstrom):
C -6.678835 0.641959 0.145892
C -6.765370 -0.738560 -0.064779
C -5.643865 -1.463913 -0.463493
C -4.436193 -0.793269 -0.651276
C -4.359417 0.587042 -0.443706
H -7.715160 -1.252232 0.087315
H -5.707423 -2.541894 -0.622300
C -2.110780 0.235020 -0.959381
C -0.406060 1.961547 -0.859360
C -1.544506 2.896453 -1.316097
C -2.823964 2.557145 -0.569588
H -3.677311 3.076347 -1.027008
N -3.078344 1.121175 -0.659974
N -0.833419 0.569525 -1.058063
C -1.194237 4.401684 -1.250370
C -1.550926 5.082765 0.075028
C -1.858644 5.145822 -2.410766
H -0.103200 4.478528 -1.389004
H -2.642820 5.169159 0.190103
H -1.152301 4.552953 0.951442
H -1.142878 6.103351 0.084960
H -1.533837 4.746989 -3.382064
H -2.955386 5.058838 -2.355726
H -1.609039 6.215772 -2.378250
H -1.711195 2.642821 -2.375945
H 0.434767 2.114695 -1.548392
C 0.064677 2.235172 0.554564
C 1.152224 3.090749 0.760328
C -0.630728 1.743204 1.664171
C 1.502838 3.495310 2.047383
H 1.721767 3.455898 -0.098212
C -0.272941 2.136358 2.954324
H -1.462339 1.047234 1.526798
C 0.783596 3.027809 3.147632
H 2.346985 4.171403 2.191054
H -0.826338 1.747929 3.810975
H 1.056295 3.342712 4.156139
C 1.478597 -0.193124 -1.199277
H 1.786056 0.731130 -0.718921
C 2.498882 -1.265273 -1.437720
O -0.367274 -1.578331 -1.726681
C 0.153719 -0.495484 -1.360701
H -2.772627 2.848627 0.490650
C -5.476661 1.321398 -0.039271
H -5.416438 2.394327 0.140784
H -7.560736 1.198586 0.464352
H 2.102404 -1.954568 -2.201700
C 1.473349 -3.084546 -0.023220
O 1.170581 -4.043168 -0.690054
C 3.856551 -0.775859 -1.923044
C 4.782686 -1.716763 -2.398175
C 4.237919 0.568471 -1.899877
C 6.056025 -1.333545 -2.811239
H 4.497348 -2.771614 -2.443424
C 5.514387 0.958733 -2.312364
H 3.538866 1.327387 -1.548867
C 6.430712 0.011424 -2.762802
H 6.757414 -2.085403 -3.177002
H 5.792262 2.013608 -2.276540
H 7.428115 0.316968 -3.082699
C 2.694879 -2.196656 -0.163338
C 2.615864 -1.405267 1.105108
H 3.597785 -2.811329 -0.275154
N 0.774350 -2.550116 1.054402
N 1.499345 -1.583076 1.717044
C -0.563260 -2.786159 1.417588
C -1.233320 -1.835674 2.201941
C -1.238754 -3.936222 0.987791
C -2.574602 -2.022461 2.524544
H -0.686767 -0.959850 2.547482

C -2.581740 -4.106405 1.322273
H -0.716544 -4.672930 0.383539
C -3.260771 -3.155102 2.083025
H -3.088196 -1.271179 3.127929
H -3.101597 -5.002746 0.979039
H -4.312733 -3.296144 2.334977
C 3.618091 -0.443276 1.586678
C 3.244140 0.568577 2.482729
C 4.951480 -0.527549 1.163296
C 4.190779 1.473326 2.952548
H 2.199079 0.642602 2.788799
C 5.897276 0.380513 1.636522
H 5.253040 -1.303073 0.456682
C 5.520338 1.380998 2.532453
H 3.888301 2.261221 3.644853
H 6.932443 0.306445 1.300008
H 6.261114 2.092665 2.900436
Se -2.757052 -1.524236 -1.172145
----
back_face_S_2-TS1
Frequencies, energies and thermodynamic properties:
Lowest Vibrational Mode (1/cm) = -284.1417
2nd Lowest Vibrational Mode (1/cm) = 17.6848
E(RM062X) (a.u.) = -4429.89174238
Thermal correction to Enthalpy (a.u.) = 0.761001
Thermal correction to Gibbs Free Energy (a.u.) = 0.644963
Total Entropy (cal/Kmol) = 244.223
Esp(RM062X) (a.u.) = -4432.40325571
Esp(RM062X) gas (a.u.) = -4432.37454462
Esp(RM062X) gas nuc (a.u.) = -762.966215426
Esp(RM062X) gas cat (a.u.) = -3669.27336885
Optimised cartesian coordinates (Angstrom):
C -6.756225 0.544642 0.296917
C -6.840046 -0.832796 0.057185
C -5.723181 -1.546104 -0.369021
C -4.521777 -0.862252 -0.557142
C -4.445313 0.512525 -0.319713
H -7.786131 -1.352781 0.209858
H -5.783464 -2.620482 -0.549099
C -2.205439 0.177936 -0.865273
C -0.514465 1.948392 -0.866791
C -1.699532 2.837500 -1.291282
C -2.924442 2.493822 -0.462428
H -3.810670 2.998856 -0.868582
N -3.167479 1.053159 -0.537881
N -0.921122 0.533207 -0.987169
C -1.386632 4.352270 -1.296355
C -1.687293 5.068259 0.024452
C -2.137959 5.035212 -2.441284
H -0.308341 4.453009 -1.501536
H -2.772907 5.130078 0.198807
H -1.225317 4.579877 0.893683
H -1.308466 6.098864 -0.022346
H -1.853605 4.613578 -3.415553
H -3.226711 4.916732 -2.322962
H -1.921675 6.112602 -2.457215
H -1.920386 2.543764 -2.330805
H 0.274494 2.096357 -1.615088
C 0.037295 2.290015 0.500526
C 1.105680 3.187740 0.598733
C -0.565771 1.817974 1.670241
C 1.526938 3.651900 1.843689
H 1.602553 3.539793 -0.309058
C -0.132141 2.266237 2.918191
H -1.379248 1.090021 1.613139
C 0.903867 3.197462 3.006481
H 2.350231 4.365141 1.904728
H -0.608769 1.889046 3.824162
H 1.235527 3.555242 3.982337
C 1.428167 -0.143040 -1.216467
H 1.724322 0.816131 -0.802651
C 2.392367 -1.075612 -1.612743
O -0.393886 -1.597141 -1.611481
C 0.061939 -0.501221 -1.292763
H -2.810056 2.789079 0.591345
C -5.561243 1.233768 0.113828
H -5.503070 2.302031 0.319736
H -7.636703 1.089320 0.638631
H 2.019396 -1.950198 -2.153071
C 1.689046 -3.183581 0.053004
O 1.357370 -4.166409 -0.601605
C 3.775746 -0.661885 -1.953935
C 4.578880 -1.525204 -2.715993
C 4.312970 0.571962 -1.558781
C 5.878771 -1.172206 -3.068163
H 4.171282 -2.489619 -3.029231
C 5.613589 0.927173 -1.911242
H 3.721453 1.259042 -0.951103
C 6.402413 0.058035 -2.664970
H 6.485303 -1.858034 -3.661766
H 6.015608 1.888178 -1.585982
H 7.420892 0.337586 -2.938880
C 2.922880 -2.421861 0.054456
C 2.777522 -1.472451 1.109450
H 3.847646 -2.814242 -0.357771

N 0.884782 -2.484498 0.973204
N 1.556817 -1.455933 1.582299
C -0.448611 -2.730413 1.323031
C -1.131426 -1.790330 2.114297
C -1.114210 -3.898941 0.917559
C -2.457367 -2.010403 2.478150
H -0.593905 -0.903752 2.444564
C -2.439722 -4.104177 1.298342
H -0.585986 -4.623002 0.303793
C -3.124861 -3.167845 2.073478
H -2.972109 -1.267676 3.091851
H -2.943323 -5.018779 0.978626
H -4.162919 -3.338658 2.362533
C 3.776624 -0.481651 1.539186
C 3.369794 0.717693 2.140446
C 5.142519 -0.708380 1.324247
C 4.314944 1.667036 2.522720
H 2.302664 0.897171 2.286599
C 6.086020 0.242218 1.707826
H 5.467144 -1.634150 0.844950
C 5.675530 1.433590 2.307776
H 3.987161 2.598406 2.988259
H 7.146622 0.054570 1.532229
H 6.413695 2.179733 2.606568
Se -2.852534 -1.570413 -1.100922

----

back_face_S_2-TS2
Frequencies, energies and thermodynamic properties:
Lowest Vibrational Mode (1/cm) = -120.5189
2nd Lowest Vibrational Mode (1/cm) = 13.8291
E(RM062X) (a.u.) = -4429.91373818
Thermal correction to Enthalpy (a.u.) = 0.762647
Thermal correction to Gibbs Free Energy (a.u.) = 0.646747
Total Entropy (cal/Kmol) = 243.932
Esp(RM062X) (a.u.) = -4432.42181759
Esp(RM062X) gas (a.u.) = -4432.39606832
Esp(RM062X) gas nuc (a.u.) = -762.120425686
Esp(RM062X) gas cat (a.u.) = -3669.94563001
Optimised cartesian coordinates (Angstrom):
C 6.851107 -0.041054 1.032631
C 7.102466 0.716758 -0.117516
C 6.132668 0.843155 -1.108468
C 4.907147 0.201289 -0.932062
C 4.665693 -0.562702 0.212792
H 8.063746 1.216814 -0.237950
H 6.325790 1.437205 -2.002719
C 2.575872 -0.856256 -0.776002
C 0.716120 -1.954490 0.338914
C 1.812231 -2.885971 0.896104
C 2.996475 -2.044290 1.335885
H 3.858588 -2.684329 1.566420
N 3.390015 -1.150182 0.245709
N 1.295664 -1.232948 -0.813003
C 1.337081 -3.867207 1.994342
C 1.497203 -3.349089 3.426962
C 2.065005 -5.204991 1.842693
H 0.265774 -4.053031 1.814447
H 2.560784 -3.296000 3.707464
H 1.050184 -2.356966 3.575863
H 1.012858 -4.045369 4.125870
H 1.873753 -5.657438 0.859420
H 3.153250 -5.072897 1.951014
H 1.737759 -5.913638 2.616380
H 2.139828 -3.491223 0.034963
H -0.072902 -2.598791 -0.070426
C 0.114135 -1.026854 1.379518
C -0.984458 -1.465401 2.127059
C 0.635708 0.246071 1.624773
C -1.556885 -0.645929 3.096864
H -1.410979 -2.454552 1.938192
C 0.060244 1.070295 2.591754
H 1.450566 0.637937 1.012515
C -1.036626 0.627846 3.328861
H -2.424173 -0.995592 3.658981
H 0.458814 2.074345 2.748143
H -1.497110 1.280987 4.071382
C -0.936866 -1.250045 -2.114549
H -0.928703 -2.334796 -2.319319
C -2.042022 -0.958570 -1.069092
O 1.163561 -0.617375 -2.998294
C 0.520109 -0.838141 -1.990802
H 2.761863 -1.436824 2.223106
C 5.632962 -0.689037 1.213123
H 5.443317 -1.266006 2.117423
H 7.616887 -0.124819 1.804111
H -1.922785 -1.664529 -0.231062
C -0.892355 1.298400 -0.740538
O 0.219218 1.089176 -1.324745
C -1.990058 0.448295 -0.553213
C -2.900189 1.203185 0.238391
N -2.440203 2.414124 0.505735
N -1.220494 2.483643 -0.086460
C -0.388400 3.589883 0.153573
C -0.728920 4.494065 1.172764
C 0.774598 3.809554 -0.602450

C 0.083936 5.594180 1.430379
H -1.634411 4.312501 1.748101
C 1.579781 4.913459 -0.324234
H 1.032038 3.111170 -1.394198
C 1.246191 5.812233 0.688363
H -0.195383 6.287820 2.225674
H 2.481015 5.072717 -0.919372
H 1.881667 6.674169 0.895744
C -4.231745 0.814471 0.748642
C -4.517333 -0.509522 1.109399
C -5.237093 1.781701 0.892378
C -5.779431 -0.861099 1.585822
H -3.738347 -1.268706 1.026347
C -6.496969 1.431876 1.373269
H -5.012463 2.811845 0.613270
C -6.775256 0.107590 1.716914
H -5.985469 -1.897740 1.858321
H -7.270155 2.195797 1.473175
H -7.764483 -0.168230 2.085872
C -3.363717 -1.336306 -1.731219
C -4.096969 -0.409555 -2.478501
C -3.846604 -2.644267 -1.623724
C -5.292907 -0.779931 -3.092535
H -3.731395 0.616714 -2.557674
C -5.042672 -3.019666 -2.235629
H -3.284168 -3.376444 -1.036547
C -5.771820 -2.085316 -2.971118
H -5.858566 -0.042250 -3.664372
H -5.409305 -4.042390 -2.131828
H -6.711810 -2.371992 -3.445261
Se 3.420185 0.175411 -2.106046
H -1.223891 -0.768031 -3.058559
----
back_face_S_2-TS3
Frequencies, energies and thermodynamic properties:
Lowest Vibrational Mode (1/cm) = -152.6017
2nd Lowest Vibrational Mode (1/cm) = 10.8097
E(RM062X) (a.u.) = -4429.91708535
Thermal correction to Enthalpy (a.u.) = 0.762499
Thermal correction to Gibbs Free Energy (a.u.) = 0.645275
Total Entropy (cal/Kmol) = 246.718
Esp(RM062X) (a.u.) = -4432.42478438
Optimised cartesian coordinates (Angstrom):
C 7.188167 0.583890 -0.324923
C 7.065248 1.271876 -1.533732
C 5.860405 1.244461 -2.238219
C 4.791935 0.518892 -1.721008
C 4.920727 -0.183067 -0.515180
H 7.909232 1.837919 -1.928742
H 5.756668 1.787122 -3.179102
C 2.659632 -0.736541 -0.910873
C 1.273556 -1.960218 0.588457
C 2.575683 -2.645979 1.049468
C 3.708288 -1.631866 1.120709
H 4.667213 -2.152653 1.250095
N 3.768402 -0.870256 -0.122773
N 1.501244 -1.228535 -0.653980
C 2.424563 -3.484173 2.338701
C 2.656025 -2.705448 3.637284
C 3.348006 -4.703418 2.290861
H 1.385409 -3.855221 2.349135
H 3.716961 -2.431170 3.747828
H 2.054782 -1.787658 3.696134
H 2.393773 -3.337090 4.497956
H 3.127364 -5.340200 1.422442
H 4.403457 -4.394363 2.226385
H 3.236464 -5.312042 3.199545
H 2.826376 -3.342914 0.232319
H 0.569118 -2.773696 0.360380
C 0.621938 -1.063231 1.627030
C -0.468061 -1.529466 2.369124
C 1.098288 0.230999 1.865976
C -1.055909 -0.728758 3.349792
H -0.866598 -2.528971 2.174010
C 0.518589 1.030287 2.848962
H 1.910405 0.639104 1.259105
C -0.559969 0.551582 3.595185
H -1.908285 -1.105045 3.917759
H 0.894371 2.043108 3.011293
H -1.021898 1.182937 4.355801
C -1.075472 -1.477876 -1.366366
H -1.057808 -1.704803 -0.294334
C -2.452579 -0.837257 -1.657867
O 0.486294 -0.425154 -2.865160
C 0.053067 -0.530647 -1.734583
H 3.580529 -0.937841 1.967296
C 6.120800 -0.146757 0.198089
H 6.227660 -0.668599 1.148409
H 8.128875 0.616806 0.225787
H -2.554176 -0.705958 -2.748113
C -1.234078 1.102955 -0.651927
O -0.003192 0.705739 -0.966015
C -3.550787 -1.771899 -1.187634
C -4.270089 -2.537941 -2.107849
C -3.847390 -1.896749 0.175013

C -5.283153 -3.398298 -1.681037
H -4.045694 -2.446498 -3.173531
C -4.858607 -2.753398 0.604419
H -3.294725 -1.294482 0.901908
C -5.583728 -3.503858 -0.323917
H -5.843152 -3.982357 -2.413261
H -5.088662 -2.830499 1.668399
H -6.381272 -4.168575 0.011548
C -2.434978 0.500354 -0.963351
C -3.399162 1.368290 -0.369462
N -1.487562 2.231781 0.066151
N -2.817025 2.387117 0.243239
C -0.573957 3.142561 0.651695
C -1.007661 3.923532 1.728253
C 0.740687 3.250505 0.184760
C -0.117235 4.793972 2.350496
H -2.037125 3.827299 2.068069
C 1.623095 4.117284 0.828455
H 1.075312 2.654519 -0.662001
C 1.205120 4.888410 1.913024
H -0.460272 5.397603 3.192159
H 2.649860 4.192241 0.466705
H 1.902322 5.564880 2.408724
C -4.869335 1.237716 -0.363956
C -5.612057 1.681591 0.738062
C -5.541064 0.662768 -1.450385
C -6.997783 1.537832 0.758513
H -5.086386 2.130287 1.582090
C -6.925975 0.511569 -1.425635
H -4.972804 0.334564 -2.322337
C -7.658013 0.945864 -0.319600
H -7.565635 1.881641 1.624603
H -7.435940 0.056294 -2.276171
H -8.742280 0.825424 -0.298947
Se 3.052963 0.335939 -2.458656
H -0.935059 -2.400573 -1.943057
----
back_face_S_3-TS1
Frequencies, energies and thermodynamic properties:
Lowest Vibrational Mode (1/cm) = -300.5000
2nd Lowest Vibrational Mode (1/cm) = 15.6841
E(RM062X) (a.u.) = -4429.88748653
Thermal correction to Enthalpy (a.u.) = 0.761381
Thermal correction to Gibbs Free Energy (a.u.) = 0.643701
Total Entropy (cal/Kmol) = 247.680
Esp(RM062X) (a.u.) = -4432.39923938
Optimised cartesian coordinates (Angstrom):
C -7.285672 1.623700 -1.552845
C -6.837756 2.895623 -1.928417
C -5.542407 3.306964 -1.625694
C -4.703421 2.430204 -0.938712
C -5.160144 1.166985 -0.554146
H -7.505163 3.568795 -2.466953
H -5.189441 4.295246 -1.923358
C -2.993265 0.958690 0.282317
C -2.120033 -1.066978 1.330733
C -3.573805 -1.267360 1.793509
C -4.529411 -0.923099 0.665885
H -5.560282 -0.886561 1.040916
N -4.204228 0.402622 0.136833
N -1.966912 0.318791 0.843230
C -3.848812 -2.658918 2.409905
C -4.295716 -3.723979 1.403550
C -4.879521 -2.538364 3.534343
H -2.901259 -2.995277 2.862290
H -5.309849 -3.511361 1.030263
H -3.620111 -3.808427 0.541156
H -4.332935 -4.704089 1.899402
H -4.530872 -1.863092 4.328314
H -5.835935 -2.148996 3.150755
H -5.078624 -3.521636 3.983088
H -3.728322 -0.518596 2.588027
H -1.496910 -1.136011 2.230627
C -1.638383 -2.086308 0.319265
C -0.923978 -3.202619 0.766547
C -1.949147 -1.975860 -1.038351
C -0.568734 -4.218179 -0.120189
H -0.647571 -3.281857 1.821389
C -1.596944 -2.990668 -1.926159
H -2.451627 -1.084684 -1.421748
C -0.919821 -4.120412 -1.467157
H -0.013830 -5.085267 0.241469
H -1.845726 -2.893941 -2.983688
H -0.650575 -4.915709 -2.164197
C 0.427256 0.228049 1.348324
H 0.357950 -0.851474 1.422255
C 1.686484 0.845799 1.358299
O -0.664478 2.182679 0.610184
C -0.678803 0.991218 0.935604
H -4.483025 -1.654755 -0.154994
C -6.455601 0.744406 -0.863874
H -6.812340 -0.247328 -0.588878
H -8.299034 1.310007 -1.804546
H 1.714271 1.917514 1.567907
C 1.818136 0.079036 -1.248627

O 0.739005 -0.478248 -1.437941
C 2.892826 0.088448 1.752002
C 3.981459 0.750620 2.336487
C 2.999412 -1.288299 1.499936
C 5.139597 0.053598 2.677720
H 3.919067 1.826685 2.515683
C 4.158469 -1.983946 1.831631
H 2.180314 -1.813608 1.001307
C 5.231558 -1.315369 2.425183
H 5.977015 0.584600 3.133301
H 4.229773 -3.050607 1.610642
H 6.141220 -1.860134 2.683078
C 2.106322 1.382514 -0.710095
C 3.534210 1.501166 -0.769422
H 1.369438 2.181806 -0.713420
N 3.095451 -0.500959 -1.413155
N 4.101094 0.380732 -1.140072
C 3.409201 -1.833586 -1.719729
C 4.751566 -2.197813 -1.913530
C 2.409526 -2.818686 -1.795688
C 5.084404 -3.522168 -2.182614
H 5.518555 -1.430116 -1.839196
C 2.765771 -4.140108 -2.062031
H 1.369113 -2.542349 -1.639267
C 4.096997 -4.505792 -2.259792
H 6.132978 -3.786806 -2.331327
H 1.978879 -4.895839 -2.111708
H 4.362717 -5.542775 -2.468971
C 4.332778 2.664081 -0.349342
C 5.735130 2.612853 -0.352413
C 3.698417 3.830646 0.098911
C 6.481403 3.702508 0.084724
H 6.225699 1.700947 -0.694528
C 4.448540 4.921503 0.539037
H 2.608036 3.886188 0.105884
C 5.841600 4.861512 0.533940
H 7.571480 3.649637 0.078922
H 3.940587 5.822524 0.886653
H 6.428882 5.714109 0.878401
Se -2.902492 2.710583 -0.410438
----
catAc-carbox_complex
Frequencies, energies and thermodynamic properties:
Lowest Vibrational Mode (1/cm) = 6.8960
2nd Lowest Vibrational Mode (1/cm) = 21.5406
E(RM062X) (a.u.) = -4013.83505681
Thermal correction to Enthalpy (a.u.) = 0.664838
Thermal correction to Gibbs Free Energy (a.u.) = 0.555360
Total Entropy (cal/Kmol) = 230.416
Esp(RM062X) (a.u.) = -4015.91203860
Optimised cartesian coordinates (Angstrom):
C -6.655789 -0.165306 0.711580
C -6.810925 1.198494 0.993666
C -5.709351 2.047433 1.012667
C -4.449024 1.510164 0.747694
C -4.296172 0.147328 0.479643
H -7.803921 1.600480 1.195772
H -5.828214 3.110429 1.225844
C -2.057638 0.734544 0.261498
C -0.323099 -0.827138 -0.471369
C -1.151465 -1.868448 0.281523
C -2.628017 -1.657226 0.018427
H -3.240018 -2.251867 0.709076
N -2.967669 -0.243647 0.243452
N -0.760223 0.516333 -0.023813
C -0.666527 -3.311884 0.027077
C -1.334513 -4.003933 -1.163915
C -0.834723 -4.149262 1.295810
H 0.415090 -3.229476 -0.172676
H -2.394910 -4.218335 -0.953990
H -1.279550 -3.408401 -2.086495
H -0.841939 -4.967459 -1.357322
H -0.261734 -3.712632 2.125961
H -1.893450 -4.205430 1.597302
H -0.480665 -5.177442 1.133433
H -0.944120 -1.642441 1.339591
H 0.685341 -0.976224 -0.080723
C -0.300353 -0.936734 -1.977505
C 0.802644 -1.570906 -2.563307
C -1.339483 -0.473343 -2.792949
C 0.845480 -1.771061 -3.942683
H 1.620571 -1.895981 -1.913428
C -1.292375 -0.671245 -4.172999
H -2.194810 0.054889 -2.362368
C -0.204197 -1.328262 -4.748768
H 1.706414 -2.268695 -4.391738
H -2.107505 -0.308074 -4.800628
H -0.169519 -1.483511 -5.828144
C 1.570924 1.316230 -0.245295
H 1.909602 0.300188 -0.443913
C 2.436876 2.347553 -0.245236
O -0.259076 2.688205 0.386705
C 0.162377 1.592999 0.068813
H -2.908804 -1.917427 -1.014017
C -5.402511 -0.707651 0.451359

H -5.296350 -1.767256 0.223780
H -7.530138 -0.816152 0.692387
H 2.045061 3.348646 -0.039362
C 3.875695 2.248768 -0.502215
C 4.531741 1.009713 -0.631666
C 4.623192 3.431415 -0.614903
C 5.900348 0.970596 -0.877631
H 3.974761 0.074927 -0.510717
C 5.993150 3.386676 -0.864744
H 4.118348 4.393752 -0.506817
C 6.633757 2.155161 -0.997944
H 6.403566 0.006807 -0.971776
H 6.561513 4.313560 -0.952538
H 7.707069 2.115357 -1.190235
Se -2.790560 2.414061 0.682295
O 1.410571 -1.004159 1.938089
C 2.471633 -1.427362 1.429634
O 2.647121 -1.680366 0.207452
C 3.676799 -1.612888 2.393432
C 4.036954 -0.225385 2.939355
C 3.237226 -2.516777 3.549535
C 4.876334 -2.222079 1.673302
H 4.343648 0.449418 2.123676
H 3.167122 0.218790 3.442600
H 4.869302 -0.292711 3.658583
H 2.977245 -3.523426 3.185057
H 4.044645 -2.620956 4.292084
H 2.352427 -2.091776 4.041991
H 5.710895 -2.373592 2.376879
H 4.613467 -3.191071 1.225456
H 5.221115 -1.564316 0.862643
----
catAc
Frequencies, energies and thermodynamic properties:
Lowest Vibrational Mode (1/cm) = 15.6212
2nd Lowest Vibrational Mode (1/cm) = 27.3948
E(RM062X) (a.u.) = -3667.66476411
Thermal correction to Enthalpy (a.u.) = 0.519763
Thermal correction to Gibbs Free Energy (a.u.) = 0.432087
Total Entropy (cal/Kmol) = 184.529
Esp(RM062X) (a.u.) = -3669.34088642
Esp(RM062X) gas (a.u.) = -3669.28927207
Optimised cartesian coordinates (Angstrom):
C -6.057523 -0.447069 0.335055
C -6.036238 -1.829973 0.563287
C -4.841127 -2.538725 0.515849
C -3.666244 -1.841218 0.230493
C -3.693023 -0.465383 -0.008633
H -6.964675 -2.355901 0.786168
H -4.822399 -3.613003 0.701592
C -1.397124 -0.753645 -0.233078
C 0.184350 1.093902 -0.645063
C -1.002936 1.723279 -1.391819
C -2.281723 1.510650 -0.605617
H -3.150729 1.801746 -1.208091
N -2.429595 0.087375 -0.283900
N -0.117878 -0.338428 -0.393254
C -0.779106 3.204370 -1.779596
C -1.222712 4.214378 -0.716476
C -1.474426 3.506166 -3.108656
H 0.304951 3.327704 -1.937995
H -2.320365 4.241455 -0.633186
H -0.805198 4.006035 0.278265
H -0.897407 5.221352 -1.012162
H -1.096924 2.862196 -3.914940
H -2.562160 3.352815 -3.029016
H -1.309022 4.552755 -3.399235
H -1.095269 1.147568 -2.327497
H 1.024154 1.106178 -1.350431
C 0.592291 1.807242 0.626820
C 1.625314 2.749642 0.575473
C -0.069770 1.584279 1.838489
C 1.968639 3.484106 1.708897
H 2.165799 2.913667 -0.360342
C 0.273705 2.319633 2.973021
H -0.856230 0.829064 1.915048
C 1.287258 3.275428 2.908371
H 2.773487 4.218143 1.655571
H -0.251175 2.140236 3.911958
H 1.554777 3.848935 3.796591
C 2.313653 -0.790290 -0.262256
H 2.511920 0.275678 -0.189396
C 3.317113 -1.689560 -0.302916
O 0.641749 -2.464345 -0.163795
C 0.932965 -1.292043 -0.269817
H -2.296319 2.085739 0.332439
C -4.891720 0.252609 0.048213
H -4.921284 1.329170 -0.113050
H -7.002235 0.094302 0.386364
H 3.039954 -2.746067 -0.372226
C 4.749507 -1.399411 -0.268375
C 5.258956 -0.100368 -0.090314
C 5.648979 -2.467321 -0.416269
C 6.630776 0.117900 -0.067561
H 4.580922 0.744618 0.041059

C 7.023989 -2.246604 -0.395757
H 5.259122 -3.478344 -0.549797
C 7.516206 -0.953729 -0.222072
H 7.016710 1.128036 0.074047
H 7.711781 -3.084588 -0.513631
H 8.592553 -0.777031 -0.203425
Se -1.910194 -2.525582 0.119046

HyperSe
Frequencies, energies and thermodynamic properties:
Lowest Vibrational Mode (1/cm) = 28.9982
2nd Lowest Vibrational Mode (1/cm) = 51.0892
E(RM062X) (a.u.) = -3245.89658069
Thermal correction to Enthalpy (a.u.) = 0.371536
Thermal correction to Gibbs Free Energy (a.u.) = 0.304461
Total Entropy (cal/Kmol) = 141.172
Esp(RM062X) (a.u.) = -3247.11262625
Optimised cartesian coordinates (Angstrom):
C -3.714705 1.859283 1.821658
C -4.688783 0.924979 1.470192
C -4.366578 -0.136066 0.619062
C -3.071566 -0.239110 0.127670
C -2.088033 0.708864 0.469574
H -5.702470 1.015099 1.861264
H -5.121121 -0.875071 0.345722
C -0.676040 -0.646646 -0.872712
C 1.606810 -0.308818 -1.231667
C 1.305136 1.193635 -1.019327
C 0.297960 1.375655 0.112800
H -0.065222 2.414093 0.125445
N -0.839164 0.491513 -0.099756
N 0.380547 -1.076083 -1.422801
C 2.554055 2.090165 -0.865958
C 3.046357 2.258160 0.575219
C 2.304929 3.464346 -1.491720
H 3.360554 1.605203 -1.442264
H 2.341243 2.867460 1.162388
H 3.184076 1.298203 1.092091
H 4.011186 2.785580 0.578478
H 2.058419 3.379824 -2.559772
H 1.468543 3.977207 -0.990315
H 3.192844 4.105708 -1.394608
H 0.792527 1.503263 -1.946021
H 2.185651 -0.386328 -2.165280
C 2.462272 -0.913340 -0.131992
C 3.846570 -1.022056 -0.303857
C 1.902472 -1.321815 1.084650
C 4.660494 -1.489829 0.727604
H 4.293229 -0.731528 -1.257793
C 2.713469 -1.789569 2.118459
H 0.820226 -1.279921 1.228413
C 4.096199 -1.867625 1.946083
H 5.738727 -1.564040 0.577042
H 2.262025 -2.099743 3.062238
H 4.729973 -2.234254 2.754959
H 0.753672 1.166608 1.095008
C -2.411067 1.760331 1.330994
H -1.657229 2.490494 1.624359
H -3.966894 2.680784 2.493399
Se -2.357333 -1.571468 -1.014919
----
nucH
Frequencies, energies and thermodynamic properties:
Lowest Vibrational Mode (1/cm) = 19.6629
2nd Lowest Vibrational Mode (1/cm) = 43.1322
E(RM062X) (a.u.) = -762.673752025
Thermal correction to Enthalpy (a.u.) = 0.254701
Thermal correction to Gibbs Free Energy (a.u.) = 0.197419
Total Entropy (cal/Kmol) = 120.560
Esp(RM062X) (a.u.) = -763.513551223
Optimised cartesian coordinates (Angstrom):
C -0.668082 1.770705 0.126391
O -1.632067 2.689573 0.229469
C 0.707164 1.856162 0.143521
C 1.133176 0.503971 0.023219
H 1.300067 2.760961 0.230663
N 0.102211 -0.319331 -0.059623
N -0.999633 0.454312 -0.002497
C -2.288903 -0.140188 -0.013598
C -2.433946 -1.427733 0.512675
C -3.388450 0.531425 -0.556041
C -3.684613 -2.038726 0.499088
H -1.560060 -1.932561 0.921657
C -4.637075 -0.088831 -0.550645
H -3.267743 1.526035 -0.980226
C -4.792033 -1.371527 -0.026651
H -3.794133 -3.043499 0.909620
H -5.494349 0.437196 -0.973071
H -5.771333 -1.851494 -0.030803
C 2.516615 -0.008157 -0.022830
C 3.607833 0.860561 0.103318
C 2.757086 -1.379261 -0.193767
C 4.912286 0.370284 0.060113
H 3.439923 1.930202 0.238376
C 4.060067 -1.867030 -0.236427

H 1.906611 -2.054177 -0.294423
C 5.143314 -0.994586 -0.109791
H 5.752159 1.059496 0.160193
H 4.233075 -2.936063 -0.370521
H 6.164089 -1.378174 -0.143862
H -1.236447 3.558213 0.380041
----
nuc
Frequencies, energies and thermodynamic properties:
Lowest Vibrational Mode (1/cm) = 25.2844
2nd Lowest Vibrational Mode (1/cm) = 30.6629
E(RM062X) (a.u.) = -762.196235259
Thermal correction to Enthalpy (a.u.) = 0.241039
Thermal correction to Gibbs Free Energy (a.u.) = 0.184338
Total Entropy (cal/Kmol) = 119.338
Esp(RM062X) (a.u.) = -763.041734140
Esp(RM062X) gas (a.u.) = -762.972930392
Optimised cartesian coordinates (Angstrom):
C 0.706800 1.887833 0.005458
O 1.555697 2.798353 0.007186
C -0.710637 1.893668 -0.000725
C -1.113351 0.542029 0.001152
H -1.323444 2.789518 0.001073
N -0.088526 -0.295479 0.005372
N 1.021874 0.495131 0.008903
C 2.285498 -0.102839 -0.000401
C 2.385203 -1.506274 -0.062156
C 3.469718 0.657601 0.053220
C 3.631142 -2.125368 -0.069843
H 1.467927 -2.088984 -0.103708
C 4.707517 0.016977 0.043512
H 3.389243 1.739564 0.100441
C 4.806052 -1.373161 -0.017205
H 3.681636 -3.215230 -0.118253
H 5.613854 0.624963 0.085712
H 5.780934 -1.862721 -0.023599
C -2.493432 0.013887 0.002593
C -3.594981 0.878423 -0.064217
C -2.731295 -1.367765 0.071065
C -4.896867 0.378450 -0.062146
H -3.428649 1.955296 -0.121273
C -4.030986 -1.867110 0.072708
H -1.874570 -2.040013 0.125711
C -5.121753 -0.996653 0.006242
H -5.741001 1.068480 -0.115123
H -4.196511 -2.944812 0.127815
H -6.140192 -1.388640 0.008250
----
prod
Frequencies, energies and thermodynamic properties:
Lowest Vibrational Mode (1/cm) = 21.2266
2nd Lowest Vibrational Mode (1/cm) = 32.1913
E(RM062X) (a.u.) = -1184.01376903
Thermal correction to Enthalpy (a.u.) = 0.390191
Thermal correction to Gibbs Free Energy (a.u.) = 0.315356
Total Entropy (cal/Kmol) = 157.504
Esp(RM062X) (a.u.) = -1185.31488074
Optimised cartesian coordinates (Angstrom):
C 0.337294 -2.901384 -0.027499
H 0.874011 -3.815966 -0.302347
C 1.006894 -1.640260 -0.616505
O -1.628957 -4.153834 -0.643197
C -1.116017 -3.109596 -0.393360
H 1.173188 -1.805810 -1.693837
C -1.305891 -0.790185 -0.266953
O -1.920620 -1.989817 -0.354090
C 0.029445 -0.513335 -0.423123
C 0.078296 0.907518 -0.299324
N -1.130292 1.406208 -0.080560
N -1.981782 0.365349 -0.058457
C -3.378645 0.575112 0.098938
C -3.924644 1.786491 -0.333810
C -4.184807 -0.402178 0.687783
C -5.288778 2.015155 -0.176968
H -3.270188 2.531823 -0.783173
C -5.551308 -0.163088 0.825130
H -3.751014 -1.336438 1.039677
C -6.107740 1.041496 0.396913
H -5.715119 2.961038 -0.513709
H -6.182000 -0.926189 1.282891
H -7.176858 1.222908 0.512479
C 1.255658 1.795997 -0.347342
C 2.345373 1.498951 -1.175394
C 1.297340 2.946086 0.451253
C 3.468810 2.322835 -1.185230
H 2.311212 0.618691 -1.819191
C 2.417964 3.773867 0.434121
H 0.445167 3.174676 1.092798
C 3.509246 3.460528 -0.377778
H 4.314140 2.077684 -1.830089
H 2.443691 4.663971 1.064653
H 4.390153 4.104267 -0.383394
C 2.361391 -1.423143 0.032737
C 2.463327 -0.856455 1.307969
C 3.528595 -1.793813 -0.639045

C 3.711046 -0.644771 1.890174
H 1.556991 -0.551715 1.837988
C 4.780057 -1.585068 -0.057642
H 3.458219 -2.234585 -1.636560
C 4.874110 -1.005011 1.206546
H 3.777055 -0.188951 2.879265
H 5.683745 -1.871095 -0.598100
H 5.851517 -0.832023 1.659328
H 0.355130 -2.818484 1.073209
----
top_face_R_1-TS1
Frequencies, energies and thermodynamic properties:
Lowest Vibrational Mode (1/cm) = -342.3924
2nd Lowest Vibrational Mode (1/cm) = 6.5428
E(RM062X) (a.u.) = -4429.88848730
Thermal correction to Enthalpy (a.u.) = 0.761426
Thermal correction to Gibbs Free Energy (a.u.) = 0.640015
Total Entropy (cal/Kmol) = 255.531
Esp(RM062X) (a.u.) = -4432.40021957
Optimised cartesian coordinates (Angstrom):
C 7.585778 1.448965 -0.977758
C 7.341716 2.819294 -0.830152
C 6.059425 3.282163 -0.546137
C 5.025935 2.355828 -0.414454
C 5.272575 0.989326 -0.573132
H 8.161205 3.530854 -0.933769
H 5.866733 4.349026 -0.425449
C 2.999405 0.819525 -0.083601
C 1.779967 -1.293886 0.100646
C 2.792260 -1.774365 -0.952630
C 4.182603 -1.266435 -0.613334
H 4.872066 -1.460049 -1.445206
N 4.132752 0.183181 -0.411539
N 1.869098 0.178484 0.205236
C 2.754049 -3.299324 -1.205044
C 3.689458 -4.115538 -0.307437
C 3.052516 -3.594165 -2.676426
H 1.719710 -3.624398 -1.005854
H 4.743622 -3.934097 -0.569781
H 3.557186 -3.896660 0.761266
H 3.499277 -5.187642 -0.456993
H 2.321870 -3.107983 -3.337972
H 4.056497 -3.235582 -2.954019
H 3.022605 -4.676081 -2.867756
H 2.482991 -1.278810 -1.887820
H 0.781494 -1.487457 -0.313777
C 1.912225 -1.963200 1.451717
C 1.101695 -3.065496 1.744254
C 2.856124 -1.543106 2.394327
C 1.256950 -3.760494 2.942234
H 0.341166 -3.381589 1.025226
C 3.011585 -2.236954 3.594569
H 3.473055 -0.661067 2.203276
C 2.218743 -3.351916 3.866968
H 0.620077 -4.619633 3.157095
H 3.752151 -1.900917 4.321609
H 2.340542 -3.893340 4.806063
C -0.421557 0.254192 1.058261
H -0.382600 -0.815517 1.227249
C -1.655956 0.926778 1.079784
O 0.745670 2.168209 0.325613
C 0.683560 0.953419 0.550156
H 4.588893 -1.741089 0.293086
C 6.558405 0.518394 -0.850671
H 6.764218 -0.546112 -0.955155
H 8.595693 1.098509 -1.192060
H -1.621894 2.012164 1.201837
C -1.994861 -0.122910 -1.405399
O -0.988152 -0.817279 -1.561196
C -2.121872 1.246454 -0.969093
C -3.534184 1.509070 -0.993738
H -1.319552 1.970839 -1.096156
N -4.225043 0.427919 -1.248042
N -3.325705 -0.569660 -1.494893
C -3.783219 -1.884627 -1.680866
C -5.111174 -2.201606 -1.352369
C -2.936593 -2.887762 -2.179171
C -5.580184 -3.500576 -1.522988
H -5.754587 -1.417093 -0.959257
C -3.423465 -4.185233 -2.335801
H -1.910533 -2.641491 -2.437065
C -4.741675 -4.503777 -2.012433
H -6.615164 -3.730978 -1.263737
H -2.756288 -4.956197 -2.725781
H -5.113017 -5.521149 -2.141721
C -4.190936 2.778119 -0.642871
C -3.423187 3.907997 -0.329851
C -5.589780 2.866376 -0.575506
C -4.040574 5.101659 0.045094
H -2.333503 3.854965 -0.376908
C -6.203133 4.058115 -0.203484
H -6.182420 1.982181 -0.812653
C -5.431096 5.180887 0.109610
H -3.430564 5.973302 0.287258
H -7.291850 4.114427 -0.153454

H -5.914256 6.114055 0.403214
C -2.872269 0.277887 1.615679
C -3.069313 -1.107288 1.509581
C -3.883147 1.058930 2.193293
C -4.240193 -1.696026 1.980105
H -2.311080 -1.725662 1.022599
C -5.053790 0.470550 2.668444
H -3.749007 2.141182 2.261198
C -5.235319 -0.908778 2.563148
H -4.382188 -2.773197 1.878298
H -5.829982 1.093063 3.116474
H -6.153800 -1.370027 2.929791
Se 3.196718 2.695280 -0.042794
----
top_face_R_2-lact
Frequencies, energies and thermodynamic properties:
Lowest Vibrational Mode (1/cm) = 11.3239
2nd Lowest Vibrational Mode (1/cm) = 17.8116
E(RM062X) (a.u.) = -4429.92469933
Thermal correction to Enthalpy (a.u.) = 0.764332
Thermal correction to Gibbs Free Energy (a.u.) = 0.645858
Total Entropy (cal/Kmol) = 249.349
Esp(RM062X) (a.u.) = -4432.43316156
Optimised cartesian coordinates (Angstrom):
C -7.161192 0.574356 -1.772640
C -6.754184 0.957159 -3.054591
C -5.400057 0.989957 -3.384862
C -4.458896 0.640319 -2.418377
C -4.873477 0.270528 -1.136070
H -7.499511 1.227700 -3.803132
H -5.081868 1.283470 -4.386648
C -2.578366 -0.003666 -0.795429
C -1.576517 -0.762991 1.282978
C -2.775618 -0.055933 1.941369
C -4.040887 -0.341879 1.147594
H -4.858808 0.302283 1.496886
N -3.813605 -0.043072 -0.266129
N -1.501117 -0.345496 -0.122678
C -2.932342 -0.346398 3.451505
C -3.806716 -1.560878 3.778756
C -3.467886 0.892745 4.171989
H -1.919426 -0.542816 3.840364
H -4.863456 -1.359227 3.543111
H -3.499334 -2.467025 3.238281
H -3.750357 -1.775611 4.855218
H -2.796981 1.753473 4.040740
H -4.461261 1.172759 3.786928
H -3.572302 0.699894 5.249041
H -2.559329 1.019873 1.831032
H -0.671316 -0.363201 1.759376
C -1.584900 -2.272129 1.427376
C -0.862918 -2.865373 2.468167
C -2.336370 -3.085385 0.572466
C -0.918274 -4.242608 2.674907
H -0.248834 -2.242332 3.123221
C -2.391688 -4.464236 0.777109
H -2.878642 -2.648997 -0.270074
C -1.689554 -5.044859 1.833334
H -0.351051 -4.691318 3.491503
H -2.982441 -5.087227 0.104172
H -1.732364 -6.123248 1.991824
C 0.822766 -1.270178 -0.428578
H 0.430949 -2.188772 -0.887981
C 2.222350 -0.945493 -0.988329
O -0.231836 0.174303 -1.997417
C -0.140873 -0.123433 -0.773962
H -4.361472 -1.390279 1.248722
C -6.227578 0.225254 -0.798730
H -6.556527 -0.082931 0.193173
H -8.223008 0.543562 -1.526527
H 2.132448 -0.946460 -2.087213
C 1.606791 1.286443 -0.009324
O 0.305264 1.076790 0.100457
C 2.576389 0.438074 -0.510484
C 3.782564 1.182282 -0.353582
N 3.555494 2.361812 0.204490
N 2.221530 2.434157 0.404330
C 1.651254 3.573922 1.020313
C 2.460775 4.379099 1.829789
C 0.306605 3.906001 0.816941
C 1.920489 5.508724 2.438291
H 3.506621 4.108492 1.964571
C -0.220861 5.034674 1.442487
H -0.313113 3.286953 0.172099
C 0.577435 5.840245 2.253944
H 2.557318 6.132900 3.067091
H -1.269149 5.290439 1.280380
H 0.157442 6.723905 2.735854
C 5.157904 0.788282 -0.716676
C 5.395800 -0.070631 -1.797684
C 6.248538 1.271749 0.019047
C 6.694982 -0.454037 -2.124424
H 4.555697 -0.435480 -2.390551
C 7.547654 0.893326 -0.312406
H 6.060969 1.942130 0.858921

C 7.774875 0.024450 -1.381349
H 6.864653 -1.126839 -2.966669
H 8.388154 1.272464 0.271359
H 8.792060 -0.277560 -1.635813
C 3.206822 -2.027779 -0.587382
C 3.809211 -2.029229 0.675900
C 3.507481 -3.065256 -1.475013
C 4.702945 -3.035961 1.036677
H 3.589896 -1.217208 1.373843
C 4.400892 -4.075815 -1.117745
H 3.045029 -3.071652 -2.465506
C 5.004812 -4.061892 0.139204
H 5.172656 -3.016481 2.021620
H 4.630717 -4.872939 -1.826884
H 5.710395 -4.845923 0.418528
Se -2.557333 0.581079 -2.597239
H 0.900269 -1.433281 0.655809

----

top_face_R_2-tet-pt
Frequencies, energies and thermodynamic properties:
Lowest Vibrational Mode (1/cm) = 14.3700
2nd Lowest Vibrational Mode (1/cm) = 16.5972
E(RM062X) (a.u.) = -4429.92297393
Thermal correction to Enthalpy (a.u.) = 0.764207
Thermal correction to Gibbs Free Energy (a.u.) = 0.644108
Total Entropy (cal/Kmol) = 252.770
Esp(RM062X) (a.u.) = -4432.43291182
Optimised cartesian coordinates (Angstrom):
C -7.173669 0.856470 -1.835653
C -6.797604 0.937330 -3.182515
C -5.474787 0.731907 -3.561976
C -4.533651 0.448002 -2.572327
C -4.910258 0.380600 -1.228508
H -7.547160 1.159154 -3.942360
H -5.180542 0.789096 -4.610586
C -2.658932 -0.126422 -0.922591
C -1.640707 -0.625474 1.258567
C -2.709300 0.345777 1.779445
C -4.038794 0.077465 1.099828
H -4.759994 0.867717 1.344223
N -3.848498 0.092439 -0.353872
N -1.585195 -0.514073 -0.217045
C -2.816813 0.381781 3.320829
C -3.809854 -0.624865 3.909987
C -3.159841 1.798206 3.786729
H -1.814587 0.139820 3.710691
H -4.847210 -0.344981 3.667578
H -3.637539 -1.651204 3.556407
H -3.723905 -0.628302 5.005601
H -2.392352 2.518370 3.469528
H -4.127654 2.125459 3.374630
H -3.235483 1.837325 4.882439
H -2.364065 1.338581 1.446419
H -0.684146 -0.215071 1.600130
C -1.802771 -2.062723 1.699843
C -1.092107 -2.517194 2.815893
C -2.679463 -2.937057 1.048497
C -1.282004 -3.811548 3.296214
H -0.383711 -1.850440 3.313741
C -2.868600 -4.233779 1.526750
H -3.217220 -2.614702 0.152993
C -2.176506 -4.670407 2.656286
H -0.723433 -4.153044 4.168592
H -3.556160 -4.905864 1.011531
H -2.323973 -5.684564 3.029791
C 0.752327 -1.464118 -0.189281
H 0.546111 -2.531806 -0.381689
C 2.144082 -1.113467 -0.749757
O -0.281232 -0.440508 -2.071901
C -0.343332 -0.713155 -0.896873
H -4.469255 -0.891908 1.393089
C -6.239529 0.577390 -0.843835
H -6.547814 0.504596 0.198214
H -8.215645 1.010968 -1.554827
H 2.080225 -1.276909 -1.839376
C 1.651831 1.281491 0.125681
O 0.425593 1.229439 0.427865
C 2.513785 0.315771 -0.444190
C 3.773374 0.965745 -0.550017
N 3.741453 2.205186 -0.091615
N 2.462632 2.413154 0.316325
C 2.113570 3.651595 0.880071
C 3.121952 4.603718 1.110567
C 0.786011 3.968586 1.217223
C 2.807114 5.838289 1.669818
H 4.145040 4.352860 0.840119
C 0.491881 5.210455 1.778944
H 0.008113 3.234236 1.030607
C 1.491291 6.154142 2.012478
H 3.605649 6.562917 1.840532
H -0.544590 5.440500 2.034564
H 1.249457 7.122691 2.452041
C 5.048422 0.430966 -1.075293
C 5.073734 -0.508941 -2.114516
C 6.267816 0.871971 -0.540216

C 6.283956 -1.006157 -2.595843
H 4.136042 -0.846046 -2.558469
C 7.477578 0.378917 -1.023684
H 6.248173 1.604717 0.267310
C 7.490682 -0.566907 -2.050720
H 6.283811 -1.738534 -3.405132
H 8.416934 0.729197 -0.591997
H 8.437727 -0.958595 -2.425537
C 3.133232 -2.134468 -0.201060
C 3.750938 -1.956475 1.040829
C 3.412179 -3.296693 -0.927725
C 4.635140 -2.912942 1.539062
H 3.549699 -1.044141 1.607146
C 4.296970 -4.255463 -0.434449
H 2.939608 -3.443625 -1.903082
C 4.914283 -4.064620 0.801925
H 5.115519 -2.754622 2.506292
H 4.511233 -5.150390 -1.021371
H 5.613666 -4.808091 1.187690
Se -2.682863 0.122585 -2.787739
H 0.737392 -1.323837 0.894751
----
top_face_R_2-tet
Frequencies, energies and thermodynamic properties:
Lowest Vibrational Mode (1/cm) = 17.8046
2nd Lowest Vibrational Mode (1/cm) = 21.2889
E(RM062X) (a.u.) = -4429.91157171
Thermal correction to Enthalpy (a.u.) = 0.763153
Thermal correction to Gibbs Free Energy (a.u.) = 0.645601
Total Entropy (cal/Kmol) = 247.408
Esp(RM062X) (a.u.) = -4432.42114850
Optimised cartesian coordinates (Angstrom):
C 6.936249 0.302684 -1.015088
C 7.028507 -1.042519 -1.387005
C 5.881852 -1.830000 -1.474205
C 4.644796 -1.258224 -1.181451
C 4.561385 0.083327 -0.797642
H 8.001849 -1.477783 -1.615296
H 5.950592 -2.878292 -1.769675
C 2.274597 -0.388263 -0.718037
C 0.545813 1.208847 -0.097591
C 1.669395 1.873370 0.717733
C 2.965133 1.875019 -0.073449
H 3.796450 2.193870 0.568471
N 3.250892 0.516787 -0.531374
N 0.990667 -0.112442 -0.570318
C 1.296075 3.270718 1.264319
C 1.606659 4.431726 0.313634
C 1.978952 3.509624 2.612112
H 0.207408 3.257181 1.442845
H 2.693922 4.579838 0.218861
H 1.189685 4.288578 -0.692538
H 1.188668 5.362669 0.722203
H 1.672920 2.757634 3.351551
H 3.075371 3.461426 2.512650
H 1.725202 4.505103 3.003416
H 1.824684 1.207550 1.584742
H -0.268591 1.009968 0.614083
C 0.003260 2.046354 -1.237307
C -1.153655 2.806997 -1.040061
C 0.656914 2.109850 -2.471788
C -1.630548 3.646601 -2.045374
H -1.691270 2.734084 -0.091010
C 0.182234 2.950457 -3.478642
H 1.538155 1.491421 -2.660871
C -0.957167 3.726845 -3.264981
H -2.534776 4.233540 -1.877642
H 0.701558 2.993312 -4.437005
H -1.328566 4.382101 -4.054059
C -1.322895 -0.850838 -0.722998
H -1.624092 0.185358 -0.608758
C -2.374468 -1.897590 -0.514334
O 0.519964 -2.333657 -0.940097
C -0.002294 -1.200896 -0.775496
H 2.921239 2.549363 -0.942994
C 5.704635 0.882207 -0.718092
H 5.646201 1.934536 -0.442812
H 7.837846 0.913332 -0.958586
H -1.937280 -2.883033 -0.741388
C -1.430321 -2.407661 1.714470
O -0.882511 -3.480910 1.677552
C -2.717666 -1.976720 1.035176
C -2.907880 -0.612285 1.641458
H -3.531970 -2.689288 1.225145
N -1.885453 -0.240895 2.330891
N -0.966695 -1.265274 2.353020
C 0.341880 -1.022527 2.819902
C 0.555688 -0.092148 3.842754
C 1.426980 -1.672540 2.215960
C 1.856704 0.179327 4.264070
H -0.299482 0.411994 4.292299
C 2.721759 -1.380752 2.641601
H 1.248906 -2.374180 1.399491
C 2.944908 -0.457876 3.665834
H 2.019365 0.899111 5.068053

H 3.564316 -1.878582 2.156089
H 3.960513 -0.237751 3.997013
C -4.050238 0.291700 1.428733
C -5.284222 -0.203098 0.988209
C -3.909658 1.668786 1.664405
C -6.355517 0.663688 0.777173
H -5.408965 -1.270244 0.799520
C -4.979244 2.531908 1.449806
H -2.948763 2.048382 2.015998
C -6.205274 2.031393 1.002455
H -7.310044 0.265908 0.429549
H -4.858123 3.601299 1.629192
H -7.042327 2.709675 0.830249
C -3.645242 -1.738302 -1.331591
C -3.934046 -0.570874 -2.043723
C -4.580959 -2.782242 -1.353533
C -5.141513 -0.436618 -2.732461
H -3.210416 0.245659 -2.059558
C -5.785863 -2.653163 -2.040110
H -4.361329 -3.710356 -0.818180
C -6.074553 -1.471513 -2.727347
H -5.352492 0.486460 -3.275304
H -6.500633 -3.477931 -2.044002
H -7.018543 -1.364863 -3.264198
Se 2.929129 -2.091366 -1.217286
----
top_face_R_2-TS1
Frequencies, energies and thermodynamic properties:
Lowest Vibrational Mode (1/cm) = -351.5642
2nd Lowest Vibrational Mode (1/cm) = 16.0417
E(RM062X) (a.u.) = -4429.89345664
Thermal correction to Enthalpy (a.u.) = 0.760837
Thermal correction to Gibbs Free Energy (a.u.) = 0.643308
Total Entropy (cal/Kmol) = 247.361
Esp(RM062X) (a.u.) = -4432.40465779
Esp(RM062X) gas (a.u.) = -4432.37894730
Esp(RM062X) gas nuc (a.u.) = -762.962789748
Esp(RM062X) gas cat (a.u.) = -3669.27008723
Optimised cartesian coordinates (Angstrom):
C 6.983793 0.470928 -0.700480
C 7.087217 -0.759026 -1.361033
C 5.945458 -1.494386 -1.667330
C 4.700290 -0.984988 -1.299746
C 4.603295 0.236070 -0.627062
H 8.067896 -1.143037 -1.642910
H 6.022766 -2.450211 -2.187340
C 2.318509 -0.216954 -0.737835
C 0.567137 1.246357 0.151169
C 1.662038 1.686705 1.136598
C 2.989787 1.840911 0.418860
H 3.795954 1.990212 1.148391
N 3.284123 0.612193 -0.320210
N 1.020445 0.043188 -0.576184
C 1.278933 2.935191 1.965302
C 1.659408 4.270739 1.317599
C 1.887245 2.845017 3.365674
H 0.182263 2.911196 2.079441
H 2.752230 4.407735 1.314733
H 1.297116 4.368045 0.284942
H 1.232310 5.097069 1.903143
H 1.516279 1.962290 3.902856
H 2.985952 2.776136 3.315638
H 1.636880 3.739066 3.954076
H 1.774850 0.841626 1.836268
H -0.284780 0.913288 0.762988
C 0.103857 2.327248 -0.803414
C -1.042948 3.065785 -0.492813
C 0.818011 2.635656 -1.965292
C -1.449718 4.119909 -1.308848
H -1.626733 2.807138 0.394430
C 0.412669 3.690873 -2.782905
H 1.693121 2.045069 -2.248712
C -0.716404 4.440375 -2.452072
H -2.345498 4.688099 -1.054261
H 0.978882 3.923633 -3.685732
H -1.033191 5.264133 -3.093058
C -1.314554 -0.538865 -1.038805
H -1.598051 0.477589 -0.792359
C -2.302953 -1.519834 -1.219179
O 0.503117 -1.992568 -1.466822
C 0.041001 -0.922689 -1.063685
H 2.993479 2.689696 -0.282370
C 5.745122 0.984105 -0.327404
H 5.679342 1.948428 0.174363
H 7.884569 1.042190 -0.474817
H -1.942424 -2.503382 -1.535153
C -1.525472 -2.833606 1.068289
O -0.966819 -3.896747 0.828336
C -2.837964 -2.327276 0.701965
C -2.972272 -1.088628 1.417696
H -3.643709 -2.991691 0.401997
N -1.842310 -0.723106 1.965868
N -0.954386 -1.751528 1.754435
C 0.337728 -1.669399 2.296757
C 0.560477 -0.915773 3.458071

C 1.411625 -2.314841 1.666645
C 1.849270 -0.807951 3.977846
H -0.285340 -0.422252 3.935867
C 2.697689 -2.177704 2.186431
H 1.222386 -2.898235 0.766612
C 2.926736 -1.426343 3.341293
H 2.012584 -0.231421 4.890190
H 3.531358 -2.666795 1.677406
H 3.934840 -1.332105 3.746946
C -4.148805 -0.203104 1.432411
C -5.412946 -0.694468 1.082176
C -4.014905 1.152217 1.768547
C -6.517709 0.153744 1.052943
H -5.529746 -1.746508 0.816271
C -5.119084 1.999894 1.735392
H -3.030028 1.527263 2.052316
C -6.374129 1.504076 1.373241
H -7.494863 -0.241174 0.770062
H -5.001928 3.054350 1.991532
H -7.238229 2.169750 1.343656
C -3.664145 -1.164828 -1.700597
C -4.080450 0.167481 -1.836164
C -4.563374 -2.182963 -2.051280
C -5.365550 0.469618 -2.281897
H -3.396854 0.982510 -1.594586
C -5.847748 -1.883320 -2.497673
H -4.246849 -3.225594 -1.965022
C -6.256219 -0.552659 -2.608607
H -5.672719 1.512842 -2.372765
H -6.531846 -2.690658 -2.764010
H -7.262905 -0.314683 -2.955931
Se 2.993102 -1.759178 -1.588803
----
top_face_R_2-TS2
Frequencies, energies and thermodynamic properties:
Lowest Vibrational Mode (1/cm) = -128.1512
2nd Lowest Vibrational Mode (1/cm) = 11.8008
E(RM062X) (a.u.) = -4429.92119233
Thermal correction to Enthalpy (a.u.) = 0.763112
Thermal correction to Gibbs Free Energy (a.u.) = 0.644539
Total Entropy (cal/Kmol) = 249.557
Esp(RM062X) (a.u.) = -4432.43028164
Esp(RM062X) gas (a.u.) = -4432.43028164
Esp(RM062X) gas nuc (a.u.) = -762.118649933
Esp(RM062X) gas cat (a.u.) = -3669.94677517
Optimised cartesian coordinates (Angstrom):
C 7.175468 -0.835892 -1.662081
C 6.795252 -1.173780 -2.966071
C 5.458565 -1.111249 -3.351443
C 4.508718 -0.710068 -2.413064
C 4.891512 -0.385897 -1.108887
H 7.550530 -1.484026 -3.688505
H 5.160374 -1.368512 -4.368721
C 2.612861 0.044062 -0.857925
C 1.579315 0.838826 1.209916
C 2.706524 0.039960 1.883296
C 4.014504 0.266491 1.145967
H 4.784177 -0.424198 1.513794
N 3.821087 -0.008475 -0.279473
N 1.531005 0.475984 -0.218526
C 2.821828 0.280209 3.405797
C 3.753973 1.430868 3.797445
C 3.253255 -1.008640 4.108560
H 1.808341 0.527037 3.762606
H 4.804622 1.175254 3.588392
H 3.516813 2.369218 3.276819
H 3.675061 1.616704 4.877768
H 2.535214 -1.821286 3.929103
H 4.241130 -1.340862 3.751854
H 3.328749 -0.850614 5.193586
H 2.425323 -1.015727 1.733343
H 0.642402 0.455030 1.629746
C 1.657000 2.338685 1.401109
C 0.926808 2.934865 2.434576
C 2.476929 3.138854 0.598018
C 1.041832 4.300706 2.686543
H 0.260494 2.323328 3.048017
C 2.591295 4.506584 0.847766
H 3.027439 2.701927 -0.239202
C 1.880761 5.088546 1.897546
H 0.468173 4.752366 3.496913
H 3.234777 5.119871 0.215563
H 1.969833 6.158215 2.091688
C -0.825122 1.372379 -0.396470
H -0.555709 2.369917 -0.781621
C -2.215993 0.974910 -0.927280
O 0.239254 0.038926 -2.056160
C 0.236968 0.415787 -0.896252
H 4.384954 1.295871 1.266770
C 6.232161 -0.437497 -0.719153
H 6.542587 -0.164666 0.288752
H 8.226486 -0.880078 -1.375593
H -2.150174 1.011753 -2.028455
C -1.584914 -1.287012 0.078064
O -0.323870 -1.114643 0.247464

C -2.541612 -0.421712 -0.468342
C -3.762412 -1.148664 -0.394013
N -3.596291 -2.340611 0.155468
N -2.273084 -2.438658 0.435044
C -1.774500 -3.598592 1.060184
C -2.680117 -4.507014 1.628726
C -0.398341 -3.867870 1.115327
C -2.211985 -5.661680 2.248992
H -3.744555 -4.289879 1.568268
C 0.051948 -5.026680 1.746449
H 0.298349 -3.167960 0.662039
C -0.843954 -5.929710 2.317675
H -2.928693 -6.358875 2.686703
H 1.124585 -5.226291 1.782510
H -0.481520 -6.834451 2.807314
C -5.112181 -0.728913 -0.825005
C -5.288778 0.123512 -1.923001
C -6.245026 -1.183509 -0.135064
C -6.564982 0.528172 -2.310378
H -4.417175 0.467332 -2.482133
C -7.521277 -0.784851 -0.526442
H -6.106157 -1.848171 0.718637
C -7.685850 0.077593 -1.611994
H -6.684602 1.195599 -3.165577
H -8.393430 -1.142547 0.023702
H -8.684986 0.396092 -1.913707
C -3.217260 2.032557 -0.490506
C -3.821033 1.985931 0.770638
C -3.526703 3.096778 -1.343167
C -4.724842 2.972282 1.163220
H -3.595163 1.150851 1.438188
C -4.431245 4.085373 -0.955157
H -3.063719 3.140379 -2.332819
C -5.036387 4.024072 0.299983
H -5.195507 2.915704 2.146341
H -4.669451 4.902298 -1.638631
H -5.750873 4.790857 0.603623
Se 2.636660 -0.523497 -2.657245
H -0.854977 1.436864 0.696613
----
top_face_R_2-TS3
Frequencies, energies and thermodynamic properties:
Lowest Vibrational Mode (1/cm) = -136.5541
2nd Lowest Vibrational Mode (1/cm) = 21.1268
E(RM062X) (a.u.) = -4429.91974647
Thermal correction to Enthalpy (a.u.) = 0.762667
Thermal correction to Gibbs Free Energy (a.u.) = 0.648641
Total Entropy (cal/Kmol) = 239.988
Esp(RM062X) (a.u.) = -4432.42703319
Optimised cartesian coordinates (Angstrom):
C -6.940338 -1.576761 1.432101
C -7.040568 -2.606665 0.493898
C -6.015232 -2.821853 -0.428196
C -4.895187 -1.996415 -0.397132
C -4.802685 -0.957592 0.537640
H -7.920921 -3.249779 0.481809
H -6.089247 -3.627427 -1.160359
C -2.703867 -0.620676 -0.464849
C -1.088872 0.977420 0.278034
C -2.313549 1.833361 0.651630
C -3.378431 0.958901 1.296939
H -4.323244 1.513476 1.386296
N -3.622545 -0.208627 0.449720
N -1.516722 -0.124707 -0.582667
C -1.963323 3.103389 1.456475
C -1.932140 2.904464 2.974482
C -2.921682 4.240606 1.096724
H -0.953794 3.409303 1.125137
H -2.946402 2.729931 3.367299
H -1.295302 2.063459 3.281980
H -1.548501 3.813229 3.459677
H -2.881655 4.468428 0.021848
H -3.960179 3.978198 1.355257
H -2.662223 5.155463 1.648920
H -2.717864 2.167430 -0.319160
H -0.462728 1.629401 -0.337324
C -0.248736 0.507269 1.451647
C 0.924184 1.198379 1.774887
C -0.628498 -0.587475 2.238301
C 1.683071 0.829904 2.886990
H 1.248970 2.033798 1.149263
C 0.132541 -0.961851 3.345088
H -1.516760 -1.169382 1.977731
C 1.284653 -0.246975 3.679016
H 2.593318 1.381125 3.127974
H -0.173662 -1.818218 3.947515
H 1.878273 -0.539565 4.546364
C 0.468821 -1.721662 -1.048058
H 0.071336 -2.731045 -1.217649
C 1.970277 -1.664231 -1.417087
O -1.166858 -1.230041 -2.748524
C -0.461819 -0.780534 -1.844311
H -3.080392 0.627038 2.304476
C -5.822736 -0.742778 1.465968
H -5.749613 0.050389 2.209406

H -7.742656 -1.419182 2.153623
H 2.110148 -2.054207 -2.438678
C 1.397439 0.723161 -1.703913
O 0.179485 0.499482 -2.189546
C 2.350354 -0.206910 -1.361206
C 3.424596 0.589078 -0.865104
N 3.124742 1.880199 -0.902753
N 1.879722 1.968676 -1.417040
C 1.190248 3.206894 -1.426408
C 1.622218 4.222528 -0.564698
C 0.065347 3.400589 -2.237572
C 0.914783 5.420445 -0.502600
H 2.507804 4.055149 0.046311
C -0.638151 4.601515 -2.152136
H -0.258719 2.618124 -2.920238
C -0.223897 5.613056 -1.285885
H 1.255869 6.207175 0.171851
H -1.518005 4.745863 -2.781079
H -0.781338 6.548546 -1.226322
C 4.737569 0.150438 -0.351410
C 5.412103 -0.925726 -0.941430
C 5.320985 0.805889 0.740206
C 6.638736 -1.352185 -0.437119
H 4.969493 -1.429377 -1.802546
C 6.549554 0.380616 1.242664
H 4.793770 1.645820 1.195481
C 7.208408 -0.702878 0.659496
H 7.152618 -2.194586 -0.902993
H 6.992631 0.893312 2.098005
H 8.167114 -1.039202 1.057452
C 2.739961 -2.546153 -0.454165
C 2.835120 -2.186744 0.896387
C 3.342606 -3.730539 -0.880972
C 3.537361 -2.983901 1.795523
H 2.369337 -1.258037 1.240201
C 4.046854 -4.534613 0.018663
H 3.271681 -4.021293 -1.931954
C 4.150209 -4.161226 1.357529
H 3.610503 -2.682673 2.842375
H 4.518621 -5.454522 -0.330945
H 4.704973 -4.785369 2.059906
Se -3.375306 -2.053858 -1.537421
H 0.400002 -1.503292 0.022939
----
top_face_R_3-TS1
Frequencies, energies and thermodynamic properties:
Lowest Vibrational Mode (1/cm) = -314.0079
2nd Lowest Vibrational Mode (1/cm) = 17.2804
E(RM062X) (a.u.) = -4429.88062506
Thermal correction to Enthalpy (a.u.) = 0.761113
Thermal correction to Gibbs Free Energy (a.u.) = 0.642869
Total Entropy (cal/Kmol) = 248.864
Esp(RM062X) (a.u.) = -4432.39285475
Optimised cartesian coordinates (Angstrom):
C 5.957629 -3.598827 -1.125845
C 5.070611 -4.567792 -1.610262
C 3.703211 -4.312230 -1.658483
C 3.234430 -3.077976 -1.208139
C 4.122383 -2.119890 -0.712585
H 5.452788 -5.527787 -1.958161
H 3.010646 -5.061880 -2.043778
C 2.169192 -0.870739 -0.455639
C 2.086929 1.406959 0.443436
C 3.368072 0.989640 1.183593
C 4.265986 0.179934 0.268676
H 5.099191 -0.245402 0.841499
N 3.497944 -0.931602 -0.294117
N 1.448736 0.210400 -0.141636
C 4.104753 2.159966 1.873159
C 5.148955 2.857657 0.995869
C 4.751600 1.672933 3.171703
H 3.336233 2.902685 2.144595
H 6.011407 2.197815 0.812583
H 4.746910 3.178004 0.024638
H 5.528594 3.749548 1.513814
H 4.002130 1.266573 3.866103
H 5.492482 0.882795 2.970983
H 5.275190 2.496568 3.676966
H 3.026252 0.304293 1.973815
H 1.393399 1.765415 1.215886
C 2.286563 2.492445 -0.593972
C 2.063306 3.827645 -0.242212
C 2.743758 2.194133 -1.881373
C 2.327927 4.852051 -1.149185
H 1.677871 4.068675 0.752005
C 3.008619 3.218556 -2.790269
H 2.886874 1.155661 -2.191282
C 2.809634 4.549417 -2.423281
H 2.151035 5.889456 -0.862350
H 3.366158 2.974071 -3.791326
H 3.015695 5.349743 -3.135113
C -0.708098 1.393963 -0.199965
H -0.220730 2.303027 0.133857
C -2.091244 1.372916 -0.418670
O -0.427225 -0.842851 -0.898838

C 0.021972 0.209040 -0.442288
H 4.681419 0.781673 -0.554397
C 5.497986 -2.365476 -0.674341
H 6.202144 -1.616912 -0.313899
H 7.027602 -3.806852 -1.103139
H -2.470942 0.499288 -0.955105
C -4.404001 0.351593 0.929662
O -5.381202 1.058569 0.694462
C -3.091519 0.676536 1.437512
C -2.443552 -0.593353 1.617913
H -2.891811 1.595146 1.980954
N -3.152000 -1.579474 1.130651
N -4.320377 -1.037957 0.676084
C -5.298346 -1.879006 0.118181
C -5.080821 -3.267052 0.098563
C -6.484609 -1.362530 -0.430950
C -6.032187 -4.116562 -0.457869
H -4.159207 -3.658759 0.522835
C -7.425164 -2.231252 -0.982494
H -6.655372 -0.290271 -0.412098
C -7.212817 -3.609057 -1.002338
H -5.845193 -5.192049 -0.463538
H -8.341672 -1.814836 -1.404824
H -7.956067 -4.278957 -1.436418
C -1.113239 -0.825729 2.203900
C -0.464875 0.189622 2.920426
C -0.462066 -2.056962 2.035571
C 0.796197 -0.027992 3.476642
H -0.956593 1.154565 3.053571
C 0.800354 -2.270525 2.580877
H -0.959014 -2.833777 1.453429
C 1.432673 -1.258748 3.309879
H 1.282010 0.768955 4.044054
H 1.297706 -3.231544 2.436299
H 2.418805 -1.430766 3.746215
C -2.869263 2.614739 -0.593495
C -2.477562 3.829201 -0.004999
C -4.024669 2.594325 -1.386027
C -3.216784 4.988368 -0.213045
H -1.590679 3.865512 0.631165
C -4.759436 3.759297 -1.604967
H -4.344679 1.653249 -1.836111
C -4.359216 4.957971 -1.018839
H -2.902398 5.923245 0.253596
H -5.652656 3.726517 -2.230495
H -4.936181 5.869209 -1.184162
Se 1.444950 -2.448544 -1.189374
----
top_face_S_1-TS1
Frequencies, energies and thermodynamic properties:
Lowest Vibrational Mode (1/cm) = -348.5398
2nd Lowest Vibrational Mode (1/cm) = 11.5869
E(RM062X) (a.u.) = -4429.89034928
Thermal correction to Enthalpy (a.u.) = 0.761009
Thermal correction to Gibbs Free Energy (a.u.) = 0.642646
Total Entropy (cal/Kmol) = 249.115
Esp(RM062X) (a.u.) = -4432.40198844
Esp(RM062X) gas (a.u.) = -4432.37713081
Esp(RM062X) gas nuc (a.u.) = -762.963452929
Esp(RM062X) gas cat (a.u.) = -3669.26867753
Optimised cartesian coordinates (Angstrom):
C -6.841295 0.487507 0.406398
C -6.965237 -0.875185 0.701350
C -5.832393 -1.669284 0.860743
C -4.574938 -1.084234 0.716205
C -4.457899 0.273993 0.408943
H -7.955057 -1.318565 0.812641
H -5.925846 -2.730218 1.096848
C -2.177073 -0.195253 0.538518
C -0.401834 1.449373 0.183494
C -1.430228 2.107434 -0.750988
C -2.805959 2.086069 -0.109207
H -3.566838 2.408307 -0.831433
N -3.129248 0.716172 0.291449
N -0.879197 0.100271 0.543332
C -1.024422 3.510507 -1.252699
C -1.427919 4.664465 -0.328307
C -1.604214 3.750571 -2.648178
H 0.075725 3.502490 -1.346862
H -2.520328 4.804978 -0.331669
H -1.102959 4.522446 0.710966
H -0.981411 5.599675 -0.694971
H -1.306565 2.957903 -3.348211
H -2.705110 3.784952 -2.616798
H -1.253892 4.712003 -3.050964
H -1.470204 1.445512 -1.633254
H 0.501855 1.288216 -0.418527
C -0.042966 2.261499 1.409589
C 1.082981 3.091268 1.370945
C -0.836273 2.239944 2.560350
C 1.391650 3.912841 2.453672
H 1.717873 3.095169 0.480388
C -0.528305 3.062660 3.644226
H -1.698552 1.571180 2.624075
C 0.581403 3.905900 3.590153

H 2.270261 4.558030 2.411093
H -1.155557 3.039490 4.536364
H 0.822219 4.547813 4.438589
C 1.427722 -0.592020 1.020720
H 1.733924 0.448755 1.024024
C 2.387573 -1.625389 1.018086
O -0.396547 -2.092124 0.955294
C 0.081096 -0.957257 0.853546
H -2.861368 2.743931 0.771944
C -5.589987 1.079032 0.258066
H -5.506761 2.143644 0.043011
H -7.735458 1.101122 0.293846
H 1.970597 -2.629344 1.138325
C 3.474916 -0.753053 -1.283508
O 4.590454 -0.287750 -1.103442
C 2.907456 -2.057762 -0.977439
C 1.607577 -2.045014 -1.593292
H 3.537979 -2.931929 -0.833931
N 1.279930 -0.841947 -1.991553
N 2.375085 -0.043659 -1.797075
C 2.365865 1.285925 -2.246283
C 1.502612 1.668315 -3.283608
C 3.211767 2.238506 -1.657098
C 1.503144 2.986307 -3.734975
H 0.852936 0.917627 -3.732392
C 3.189366 3.555622 -2.112953
H 3.883866 1.930882 -0.858290
C 2.340494 3.938695 -3.152569
H 0.841482 3.271161 -4.554620
H 3.848723 4.290345 -1.647521
H 2.335512 4.969291 -3.509500
C 0.644636 -3.157311 -1.671666
C -0.631138 -2.955076 -2.217244
C 0.981702 -4.426437 -1.186852
C -1.549912 -3.997845 -2.267583
H -0.892309 -1.963447 -2.589603
C 0.059122 -5.470950 -1.236076
H 1.970826 -4.598975 -0.758114
C -1.210016 -5.260497 -1.774025
H -2.542016 -3.826186 -2.689224
H 0.333719 -6.453939 -0.850130
H -1.932264 -6.077514 -1.811424
C 3.737359 -1.462075 1.609480
C 4.277786 -0.204641 1.910909
C 4.504644 -2.603229 1.887214
C 5.546196 -0.094154 2.478268
H 3.700905 0.699647 1.710310
C 5.772080 -2.495750 2.452418
H 4.093076 -3.589446 1.656711
C 6.297980 -1.236941 2.749789
H 5.950401 0.892800 2.708713
H 6.350770 -3.395895 2.665421
H 7.290711 -1.148021 3.193760
Se -2.874083 -1.914212 0.884157
----
top_face_S_2-TS1
Frequencies, energies and thermodynamic properties:
Lowest Vibrational Mode (1/cm) = -416.9644
2nd Lowest Vibrational Mode (1/cm) = 10.0921
E(RM062X) (a.u.) = -4429.88863058
Thermal correction to Enthalpy (a.u.) = 0.761347
Thermal correction to Gibbs Free Energy (a.u.) = 0.643108
Total Entropy (cal/Kmol) = 248.854
Esp(RM062X) (a.u.) = -4432.39792075
Optimised cartesian coordinates (Angstrom):
C -6.496613 2.044445 -0.679122
C -5.949632 3.063507 -1.467797
C -4.597346 3.050443 -1.800090
C -3.798669 2.006540 -1.333111
C -4.346943 1.001152 -0.531299
H -6.586591 3.871412 -1.828941
H -4.169812 3.842173 -2.417408
C -2.161022 0.190470 -0.572998
C -1.422118 -1.859938 0.537849
C -2.555734 -1.535735 1.523792
C -3.787279 -1.063864 0.772536
H -4.536889 -0.679667 1.476120
N -3.415436 0.032298 -0.122112
N -1.189737 -0.685975 -0.322182
C -2.858545 -2.672563 2.525162
C -3.905334 -3.680781 2.040972
C -3.279770 -2.084587 3.873388
H -1.910911 -3.213833 2.681875
H -4.905569 -3.221309 2.002751
H -3.675951 -4.090809 1.047402
H -3.962254 -4.521127 2.747139
H -2.492277 -1.442260 4.291995
H -4.195004 -1.479909 3.770816
H -3.491228 -2.884929 4.596520
H -2.176224 -0.675132 2.099842
H -0.514491 -1.948921 1.146072
C -1.621747 -3.116837 -0.280425
C -1.045456 -4.315011 0.154183
C -2.411878 -3.122035 -1.435160
C -1.284120 -5.505671 -0.530277

H -0.404290 -4.314899 1.039375
C -2.650022 -4.312095 -2.122324
H -2.840925 -2.190539 -1.813826
C -2.093761 -5.507634 -1.666517
H -0.830556 -6.433590 -0.179207
H -3.268981 -4.303668 -3.020556
H -2.280007 -6.437891 -2.204896
C 1.191478 -1.298637 -0.526652
H 0.996421 -2.299014 -0.151069
C 2.510430 -0.782497 -0.575867
O 0.306130 0.752123 -1.261176
C 0.163372 -0.368383 -0.754973
H -4.248216 -1.871032 0.182403
C -5.705419 1.002896 -0.202555
H -6.145866 0.210934 0.401892
H -7.558565 2.060471 -0.432529
H 2.616066 0.119902 -1.183549
C 1.598053 0.556802 1.603884
O 0.674133 -0.136227 2.017479
C 2.933460 0.166192 1.185474
C 3.564122 1.427736 0.844236
H 3.439629 -0.654730 1.688613
N 2.706443 2.412288 0.832579
N 1.511717 1.916076 1.269704
C 0.347390 2.697623 1.199704
C 0.352026 3.878014 0.442584
C -0.827048 2.313211 1.869535
C -0.799789 4.655706 0.356439
H 1.265114 4.166624 -0.074493
C -1.974051 3.099037 1.761918
H -0.827185 1.408543 2.470798
C -1.972787 4.272922 1.009246
H -0.778596 5.571684 -0.237010
H -2.880474 2.787103 2.285841
H -2.874590 4.881981 0.931721
C 4.974686 1.635797 0.474774
C 5.407641 2.877188 -0.017940
C 5.907567 0.599607 0.615778
C 6.740360 3.072217 -0.365282
H 4.679794 3.682498 -0.123083
C 7.242939 0.796895 0.265194
H 5.587779 -0.373521 0.993153
C 7.663665 2.031755 -0.226470
H 7.064002 4.041115 -0.749120
H 7.955985 -0.021742 0.375936
H 8.708365 2.185816 -0.501073
C 3.667616 -1.713354 -0.677377
C 3.755470 -2.864697 0.119319
C 4.701091 -1.441655 -1.581285
C 4.850276 -3.718493 0.015150
H 2.963747 -3.080852 0.841096
C 5.795143 -2.300482 -1.693255
H 4.649292 -0.540496 -2.196280
C 5.875334 -3.438974 -0.893101
H 4.907649 -4.606384 0.646971
H 6.592024 -2.072064 -2.402961
H 6.733542 -4.107906 -0.973452
Se -1.938800 1.752018 -1.600843
----
top_face_S_3-TS1
Frequencies, energies and thermodynamic properties:
Lowest Vibrational Mode (1/cm) = -270.9467
2nd Lowest Vibrational Mode (1/cm) = 11.4514
E(RM062X) (a.u.) = -4429.88895000
Thermal correction to Enthalpy (a.u.) = 0.761056
Thermal correction to Gibbs Free Energy (a.u.) = 0.643073
Total Entropy (cal/Kmol) = 248.315
Esp(RM062X) (a.u.) = -4432.40192831
Optimised cartesian coordinates (Angstrom):
C -7.653126 -1.617908 0.093865
C -7.358192 -2.983594 0.183009
C -6.036858 -3.421169 0.209545
C -5.016027 -2.473786 0.140093
C -5.315327 -1.112762 0.038728
H -8.168291 -3.711155 0.237489
H -5.803767 -4.484056 0.286236
C -2.993219 -0.898439 0.070070
C -1.794184 1.239575 -0.020445
C -3.022998 1.691683 -0.829669
C -4.293398 1.164295 -0.188303
H -5.154468 1.358380 -0.840208
N -4.180495 -0.285291 -0.023296
N -1.836252 -0.233720 0.114412
C -3.085330 3.209473 -1.110012
C -3.755911 4.037567 -0.008084
C -3.799724 3.462849 -2.440042
H -2.041608 3.550819 -1.218365
H -4.837223 3.832550 0.030671
H -3.334956 3.854429 0.989315
H -3.639133 5.107326 -0.231542
H -3.322020 2.921553 -3.268775
H -4.851253 3.139159 -2.389494
H -3.797139 4.534989 -2.681733
H -2.921632 1.186300 -1.804090
H -0.912294 1.455208 -0.641495

C -1.632136 1.930513 1.316259
C -0.791547 3.045447 1.401866
C -2.334391 1.514729 2.450762
C -0.682728 3.758218 2.594040
H -0.216169 3.354761 0.524333
C -2.225358 2.227299 3.645356
H -2.964181 0.621782 2.415606
C -1.406912 3.354416 3.716787
H -0.025452 4.626971 2.648992
H -2.777945 1.895396 4.525264
H -1.321352 3.909343 4.651916
C 0.548108 -0.271270 0.691550
H 0.549212 0.810454 0.763447
C 1.729202 -0.992590 0.894040
O -0.687182 -2.210395 0.174017
C -0.606527 -0.988348 0.324857
H -4.487705 1.625983 0.791994
C -6.639548 -0.666933 0.022466
H -6.883014 0.393292 -0.031612
H -8.692103 -1.287731 0.084085
H 1.631427 -2.065028 1.078763
C 3.717816 -2.085293 -0.779840
O 4.082011 -3.136610 -0.251943
C 2.404888 -1.623366 -1.146752
C 2.607300 -0.334476 -1.726354
H 1.540017 -2.273942 -1.252942
N 3.855231 0.054925 -1.638196
N 4.545375 -0.978060 -1.069092
C 5.895211 -0.793111 -0.727797
C 6.497405 0.454110 -0.963083
C 6.652031 -1.821677 -0.142403
C 7.824469 0.669265 -0.604924
H 5.903693 1.244885 -1.415821
C 7.979258 -1.584911 0.212410
H 6.185171 -2.785192 0.038477
C 8.576744 -0.344640 -0.009205
H 8.274226 1.646319 -0.792022
H 8.553627 -2.391913 0.671644
H 9.616058 -0.171109 0.272983
C 1.569047 0.557260 -2.268370
C 1.767995 1.946276 -2.294131
C 0.364142 0.034933 -2.761411
C 0.793376 2.790779 -2.820851
H 2.702395 2.348039 -1.899148
C -0.601843 0.880122 -3.308958
H 0.195042 -1.043527 -2.738449
C -0.389634 2.259862 -3.341921
H 0.960710 3.869103 -2.838061
H -1.519278 0.456834 -3.723573
H -1.137406 2.923386 -3.778983
C 2.940865 -0.364977 1.443264
C 3.227399 0.994629 1.237253
C 3.850040 -1.144479 2.178234
C 4.386374 1.559624 1.764928
H 2.543829 1.609236 0.648132
C 5.005563 -0.576482 2.706397
H 3.646489 -2.207160 2.316268
C 5.275995 0.777760 2.502856
H 4.600853 2.615610 1.592503
H 5.703974 -1.194624 3.272691
H 6.186035 1.221436 2.910267
Se -3.144114 -2.776173 0.166334
----
prod_1-2
Frequencies, energies and thermodynamic properties:
Lowest Vibrational Mode (1/cm) = 19.7003
2nd Lowest Vibrational Mode (1/cm) = 22.8261
E(RM062X) (a.u.) = -1183.99072171
Thermal correction to Enthalpy (a.u.) = 0.389218
Thermal correction to Gibbs Free Energy (a.u.) = 0.309892
Total Entropy (cal/Kmol) = 166.956
Esp(RM062X) (a.u.) = -1185.29366835
Optimised cartesian coordinates (Angstrom):
C 7.033282 0.355539 -0.116027
C 5.658491 0.153333 -0.082539
C 5.131769 -1.149366 -0.043187
C 6.018060 -2.237406 -0.037963
C 7.396011 -2.034090 -0.071542
C 7.905861 -0.737060 -0.110725
H 7.430686 1.370863 -0.146542
H 4.989257 1.014866 -0.086920
H 5.615899 -3.252088 -0.007532
H 8.072365 -2.889737 -0.067232
H 8.984200 -0.573712 -0.137245
C 3.691915 -1.419892 -0.007593
C 2.697183 -0.516827 -0.009236
H 3.400385 -2.474975 0.023673
H 2.855246 0.561507 -0.040462
C 1.303139 -0.988151 0.031365
O 0.921691 -2.124239 0.057862
O 0.450860 0.080700 0.039307
C -0.889235 -0.085901 0.062452
C -1.732330 -1.175186 0.038906
N -1.657238 1.041641 0.073044
C -3.028373 -0.588791 0.028534

H	-1.446396	-2.218091	0.027679
N	-2.960942	0.733320	0.044541
C	-1.241100	2.401225	0.050901
C	-4.330312	-1.283413	0.011591
C	-1.986649	3.312704	-0.700964
C	-0.129182	2.822988	0.784107
C	-4.394180	-2.681132	-0.049652
C	-5.524480	-0.549767	0.056106
C	-1.609724	4.652855	-0.722498
H	-2.854504	2.957332	-1.255240
C	0.244553	4.165231	0.742502
H	0.431096	2.110756	1.387653
C	-5.626273	-3.332967	-0.066548
H	-3.474768	-3.267316	-0.086588
C	-6.753830	-1.202681	0.038952
H	-5.473132	0.538289	0.106033
C	-0.490865	5.082653	-0.007515
H	-2.192248	5.364615	-1.308902
H	1.112503	4.495876	1.314453
C	-6.810029	-2.596777	-0.022244
H	-5.660305	-4.422381	-0.114917
H	-7.675811	-0.620137	0.074810
H	-0.195623	6.132263	-0.031159
H	-7.774226	-3.107080	-0.035110

1-2_bound

Frequencies, energies and thermodynamic properties:

Lowest Vibrational Mode (1/cm) =	13.9953
2nd Lowest Vibrational Mode (1/cm) =	20.1905
E(RM062X) (a.u.) =	-4429.89927388
Thermal correction to Enthalpy (a.u.) =	0.762137
Thermal correction to Gibbs Free Energy (a.u.) =	0.642701
Total Entropy (cal/Kmol) =	251.374
Esp(RM062X) (a.u.) =	-4432.40801728

Optimised cartesian coordinates (Angstrom):

C	4.594652	-4.208807	-1.752172
C	3.703520	-4.512528	-2.786157
C	2.521704	-3.787653	-2.934096
C	2.240278	-2.761133	-2.034621
C	3.128256	-2.472789	-0.994042
H	3.934901	-5.317478	-3.484416
H	1.826710	-4.020148	-3.742905
C	1.519770	-0.857469	-0.469506
C	1.693544	0.712408	1.378700
C	2.528325	-0.396066	2.047147
C	3.459394	-1.027309	1.024504
H	3.926731	-1.925857	1.449074
N	2.696760	-1.433140	-0.153787
N	1.009771	0.143543	0.209828
C	3.258296	0.033804	3.339566
C	4.671691	0.581957	3.119557
C	3.298145	-1.134371	4.327451
H	2.650172	0.831899	3.796024
H	5.359040	-0.218921	2.805148
H	4.707459	1.381843	2.367001
H	5.059843	0.989993	4.063457
H	2.285260	-1.471909	4.589553
H	3.842951	-1.991795	3.900819
H	3.812799	-0.842902	5.253906
H	1.794616	-1.168053	2.328070
H	0.895530	0.985025	2.083448
C	2.484596	1.961555	1.035592
C	2.530651	3.014441	1.956446
C	3.195937	2.076544	-0.162691
C	3.303581	4.146792	1.702600
H	1.951933	2.949105	2.881132
C	3.963736	3.212645	-0.421813
H	3.142437	1.283384	-0.912632
C	4.026576	4.246257	0.513009
H	3.332207	4.958167	2.431367
H	4.510938	3.291539	-1.362140
H	4.626898	5.133752	0.308311
C	-0.337054	2.177349	-0.185431
H	-0.056457	2.690280	0.737139
C	-0.602704	2.853473	-1.306243
O	-0.873406	0.072501	-1.174865
C	-0.401191	0.663792	-0.169955
H	4.260136	-0.336324	0.717533
C	4.319990	-3.185607	-0.846478
H	5.028616	-2.951081	-0.052960
H	5.520940	-4.774932	-1.649469
H	-0.933145	2.274395	-2.174470
C	-0.474344	4.313248	-1.460534
C	0.400894	5.057225	-0.652030
C	-1.209979	4.984252	-2.448168
C	0.514596	6.435576	-0.811102
H	1.019238	4.542160	0.087259
C	-1.100184	6.365382	-2.603859
H	-1.879791	4.412690	-3.094291
C	-0.239665	7.095990	-1.783941
H	1.205582	6.997021	-0.179590
H	-1.685726	6.873061	-3.372057
H	-0.147900	8.175941	-1.909590
Se	0.704038	-1.639580	-1.988253
O	-1.131795	0.327707	1.155402

C -2.358706 -0.151959 0.995949
C -3.584640 0.481331 0.990361
N -2.604610 -1.485654 0.777152
C -4.521469 -0.558864 0.753536
H -3.750376 1.544977 1.127589
N -3.911796 -1.729862 0.622702
C -1.656782 -2.528002 0.636175
C -5.990652 -0.459559 0.657762
C -1.898810 -3.555034 -0.279664
C -0.495982 -2.536690 1.414594
C -6.637448 0.773398 0.812870
C -6.764787 -1.602651 0.409036
C -0.959357 -4.571290 -0.437695
H -2.817940 -3.533581 -0.863980
C 0.444758 -3.549946 1.235232
H -0.341050 -1.746525 2.148593
C -8.025690 0.863007 0.721412
H -6.051214 1.672977 1.007073
C -8.150931 -1.511626 0.318306
H -6.260612 -2.562165 0.289492
C 0.222293 -4.566332 0.305191
H -1.147440 -5.367362 -1.159799
H 1.354699 -3.555130 1.840842
C -8.788084 -0.278526 0.474055
H -8.513831 1.831127 0.844416
H -8.740227 -2.409516 0.124528
H 0.963696 -5.354616 0.165791
H -9.874532 -0.208459 0.402683

TS_add_1-2
Frequencies, energies and thermodynamic properties:
Lowest Vibrational Mode (1/cm) = -133.3802
2nd Lowest Vibrational Mode (1/cm) = 13.2871
E(RM062X) (a.u.) = -4429.89746828
Thermal correction to Enthalpy (a.u.) = 0.761378
Thermal correction to Gibbs Free Energy (a.u.) = 0.641131
Total Entropy (cal/Kmol) = 253.082
Esp(RM062X) (a.u.) = -4432.40753147
Optimised cartesian coordinates (Angstrom):
C 5.550886 -3.159339 -1.507158
C 4.749863 -3.785543 -2.468540
C 3.434031 -3.373605 -2.667636
C 2.930446 -2.331809 -1.890576
C 3.729644 -1.719401 -0.921220
H 5.158024 -4.598604 -3.069527
H 2.807221 -3.858511 -3.417609
C 1.797870 -0.462713 -0.550130
C 1.608503 1.325742 1.101490
C 2.671710 0.518448 1.865573
C 3.724132 -0.001951 0.901402
H 4.382484 -0.716033 1.413794
N 3.072568 -0.708372 -0.200878
N 1.074589 0.492379 0.011734
C 3.280191 1.254327 3.080410
C 4.518305 2.096937 2.758196
C 3.603820 0.249567 4.188231
H 2.498008 1.929845 3.464238
H 5.377514 1.454286 2.510104
H 4.356208 2.793350 1.923761
H 4.800748 2.688443 3.640430
H 2.703783 -0.291012 4.513869
H 4.341275 -0.492586 3.842821
H 4.032697 0.759876 5.062088
H 2.130806 -0.360390 2.251093
H 0.767411 1.468979 1.791966
C 2.082336 2.674396 0.597757
C 1.842335 3.815816 1.370959
C 2.786573 2.802424 -0.603503
C 2.325695 5.059541 0.967880
H 1.268919 3.730086 2.297693
C 3.269939 4.046890 -1.008707
H 2.953145 1.930565 -1.241214
C 3.046603 5.176302 -0.221419
H 2.133004 5.941023 1.581096
H 3.817780 4.134163 -1.947903
H 3.423056 6.149002 -0.540727
C -0.866709 2.030010 -0.278795
H -0.565735 2.651659 0.563458
C -1.713207 2.481179 -1.211421
O -0.753866 -0.146264 -1.234225
C -0.358121 0.625122 -0.358447
H 4.346976 0.808729 0.492466
C 5.053482 -2.119792 -0.724765
H 5.691893 -1.632759 0.011351
H 6.582197 -3.483756 -1.365860
H -2.013312 1.783943 -1.999533
C -2.290860 3.834600 -1.257106
C -1.744498 4.906545 -0.531157
C -3.417932 4.072750 -2.057372
C -2.324866 6.169904 -0.588823
H -0.842758 4.753493 0.066228
C -4.001434 5.337642 -2.112622
H -3.842378 3.249521 -2.636008
C -3.458388 6.389743 -1.375932
H -1.885823 6.992908 -0.022629

H -4.881956 5.502339 -2.735542
H -3.910439 7.381731 -1.420184
Se 1.183345 -1.598201 -1.925051
O -0.975802 0.090574 1.284706
C -2.027168 -0.654214 1.142258
C -3.381883 -0.348943 1.071979
N -1.949729 -2.027759 0.952759
C -4.030029 -1.589124 0.842549
H -3.808562 0.643894 1.173541
N -3.157752 -2.585253 0.768779
C -0.780731 -2.811728 0.860830
C -5.475874 -1.848408 0.698133
C -0.771755 -3.945607 0.041307
C 0.367291 -2.464999 1.583717
C -6.402097 -0.798508 0.754425
C -5.948819 -3.154424 0.499749
C 0.391869 -4.701944 -0.081652
H -1.679481 -4.208813 -0.500007
C 1.529316 -3.221520 1.440465
H 0.333844 -1.599481 2.242760
C -7.767045 -1.046291 0.615057
H -6.052273 0.223720 0.906962
C -7.311903 -3.400707 0.360606
H -5.227606 -3.971311 0.458784
C 1.552978 -4.337105 0.601813
H 0.393353 -5.579277 -0.730809
H 2.424143 -2.943672 2.003970
C -8.228073 -2.347869 0.417473
H -8.474264 -0.216461 0.660891
H -7.663975 -4.422348 0.207110
H 2.467646 -4.921223 0.487960
H -9.296223 -2.541991 0.308553

TS_turnover_1-2
Frequencies, energies and thermodynamic properties:
Lowest Vibrational Mode (1/cm) = -180.2150
2nd Lowest Vibrational Mode (1/cm) = 10.2018
E(RM062X) (a.u.) = -4429.89426641
Thermal correction to Enthalpy (a.u.) = 0.760655
Thermal correction to Gibbs Free Energy (a.u.) = 0.641038
Total Entropy (cal/Kmol) = 251.757
Esp(RM062X) (a.u.) = -4432.40346592
Optimised cartesian coordinates (Angstrom):
C 3.735129 -4.954203 -1.673908
C 2.895956 -5.017922 -2.788272
C 1.910775 -4.048115 -2.981917
C 1.776041 -3.025171 -2.048365
C 2.608617 -2.970851 -0.922427
H 3.011449 -5.823690 -3.513733
H 1.255473 -4.090568 -3.853465
C 1.327463 -1.055481 -0.418954
C 1.616496 0.329536 1.511914
C 2.193585 -0.938788 2.178858
C 3.064819 -1.697018 1.185135
H 3.323169 -2.682648 1.596911
N 2.336735 -1.903595 -0.062025
N 0.960593 -0.028727 0.259917
C 2.893851 -0.706886 3.536621
C 4.393857 -0.411086 3.438818
C 2.658370 -1.906042 4.458139
H 2.404658 0.166155 3.999507
H 4.950390 -1.306958 3.121791
H 4.623838 0.402675 2.737016
H 4.779062 -0.123208 4.427400
H 1.585814 -2.070019 4.636273
H 3.074605 -2.826820 4.018712
H 3.147642 -1.753239 5.430672
H 1.315402 -1.572756 2.375430
H 0.822285 0.712147 2.173043
C 2.645484 1.432264 1.331687
C 2.786195 2.413080 2.320409
C 3.499766 1.462212 0.224108
C 3.792410 3.375346 2.231008
H 2.103038 2.421232 3.173261
C 4.503594 2.426728 0.130252
H 3.377771 0.732180 -0.579941
C 4.660851 3.378250 1.138898
H 3.893105 4.128699 3.013855
H 5.162671 2.437822 -0.739058
H 5.446920 4.131088 1.064262
C 0.058730 2.301685 -0.307373
H 0.354842 2.718895 0.657026
C 0.262635 2.940864 -1.464188
O -1.021755 0.415327 -1.308842
C -0.577010 0.948230 -0.303616
H 4.003924 -1.159019 0.977765
C 3.603791 -3.932029 -0.732953
H 4.273890 -3.888743 0.125000
H 4.509160 -5.709288 -1.532248
H -0.109157 2.460826 -2.375313
C 0.955188 4.229924 -1.617343
C 1.806091 4.731018 -0.618627
C 0.791300 4.972643 -2.795956
C 2.457071 5.949110 -0.788582
H 1.982394 4.145092 0.286119

C 1.438516 6.195881 -2.963631
H 0.144219 4.583912 -3.585270
C 2.271937 6.688977 -1.959355
H 3.122287 6.318411 -0.005831
H 1.295528 6.763996 -3.884129
H 2.784469 7.643072 -2.091744
Se 0.517355 -1.610176 -2.065376
O -1.296228 0.757271 0.938944
C -2.586662 0.410127 0.805443
C -3.711726 1.203017 0.842682
N -3.004037 -0.874228 0.587392
C -4.784007 0.298540 0.626304
H -3.728582 2.277843 0.990527
N -4.335405 -0.940187 0.470609
C -2.219055 -2.046809 0.436197
C -6.229006 0.591725 0.568066
C -2.588832 -2.991954 -0.523623
C -1.115308 -2.266453 1.260708
C -6.703726 1.896441 0.753336
C -7.150839 -0.436379 0.322718
C -1.832748 -4.151252 -0.671548
H -3.462926 -2.799437 -1.144864
C -0.359070 -3.427164 1.095912
H -0.860996 -1.527443 2.021696
C -8.069706 2.168628 0.694122
H -6.000852 2.708692 0.945502
C -8.514507 -0.162828 0.264090
H -6.779817 -1.451711 0.179477
C -0.709594 -4.368892 0.128534
H -2.117248 -4.885514 -1.426635
H 0.505341 -3.606936 1.739789
C -8.980204 1.140782 0.449213
H -8.423800 3.190327 0.839983
H -9.220491 -0.972517 0.072050
H -0.110828 -5.272286 0.002218
H -10.049160 1.354013 0.402437
----
catAc-nuc
Frequencies, energies and thermodynamic properties:
Lowest Vibrational Mode (1/cm) = 8.9496
2nd Lowest Vibrational Mode (1/cm) = 19.9546
E(RM062X) (a.u.) = -4429.90415180
Thermal correction to Enthalpy (a.u.) = 0.762069
Thermal correction to Gibbs Free Energy (a.u.) = 0.641968
Total Entropy (cal/Kmol) = 252.775
Esp(RM062X) (a.u.) = -4432.41851823
Optimised cartesian coordinates (Angstrom):
C 6.918001 0.457325 -0.381766
C 7.044114 -0.665749 -1.209941
C 5.915301 -1.321768 -1.689218
C 4.657976 -0.839739 -1.322970
C 4.535733 0.270973 -0.484153
H 8.034904 -1.027517 -1.485484
H 6.010306 -2.192914 -2.338501
C 2.261748 -0.125901 -0.772087
C 0.471887 1.265648 0.202235
C 1.521673 1.573019 1.280567
C 2.889735 1.770127 0.658521
H 3.654498 1.811360 1.443906
N 3.205197 0.625683 -0.201722
N 0.947274 0.132050 -0.630111
C 1.114343 2.733935 2.218674
C 1.566563 4.120138 1.748427
C 1.626974 2.469690 3.635484
H 0.012545 2.729263 2.262482
H 2.660054 4.224038 1.829919
H 1.275303 4.340291 0.711976
H 1.120511 4.890145 2.393411
H 1.197408 1.546823 4.047313
H 2.724343 2.368859 3.647740
H 1.362140 3.302132 4.302579
H 1.583180 0.659567 1.890034
H -0.410780 0.881220 0.738146
C 0.088309 2.453604 -0.655319
C -1.050791 3.194106 -0.319687
C 0.867315 2.858521 -1.743812
C -1.385340 4.342122 -1.035297
H -1.684938 2.864805 0.507533
C 0.533257 4.007920 -2.460946
H 1.739054 2.274455 -2.050837
C -0.587739 4.756437 -2.102856
H -2.274873 4.911098 -0.761741
H 1.149710 4.315529 -3.306551
H -0.848129 5.654094 -2.665078
C -1.393404 -0.280652 -1.303103
H -1.666956 0.705695 -0.942076
C -2.328064 -1.137212 -1.762799
O 0.415138 -1.711042 -1.846901
C 0.001588 -0.713145 -1.291256
H 2.956674 2.689131 0.056138
C 5.668295 0.940564 -0.010636
H 5.587135 1.821667 0.623847
H 7.811915 0.966354 -0.020902
H -2.000460 -2.136875 -2.061740
C -1.416215 -3.042847 0.860650

O -0.801029 -4.059874 0.508059
C -2.783003 -2.651931 0.817631
C -2.848748 -1.361206 1.379599
H -3.587294 -3.249813 0.400049
N -1.666370 -0.917139 1.786019
N -0.788587 -1.917286 1.468128
C 0.508502 -1.893134 1.987508
C 0.769283 -1.248219 3.207163
C 1.563518 -2.500307 1.288314
C 2.071822 -1.184889 3.699043
H -0.063772 -0.793893 3.743272
C 2.863424 -2.414002 1.784012
H 1.338577 -3.016380 0.356244
C 3.129578 -1.752310 2.985568
H 2.262796 -0.681653 4.649012
H 3.680909 -2.869051 1.219263
H 4.149221 -1.693267 3.368736
C -4.033159 -0.485736 1.472917
C -5.318326 -0.986040 1.225584
C -3.890541 0.874426 1.787265
C -6.430373 -0.148675 1.285120
H -5.445254 -2.041632 0.979349
C -5.001880 1.712390 1.843551
H -2.889285 1.258156 1.991926
C -6.278052 1.204440 1.590208
H -7.423509 -0.554804 1.084005
H -4.873474 2.768777 2.087945
H -7.149316 1.860324 1.632142
C -3.753261 -0.835560 -1.913965
C -4.266118 0.465565 -1.772192
C -4.636472 -1.881166 -2.220749
C -5.625705 0.707318 -1.929078
H -3.596308 1.297694 -1.546882
C -6.000195 -1.639521 -2.371142
H -4.242102 -2.893439 -2.332650
C -6.496672 -0.344288 -2.226734
H -6.012069 1.721050 -1.814306
H -6.676181 -2.463134 -2.605289
H -7.564147 -0.151443 -2.345960
Se 2.968027 -1.521710 -1.817778
----
amide-1-2-twist
Frequencies, energies and thermodynamic properties:
Lowest Vibrational Mode (1/cm) = 14.3958
2nd Lowest Vibrational Mode (1/cm) = 19.8890
E(RM062X) (a.u.) = -1780.41660230
Thermal correction to Enthalpy (a.u.) = 0.345218
Thermal correction to Gibbs Free Energy (a.u.) = 0.258659
Total Entropy (cal/Kmol) = 182.180
Esp(RM062X) (a.u.) = -1782.19146341
Optimised cartesian coordinates (Angstrom):
C 3.618821 -0.441863 -0.976765
C 2.626512 -0.164791 -0.134708
H 3.565845 -0.177938 -2.035723
H 2.687985 -0.457747 0.911270
C 1.425919 0.545371 -0.677143
O 1.306934 0.784666 -1.852929
C -0.568416 1.847688 -0.255578
C -0.579713 3.218957 -0.389599
N -1.803106 1.392783 -0.604920
C -1.894910 3.512137 -0.826715
H 0.243888 3.900915 -0.198678
N -2.610613 2.399181 -0.939617
C -2.275189 0.051852 -0.552728
C -3.446108 -0.234150 0.141417
C -1.566568 -0.969329 -1.191993
C -3.922764 -1.541993 0.204773
H -3.981792 0.576371 0.635702
C -2.025761 -2.275751 -1.115124
H -0.672483 -0.729670 -1.769641
C -3.206573 -2.573392 -0.417737
H -4.842349 -1.745579 0.750651
H -1.492975 -3.090673 -1.605308
N 0.438062 0.983607 0.223922
C -2.488767 4.846179 -1.149588
H -2.409049 5.531935 -0.294921
H -1.973930 5.312494 -2.001212
H -3.547808 4.723459 -1.405880
C 4.871734 -1.131348 -0.529985
F 4.855176 -1.431943 0.767482
F 5.939039 -0.360654 -0.750917
F 5.056893 -2.265750 -1.206236
O -3.580208 -3.867933 -0.401830
C -4.761356 -4.218338 0.282039
H -4.876813 -5.301512 0.172519
H -4.693356 -3.967222 1.352288
H -5.639374 -3.714449 -0.152118
S 0.304562 0.529696 1.887185

C	1.421058	1.635517	2.703505
H	2.434166	1.476345	2.317806
H	1.071392	2.657476	2.520304
H	1.364470	1.386142	3.770367
O	-1.050057	0.865917	2.265391
O	0.784746	-0.832626	2.002435

----

8. Crystallographic Details

X-ray Crystallography

X-ray diffraction data for compounds **39** and **49** were collected using a Rigaku MM-007HF High Brilliance RA generator/confocal optics with XtaLAB P200 diffractometer [Cu Kα radiation (λ = 1.54187 Å)]. X-ray diffraction data for compound **45** were collected using a Rigaku FR-X Ultrahigh Brilliance Microfocus RA generator/confocal optics with XtaLAB P200 diffractometer [Mo Kα radiation (λ = 0.71073 Å)]. Data for all compounds analysed were collected (using a calculated strategy) and processed (including correction for Lorentz, polarization and absorption) using CrysAlisPro. [71] Structures were solved by dual-space methods (SHELXT [72]) and refined by full-matrix least-squares against F^2 (SHELXL-2019/3 [73]). Non-hydrogen atoms were refined anisotropically, and hydrogen atoms were refined using a riding model. All calculations were performed using the Olex2 [74] interface. Selected crystallographic data are presented in Table 6. CCDC 2410530 - 2410532 contains the supplementary crystallographic data for this paper. These data can be obtained free of charge from The Cambridge Crystallographic Data Centre via www.ccdc.cam.ac.uk/structures.

Table 6: Selected crystallographic data

	**39**	**45**	**49**
formula	$C_{16}H_{16}N_3O_3F_3S$	$C_{24}H_{18}N_2O_2$	$C_{25}H_{17}N_2O_2F_3$
fw	387.38	366.40	434.40
temperature [K]	100	100	100
crystal description	Colourless prism	Colourless prism	Colourless needle
crystal size [mm^3]	0.1 × 0.06 × 0.04	0.26 × 0.25 × 0.14	0.36 × 0.05 × 0.03
space group	$P2_1$	$P2_1$/c	$P2_1$
a [Å]	8.92503(8)	7.18601(15)	11.7501(2)
b [Å]	9.76362(8)	22.9853(5)	7.67843(10)
c [Å]	9.96666(10)	11.2434(3)	12.2764(2)
β [°]	104.8276(10)	93.6583(19)	117.049(2)
vol [Å]^3	839.580(14)	1853.32(7)	986.45(3)
Z	2	4	2
ρ (calc) [g/cm^3]	1.532	1.313	1.463

μ [mm^{-1}]	2.226	0.084	0.954
F(000)	400	768	448
reflections collected	30004	34778	34537
independent reflections (R_{int})	3384 (0.0604)	4403 (0.0388)	3964 (0.0816)
parameters, restraints	238, 1	253, 0	289, 1
GooF on F^2	1.106	1.073	1.140
R_1 [$I > 2\sigma(I)$]s	0.0319	0.0362	0.0384
wR_2 (all data)	0.0876	0.0917	0.1075
largest diff. peak/hole [e/Å^3]	0.38/-0.28	0.28/-0.24	0.23/-0.30
flack parameter	<0.01		0.08(10)

9. References

[1] E. Vitaku, D. T. Smith, J. T. Njardarson, *J. Med. Chem.* **2014**, *57*, 10257-10274.

[2] C. Lamberth, *Pest Manag. Sci.* **2013**, *69*, 1106-1114.

[3] N. Kerru, L. Gummidi, S. Maddila, K. K. Gangu, S. B. Jonnalagadda, *Molecules* **2020**, *25*, 1909.

[4] C. M. Marshall, J. G. Federice, C. N. Bell, P. B. Cox, J. T. Njardarson, *J. Med. Chem.* **2024**, *67*, 11622-11655.

[5] B. B. Hansen, T. H. Jepsen, M. Larsen, R. Sindet, T. Vifian, M. N. Burhardt, J. Larsen, J. G. Seitzberg, M. A. Carnerup, A. Jerre, C. Mølck, P. Lovato, S. Rai, V. R. Nasipireddy, A. Ritzén, *J. Med. Chem.* **2020**, *63*, 7008-7032.

[6] J. Li, X.-Y. Duan, X. Ren, Y. Li, J. Qi, *J. Org. Chem.* **2023**, *88*, 16621-16632.

[7] Y. Li, X. Huang, J. He, S. Peng, J. Wang, M. Lang, *Adv. Synth. Catal* **2023**, *365*, 490-495.

[8] S. Minami, M. Tomita, K. Kawaguchi, *Chem. Pharm. Bull.* **1972**, *20*, 1716-1728.

[9] J. D. Ratajczyk, L. R. Swett, *J. Heterocycl. Chem.* **1975**, *12*, 517-522.

[10] L. R. Swett, J. D. Ratajczyk, C. W. Nordeen, G. H. Aynilian, *J. Heterocycl. Chem.* **1975**, *12*, 1137-1142.

[11] V. Y. Shuvalov, E. Y. Vlasova, T. Y. Zheleznova, A. S. Fisyuk, *Beilstein J. Org. Chem.* **2023**, *19*, 1155-1160.

[12] J. Quiroga, A. Hormaza, B. Insuasty, M. Márquez, *J. Heterocycl. Chem.* **1998**, *35*, 409-412.

[13] G. Nie, J. Sun, C. Mou, K. Tang, Y. R. Chi, T. Li, *Org. Lett.* **2023**, *25*, 134-139.

[14] Q. Wu, J. Han, J. Huang, H. Zhang, M. Ren, X. Zhang, Z. Fu, *Org. Biomol. Chem.* **2023**, *21*, 6898-6902.

[15] S. Wang, J. Izquierdo, C. Rodríguez-Escrich, M. A. Pericàs, *ACS Catal.* **2017**, *7*, 2780-2785.

[16] V. B. Birman, X. Li, *Org. Lett.* **2006**, *8*, 1351-1354.

[17] J. Merad, J.-M. Pons, O. Chuzel, C. Bressy, *Eur. J. Org. Chem.* **2016**, *2016*, 5589-5610.

[18] V. B. Birman, *Aldrichimca Acta* **2016**, *49*, 23-41.

[19] J. E. Taylor, S. D. Bull, J. M. J. Williams, *Chem. Soc. Rev.* **2012**, *41*, 2109-2121.

[20] C. McLaughlin, A. D. Smith, *Chem. Eur. J.* **2021**, *27*, 1533-1555.

[21] J. Bitai, M. T. Westwood, A. D. Smith, *Org. Biomol. Chem.* **2021**, *19*, 2366-2384.

[22] S. Vellalath, D. Romo, *Angew. Chem. Int. Ed.* **2016**, *55*, 13934-13943.

[23] B. K. Redden, R. W. Clark, Z. Gong, M. M. Rahman, D. V. Peryshkov, S. L. Wiskur, *Org. Biomol. Chem.* **2021**, *19*, 10181-10188.

[24] L. S. Vogl, P. Mayer, R. Robiette, M. Waser, *Angew. Chem. Int. Ed.* **2024**, *63*, e202315345.

[25] M. Piringer, M. Hofer, L. S. Vogl, P. Mayer, M. Waser, *Adv. Synth. Catal* **2024**, *366*, 2115-2122.

[26] C. M. Young, A. Elmi, D. J. Pascoe, R. K. Morris, C. McLaughlin, A. M. Woods, A. B. Frost, A. d. l. Houpliere, K. B. Ling, T. K. Smith, A. M. Z. Slawin, P. H. Willoughby, S. L. Cockroft, A. D. Smith, *Angew. Chem. Int. Ed.* **2020**, *59*, 3705-3710.

[27] G. Li, M. Szostak, *Synthesis* **2020**, *52*, 2579-2599.

[28] R. Takise, K. Muto, J. Yamaguchi, *Chem. Soc. Rev.* **2017**, *46*, 5864-5888.

[29] D. Kaiser, A. Bauer, M. Lemmerer, N. Maulide, *Chem. Soc. Rev.* **2018**, *47*, 7899-7925.

[30] C. Liu, M. Szostak, *Chem. Eur. J.* **2017**, *23*, 7157-7173.

[31] P. Gao, M. M. Rahman, A. Zamalloa, J. Feliciano, M. Szostak, *J. Org. Chem.* **2023**, *88*, 13371-13391.
[32] F. K. Winkler, J. D. Dunitz, *J. Mol. Biol.* **1971**, *59*, 169-182.
[33] G. Meng, J. Zhang, M. Szostak, *Chem. Rev.* **2021**, *121*, 12746-12783.
[34] J. Bitai, A. J. Nimmo, A. M. Z. Slawin, A. D. Smith, *Angew. Chem., Int. Ed. Engl.* **2022**, *61*, e202202621.
[35] Deposition numbers 2410530 (for **39**), 2410531 (for **45**), and 2410532 (for **49**) contain the supplementary crystallographic data for this paper. These data are provided free of charge by the joint Cambridge Crystallographic Data Centre and Fachinformationszentrum Karlsruhe.
[36] S. R. Yetra, S. Mondal, E. Suresh, A. T. Biju, *Org. Lett.* **2015**, *17*, 1417-1420.
[37] Y. Zhao, D. G. Truhlar, *Theor. Chem. Acc.* **2008**, *120*, 215-241.
[38] F. Weigend, *Phys. Chem. Chem. Phys.* **2006**, *8*, 1057-1065.
[39] F. Weigend, R. Ahlrichs, *Phys. Chem. Chem. Phys.* **2005**, *7*, 3297-3305.
[40] J. Tomasi, B. Mennucci, E. Cancès, *J. Mol. Struct.: THEOCHEM* **1999**, *464*, 211-226.
[41] M. J. Frisch, G. W. Trucks, H. B. Schlegel, G. E. Scuseria, M. A. Robb, J. R. Cheeseman, G. Scalmani, V. Barone, G. A. Petersson, H. Nakatsuji, X. Li, M. Caricato, A. V. Marenich, J. Bloino, B. G. Janesko, R. Gomperts, B. Mennucci, H. P. Hratchian, J. V. Ortiz, A. F. Izmaylov, J. L. Sonnenberg, Williams, F. Ding, F. Lipparini, F. Egidi, J. Goings, B. Peng, A. Petrone, T. Henderson, D. Ranasinghe, et al., *Gaussian 16, Revision. C.01*, Guassian Inc., Wallingford CT, **2019**.
[42] An alternative route to generate **65** from **64** via an anionic oxy-Cope mechanism has not been computationally probed.
[43] Q. Shi, W. Zhang, Y. Wang, L. Qu, D. Wei, *Org. Biomol. Chem.* **2018**, *16*, 2301-2311.
[44] F. M. Bickelhaupt, K. N. Houk, *Angew. Chem., Int. Ed. Engl.* **2017**, *56*, 10070-10086.
[45] G. Szilágyi, P. Dvortsák, *Monatsh. Chem.* **1989**, *120*, 131-137.
[46] M. Nayak, H. Batchu, S. Batra, *Tetrahedron Lett.* **2012**, *53*, 4206-4208.
[47] S. Azeez, P. Sureshbabu, S. Sabiah, J. Kandasamy, *Org. Biomol. Chem.* **2022**, *20*, 2048-2053.
[48] A. Matviitsuk, M. D. Greenhalgh, D.-J. B. Antúnez, A. M. Z. Slawin, A. D. Smith, *Angew. Chem., Int. Ed. Engl.* **2017**, *56*, 12282-12287.
[49] J.-E. Lee, J. Kwon, J. Yun, *Chem. Commun.* **2008**, *2008*, 733-734.
[50] L. Zhang, R. Qiu, X. Xue, Y. Pan, C. Xu, D. Wang, X. Wang, L. Xu, H. Li, *Chem. Commun.* **2014**, *50*, 12385-12388.
[51] R. d. Río-Rodríguez, M. T. Westwood, M. Sicignano, M. Juhl, J. A. Fernández-Salas, J. Alemán, A. D. Smith, *Chem. Commun.* **2022**, *58*, 7277-7280.
[52] W. C. Hartley, F. Schiel, E. Ermini, P. Melchiorre, *Angew. Chem. Int. Ed.* **2022**, *61*, e202204735.
[53] N. Everson, K. Yniguez, L. Loop, H. Lazaro, B. Belanger, G. Koch, J. Bach, A. Manjunath, R. Schioldager, J. Law, M. Grabenauer, S. Eagon, *Tetrahedron Lett.* **2019**, *60*, 72-74.
[54] M. Marinozzi, A. Carotti, E. Sansone, A. Macchiarulo, E. Rosatelli, R. Sardella, B. Natalini, G. Rizzo, L. Adorini, D. Passeri, F. De Franco, M. Pruzanski, R. Pellicciari, *Bioorganic & Medicinal Chemistry* **2012**, *20*, 3429-3445.
[55] Y.-Y. Huang, H.-C. Lin, K.-M. Cheng, W.-N. Su, K.-C. Sung, T.-P. Lin, J.-J. Huang, S.-K. Lin, F. F. Wong, *Tetrahedron* **2009**, *65*, 9592-9597.
[56] M. J. Sarma, S. Jindani, B. Ganguly, S. Pabbaraja, G. Mehta, *J. Org. Chem.* **2022**, *87*, 884-891.
[57] D. Krištofíková, M. Mečiarová, E. Rakovský, R. Šebesta, *ACS Sustain. Chem. Eng.* **2020**, *8*, 14417-14424.

[58] M. Desroses, M.-C. Jacques-Cordonnier, S. Llona-Minguez, S. Jacques, T. Koolmeister, T. Helleday, M. Scobie, *Eur. J. Org. Chem.* **2013**, *2013*, 5879-5885.
[59] A. Kimata, H. Nakagawa, R. Ohyama, T. Fukuuchi, S. Ohta, T. Suzuki, N. Miyata, *J. Med. Chem.* **2007**, *50*, 5053-5056.
[60] T. M. Beck, B. Breit, *Eur. J. Org. Chem.* **2016**, *2016*, 5839-5844.
[61] A. Dahal, M. Lo, S. Singh, H. Vo, D. ElHage, S. D. Jois, S. Murru, *Chem. Biol. Drug. Des.* **2022**, *99*, 620-633.
[62] N. Depa, H. Erothu, *Med. Chem. Res.* **2021**, *30*, 1087-1098.
[63] K. Kumar, B. Singh, S. Hore, R. P Singh, *New J. Chem.* **2021**, *45*, 13747-13750.
[64] X. Han, W. Yao, T. Wang, Y. R. Tan, Z. Yan, J. Kwiatkowski, Y. Lu, *Angew. Chem., Int. Ed. Engl.* **2014**, *53*, 5643-5647.
[65] A. Schäfer, H. Horn, R. Ahlrichs, *J. Chem. Phys.* **1992**, *97*, 2571-2577.
[66] A. Schäfer, C. Huber, R. Ahlrichs, *J. Chem. Phys.* **1994**, *100*, 5829-5835.
[67] R. L. Martin, P. J. Hay, L. R. Pratt, *J. Phys. Chem. A* **1998**, *102*, 3565-3573.
[68] The PyMOL Molecular Graphics System, Version 2.4.1, Schrödinger, LLC.
[69] C. Wang, S.-J. Li, Q.-C. Zhang, D. Wei, L. Ding, *Catal. Sci. Technol.* **2020**, *10*, 3664-3669;
[70] J. Wu, C. M. Young, A. A. Watts, A. M. Z. Slawin, G. R. Boyce, M. Bühl, A. D. Smith, *Org. Lett.* **2022**, *24*, 4040-4045.
[71] CrysAlisPro v1.171.42.94a, 42.96a, & 43.109a Rigaku Oxford Diffraction, Rigaku Corporation, Tokyo, Japan, **2023**.
[72] G. M. Sheldrick, *Acta Cryst. C* **2015**, *71*, 3-8.
[73] G. M. Sheldrick, *Acta Cryst. A* **2015**, *71*, 3-8.
[74] O. V. Dolomanov, L. J. Bourhis, R. J. Gildea, J. a. K. Howard, H. Puschmann, *J. Appl. Crystallogr.* **2009**, *42*, 339-341.

10. NMR Spectra

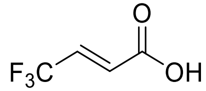

S17, ^{1}H, $CDCl_3$, 400 MHz

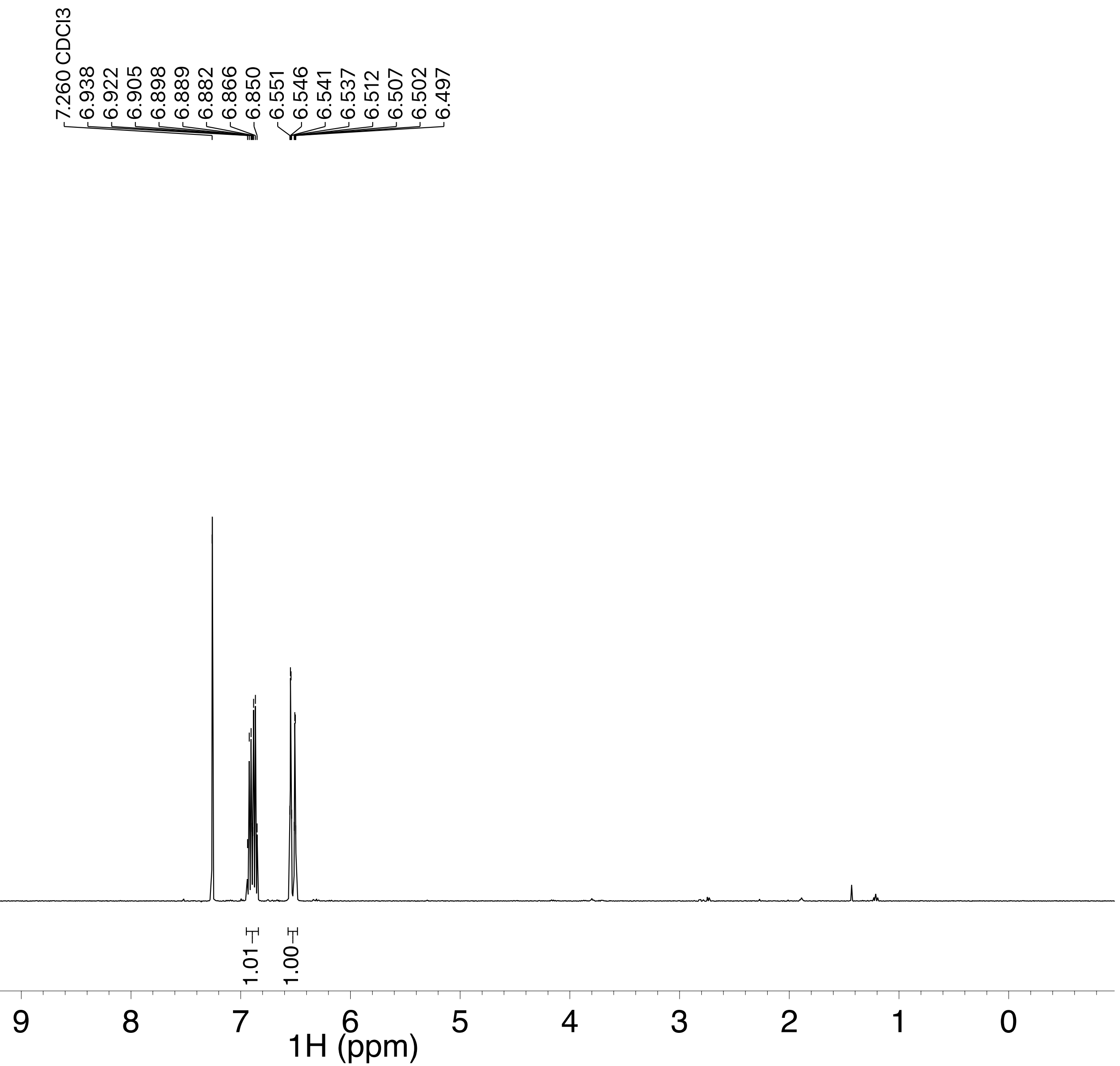

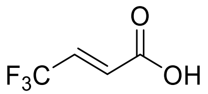

S17, $^{19}F\{^1H\}$, $CDCl_3$, 377 MHz

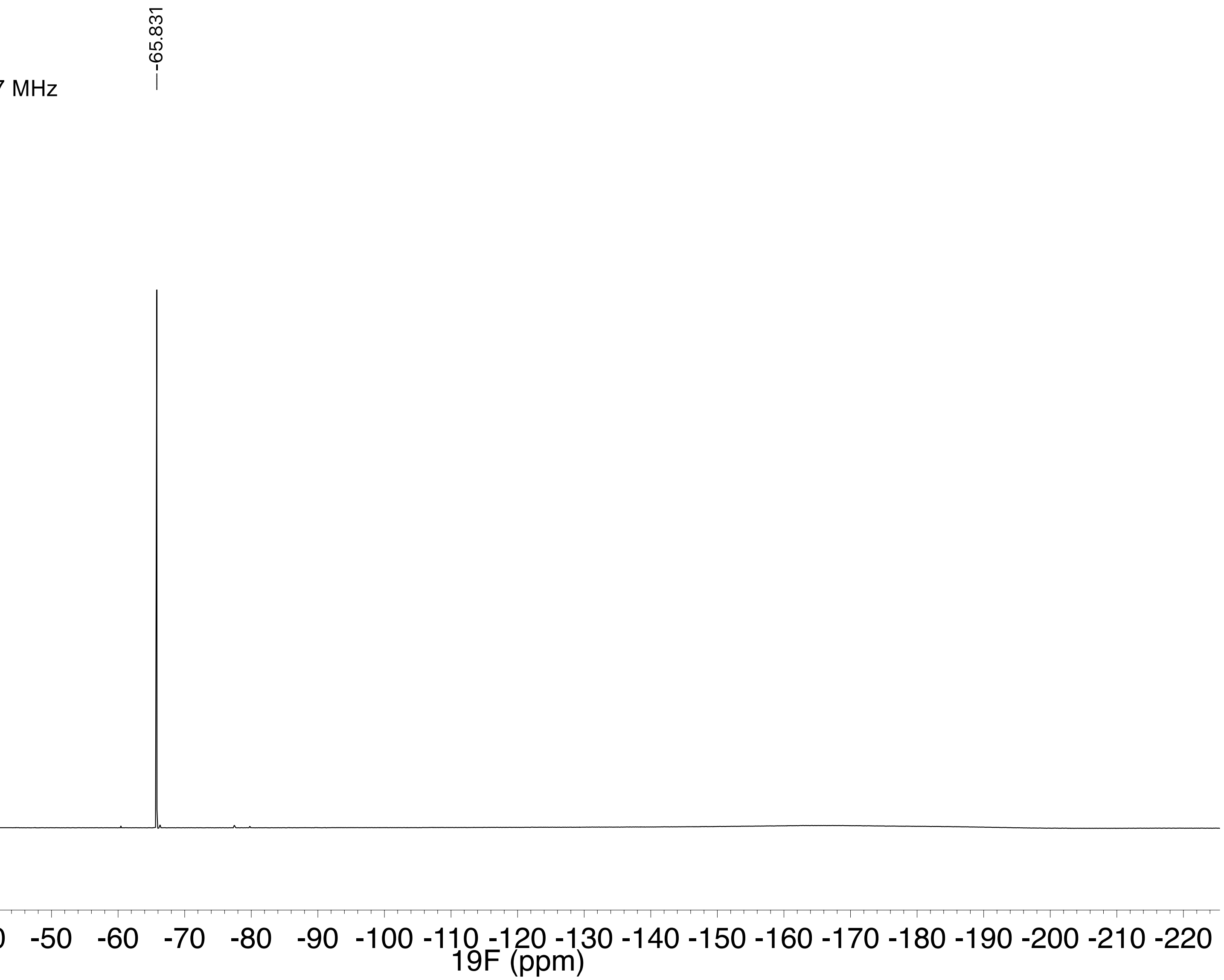

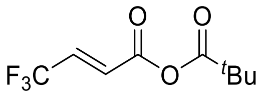

14, ^{1}H, $CDCl_3$, 400 MHz

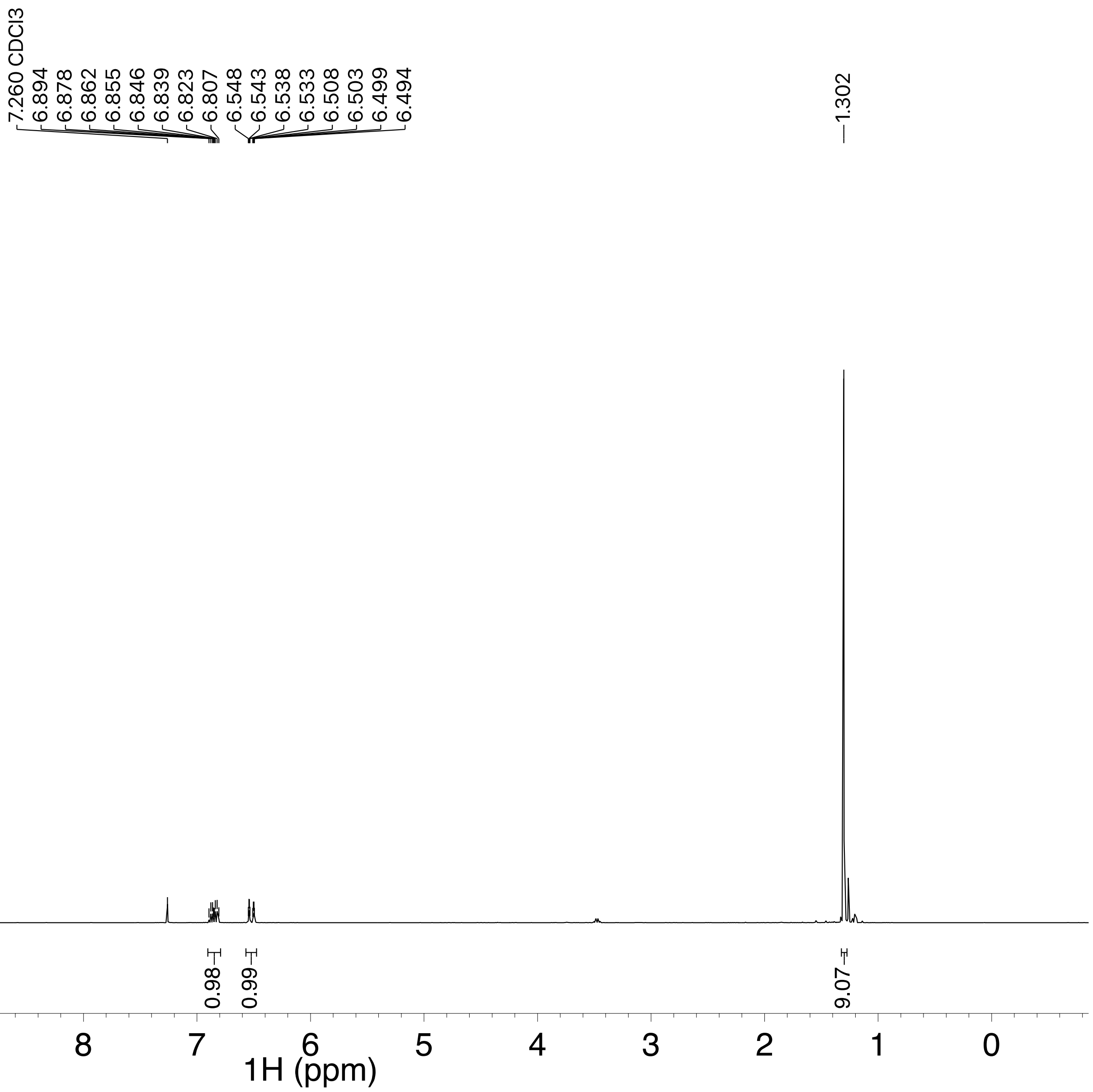

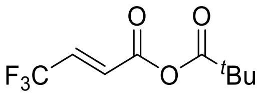

14, $^{13}C\{^1H\}$, $CDCl_3$, 126 MHz

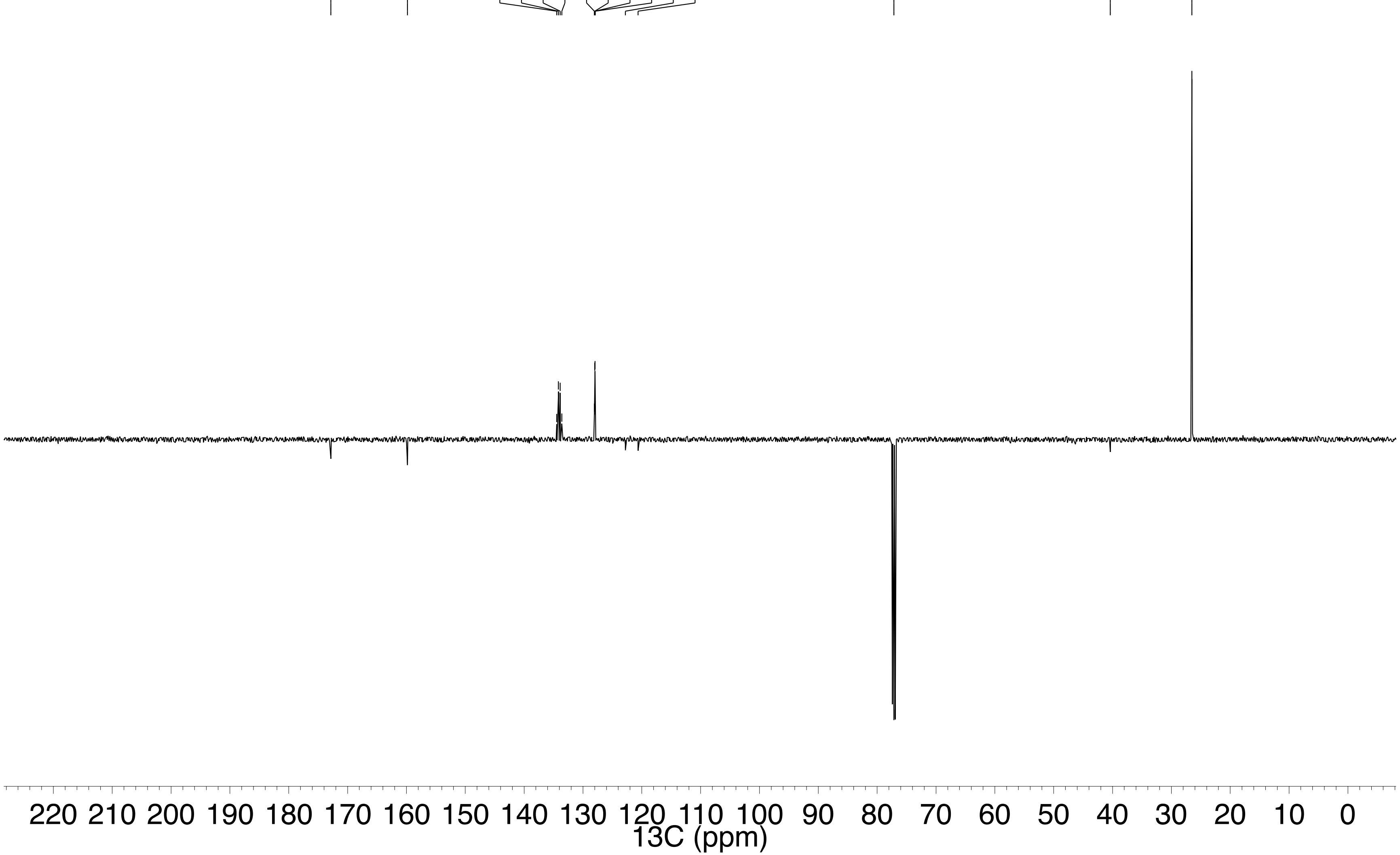

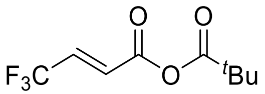

14, $^{19}F\{^{1}H\}$, $CDCl_3$, 377 MHz

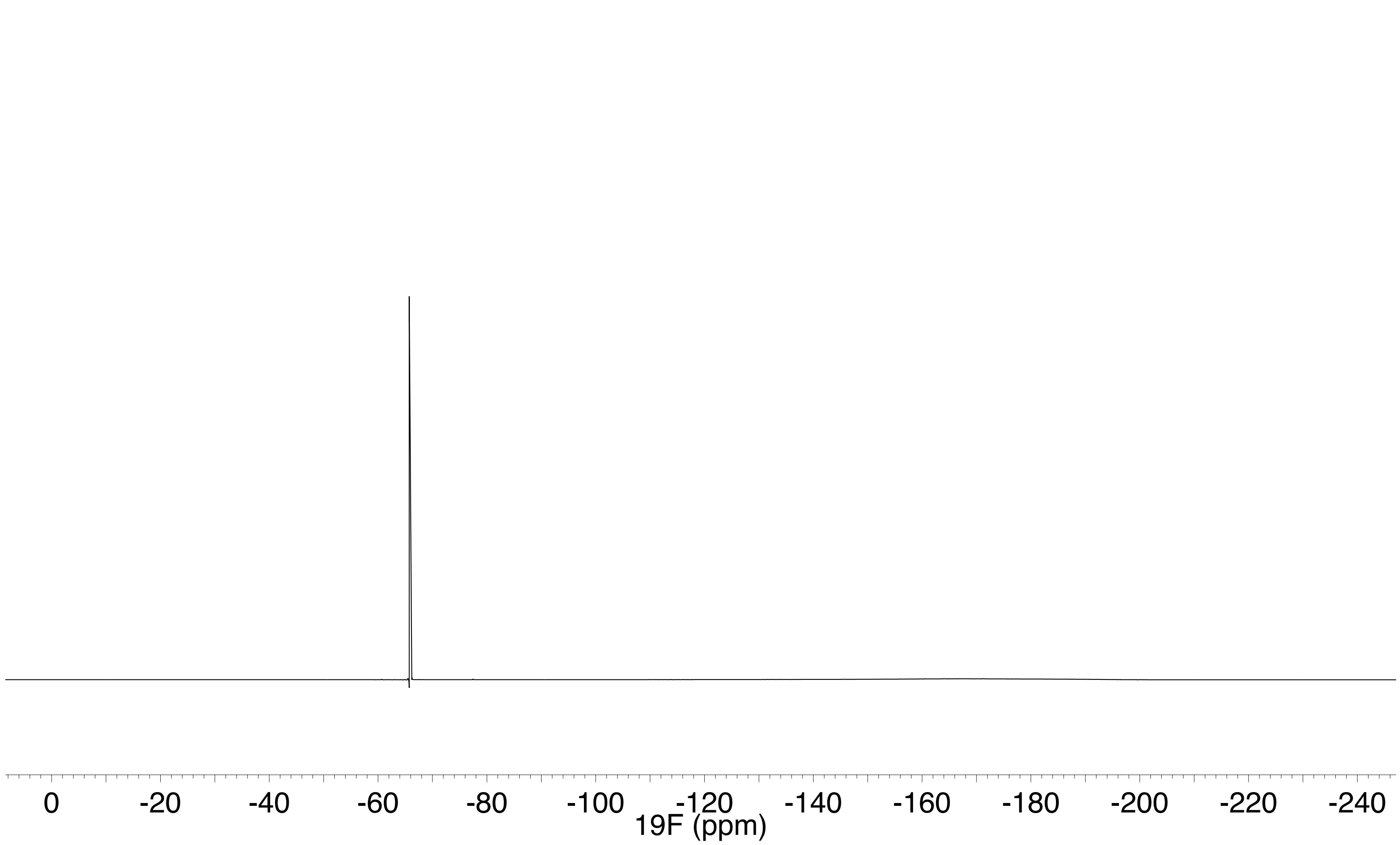

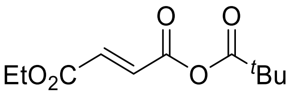

(*E*)-**S18**, ^{1}H, $CDCl_3$, 500 MHz

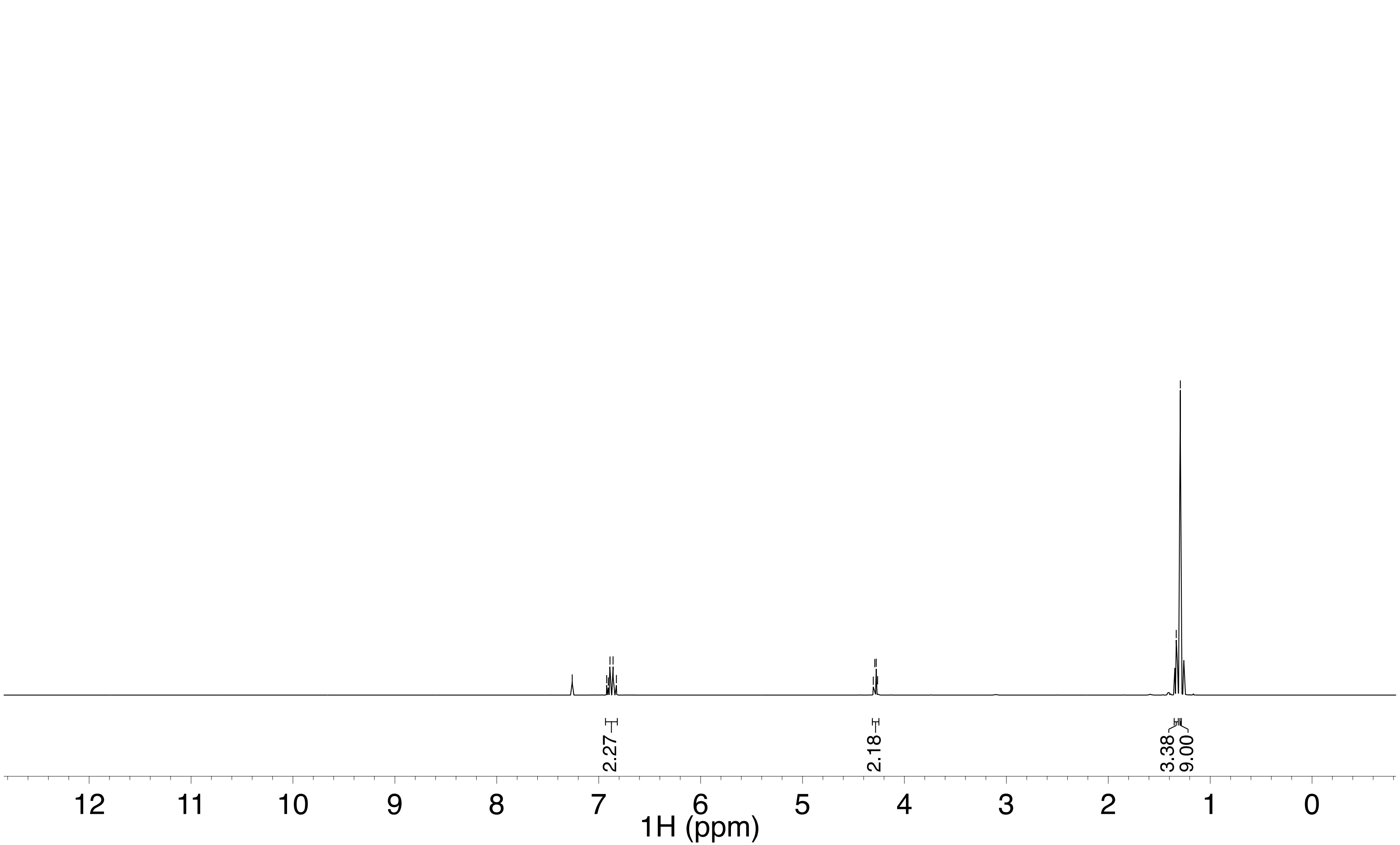

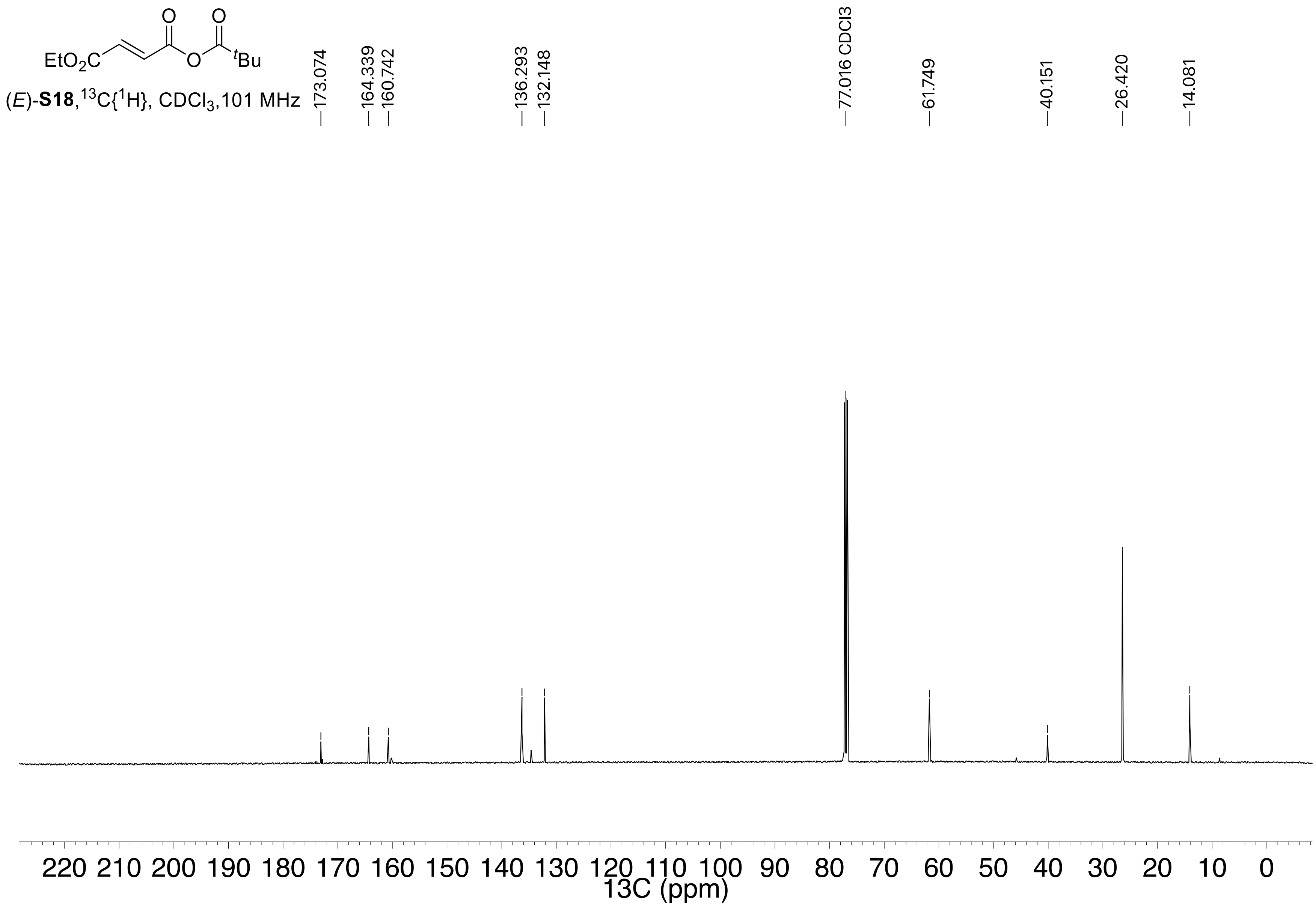

EtO2C
O
O
O
tBu
(E)-S18, 13C{1H}, CDCl3, 101 MHz
173.074
164.339
160.742
136.293
132.148
77.016 CDCl3
61.749
40.151
26.420
14.081
220 210 200 190 180 170 160 150 140 130 120 110 100 90 80 70 60 50 40 30 20 10 0
13C (ppm)

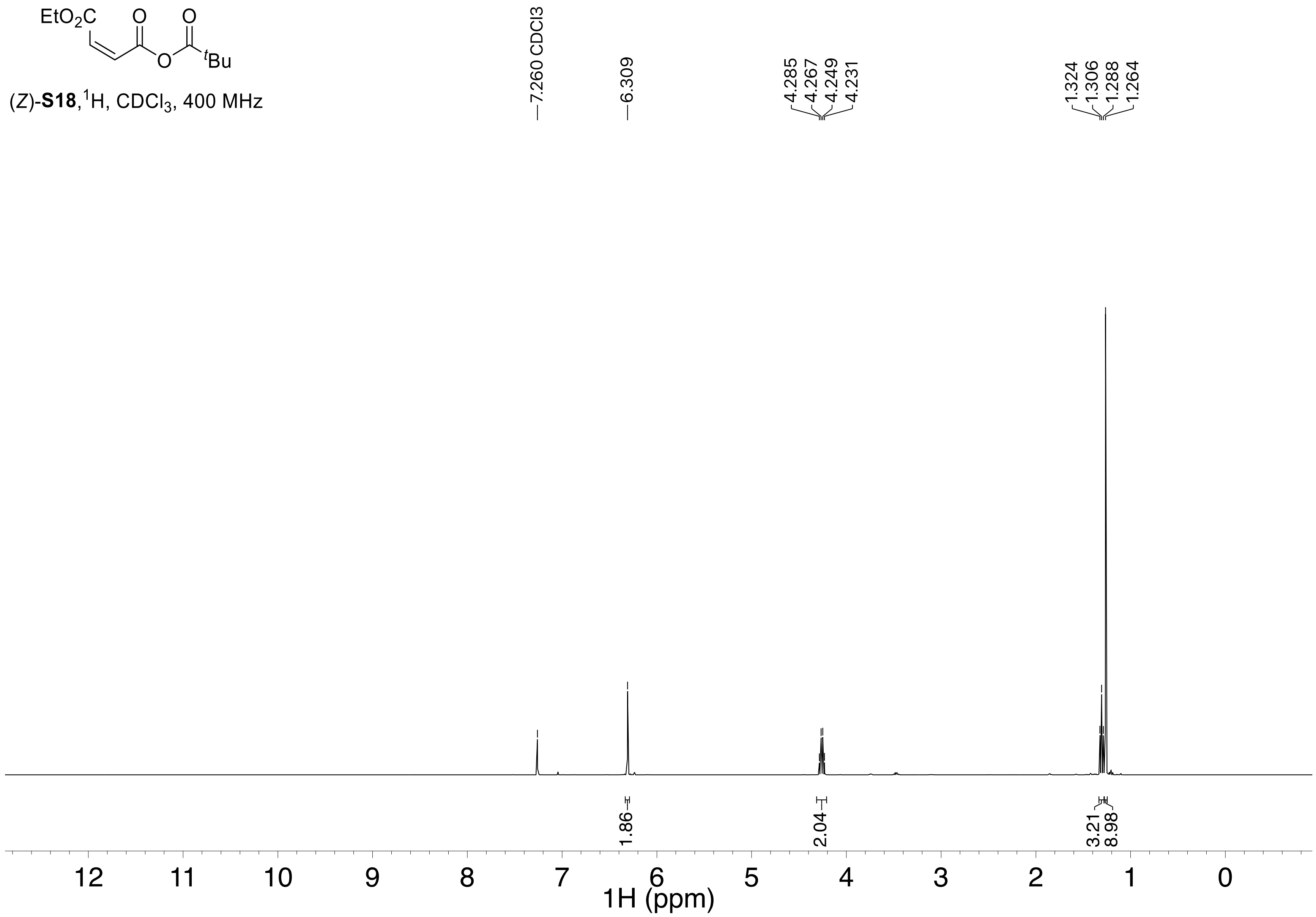

(*Z*)-**S18**, ^{1}H, $CDCl_3$, 400 MHz

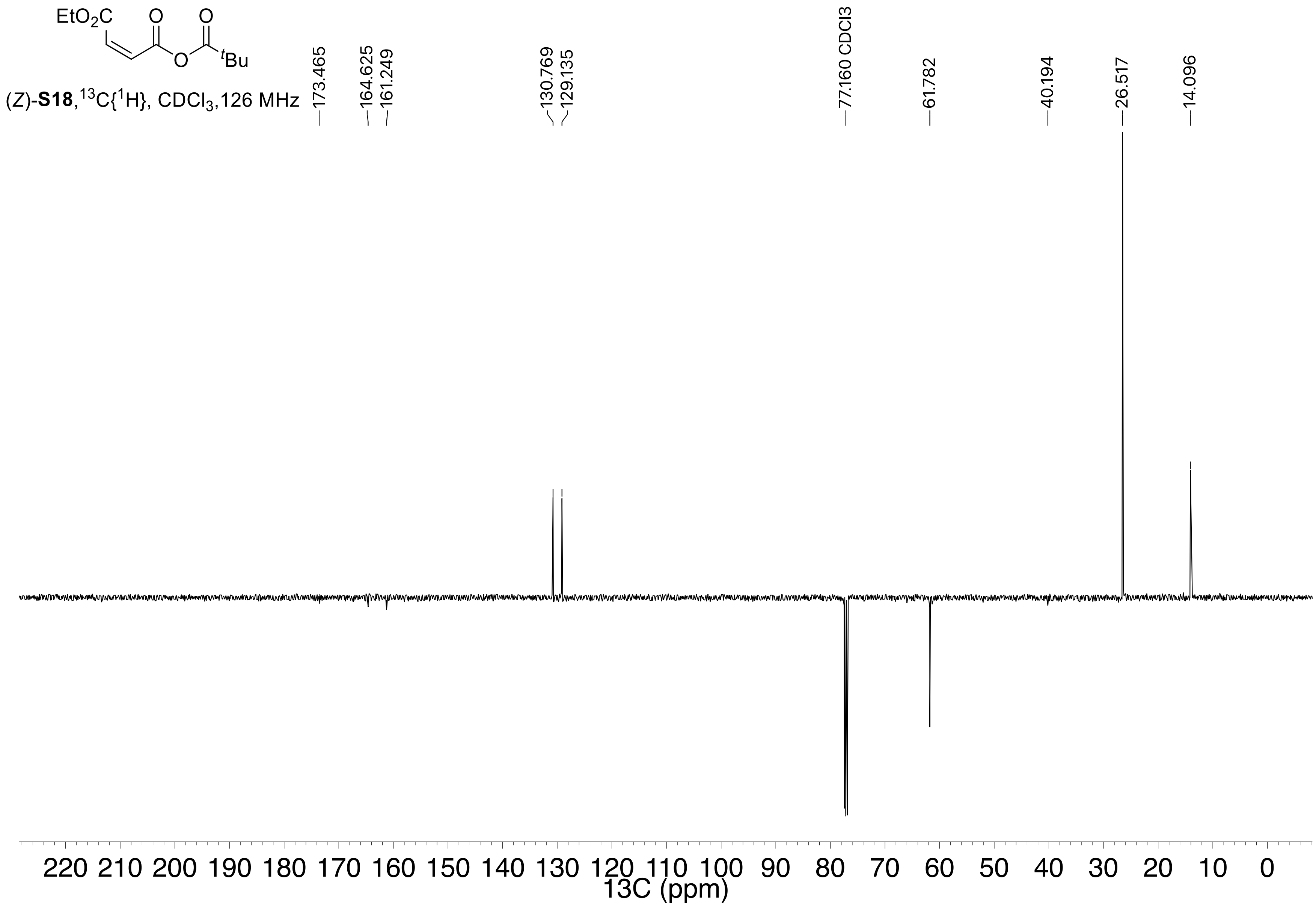

EtO2C
O
O
O
tBu
(Z)-S18, 13C{1H}, CDCl3, 126 MHz
173.465
164.625
161.249
130.769
129.135
77.160 CDCl3
61.782
40.194
26.517
14.096
220 210 200 190 180 170 160 150 140 130 120 110 100 90 80 70 60 50 40 30 20 10 0
13C (ppm)

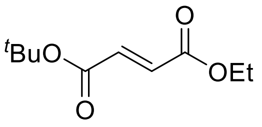

S19, ^{1}H, $CDCl_3$, 400 MHz

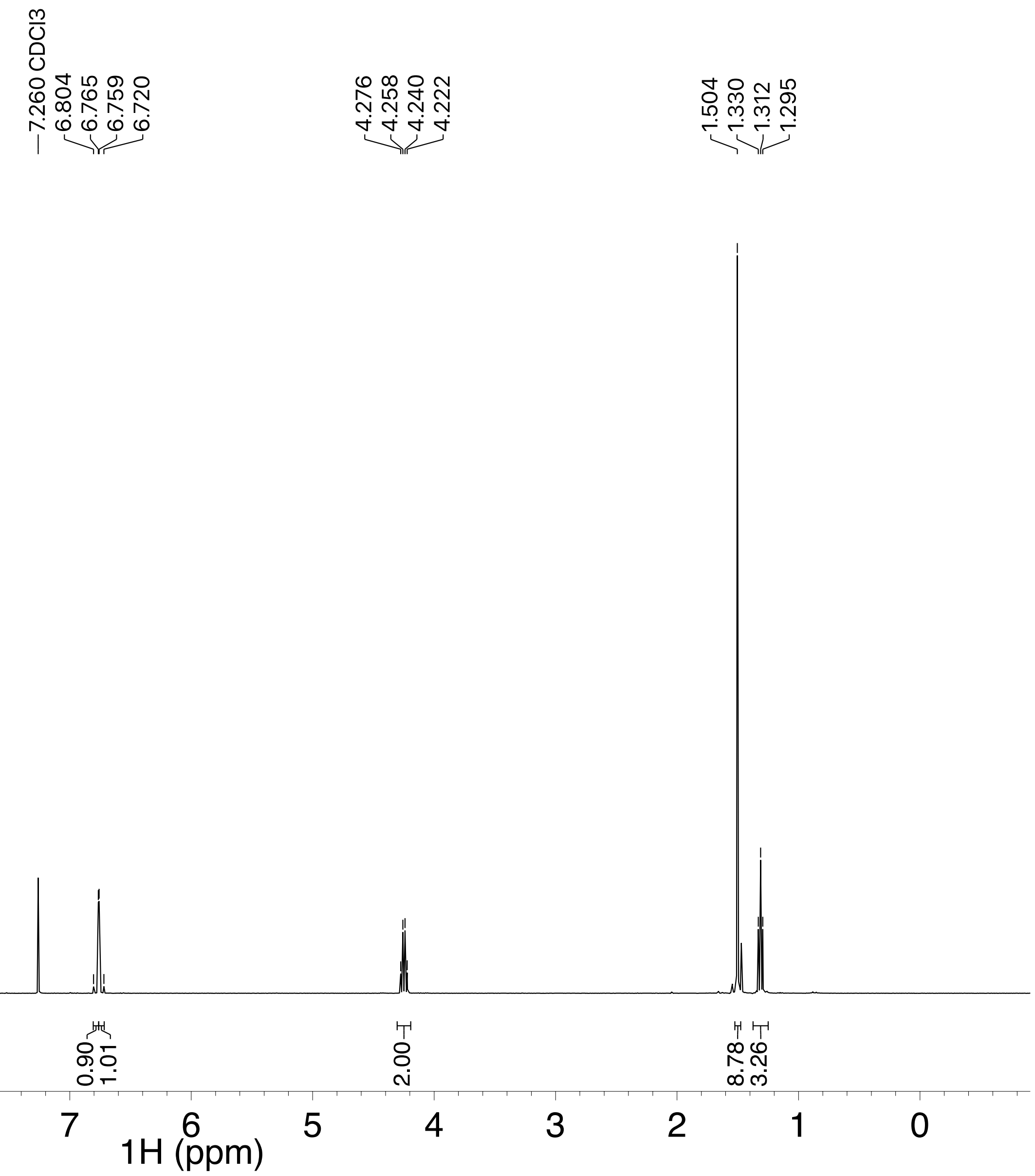

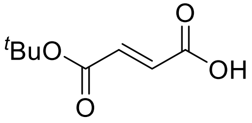

S20, ^{1}H, $CDCl_3$, 400 MHz

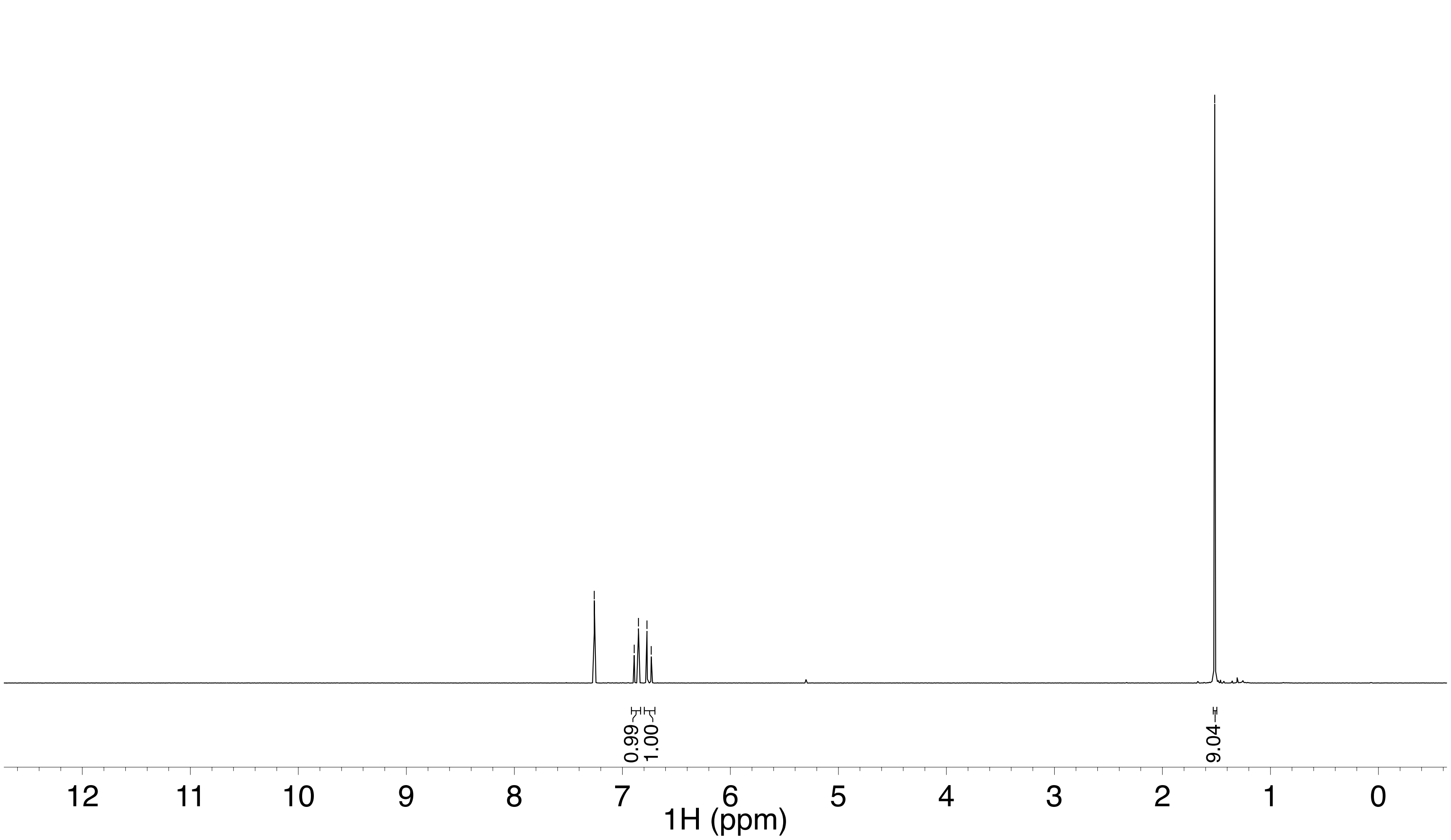

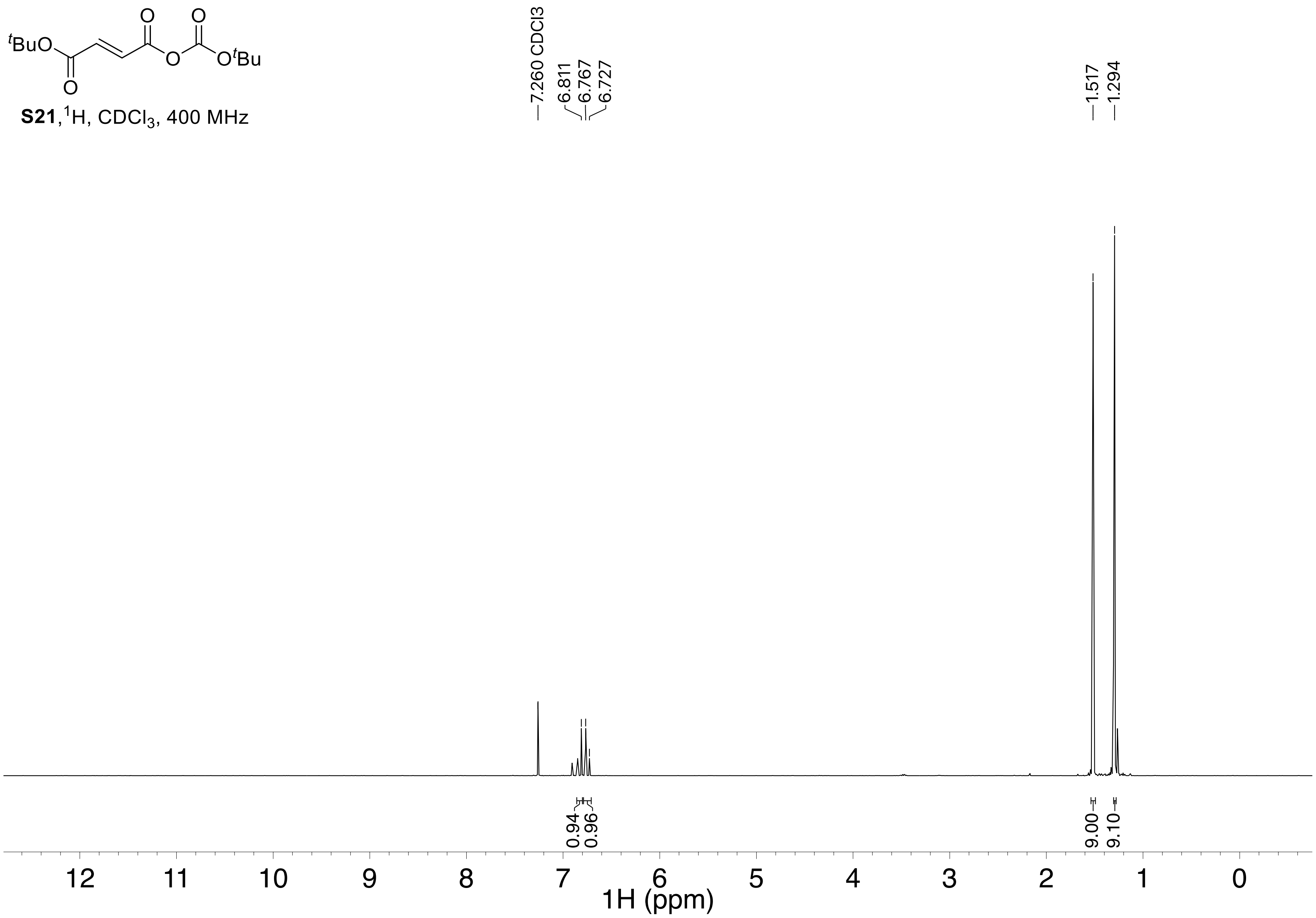

S21, ^{1}H, $CDCl_3$, 400 MHz

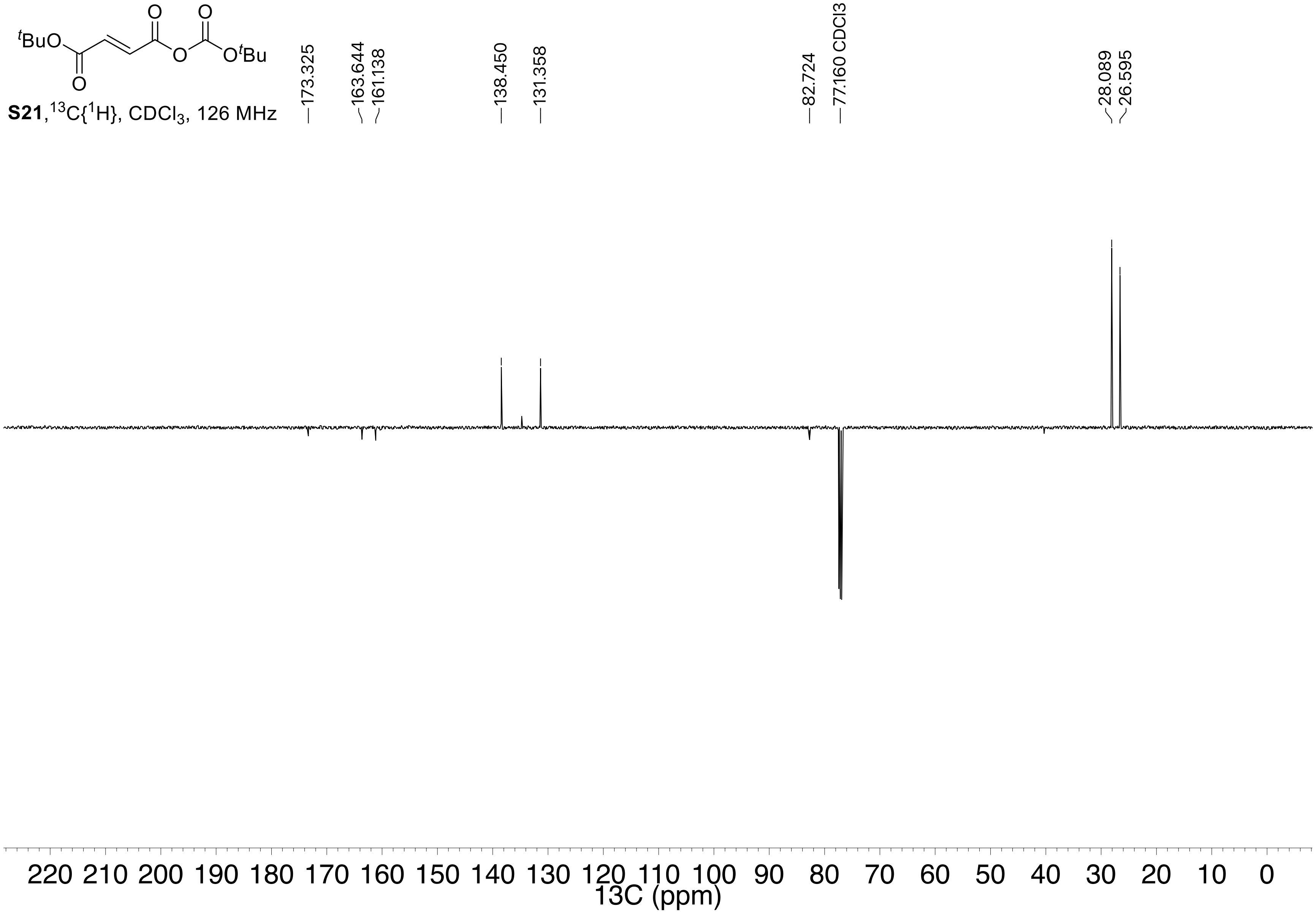

S21, $^{13}C\{^{1}H\}$, $CDCl_3$, 126 MHz

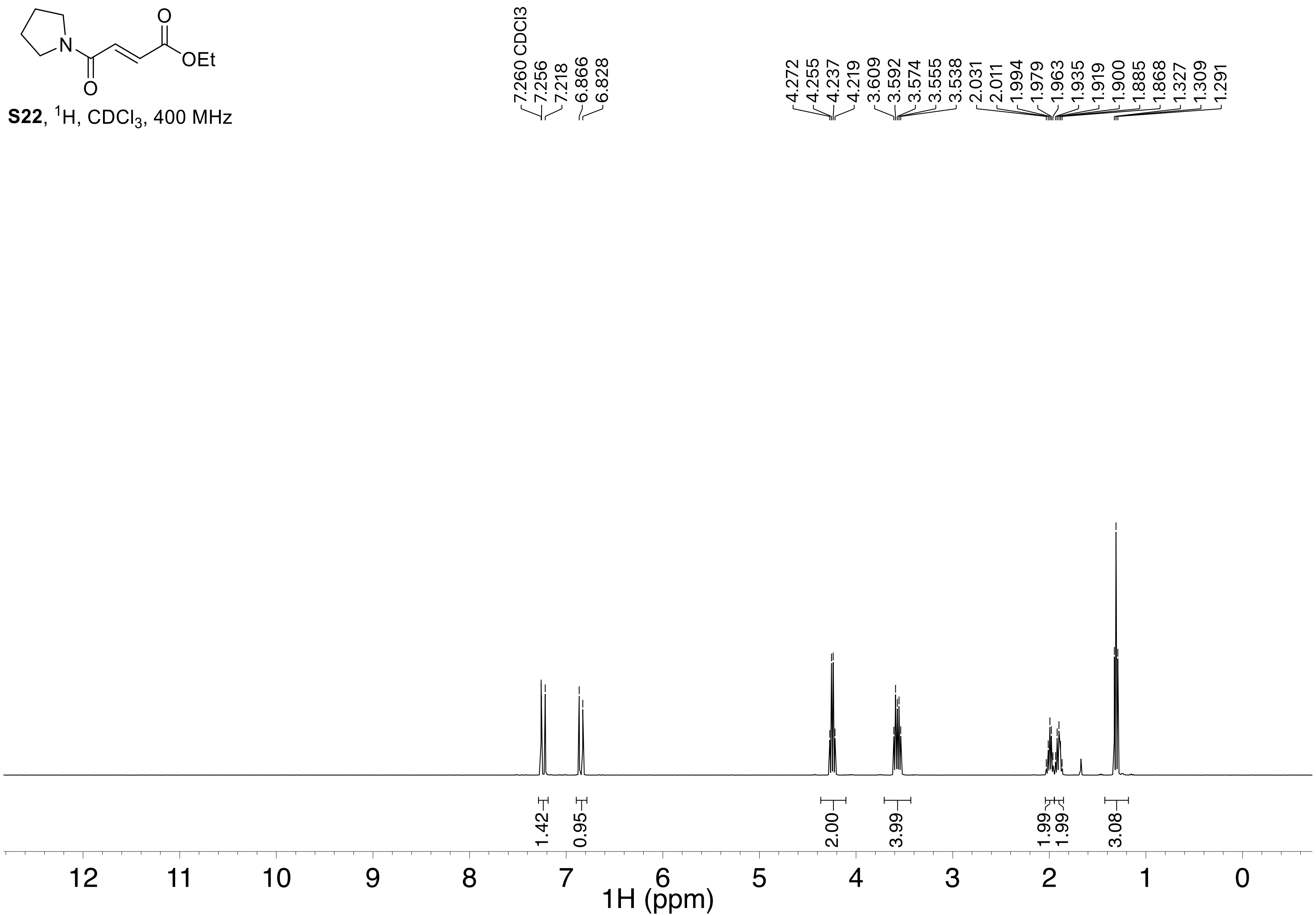

S22, ^{1}H, $CDCl_3$, 400 MHz

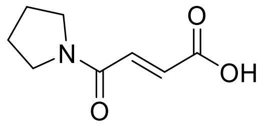

S23, ^{1}H, $CDCl_3$, 500 MHz

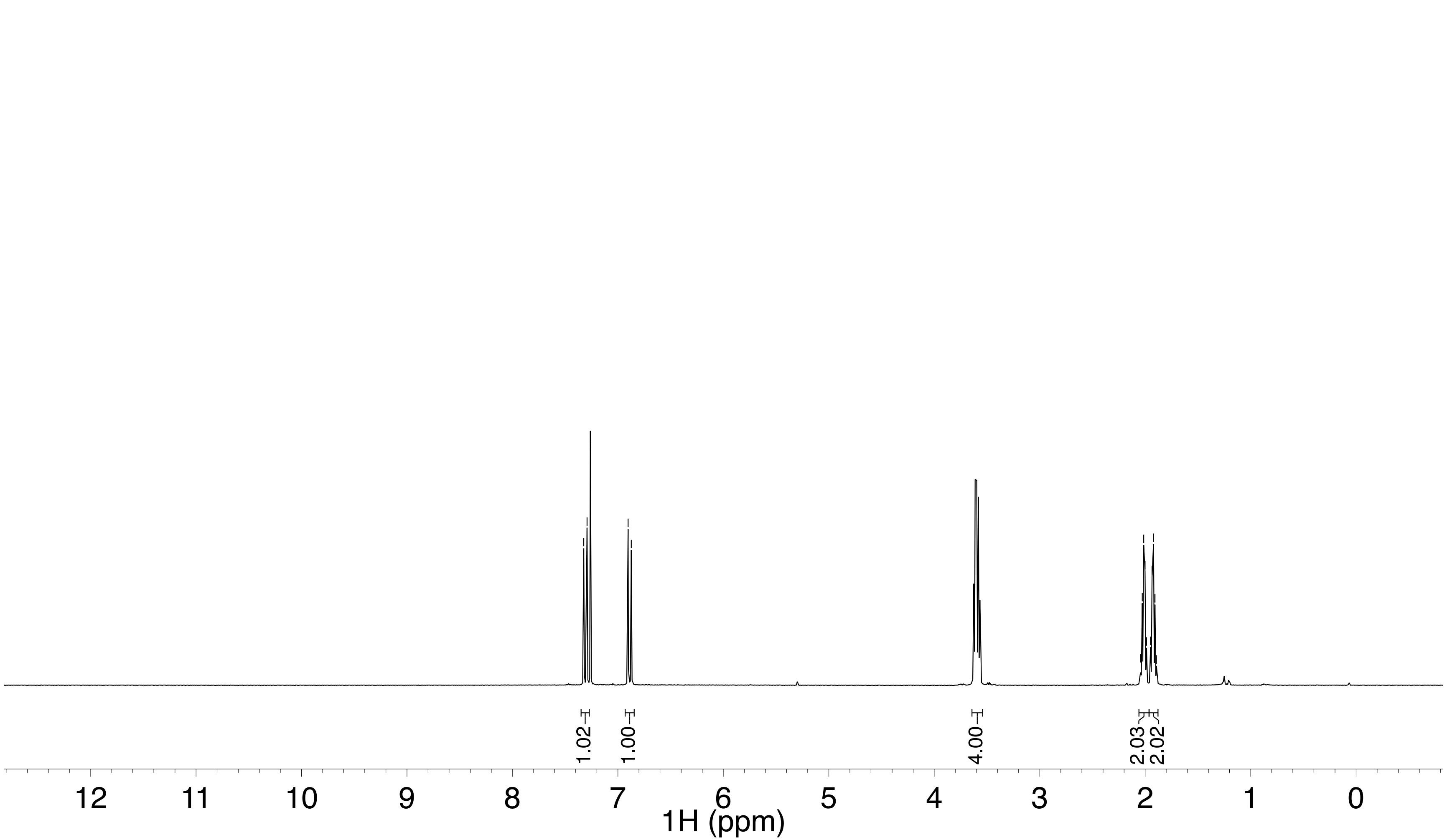

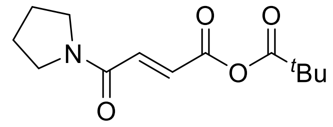

S24, ^{1}H, $CDCl_3$, 400 MHz

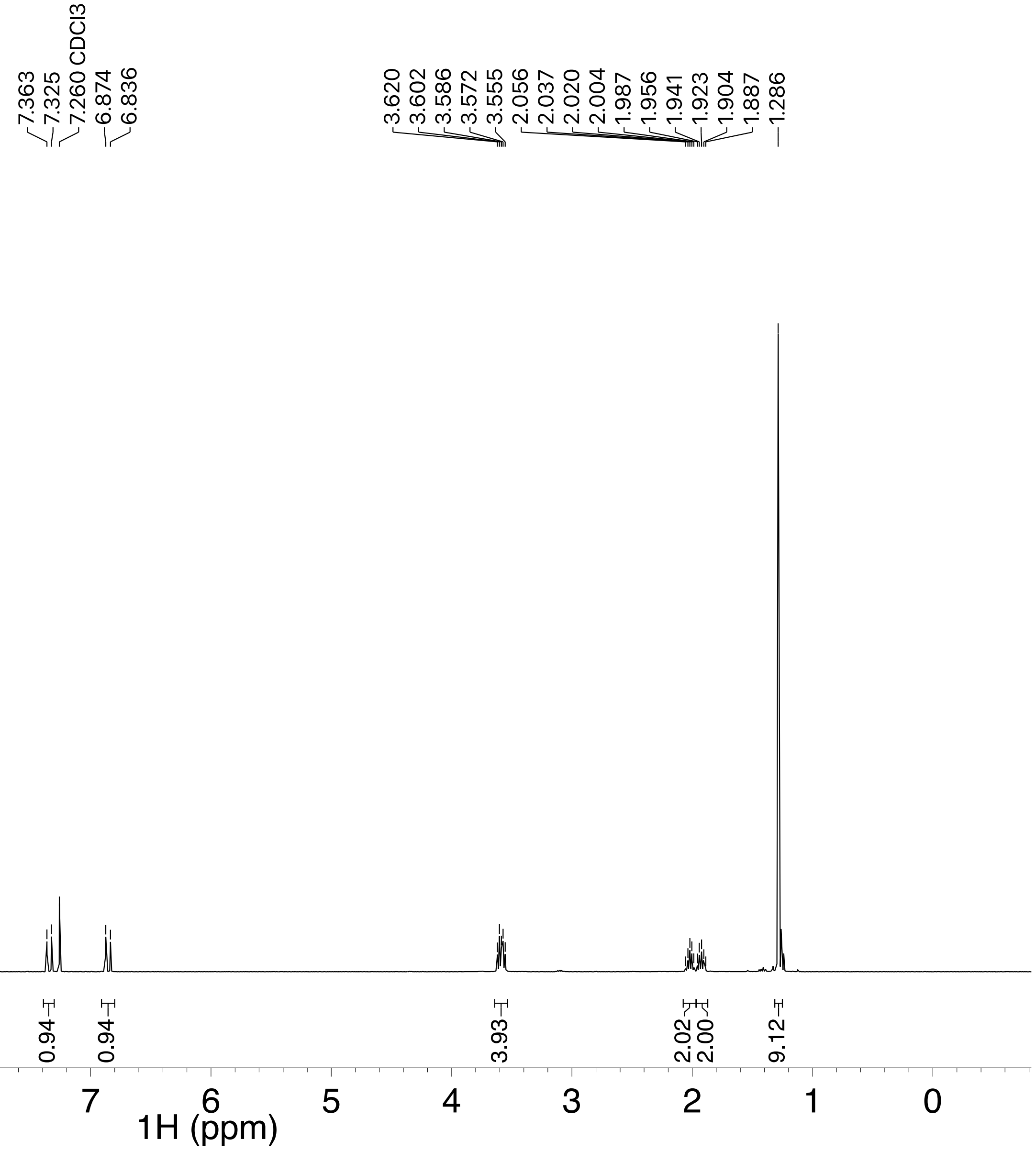

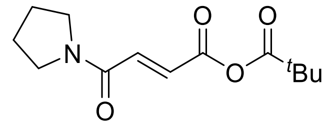

S24, $^{13}C\{^{1}H\}$, $CDCl_3$, 101 MHz

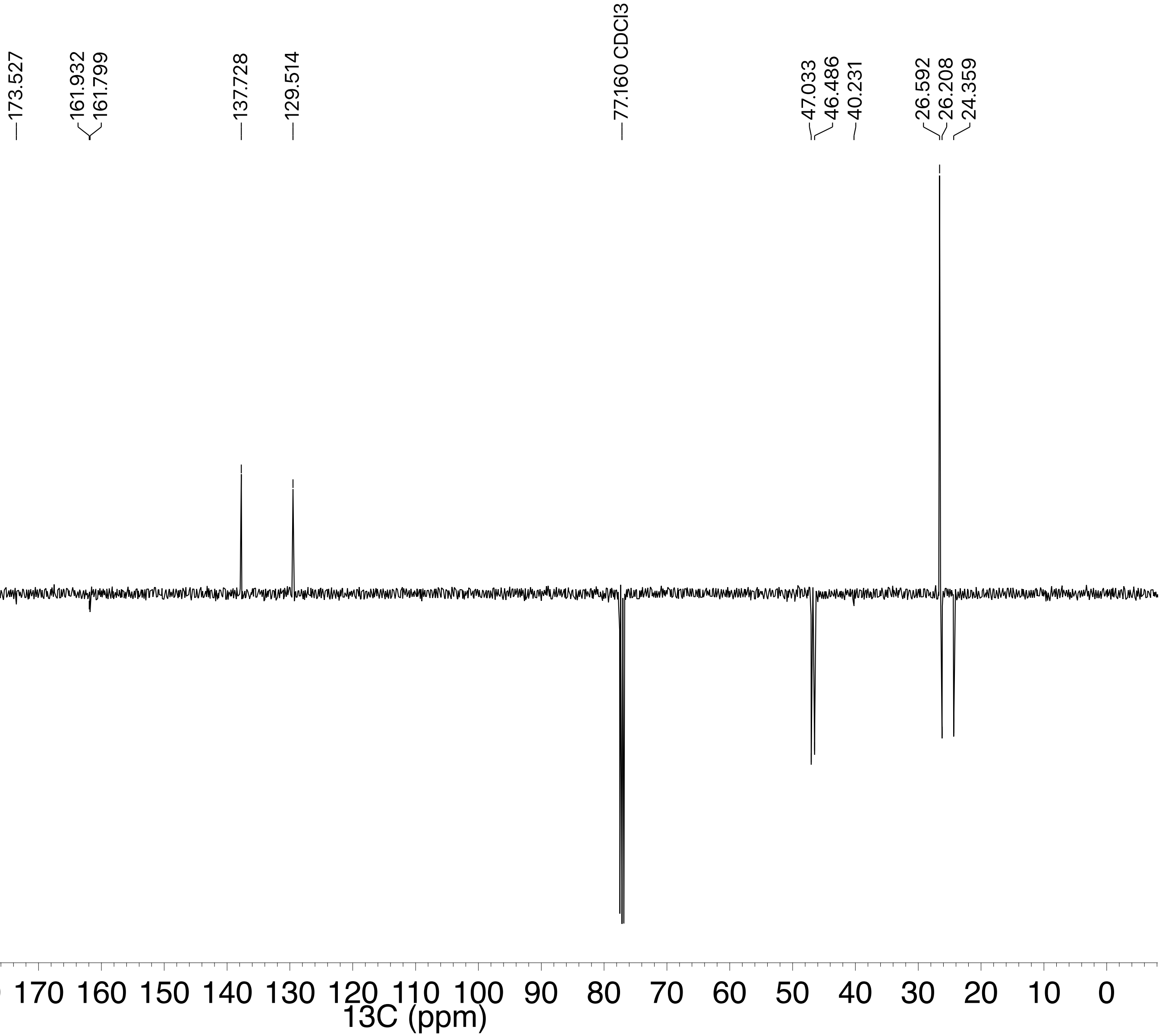

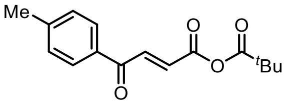

S25, ^{1}H, $CDCl_3$, 400 MHz

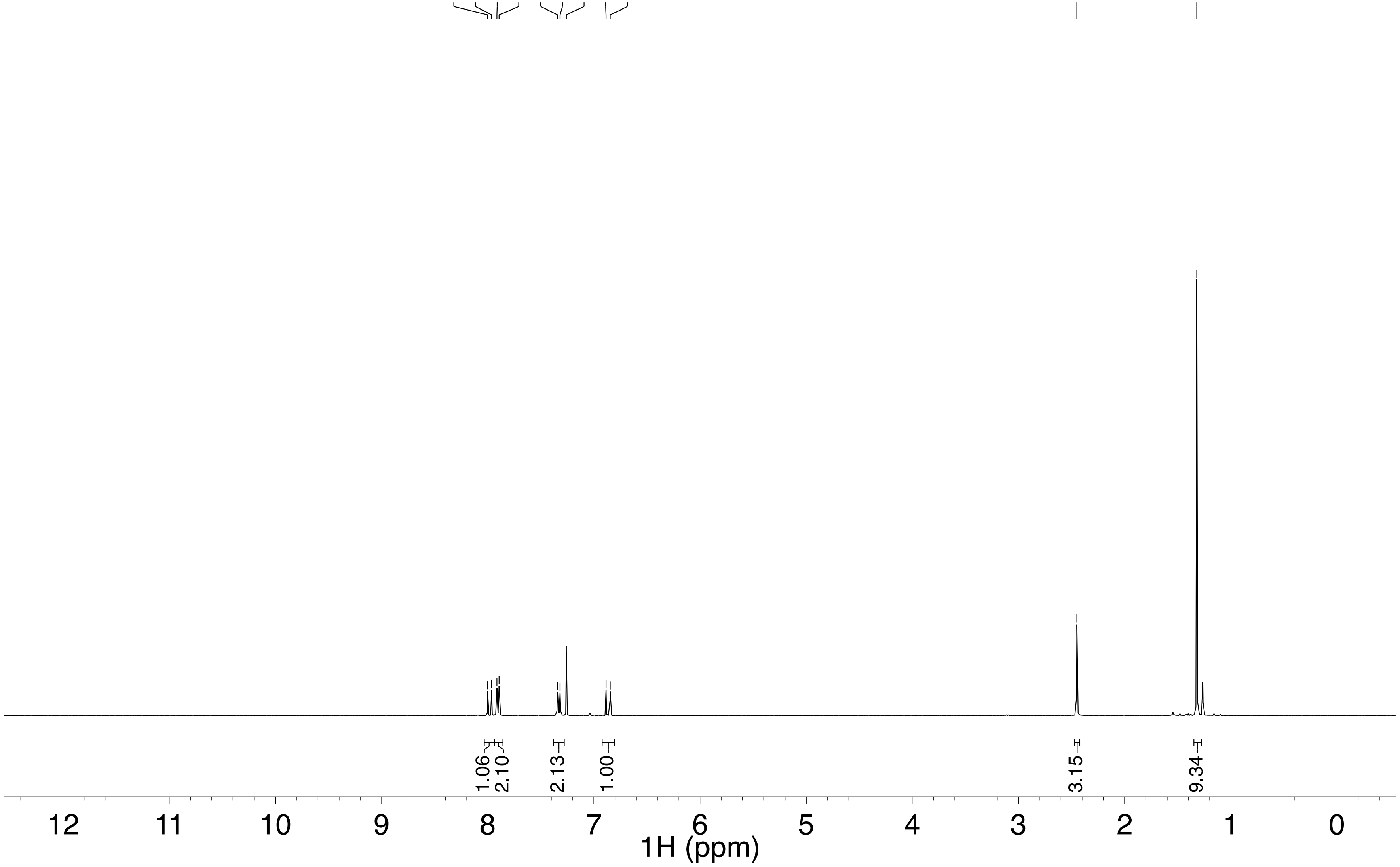

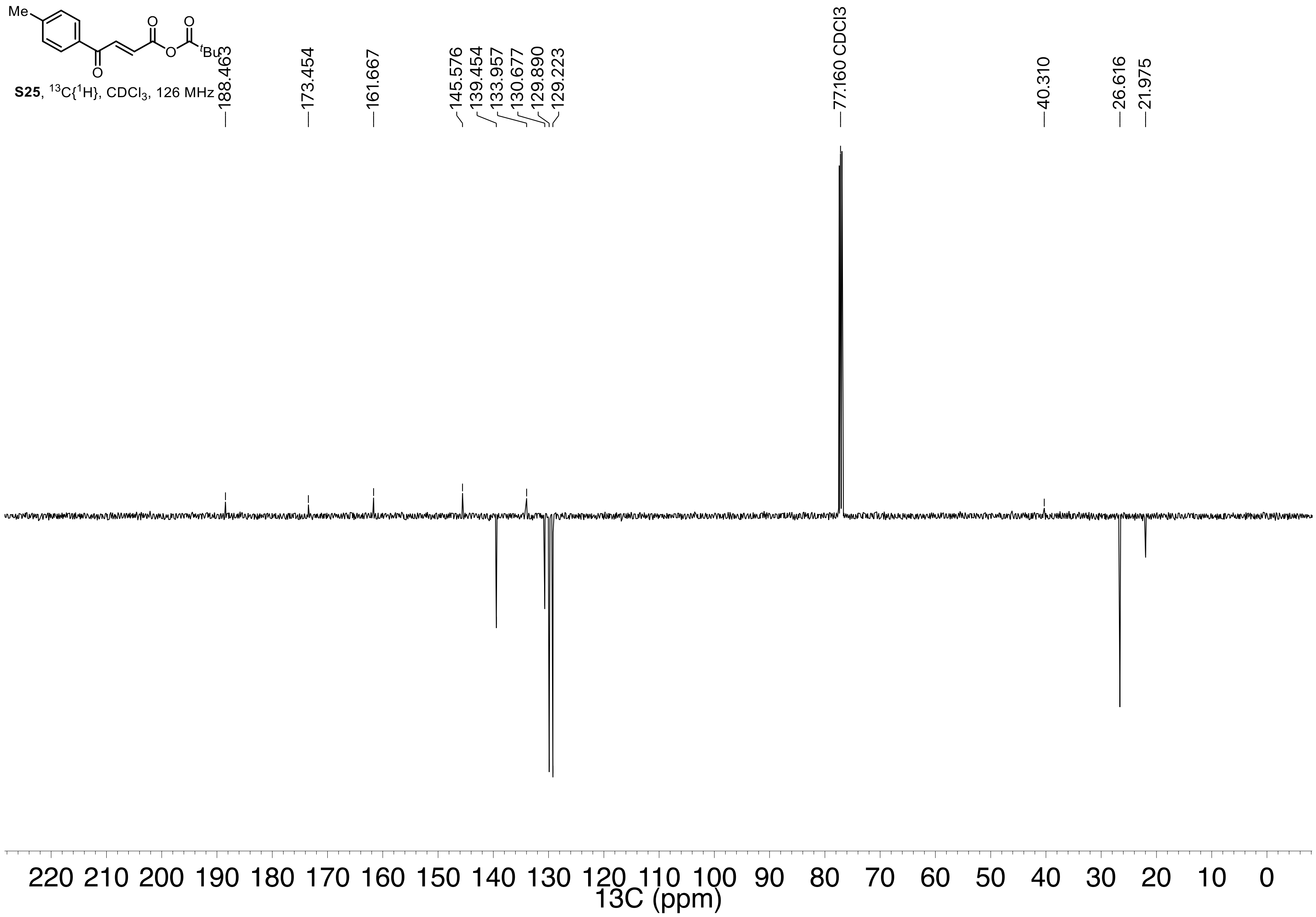
Me
O
O
O
O
tBu
S25, ¹³C{¹H}, CDCl₃, 126 MHz
188.463
173.454
161.667
145.576
139.454
133.957
130.677
129.890
129.223
77.160 CDCl3
40.310
26.616
21.975
220 210 200 190 180 170 160 150 140 130 120 110 100 90 80 70 60 50 40 30 20 10 0
13C (ppm)

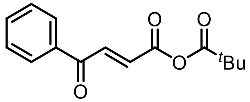

S26, ^{1}H, $CDCl_3$, 400 MHz

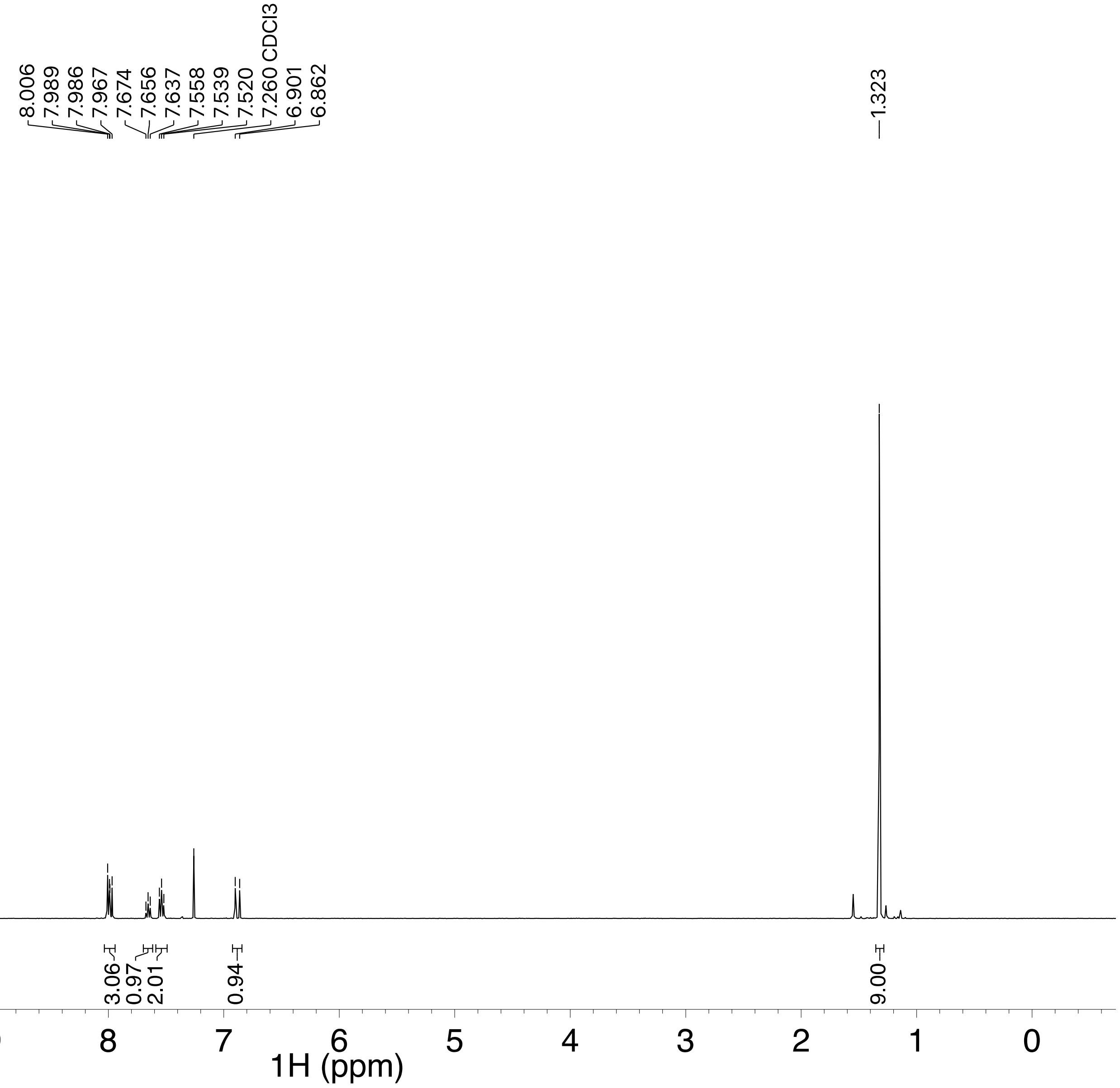

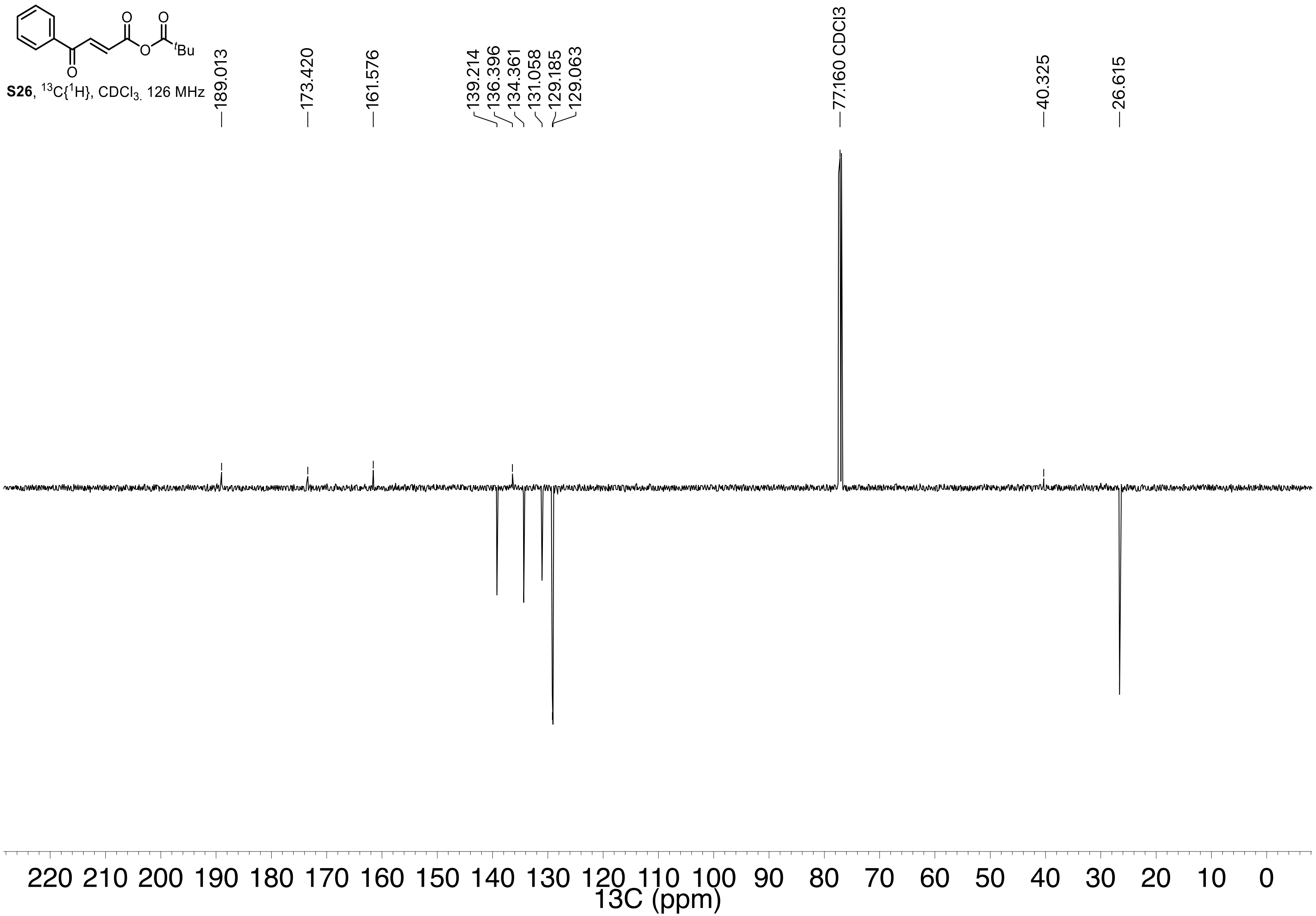
O
O
O
tBu
O
S26, 13C{1H}, CDCl3. 126 MHz
189.013
173.420
161.576
139.214
136.396
134.361
131.058
129.185
129.063
77.160 CDCl3
40.325
26.615
220 210 200 190 180 170 160 150 140 130 120 110 100 90 80 70 60 50 40 30 20 10 0
13C (ppm)

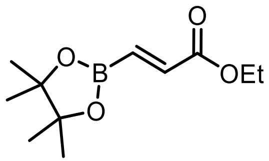

S27, ^{1}H, $CDCl_3$, 400 MHz

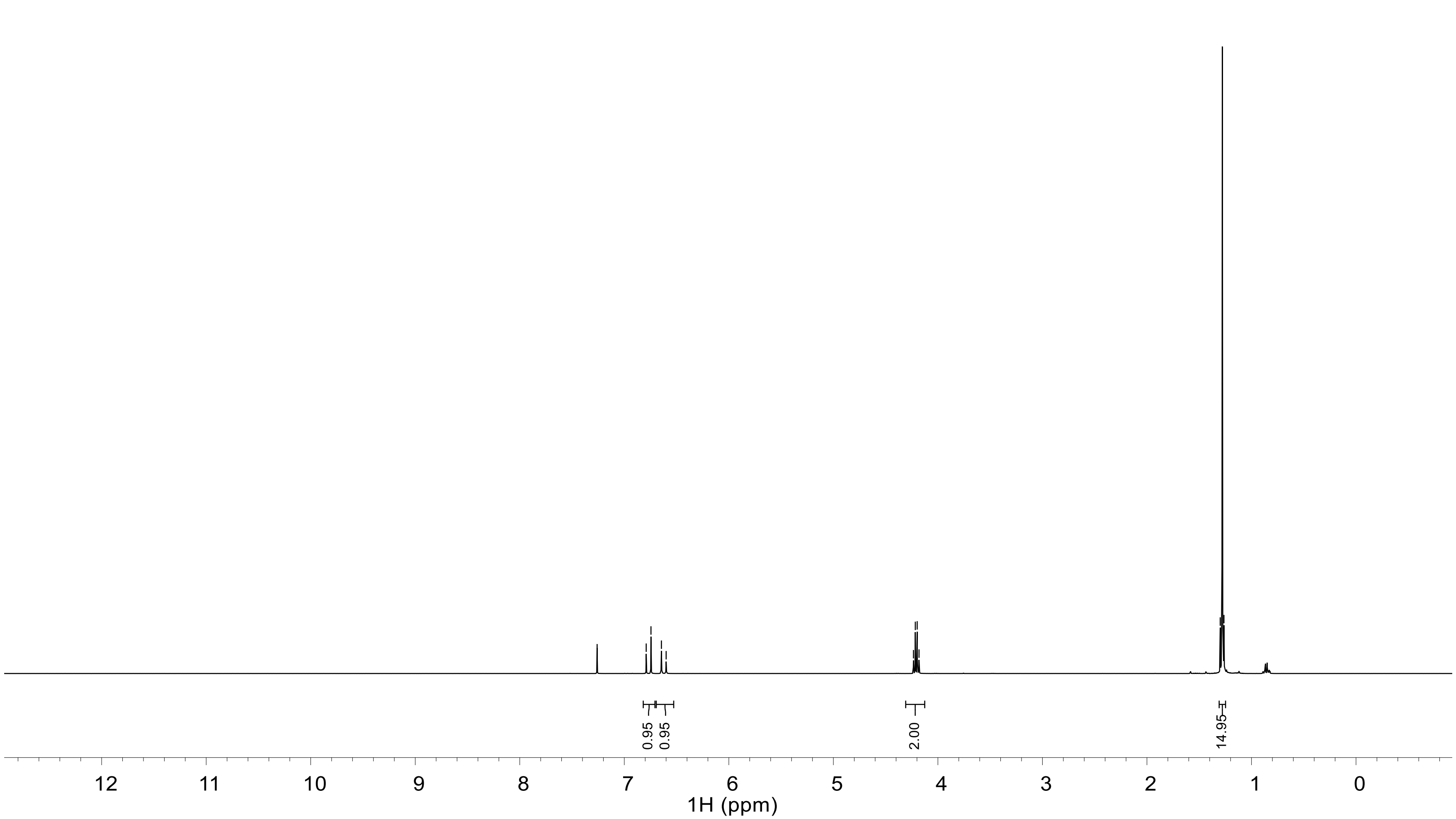

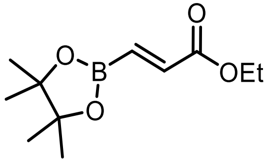

S27, $^{11}B\{^{1}H\}$, $CDCl_3$, 128 MHz

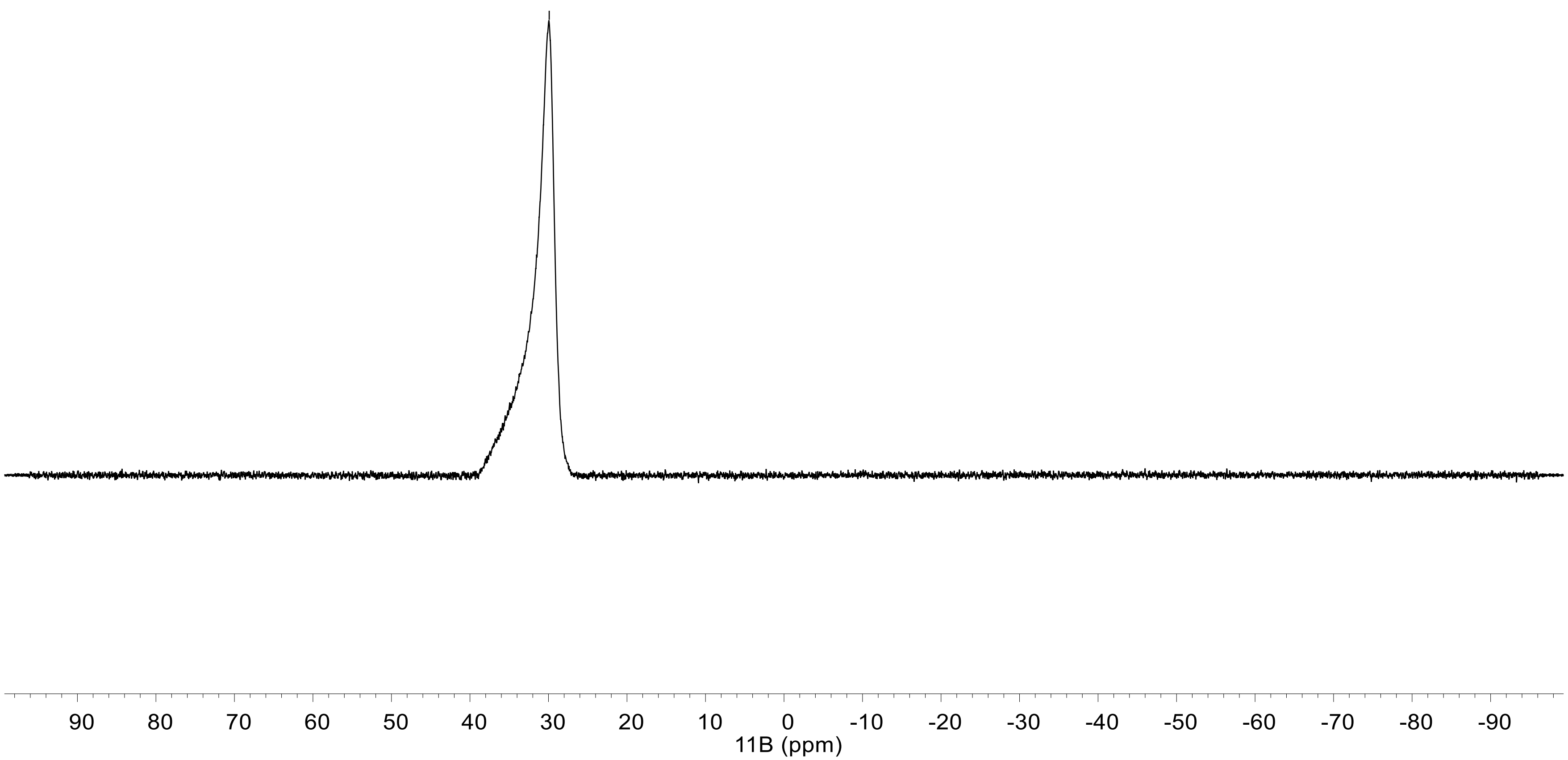

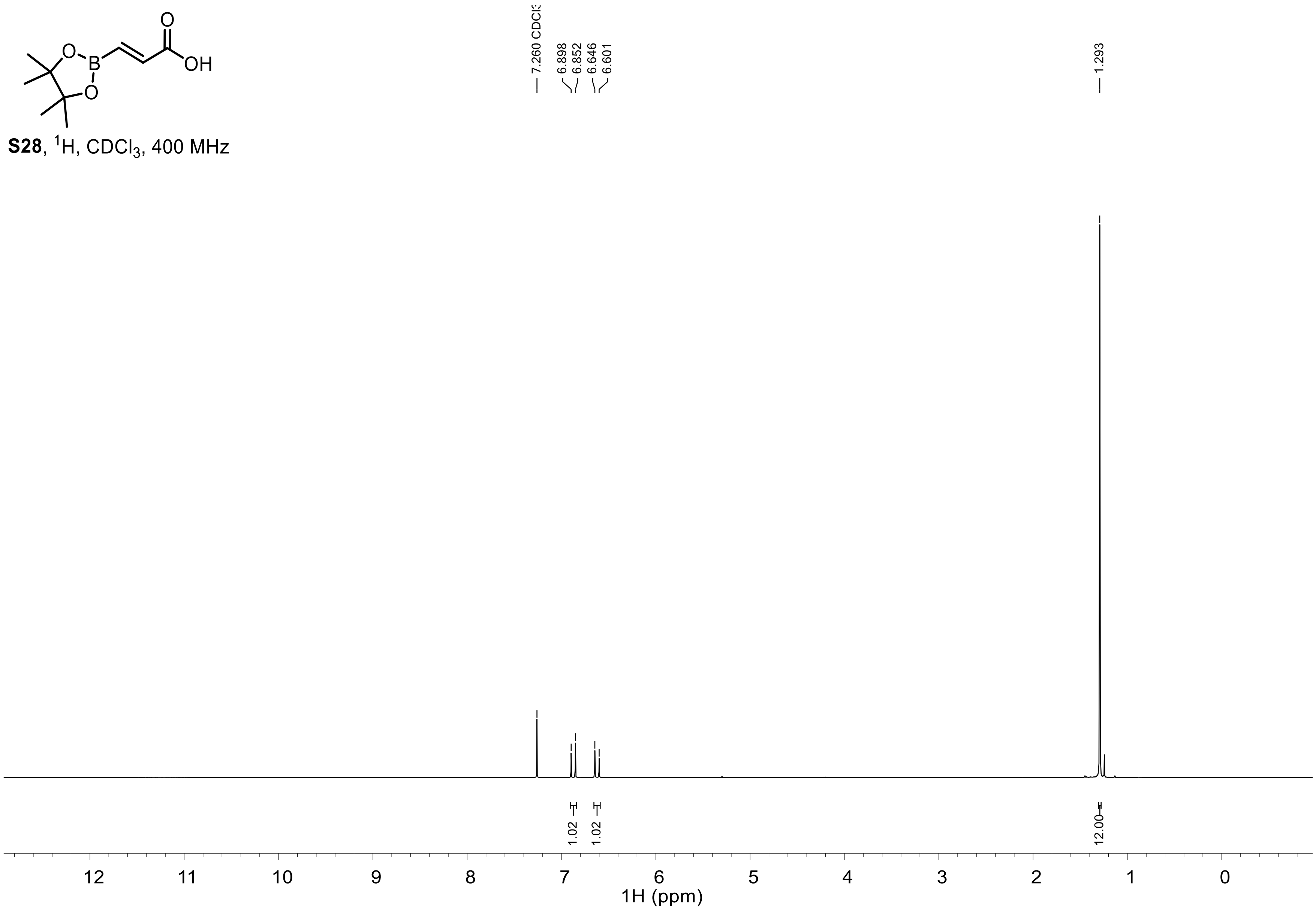

S28, ^{1}H, $CDCl_3$, 400 MHz

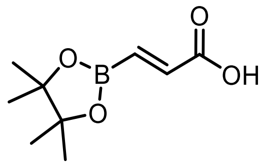

S28, $^{13}C\{^1H\}$, $CDCl_3$, 126 MHz

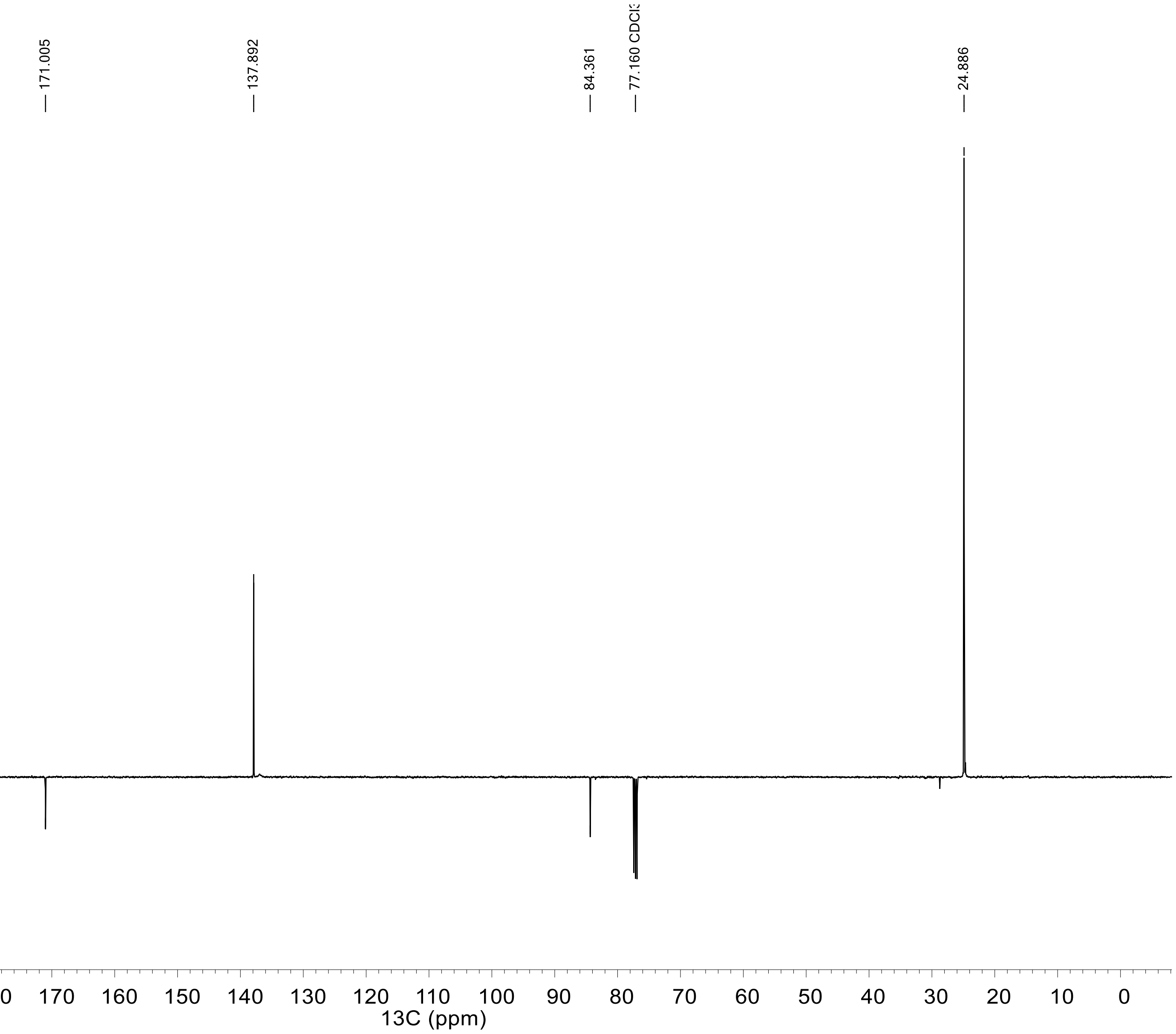

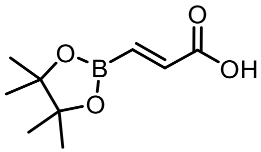

S28, $^{11}B\{^{1}H\}$, $CDCl_3$, 128 MHz

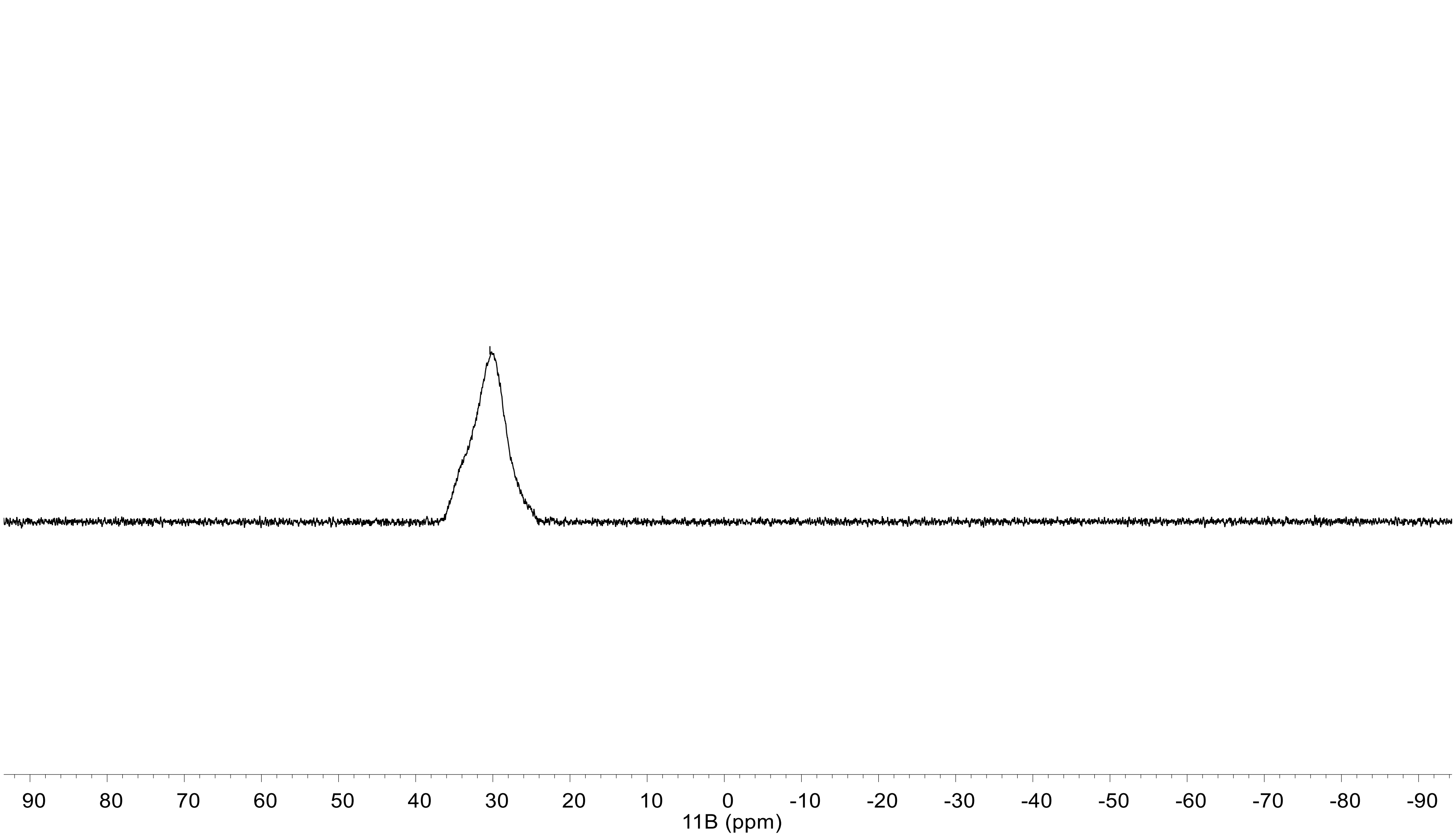

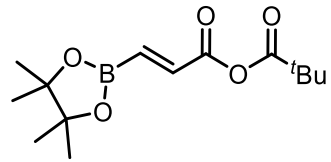

S6, ^{1}H, $CDCl_3$, 400 MHz

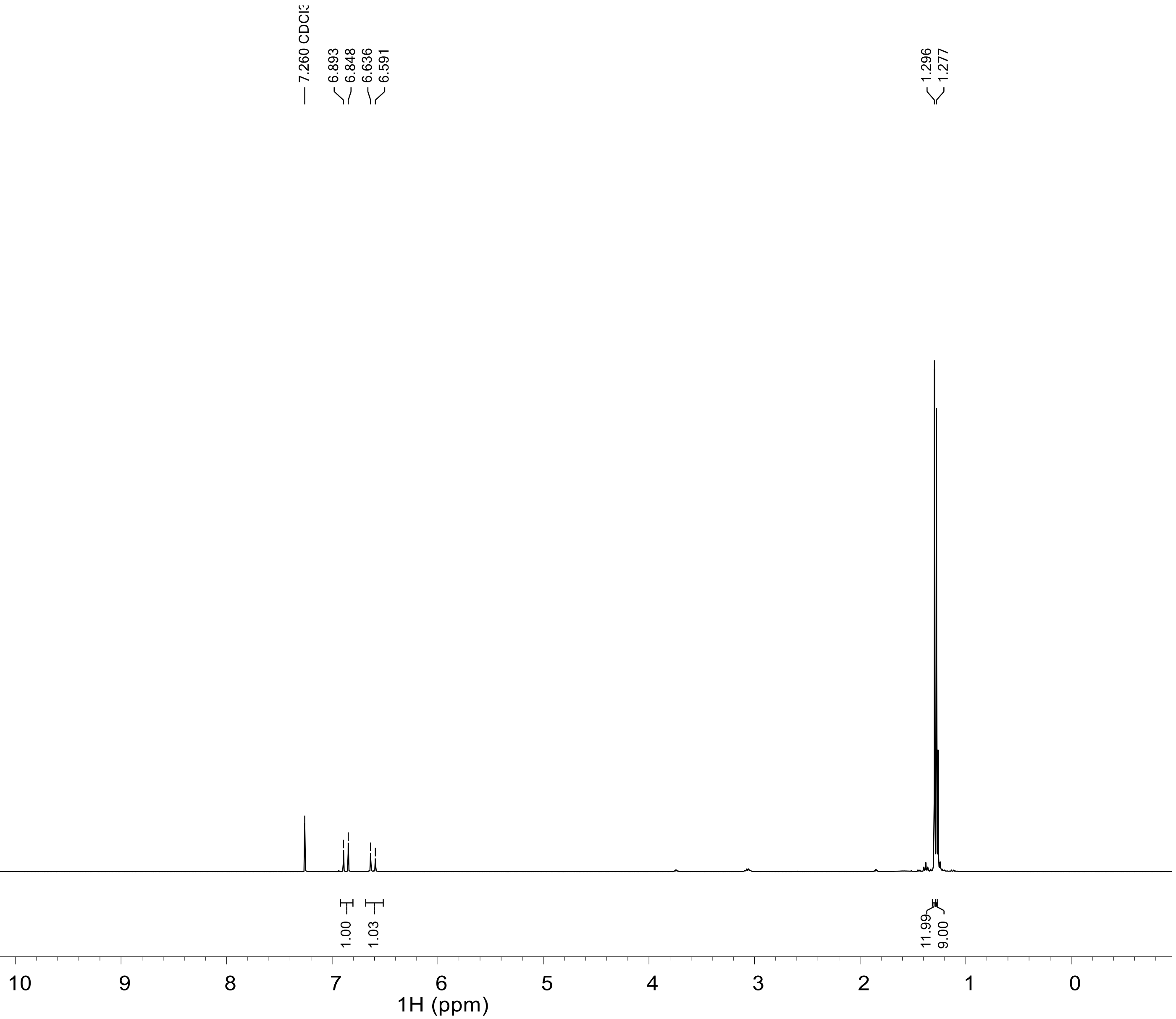

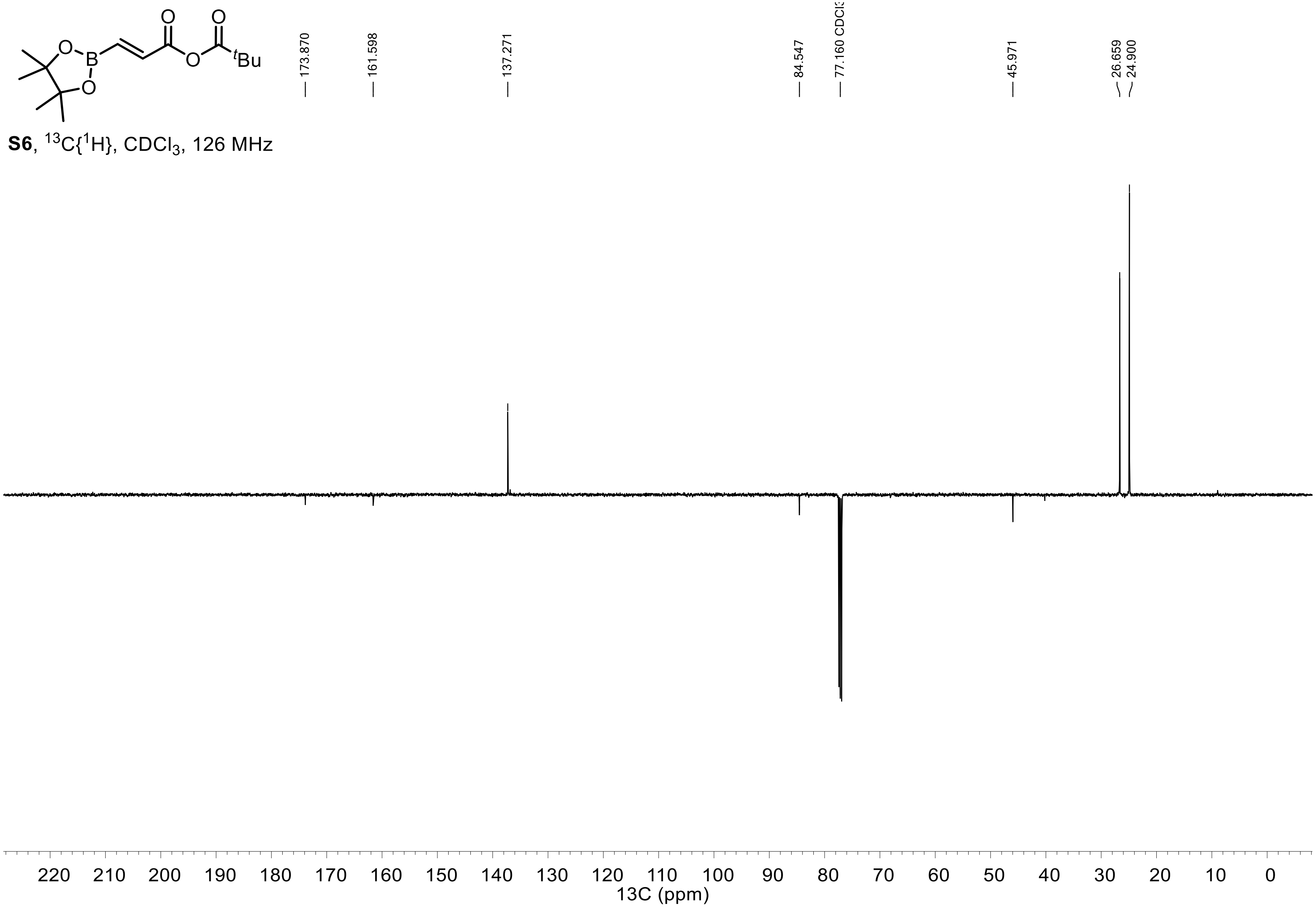

S6, $^{13}C\{^{1}H\}$, $CDCl_3$, 126 MHz

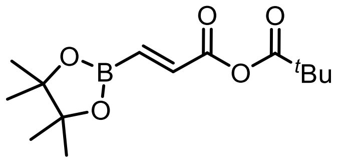

S6, $^{11}B\{^{1}H\}$, $CDCl_3$, 128 MHz

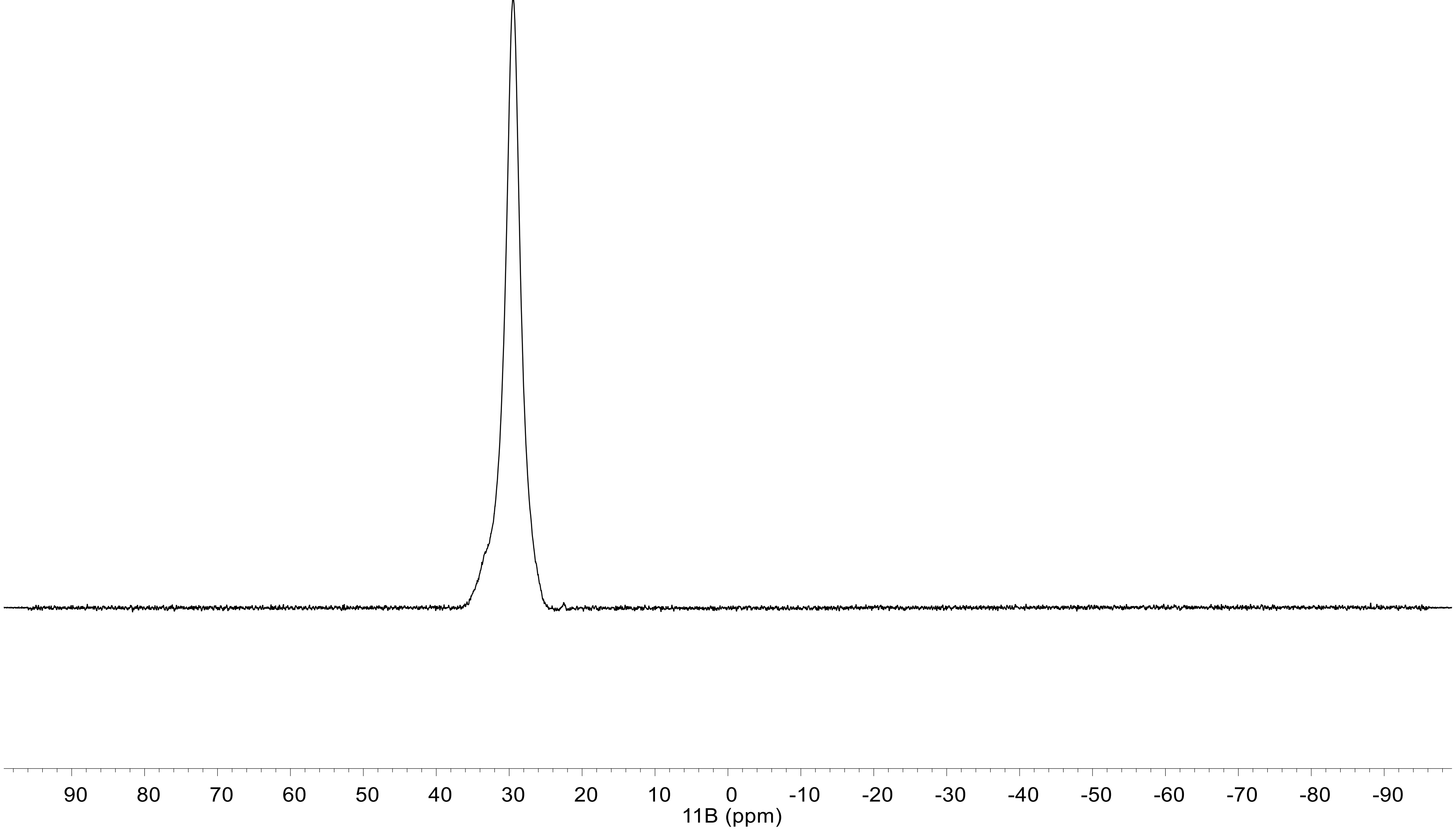

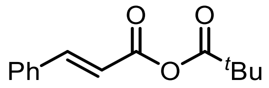

42, ^{1}H, $CDCl_3$, 300 MHz

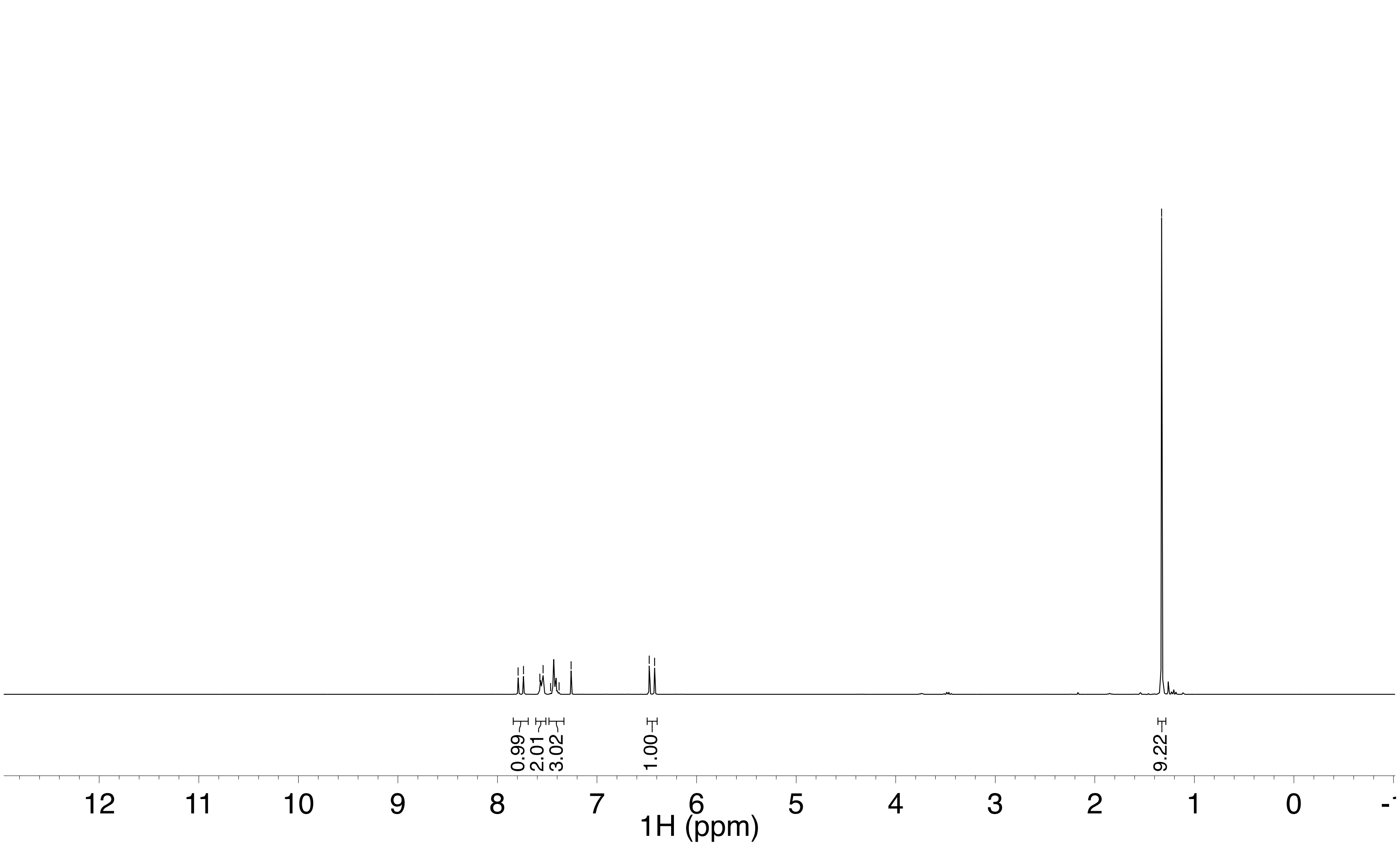

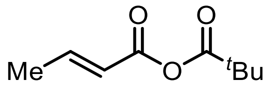

S9, ^{1}H, $CDCl_3$, 500 MHz

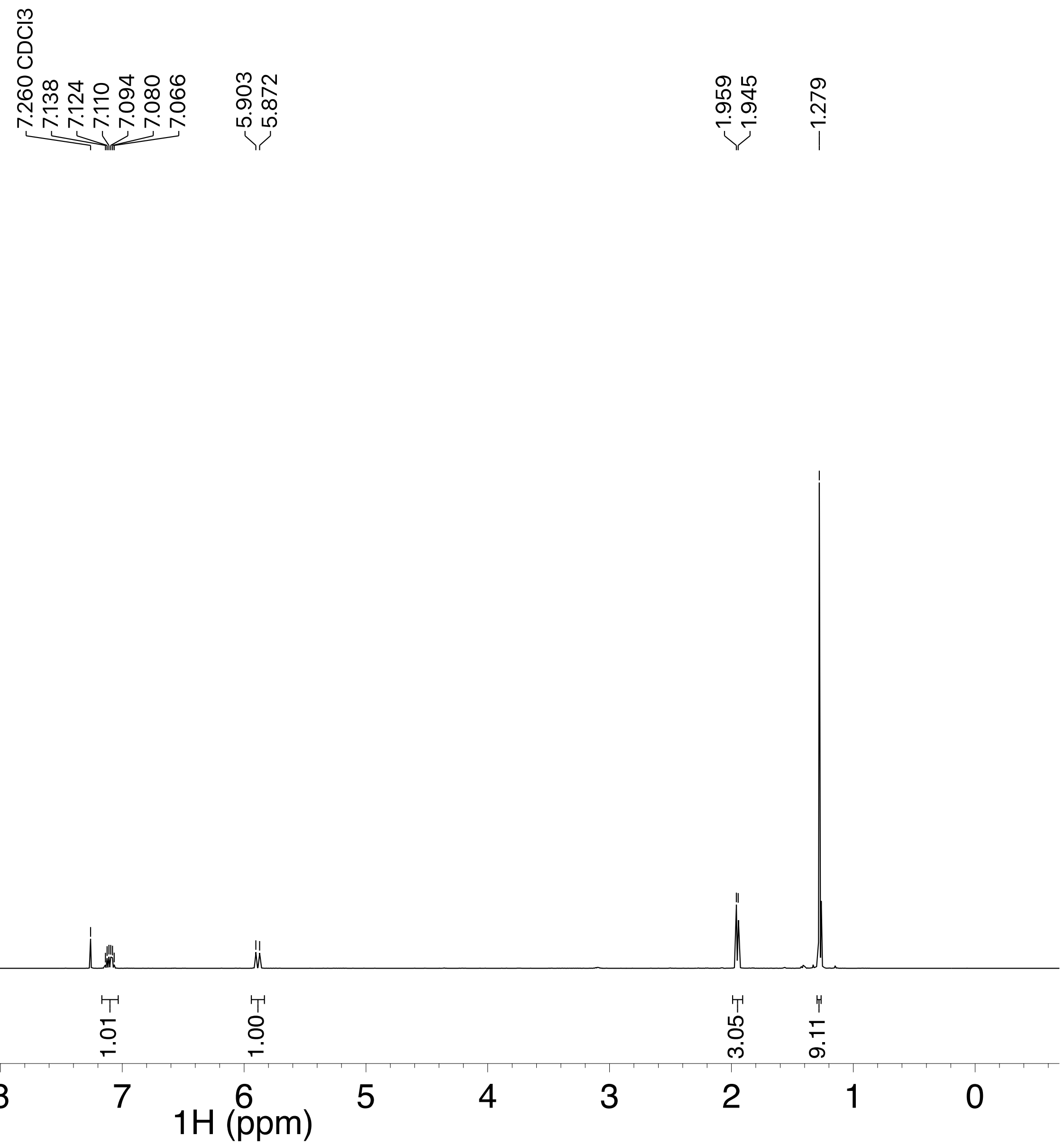

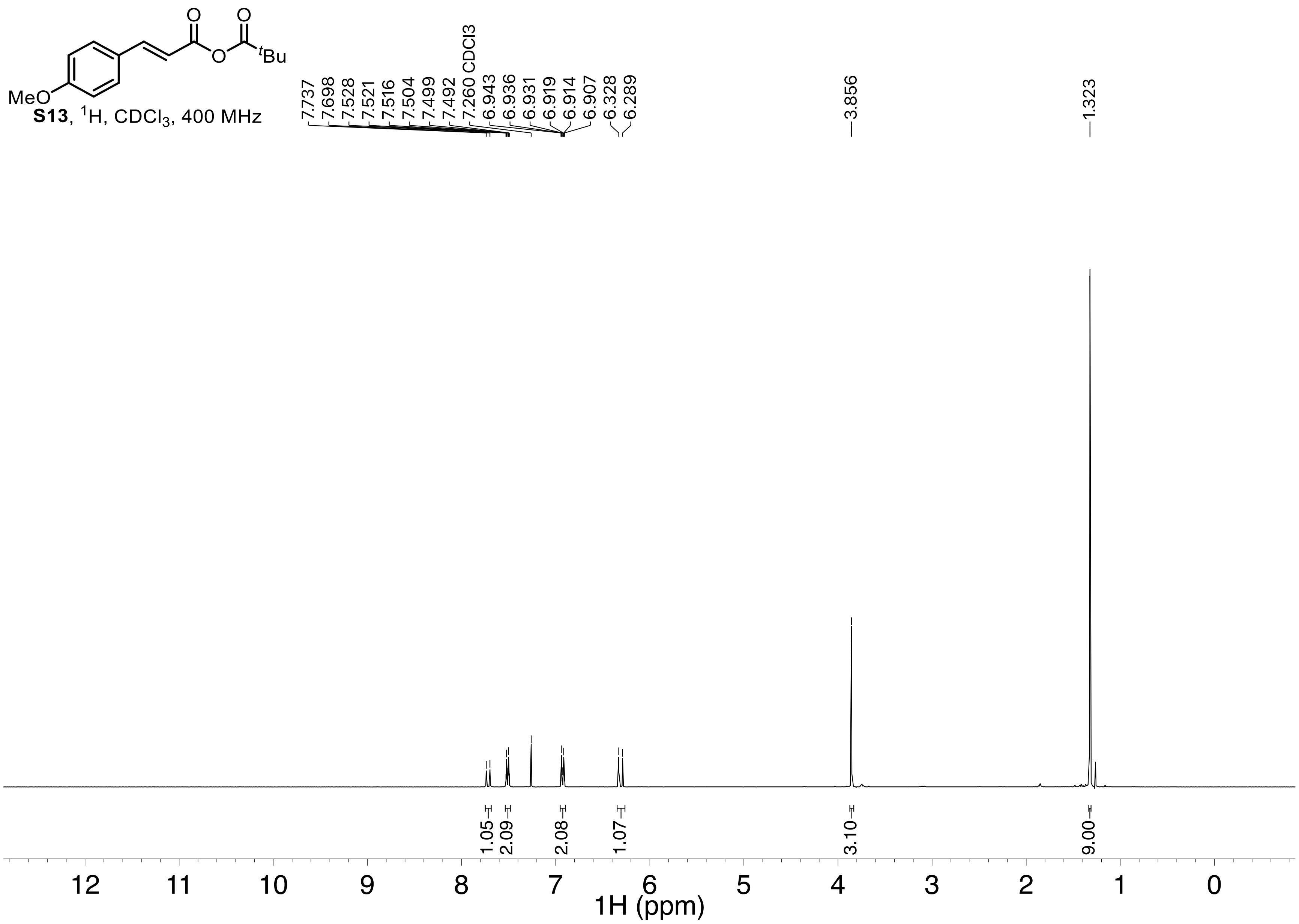
MeO
tBu
S13, 1H, CDCl3, 400 MHz
7.737
7.698
7.528
7.521
7.516
7.504
7.499
7.492
7.260 CDCl3
6.943
6.936
6.931
6.919
6.914
6.907
6.328
6.289
3.856
1.323
1.05
2.09
2.08
1.07
3.10
9.00
12 11 10 9 8 7 6 5 4 3 2 1 0
1H (ppm)

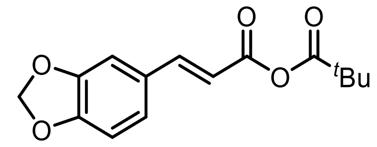

S29, ^{1}H, $CDCl_3$, 400 MHz

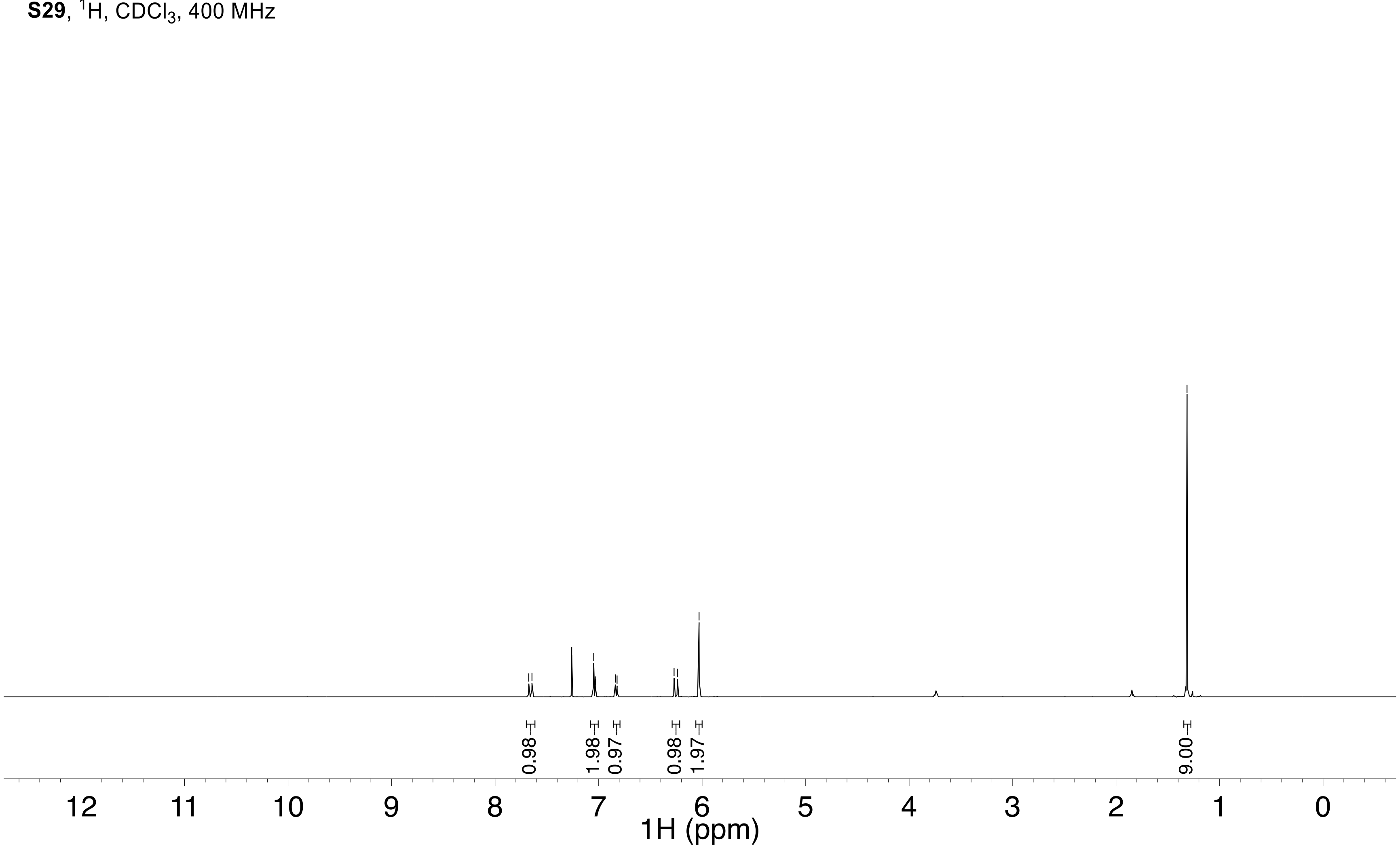

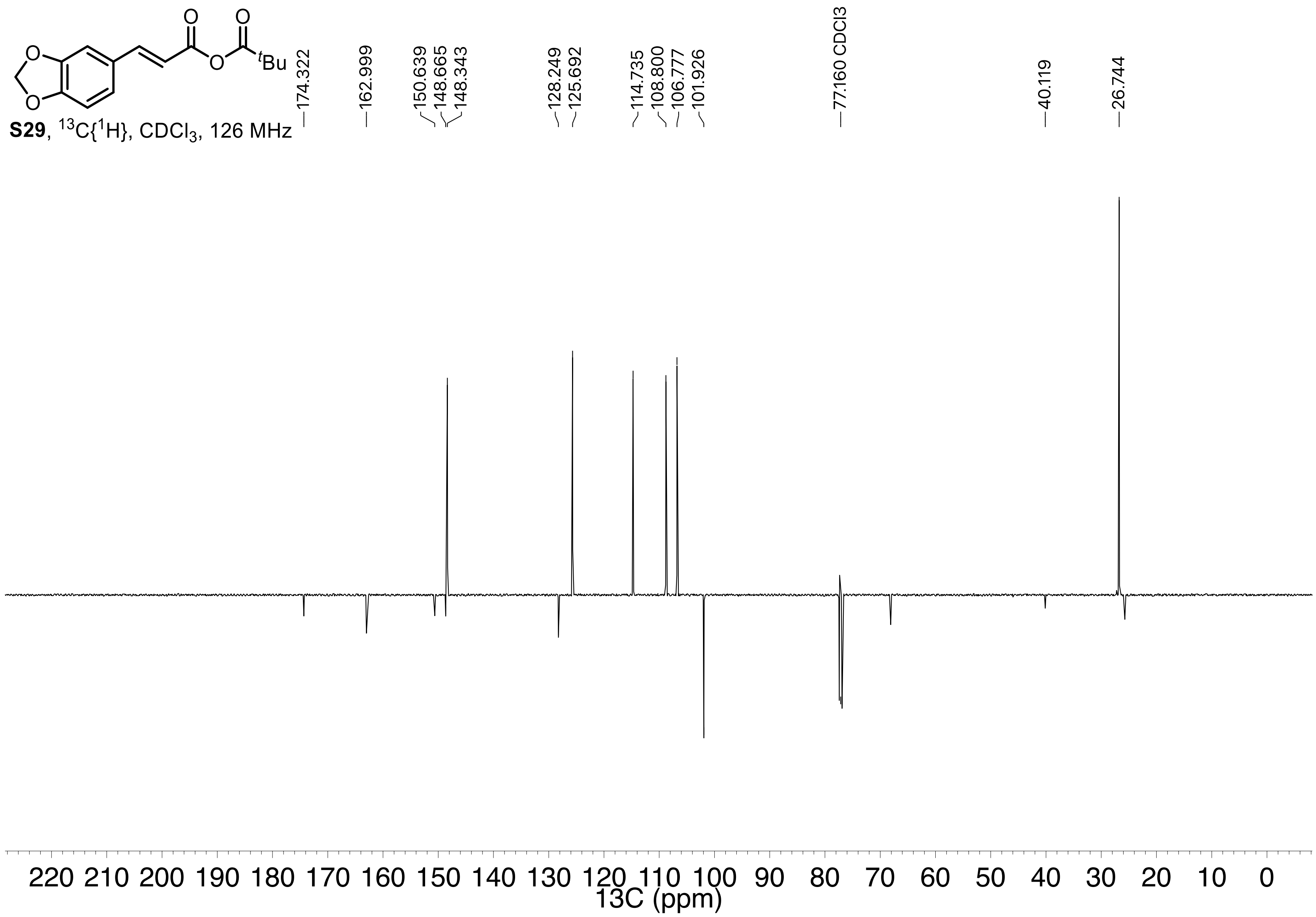

O
O
O
O
O
tBu
S29, 13C{1H}, CDCl3, 126 MHz
174.322
162.999
150.639
148.665
148.343
128.249
125.692
114.735
108.800
106.777
101.926
77.160 CDCl3
40.119
26.744
220 210 200 190 180 170 160 150 140 130 120 110 100 90 80 70 60 50 40 30 20 10 0
13C (ppm)

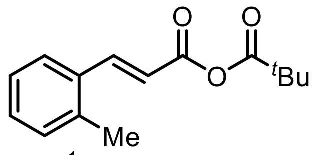

S30, ^{1}H, $CDCl_3$, 400 MHz

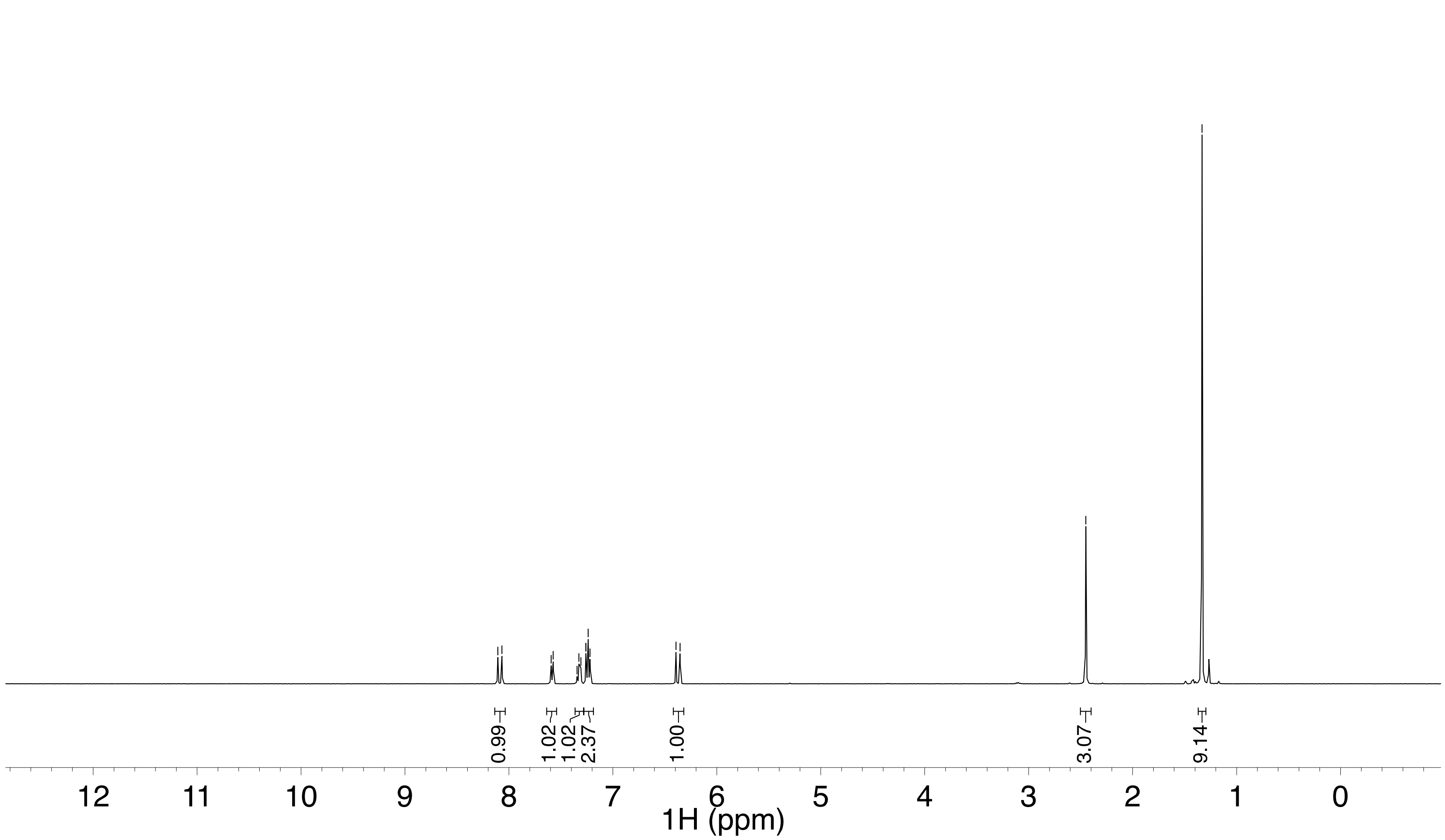

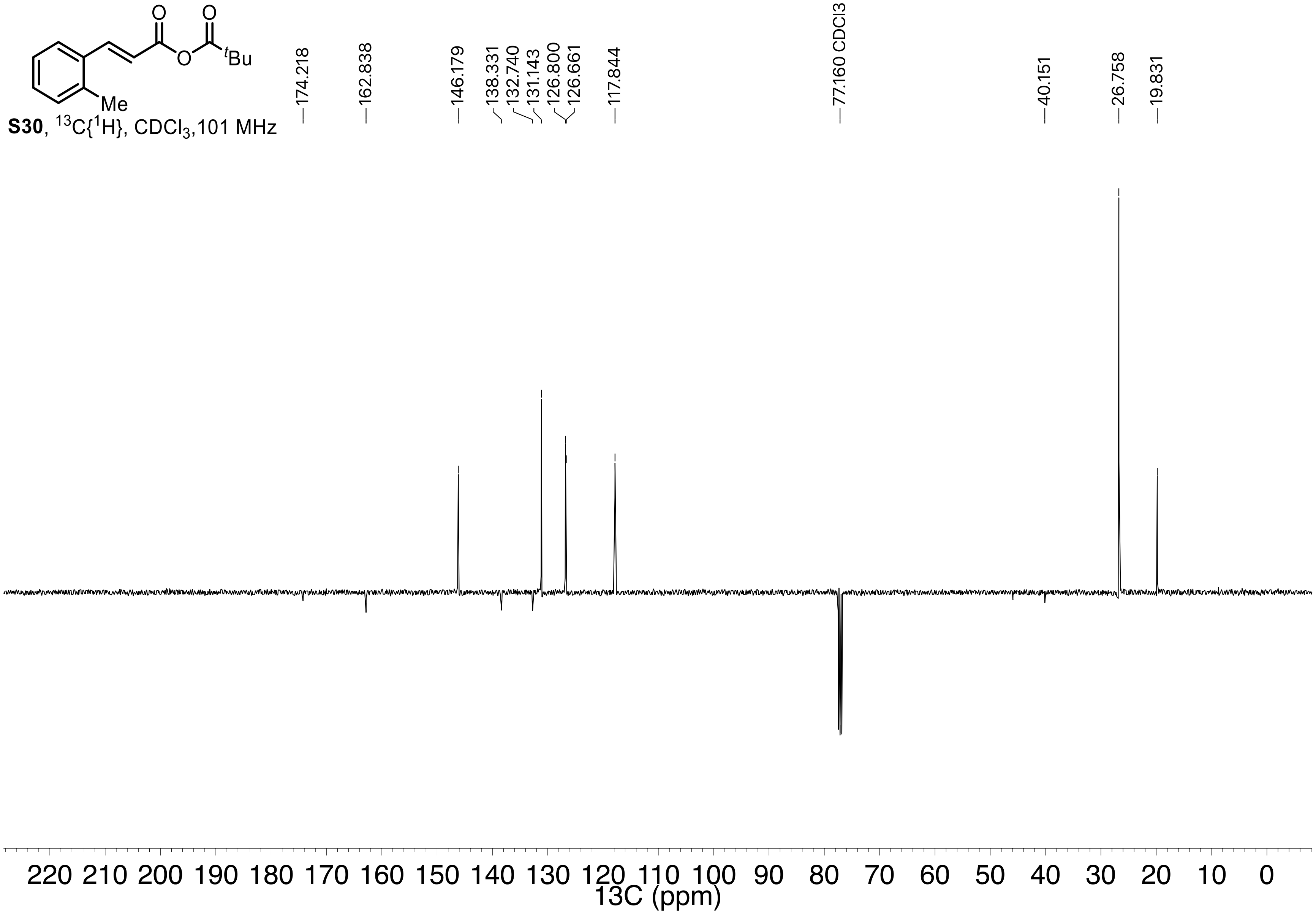

S30, $^{13}C\{^1H\}$, $CDCl_3$,101 MHz
174.218
162.838
146.179
138.331
132.740
131.143
126.800
126.661
117.844
77.160 CDCl3
40.151
26.758
19.831
220 210 200 190 180 170 160 150 140 130 120 110 100 90 80 70 60 50 40 30 20 10 0
13C (ppm)

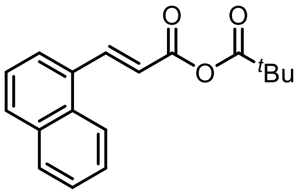

S31, ^{1}H, $CDCl_3$, 400 MHz

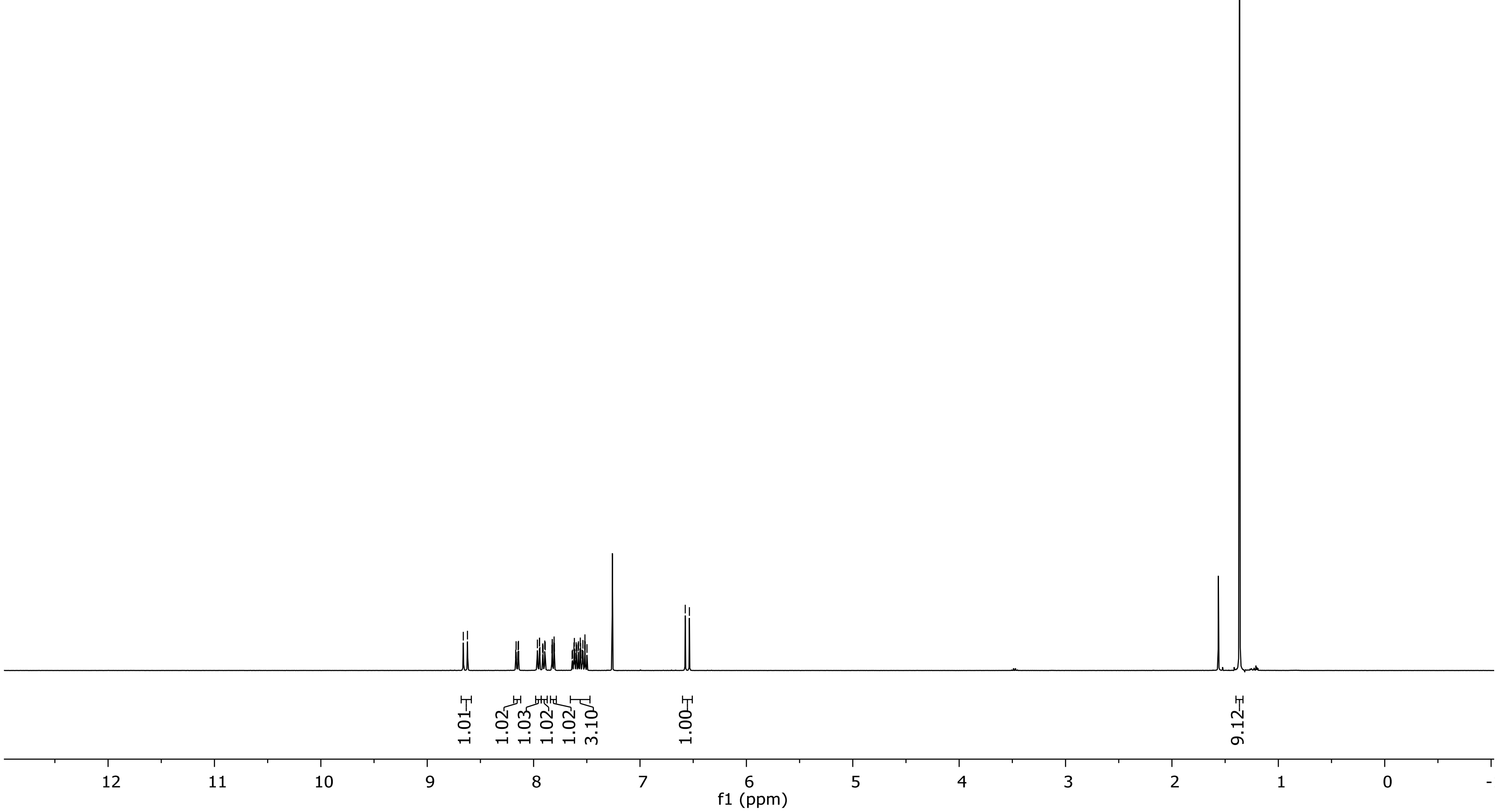

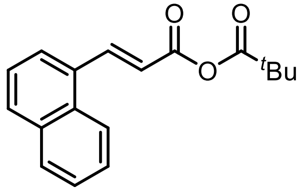

S31, $^{13}C\{^{1}H\}$, $CDCl_3$, 101 MHz

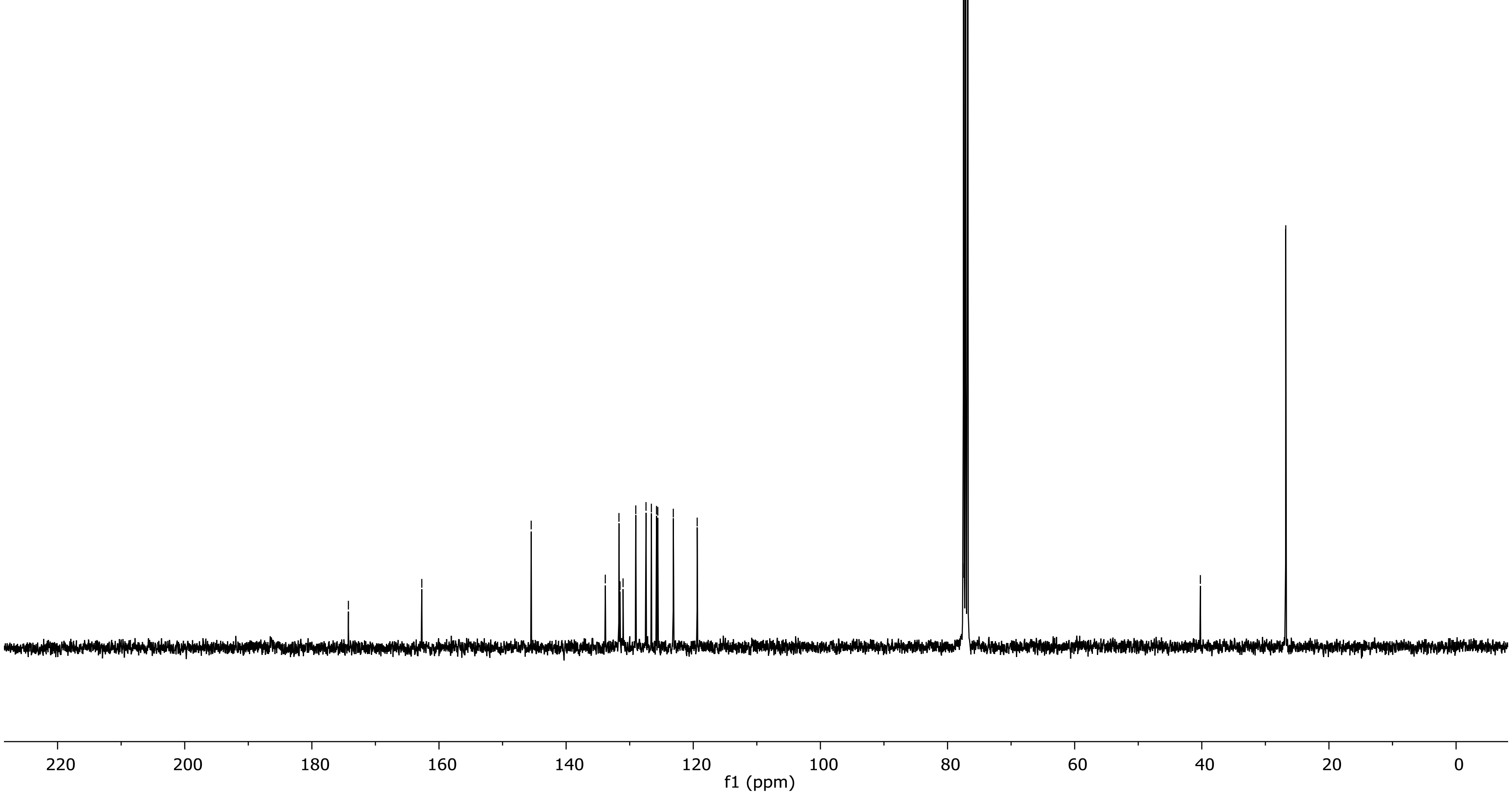

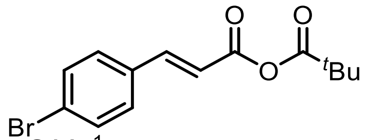

S14, ^{1}H, $CDCl_3$, 500 MHz

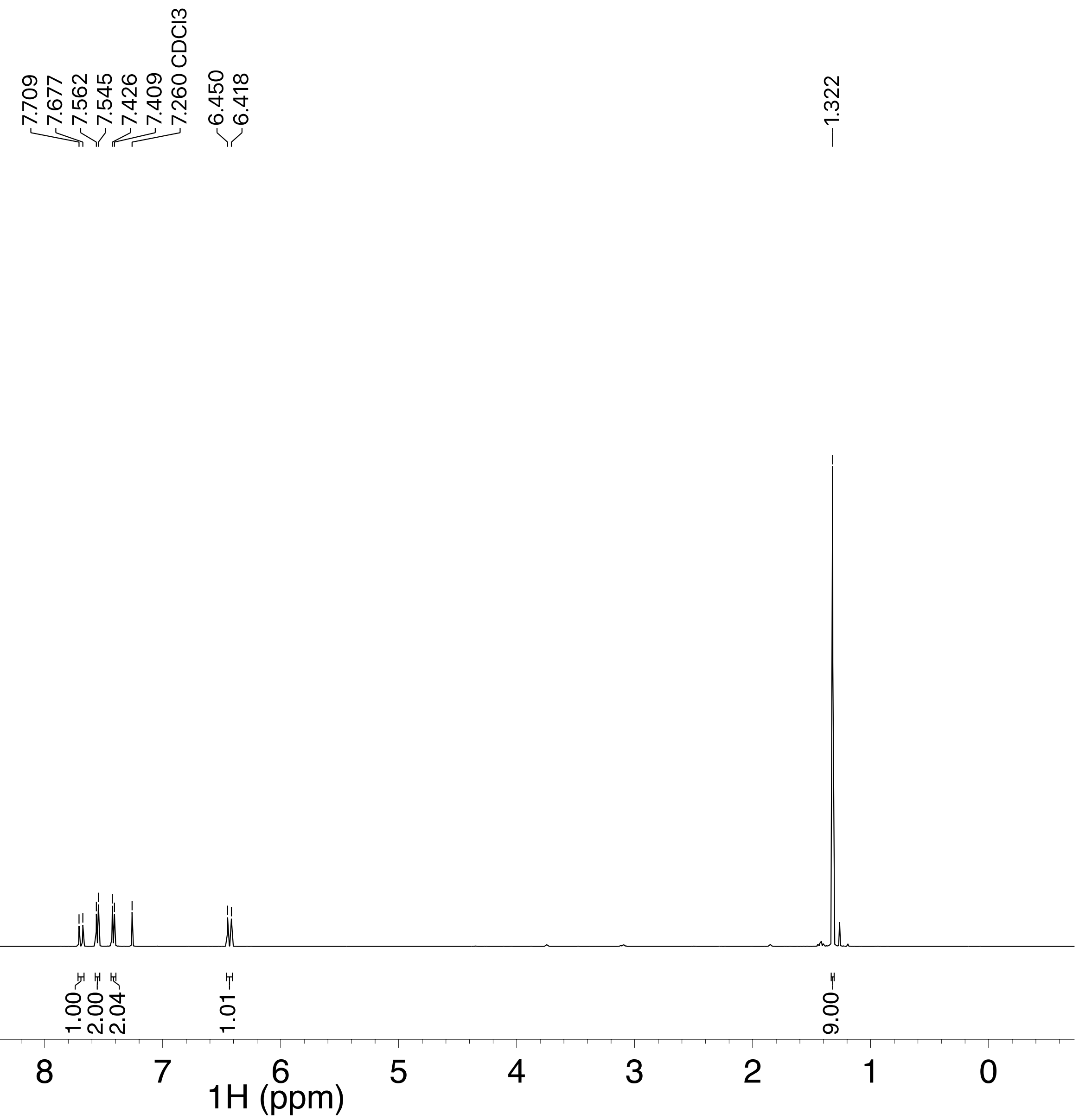

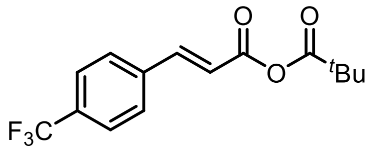

S32, ^{1}H, $CDCl_3$, 400 MHz

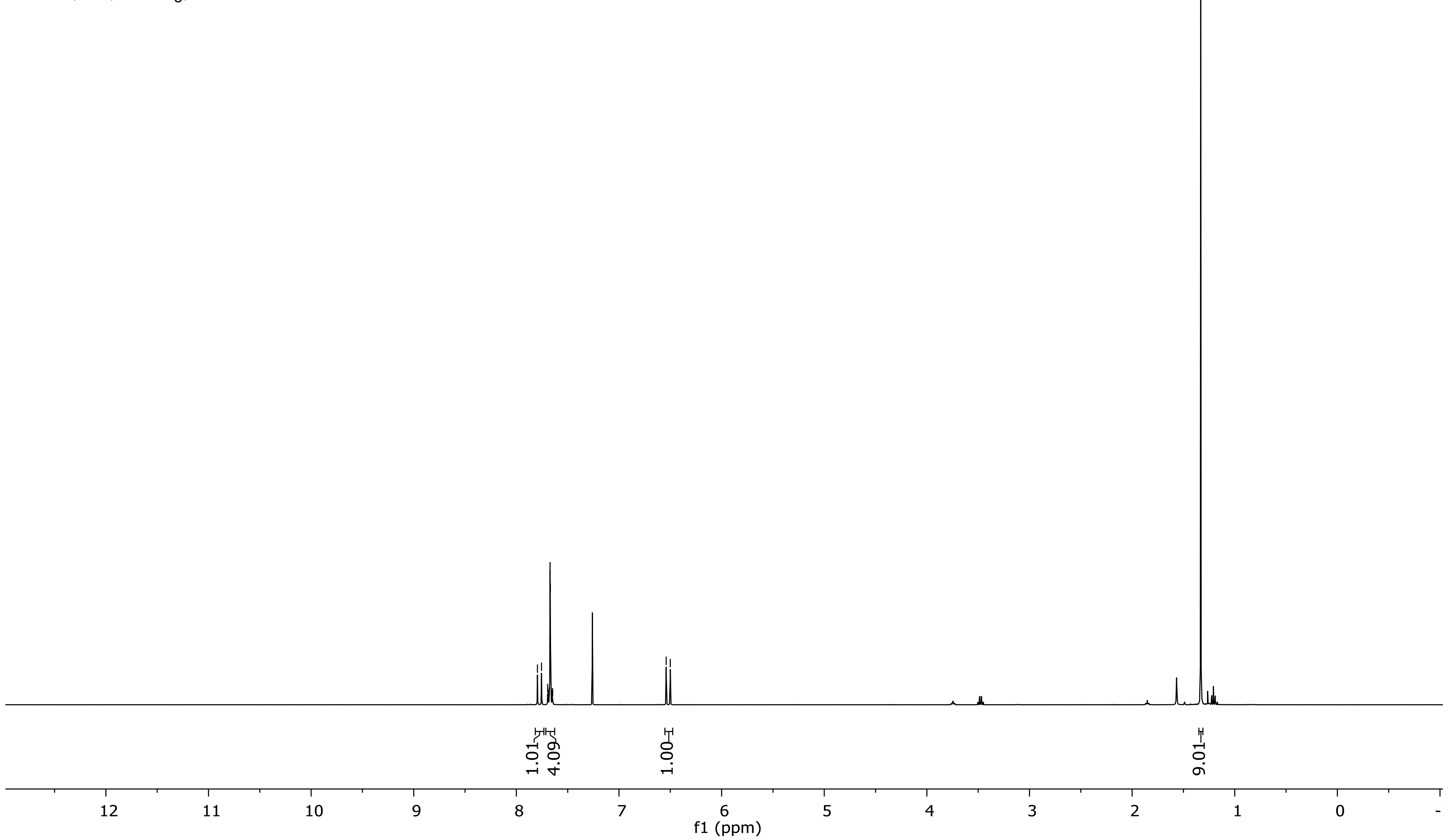

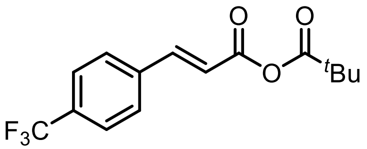

S32, $^{19}F\{^{1}H\}$, $CDCl_3$, 376 MHz

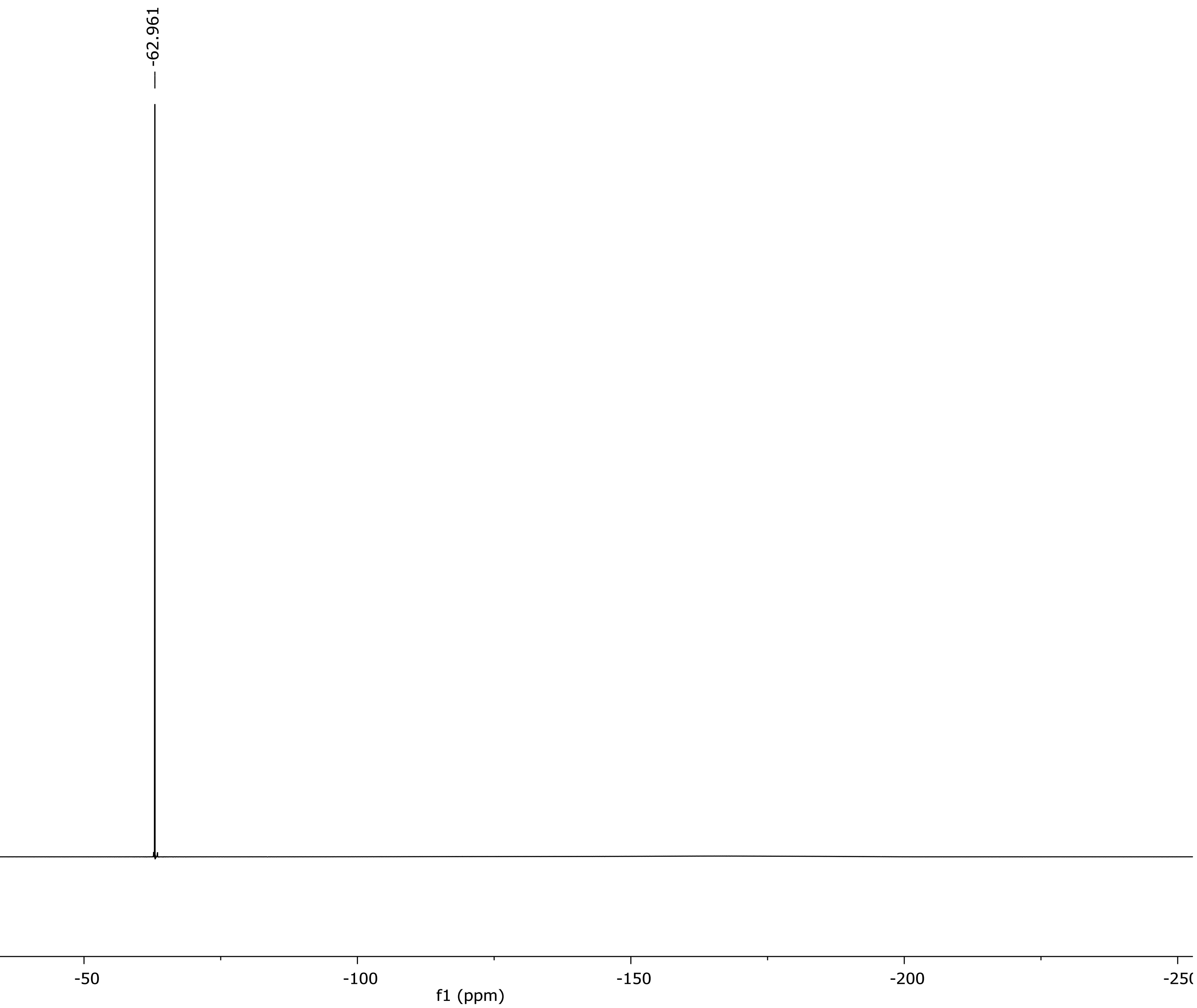

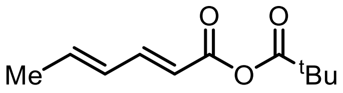

S10, ^{1}H, $CDCl_3$, 500 MHz

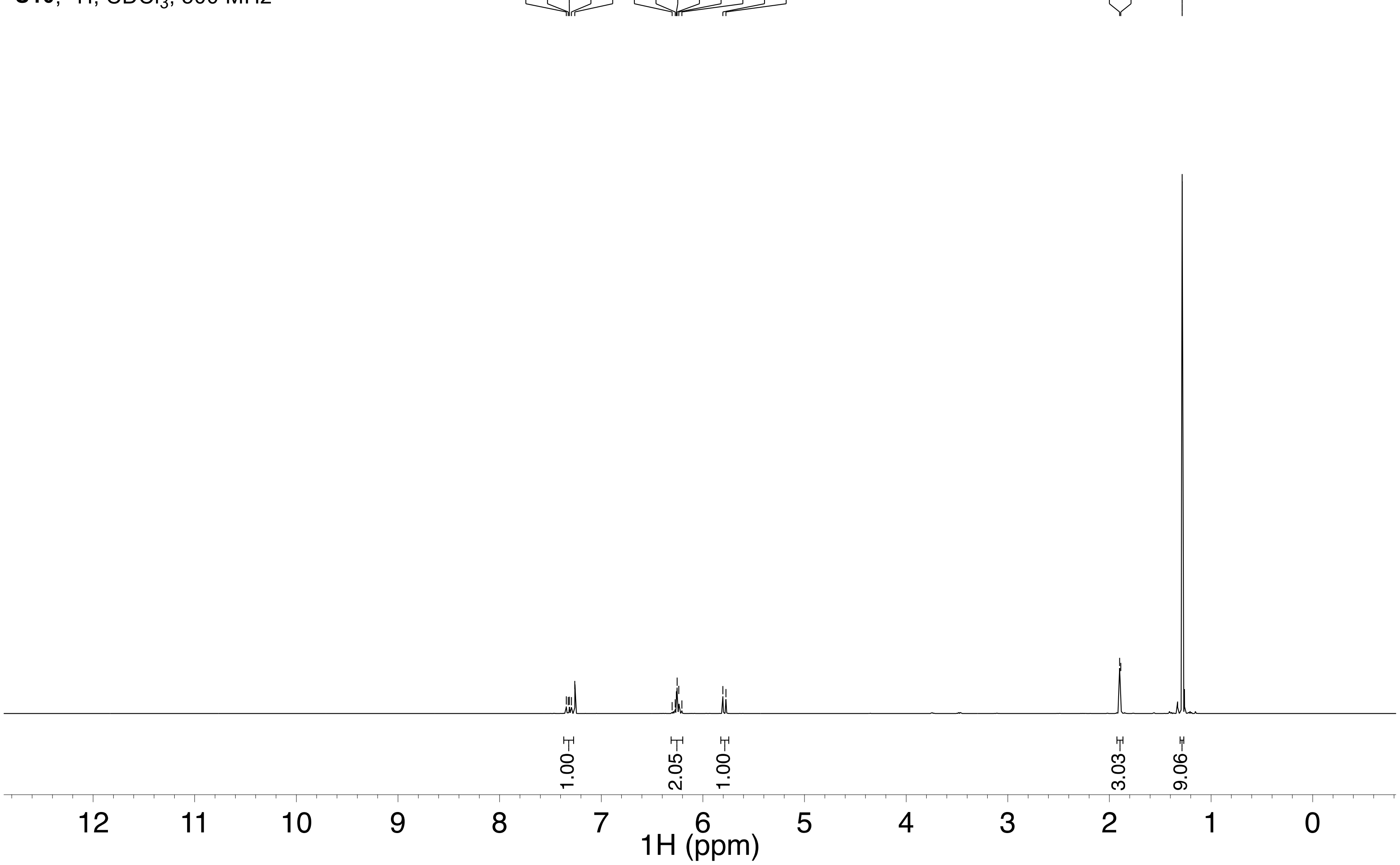

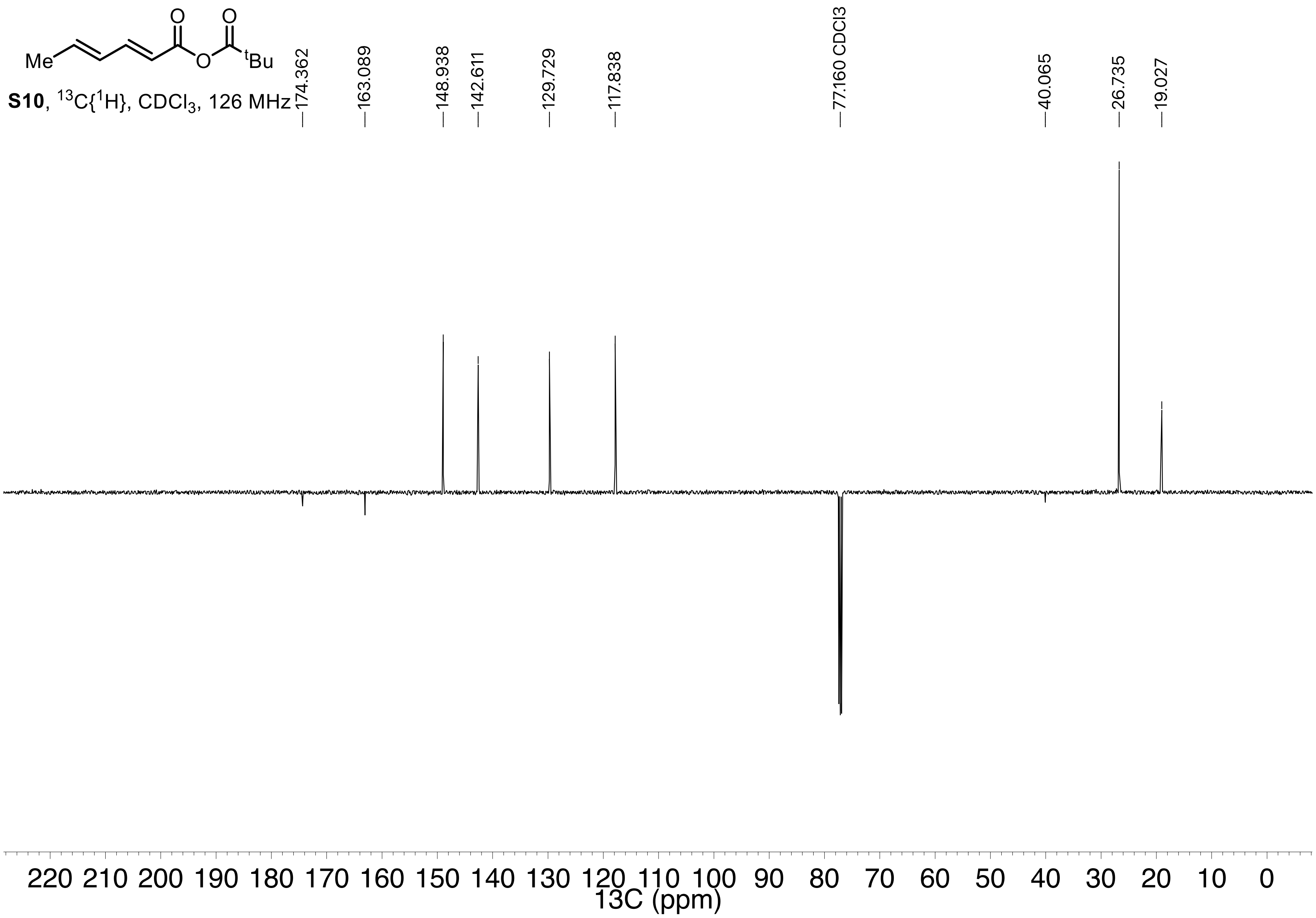
Me
O
O
O
tBu
S10, 13C{1H}, CDCl3, 126 MHz
174.362
163.089
148.938
142.611
129.729
117.838
77.160 CDCl3
40.065
26.735
19.027
220 210 200 190 180 170 160 150 140 130 120 110 100 90 80 70 60 50 40 30 20 10 0
13C (ppm)

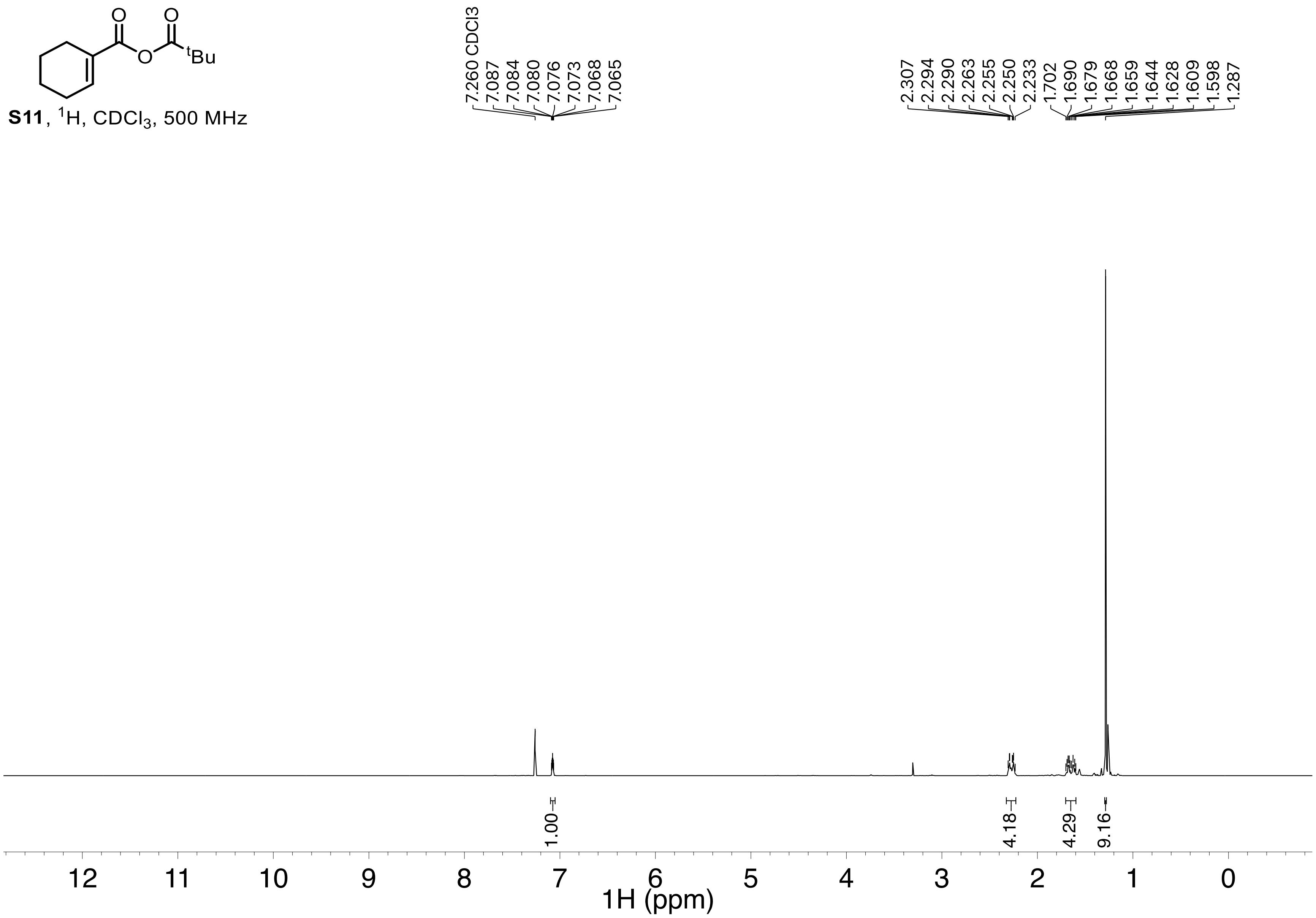
S11, 1H, CDCl3, 500 MHz
tBu
O
O
O
7.260 CDCl3
7.087
7.084
7.080
7.076
7.073
7.068
7.065
2.307
2.294
2.290
2.263
2.255
2.250
2.233
1.702
1.690
1.679
1.668
1.659
1.644
1.628
1.609
1.598
1.287
1.00
4.18
4.29
9.16
12 11 10 9 8 7 6 5 4 3 2 1 0
1H (ppm)

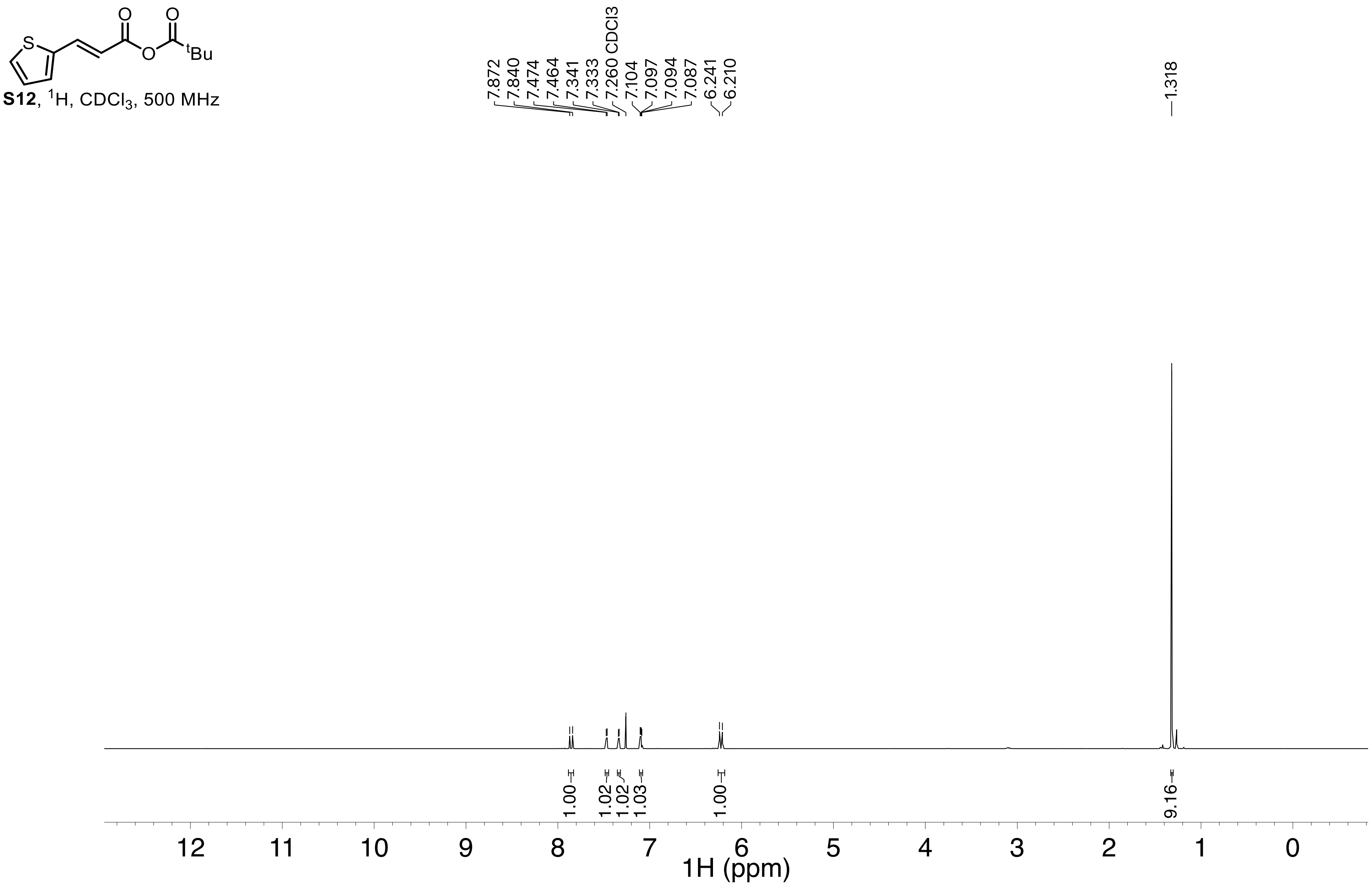

S12, ^{1}H, $CDCl_3$, 500 MHz

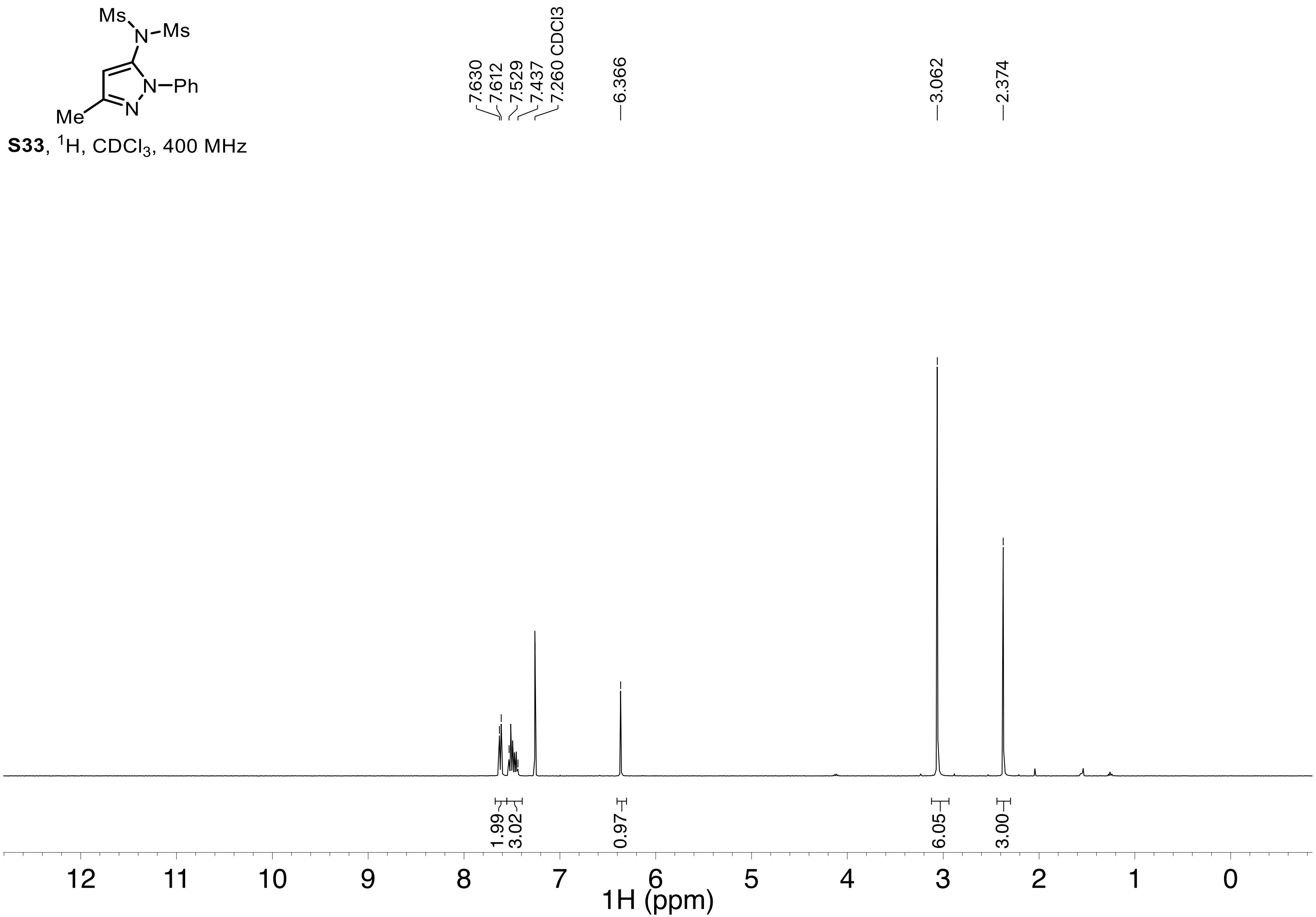

S33, ^{1}H, $CDCl_3$, 400 MHz

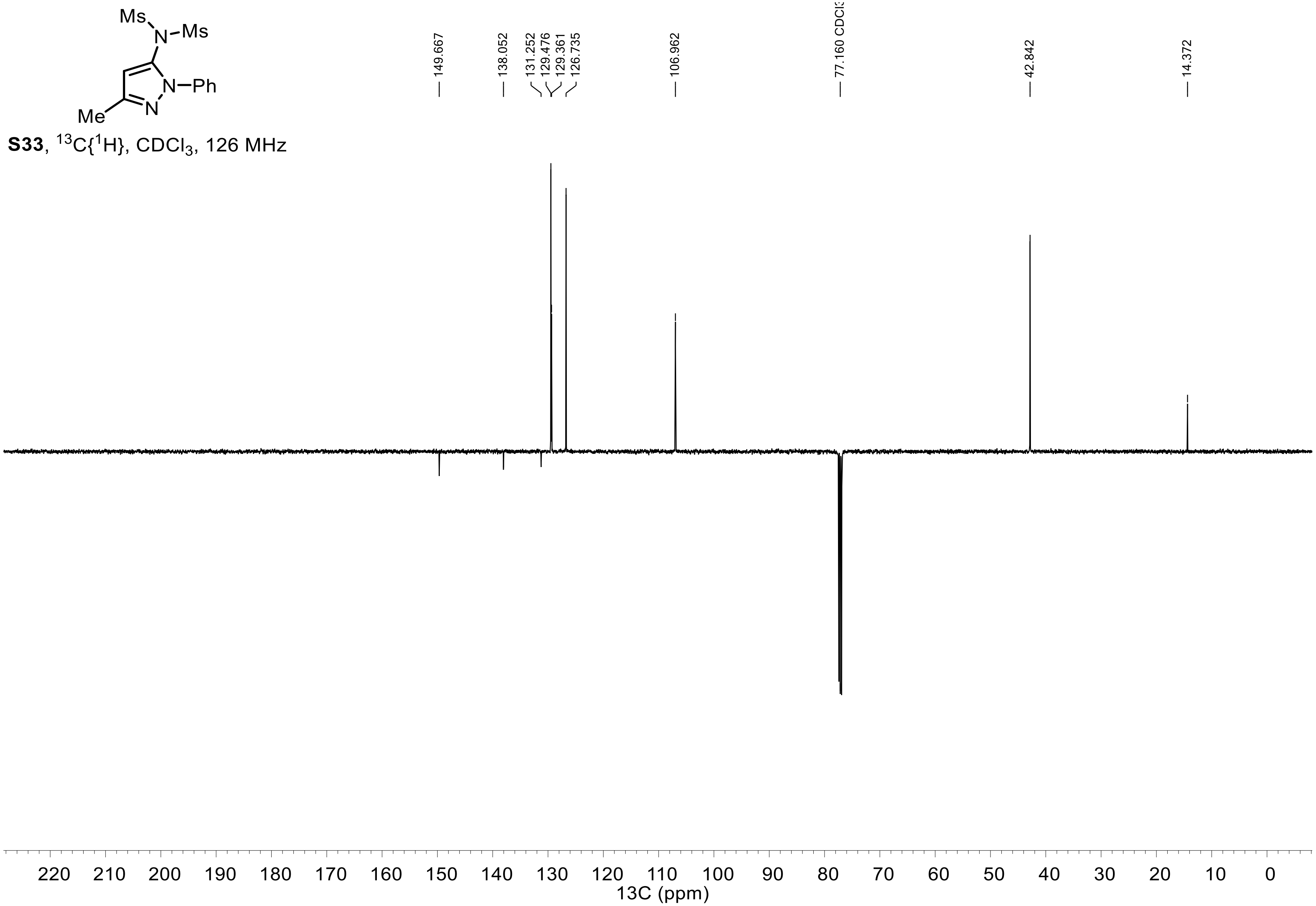

S33, $^{13}C\{^{1}H\}$, $CDCl_3$, 126 MHz

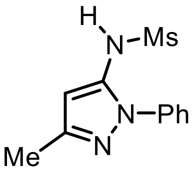

15, ^{1}H, $CDCl_3$, 400 MHz

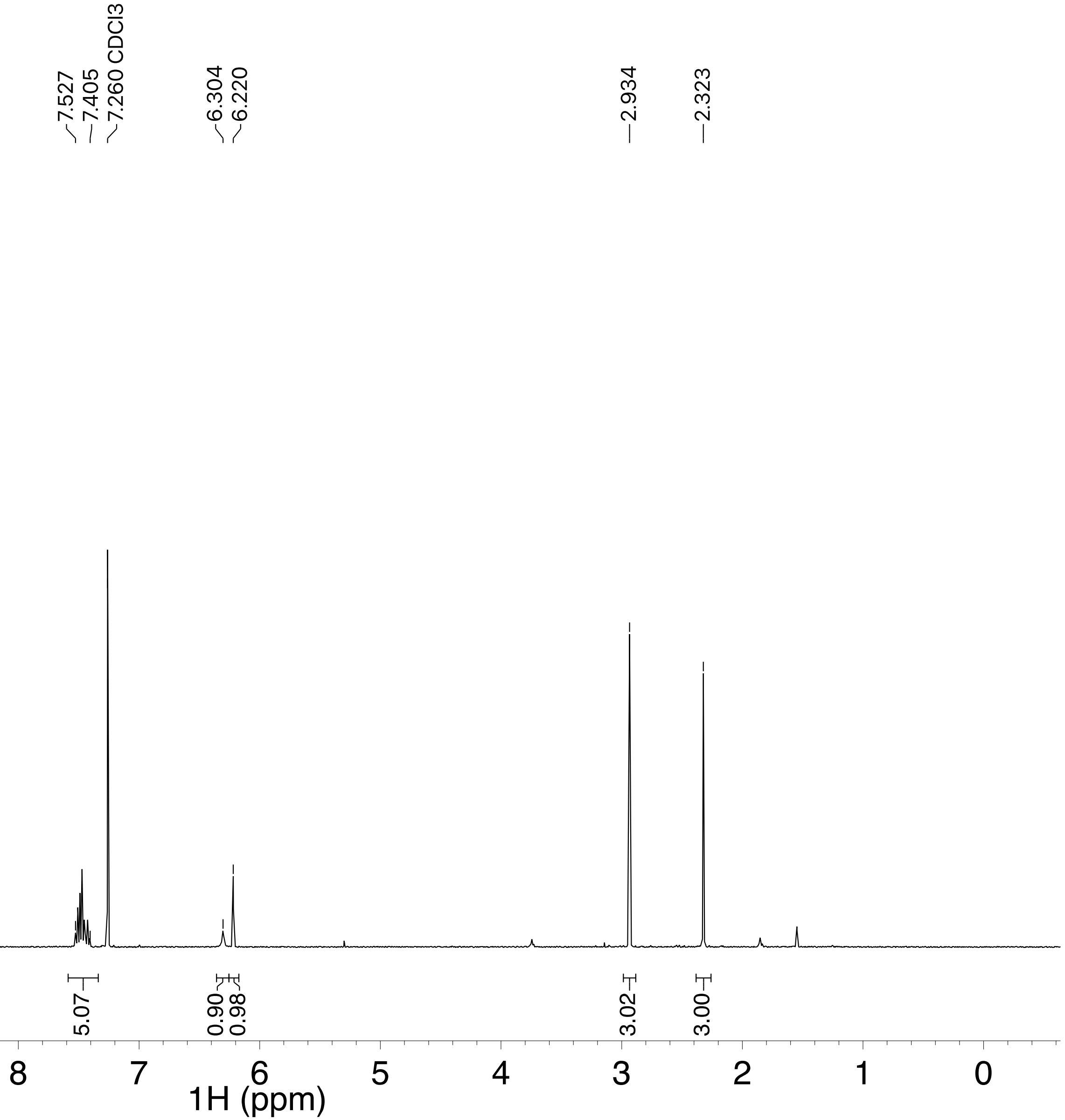

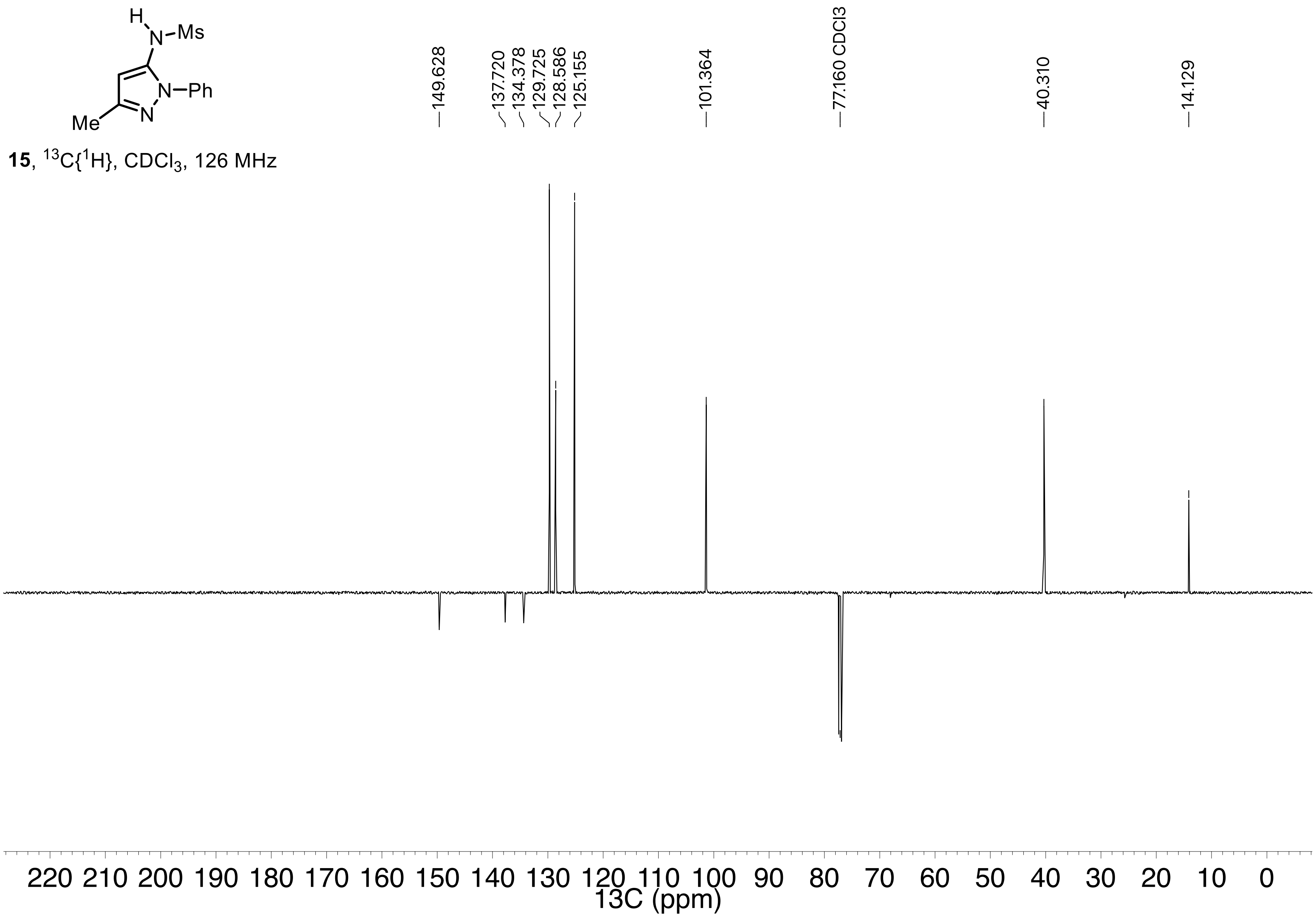

15, $^{13}C\{^{1}H\}$, $CDCl_3$, 126 MHz

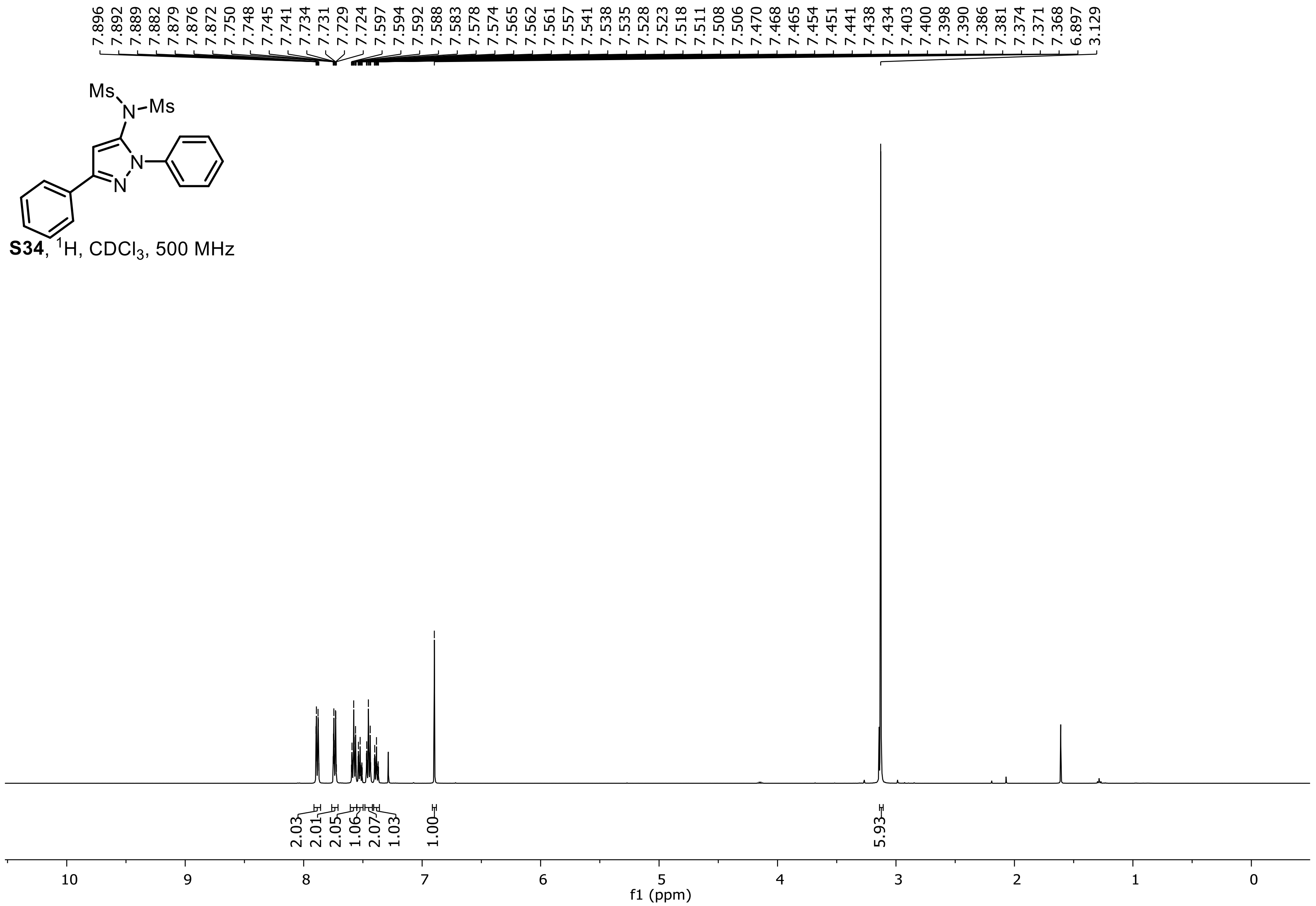

S34, ^{1}H, $CDCl_3$, 500 MHz

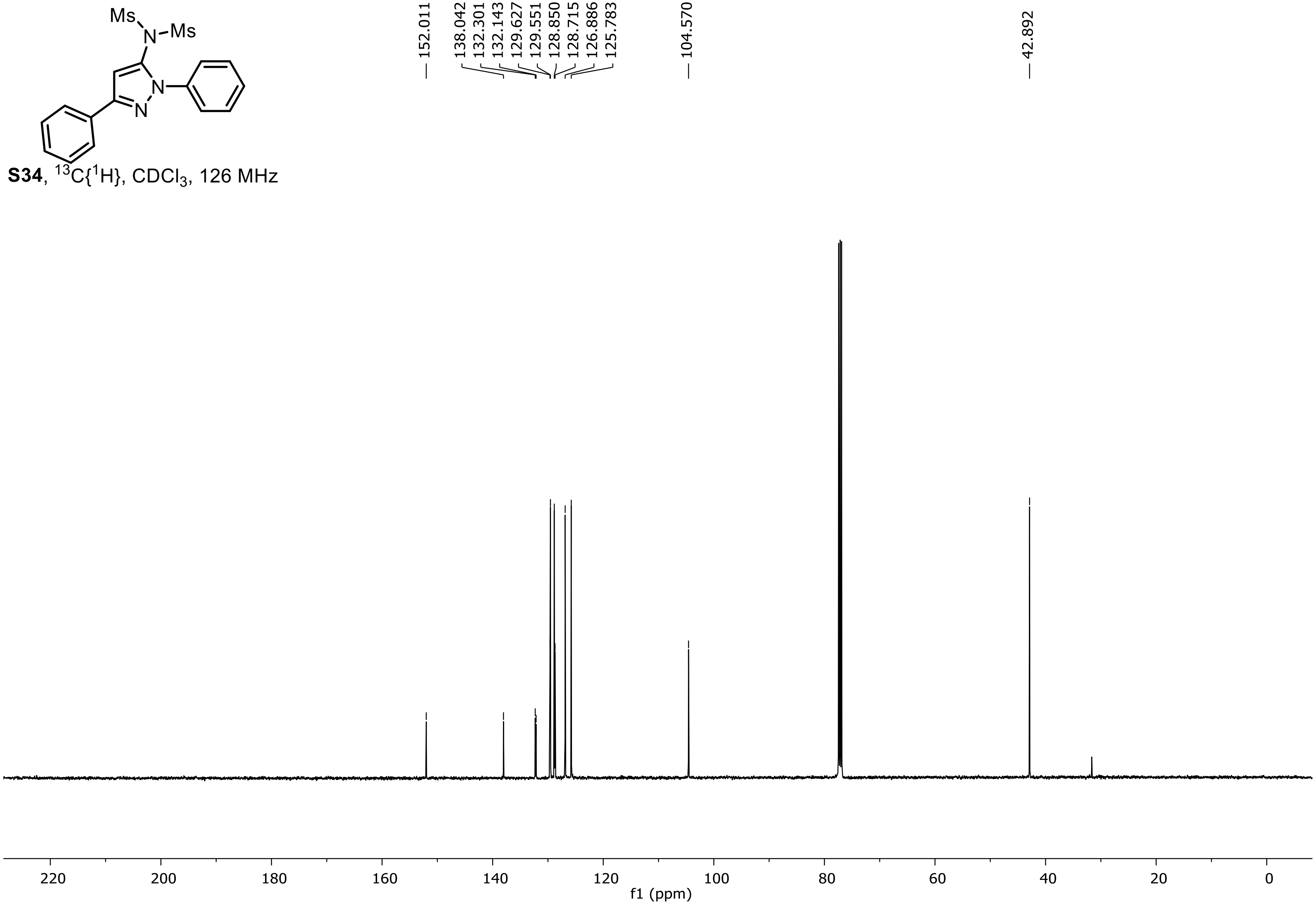
Ms
N
Ms
N
N
S34, $^{13}C\{^{1}H\}$, $CDCl_3$, 126 MHz
152.011
138.042
132.301
132.143
129.627
129.551
128.850
128.715
126.886
125.783
104.570
42.892
220
200
180
160
140
120
100
80
60
40
20
0
f1 (ppm)

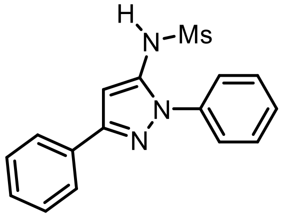

S35, ^{1}H, $CDCl_3$, 400 MHz

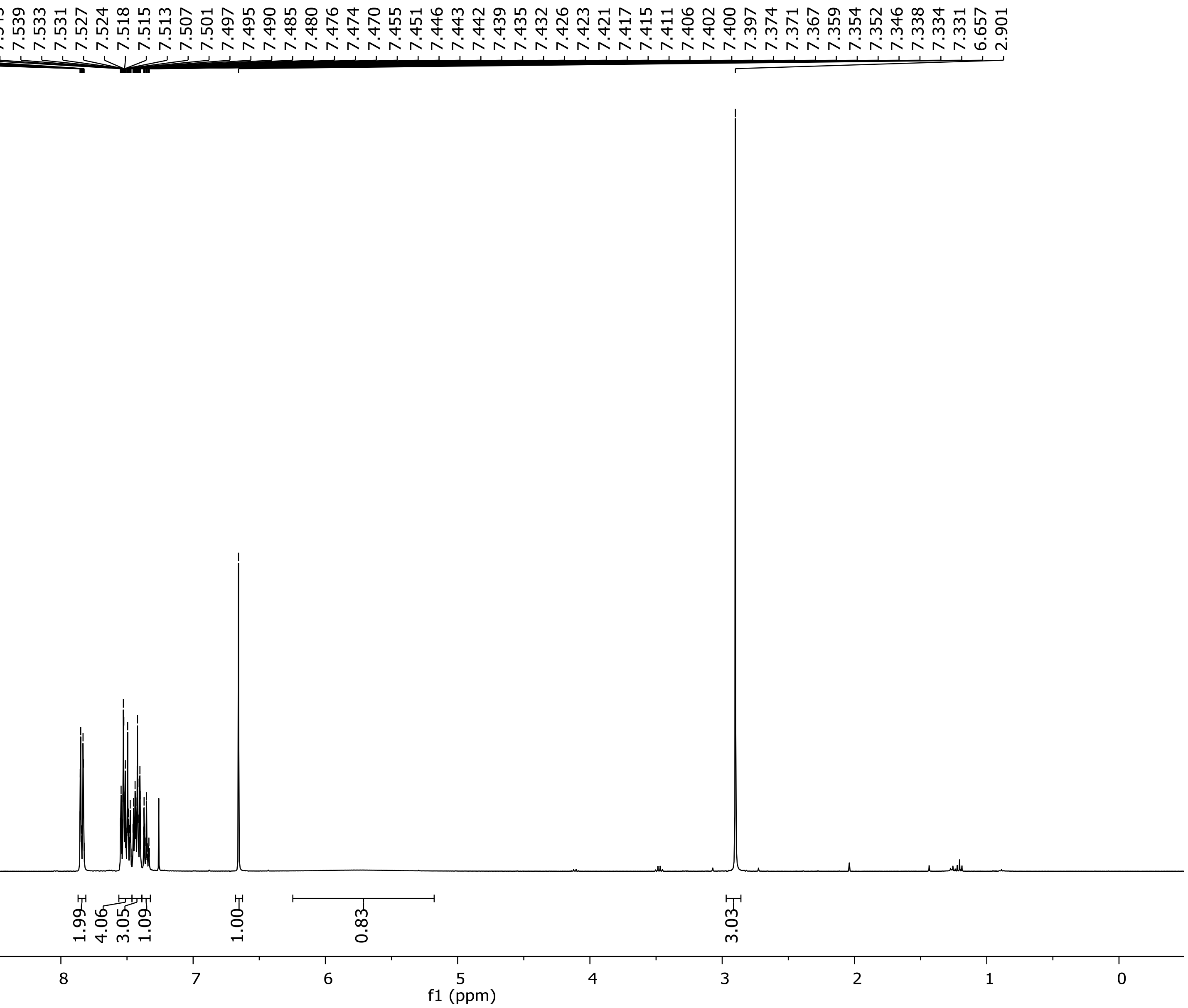

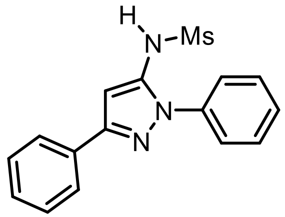

S35, $^{13}C\{^{1}H\}$, $CDCl_3$, 101 MHz

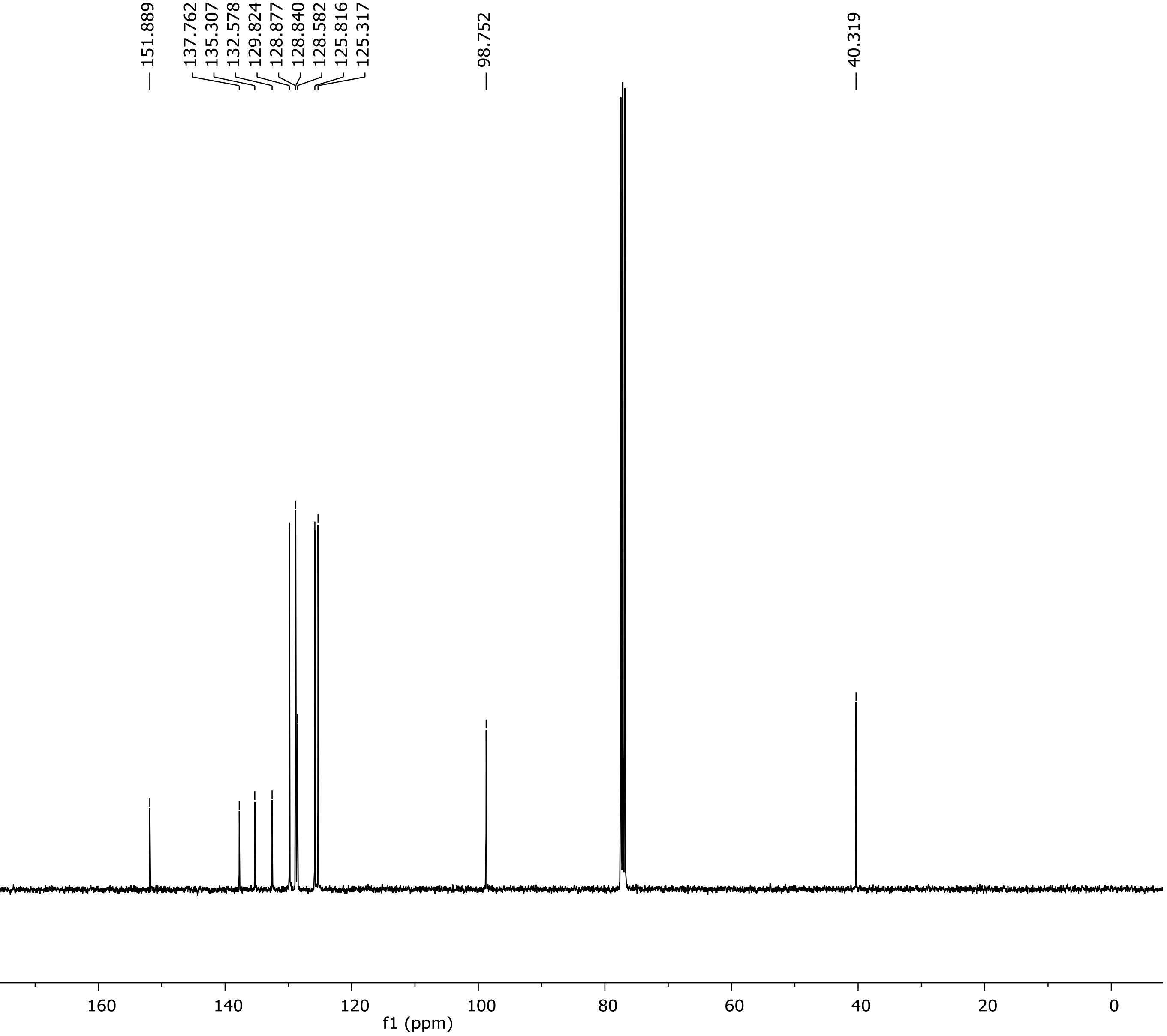

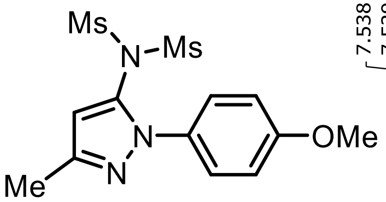

S36, ^1H, $CDCl_3$, 400 MHz

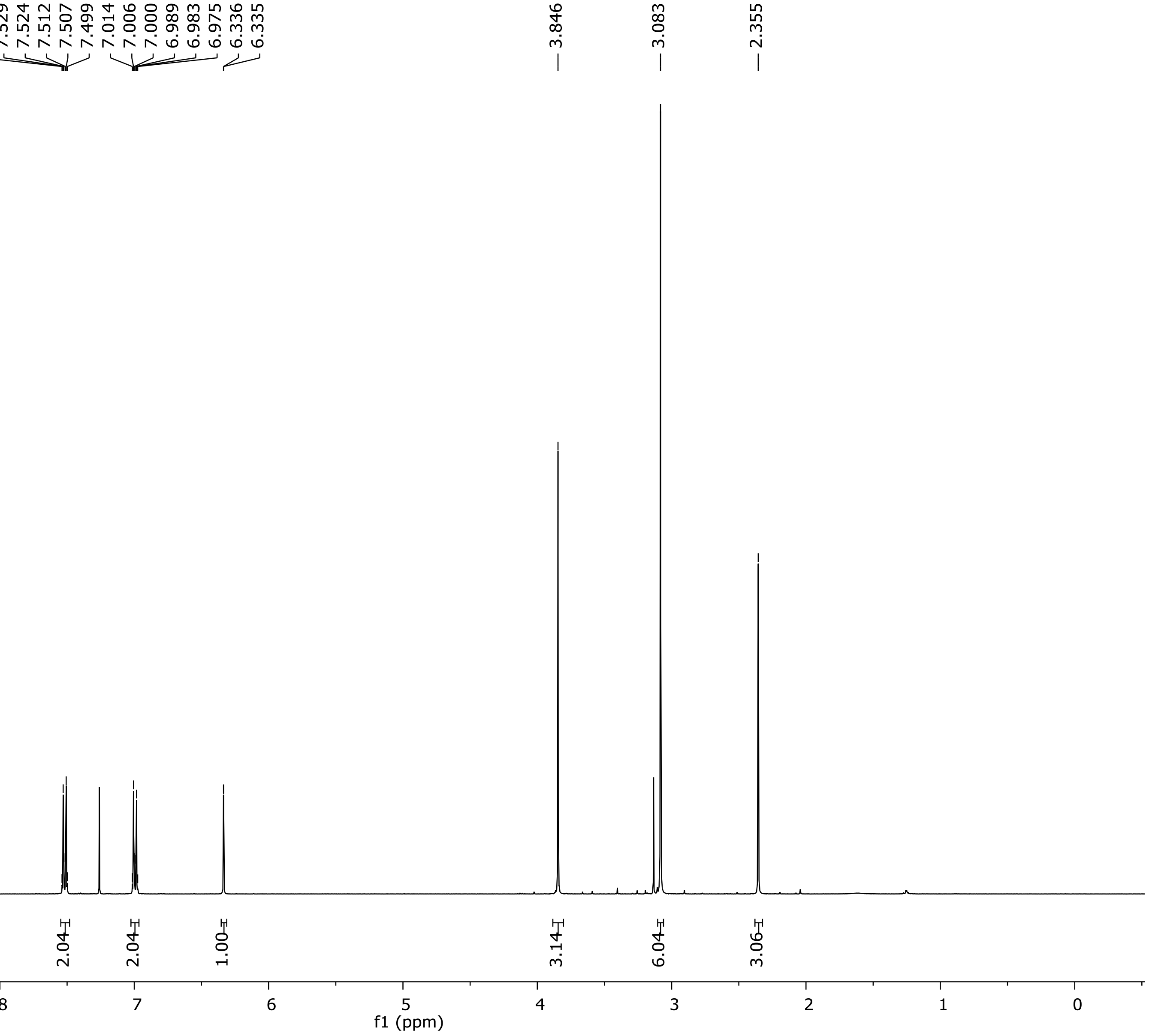

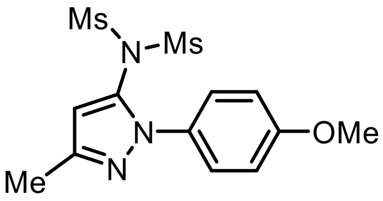

S36, $^{13}C\{^{1}H\}$, $CDCl_3$, 101 MHz

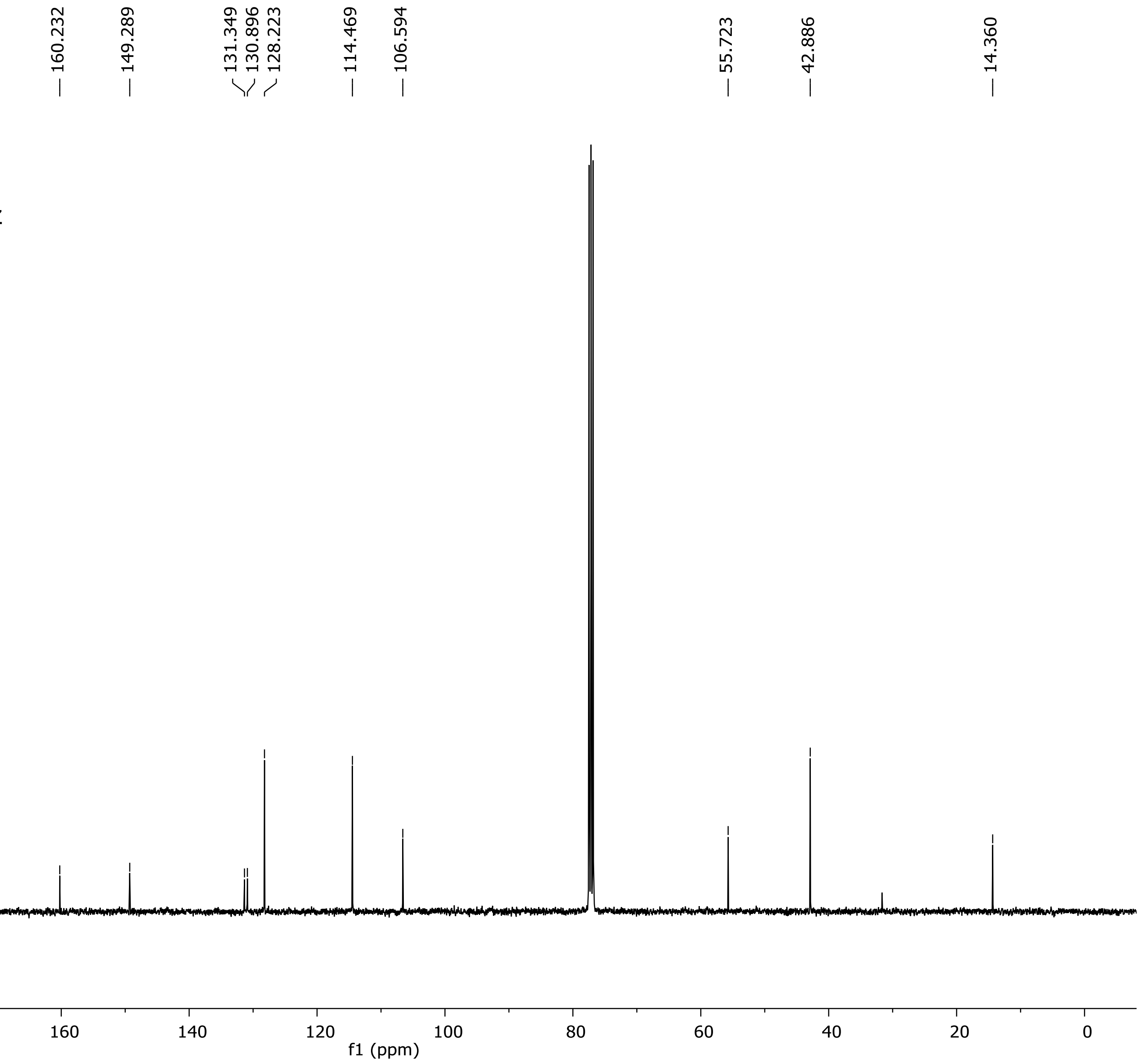

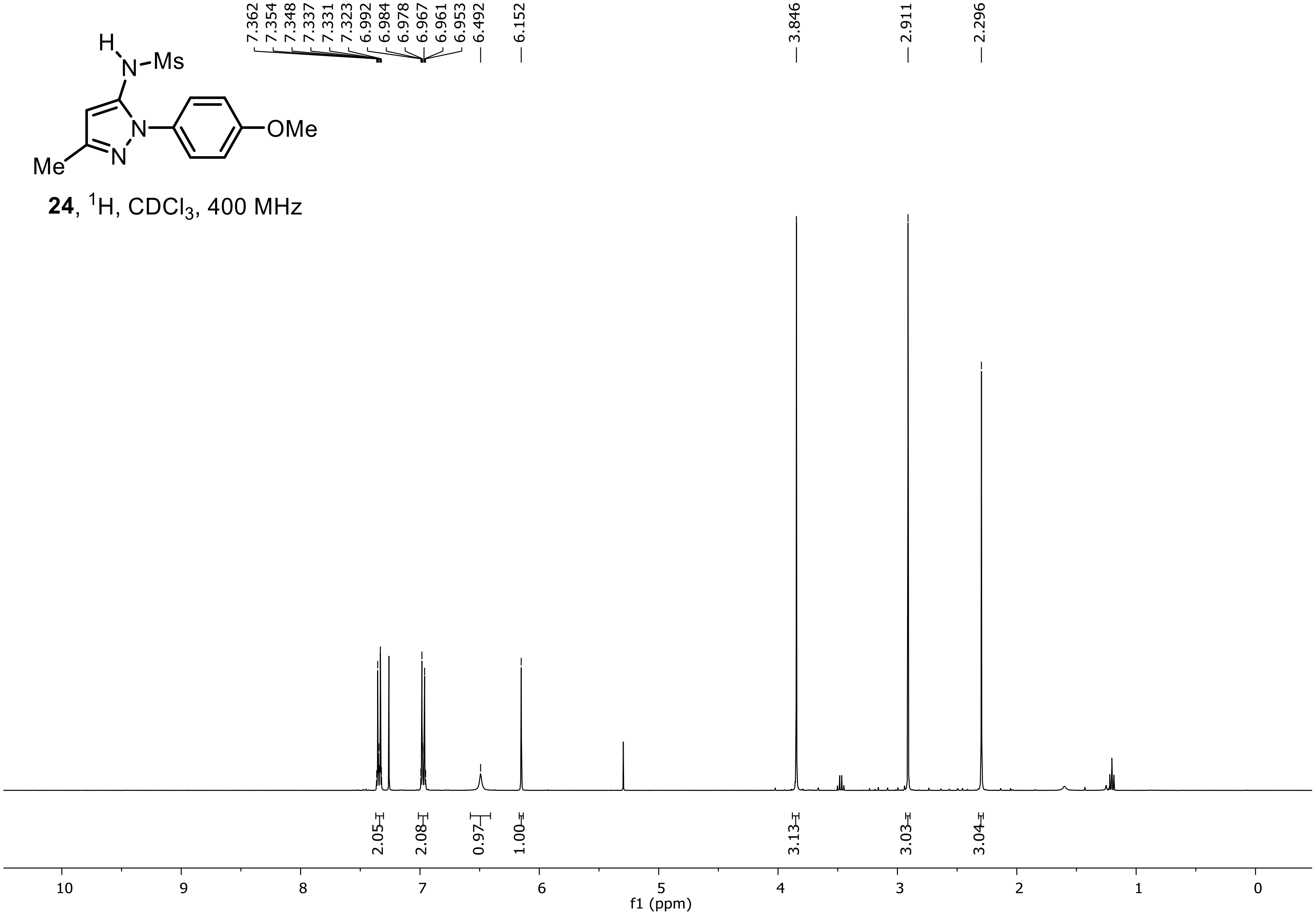

24, ^{1}H, $CDCl_3$, 400 MHz

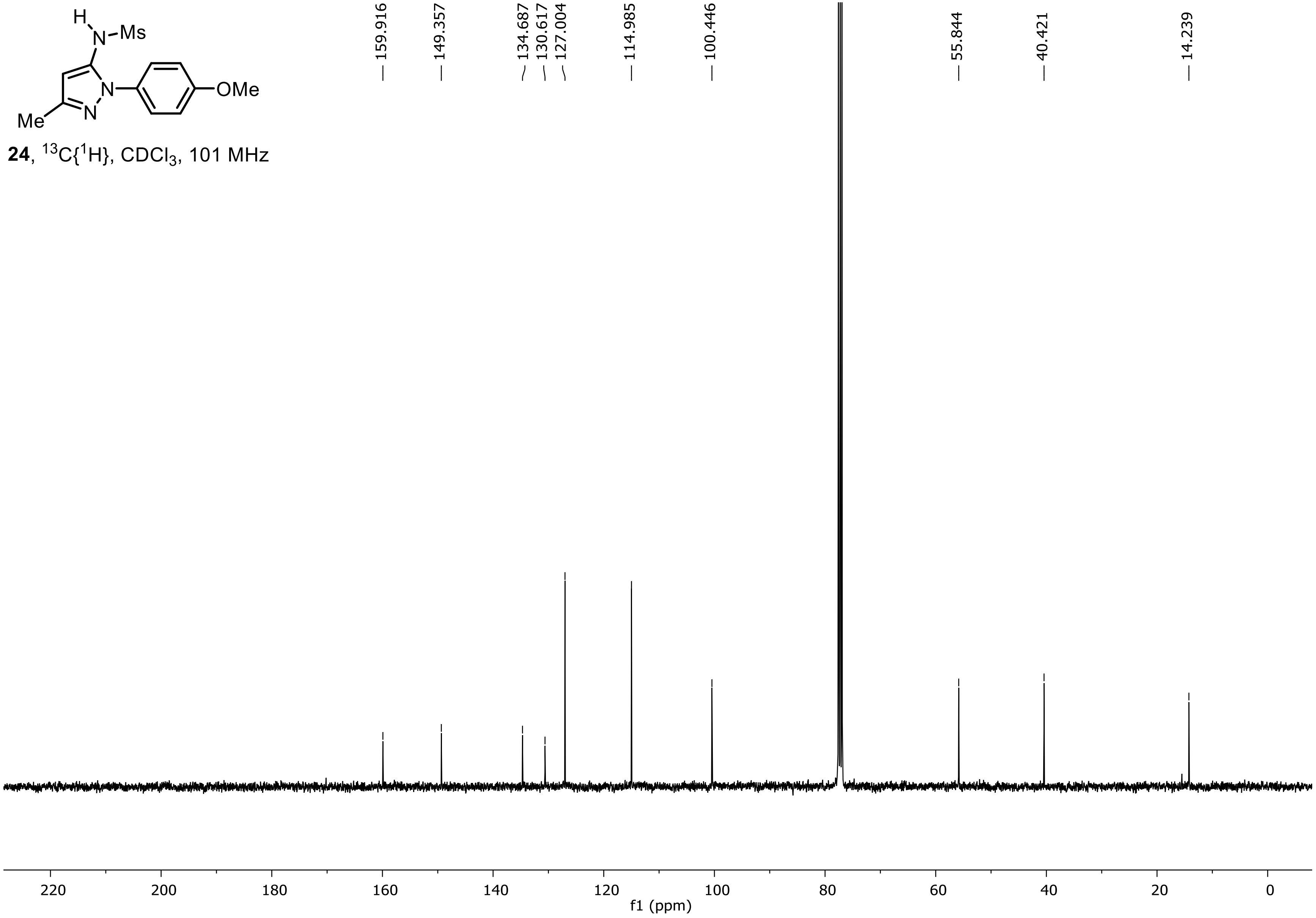

24, $^{13}C\{^{1}H\}$, $CDCl_3$, 101 MHz

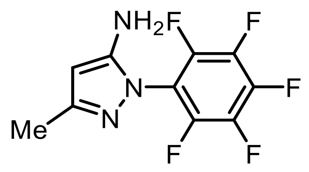

S37, ^{1}H, $CDCl_3$, 500 MHz

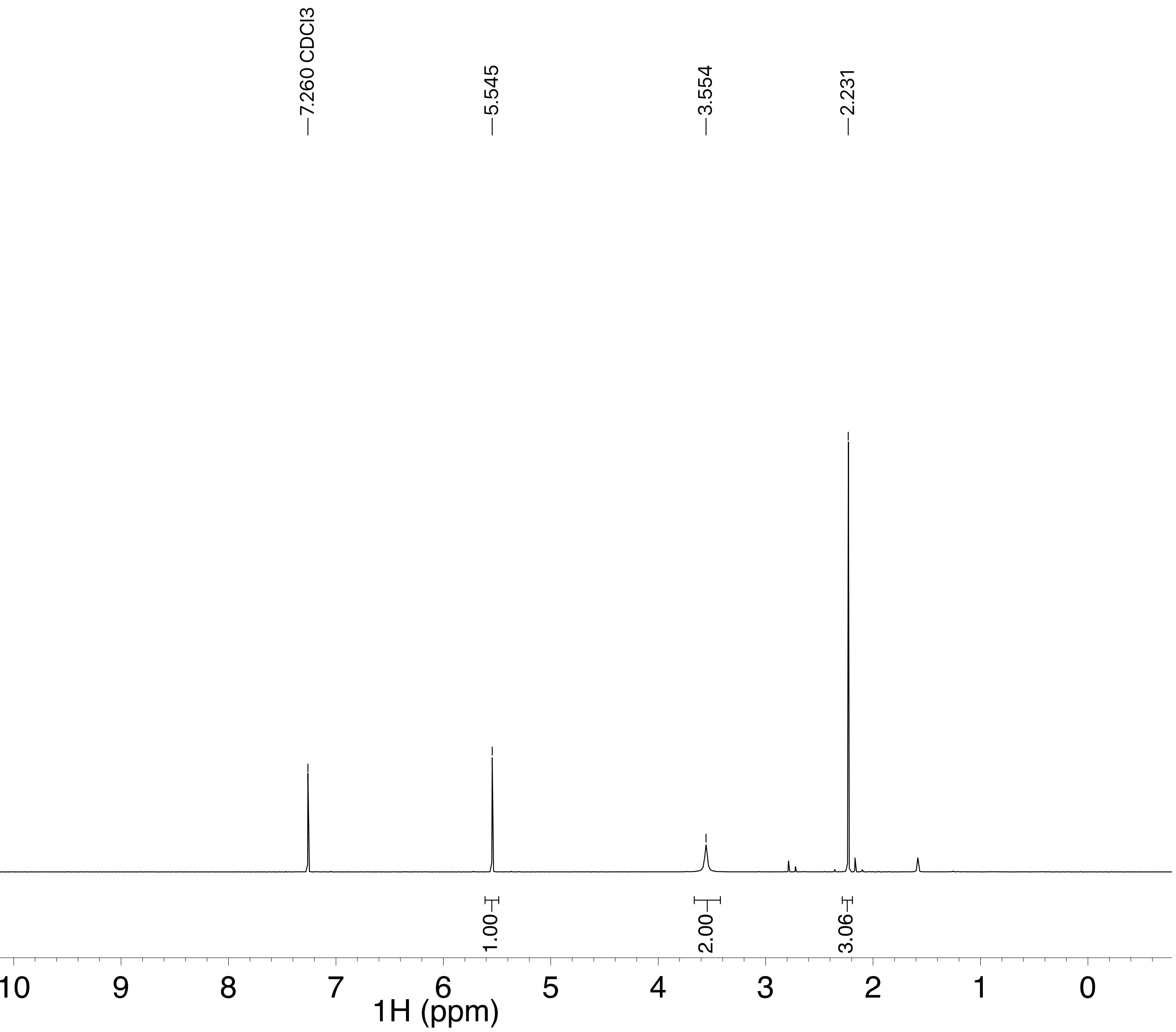

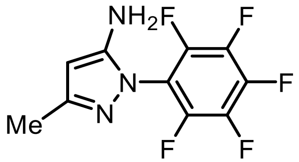

S37, $^{13}C\{^1H\}$, $CDCl_3$, 126 MHz

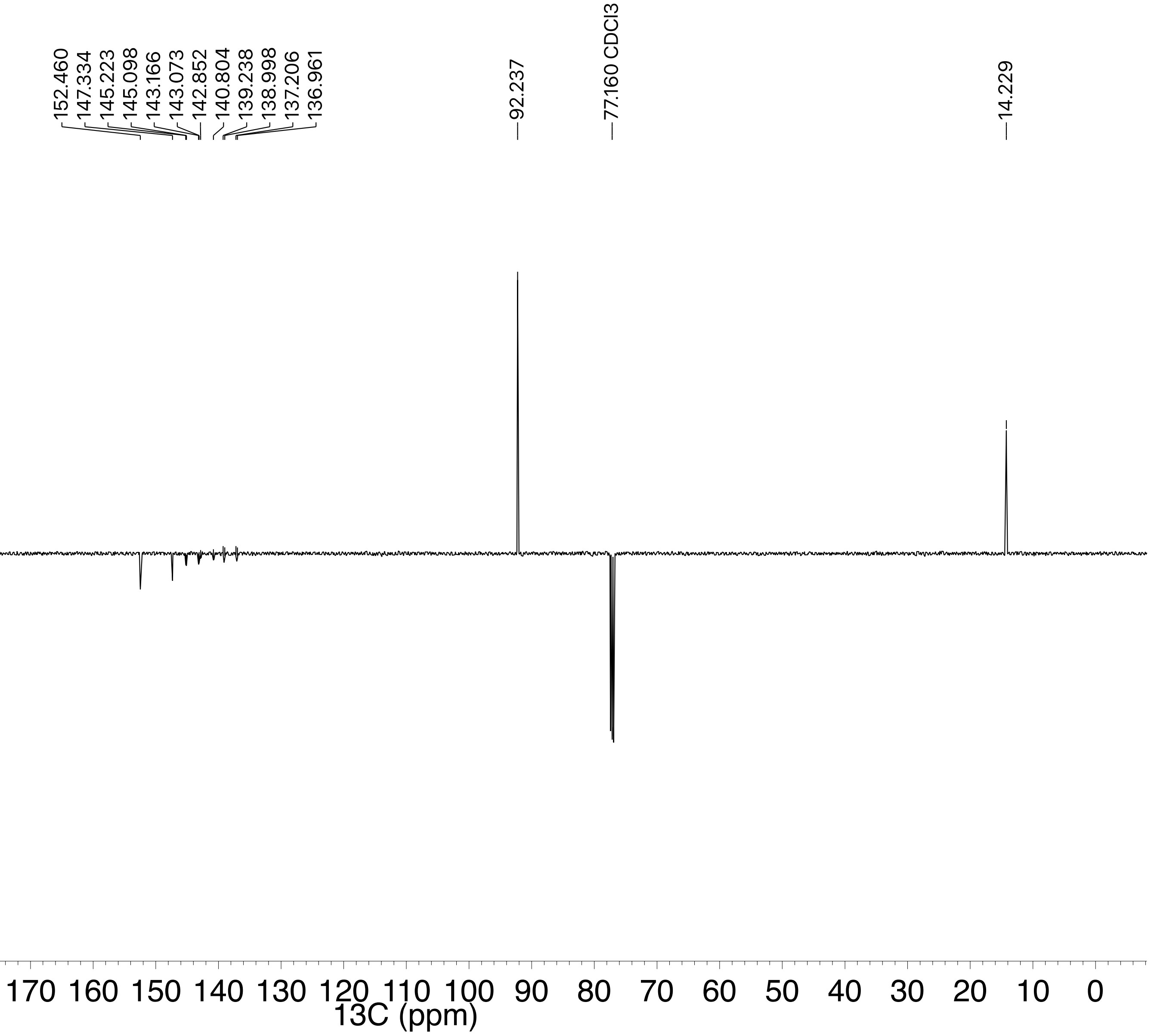

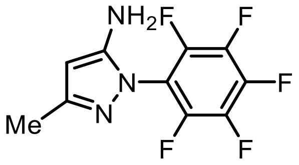

S37, $^{13}C\{^{1}H\}$, $CDCl_3$, 376 MHz

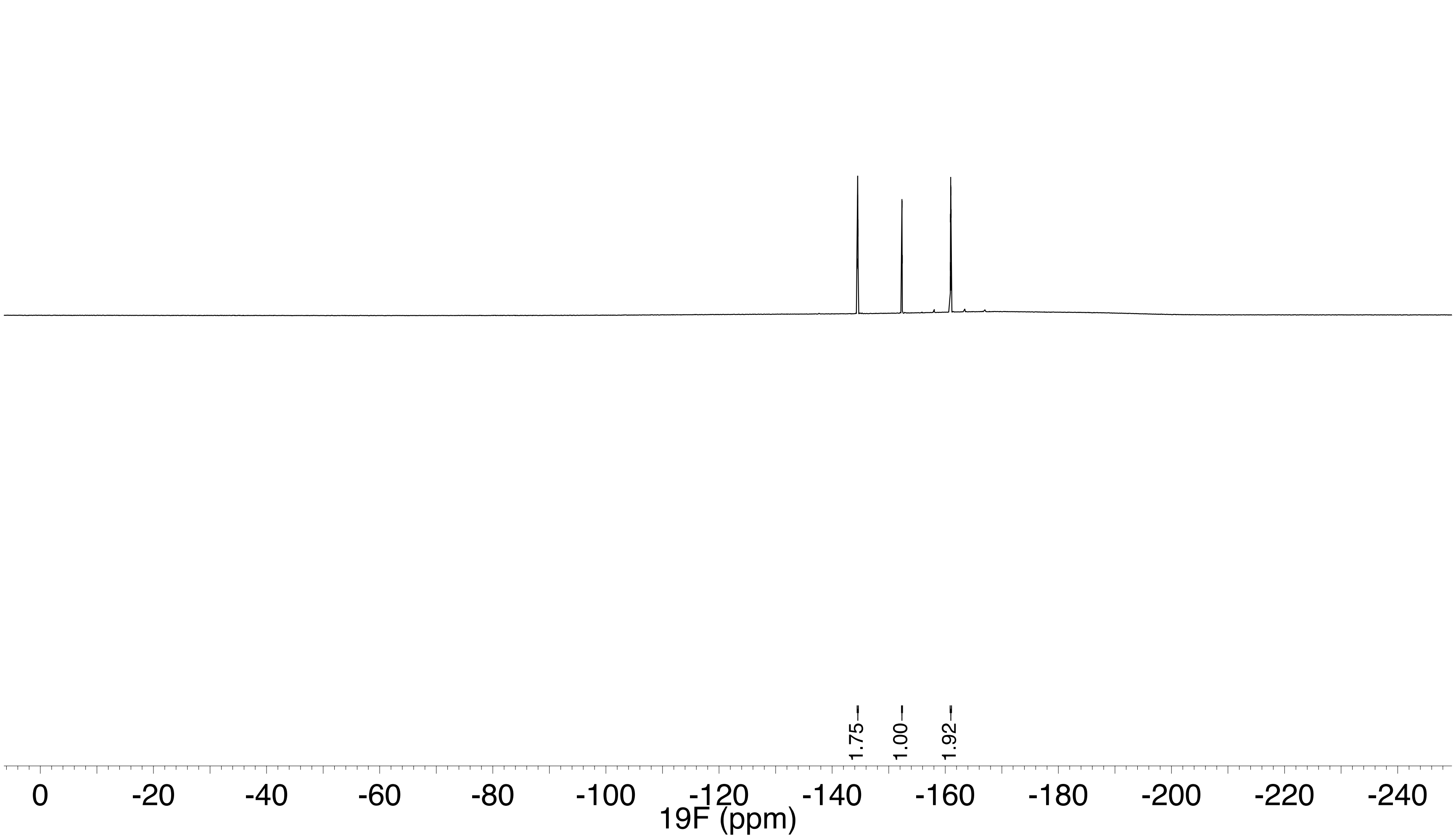

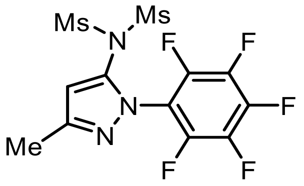

S38, ^{1}H, $CDCl_3$, 400 MHz

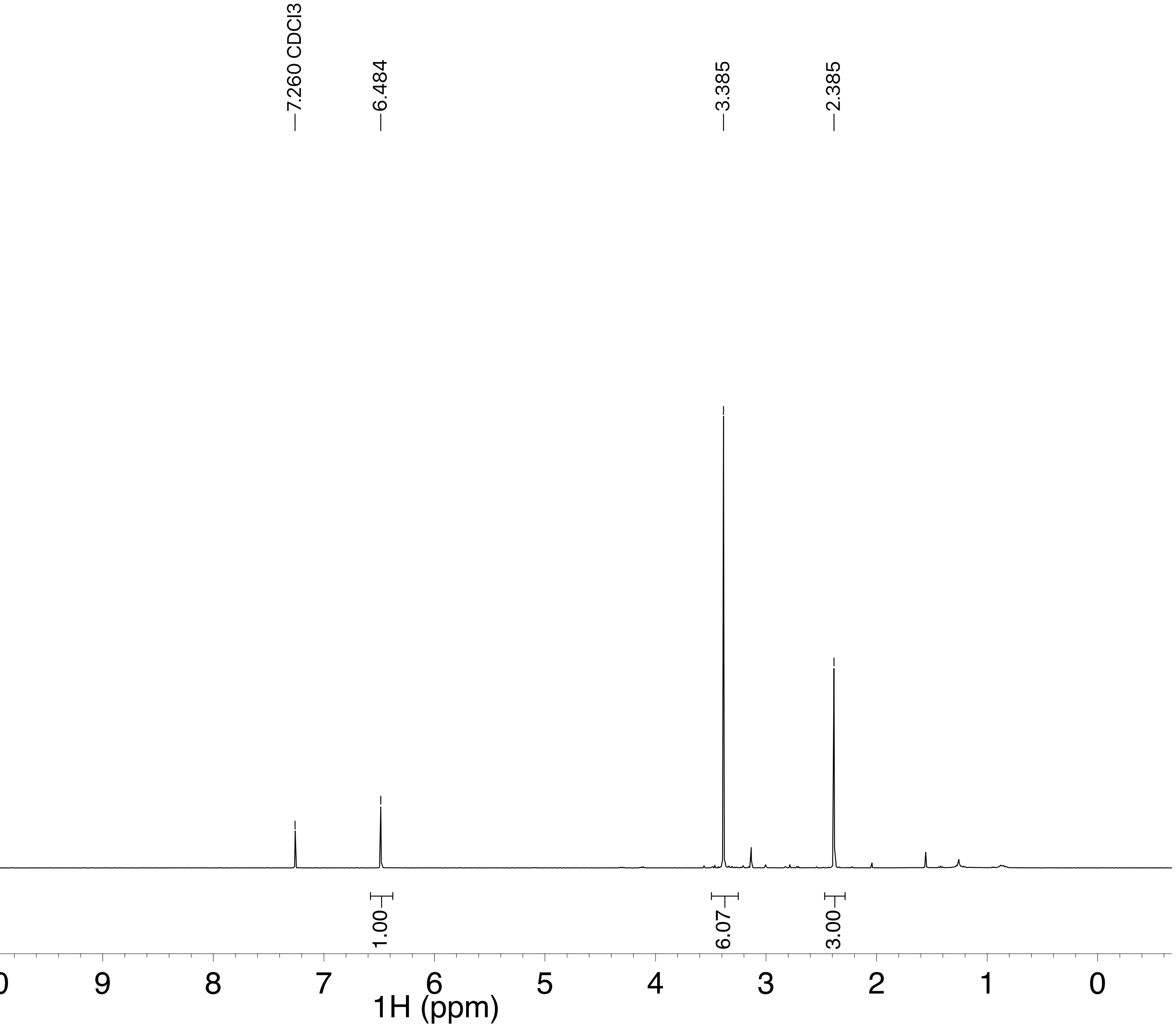

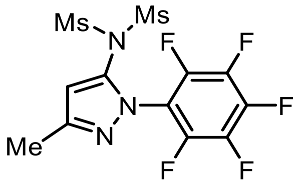

S38, $^{13}C\{^{1}H\}$, $CDCl_3$, 126 MHz

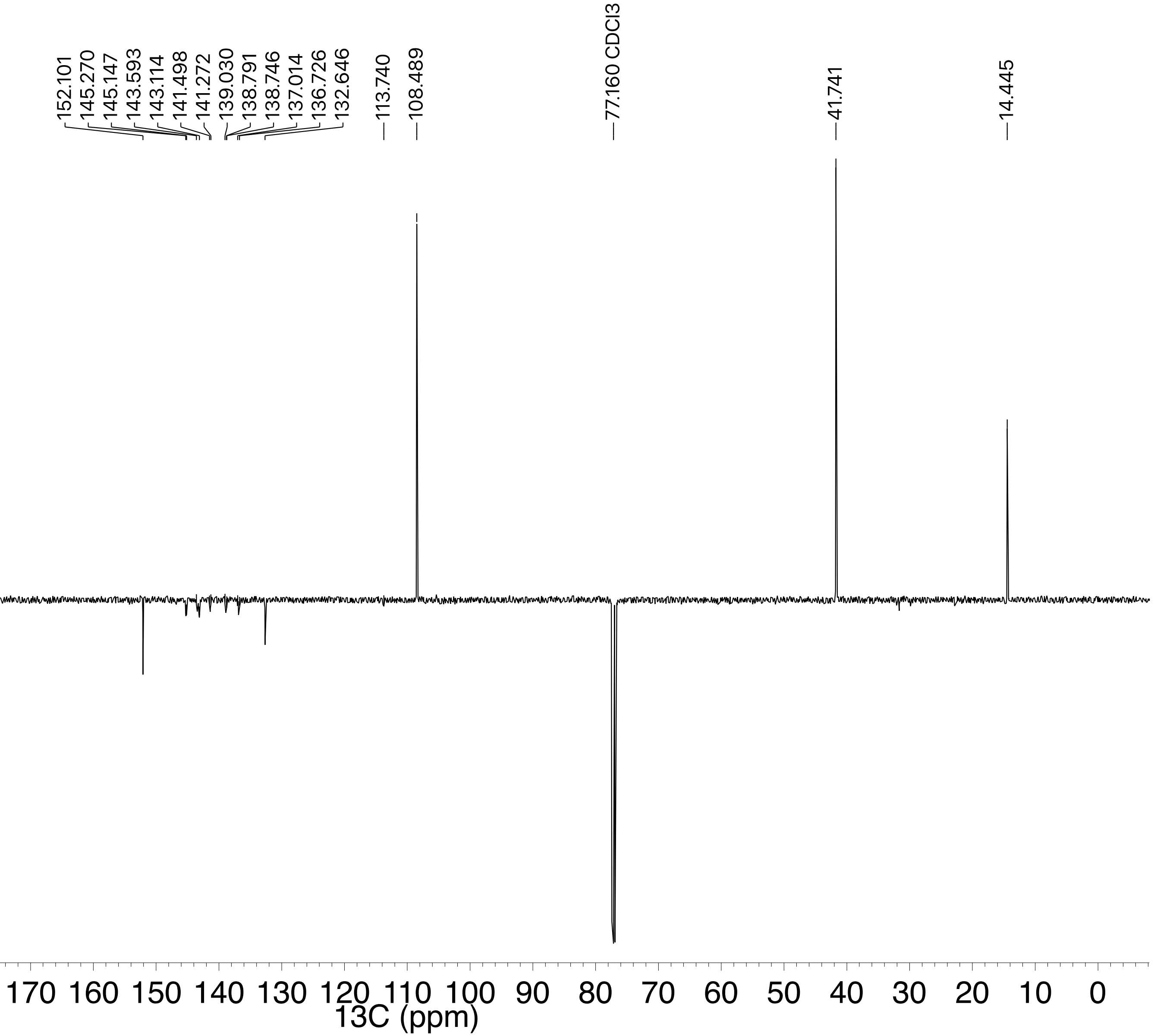

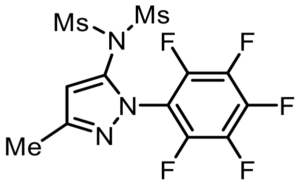

S38, $^{19}F\{^{1}H\}$, $CDCl_3$, 376 MHz

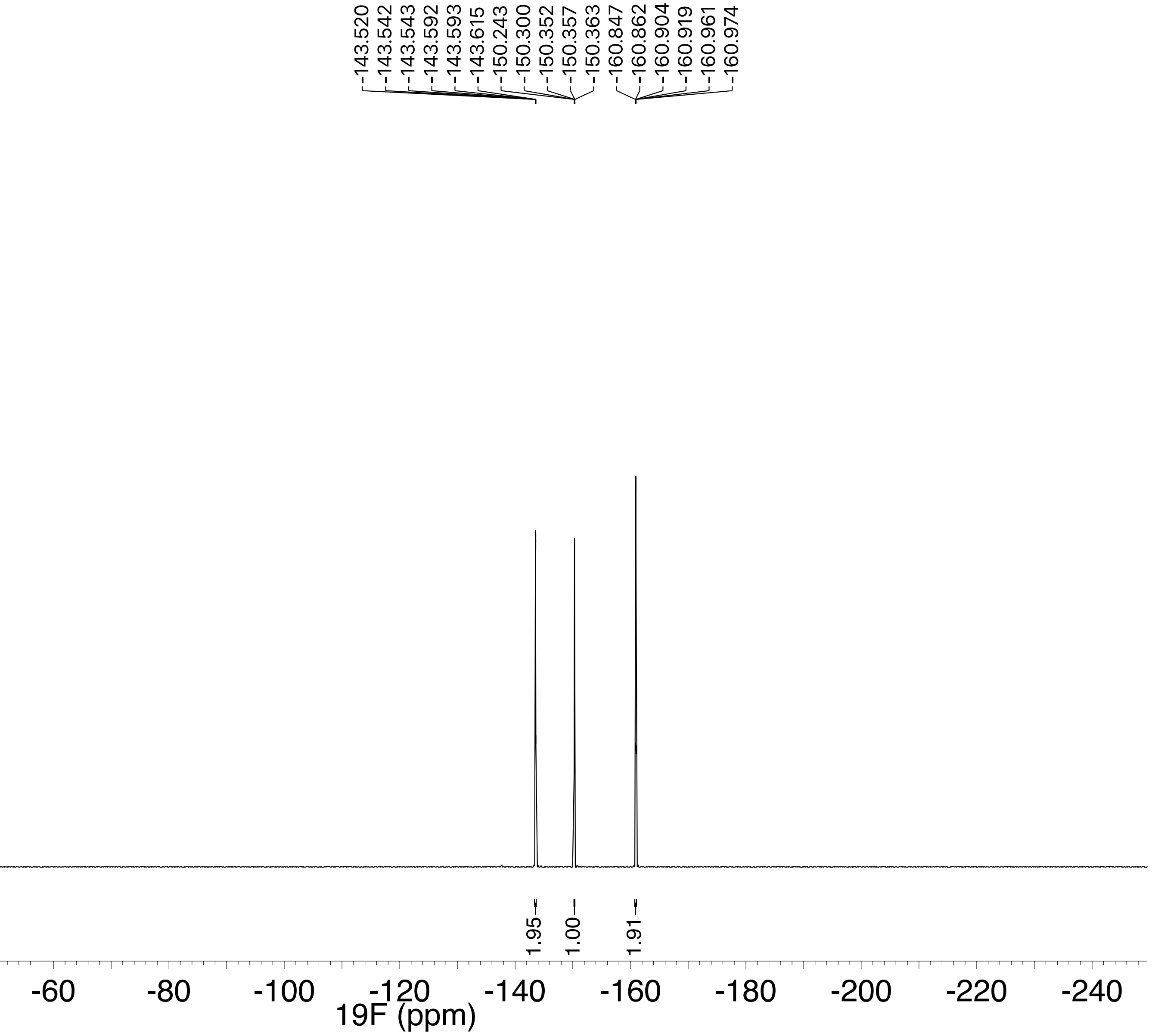

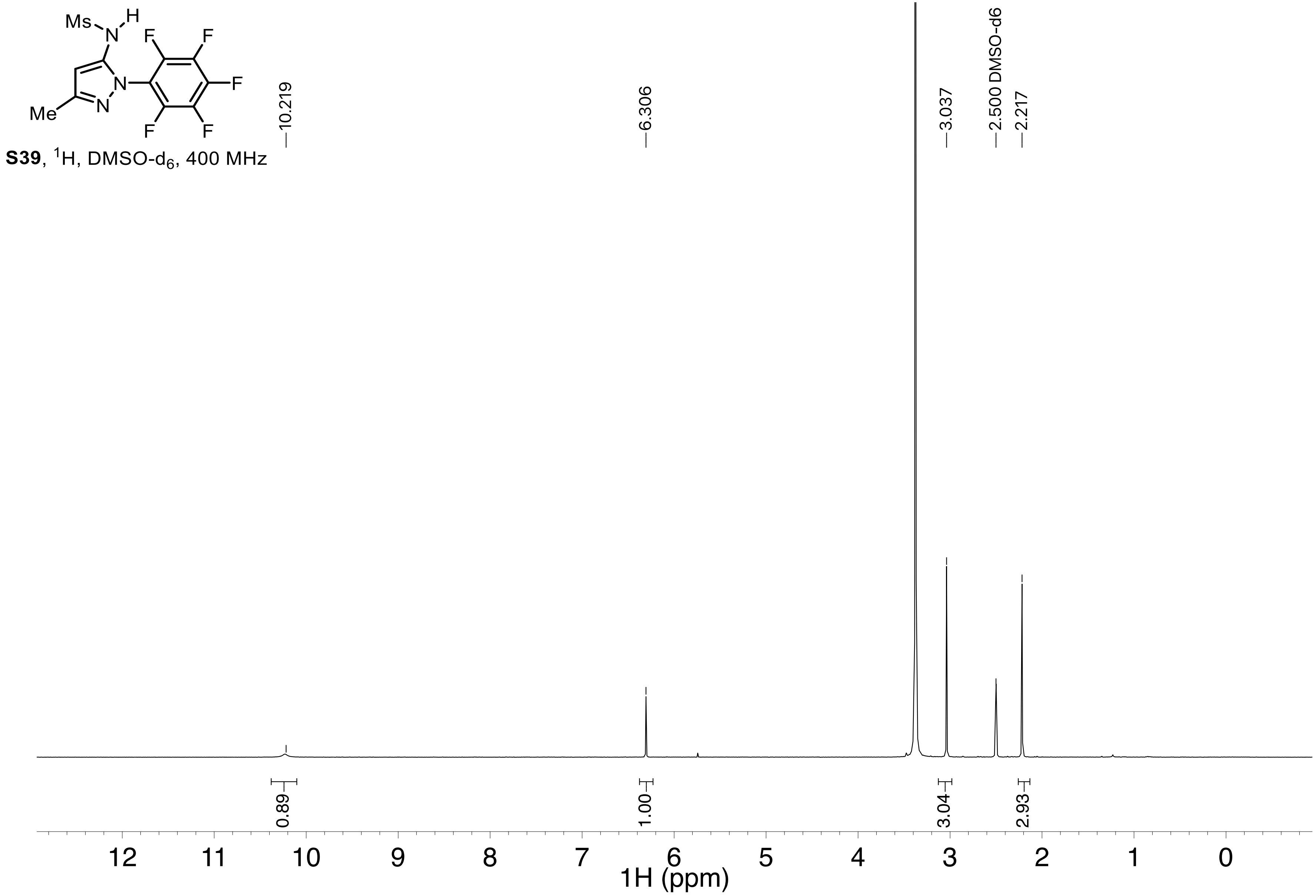

Ms
N
H
F
F
F
F
F
N
N
Me
S39, 1H, DMSO-d6, 400 MHz
10.219
6.306
3.037
2.500 DMSO-d6
2.217
0.89
1.00
3.04
2.93
12
11
10
9
8
7
6
5
4
3
2
1
0
1H (ppm)

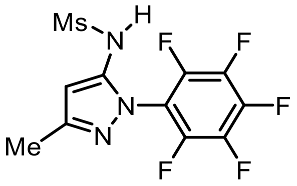

S39, $^{13}C\{^{1}H\}$, DMSO-d_6, 126 MHz

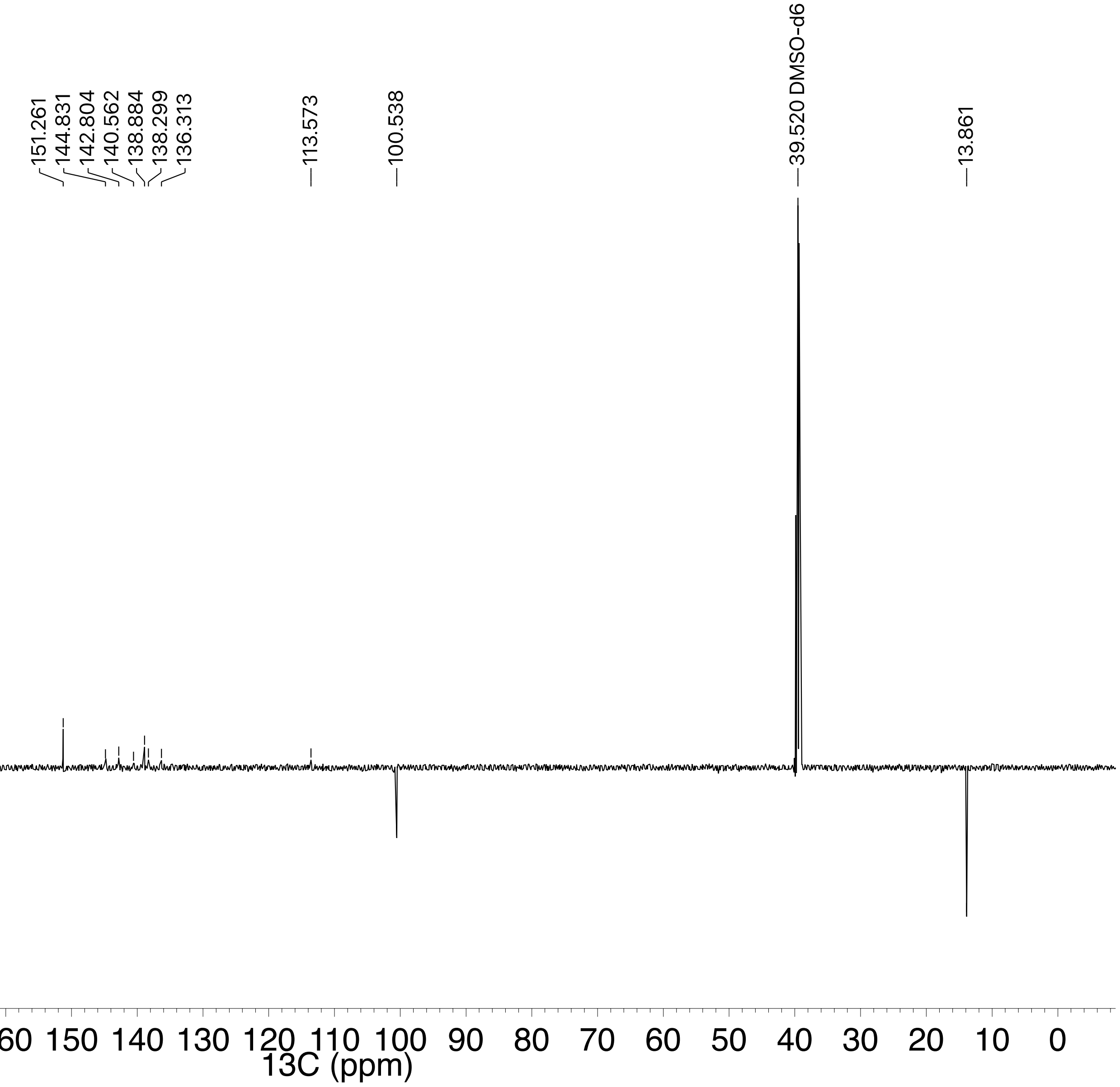

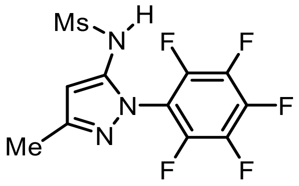

S39, $^{19}F\{^1H\}$, DMSO-d$_6$, 377 MHz

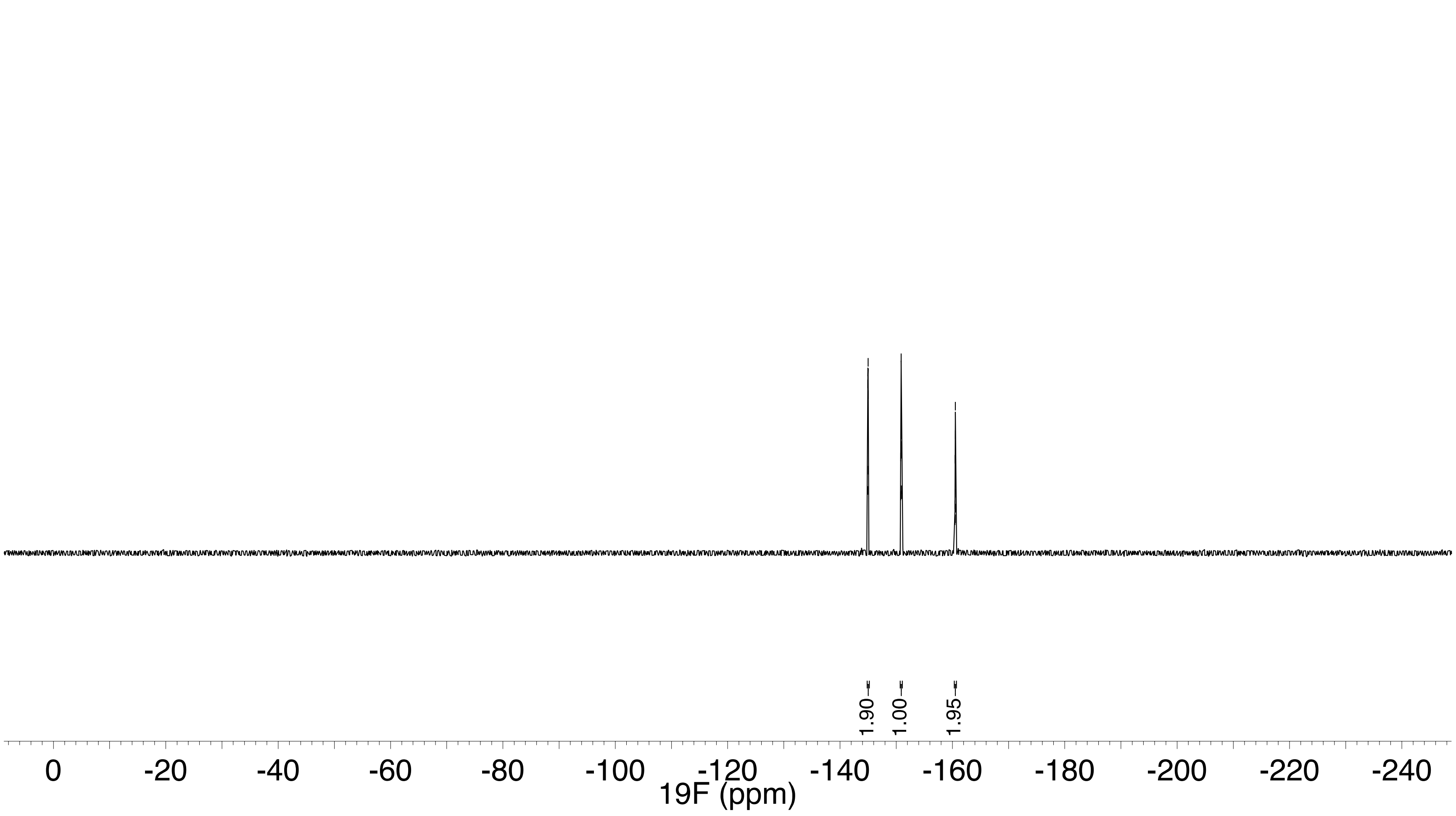

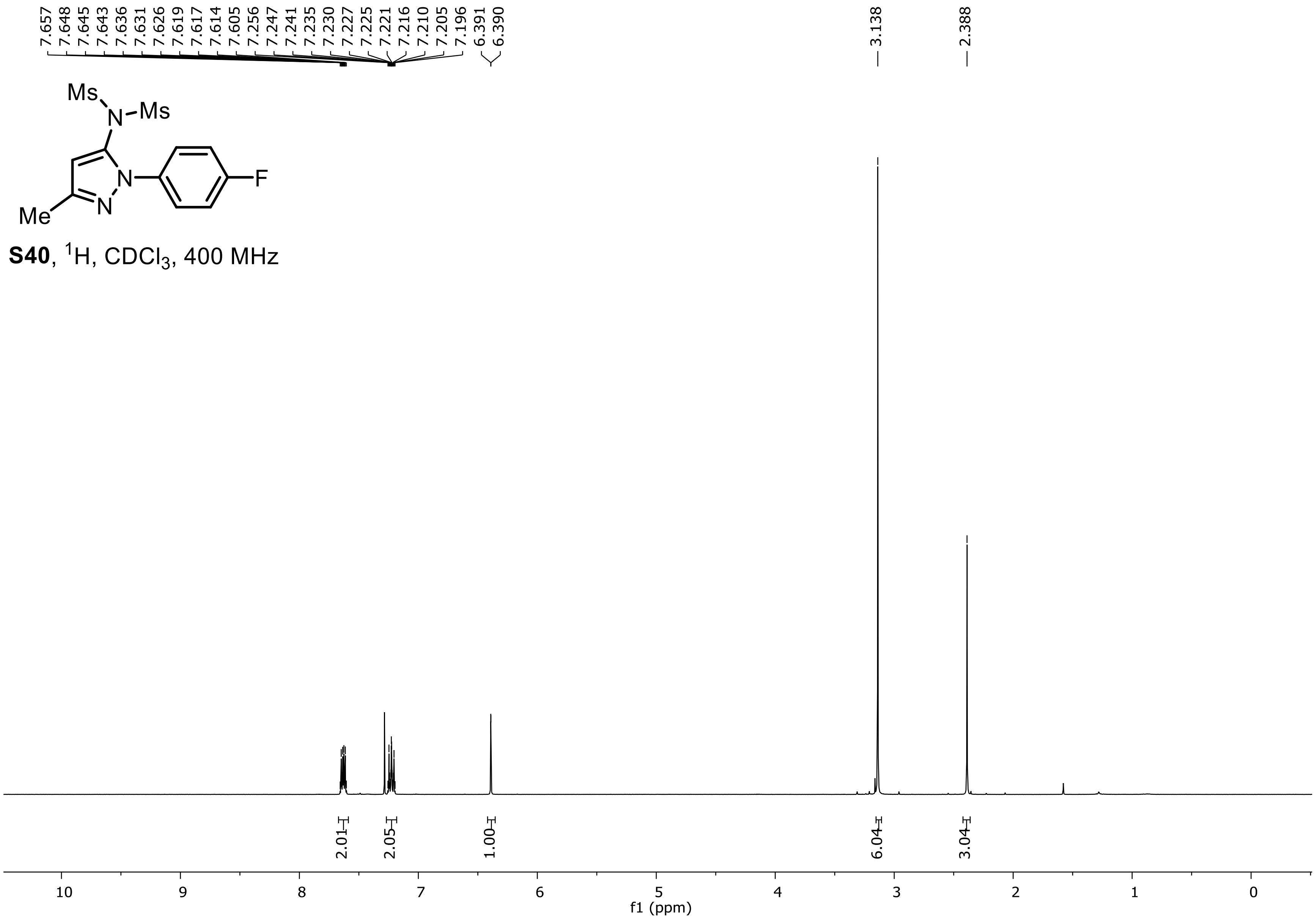
7.657
7.648
7.645
7.643
7.636
7.631
7.626
7.619
7.617
7.614
7.605
7.256
7.247
7.241
7.235
7.230
7.227
7.225
7.221
7.216
7.210
7.205
7.196
6.391
6.390
3.138
2.388
Ms
N
Ms
N
N
F
Me
S40, 1H, CDCl3, 400 MHz
2.01
2.05
1.00
6.04
3.04
10
9
8
7
6
5
4
3
2
1
0
f1 (ppm)

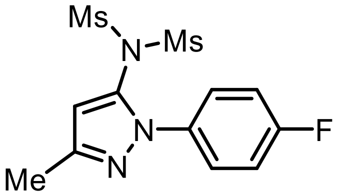

S40, $^{19}F\{^{1}H\}$, $CDCl_3$, 377 MHz

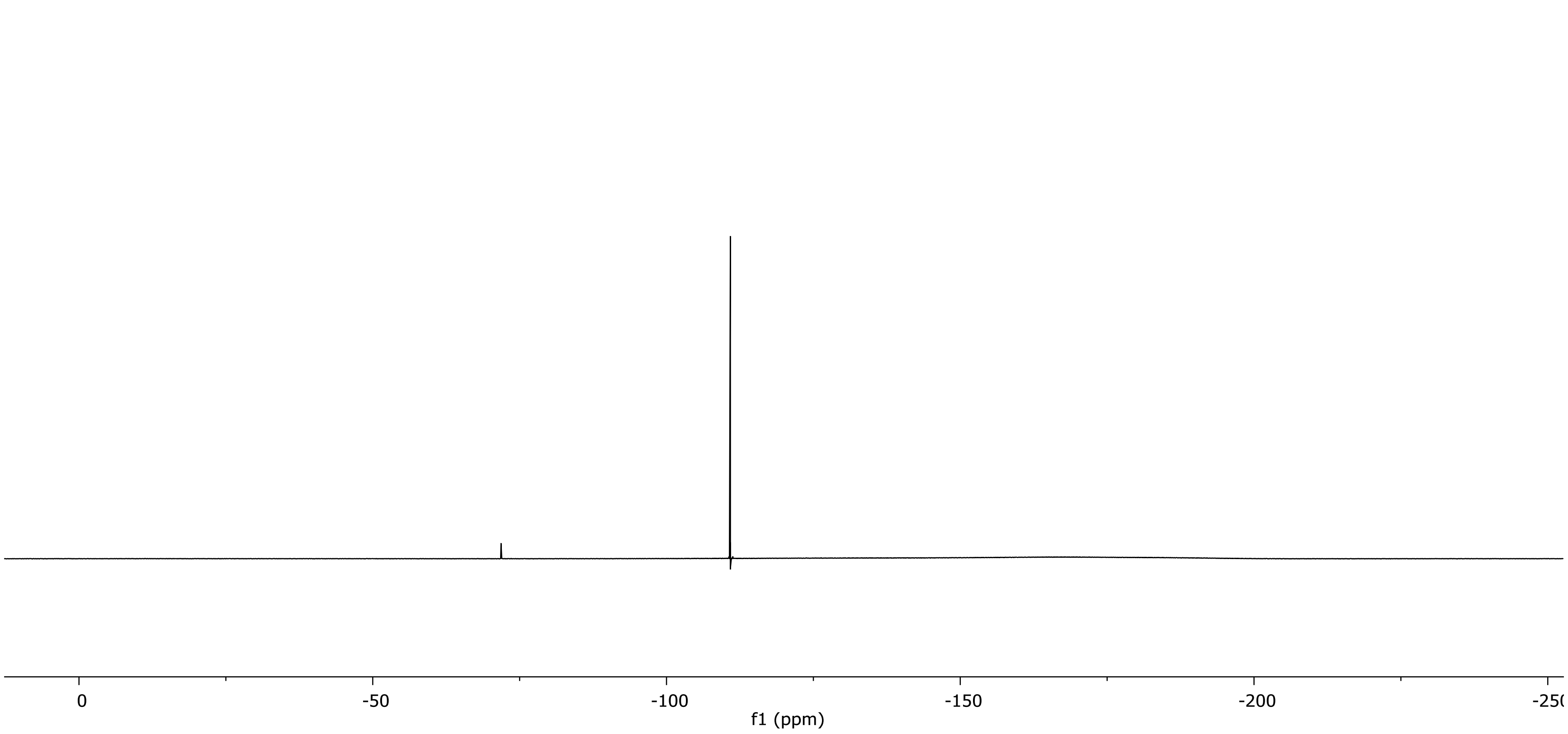

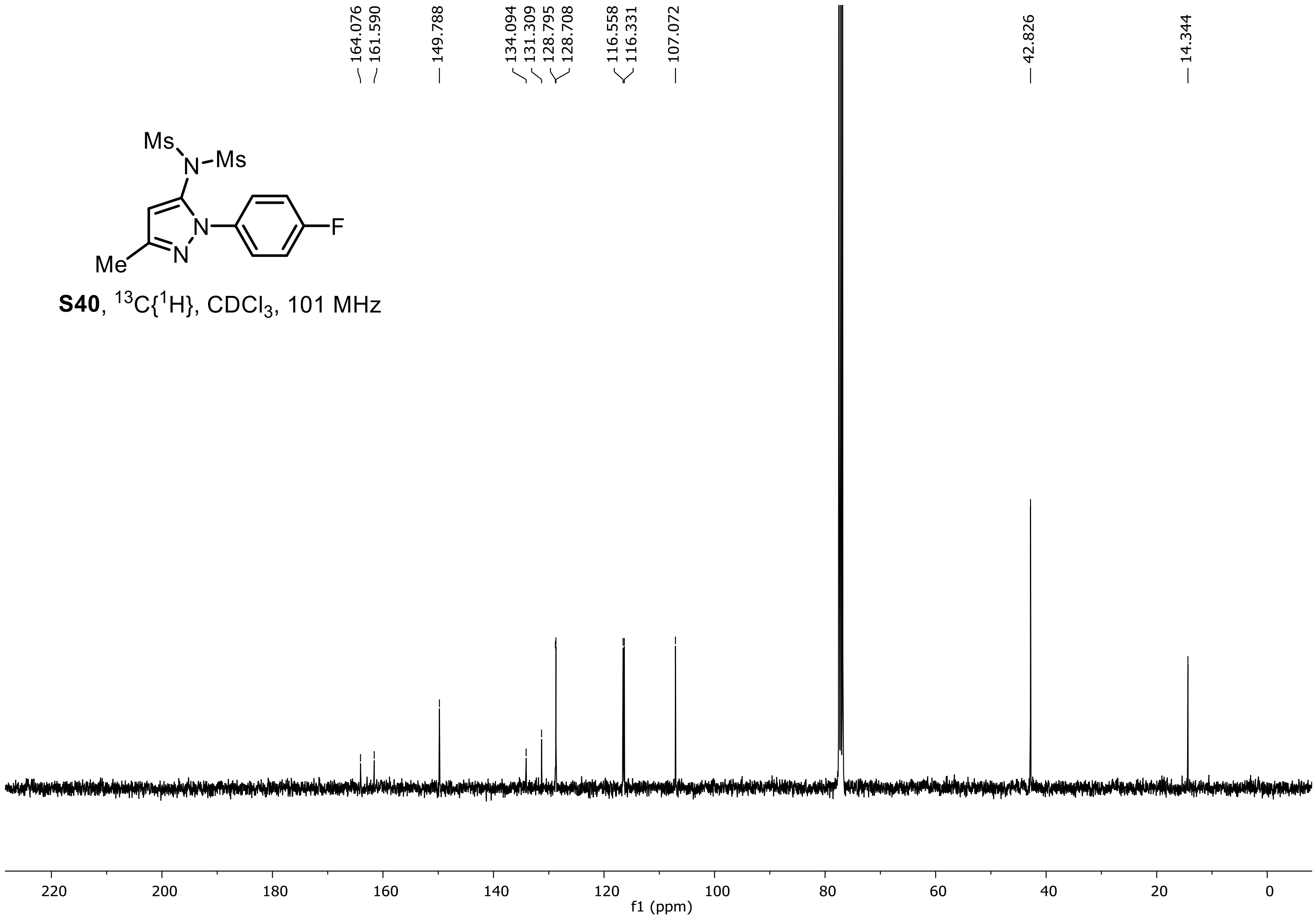
164.076
161.590
149.788
134.094
131.309
128.795
128.708
116.558
116.331
107.072
42.826
14.344
Ms
N
Ms
N
N
F
Me
S40, $^{13}C\{^1H\}$, $CDCl_3$, 101 MHz
220
200
180
160
140
120
100
80
60
40
20
0
f1 (ppm)

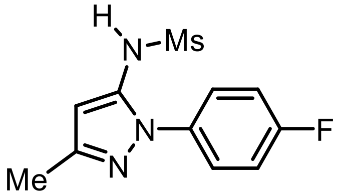

S41, ^{1}H, $CDCl_3$, 400 MHz

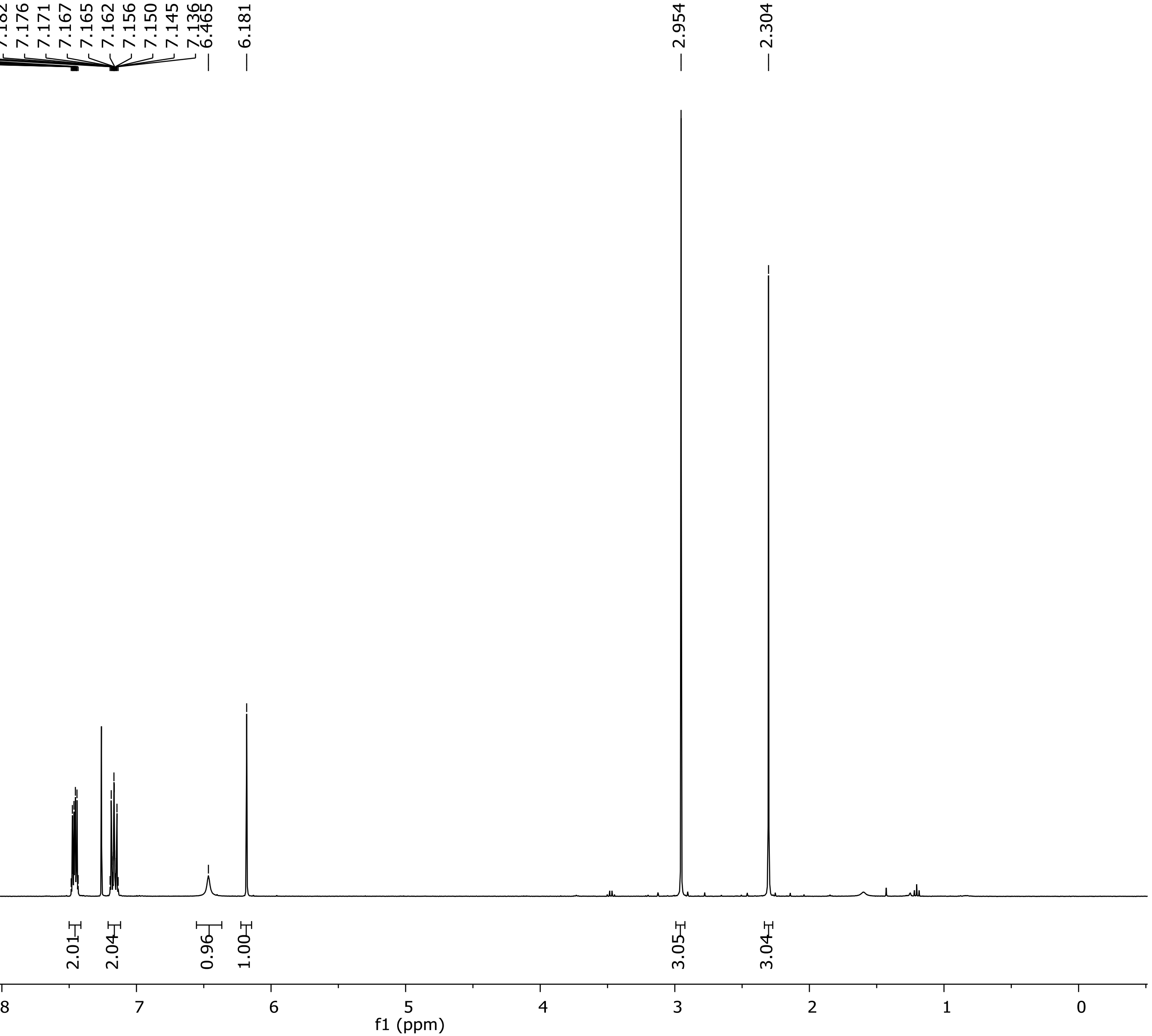

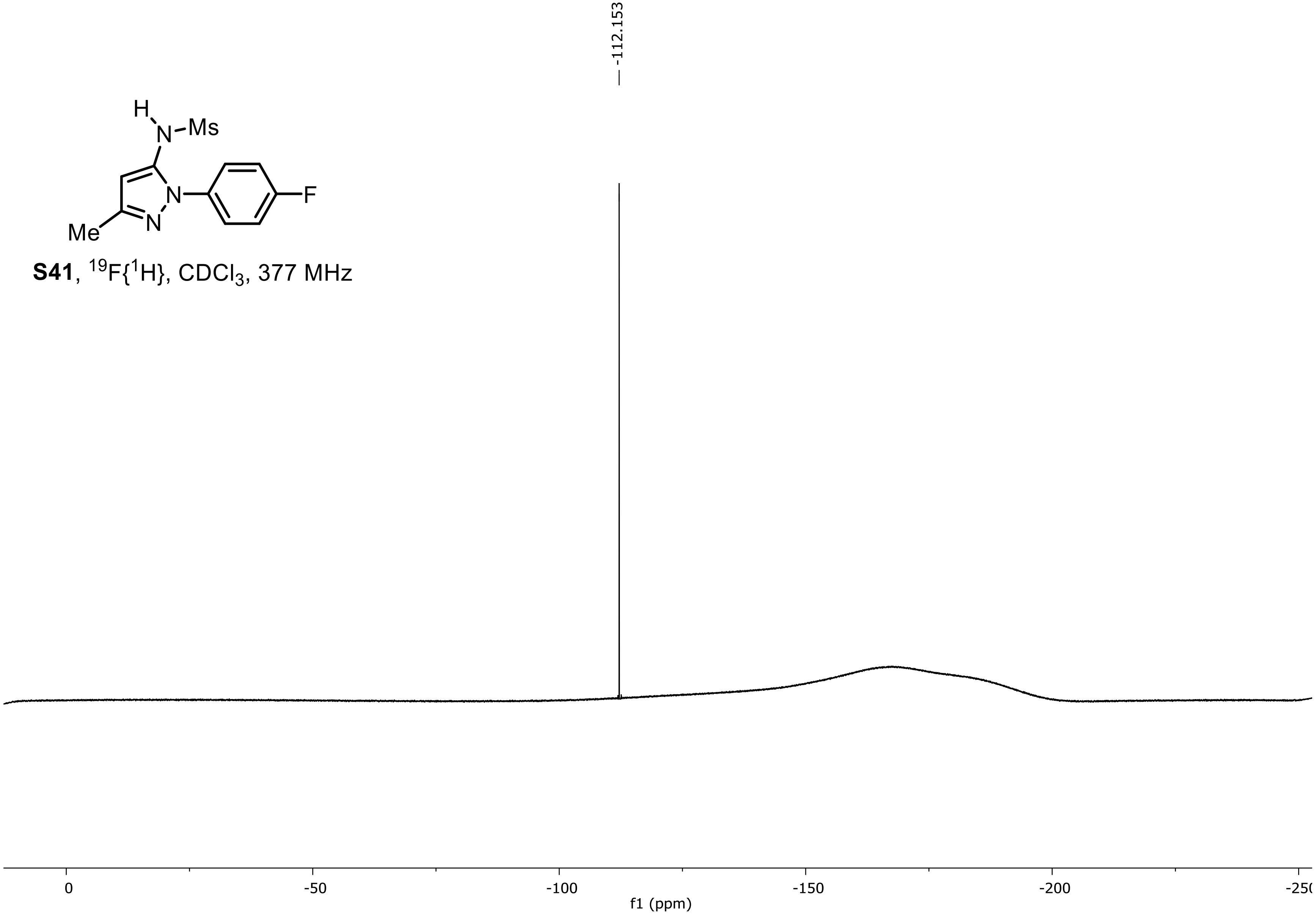

S41, $^{19}F\{^{1}H\}$, $CDCl_3$, 377 MHz

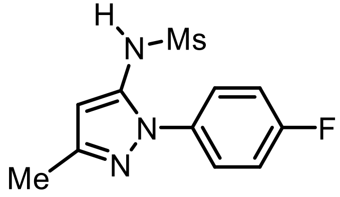

S41, $^{13}C\{^{1}H\}$. $CDCl_3$, 101 MHz

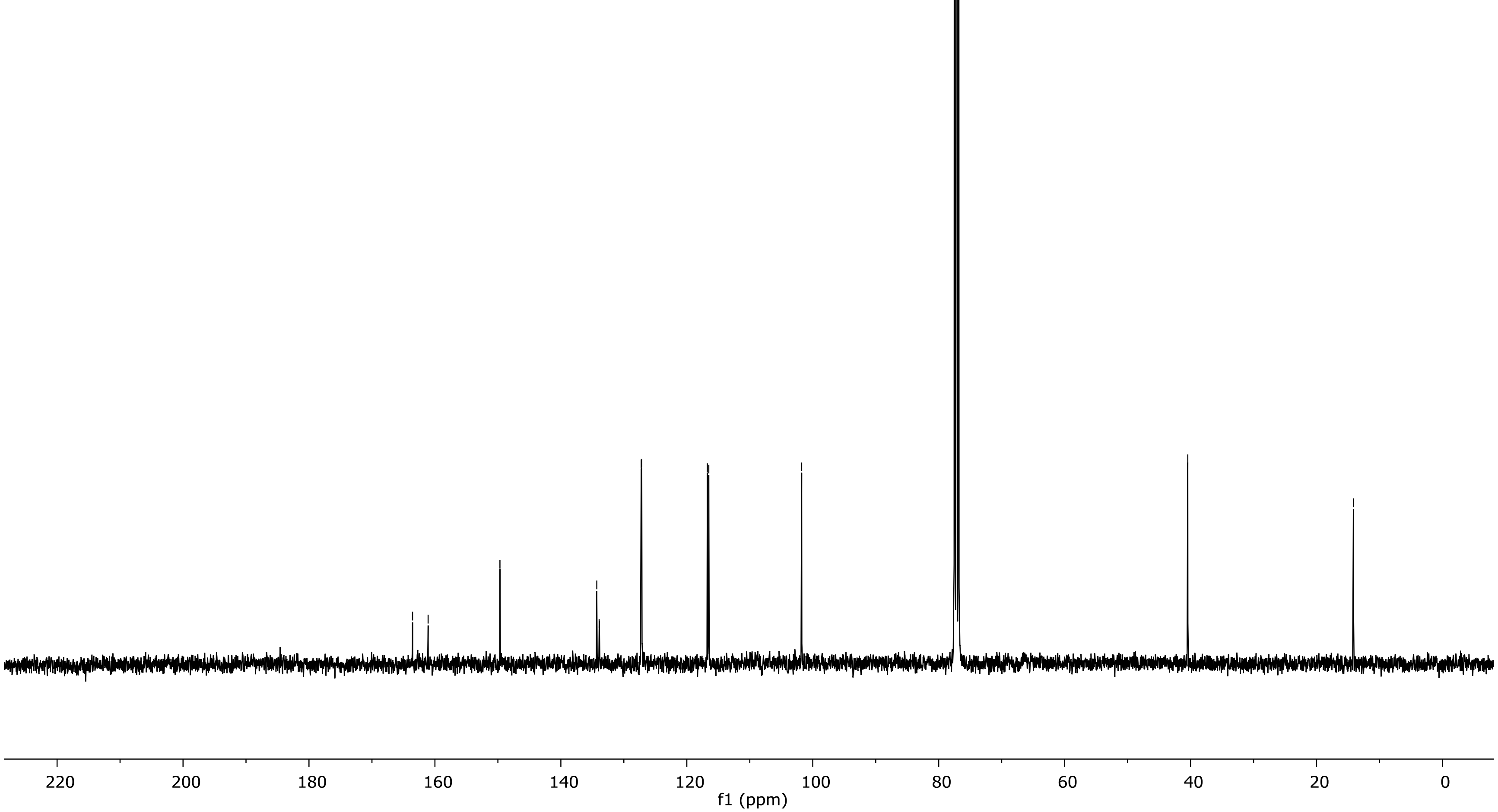

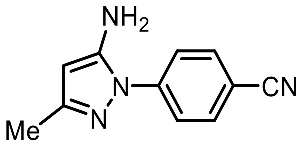

S42, ^{1}H, $CDCl_3$, 400 MHz

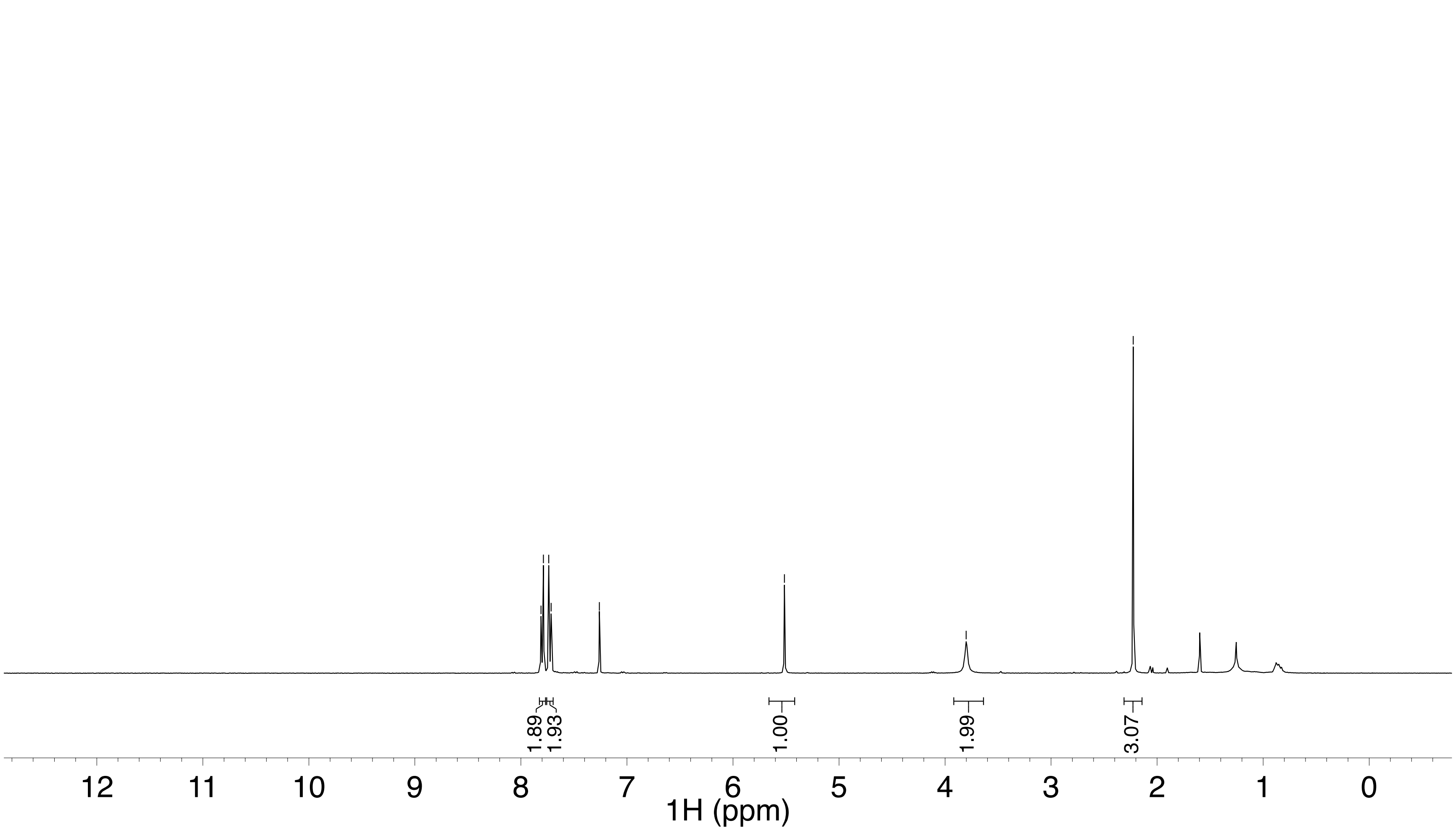

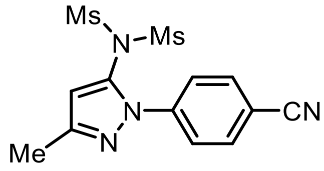

S43, ${}^{1}H$, $CDCl_3$, 500 MHz

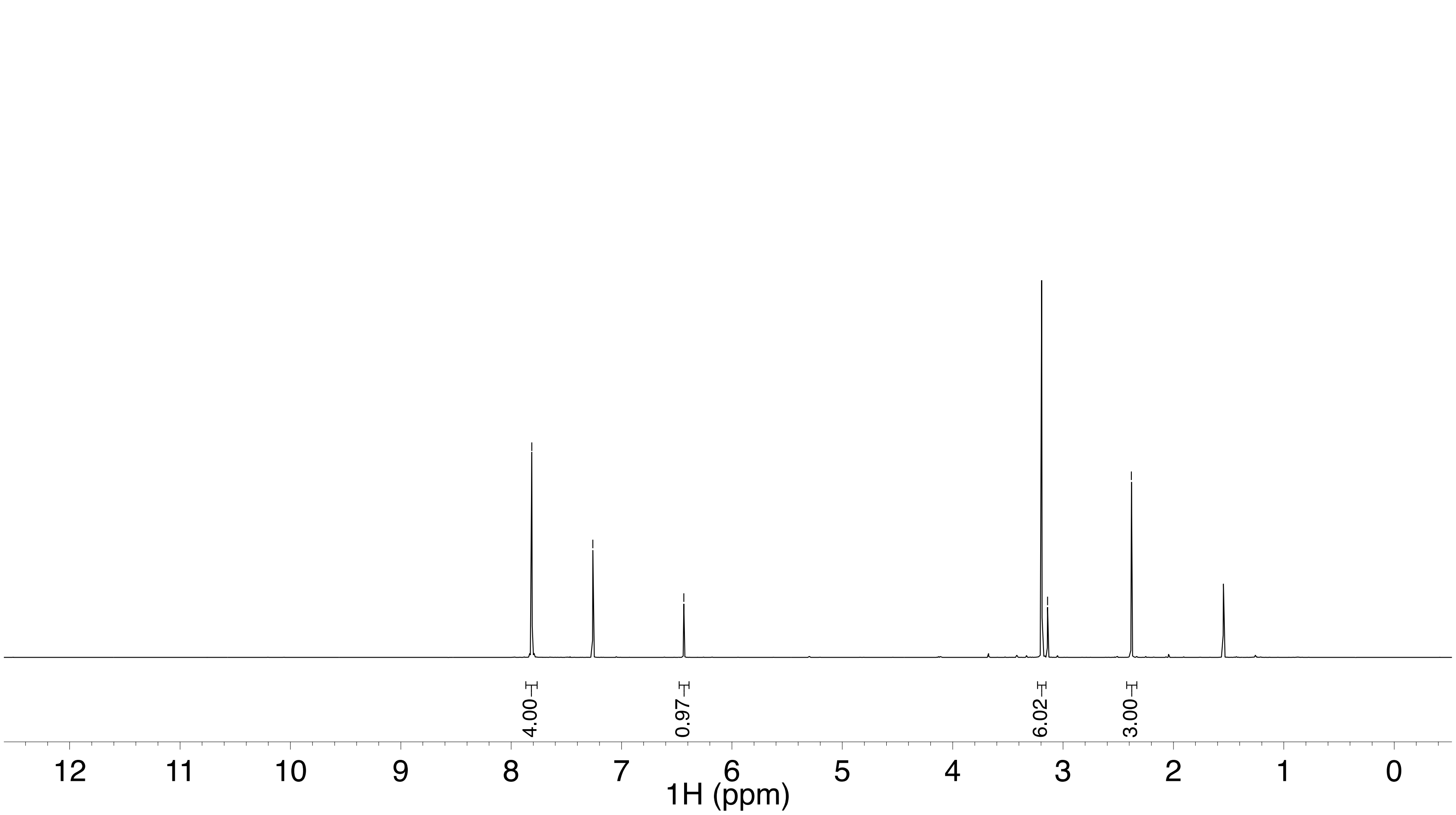

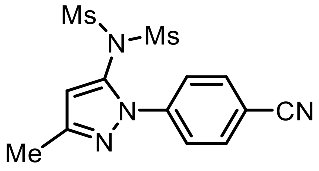

S43, $^{13}C\{^{1}H\}$, $CDCl_3$, 126 MHz

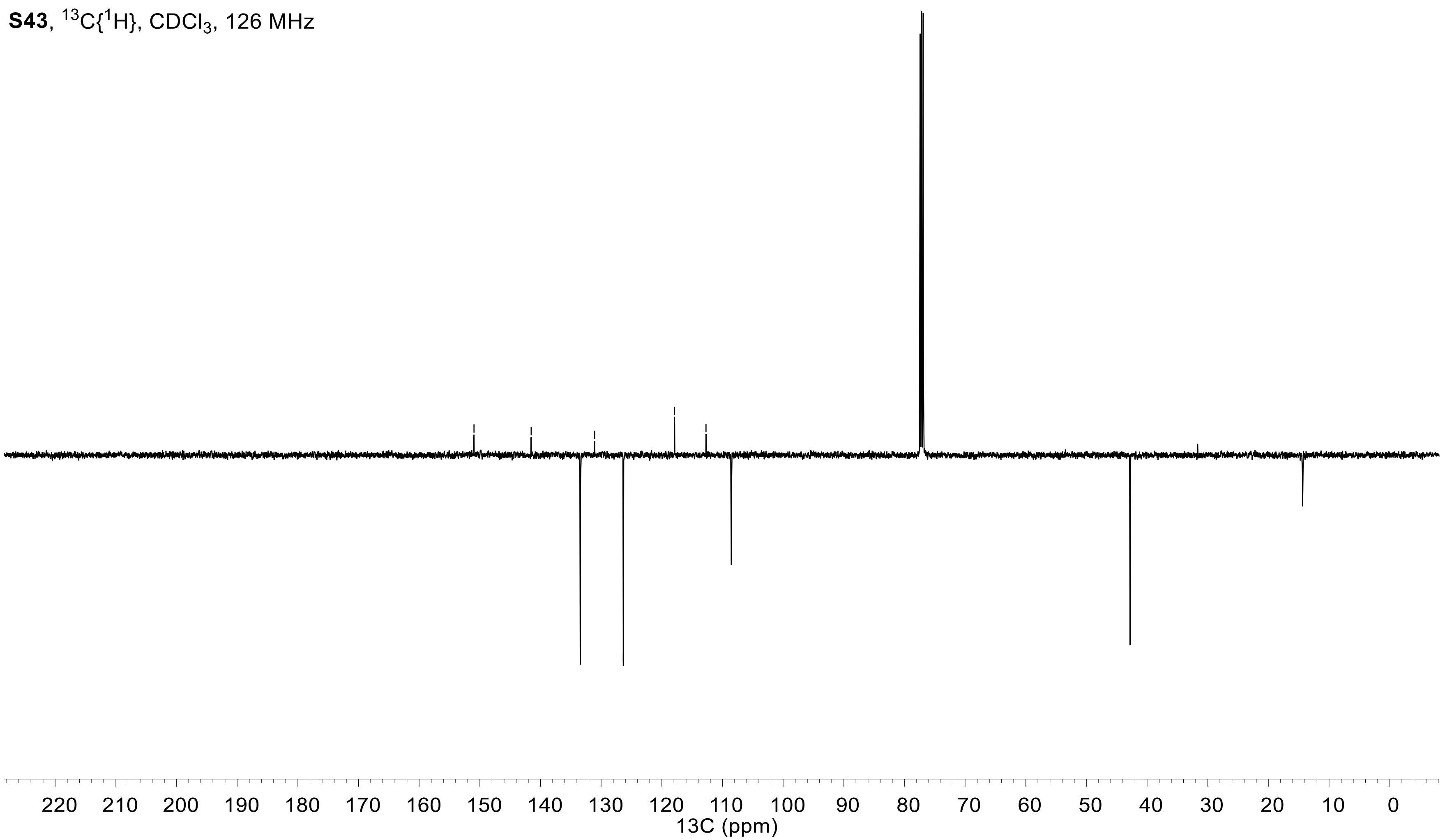

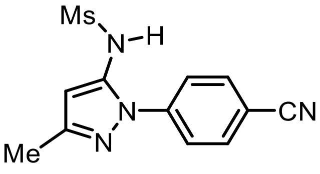

S7, ^{1}H, $CDCl_3$, 500 MHz

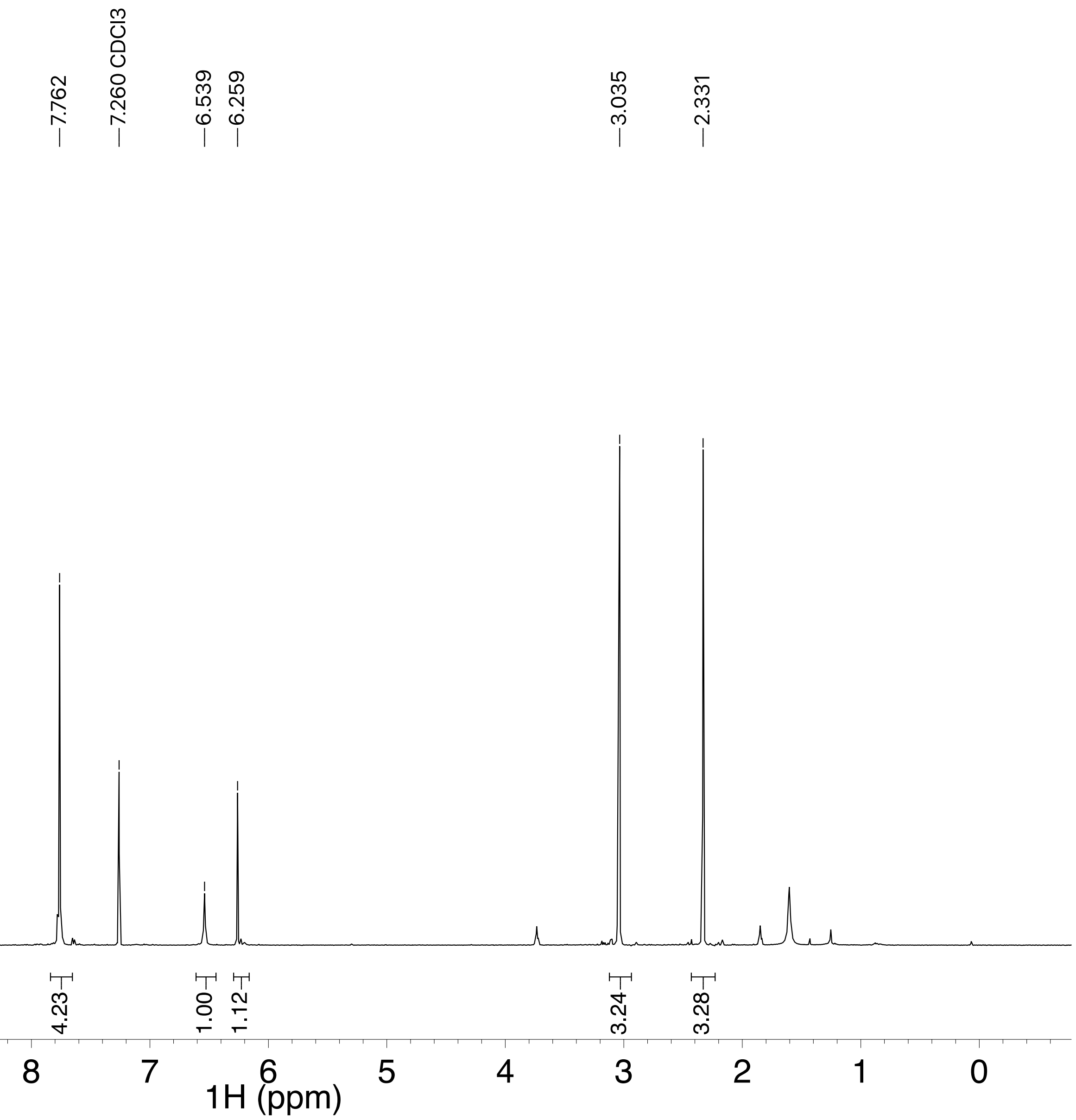

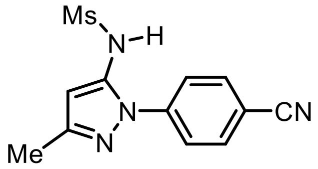

S7, $^{13}C\{^1H\}$, $CDCl_3$, 126 MHz

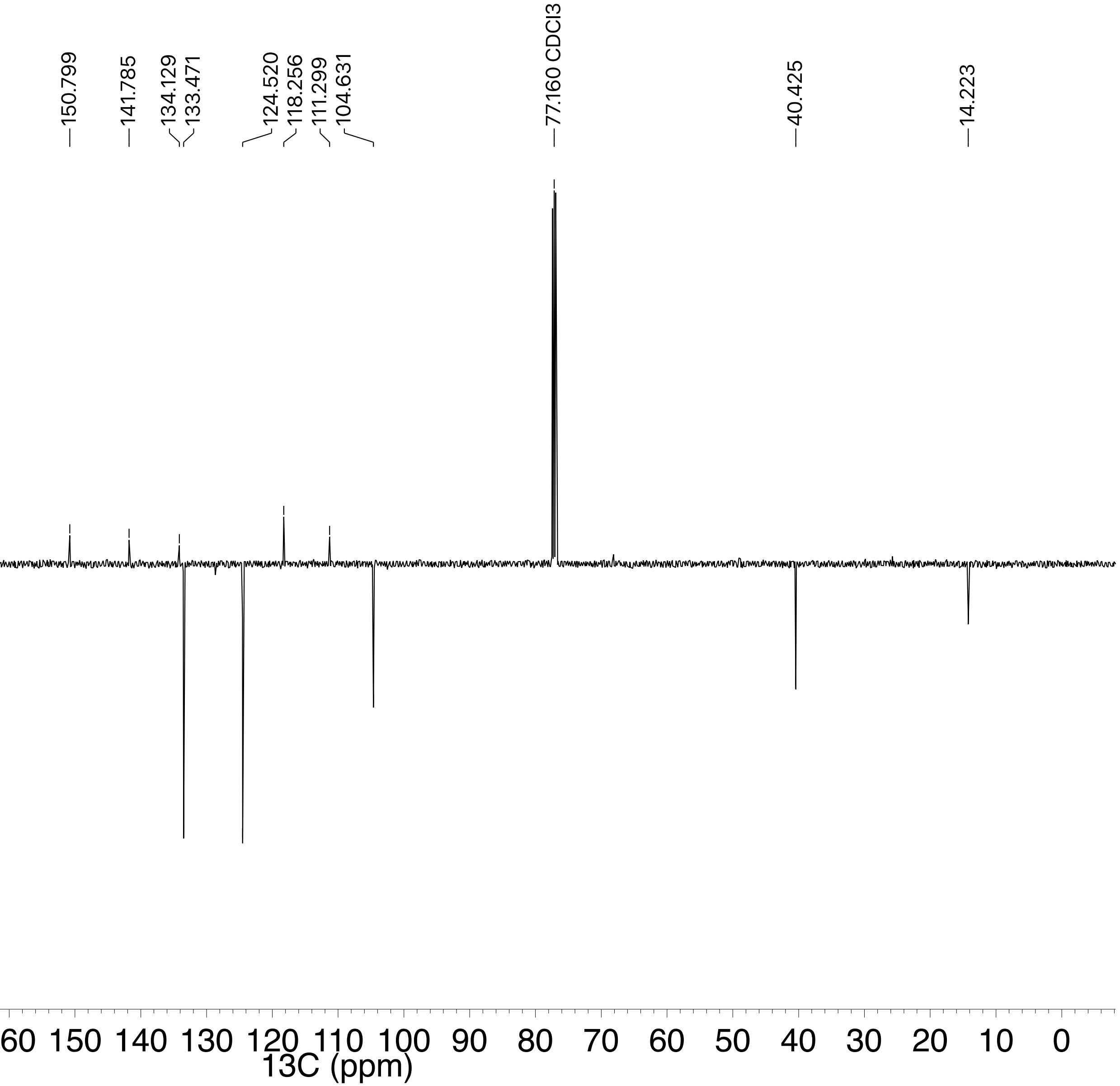

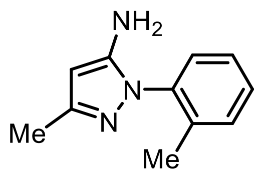

S44, ^{1}H, $CDCl_3$, 500 MHz

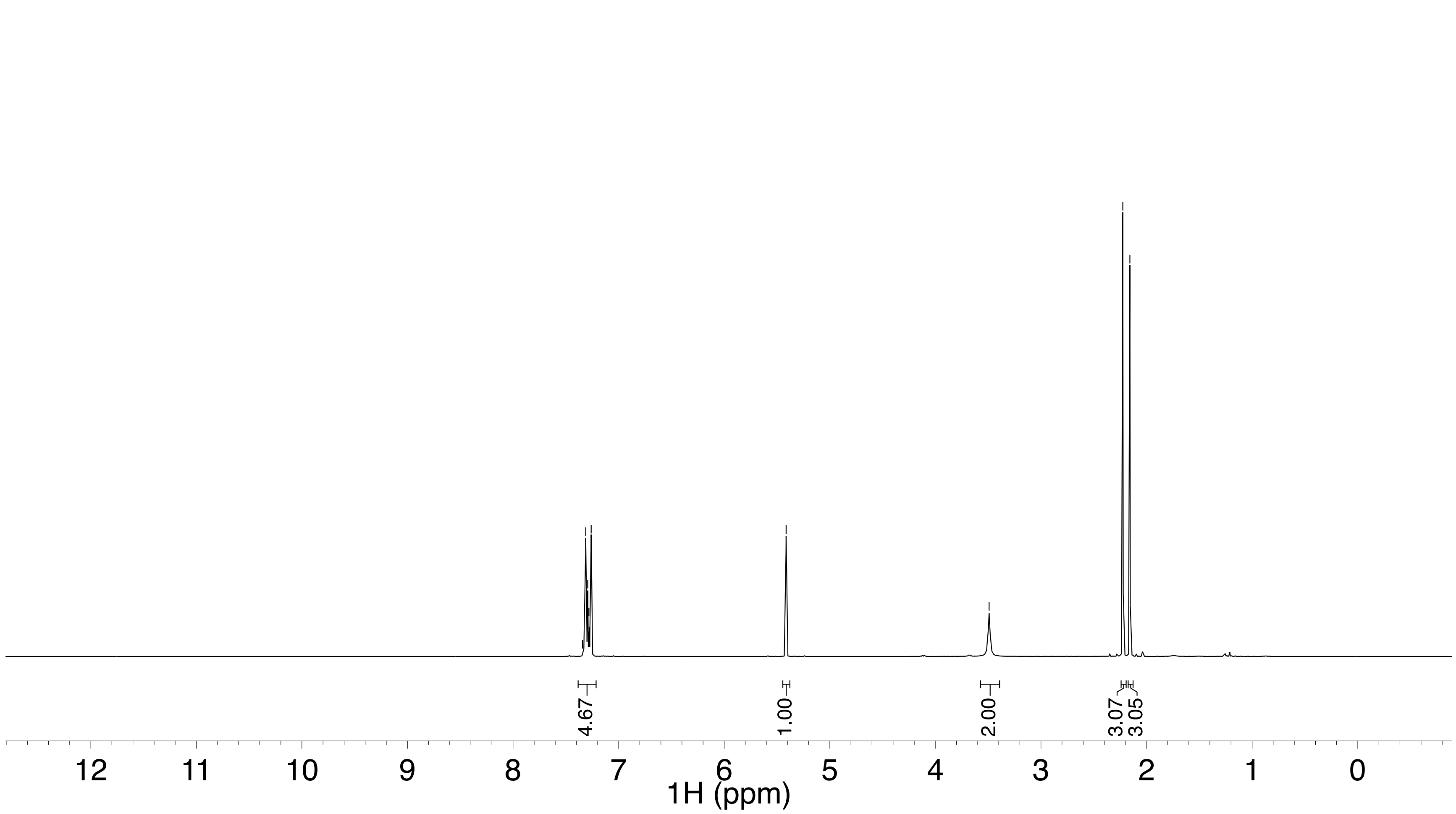

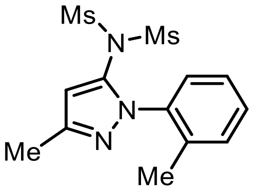

S45, ^{1}H, $CDCl_3$, 500 MHz

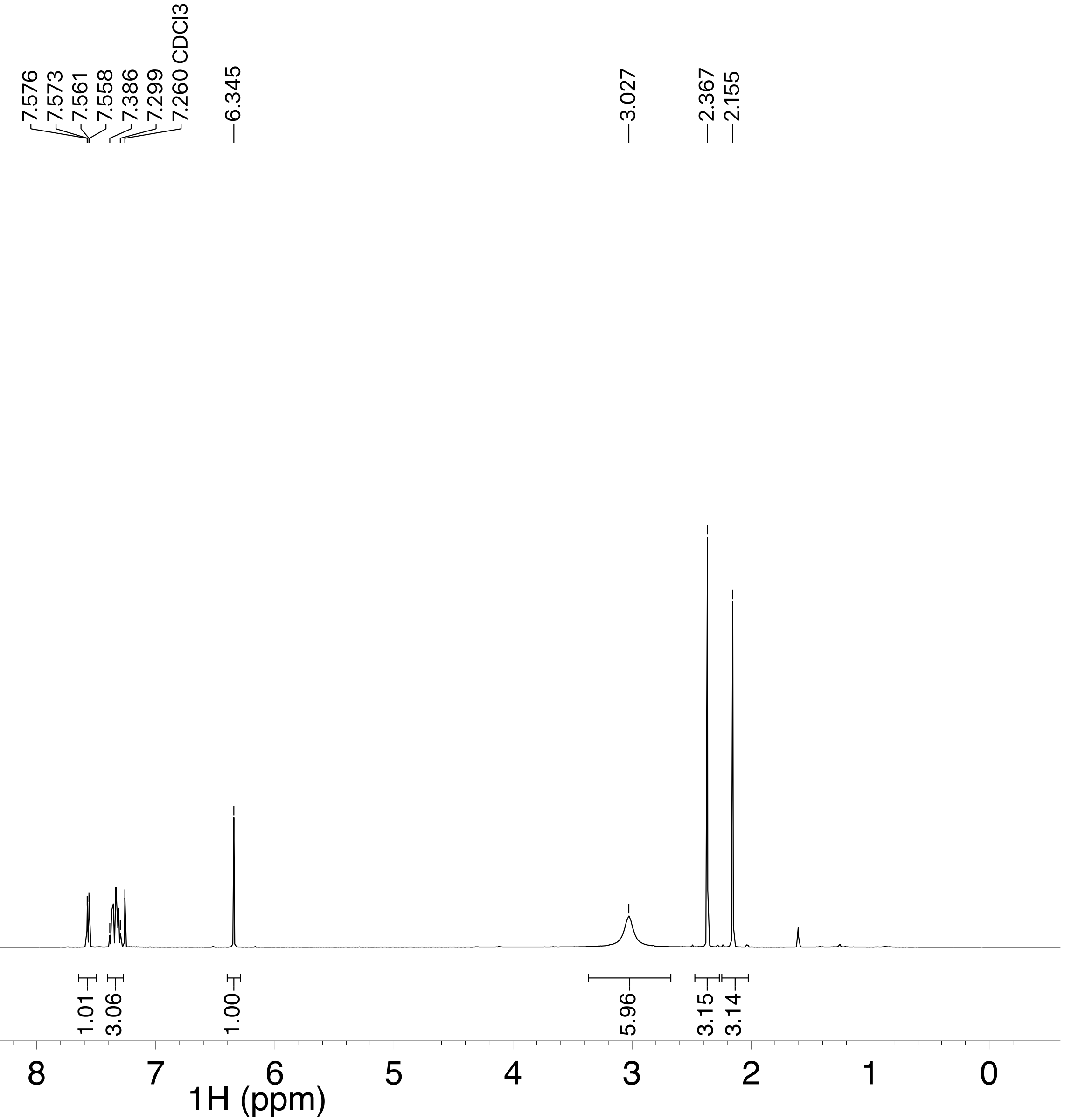

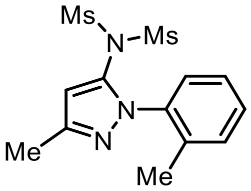

S45, $^{13}C\{^{1}H\}$, $CDCl_3$, 126 MHz

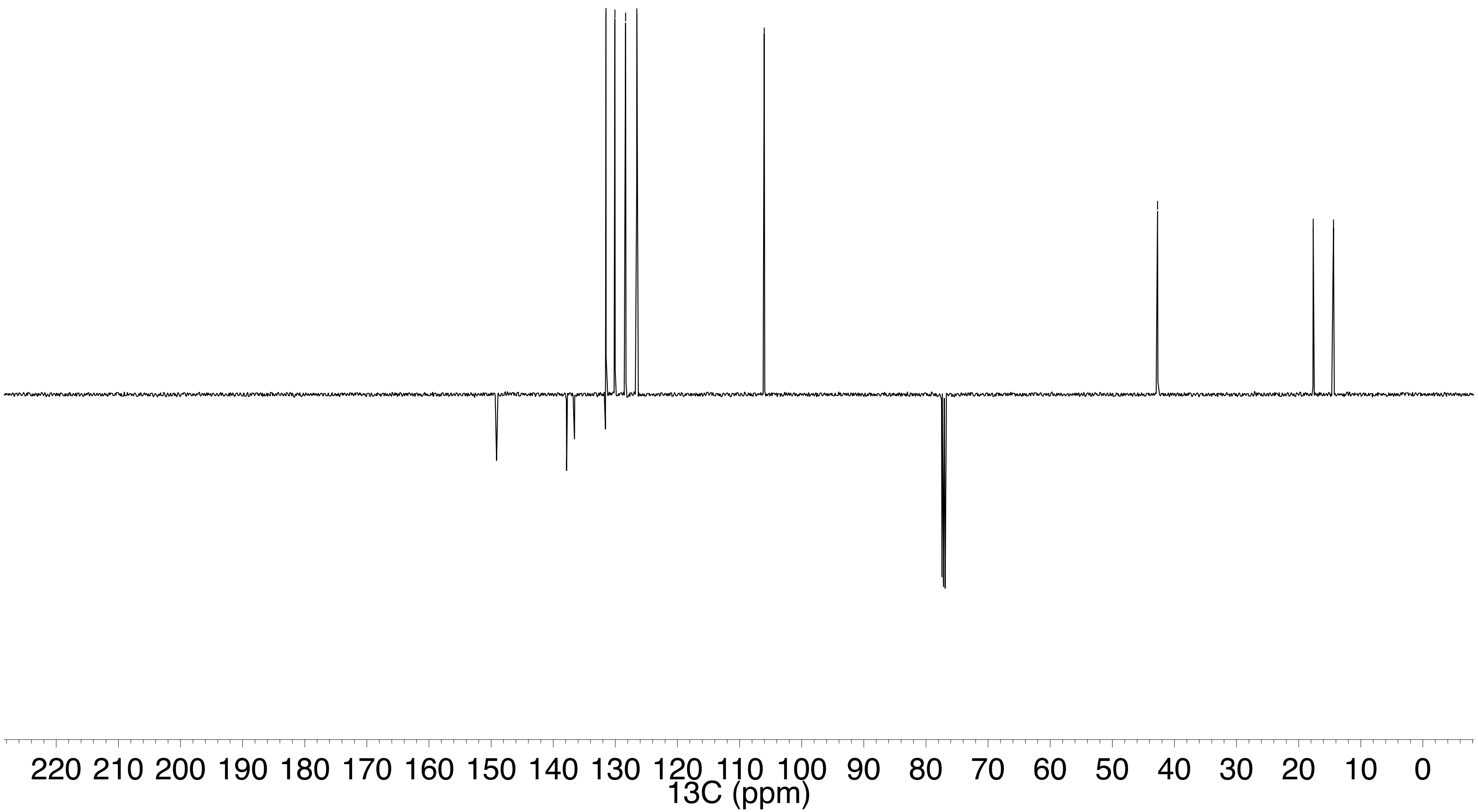

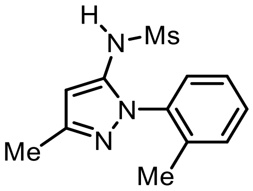

S46, ^{1}H, $CDCl_3$, 500 MHz

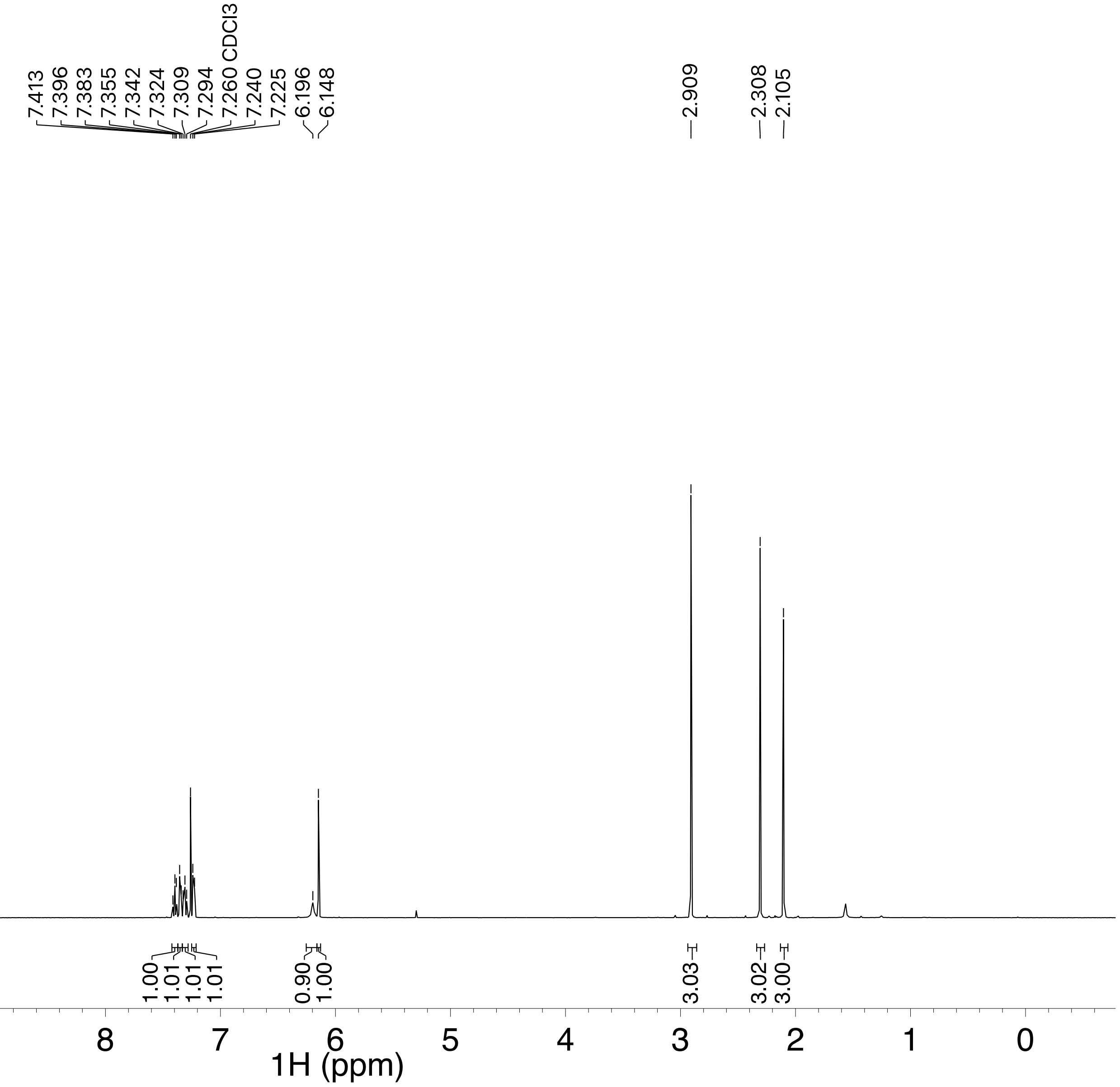

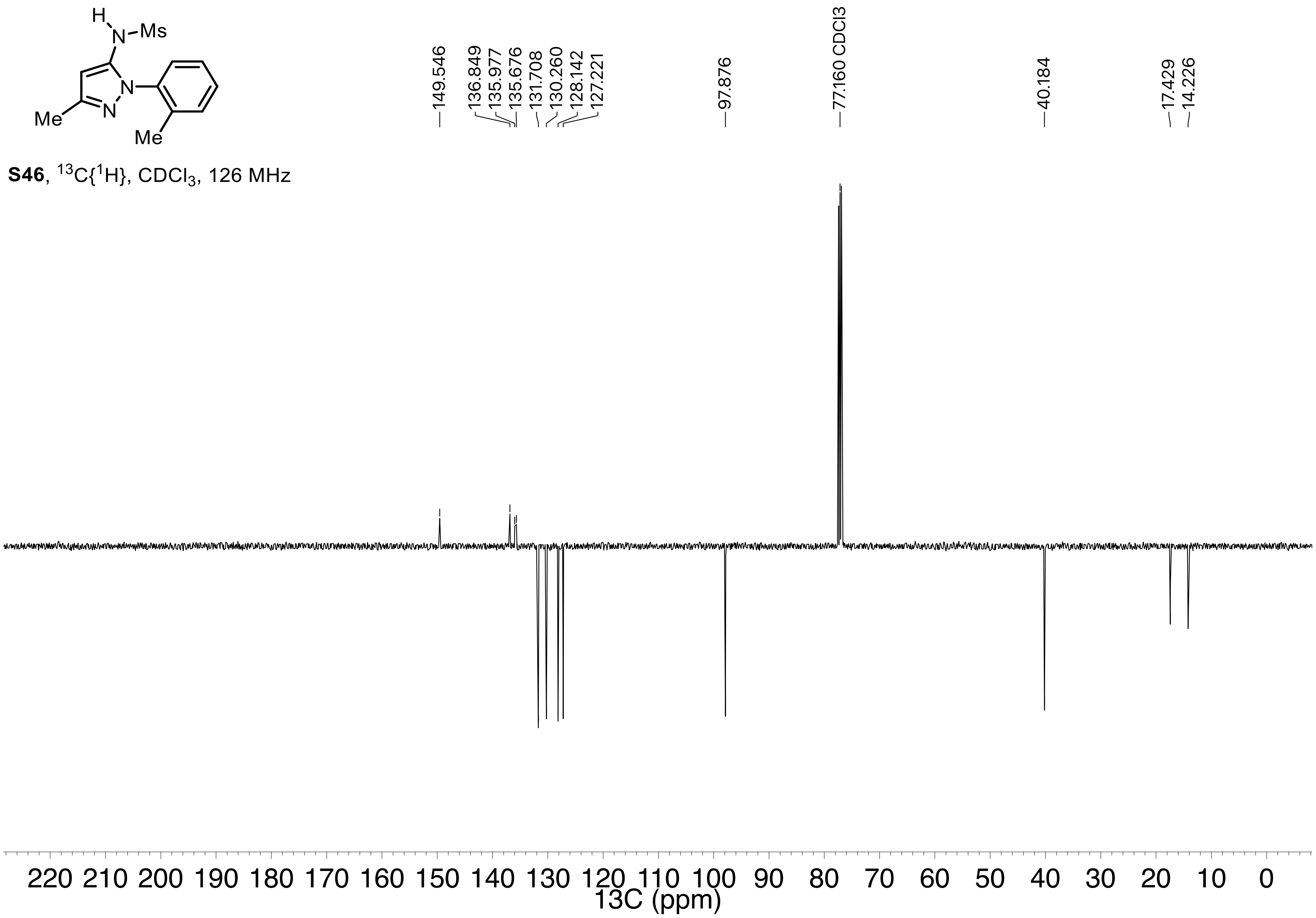

S46, $^{13}C\{^{1}H\}$, $CDCl_3$, 126 MHz

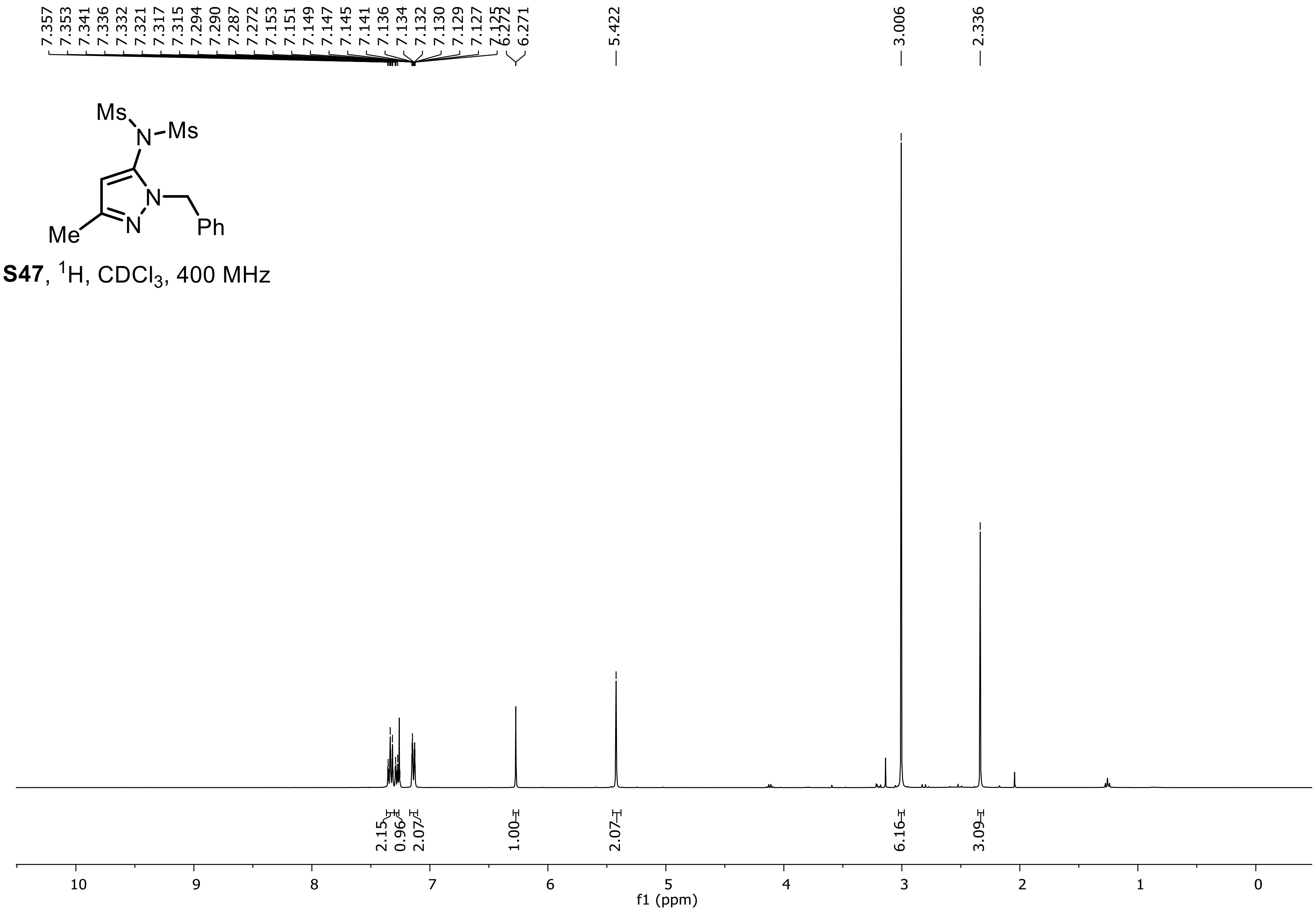

S47, ^{1}H, $CDCl_3$, 400 MHz

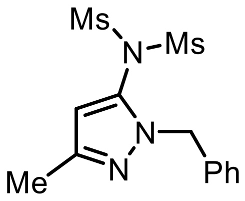

S47, $^{13}C\{^{1}H\}$, $CDCl_3$, 101 MHz

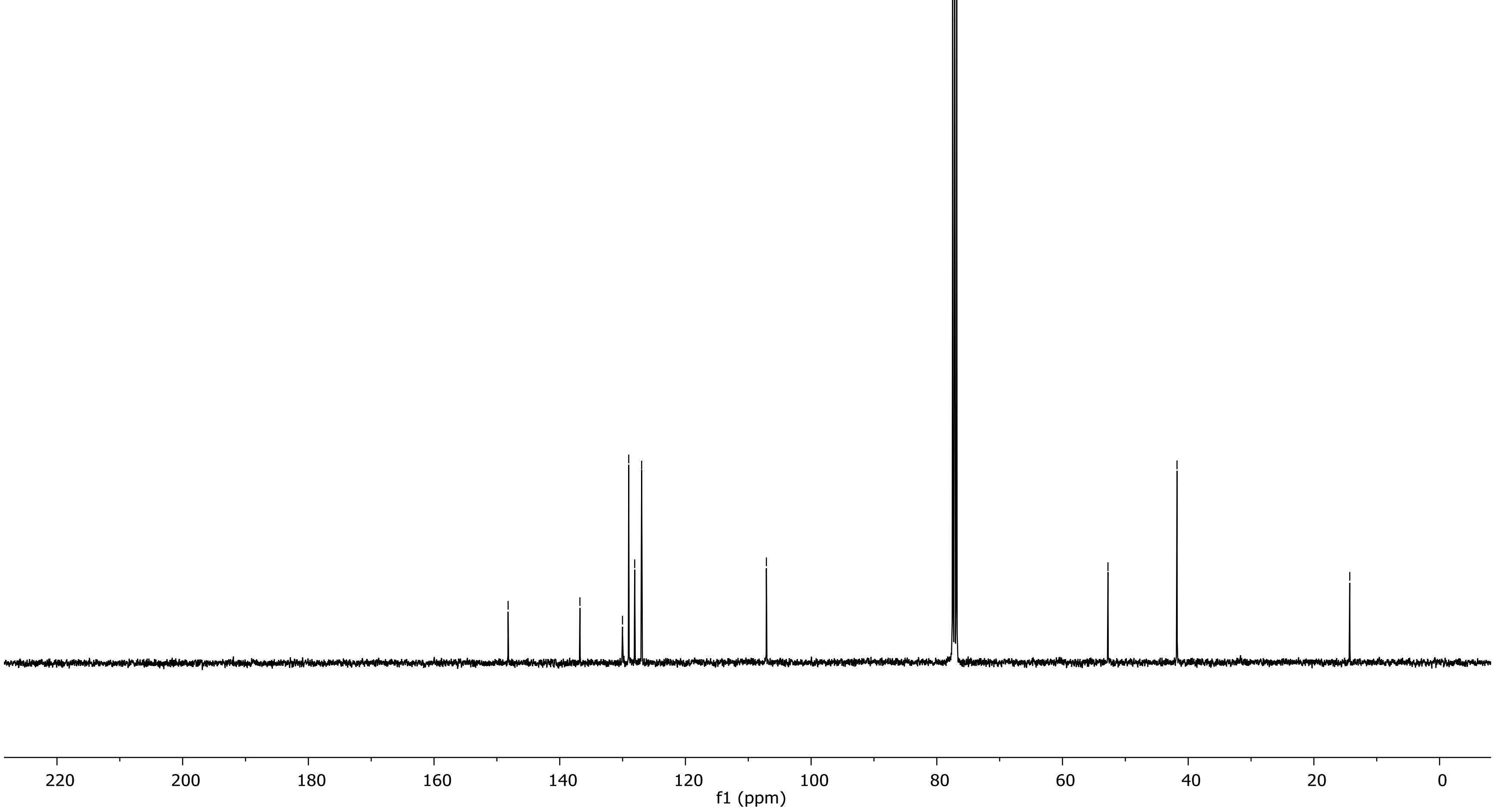

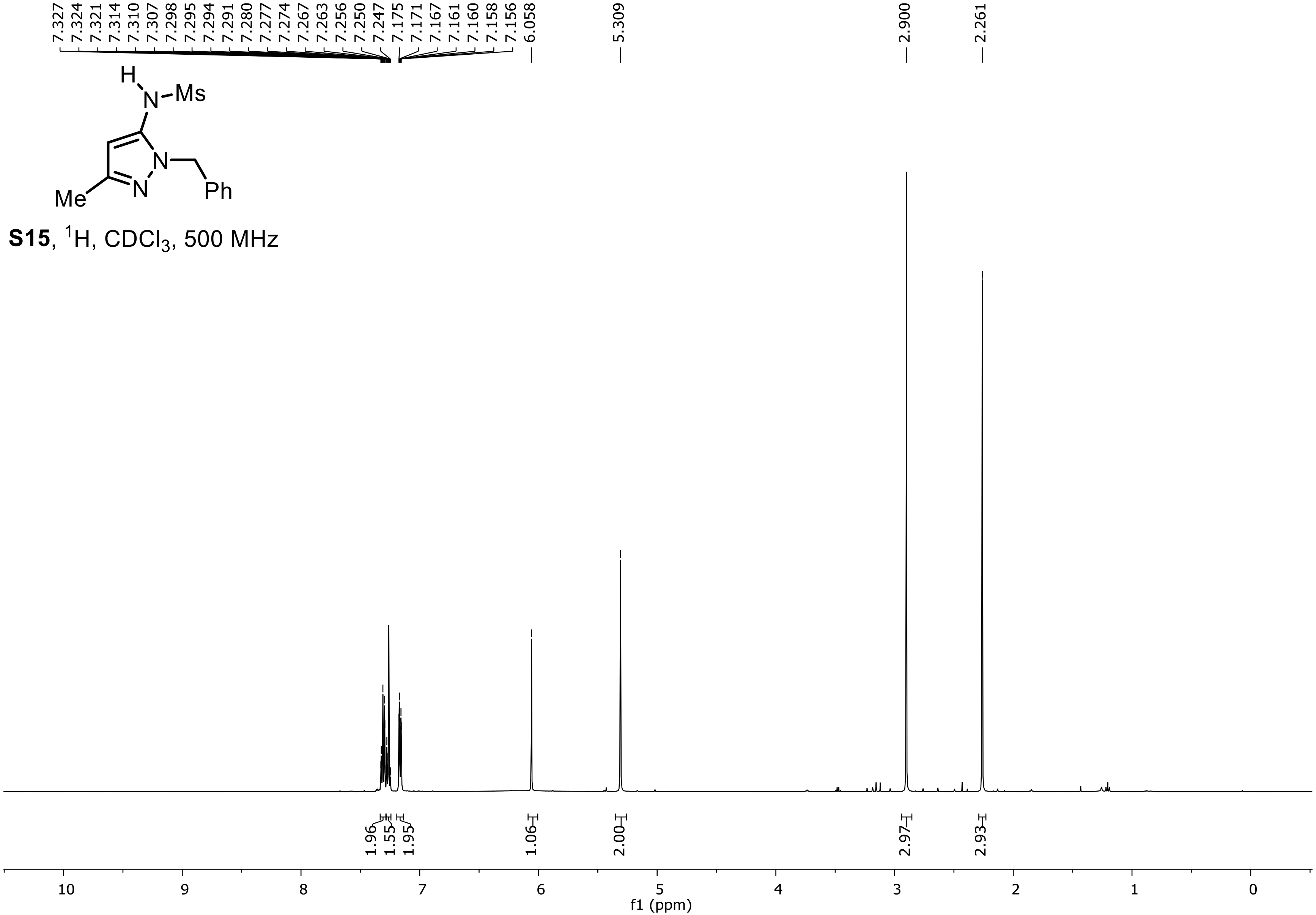

S15, ^{1}H, $CDCl_3$, 500 MHz

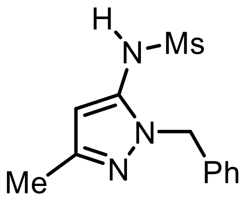

S15, $^{13}C\{^{1}H\}$, $CDCl_3$, 101 MHz

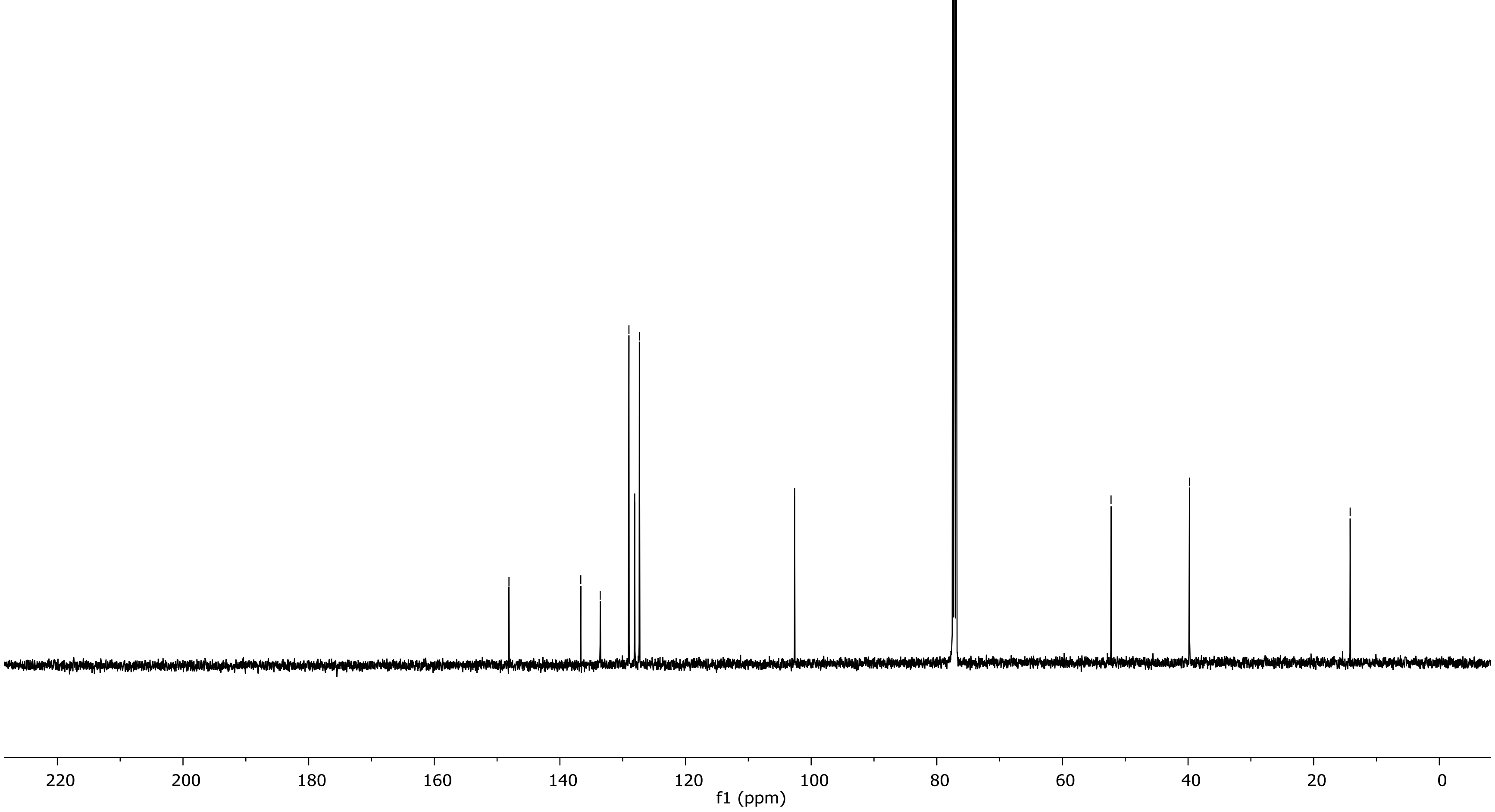

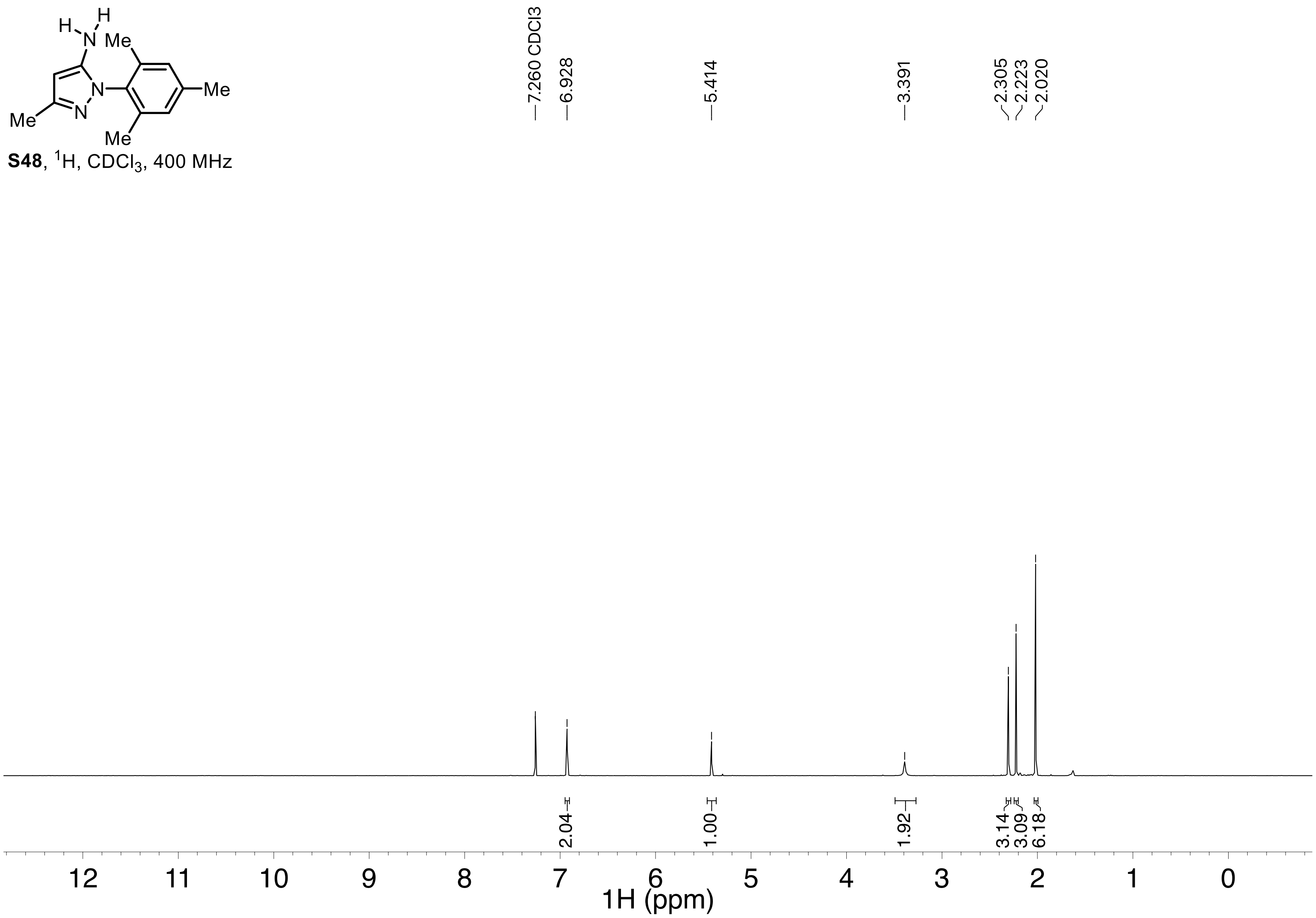

S48, ^{1}H, $CDCl_3$, 400 MHz

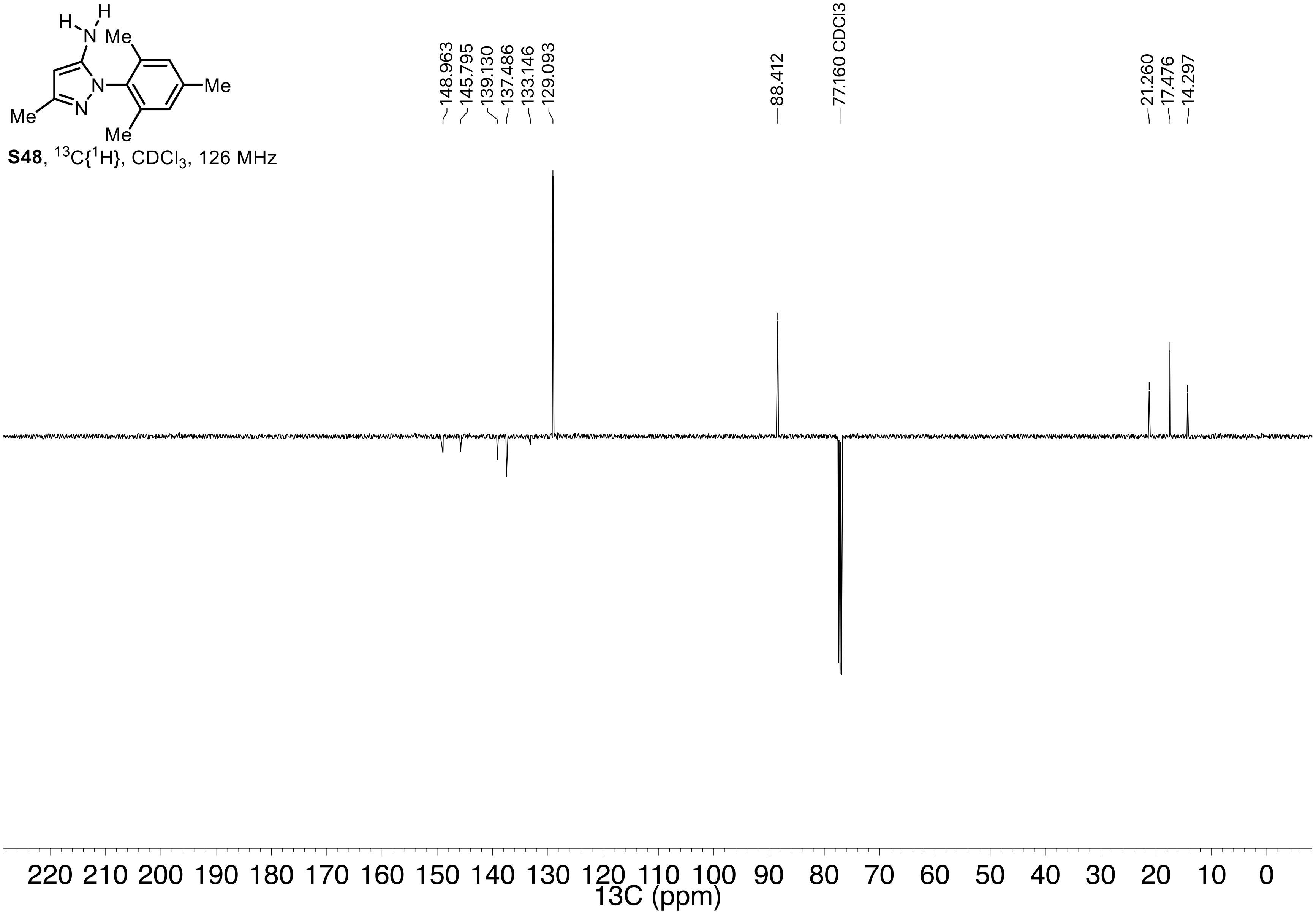

S48, $^{13}C\{^1H\}$, $CDCl_3$, 126 MHz
148.963
145.795
139.130
137.486
133.146
129.093
88.412
77.160 CDCl3
21.260
17.476
14.297
220 210 200 190 180 170 160 150 140 130 120 110 100 90 80 70 60 50 40 30 20 10 0
13C (ppm)

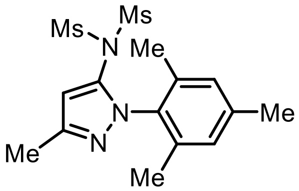

S49, ^1H, $CDCl_3$, 400 MHz

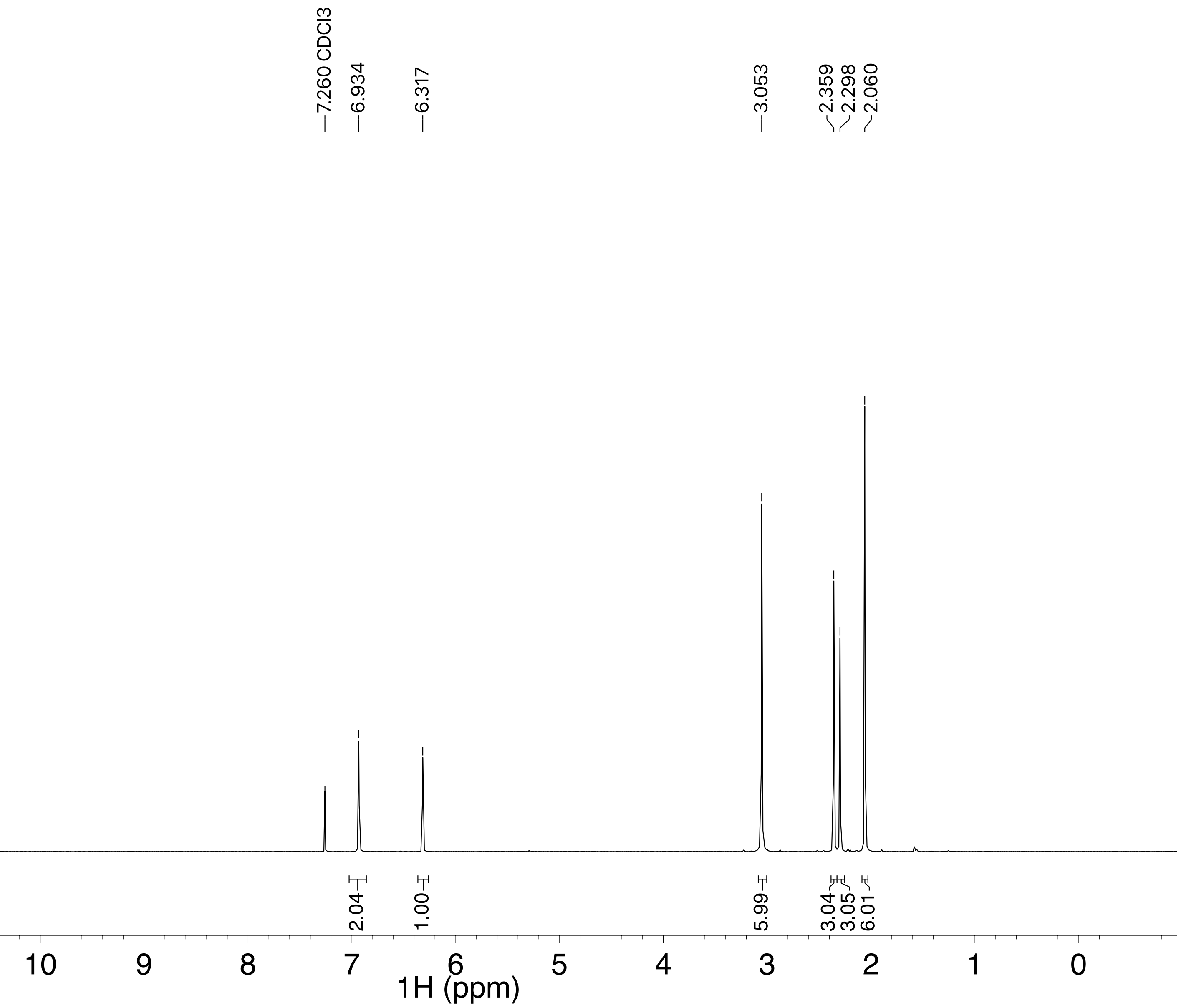

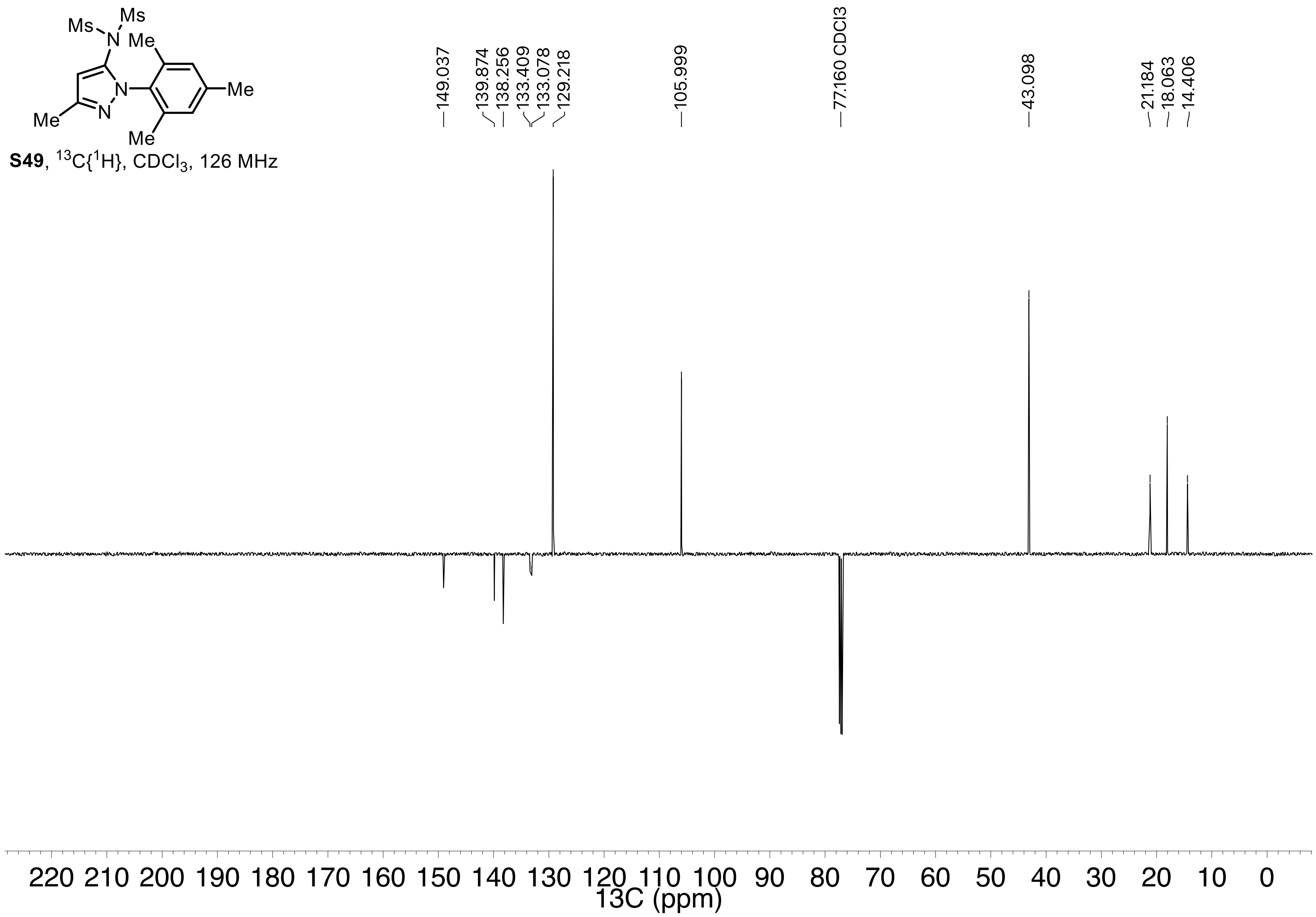

S49, $^{13}C\{^1H\}$, $CDCl_3$, 126 MHz

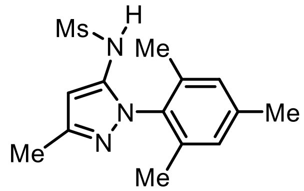

S16, ^{1}H, $CDCl_3$, 400 MHz

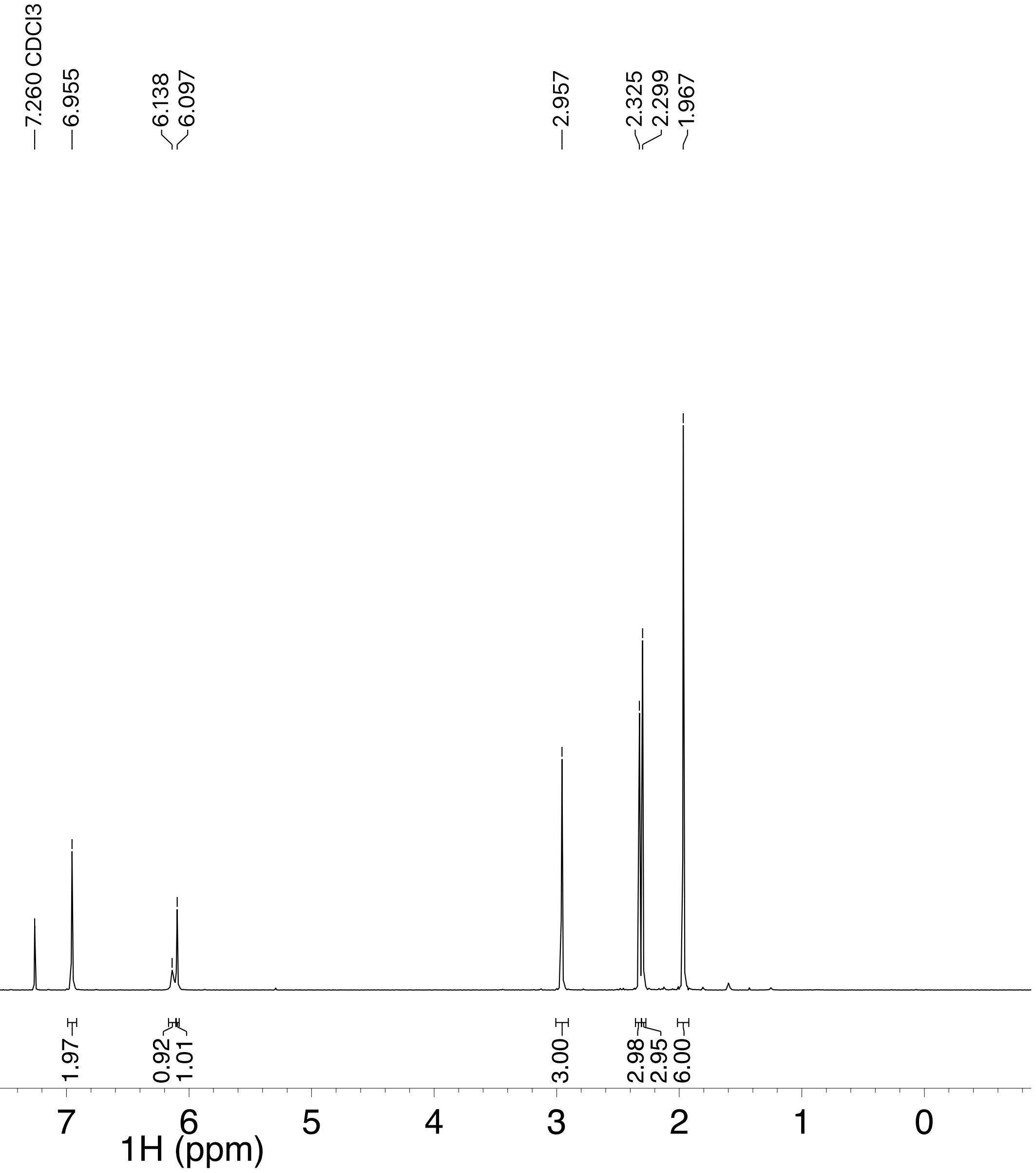

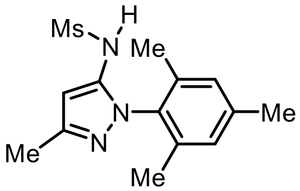

S16, $^{13}C\{^{1}H\}$, $CDCl_3$, 126 MHz

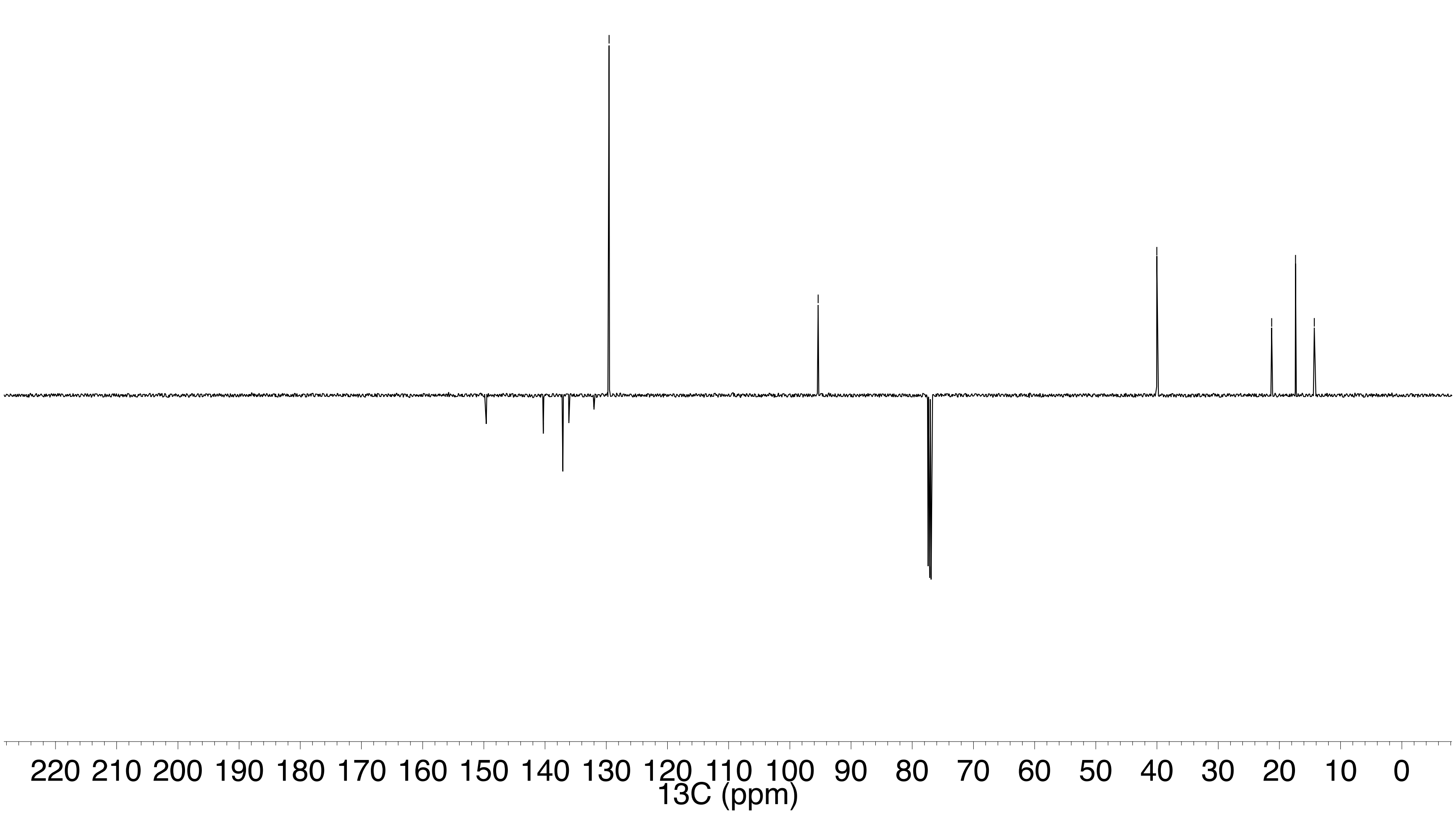

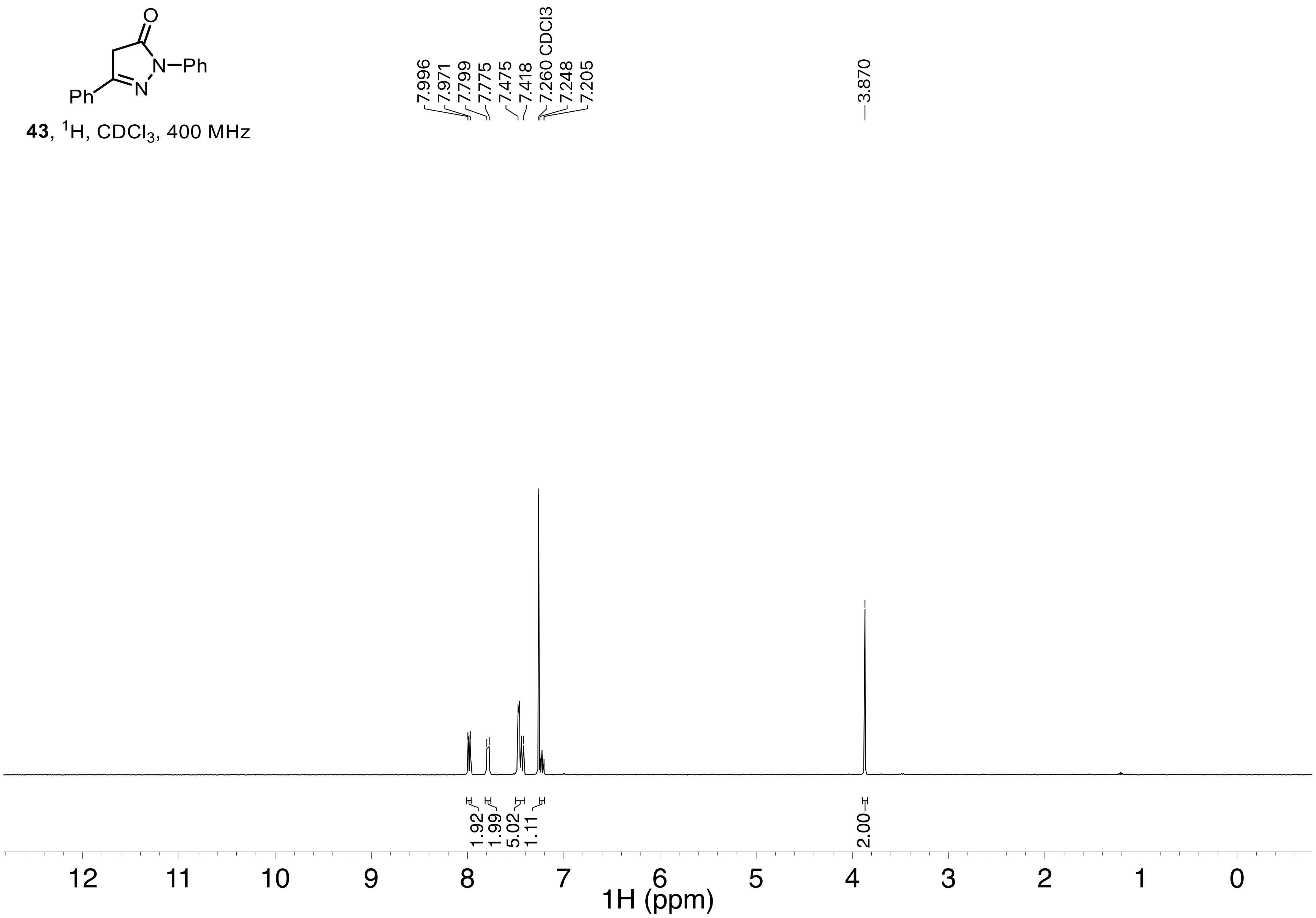

43, ^{1}H, $CDCl_3$, 400 MHz

43, $^{13}C\{^{1}H\}$, $CDCl_3$, 101 MHz

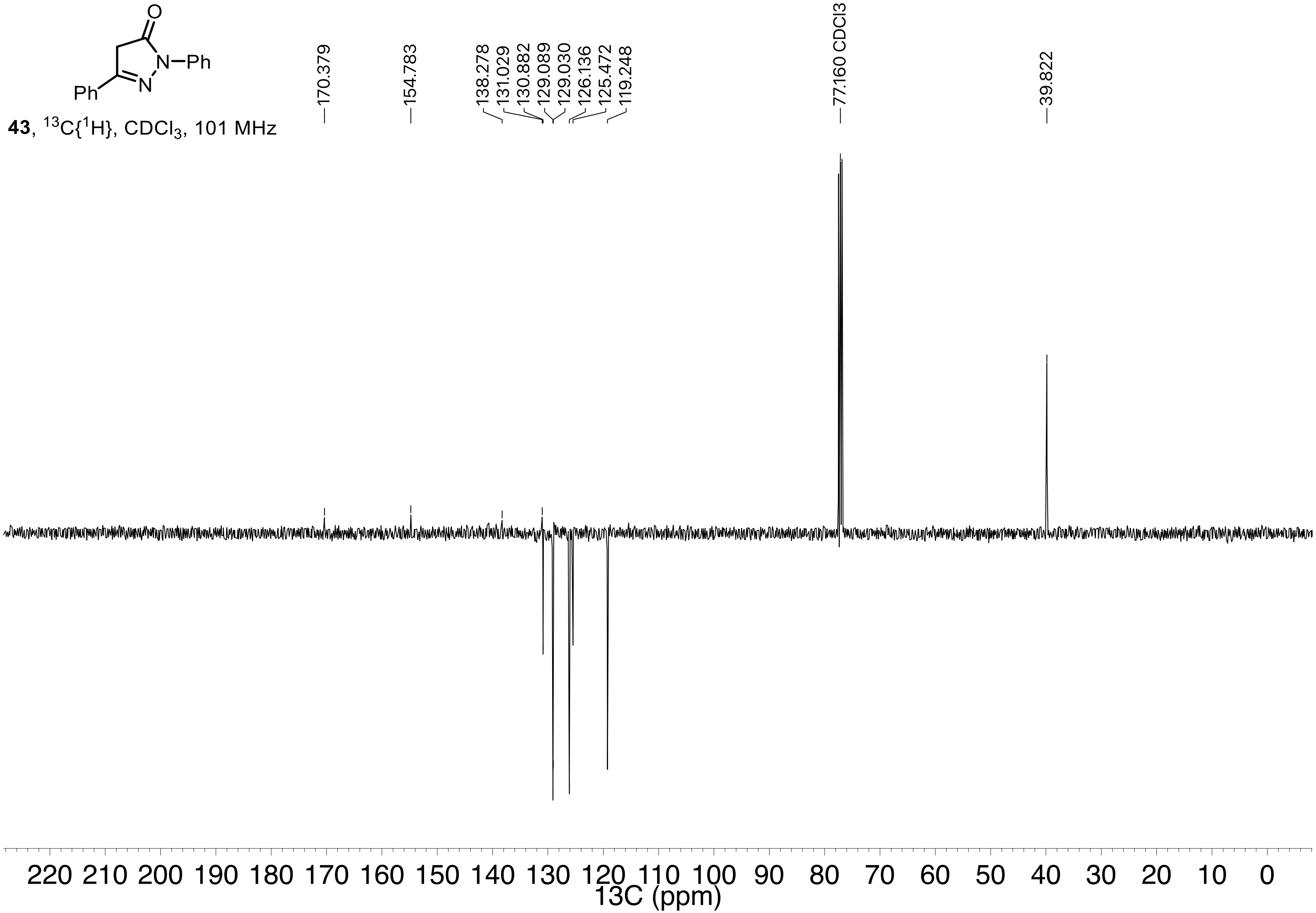

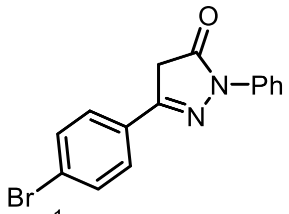

S50, ^{1}H, $CDCl_3$, 300 MHz

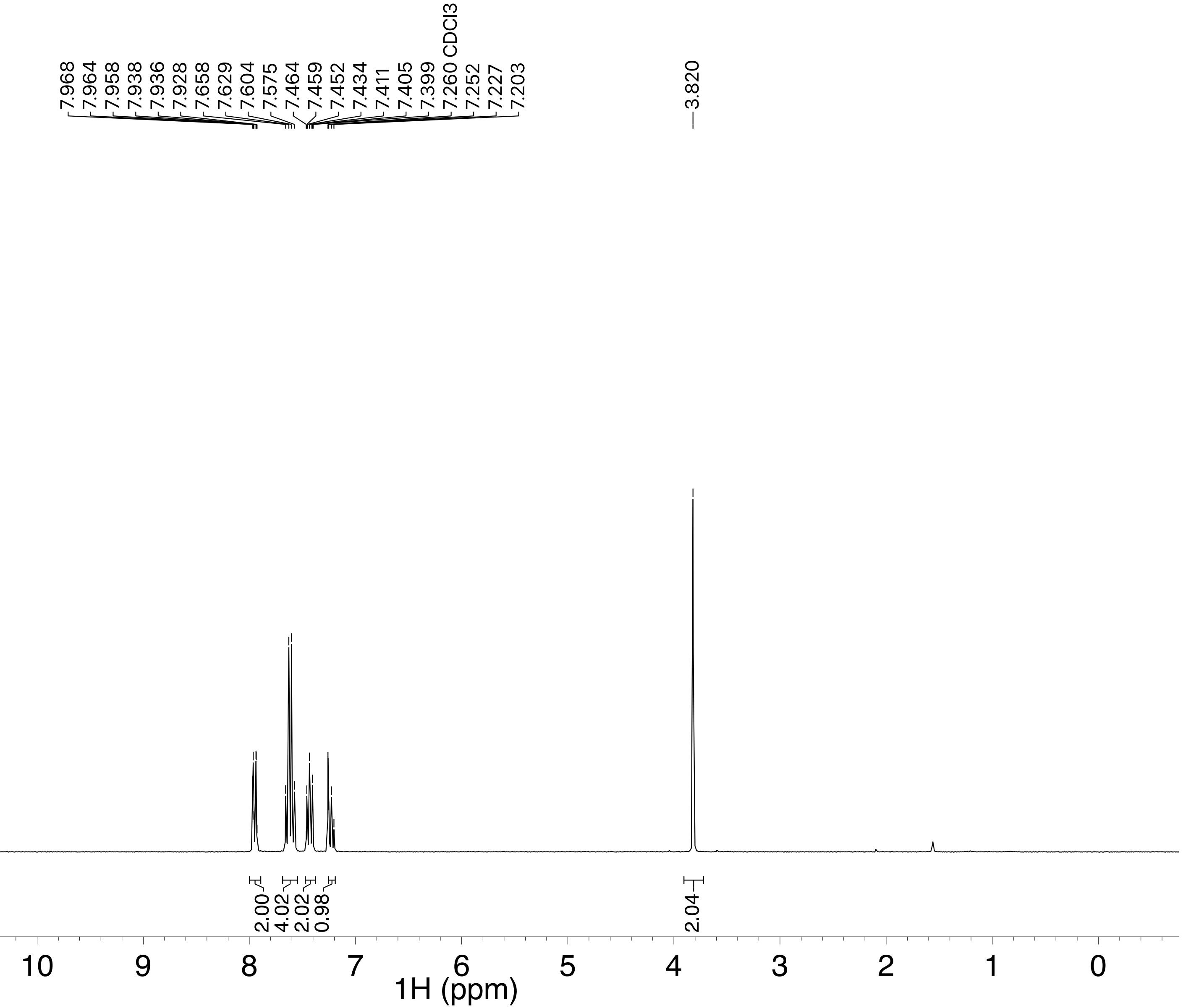

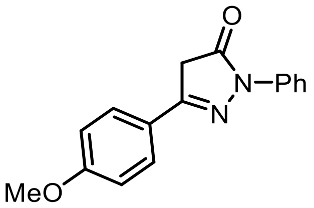

S51, ^{1}H, $CDCl_3$, 300 MHz

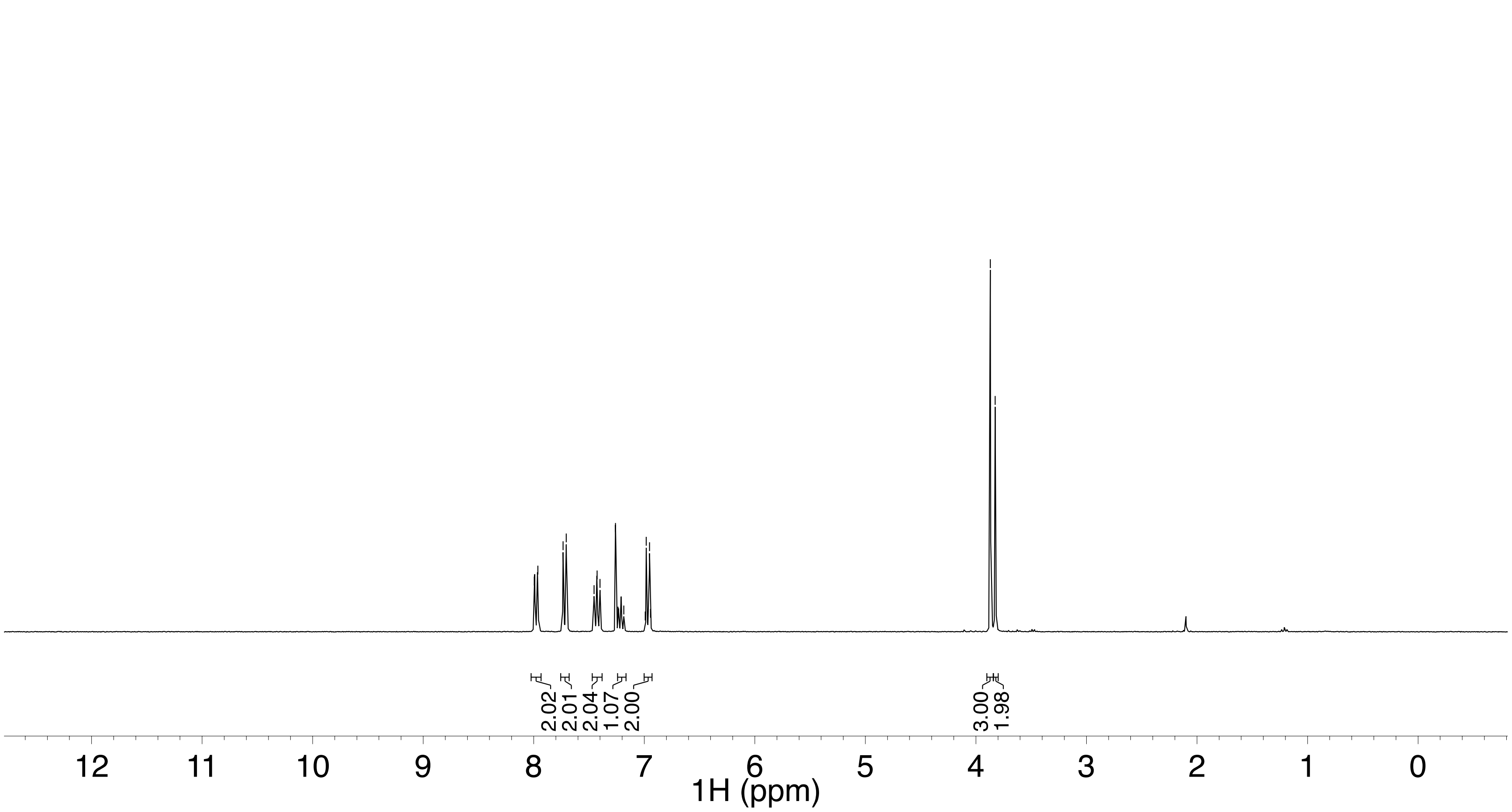

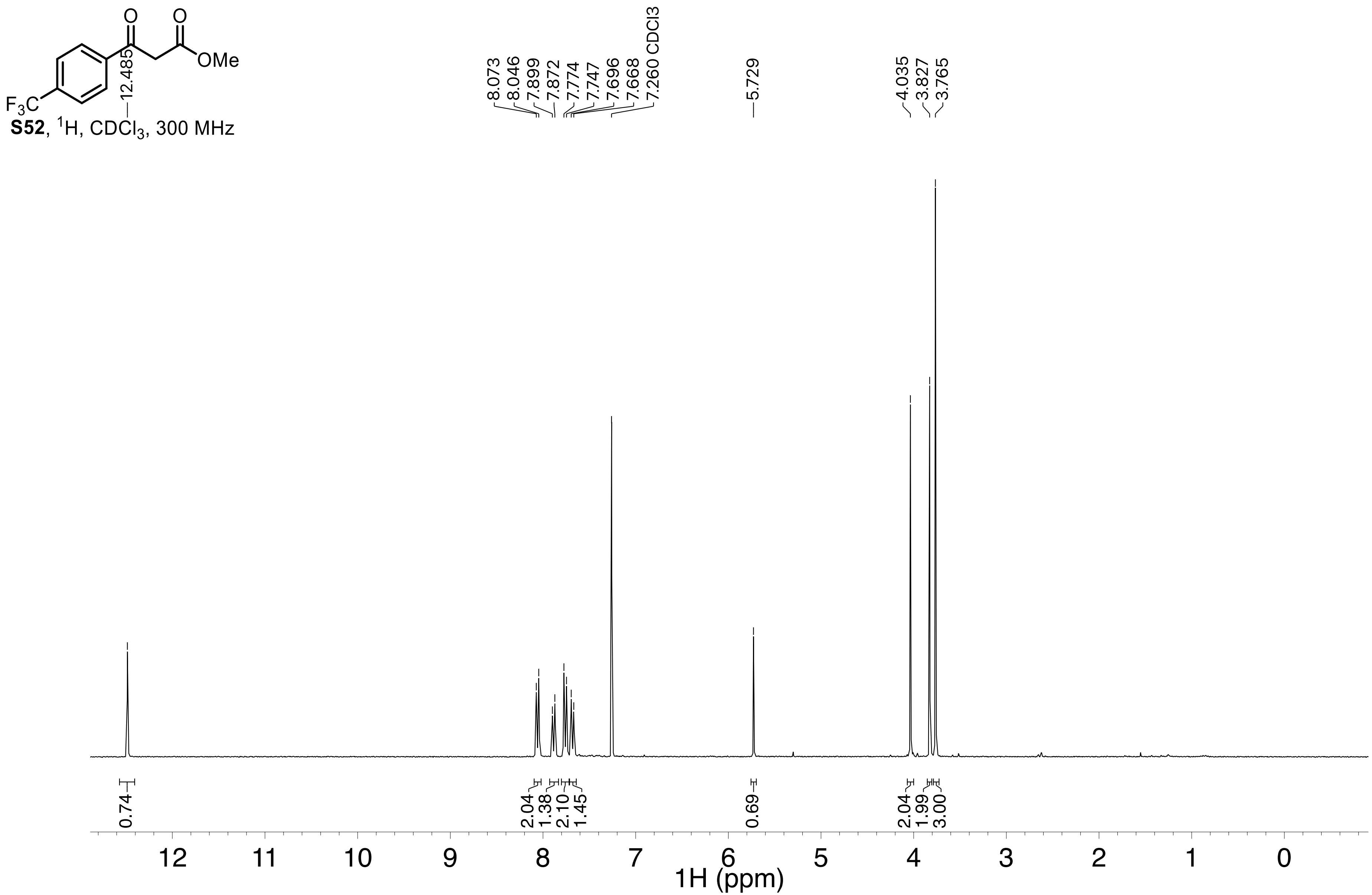

S52, ^{1}H, $CDCl_3$, 300 MHz

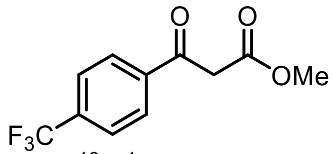

S52, $^{19}F\{^1H\}$, $CDCl_3$, 376 MHz

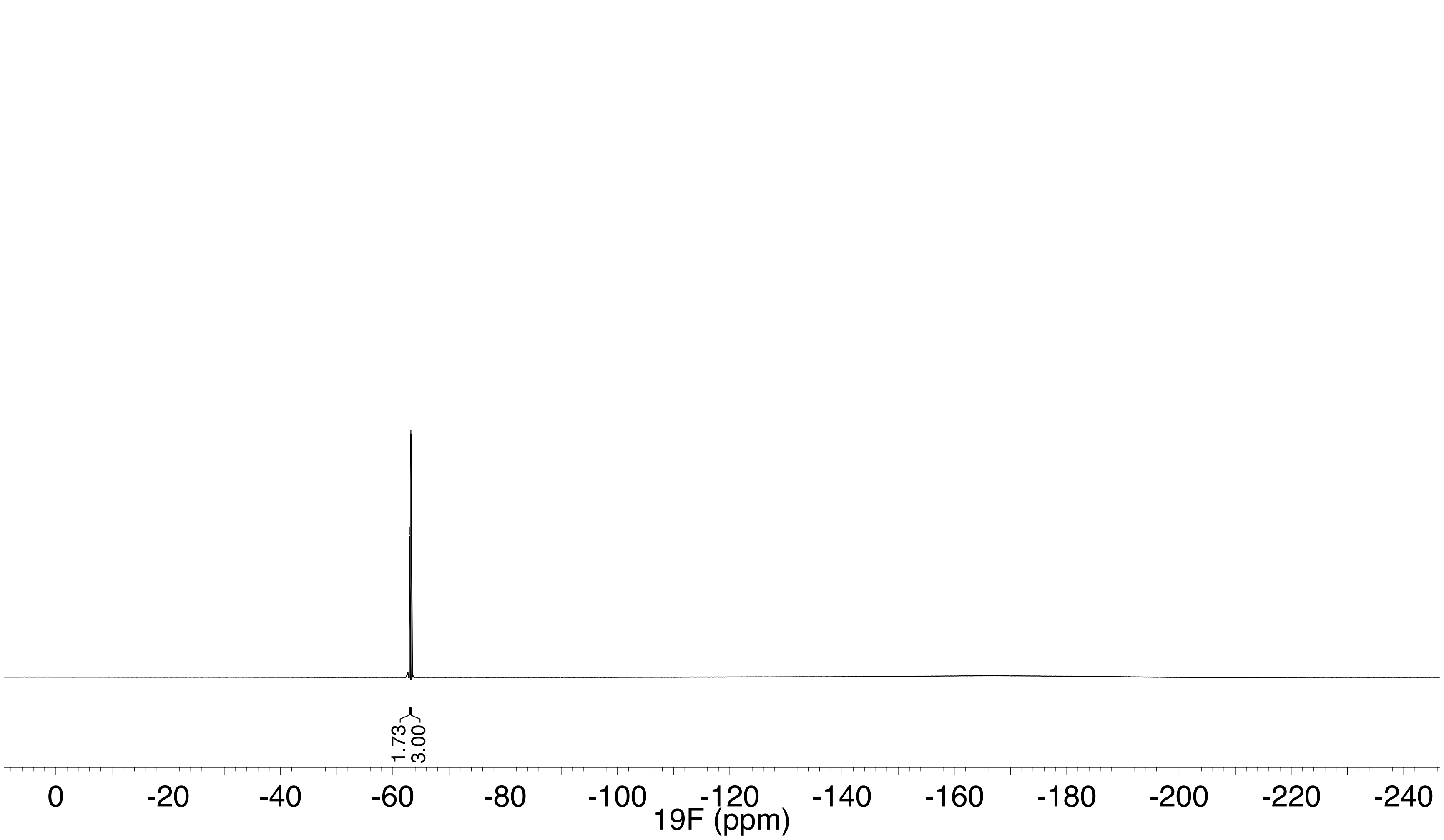

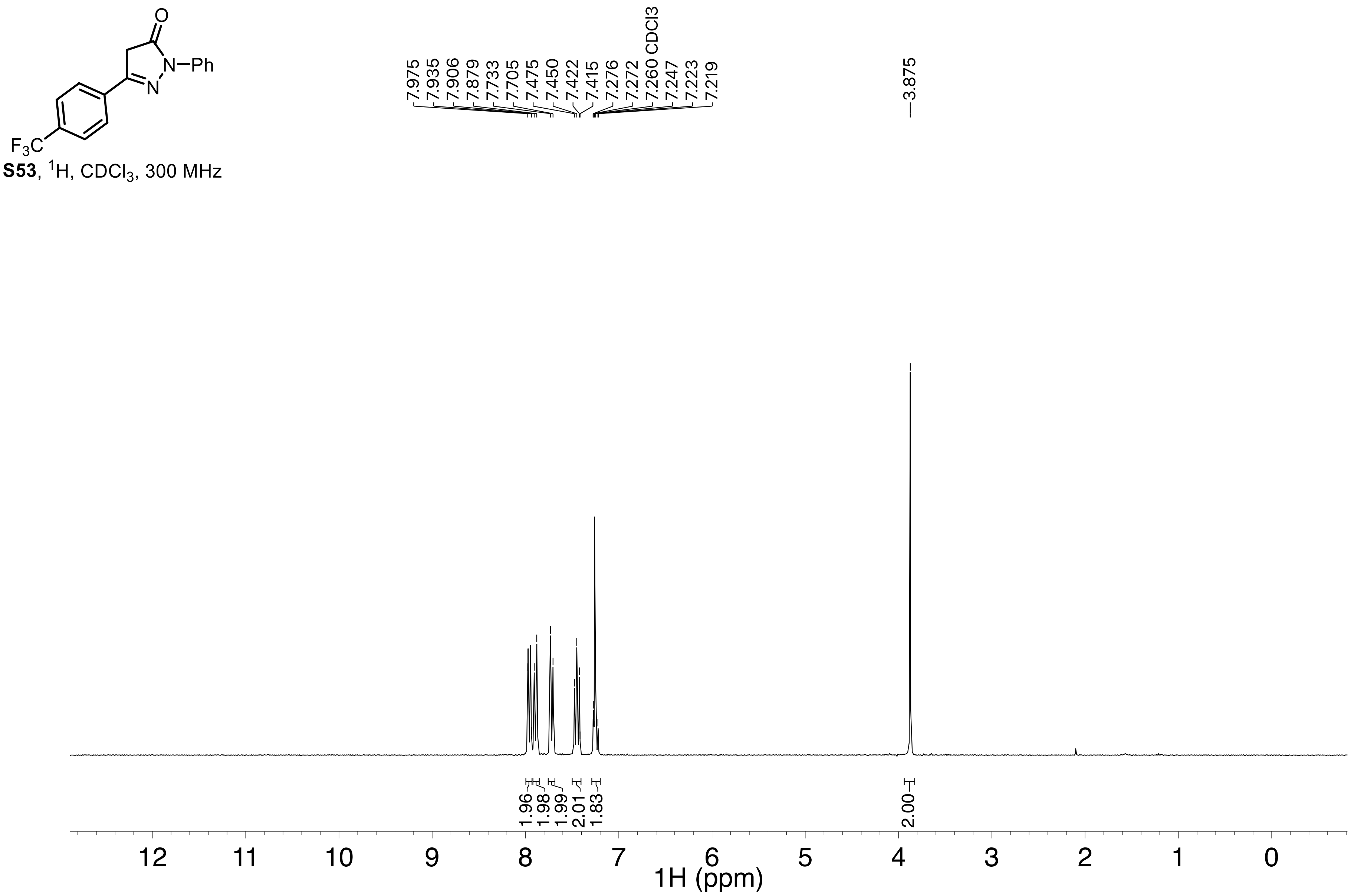

S53, ^{1}H, $CDCl_3$, 300 MHz

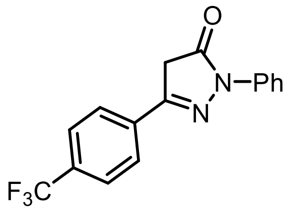

S53, $^{19}F\{^{1}H\}$, $CDCl_3$, 471 MHz

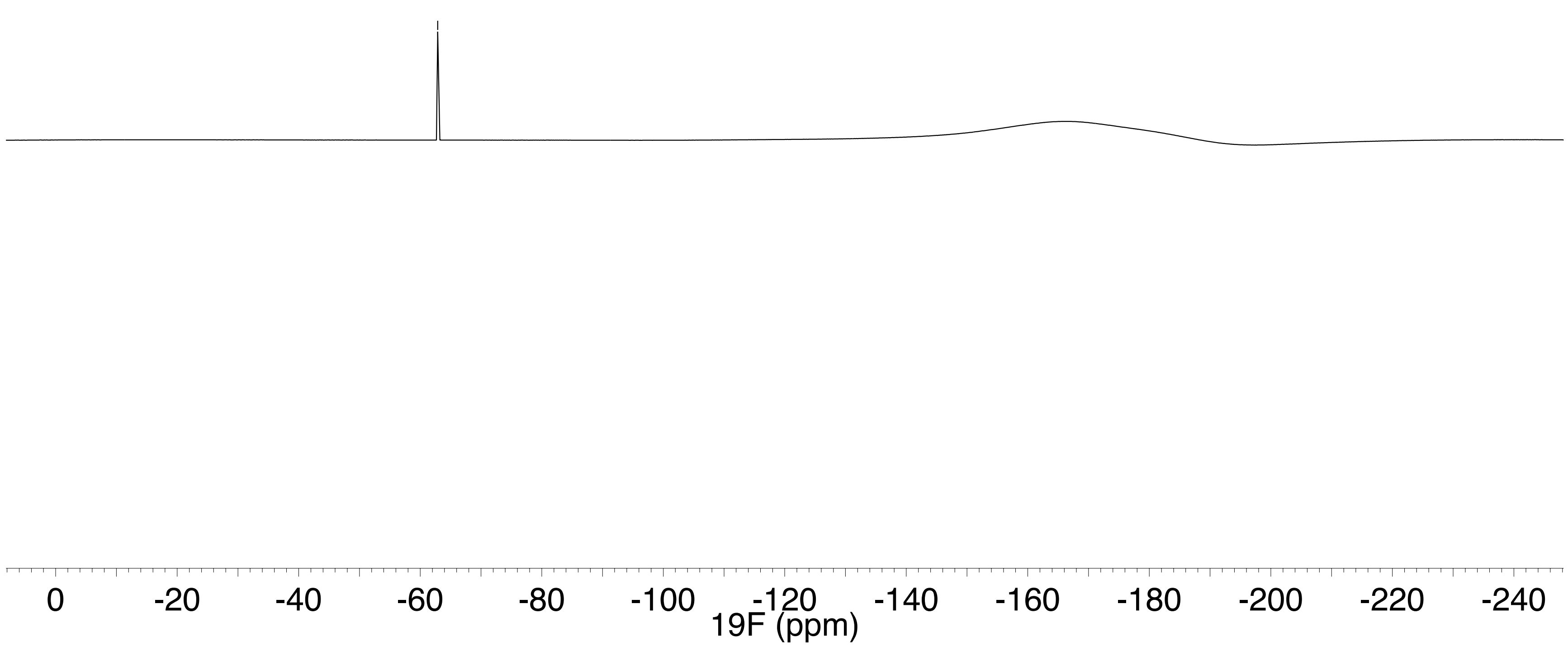

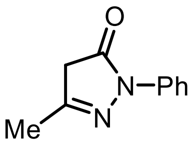

S8, ^1H, $CDCl_3$, 300 MHz

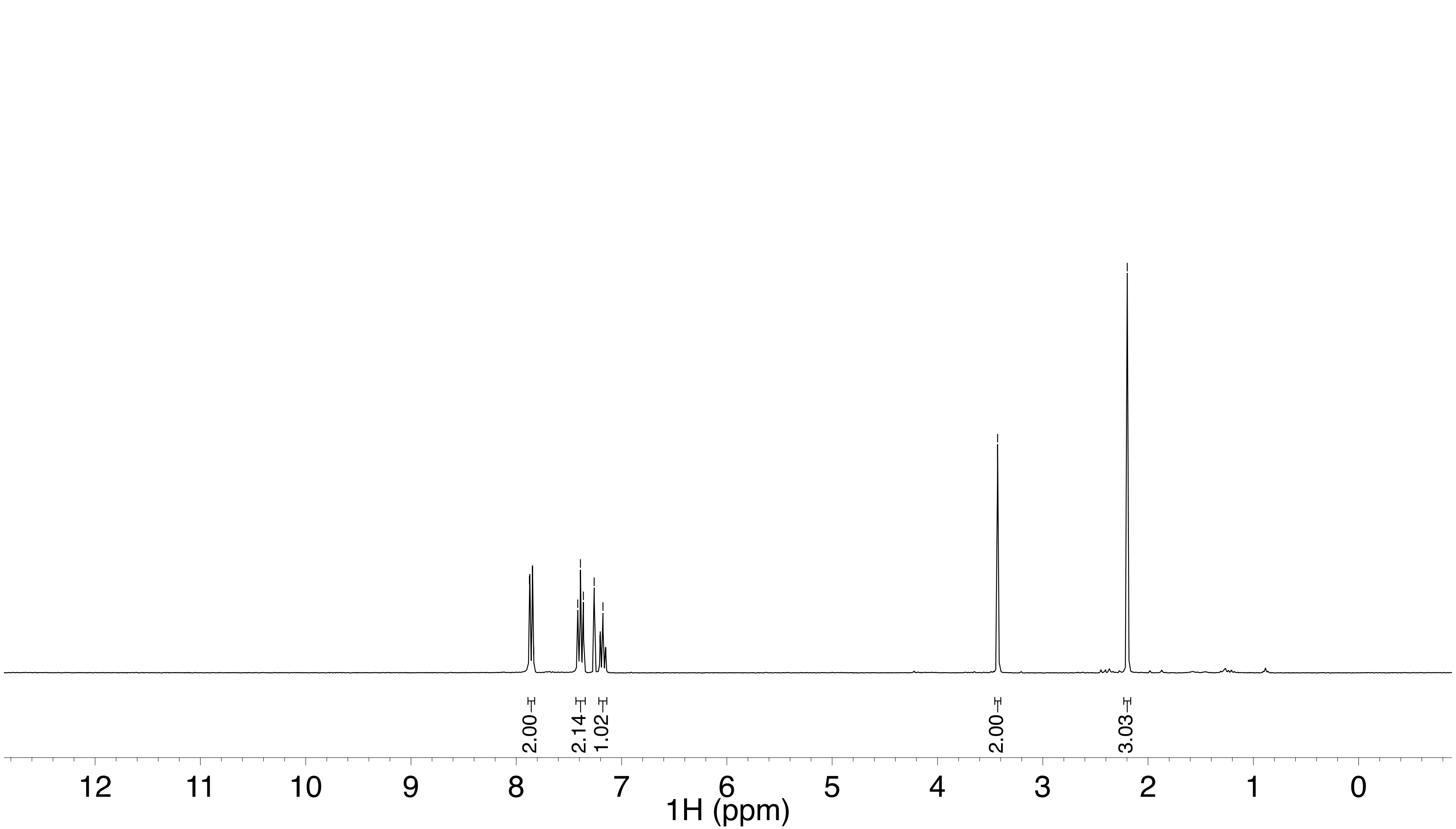

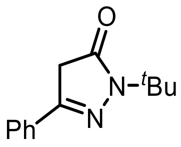

S54, ^{1}H, $CDCl_3$, 300 MHz

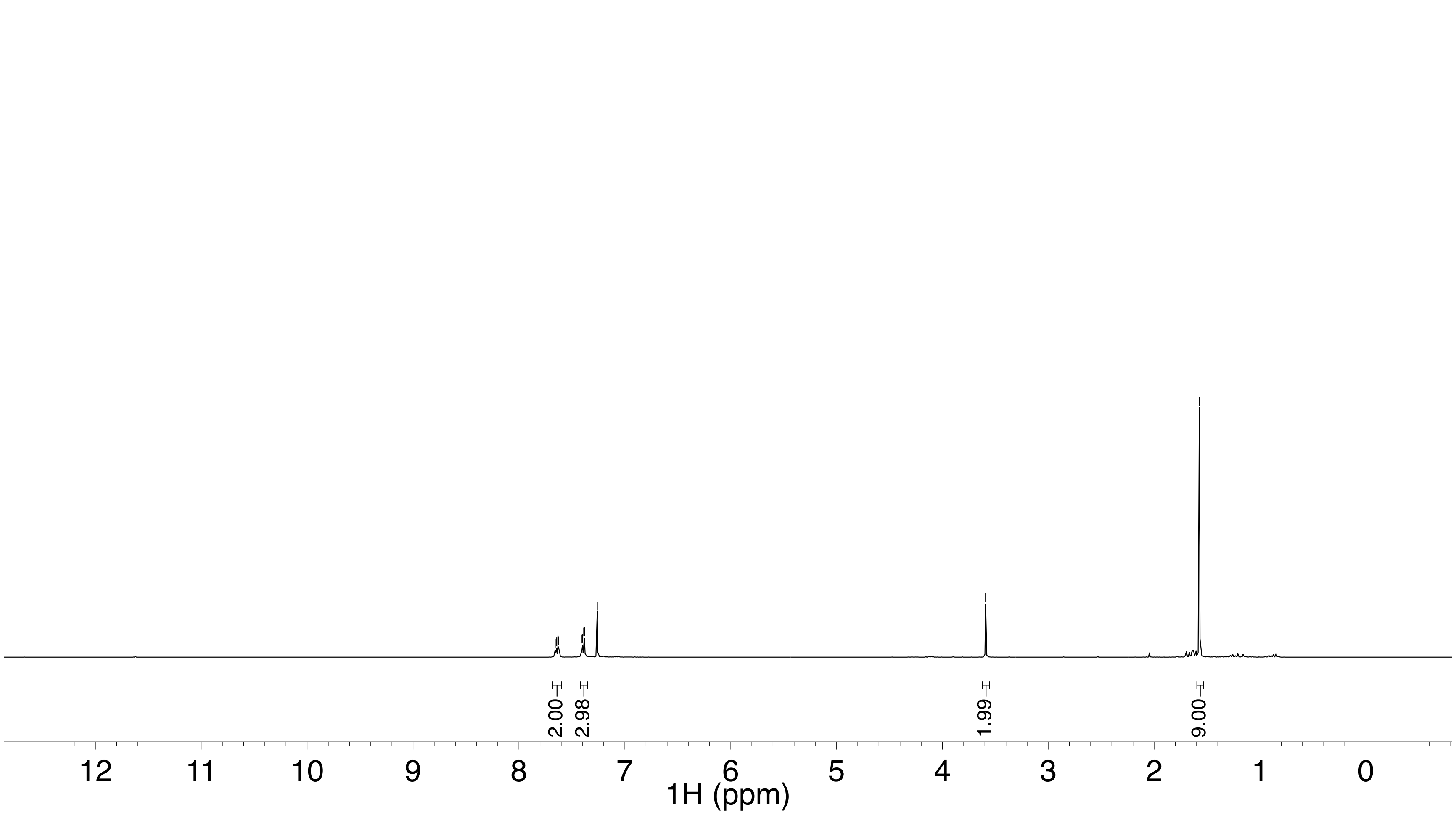

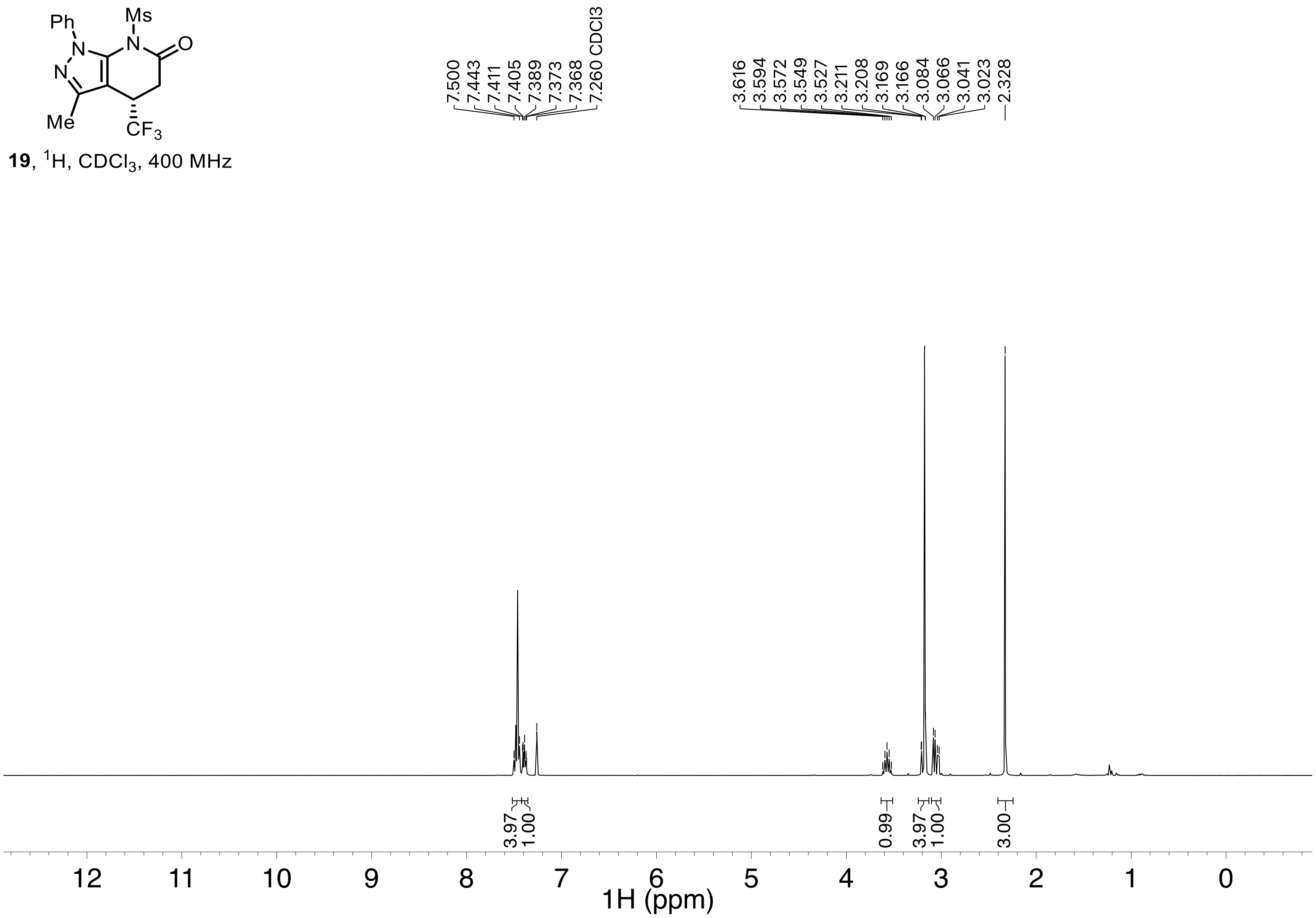
Ph
Ms
N
N
N
O
Me
CF3
19, 1H, CDCl3, 400 MHz
7.500
7.443
7.411
7.405
7.389
7.373
7.368
7.260 CDCl3
3.616
3.594
3.572
3.549
3.527
3.211
3.208
3.169
3.166
3.084
3.066
3.041
3.023
2.328
3.97
1.00
0.99
3.97
1.00
3.00
12
11
10
9
8
7
6
5
4
3
2
1
0
1H (ppm)

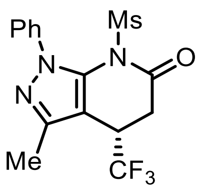

19, $^{13}C\{^1H\}$, $CDCl_3$, 126 MHz

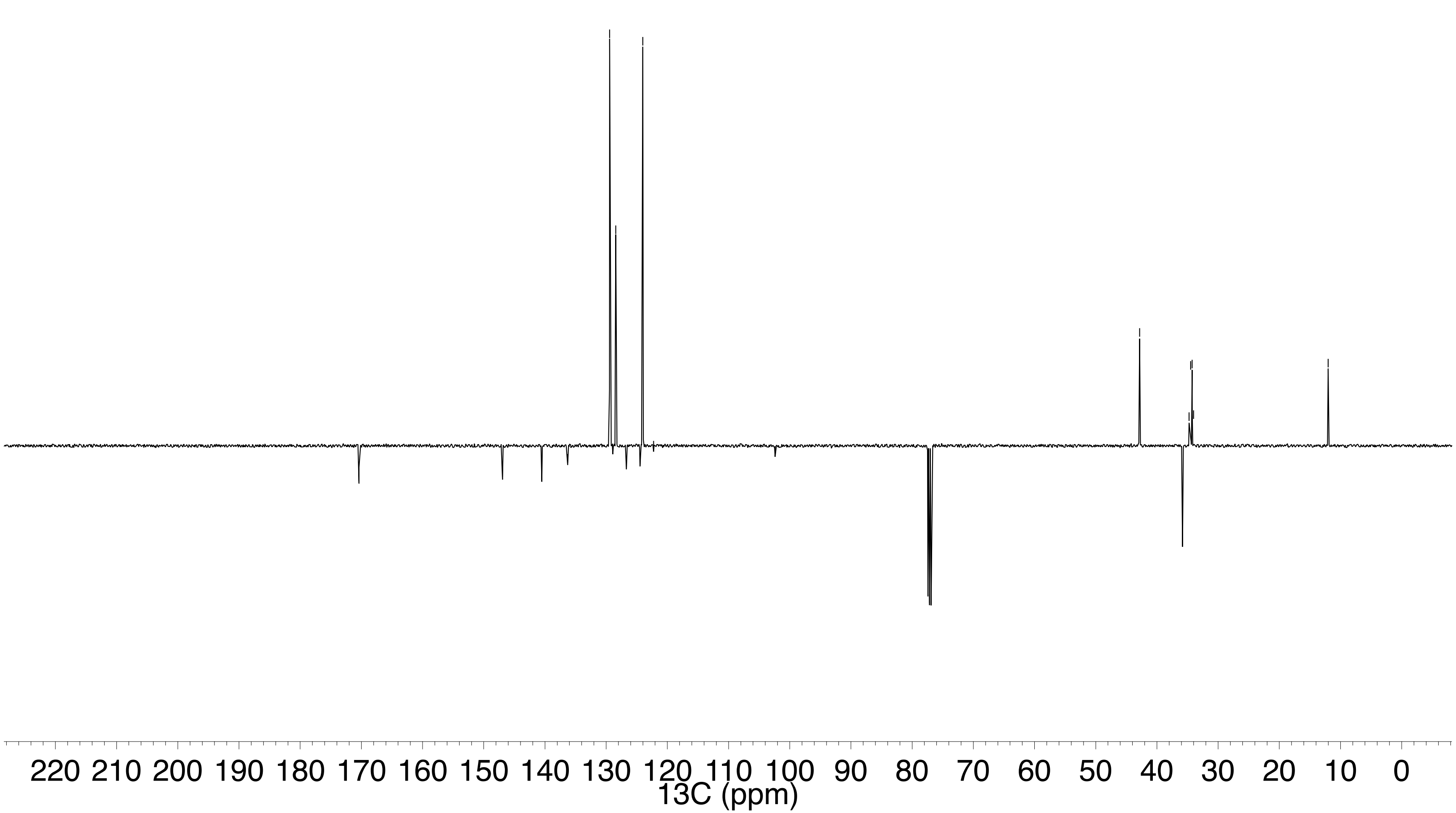

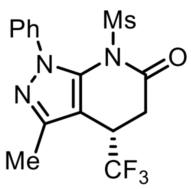

19, $^{19}F\{^{1}H\}$, $CDCl_3$, 377 MHz

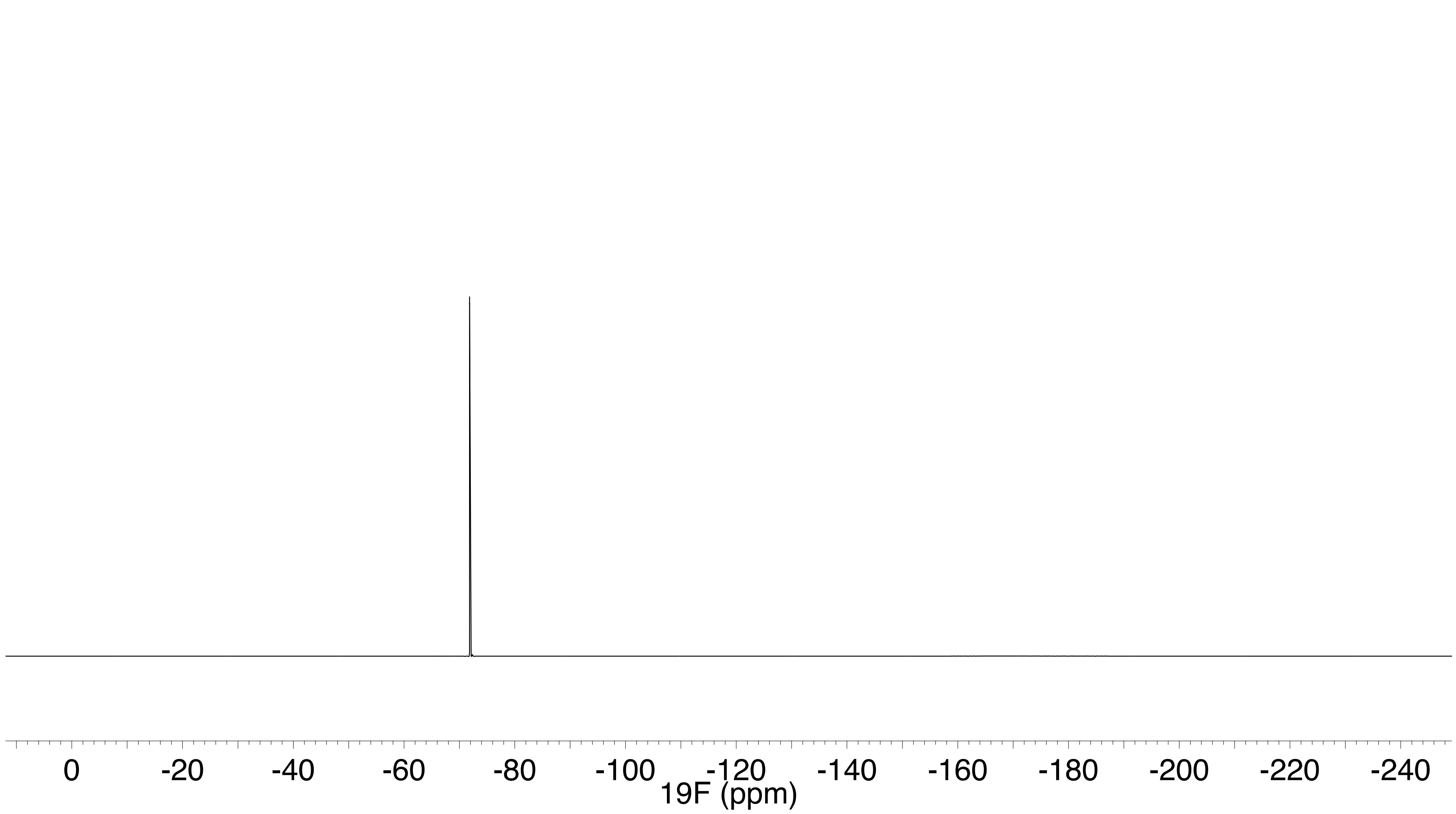

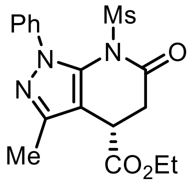

28, ^{1}H, $CDCl_3$, 500 MHz

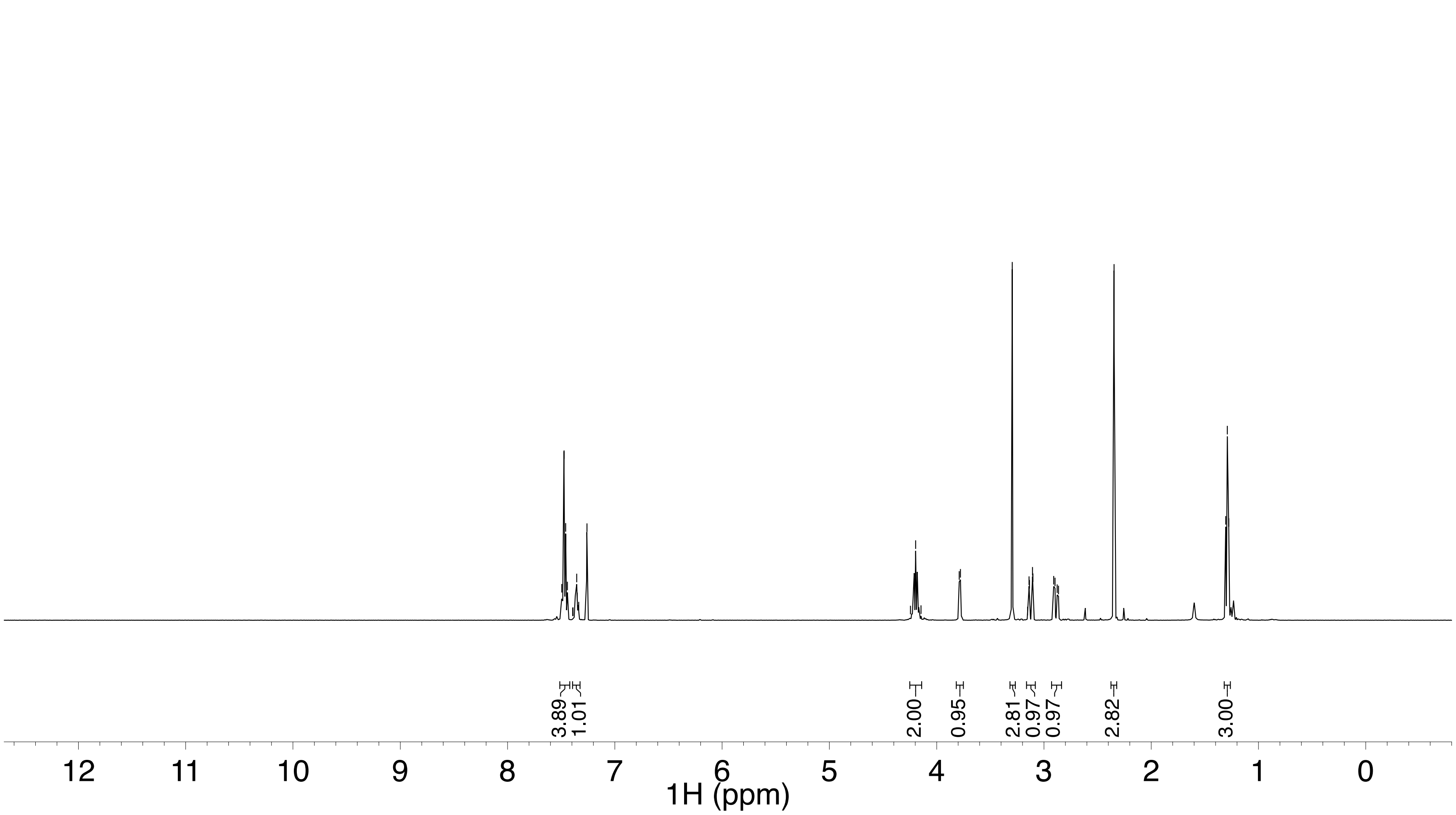

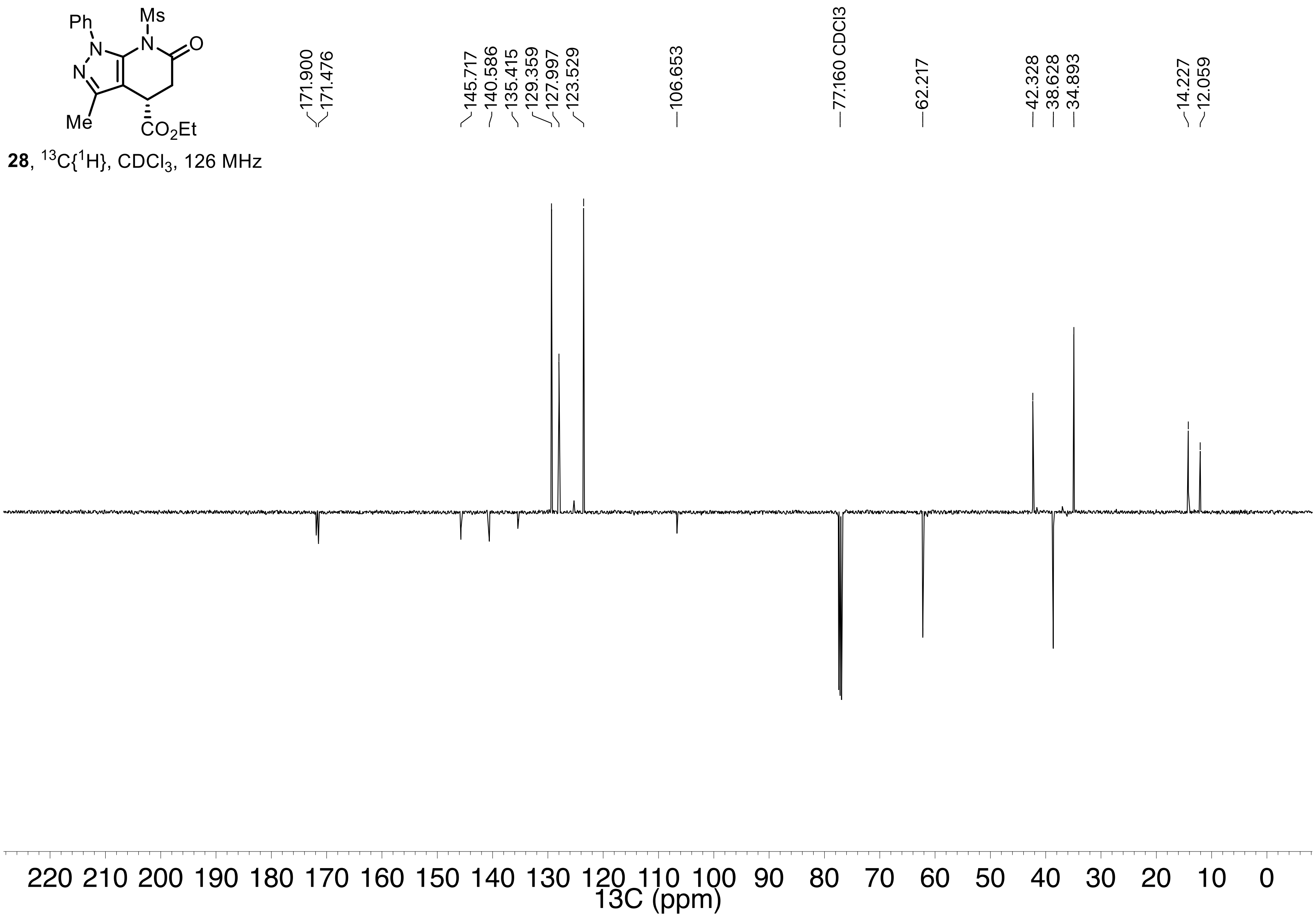

28, $^{13}C\{^1H\}$, $CDCl_3$, 126 MHz

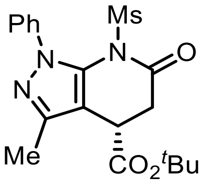

29, ^{1}H, $CDCl_3$, 400 MHz

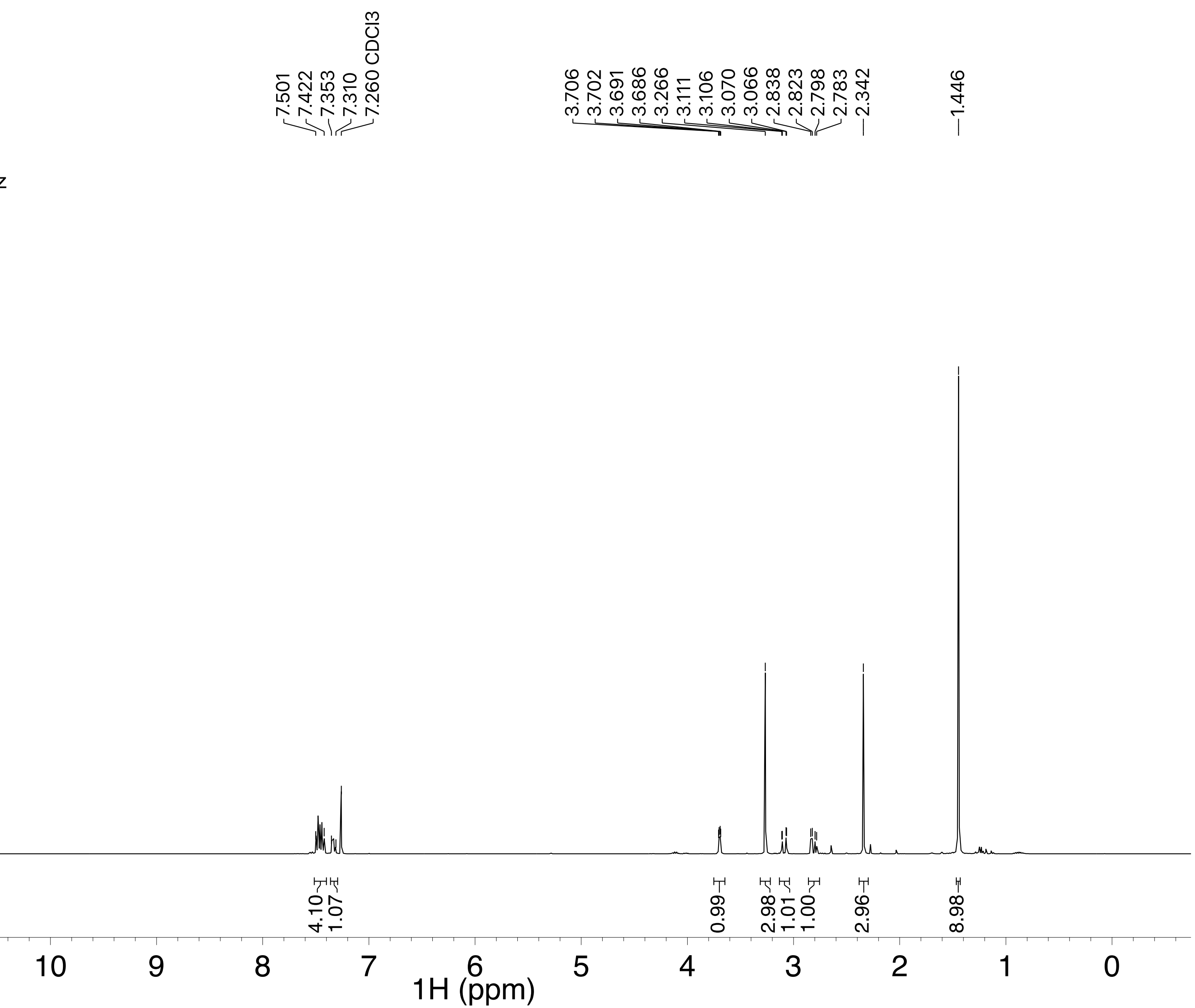

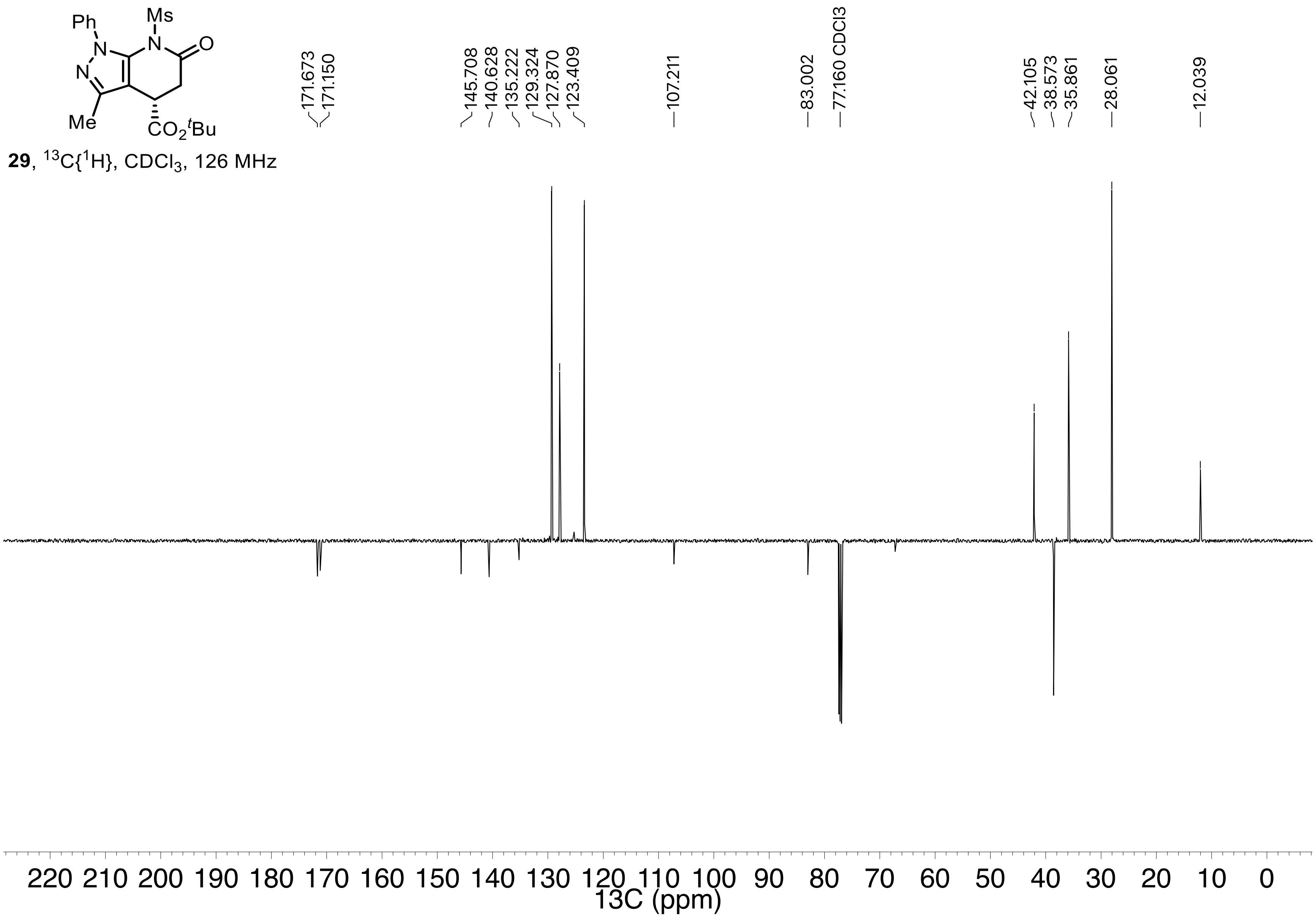
Ph
Ms
N
N
N
O
Me
CO2tBu
29, 13C{1H}, CDCl3, 126 MHz
171.673
171.150
145.708
140.628
135.222
129.324
127.870
123.409
107.211
83.002
77.160 CDCl3
42.105
38.573
35.861
28.061
12.039
220 210 200 190 180 170 160 150 140 130 120 110 100 90 80 70 60 50 40 30 20 10 0
13C (ppm)

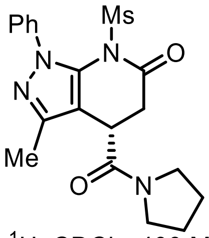

30, ^{1}H, $CDCl_3$, 400 MHz

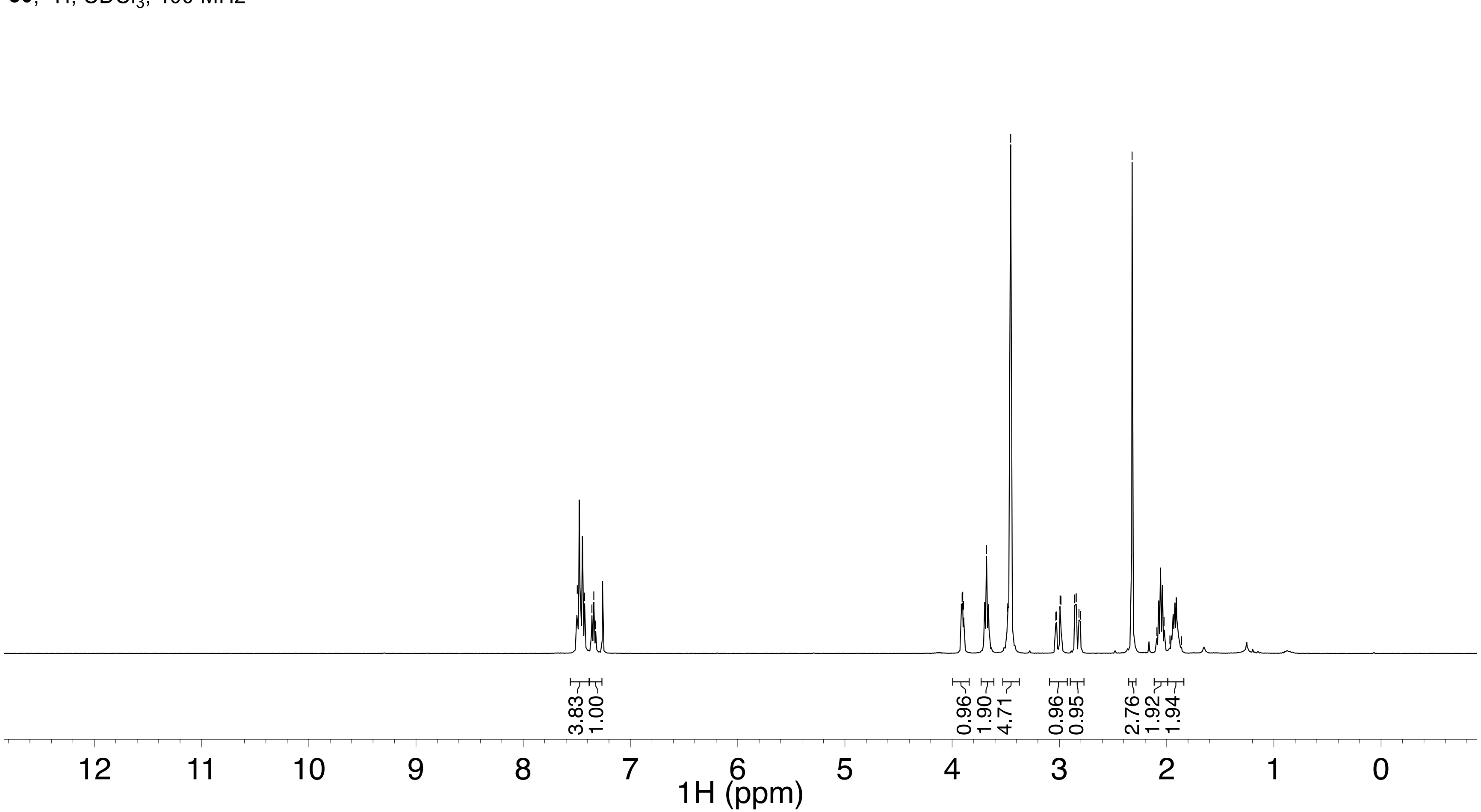

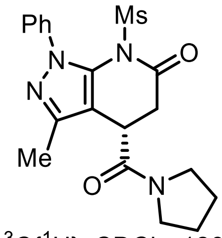

30, $^{13}C\{^{1}H\}$, $CDCl_3$, 126 MHz

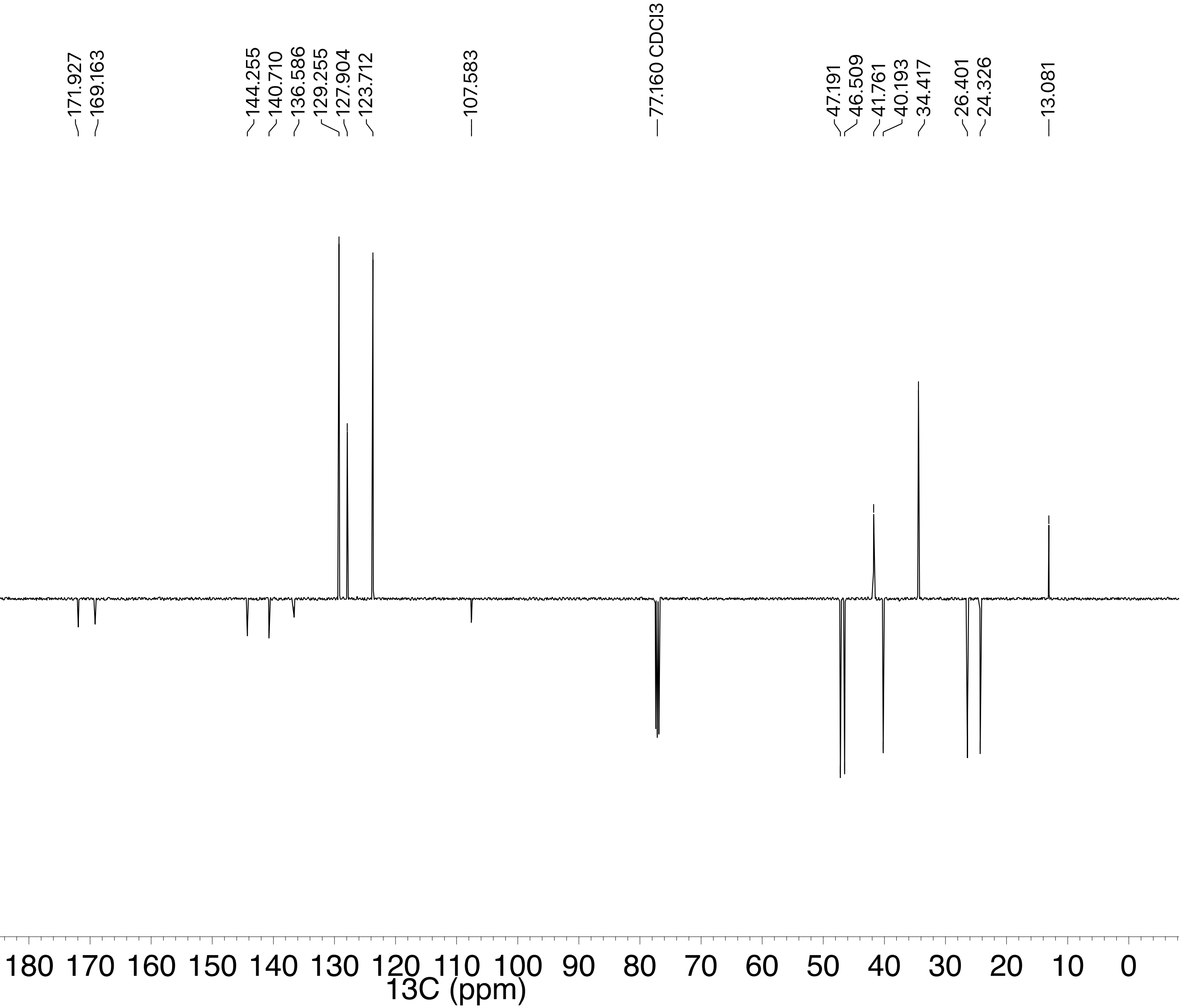

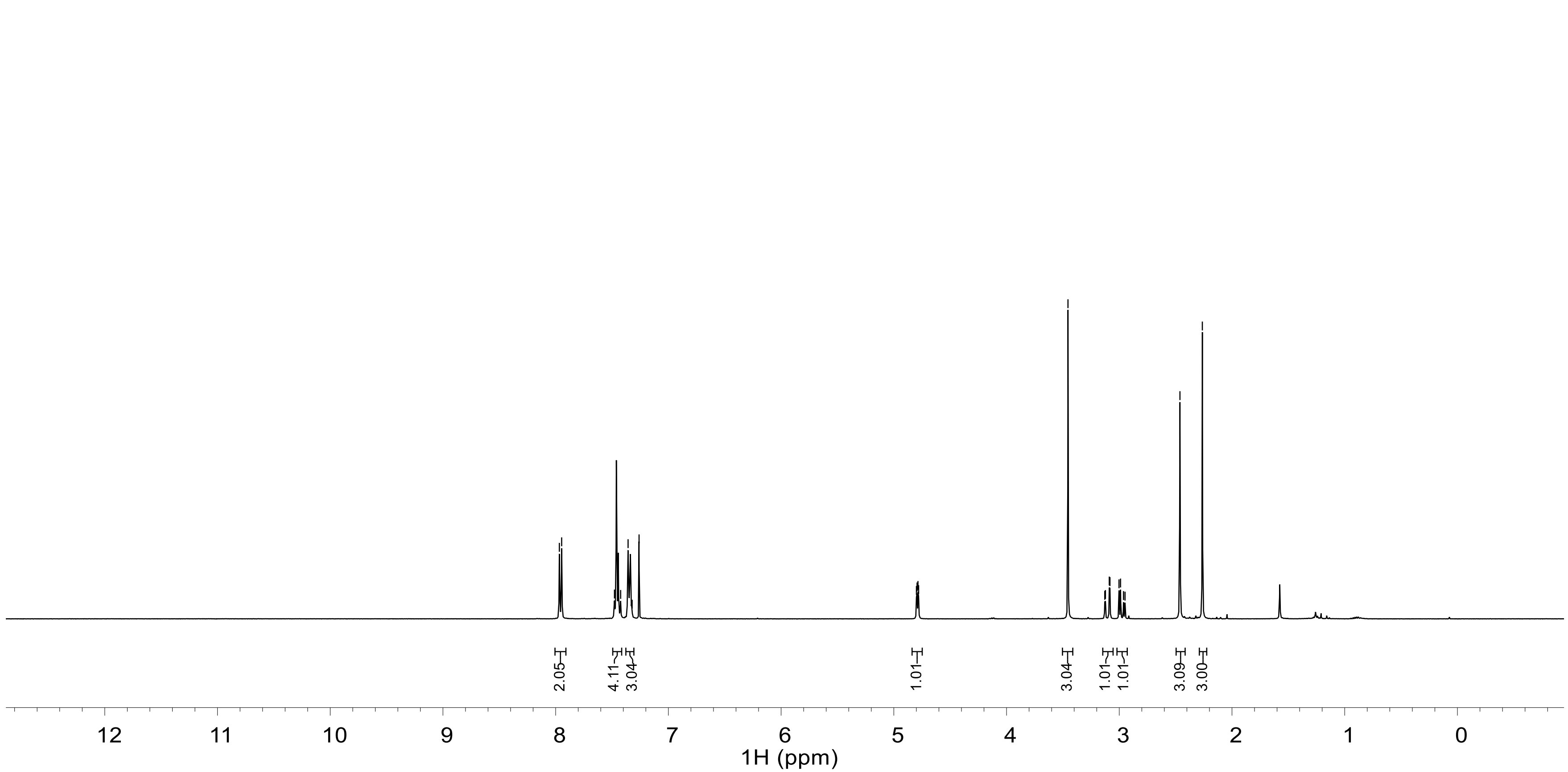

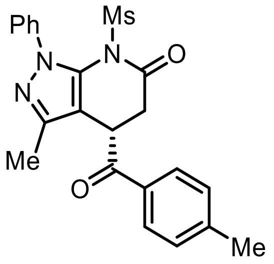

31, ^{1}H, $CDCl_3$, 400 MHz

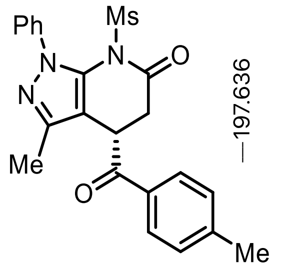

31, ^{1}H, $CDCl_3$, 126 MHz

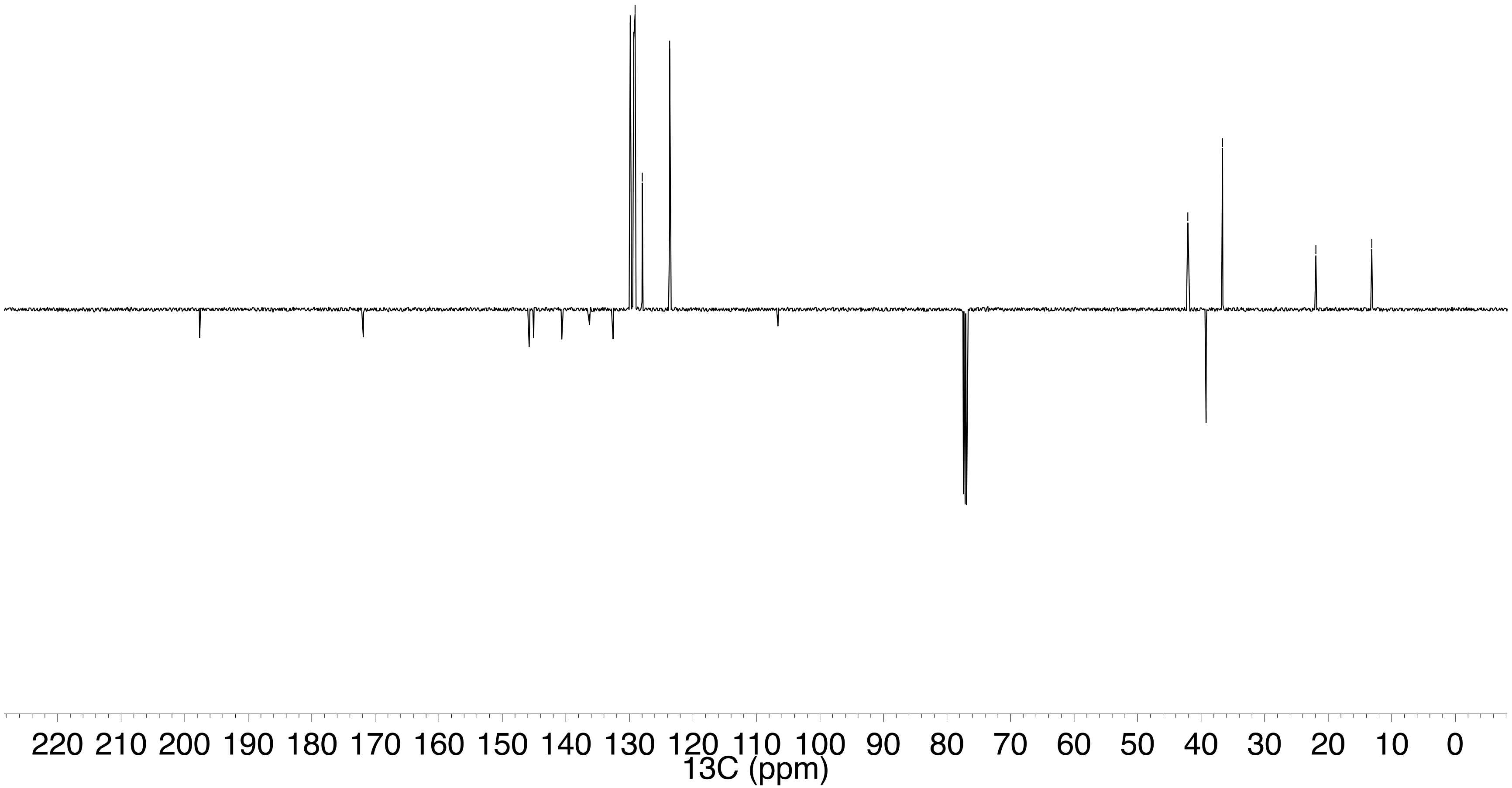

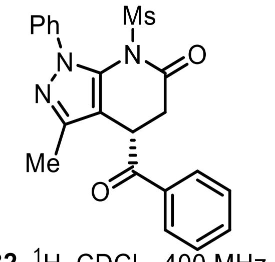

32, ^{1}H, $CDCl_3$, 400 MHz

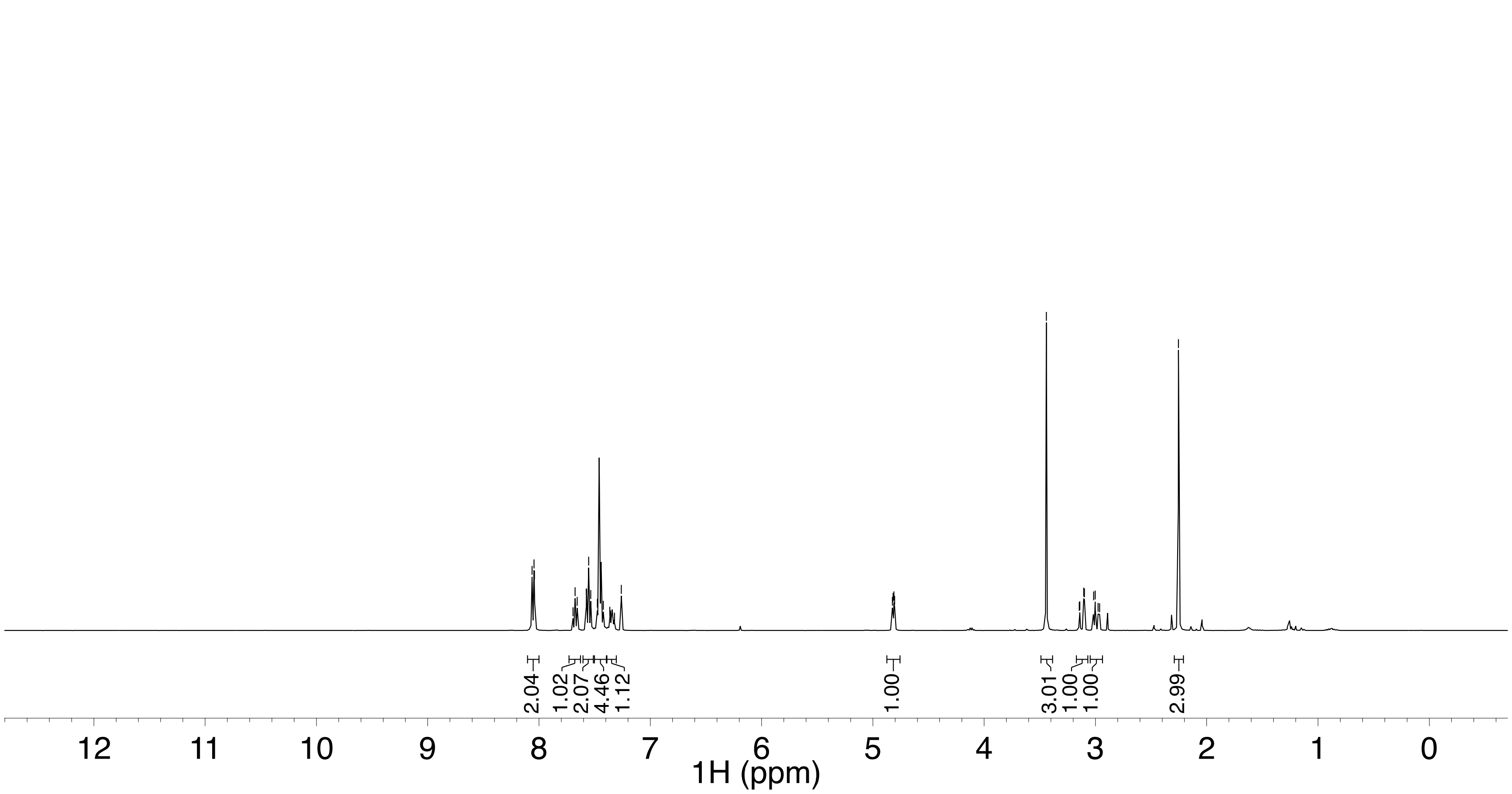

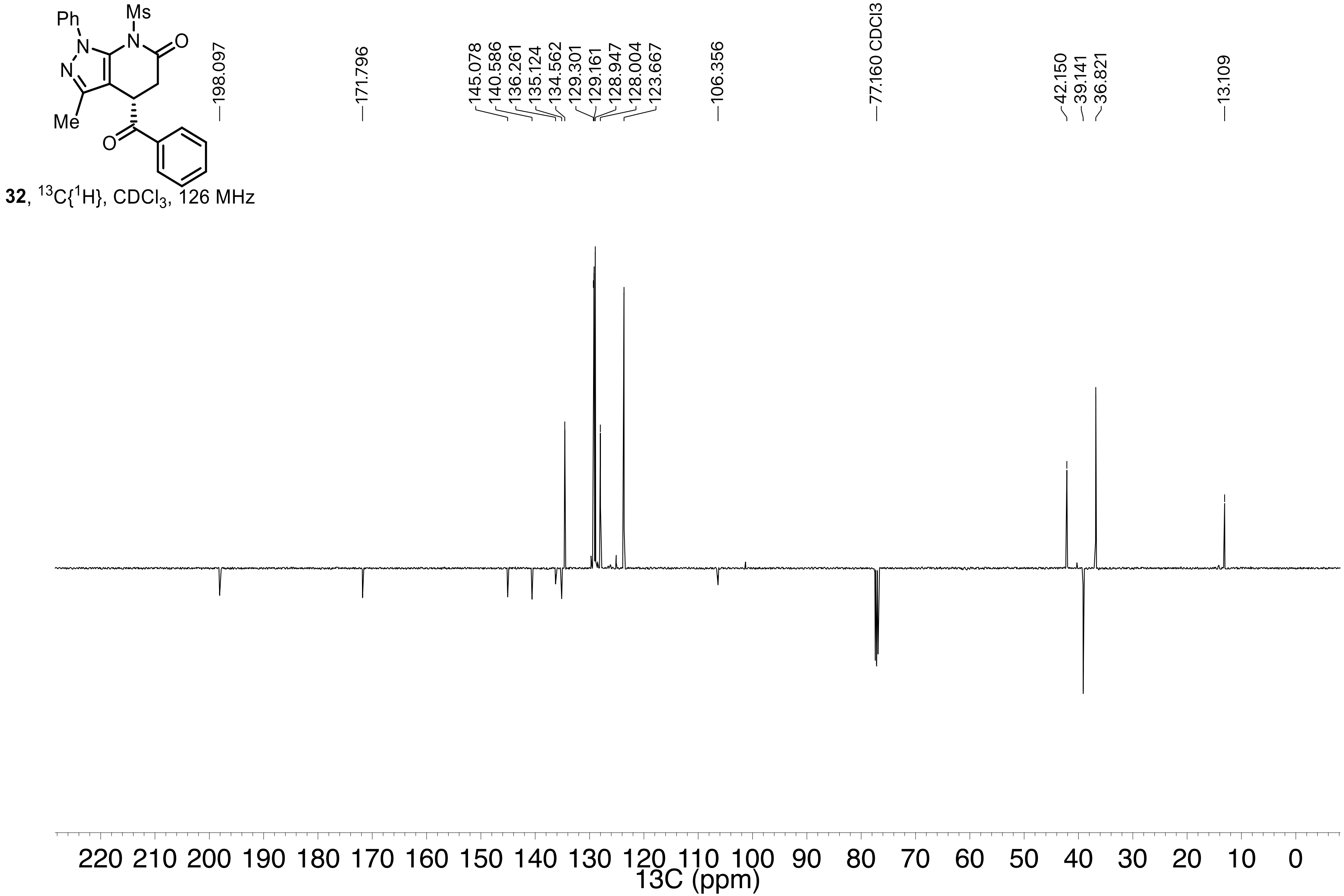

Ph
Ms
N
N
O
N
Me
O
32, $^{13}C\{^1H\}$, $CDCl_3$, 126 MHz
198.097
171.796
145.078
140.586
136.261
135.124
134.562
129.301
129.161
128.947
128.004
123.667
106.356
77.160 CDCl3
42.150
39.141
36.821
13.109
220 210 200 190 180 170 160 150 140 130 120 110 100 90 80 70 60 50 40 30 20 10 0
13C (ppm)

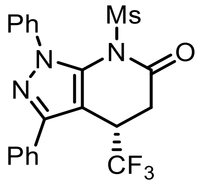

33, ^{1}H, $CDCl_3$, 500 MHz

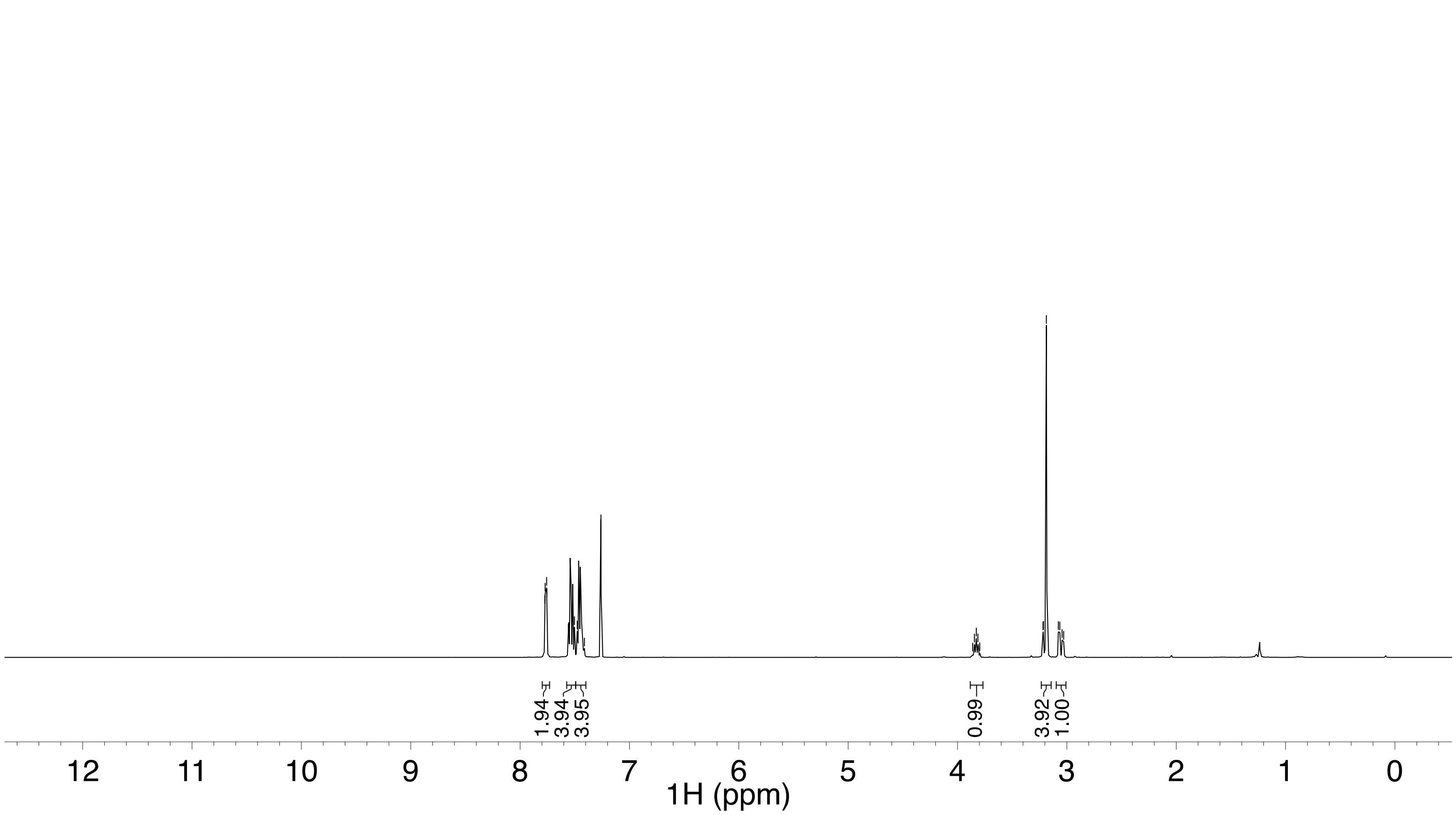

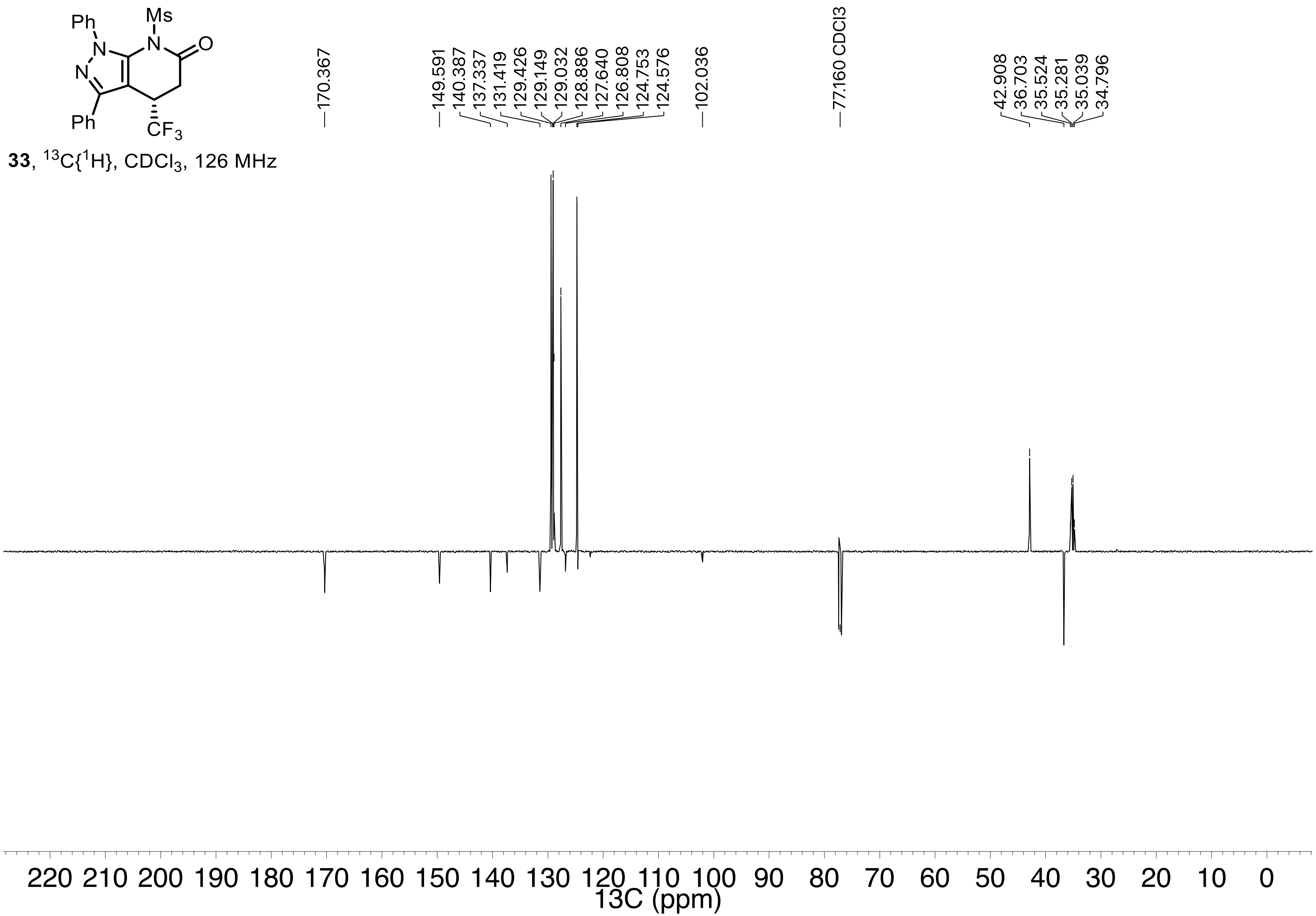
Ph
Ms
N
N
O
N
Ph
CF3
33, 13C{1H}, CDCl3, 126 MHz
170.367
149.591
140.387
137.337
131.419
129.426
129.149
129.032
128.886
127.640
126.808
124.753
124.576
102.036
77.160 CDCl3
42.908
36.703
35.524
35.281
35.039
34.796
220 210 200 190 180 170 160 150 140 130 120 110 100 90 80 70 60 50 40 30 20 10 0
13C (ppm)

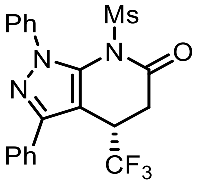

33, $^{19}F\{^1H\}$, $CDCl_3$, 377 MHz

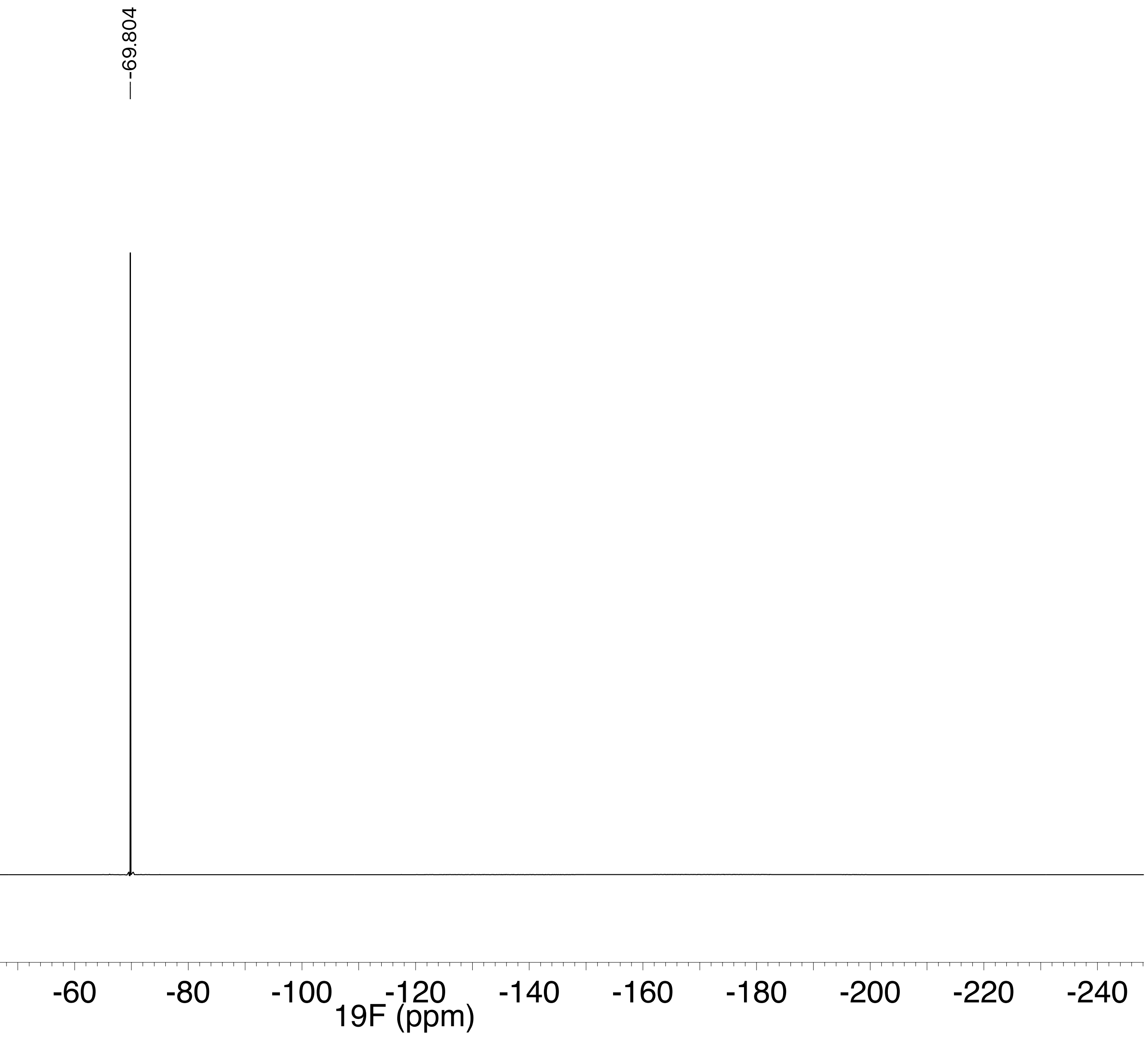

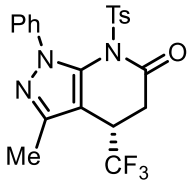

34, ^{1}H, $CDCl_3$, 500 MHz

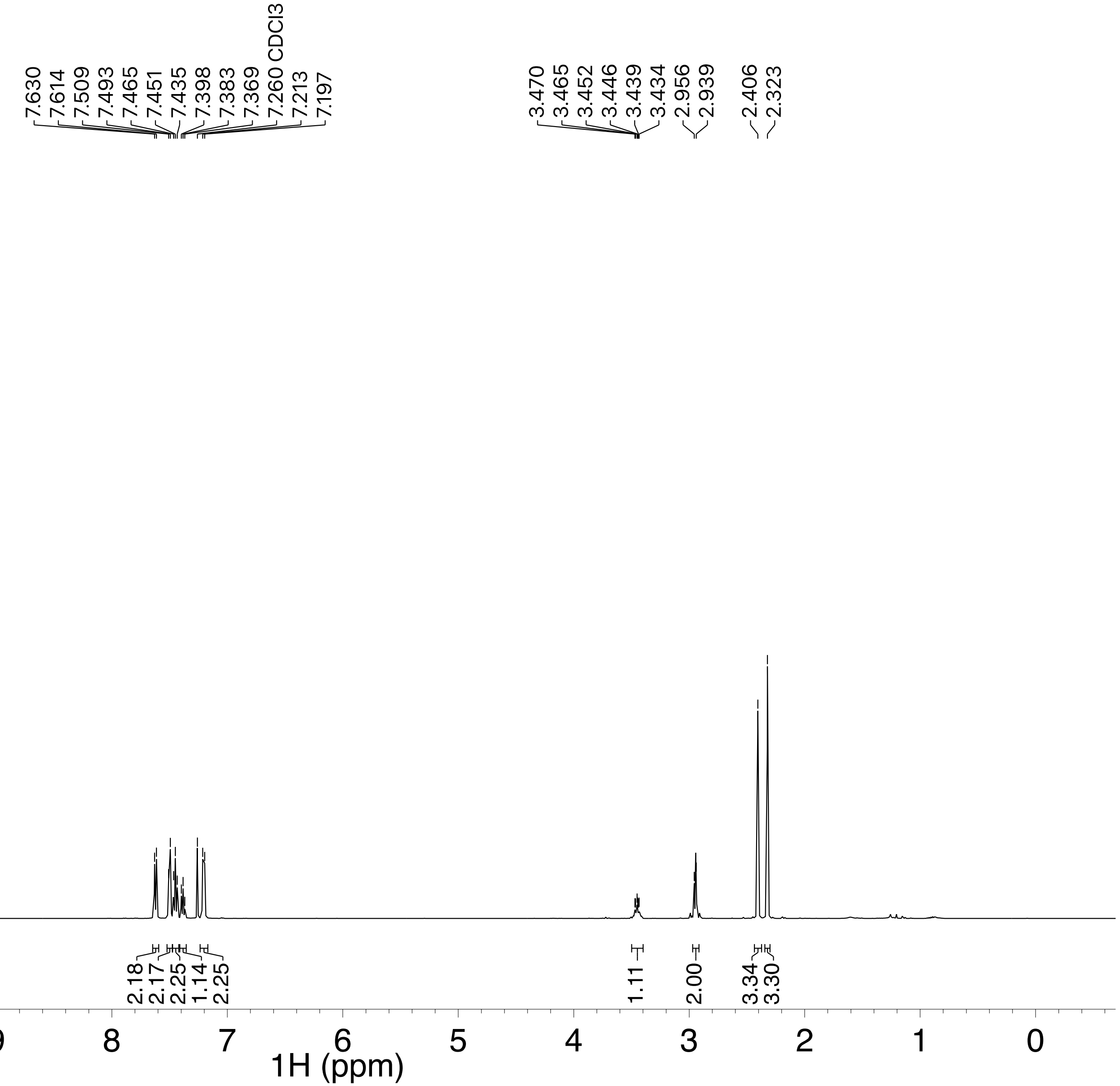

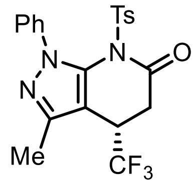

34, $^{13}C\{^{1}H\}$, $CDCl_3$, 126 MHz

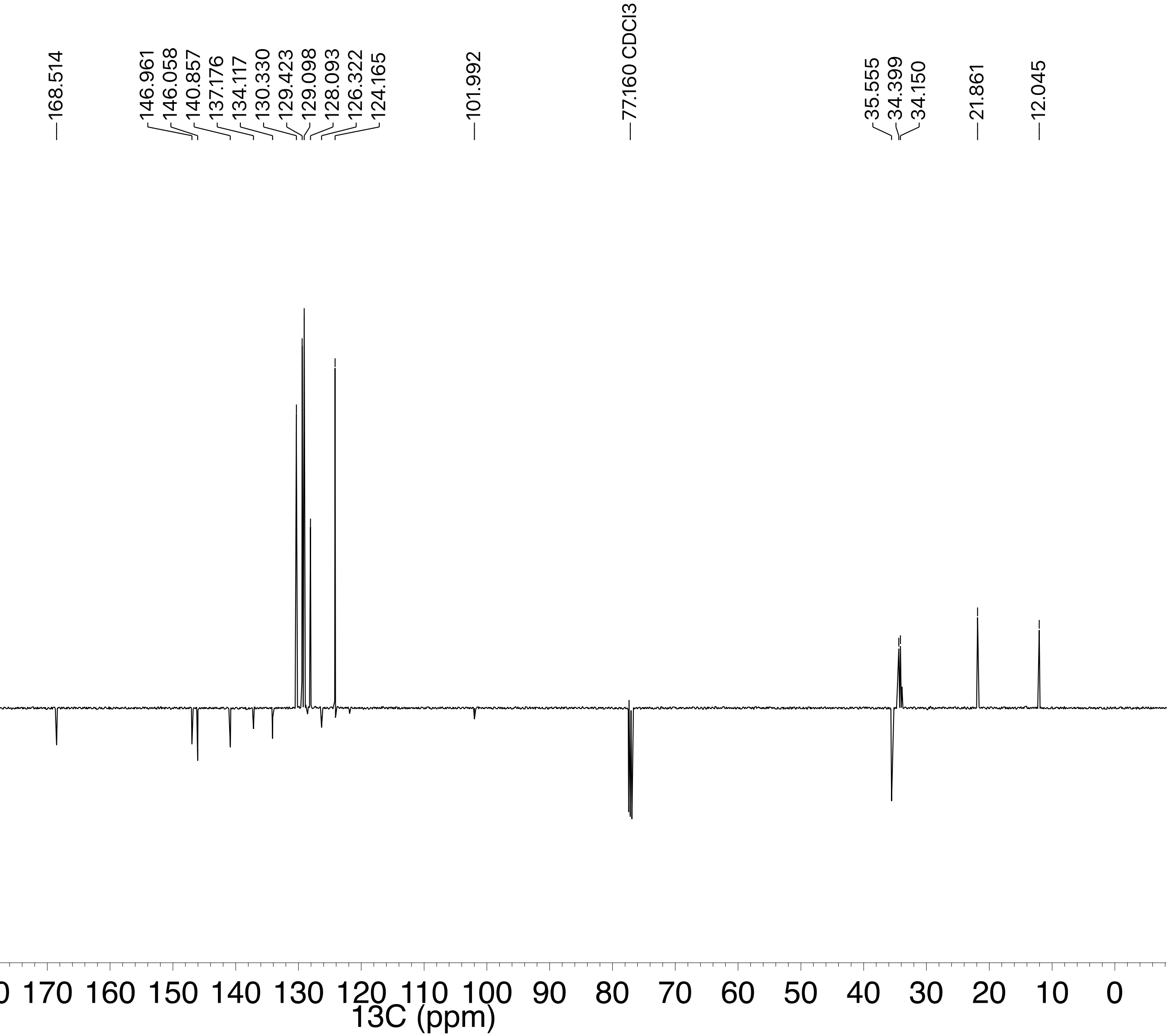

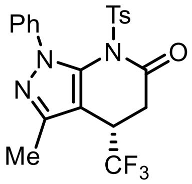

34, $^{19}F\{^{1}H\}$, $CDCl_3$, 377 MHz

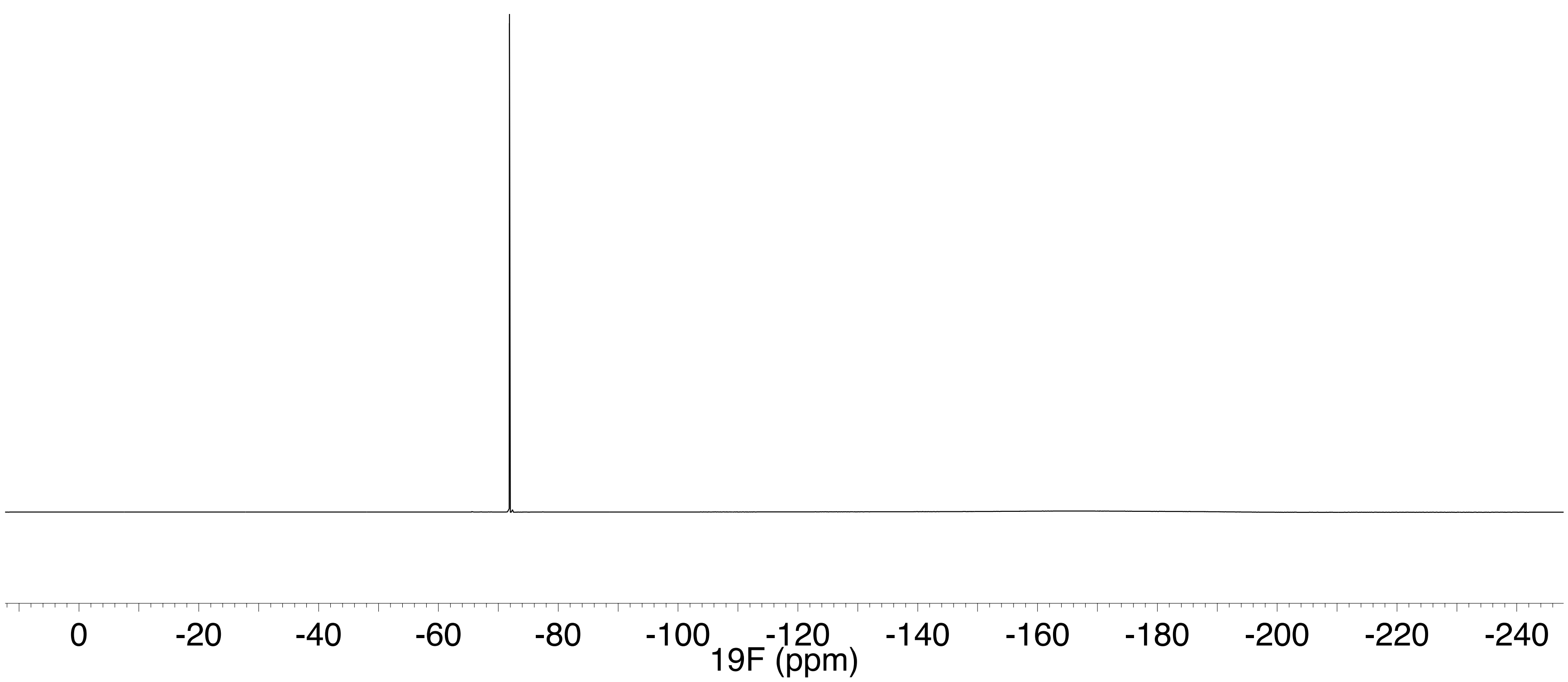

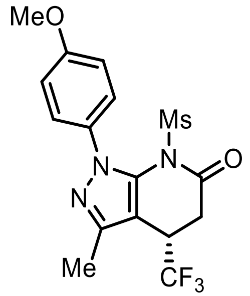

27, ^{1}H, $CDCl_3$, 500 MHz

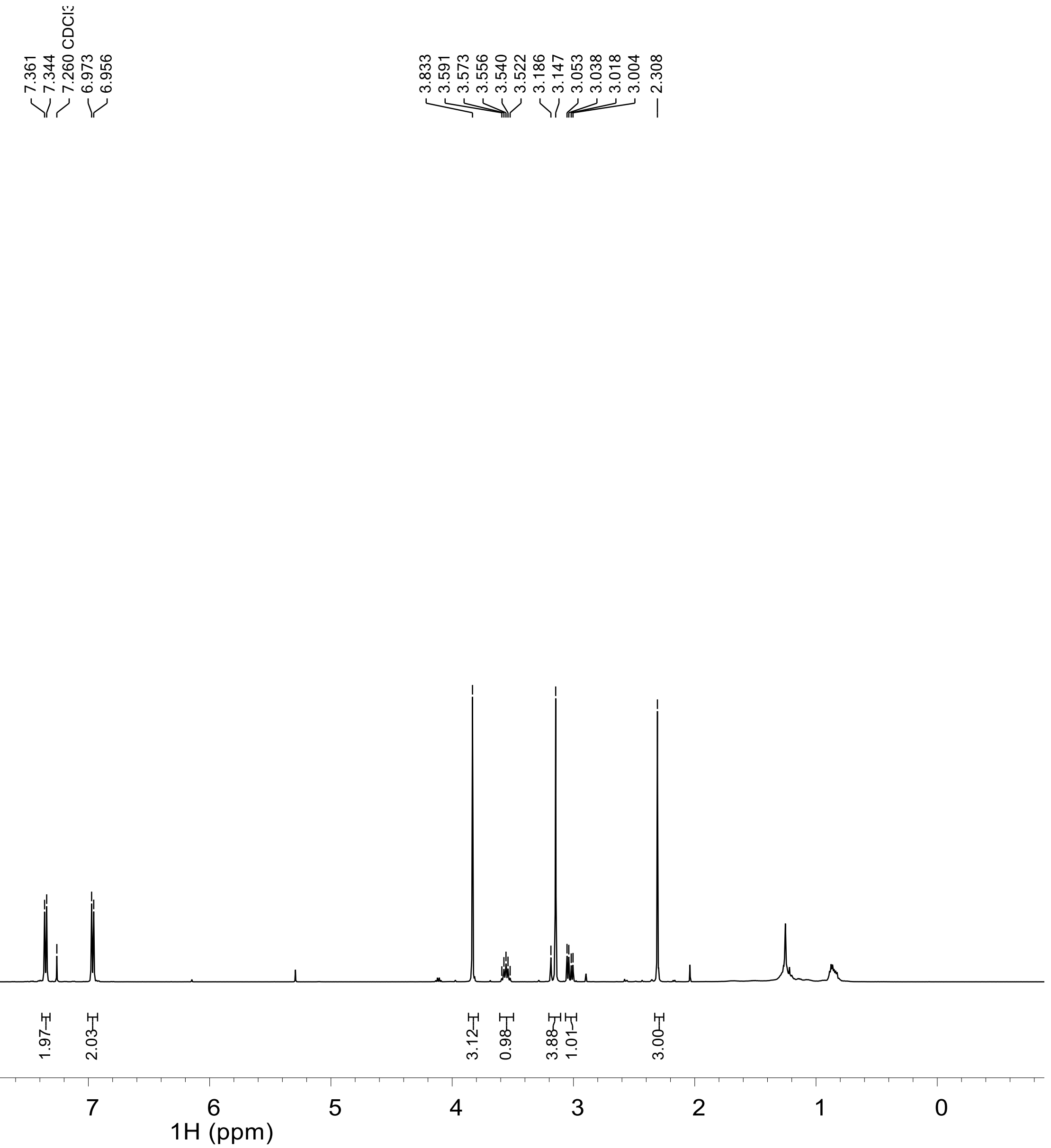

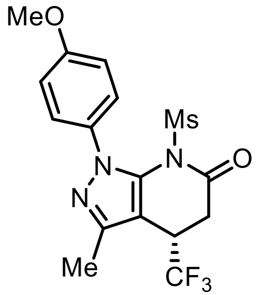

27, $^{13}C\{^{1}H\}$, $CDCl_3$, 126 MHz

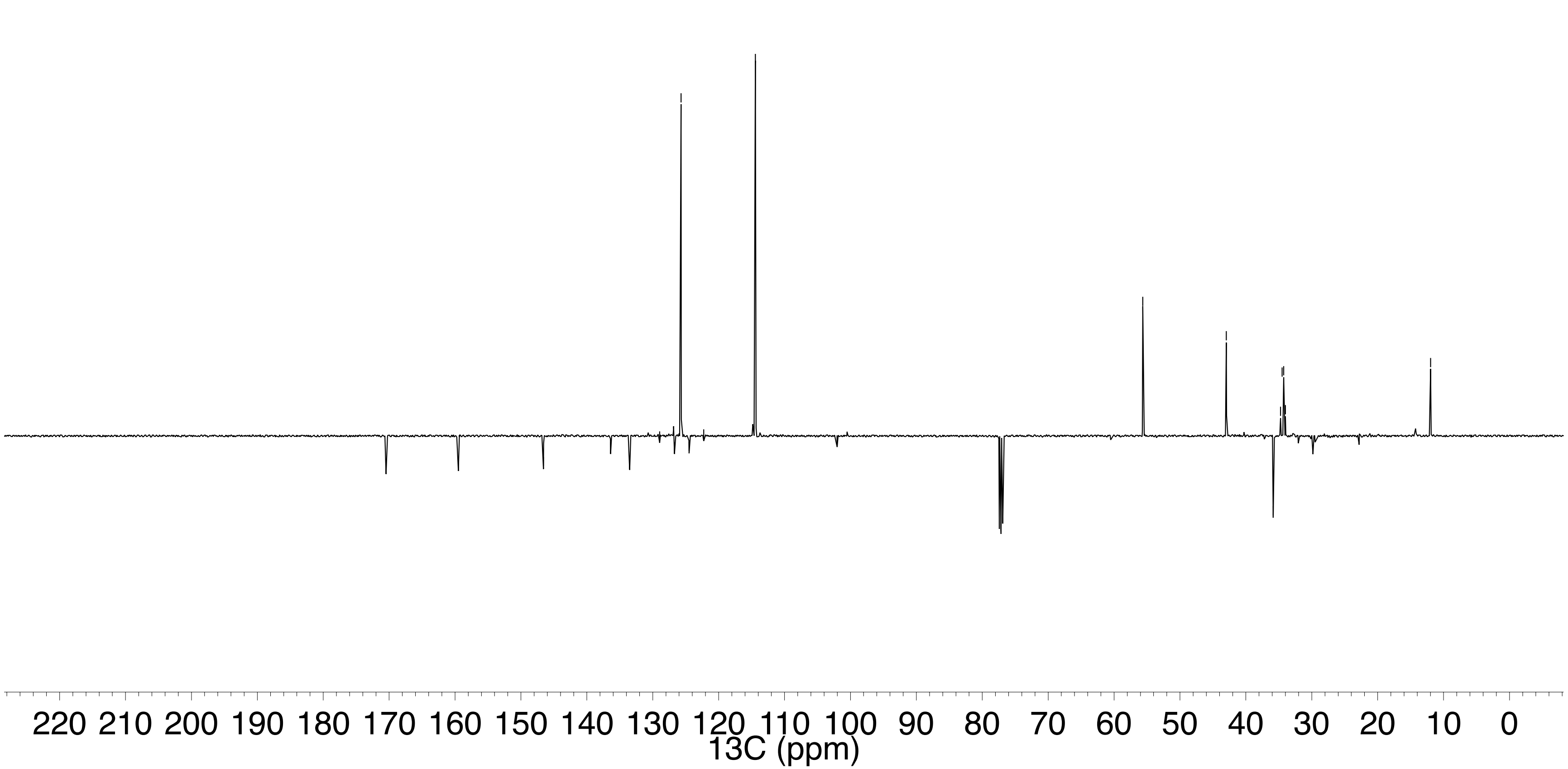

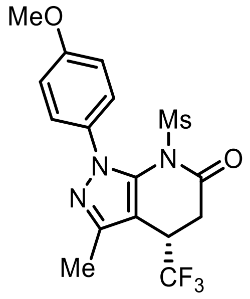

27, $^{19}F\{^1H\}$, $CDCl_3$, 377 MHz

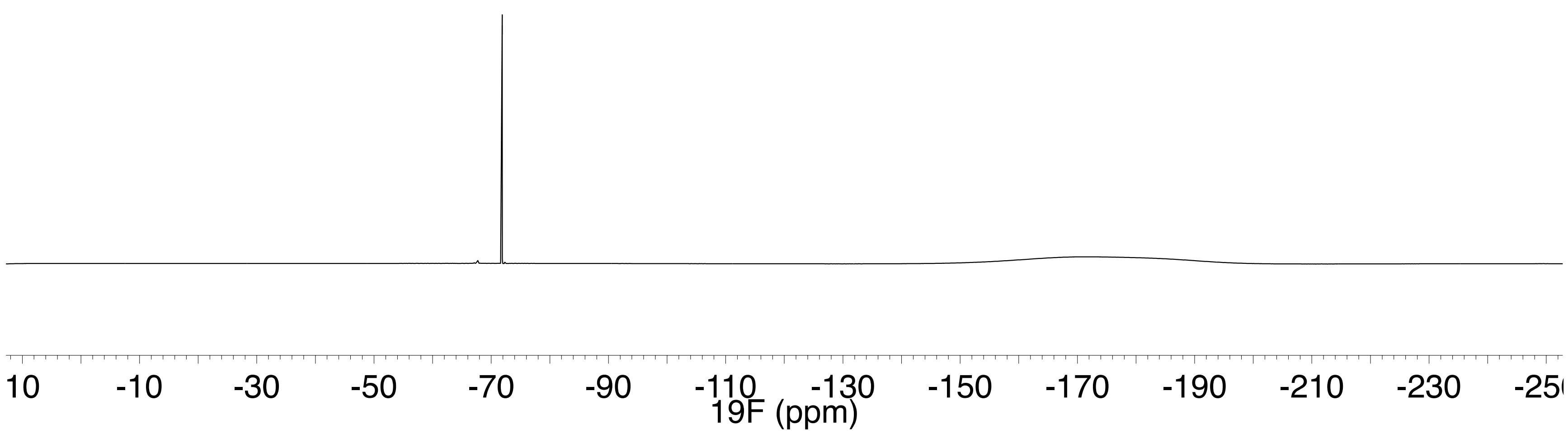

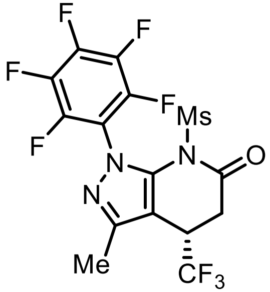

35, ^{1}H, $CDCl_3$, 500 MHz

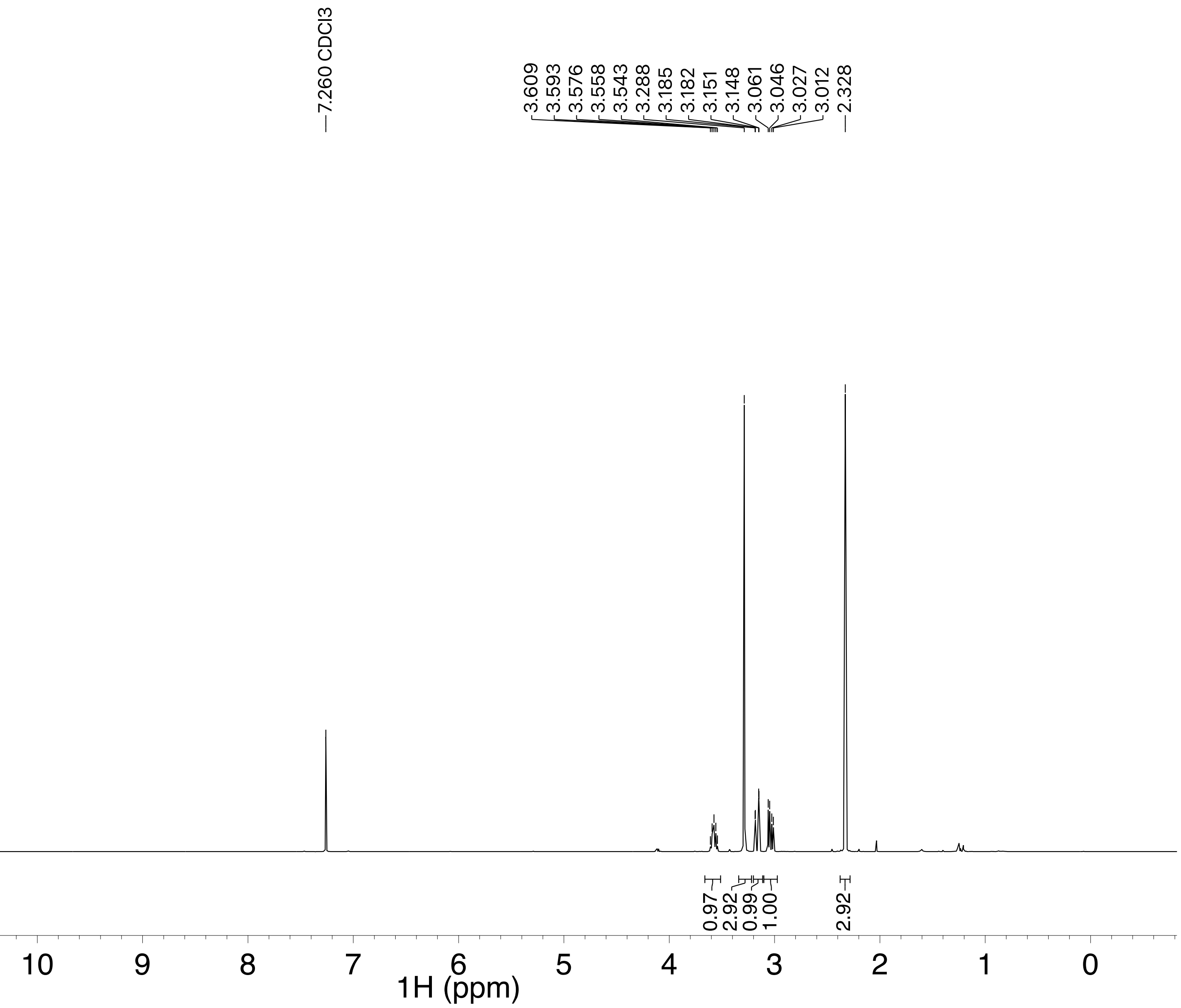

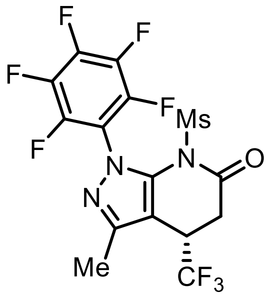

35, $^{13}C\{^{1}H\}$, $CDCl_3$, 126 MHz

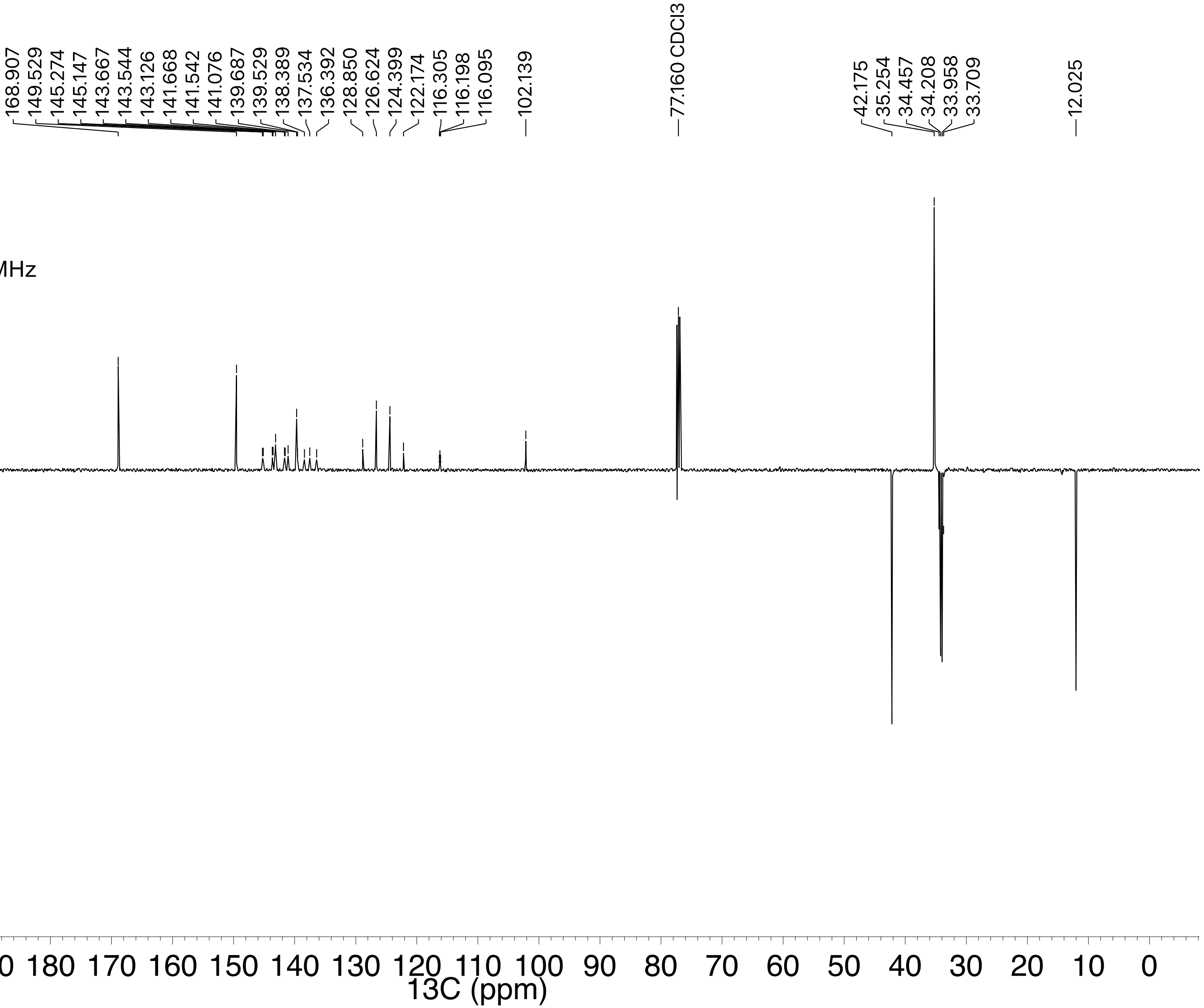

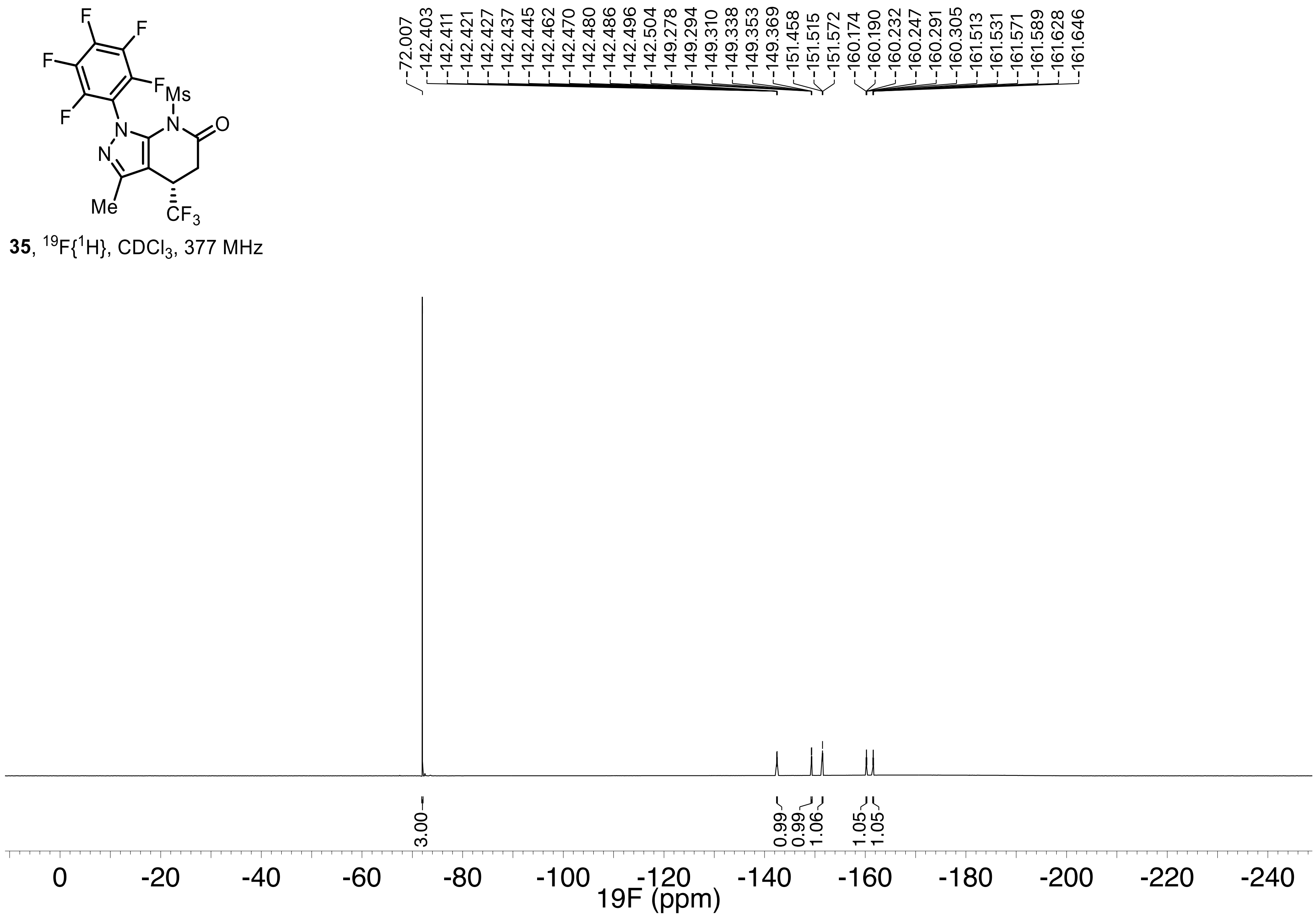

35, $^{19}F\{^{1}H\}$, $CDCl_3$, 377 MHz

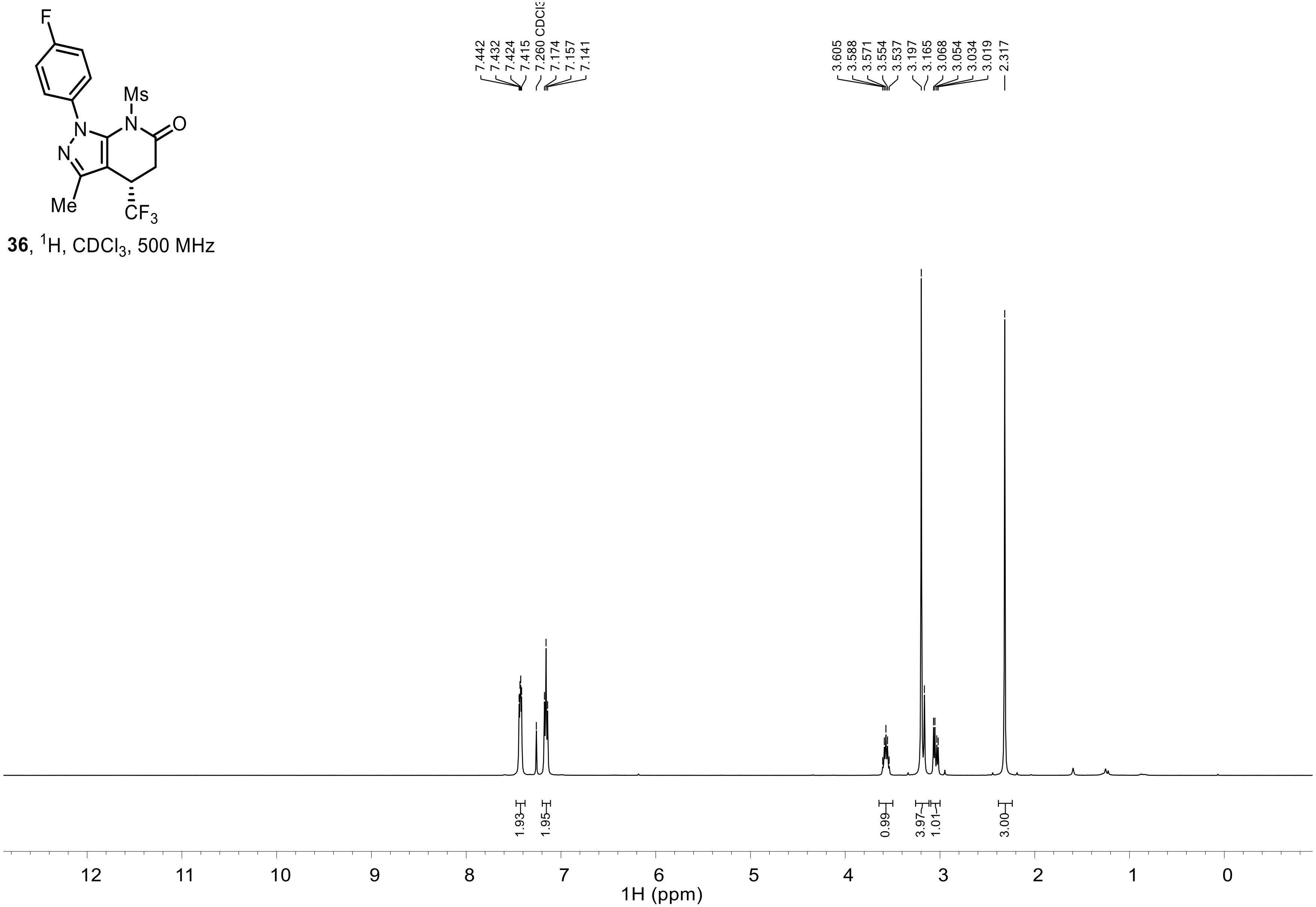

36, ^{1}H, $CDCl_3$, 500 MHz

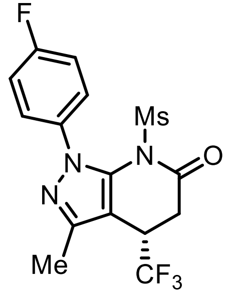

36, $^{13}C\{^1H\}$, $CDCl_3$, 126 MHz

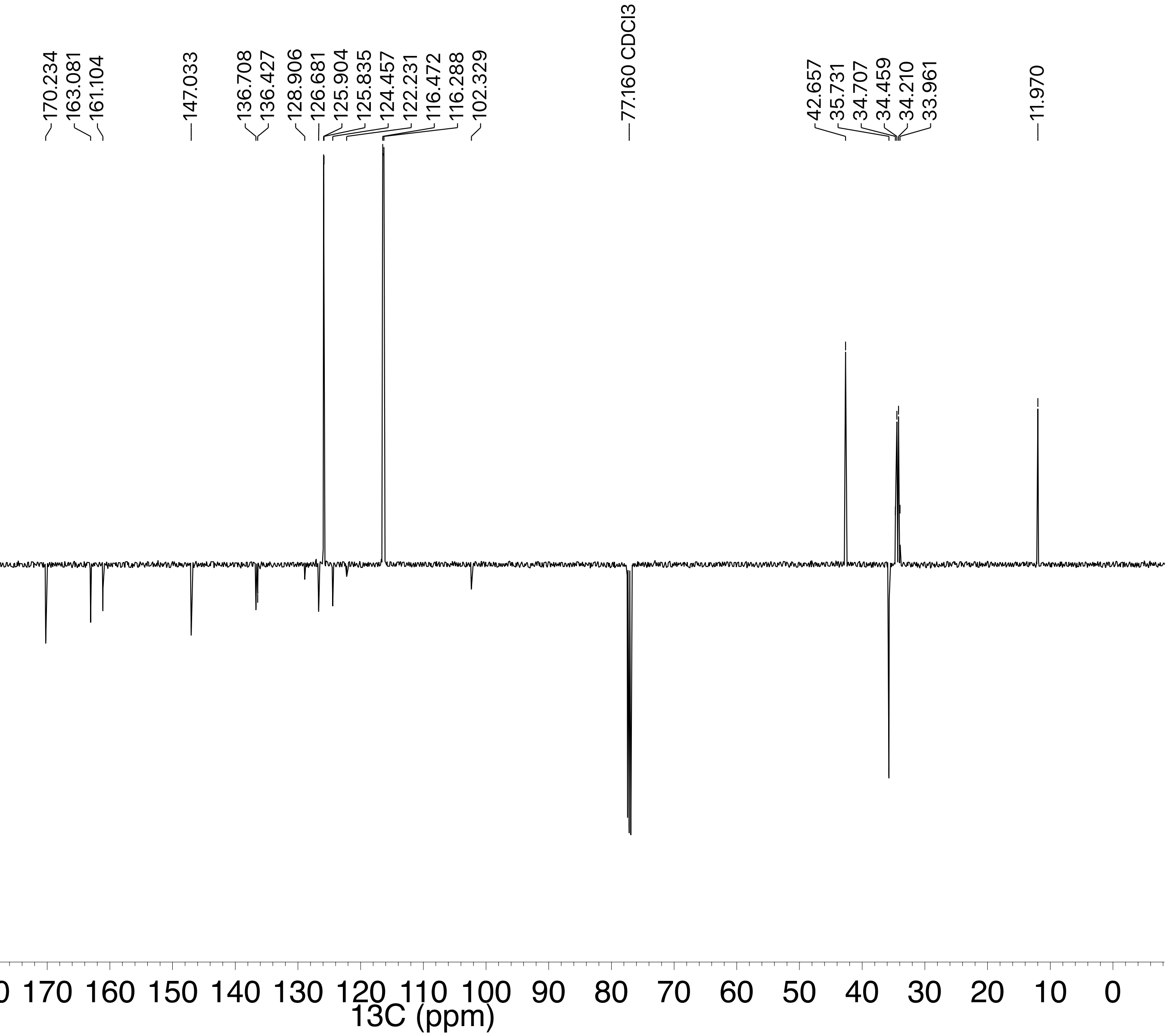

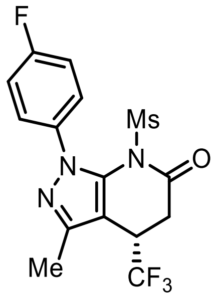

36, $^{19}F\{^{1}H\}$, $CDCl_3$, 377 MHz

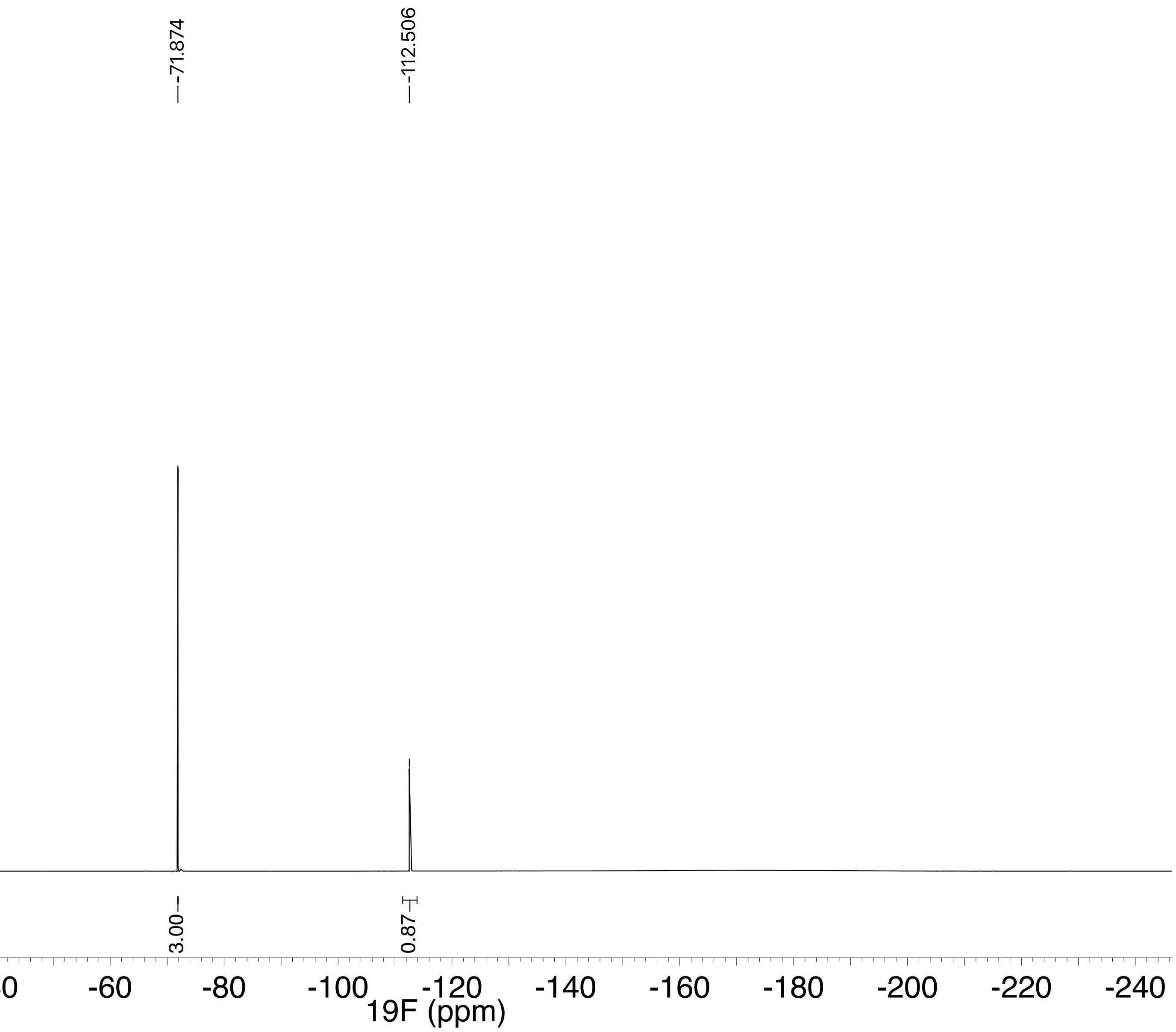

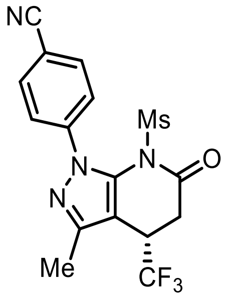

37, ^{1}H, $CDCl_3$, 400 MHz

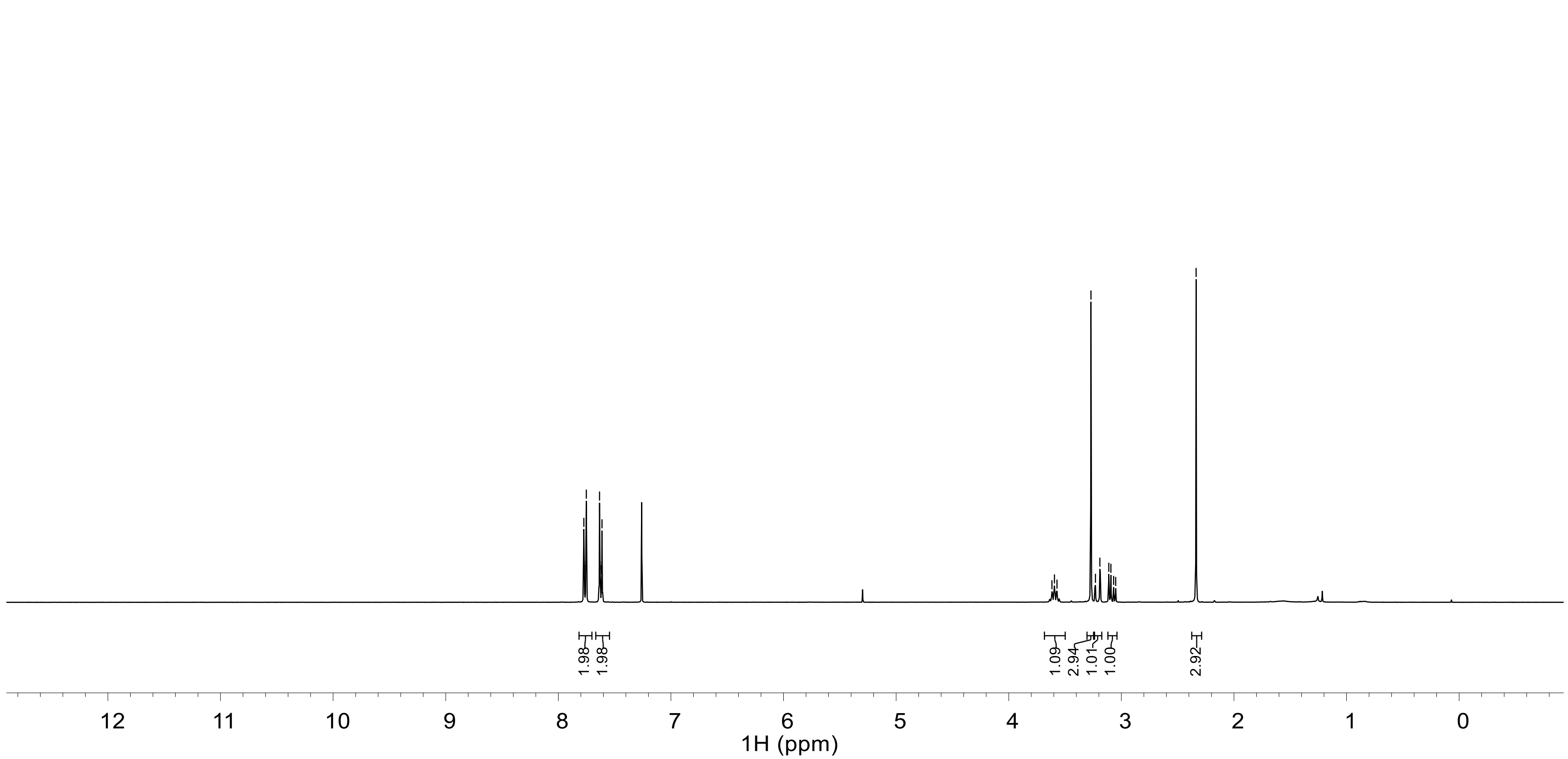

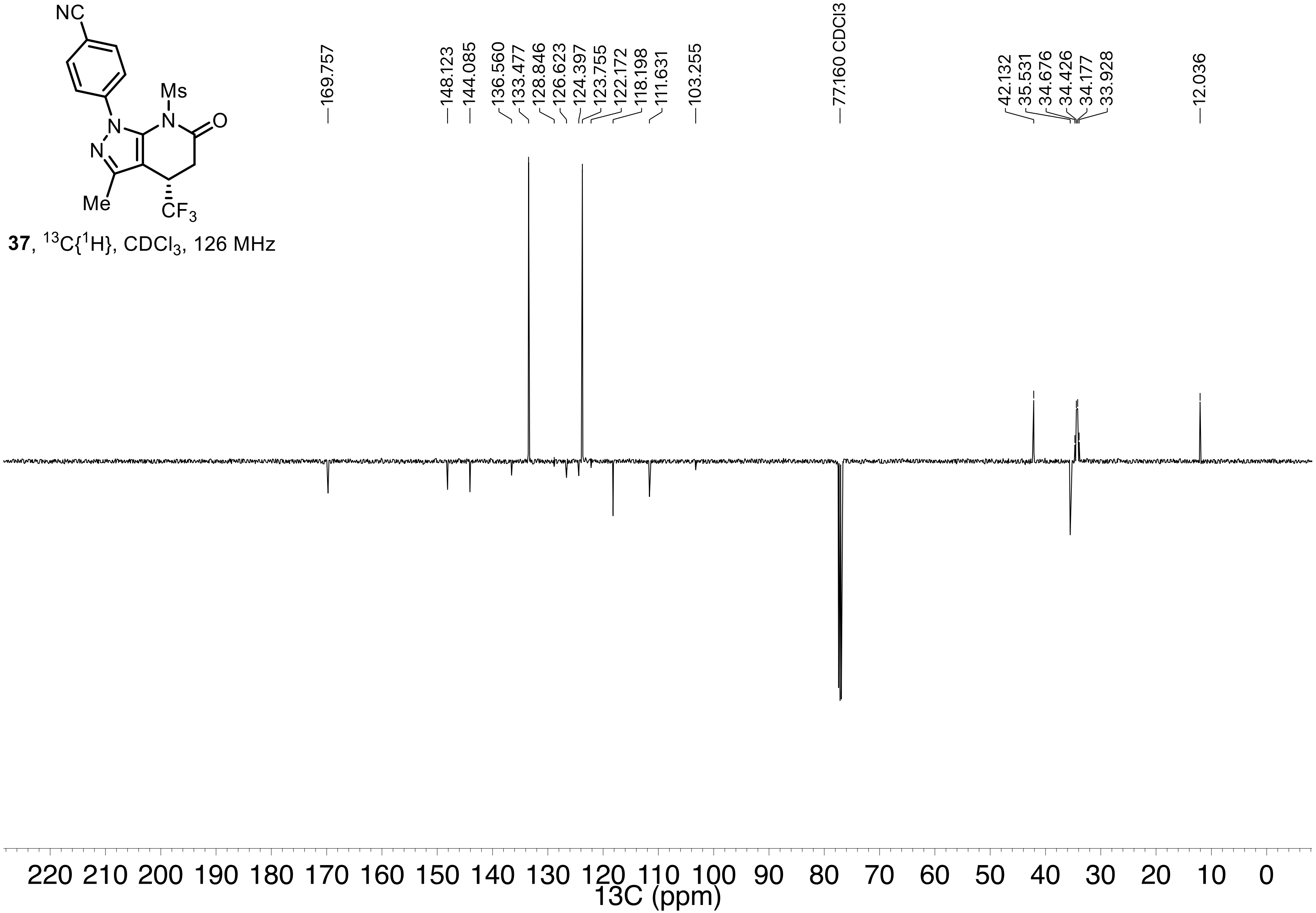

37, $^{13}C\{^{1}H\}$, $CDCl_3$, 126 MHz

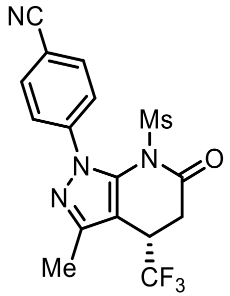

37, $^{19}F\{^{1}H\}$, $CDCl_3$, 377 MHz

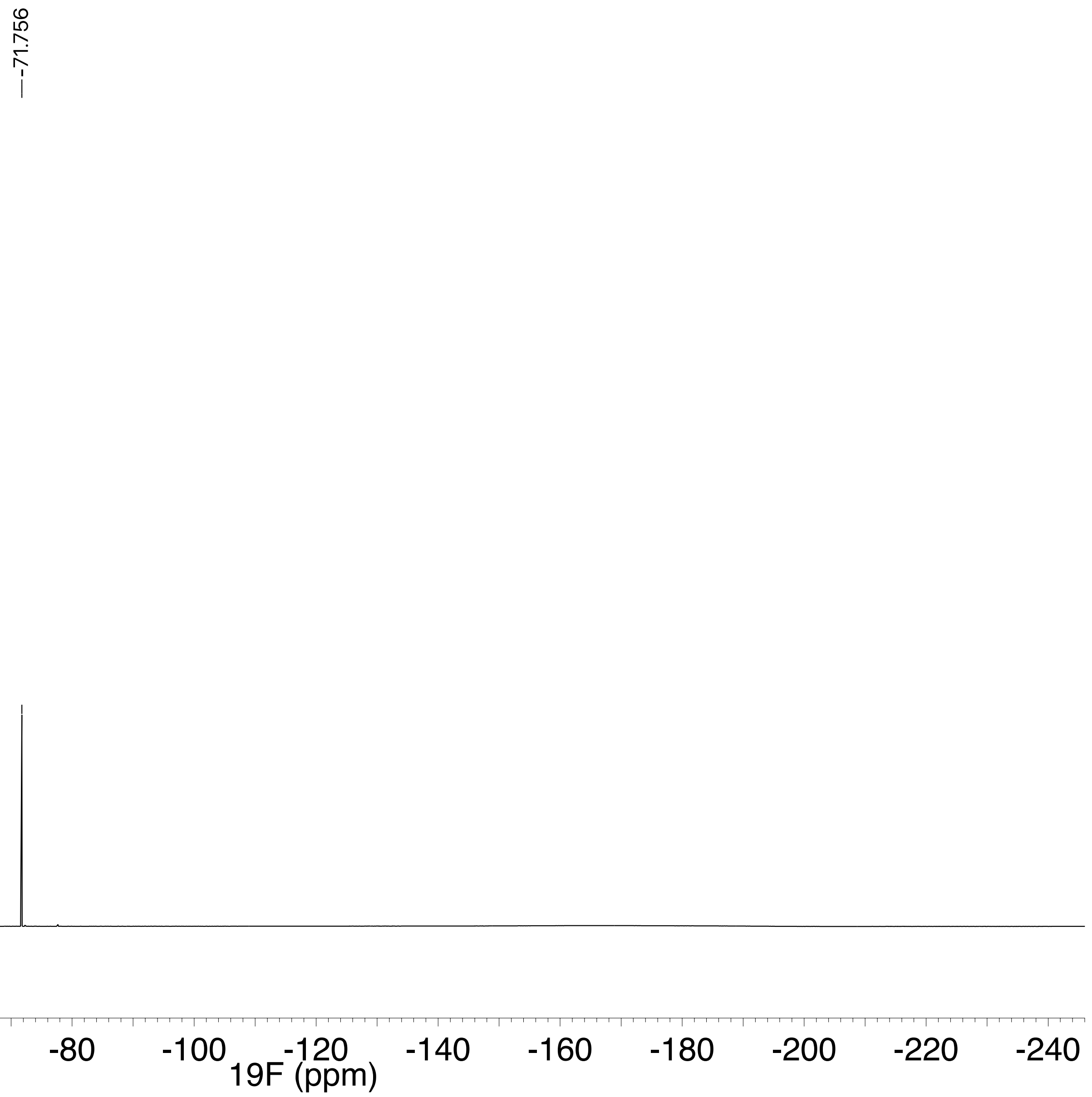

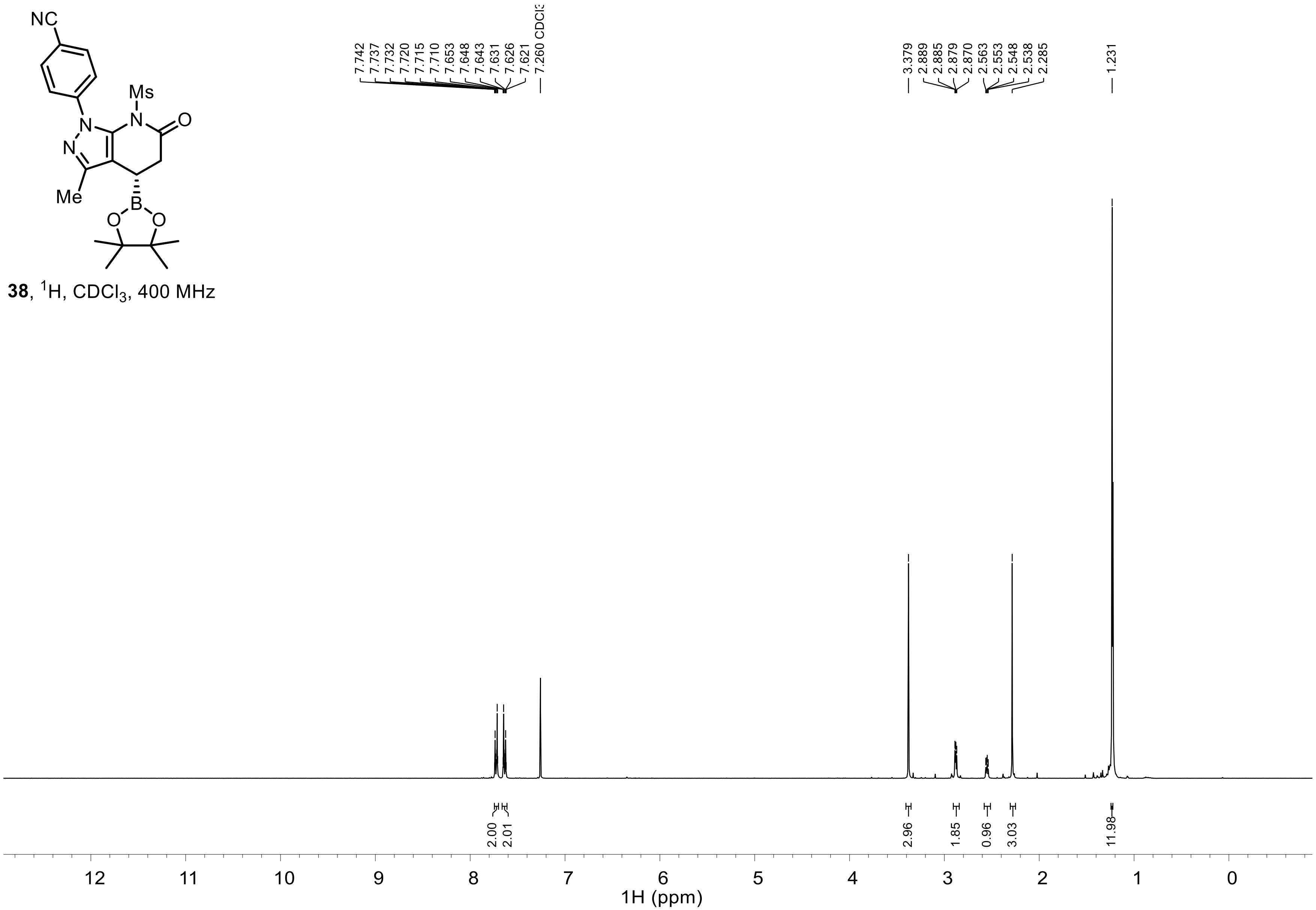

38, ^{1}H, $CDCl_3$, 400 MHz

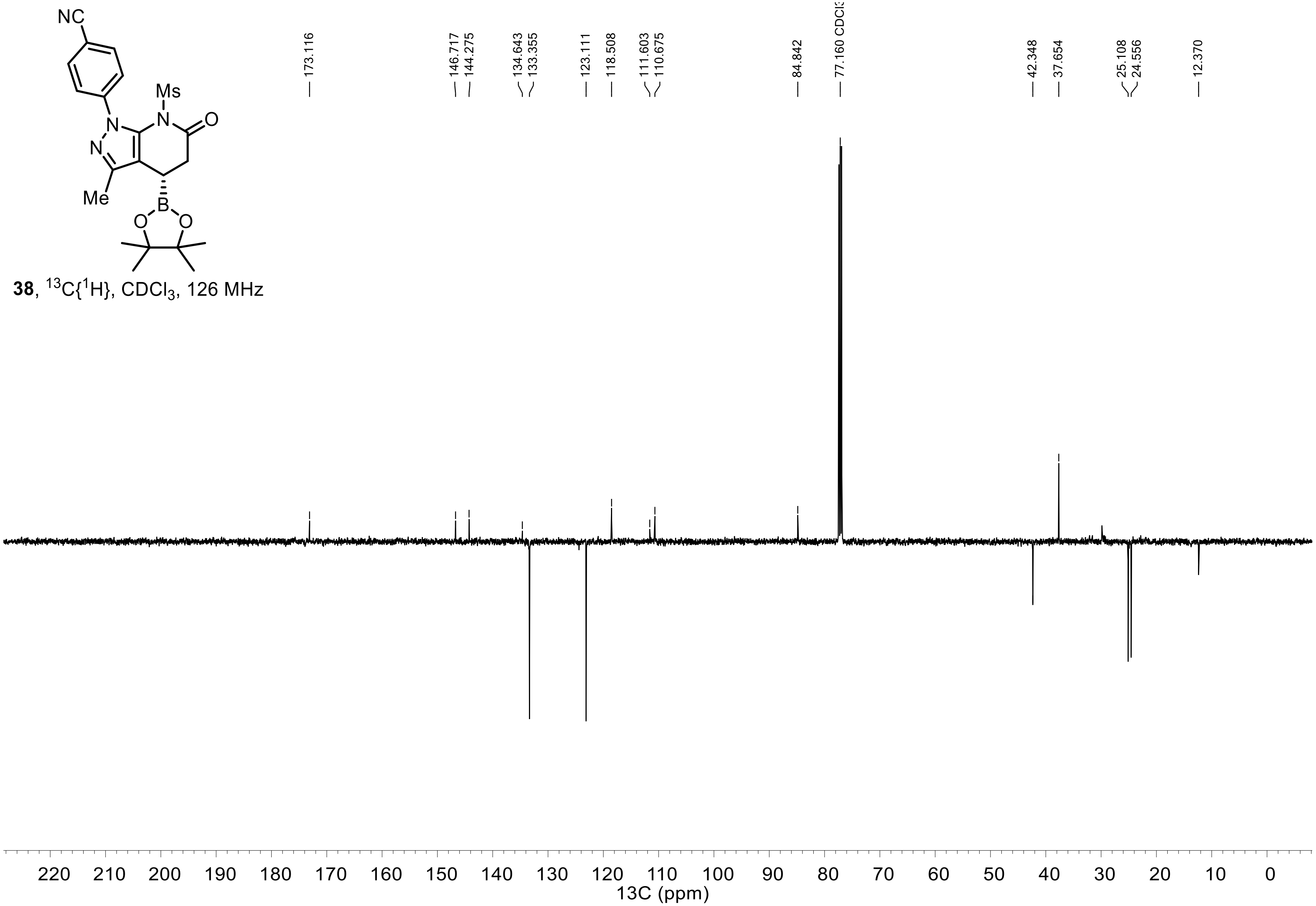

38, $^{13}C\{^{1}H\}$, $CDCl_3$, 126 MHz

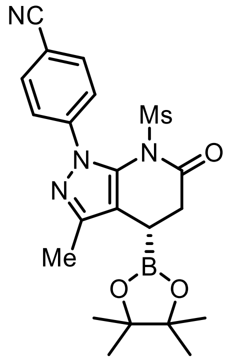

38, $^{11}B\{^{1}H\}$, $CDCl_3$, 128 MHz

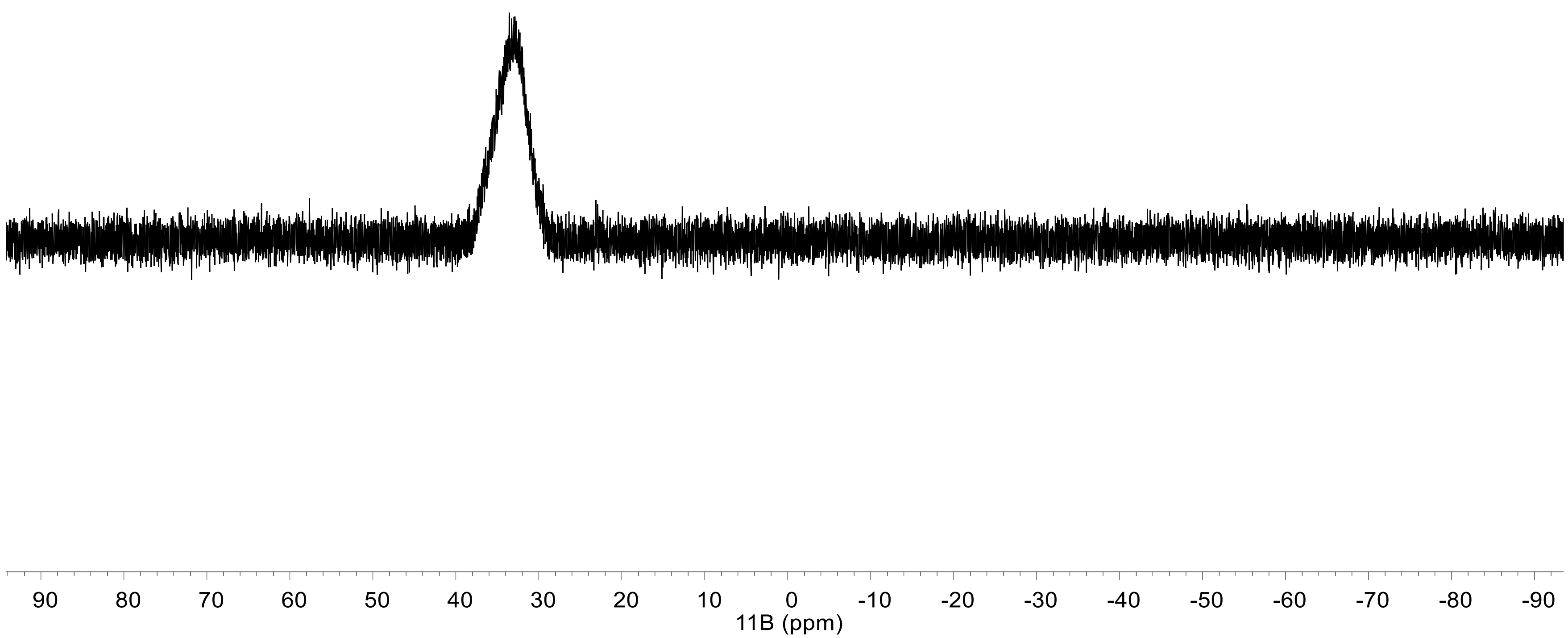

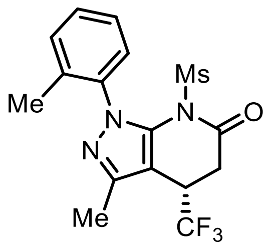

39, ^{1}H, DMSO-d_6, 500 MHz

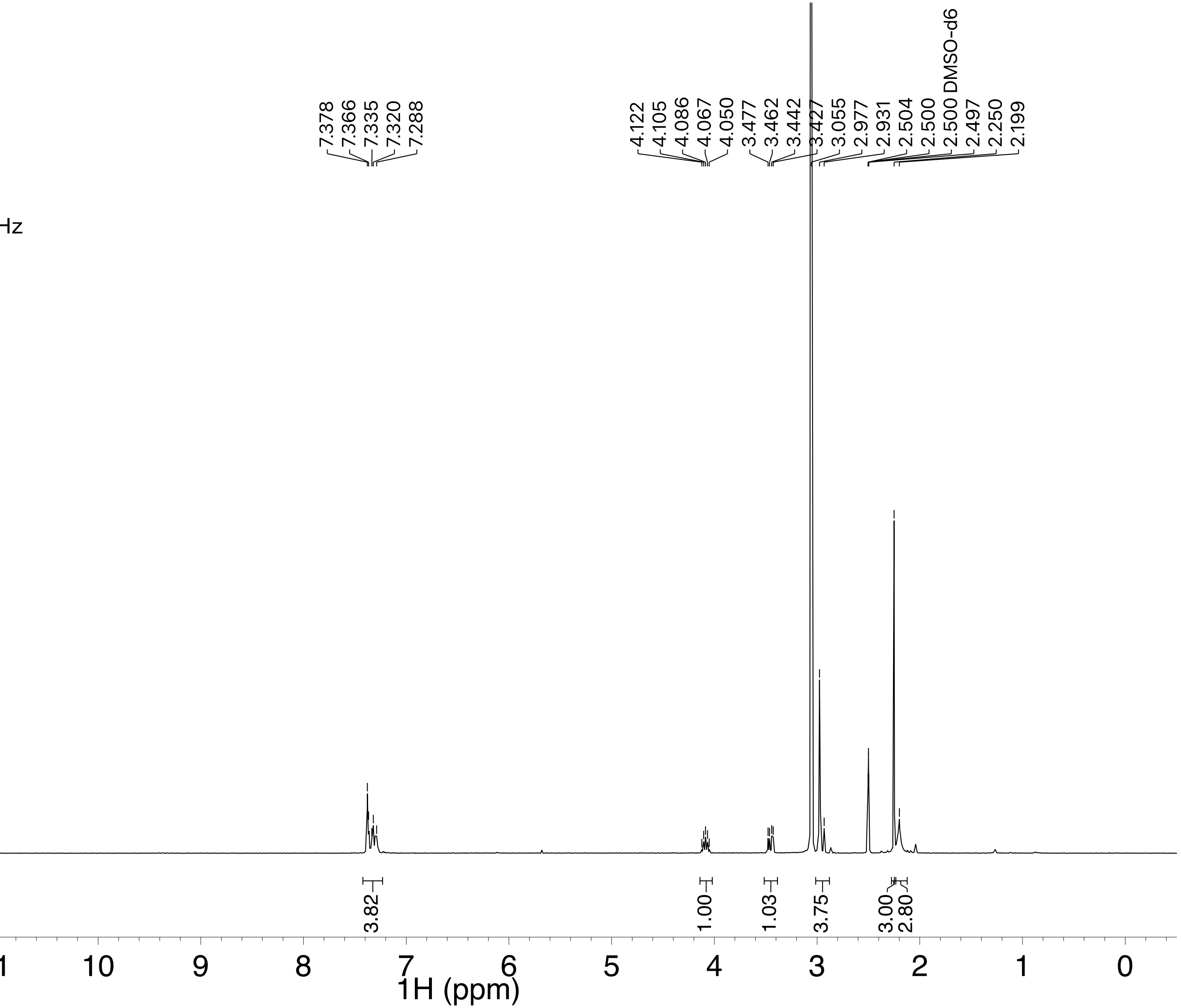

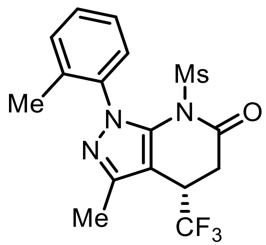

39, $^{13}C\{^{1}H\}$, DMSO-d_6, 126 MHz

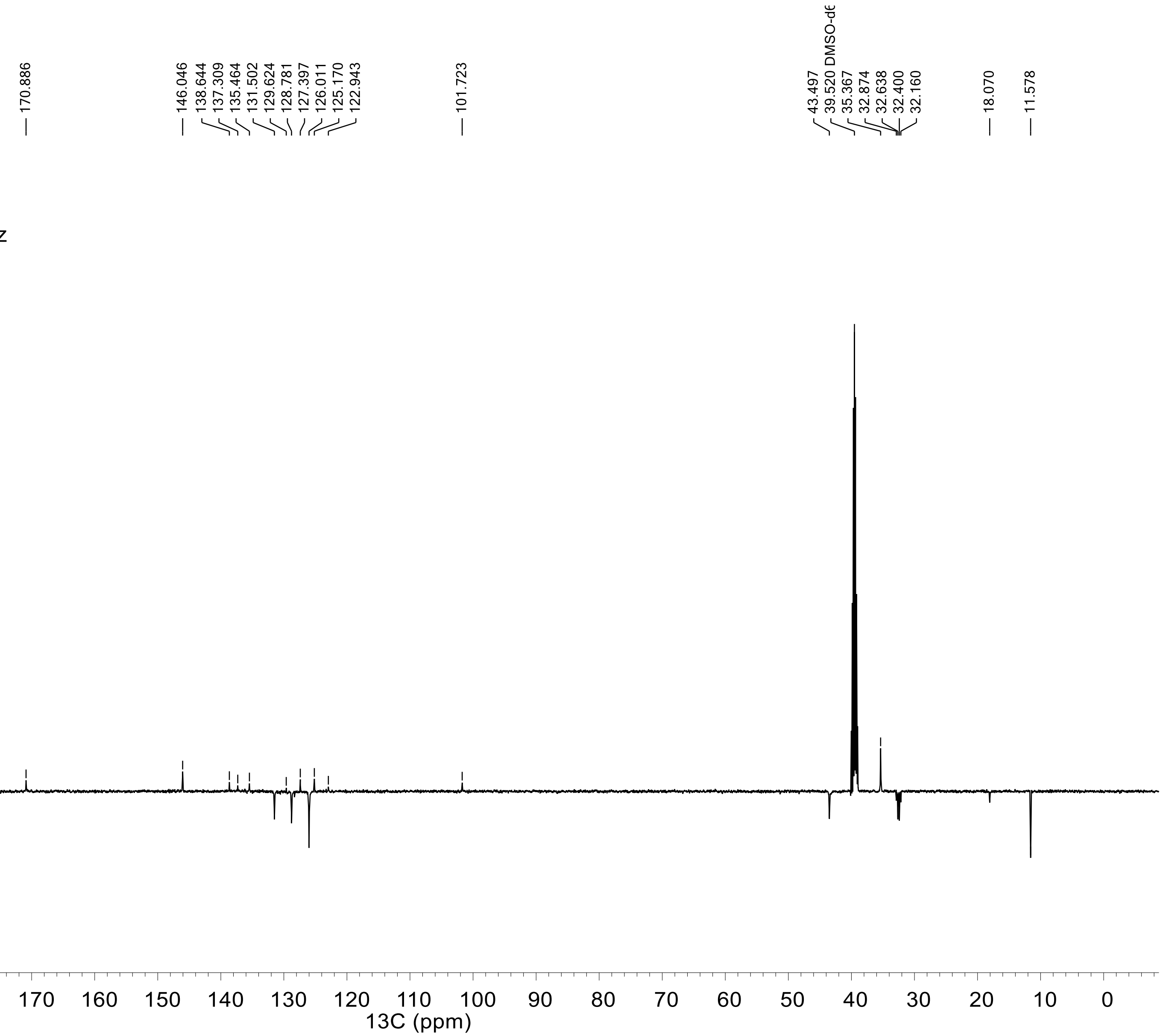

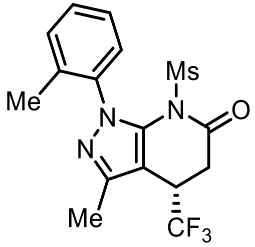

39, $^{19}F\{^{1}H\}$, DMSO-d$_6$, 470 MHz

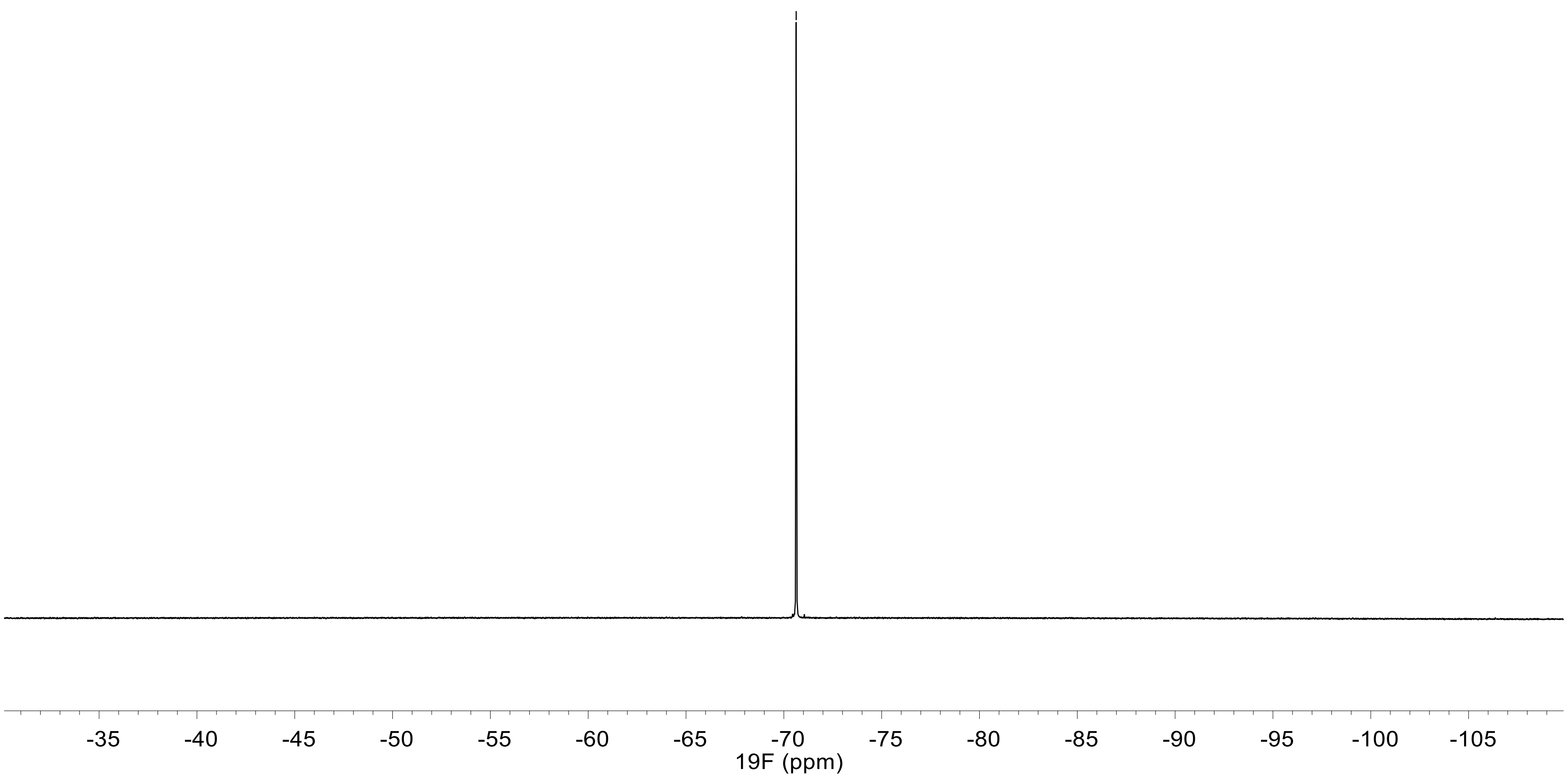

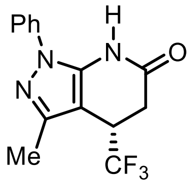

41, ^{1}H, $CDCl_3$, 400 MHz

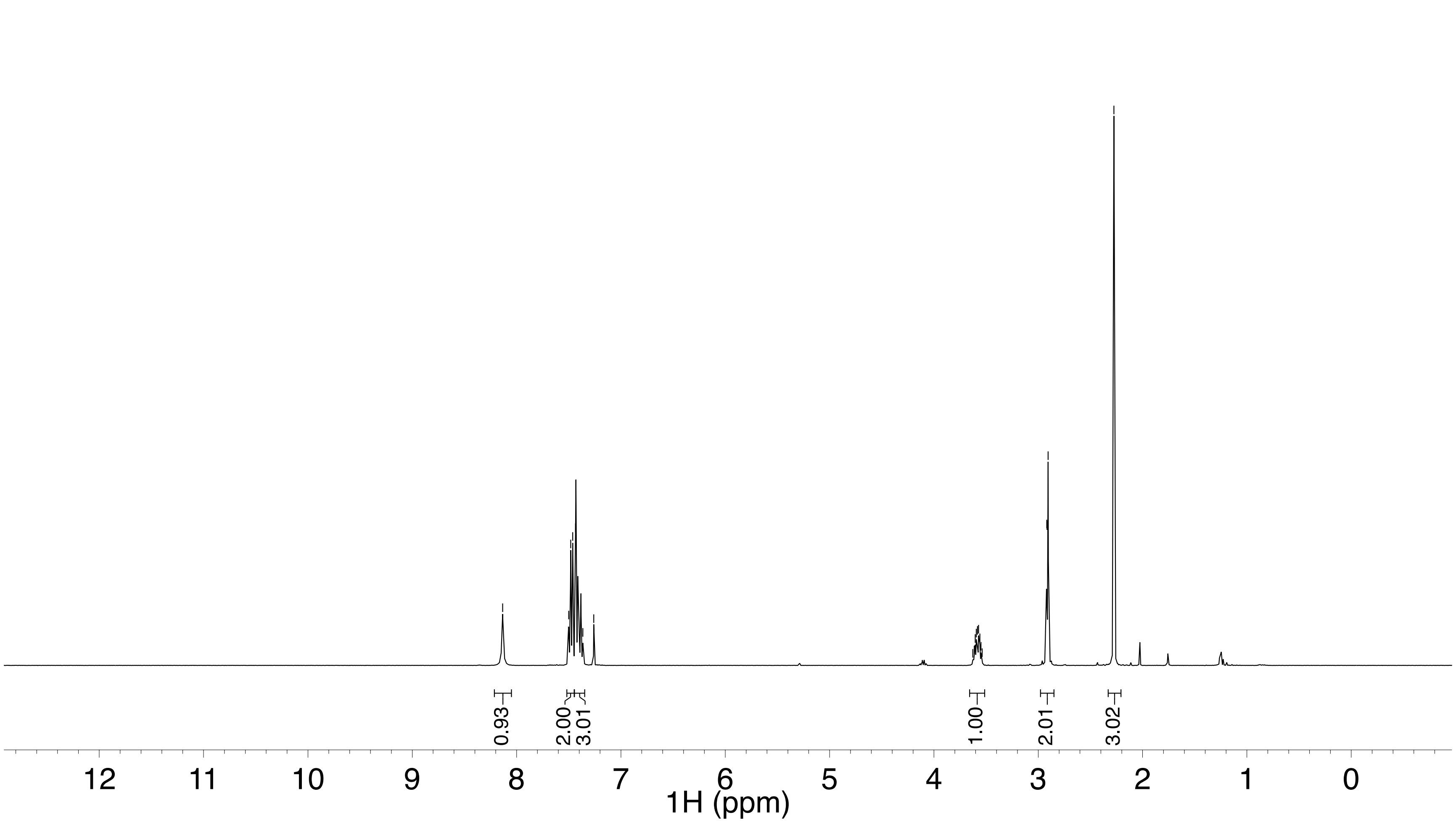

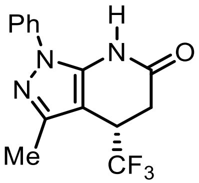

41, $^{13}C\{^{1}H\}$, $CDCl_3$, 126 MHz

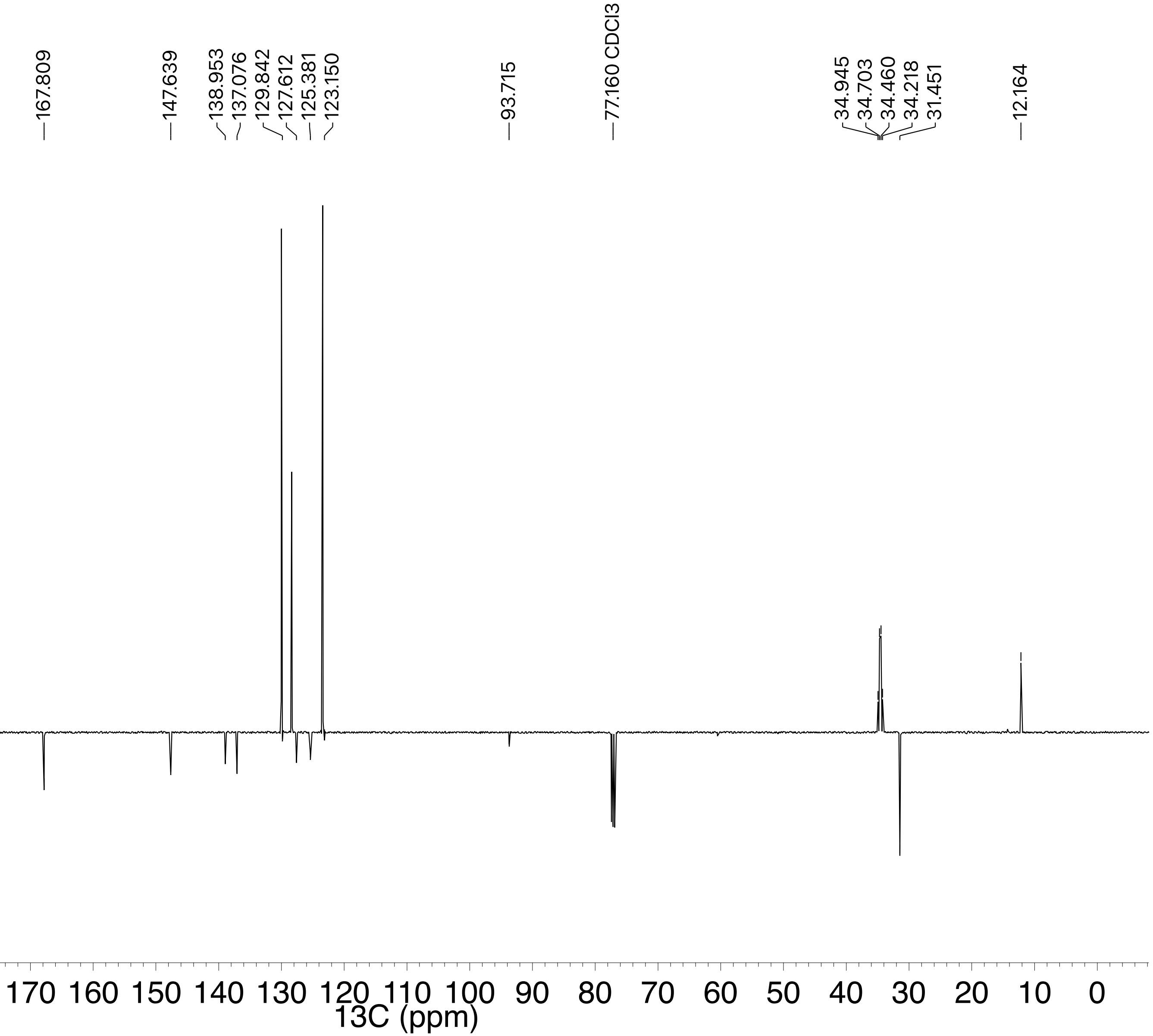

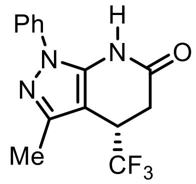

41, $^{19}F\{^1H\}$, $CDCl_3$, 377 MHz

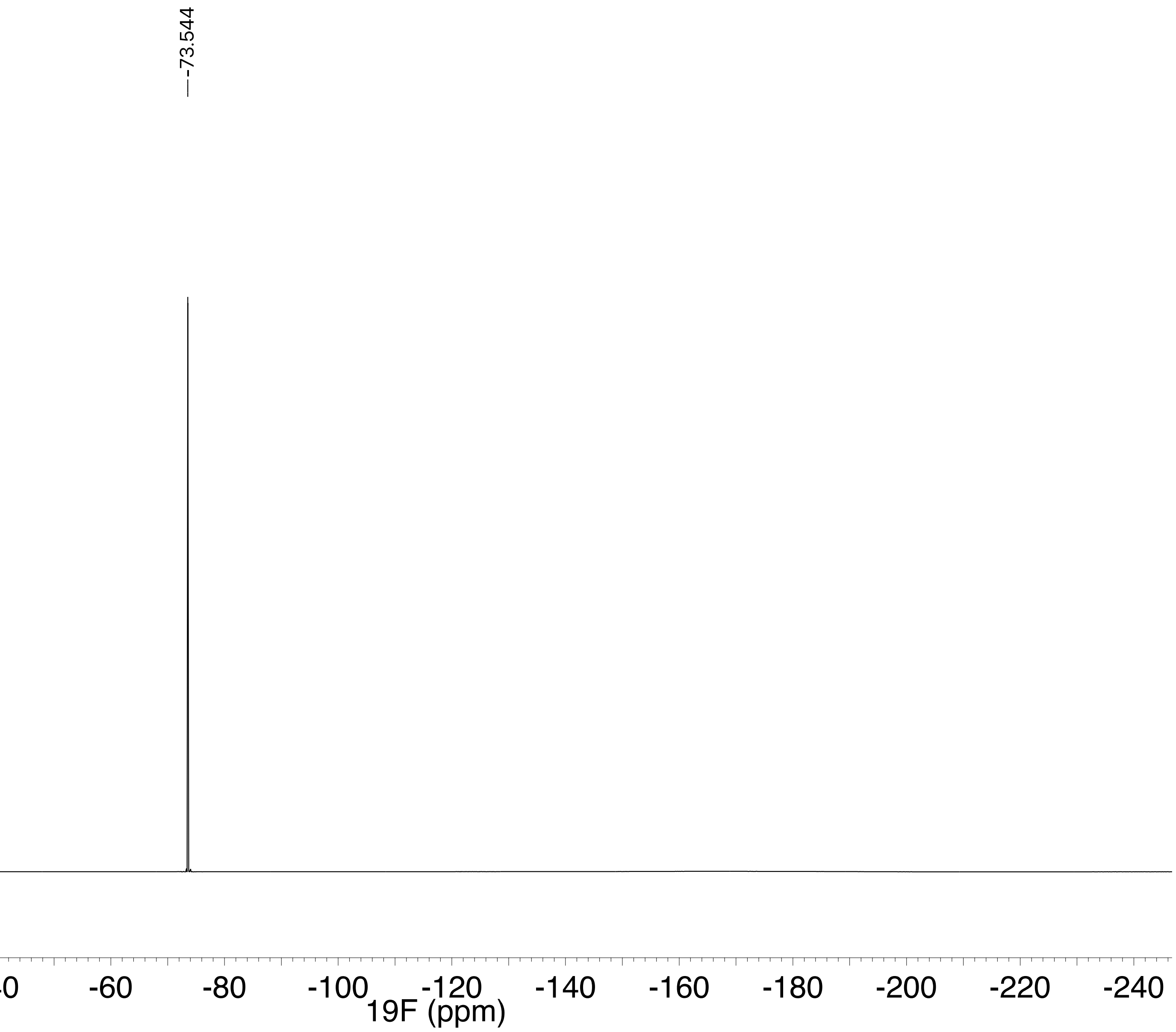

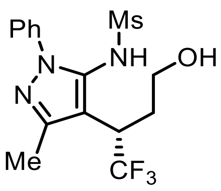

40, ^{1}H, $CDCl_3$, 500 MHz

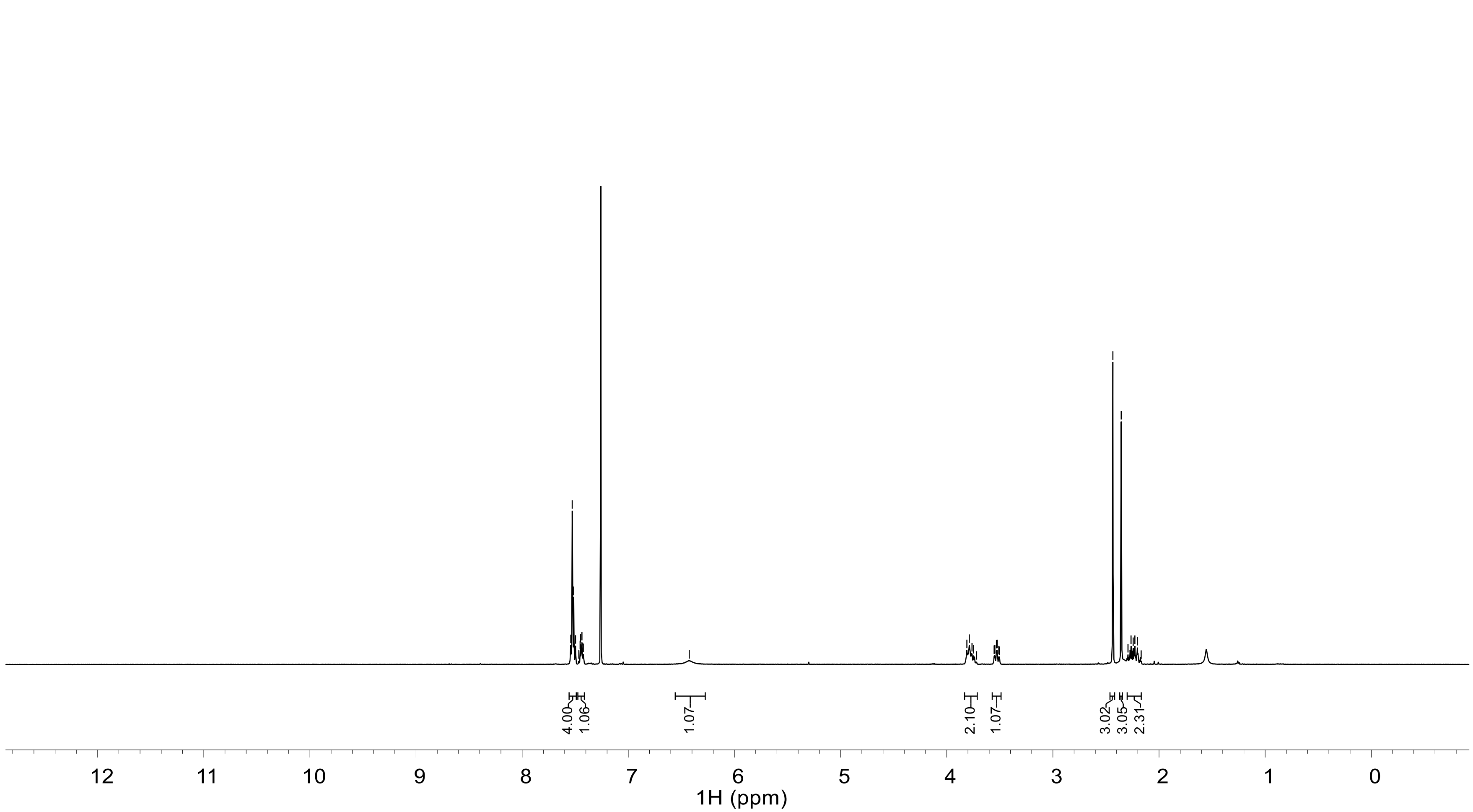

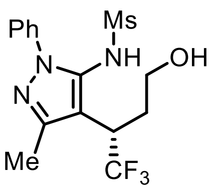

40, $^{13}C\{^{1}H\}$, $CDCl_3$,126 MHz

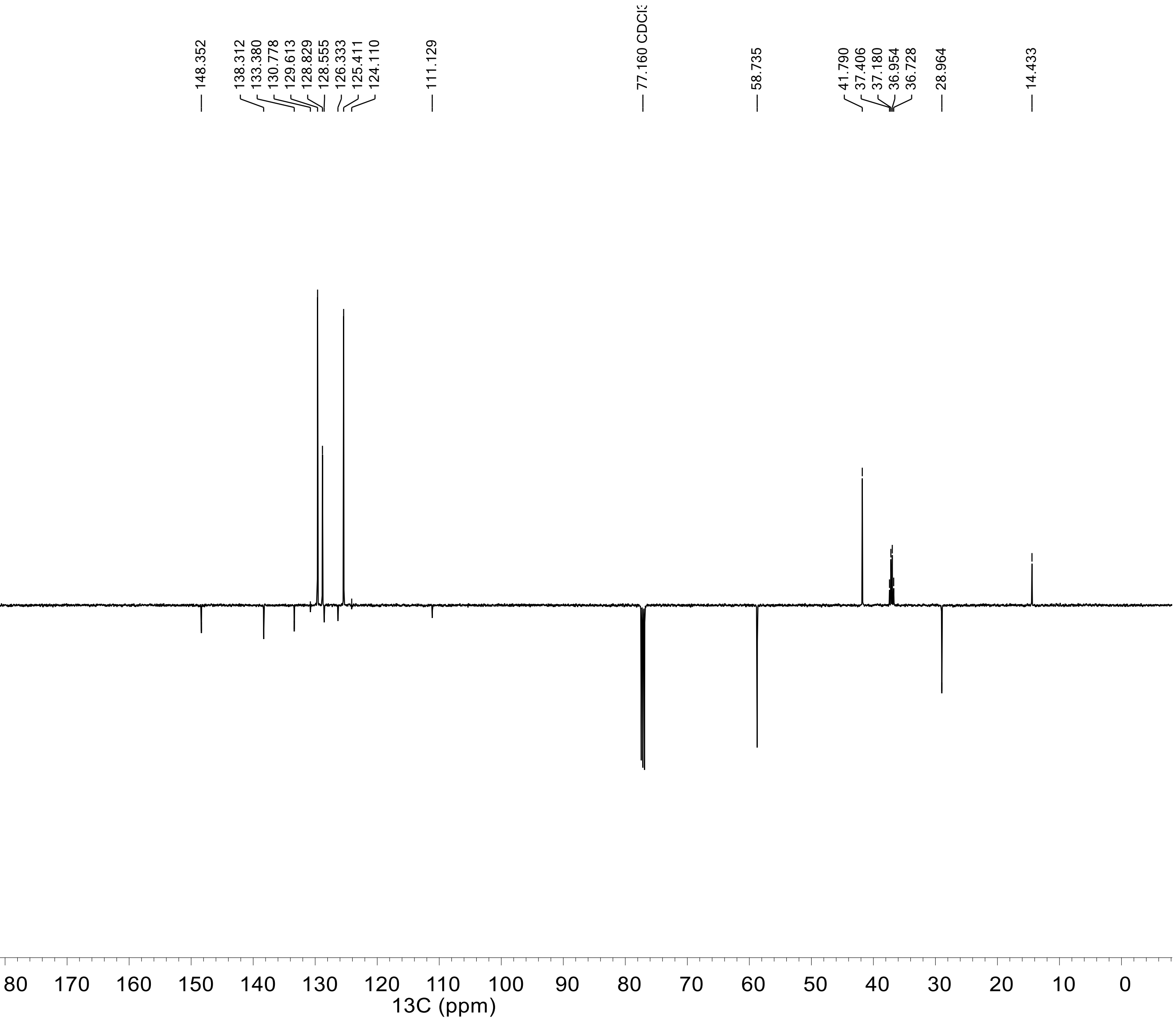

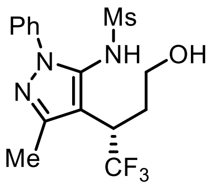

40, $^{19}F\{^1H\}$, $CDCl_3$, 376 MHz

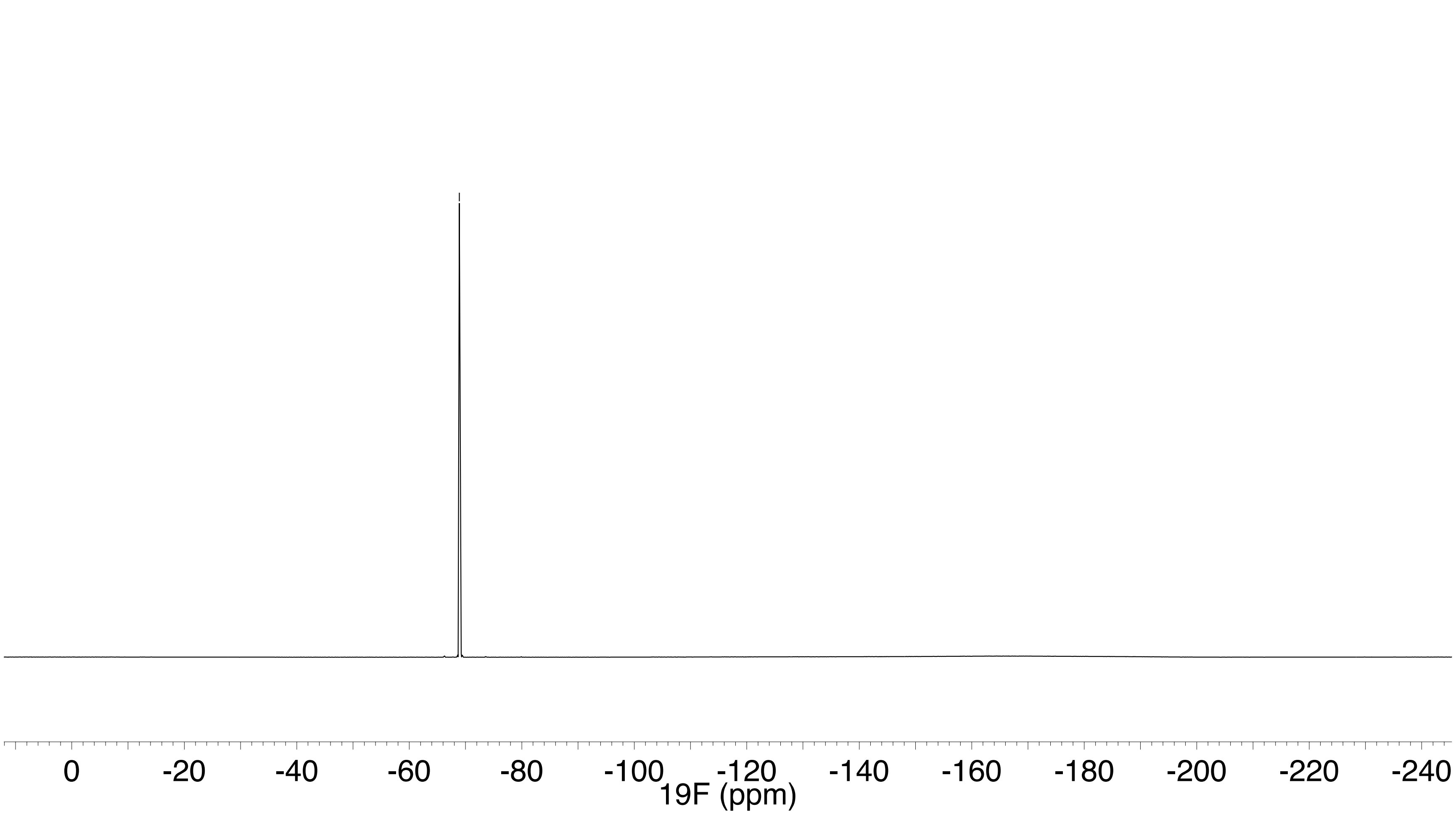

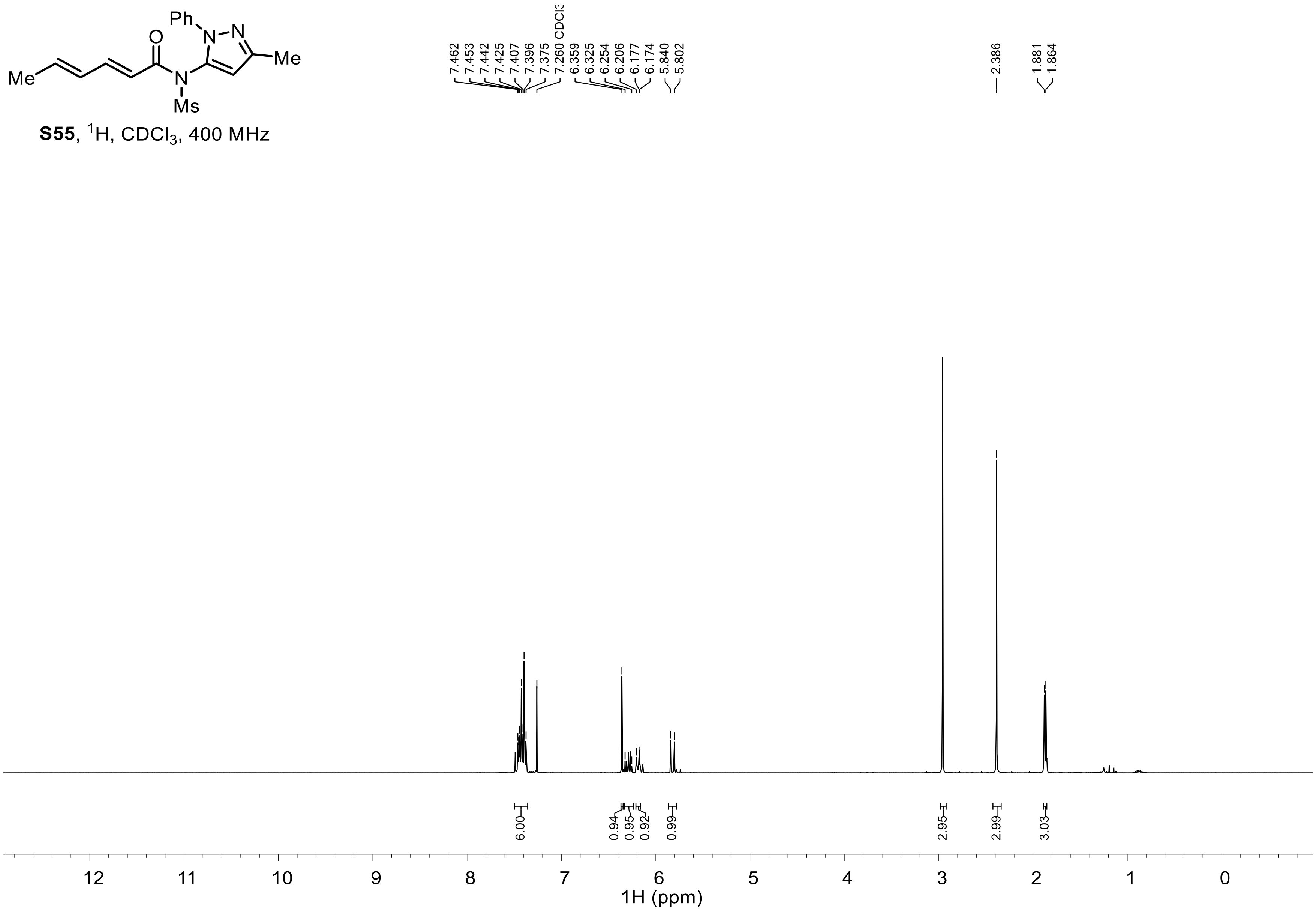

S55, ^{1}H, $CDCl_3$, 400 MHz

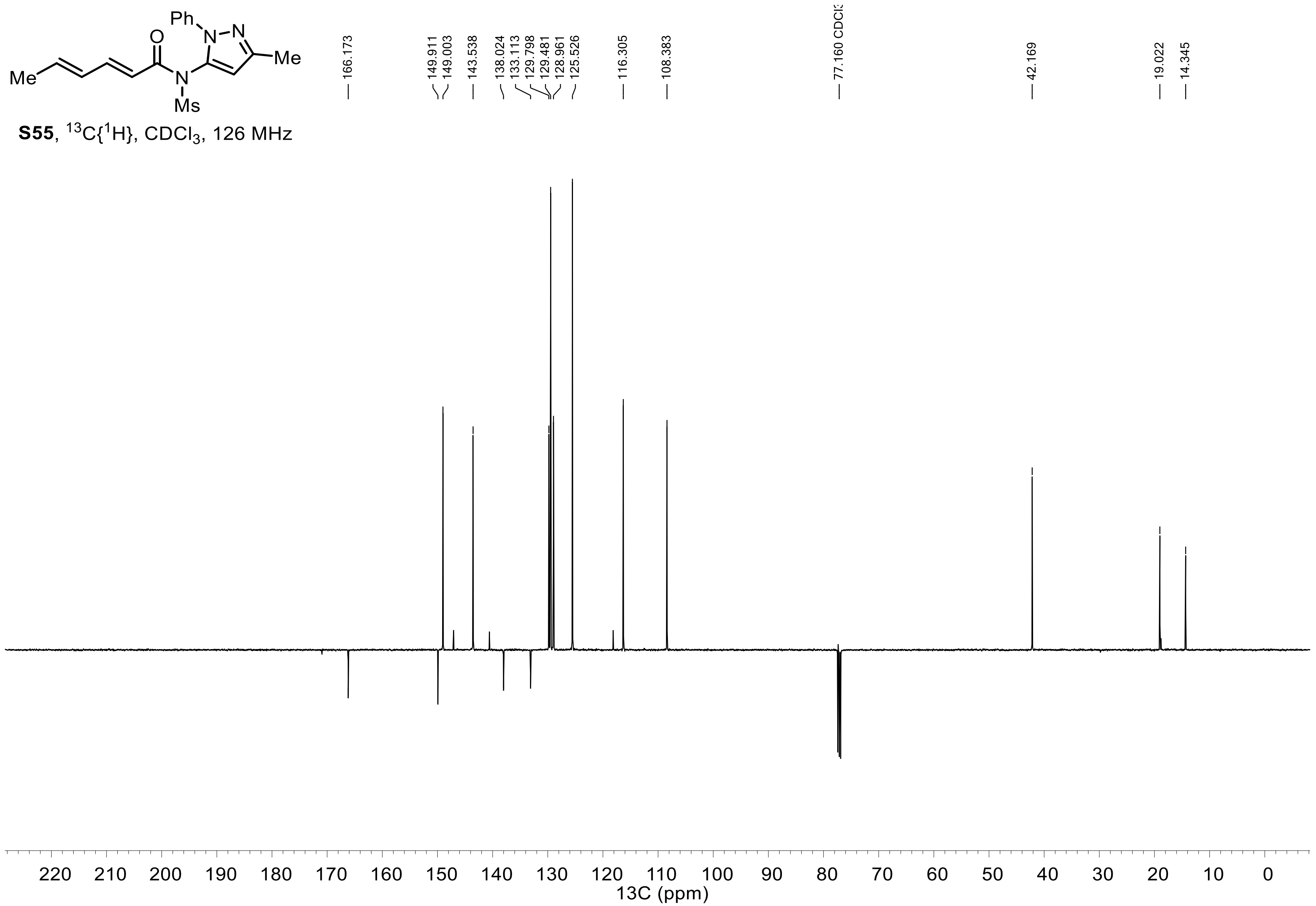
Ph
N
N
O
Me
Me
N
Ms
S55, 13C{1H}, CDCl3, 126 MHz
166.173
149.911
149.003
143.538
138.024
133.113
129.798
129.481
128.961
125.526
116.305
108.383
77.160 CDCl3
42.169
19.022
14.345
220 210 200 190 180 170 160 150 140 130 120 110 100 90 80 70 60 50 40 30 20 10 0
13C (ppm)

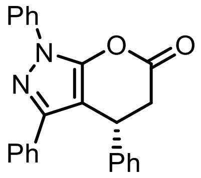

44, ^{1}H, $CDCl_3$, 300 MHz

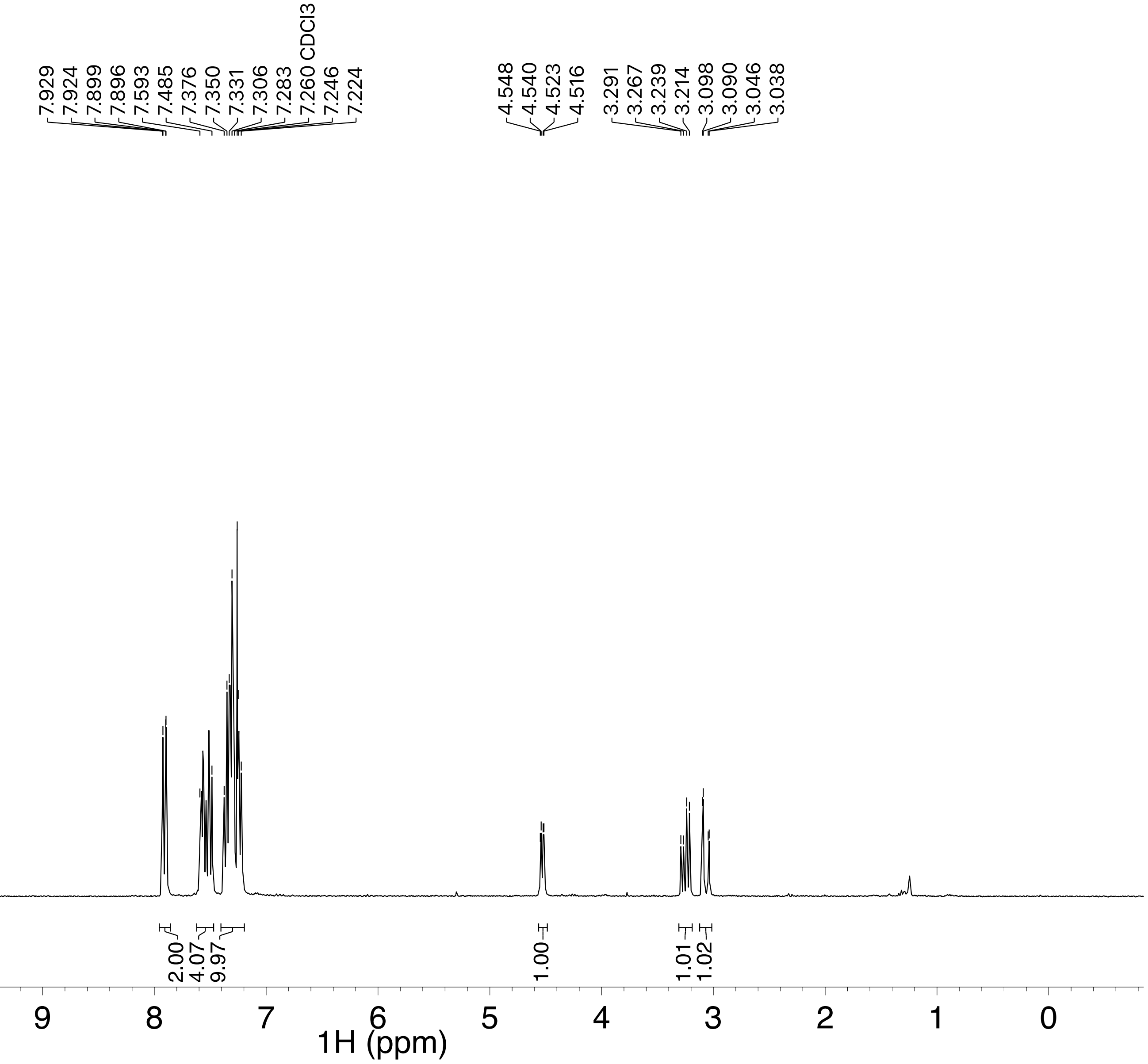

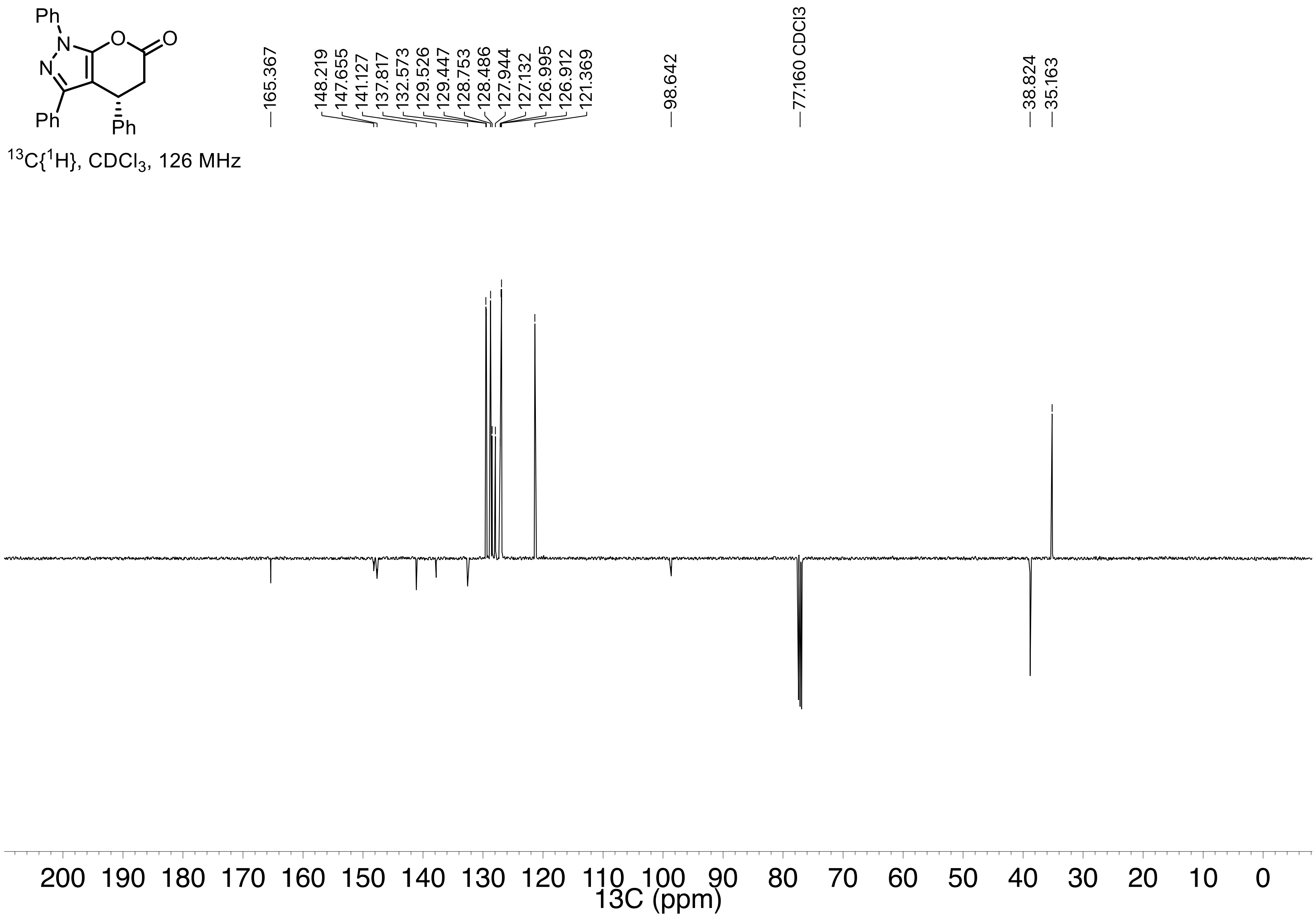

44, $^{13}C\{^{1}H\}$, $CDCl_3$, 126 MHz

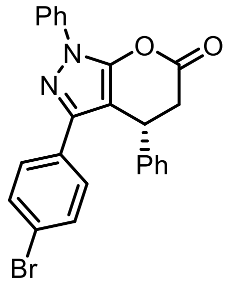

47, ^{1}H, $CDCl_3$, 400 MHz

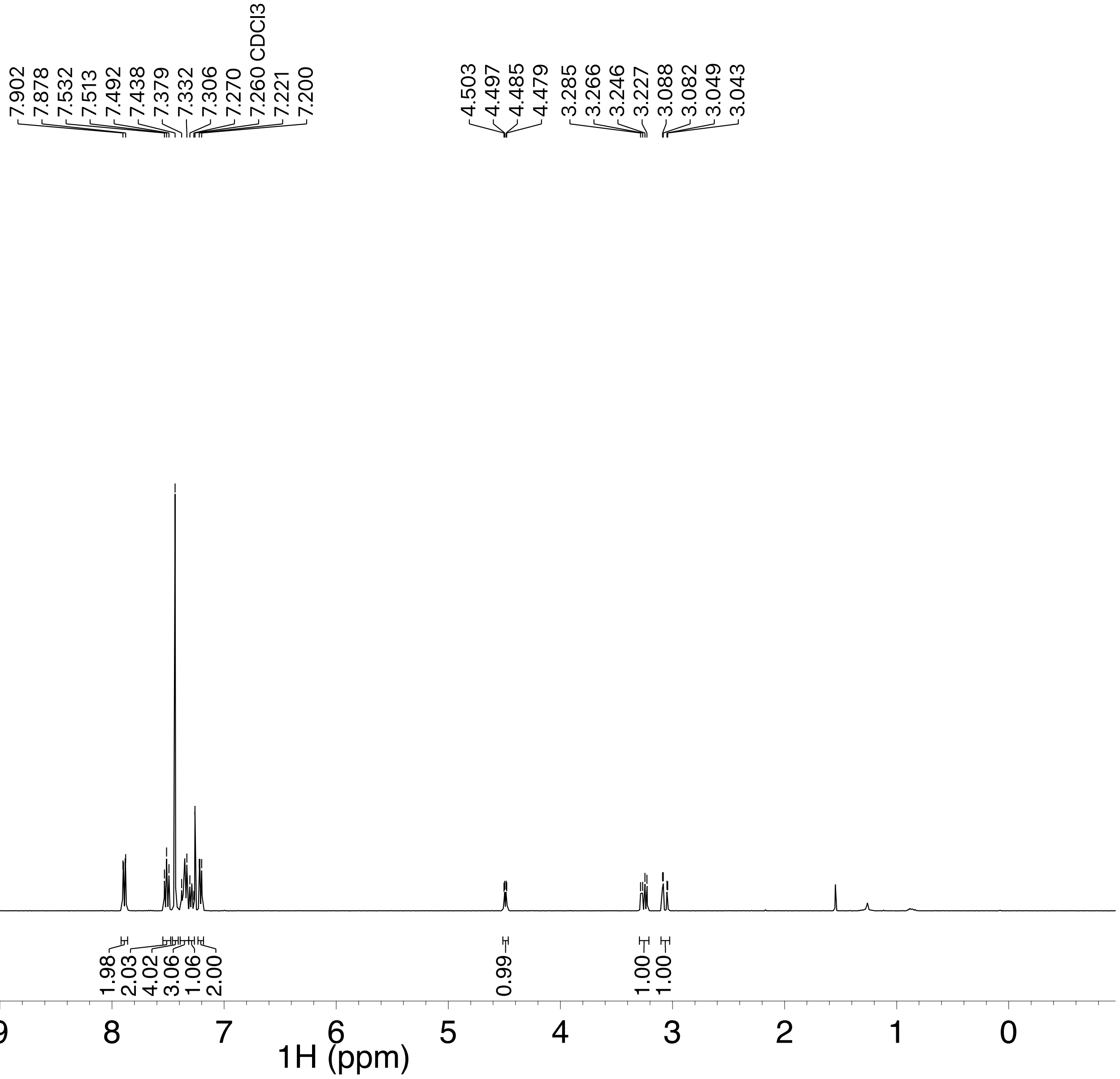

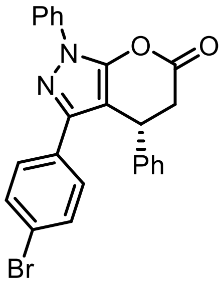

47, $^{13}C\{^{1}H\}$, $CDCl_3$, 126 MHz

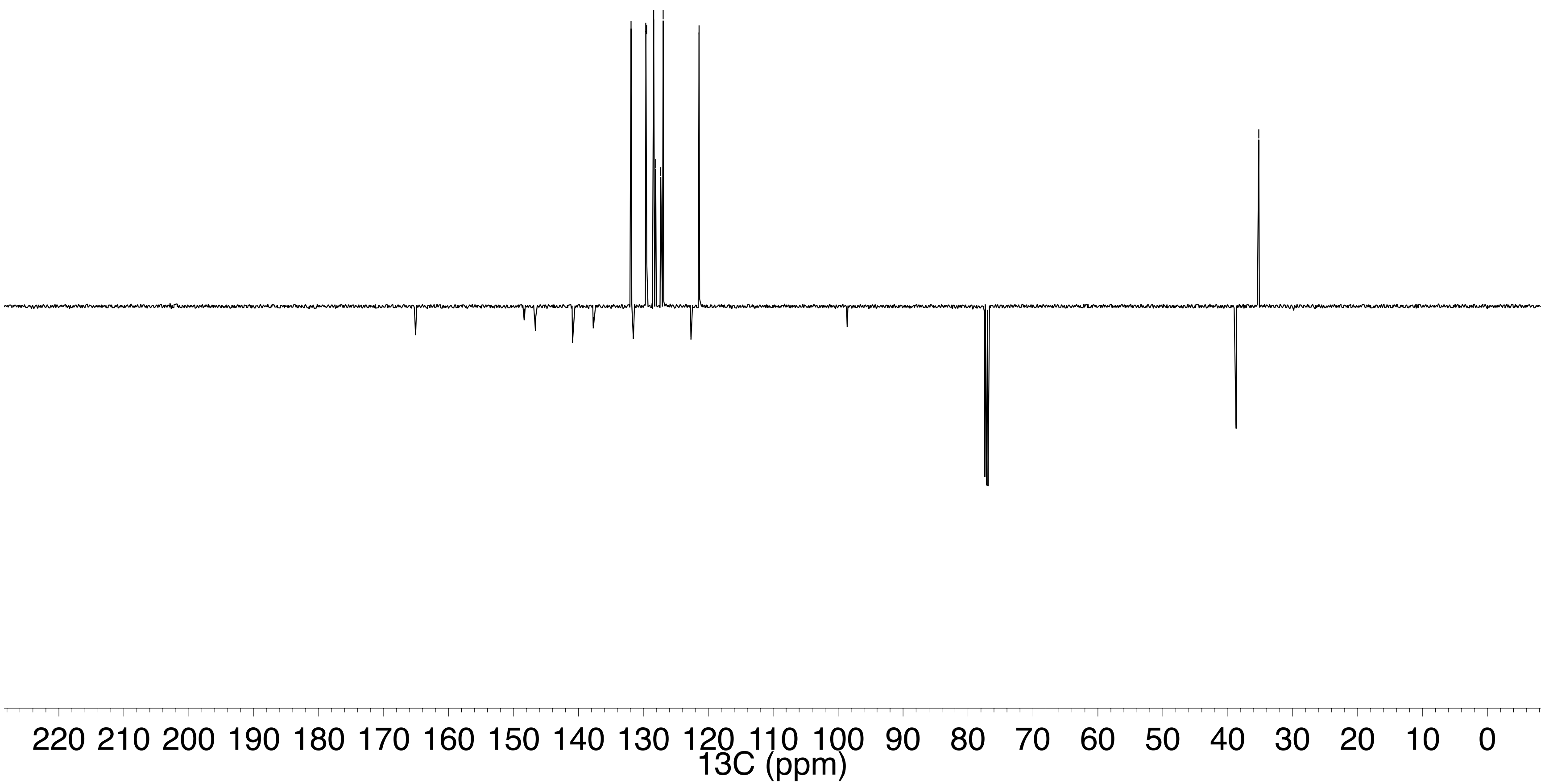

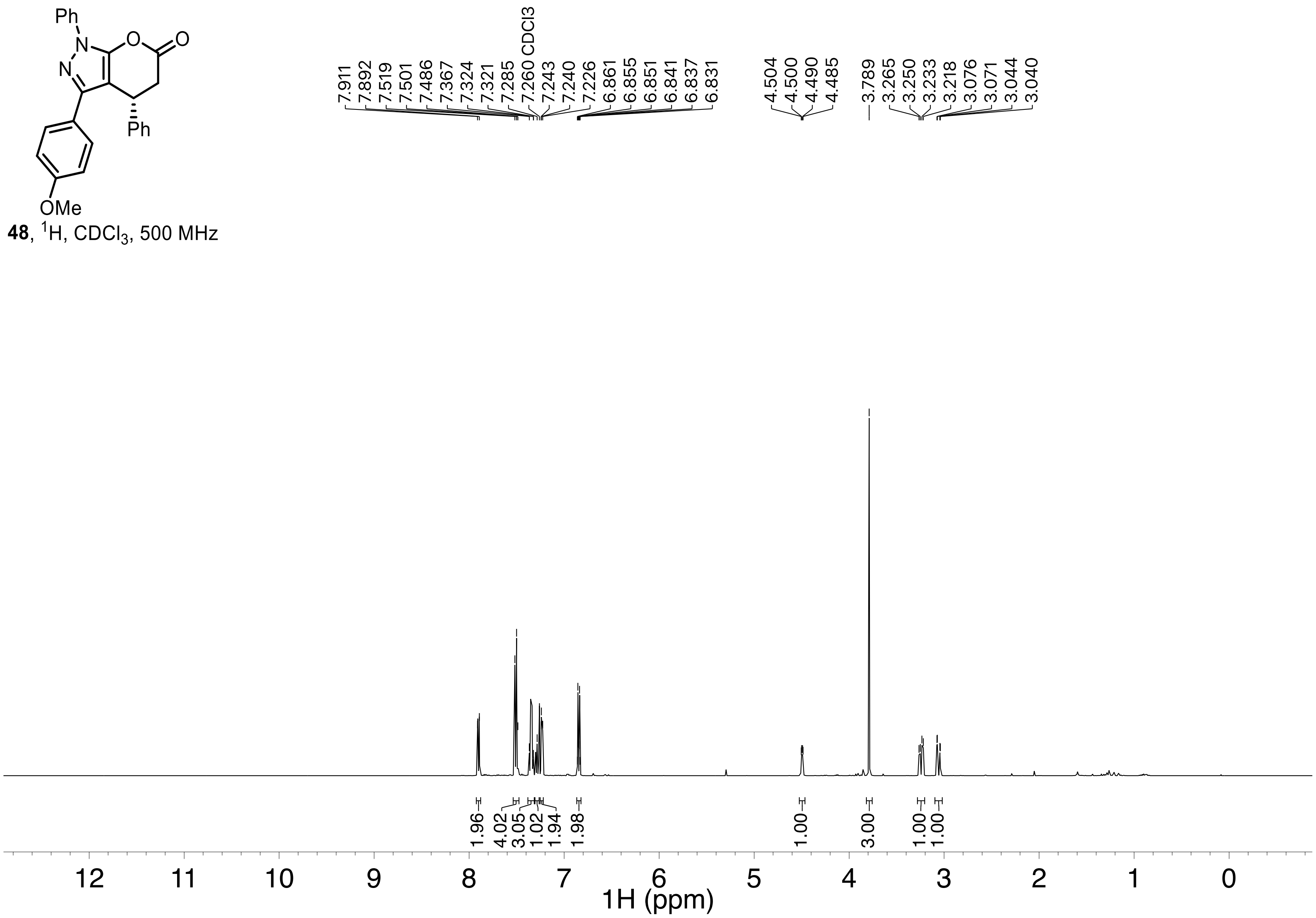

48, ^{1}H, $CDCl_3$, 500 MHz

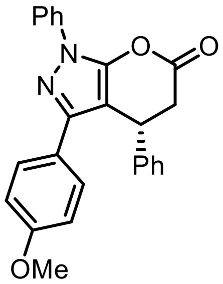

48, $^{13}C\{^{1}H\}$, $CDCl_3$, 126 MHz

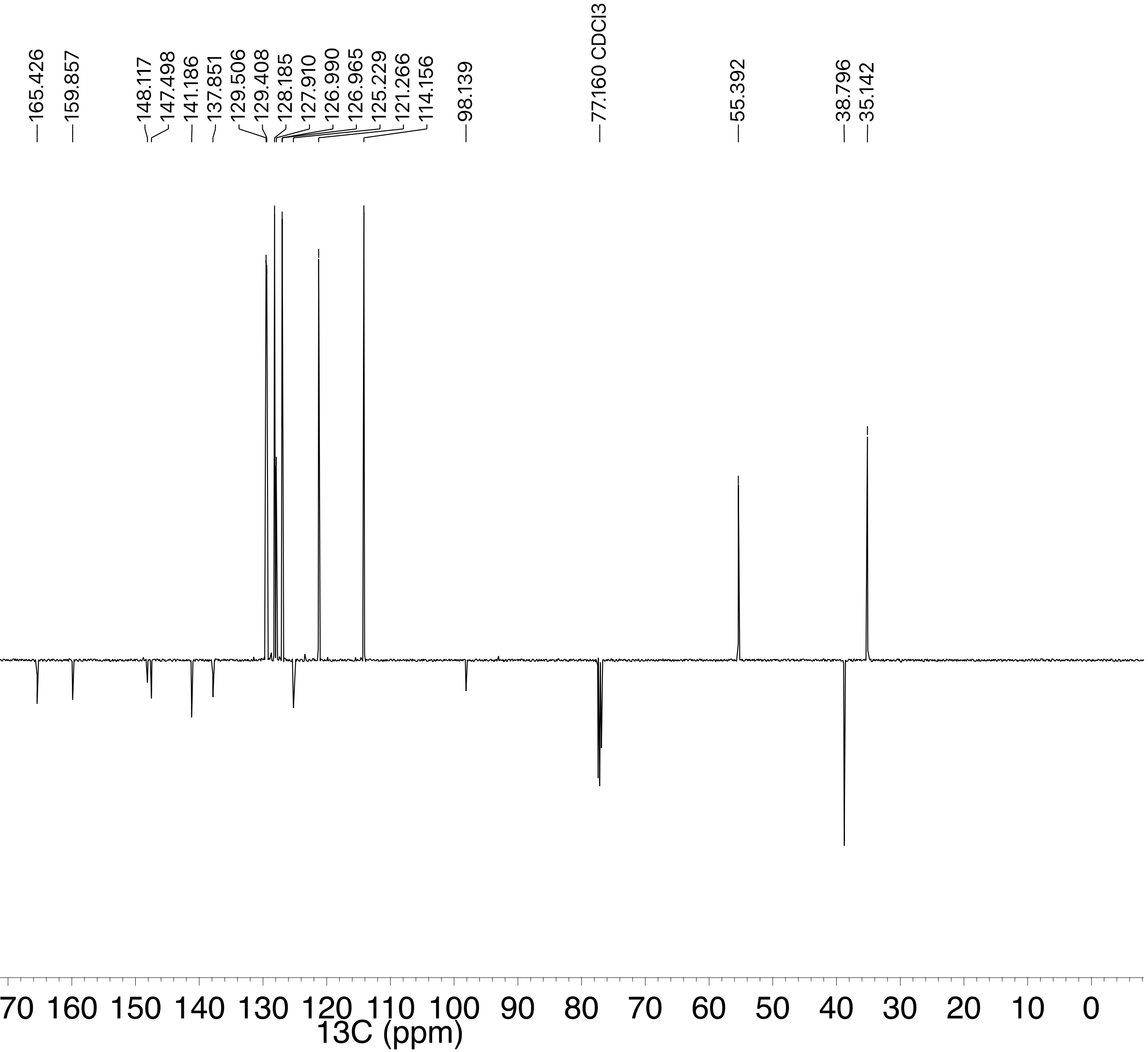

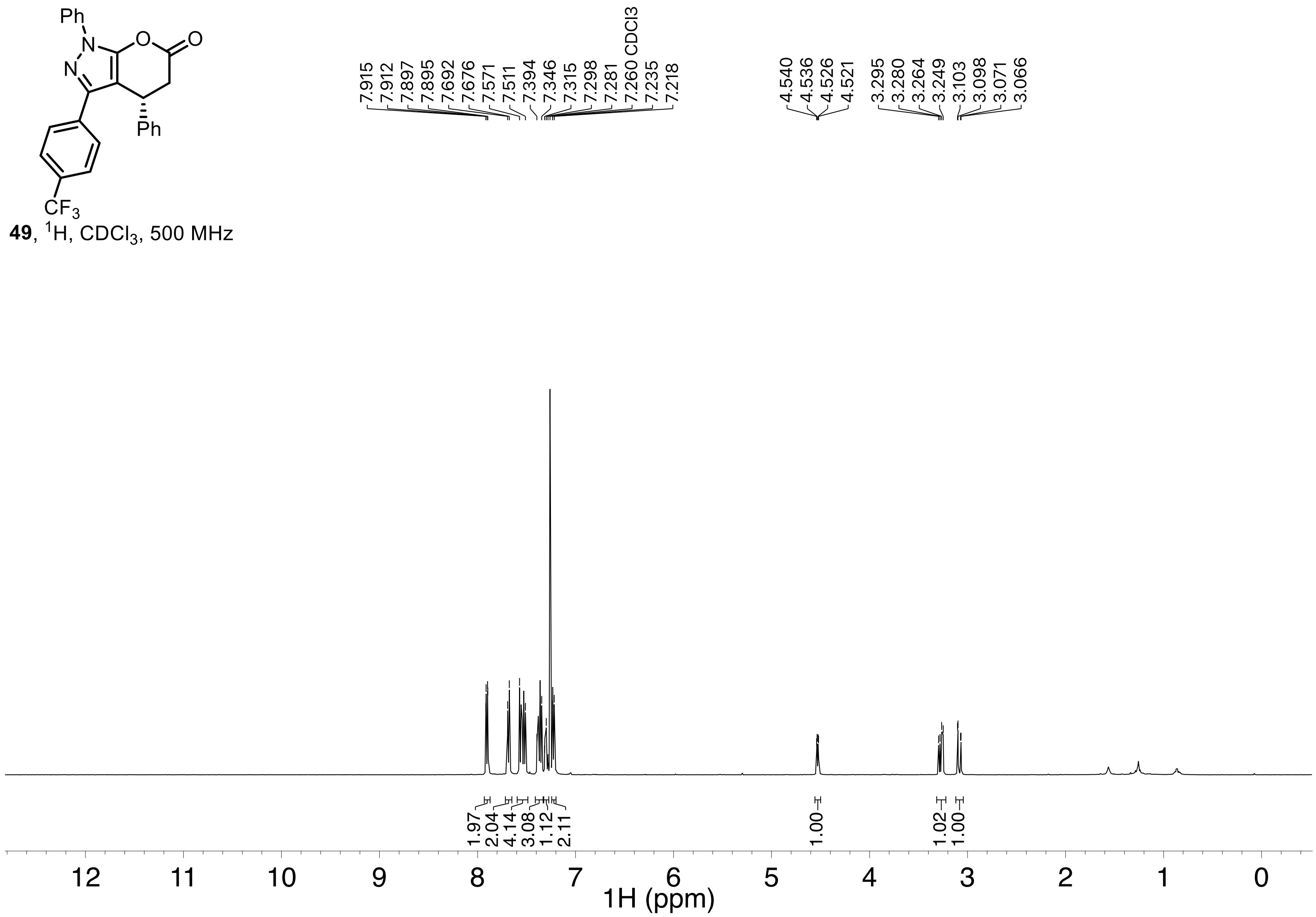

49, ^{1}H, $CDCl_3$, 500 MHz

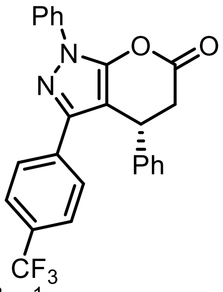

49, $^{13}C\{^1H\}$, $CDCl_3$, 126 MHz

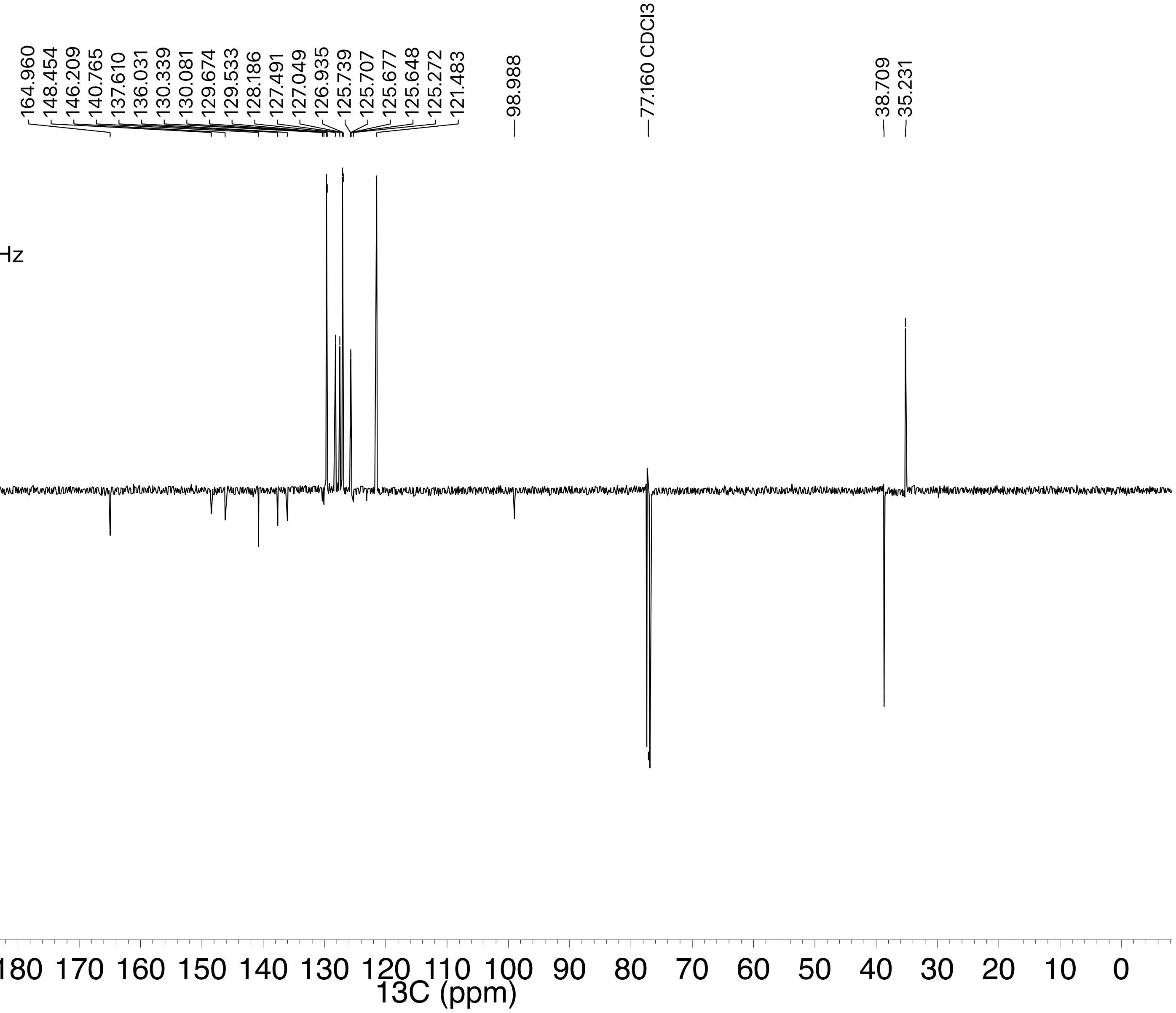

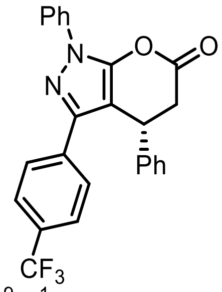

49, $^{19}F\{^{1}H\}$, $CDCl_3$, 470 MHz

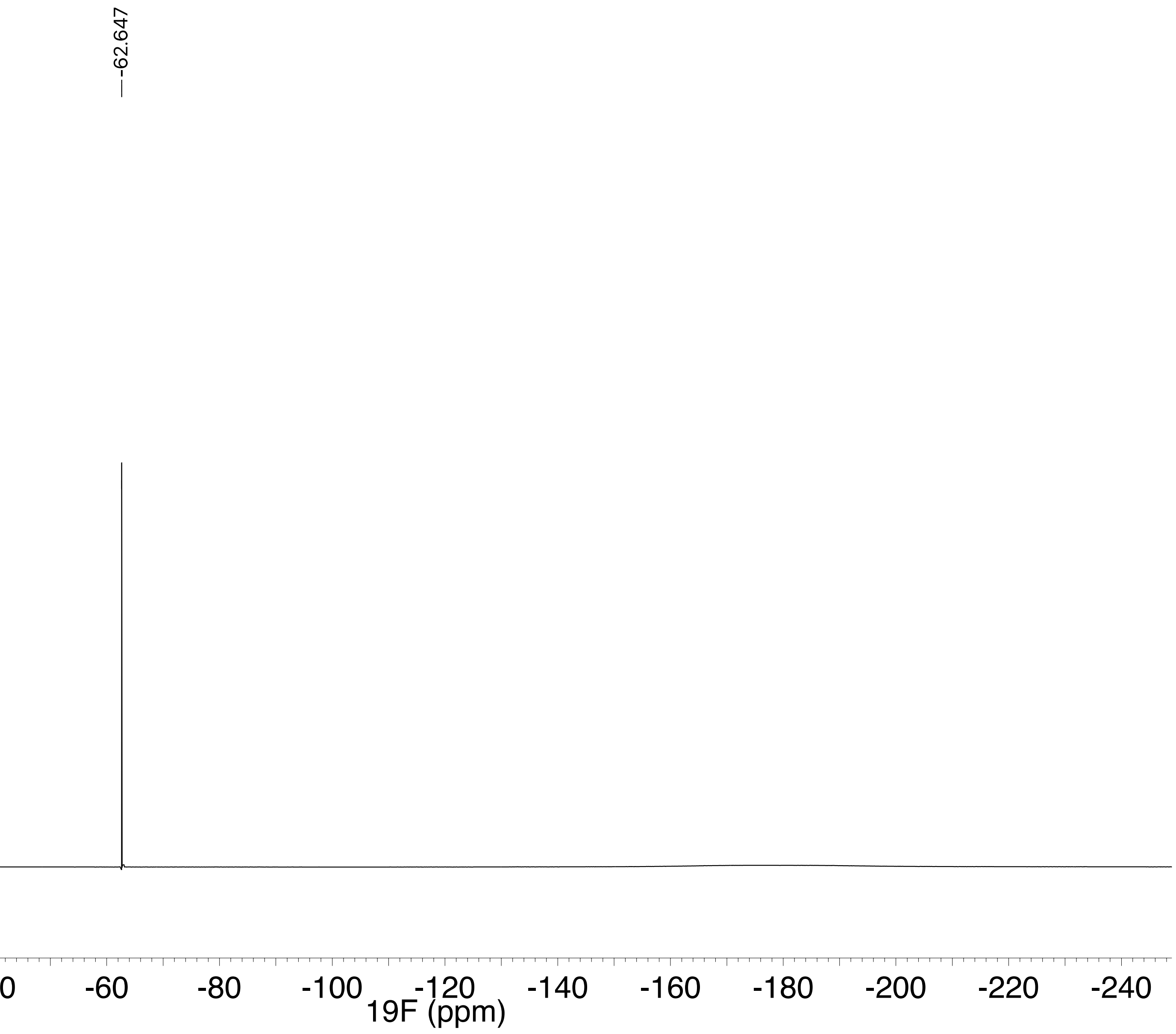

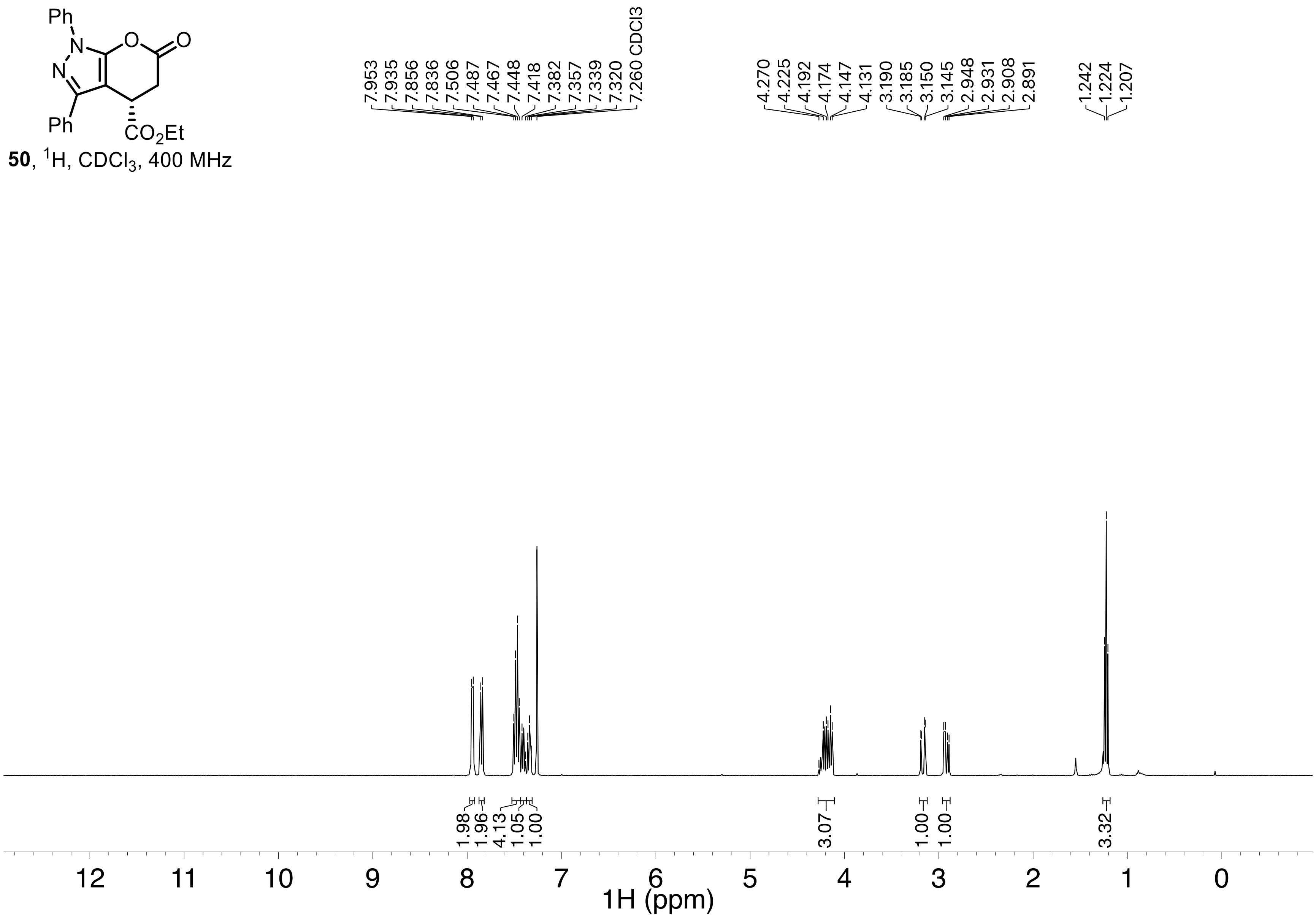

50, ^{1}H, $CDCl_3$, 400 MHz

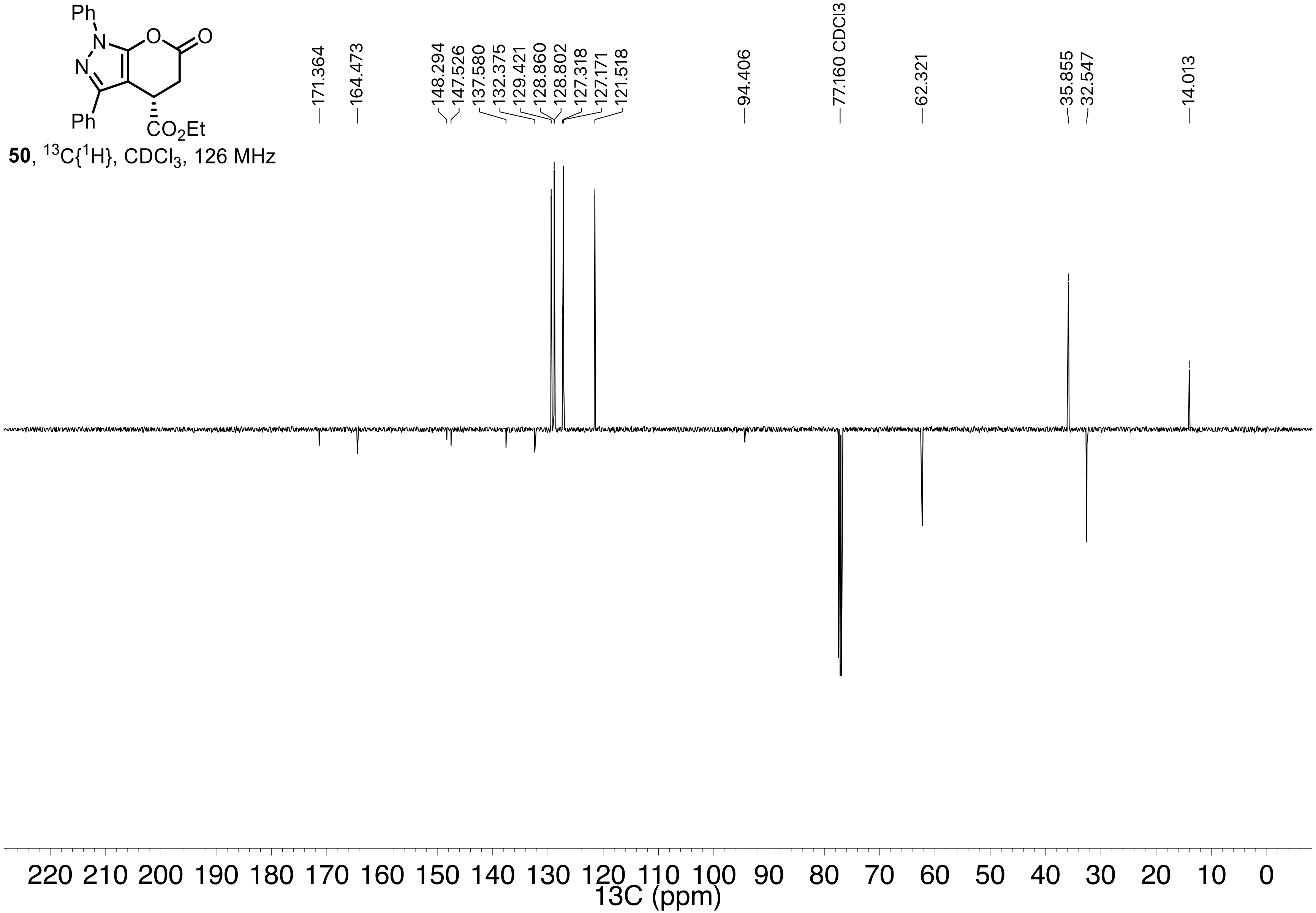

50, $^{13}C\{^{1}H\}$, $CDCl_3$, 126 MHz

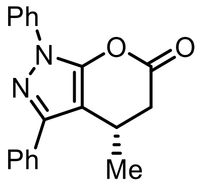

51, ^{1}H, $CDCl_3$, 400 MHz

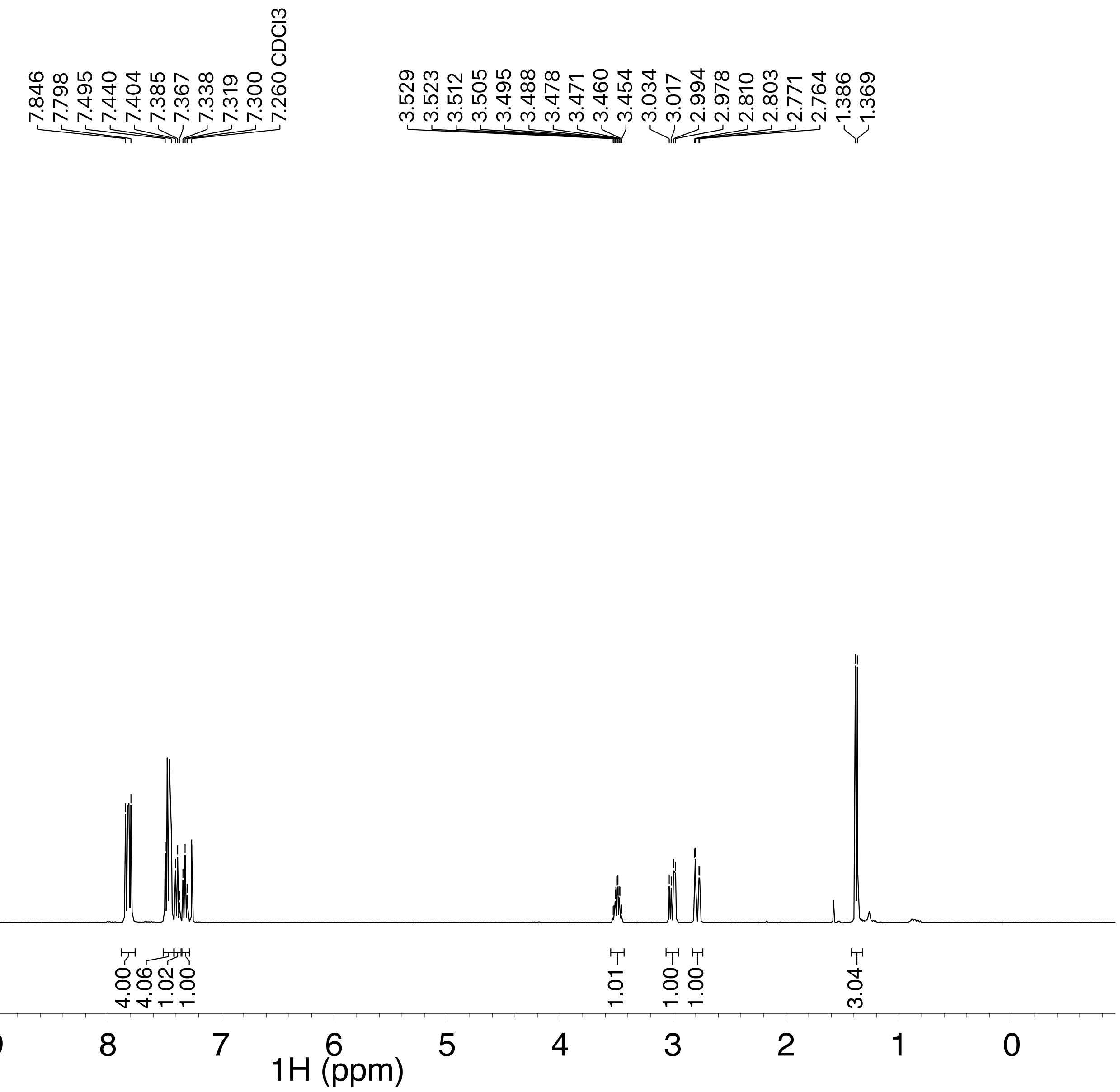

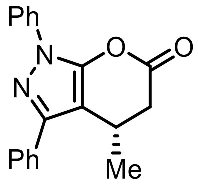

51, $^{13}C\{^{1}H\}$, $CDCl_3$, 126 MHz

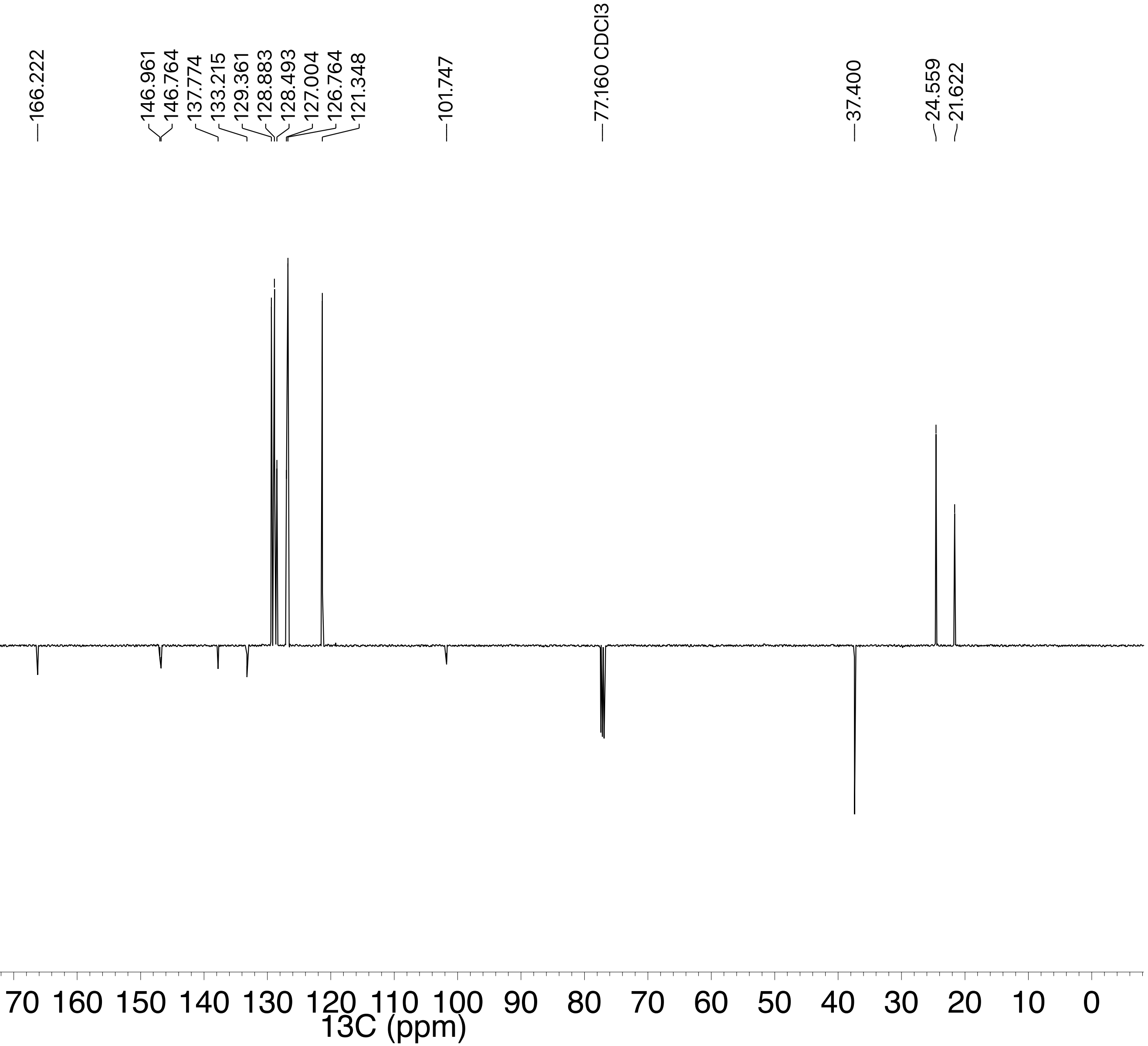

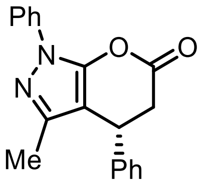

52, ^{1}H, $CDCl_3$, 400 MHz

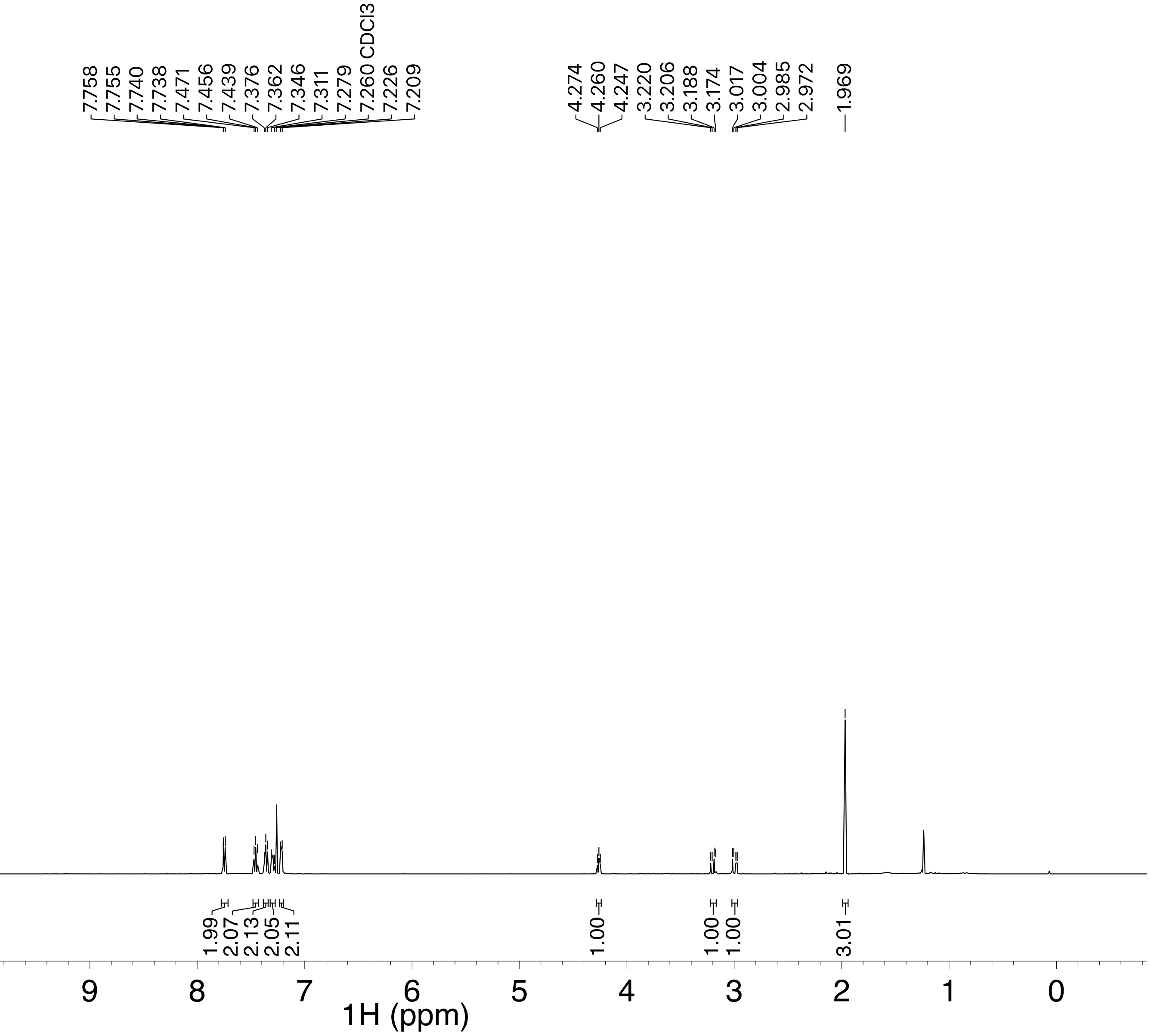

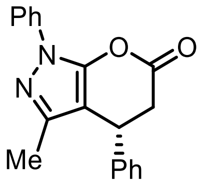

52, $^{13}C\{^{1}H\}$, $CDCl_3$, 126 MHz

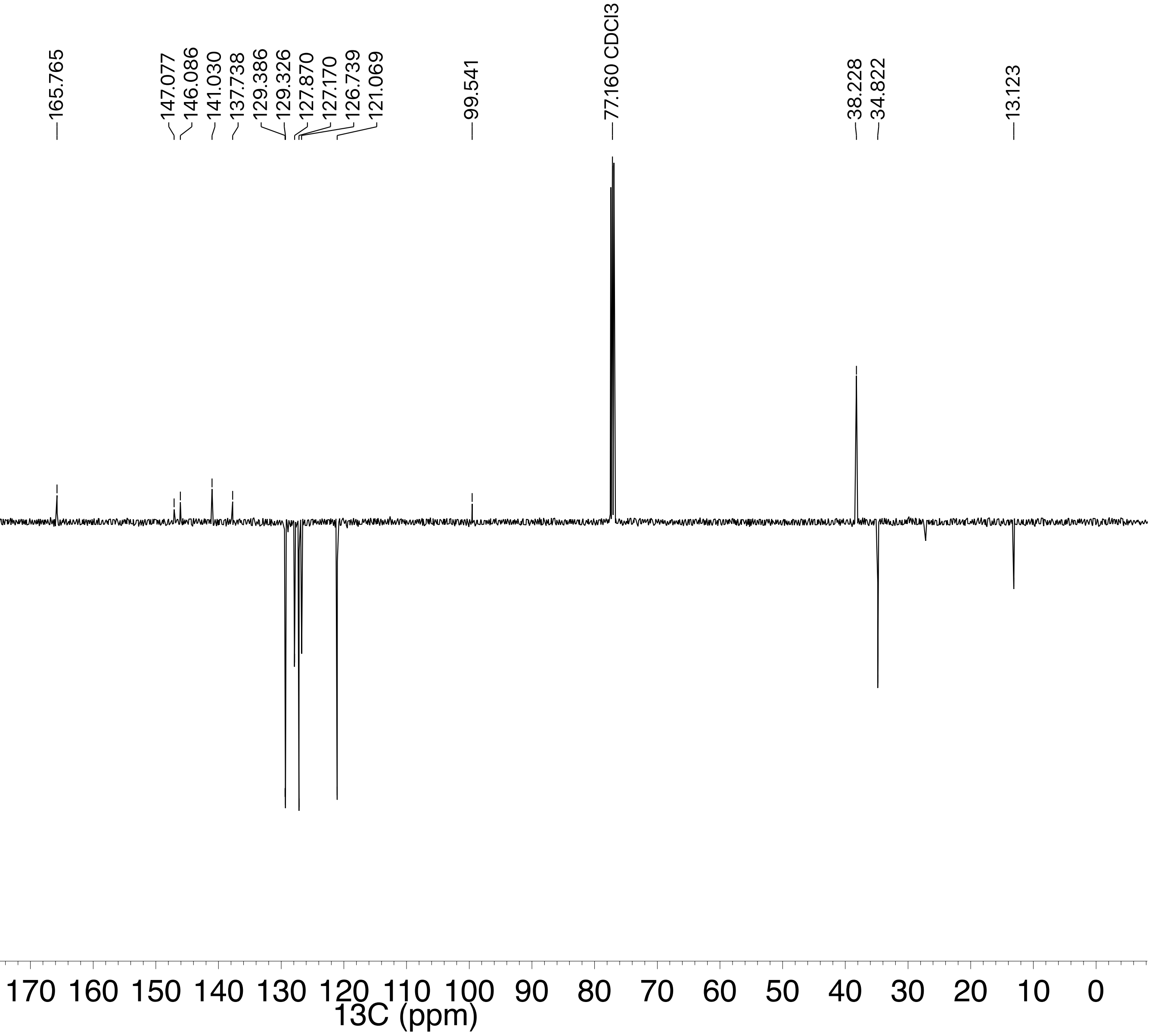

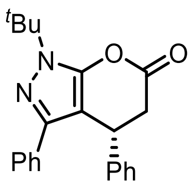

52, ^{1}H, $CDCl_3$, 500 MHz

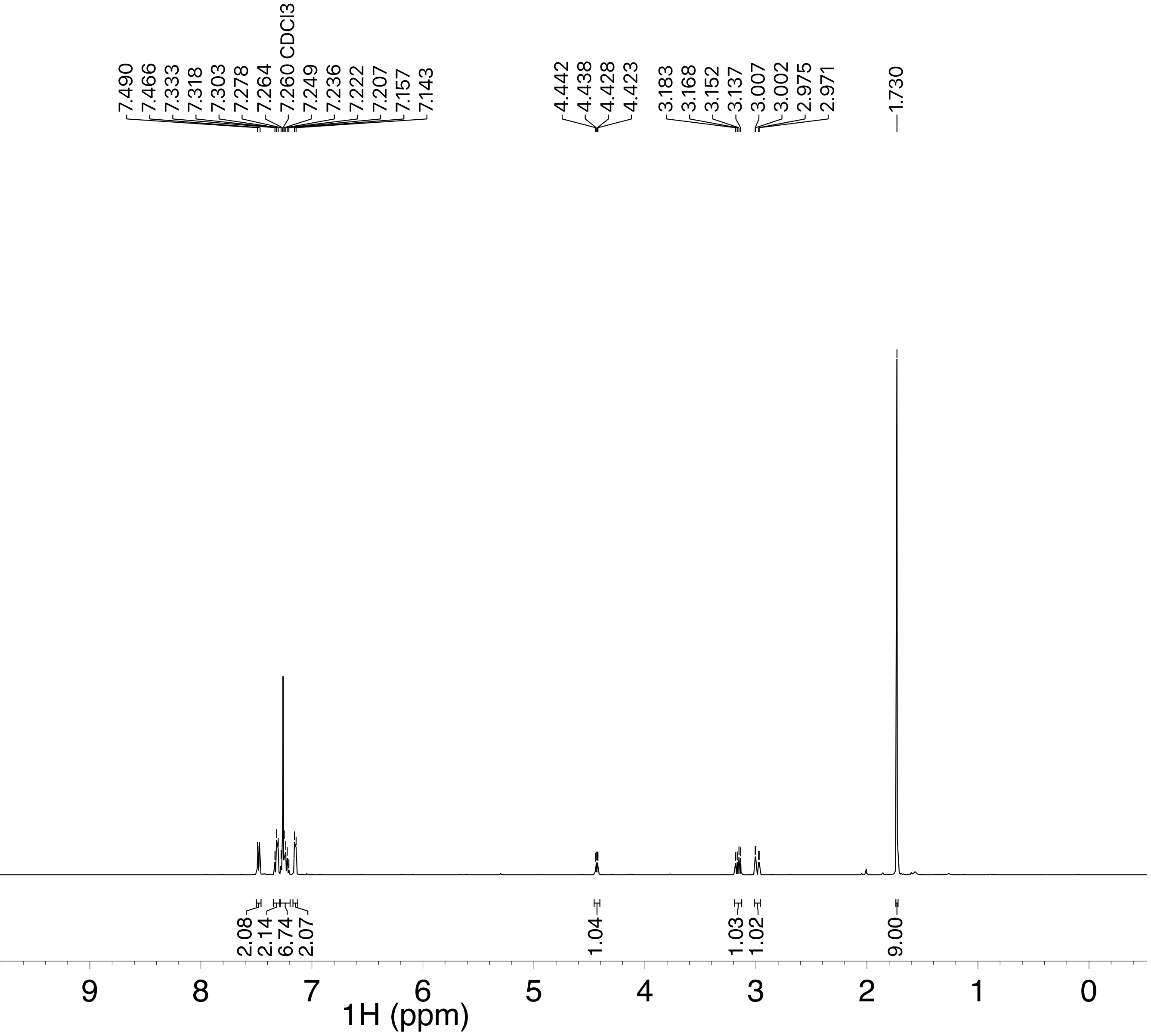

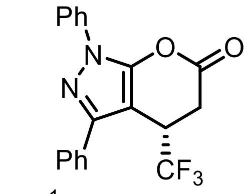

54, ^{1}H, $CDCl_3$, 500 MHz

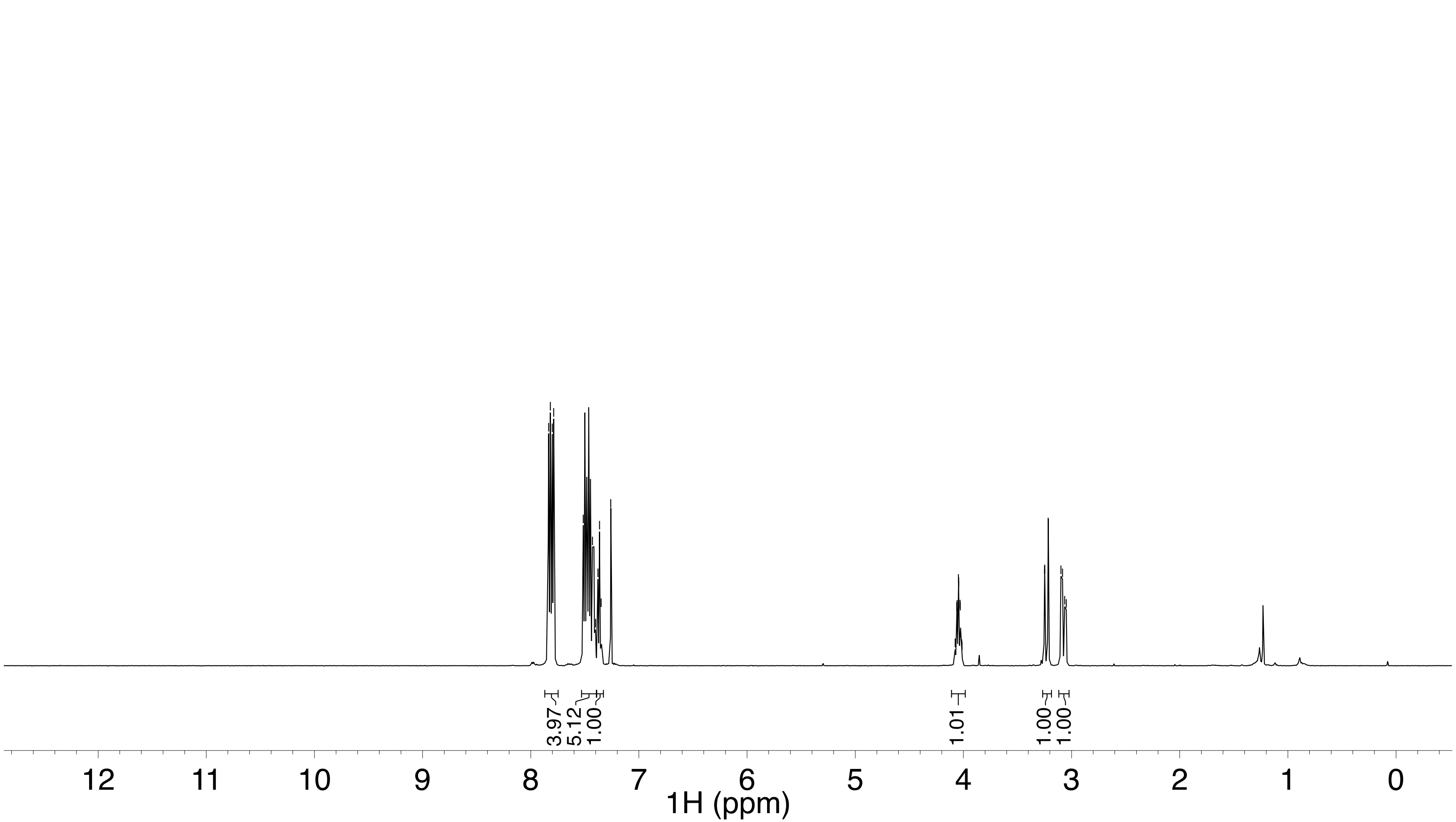

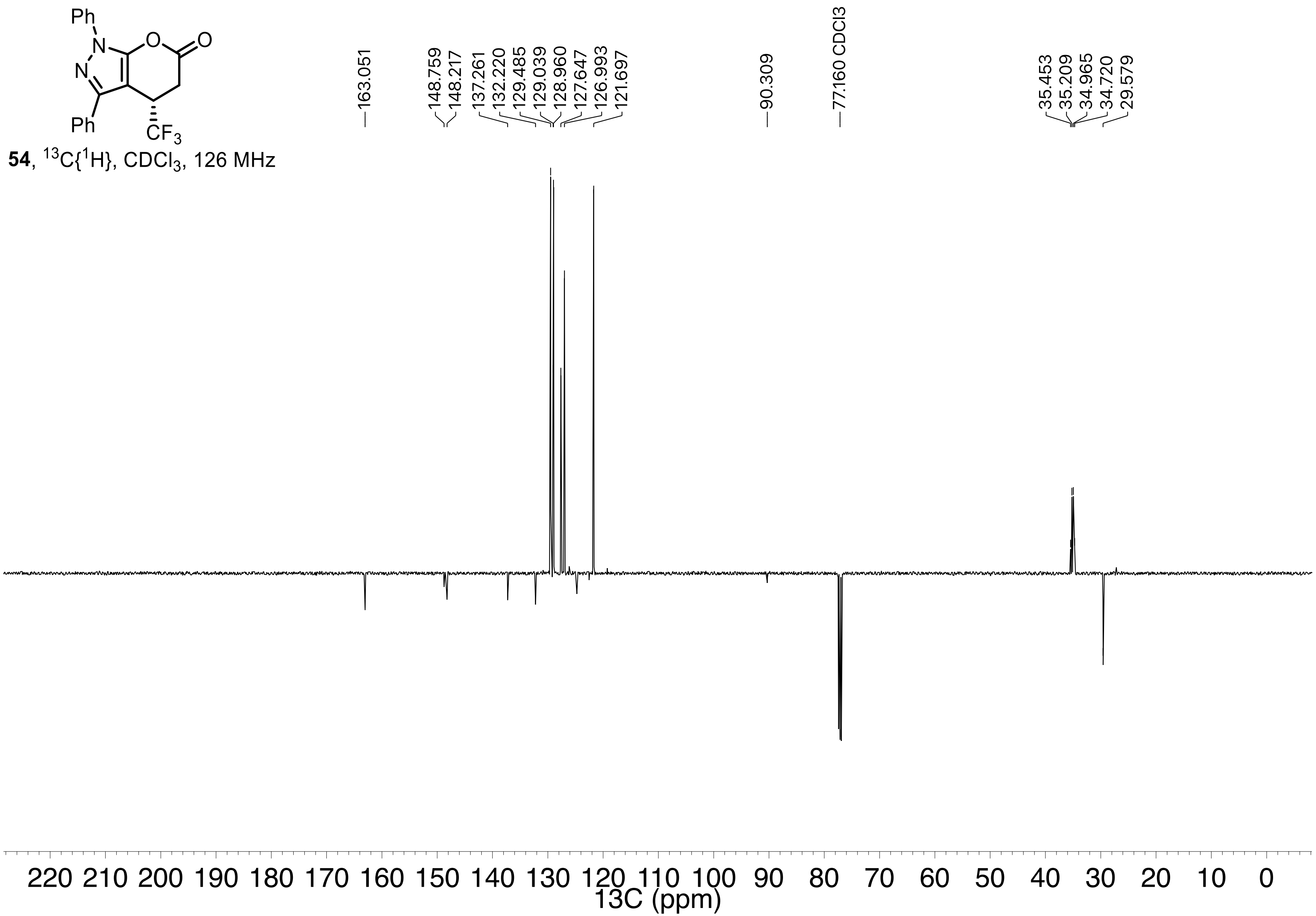

54, $^{13}C\{^{1}H\}$, $CDCl_3$, 126 MHz

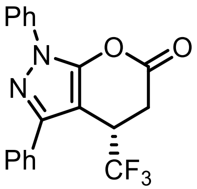

54, $^{19}F\{^1H\}$, $CDCl_3$, 377 MHz

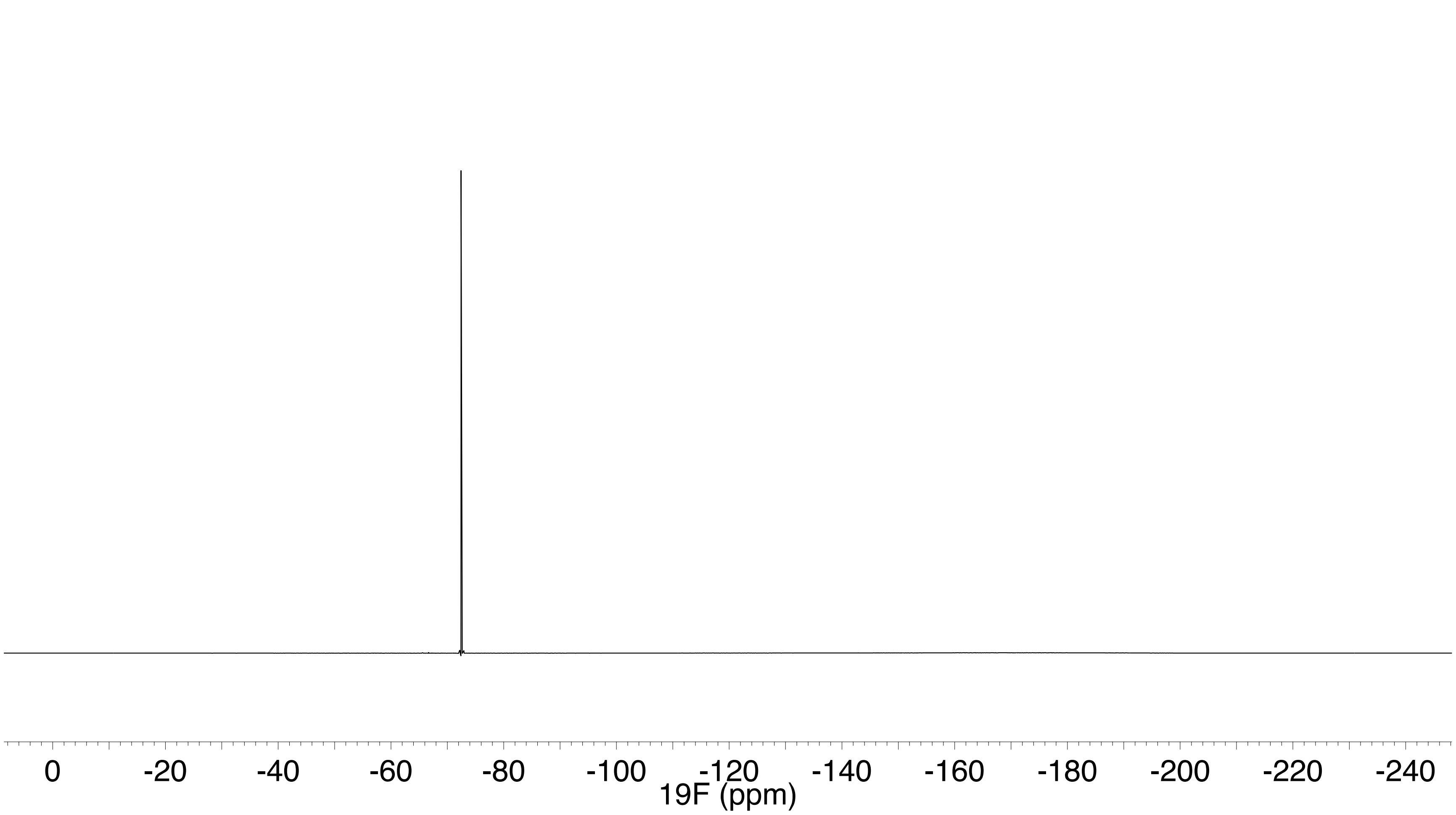

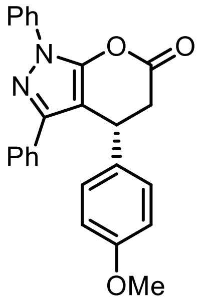

55, ^{1}H, $CDCl_3$, 500 MHz

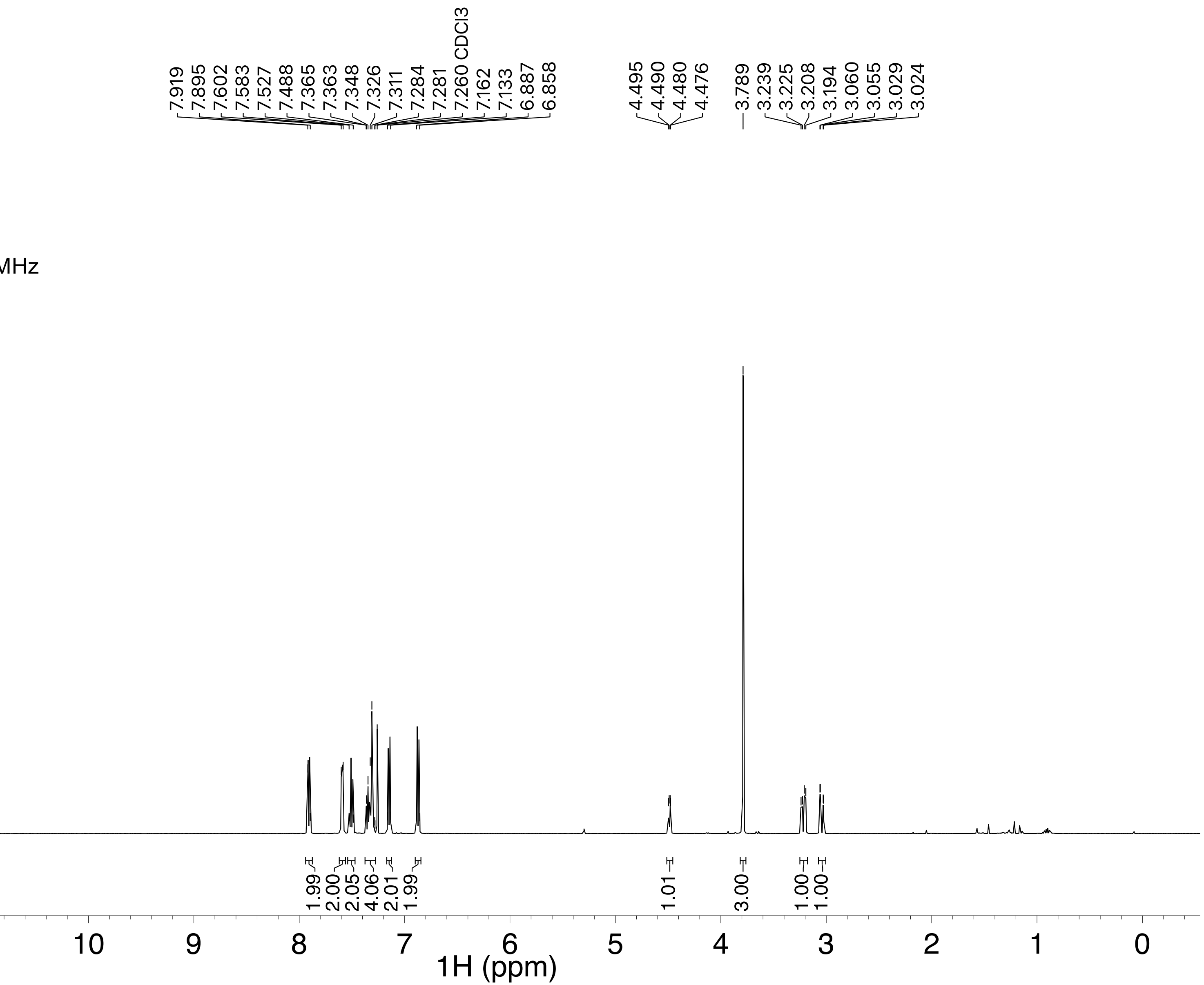

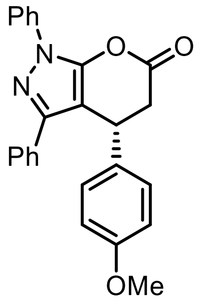

55, $^{13}C\{^{1}H\}$, $CDCl_3$, 126 MHz

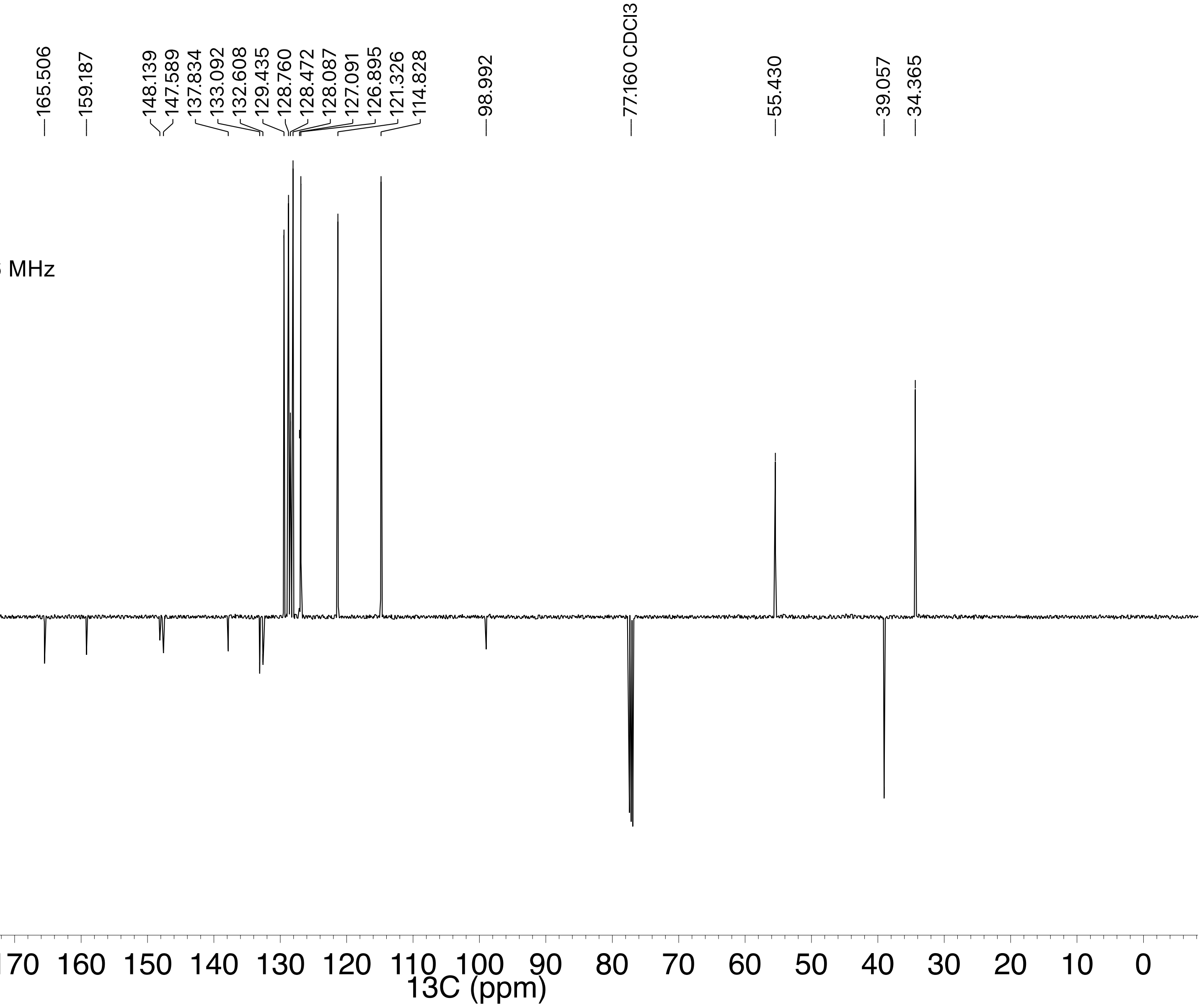

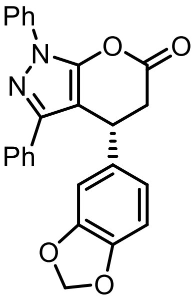

56, ^{1}H, $CDCl_3$, 500 MHz

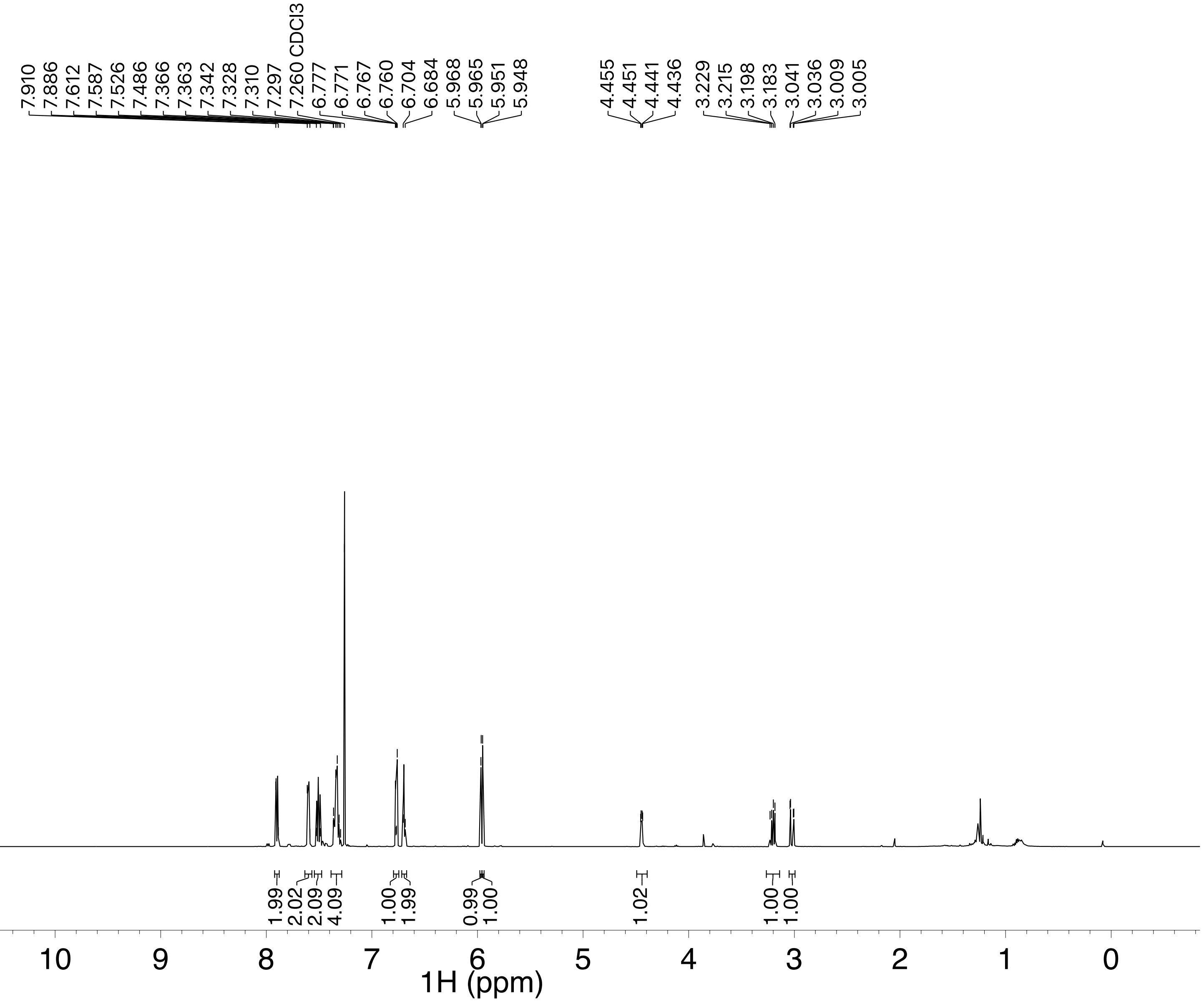

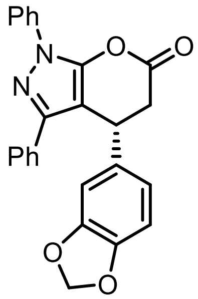

56, $^{13}C\{^{1}H\}$, $CDCl_3$, 126 MHz

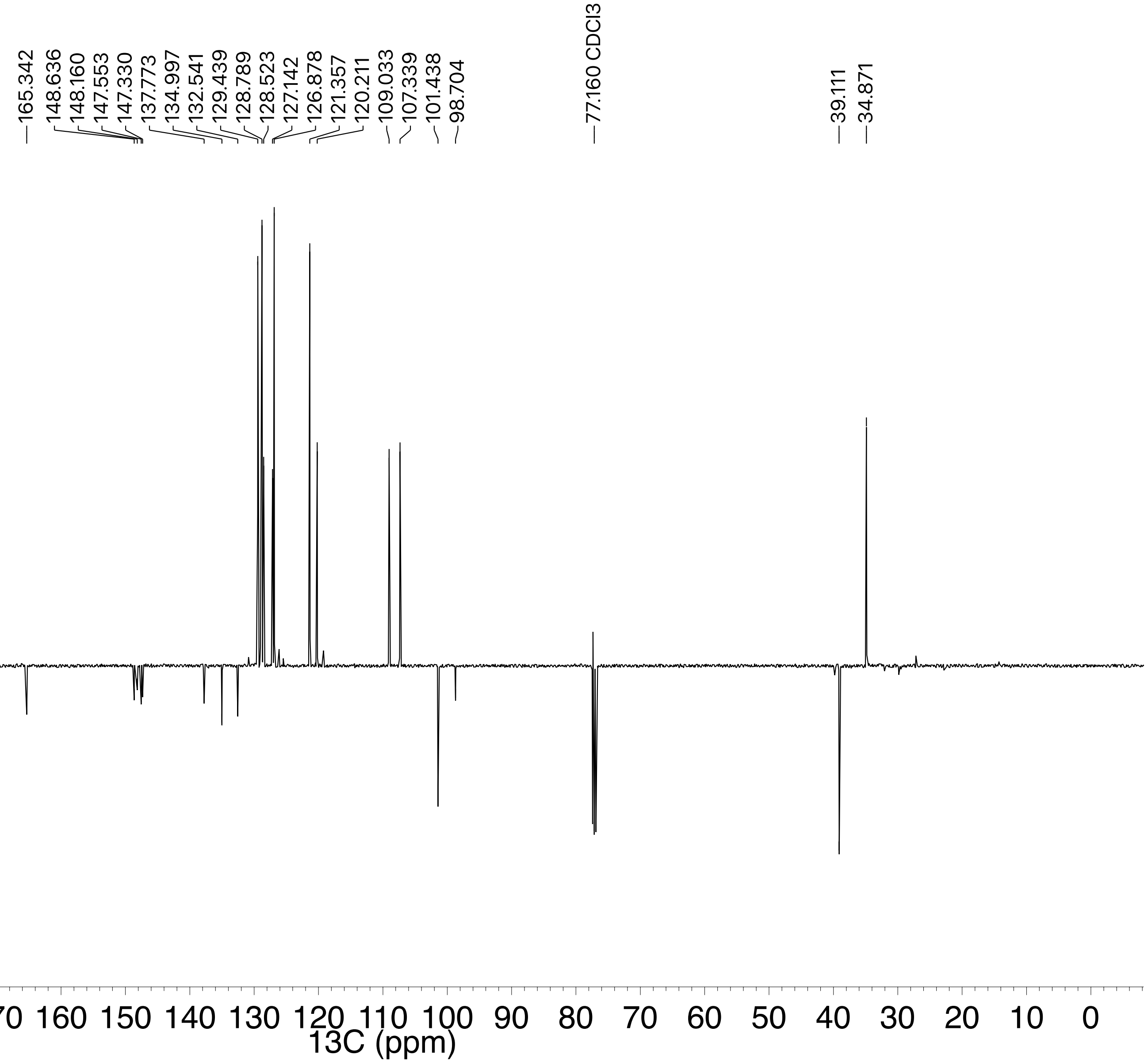

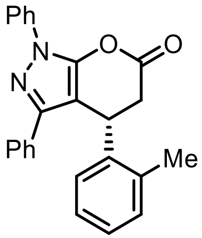

57, ^{1}H, $CDCl_3$, 500 MHz

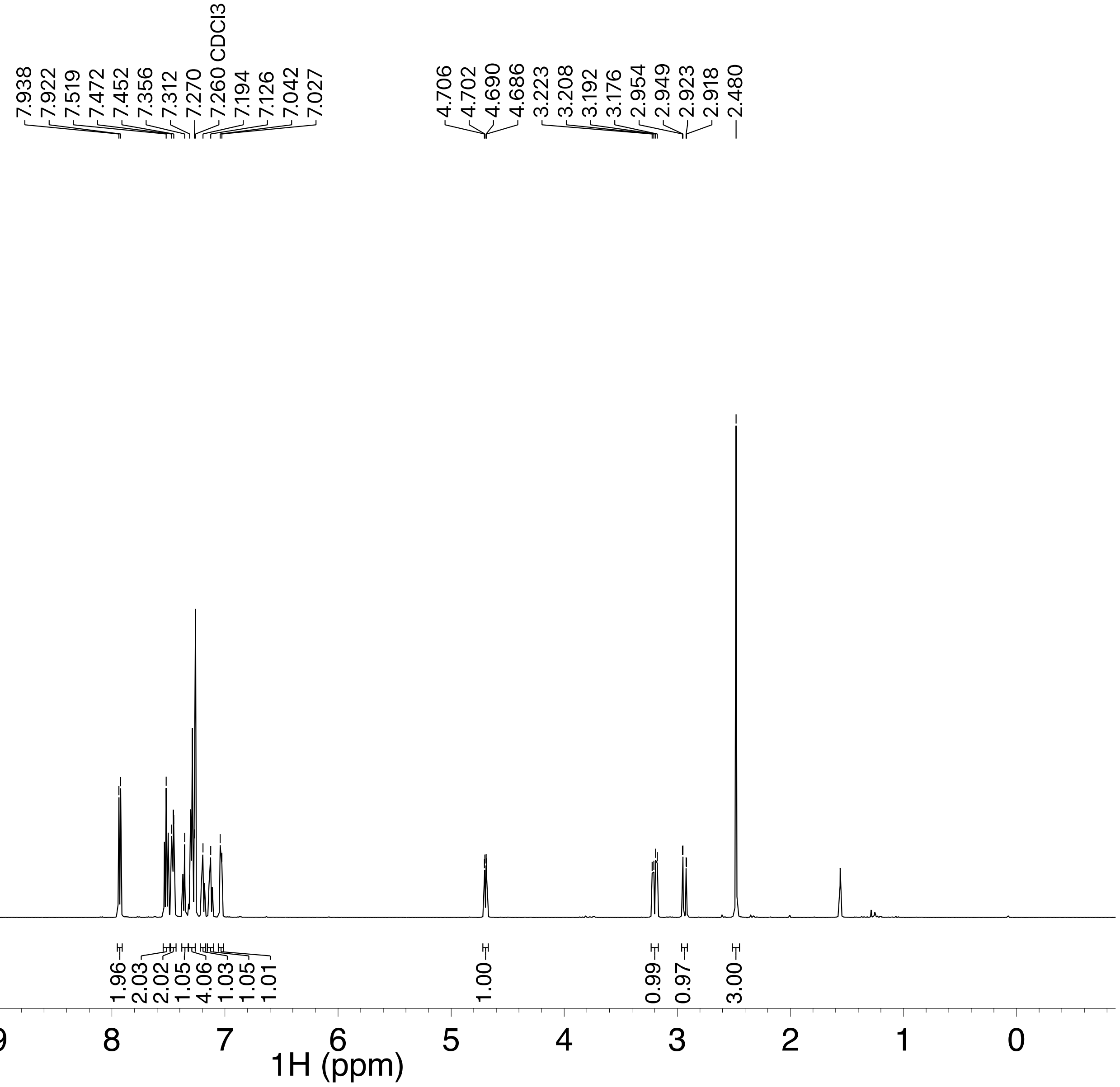

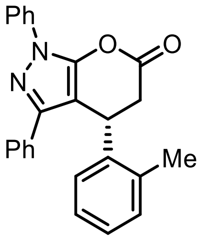

57, $^{13}C\{^1H\}$, $CDCl_3$, 126 MHz

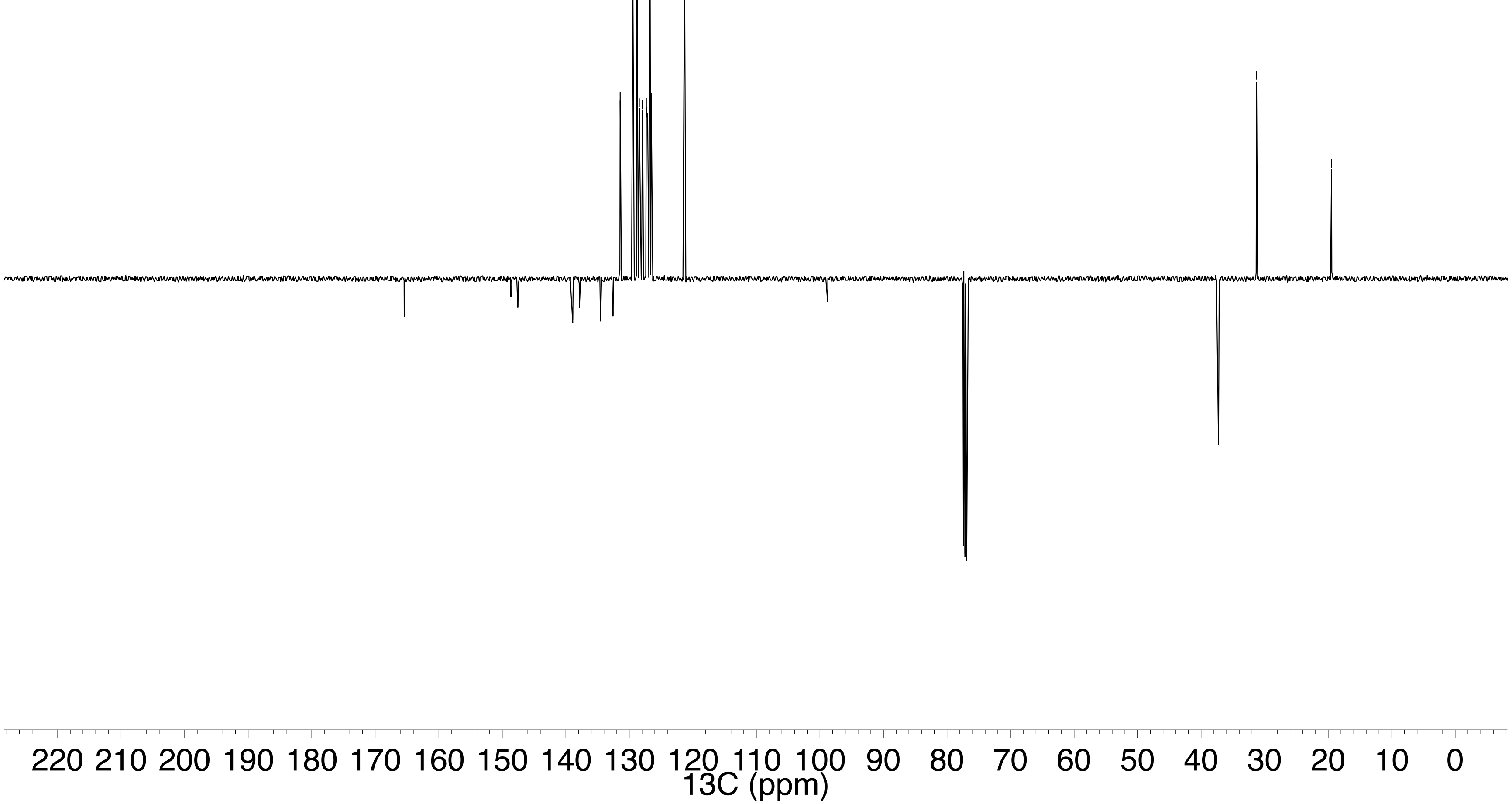

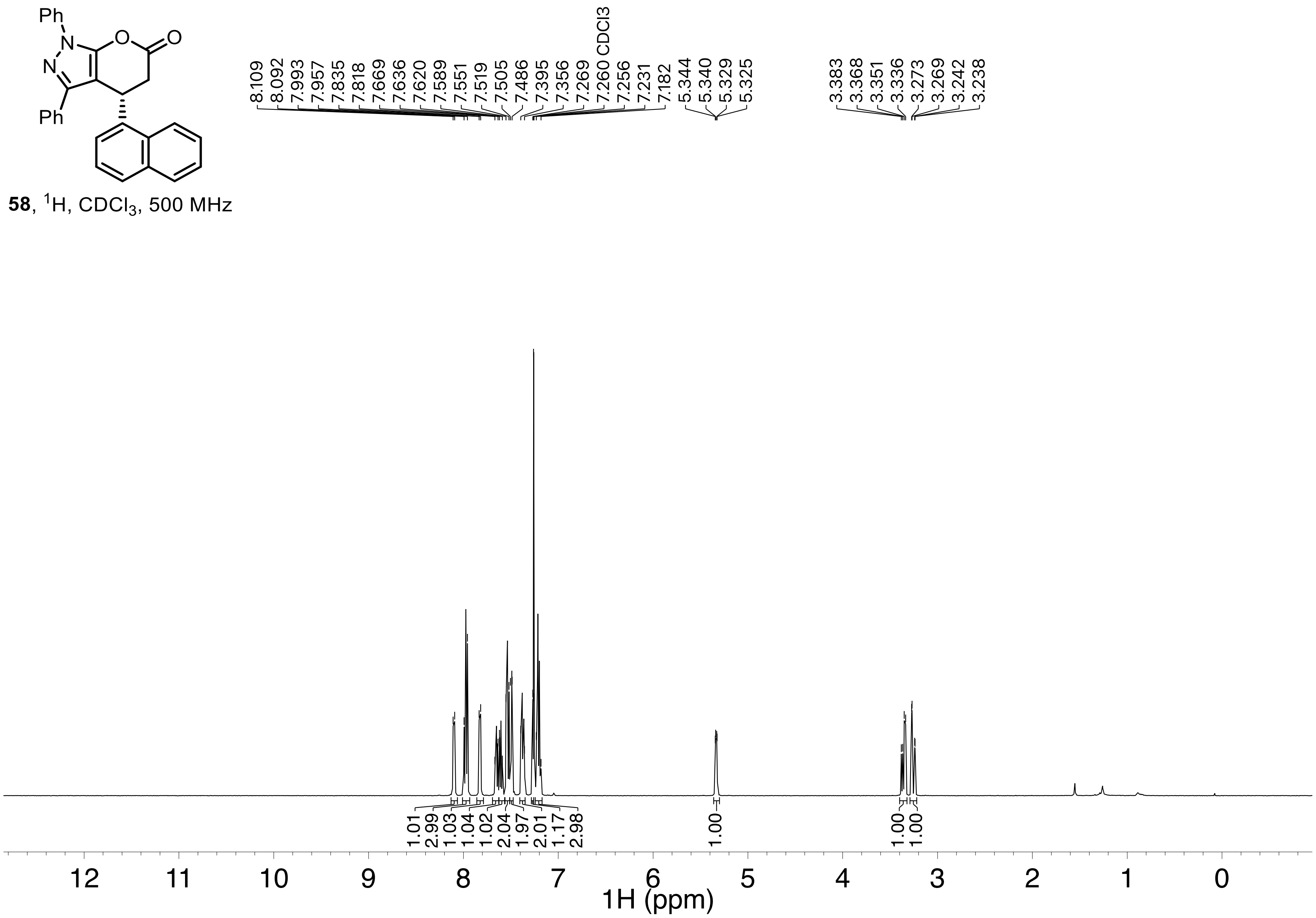

58, ^{1}H, $CDCl_3$, 500 MHz

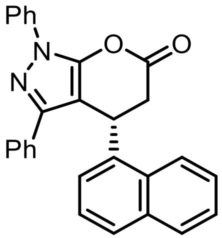

58, $^{13}C\{^1H\}$, $CDCl_3$, 126 MHz

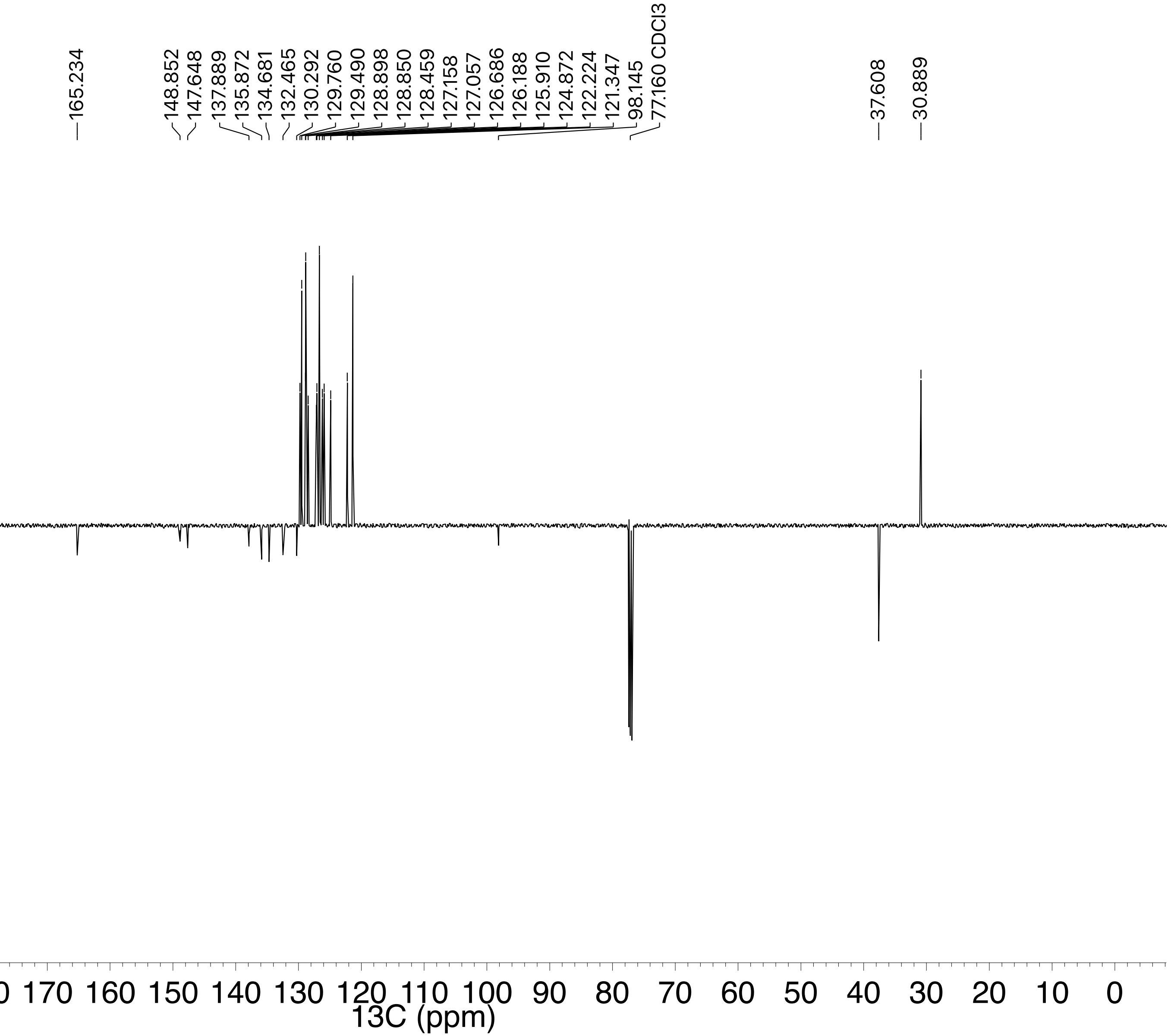

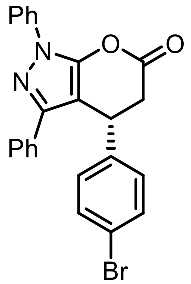

59, ^{1}H, $CDCl_3$, 300 MHz

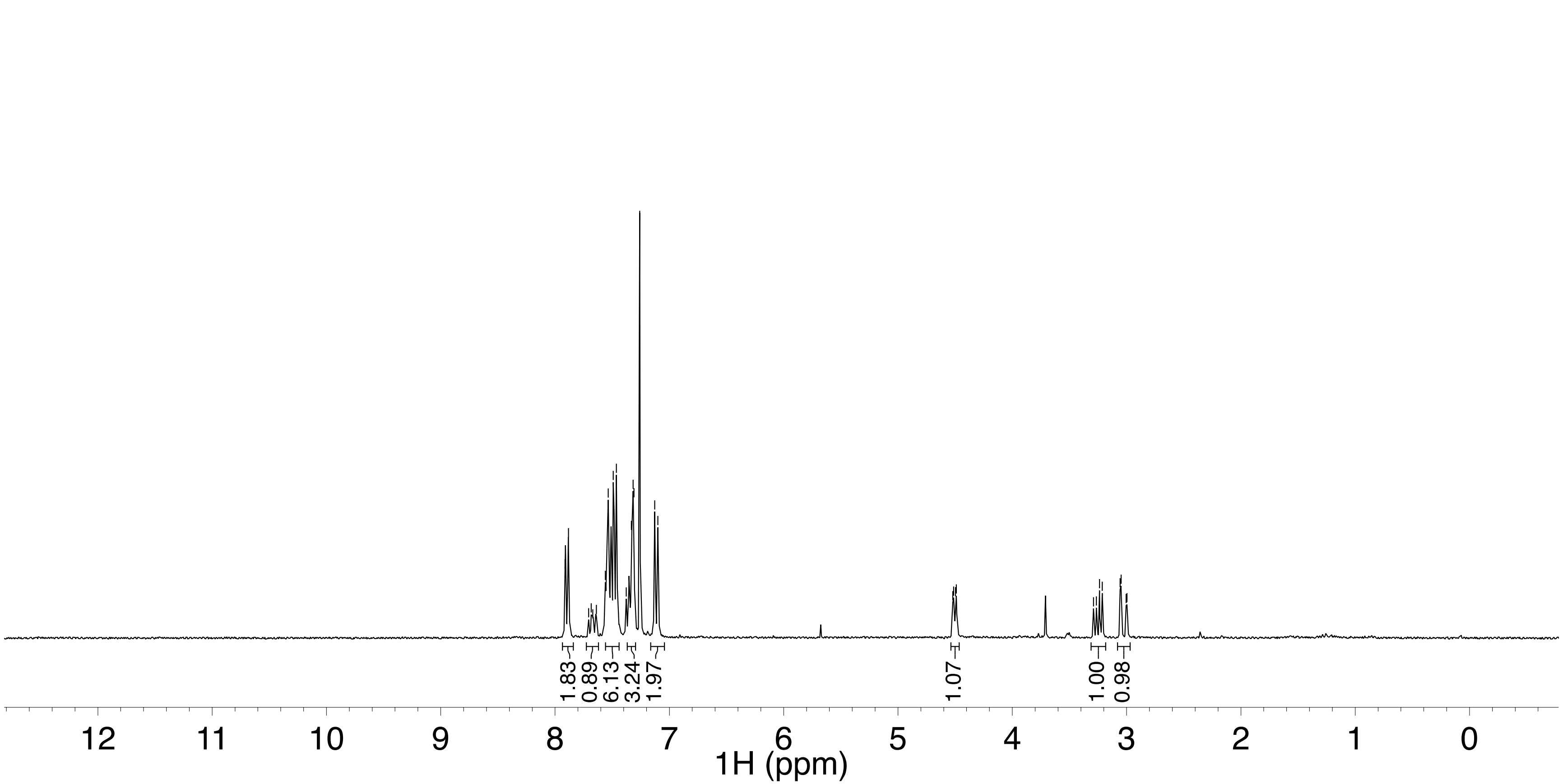

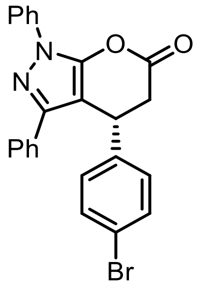

59, $^{13}C\{^{1}H\}$, $CDCl_3$, 126 MHz

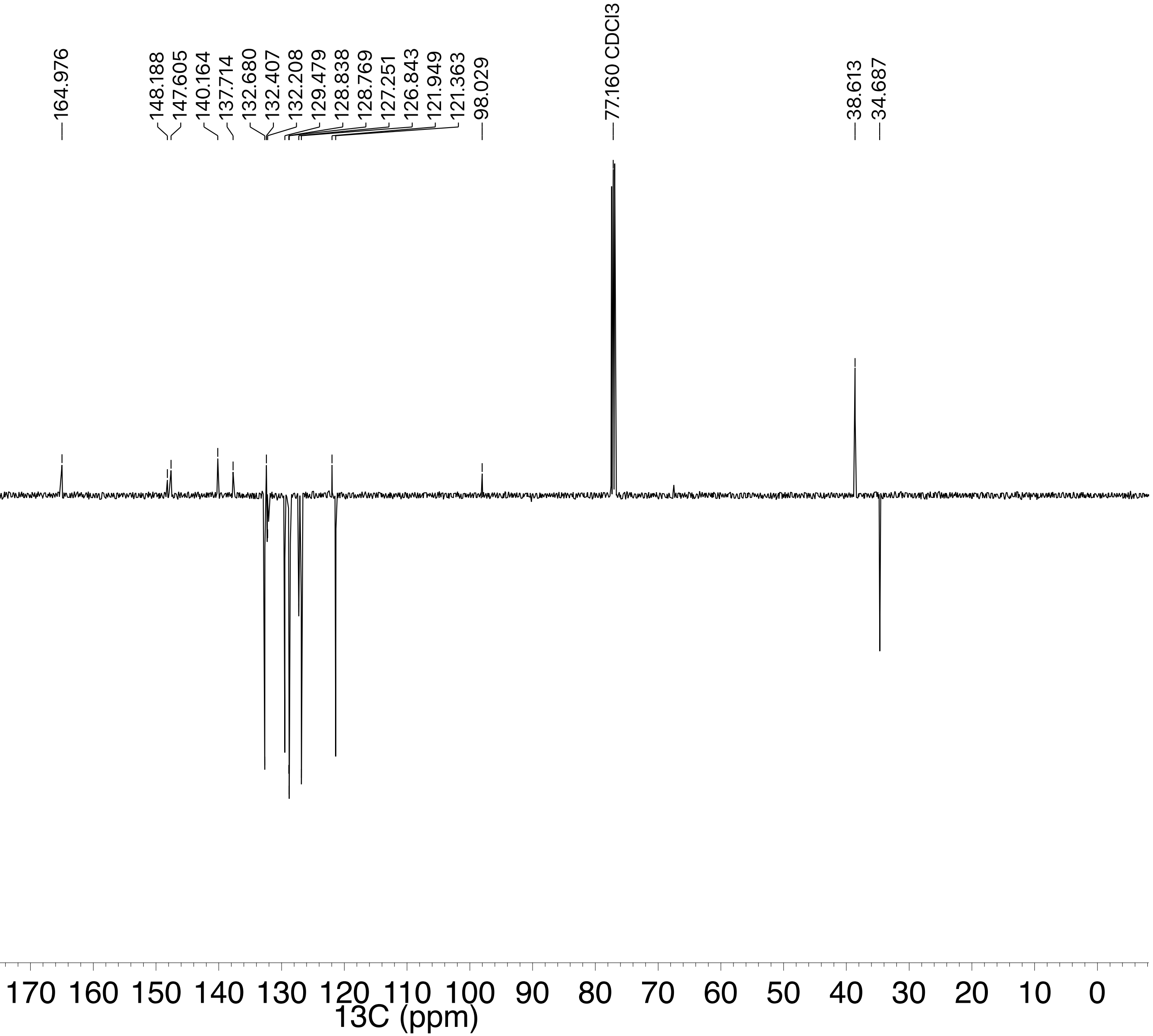

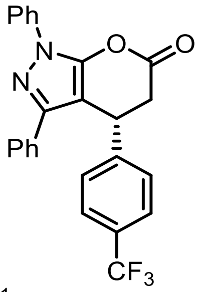

60, ^{1}H, $CDCl_3$, 300 MHz

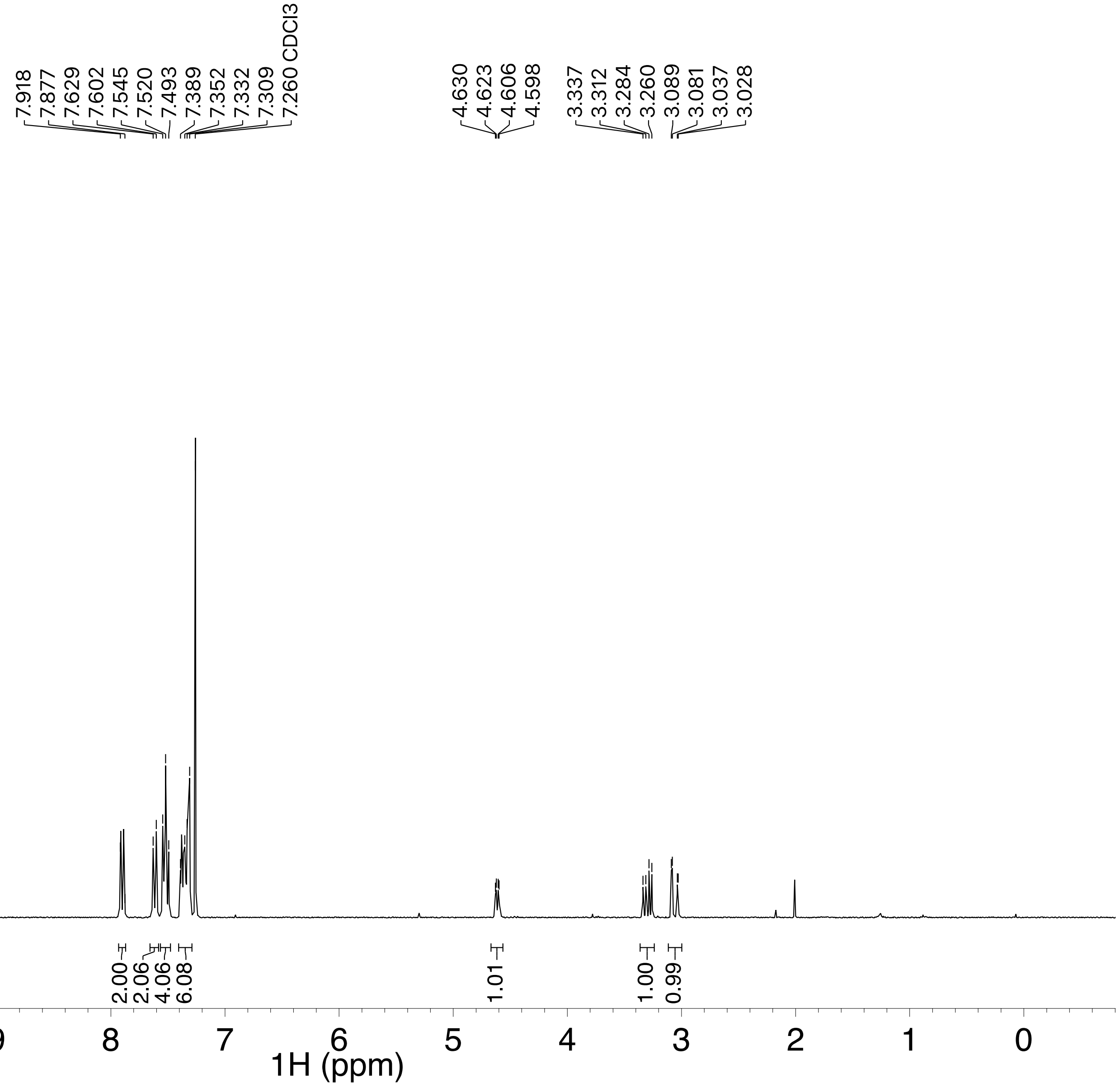

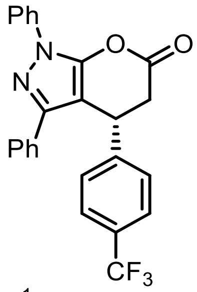

60, $^{13}C\{^1H\}$, $CDCl_3$, 126 MHz

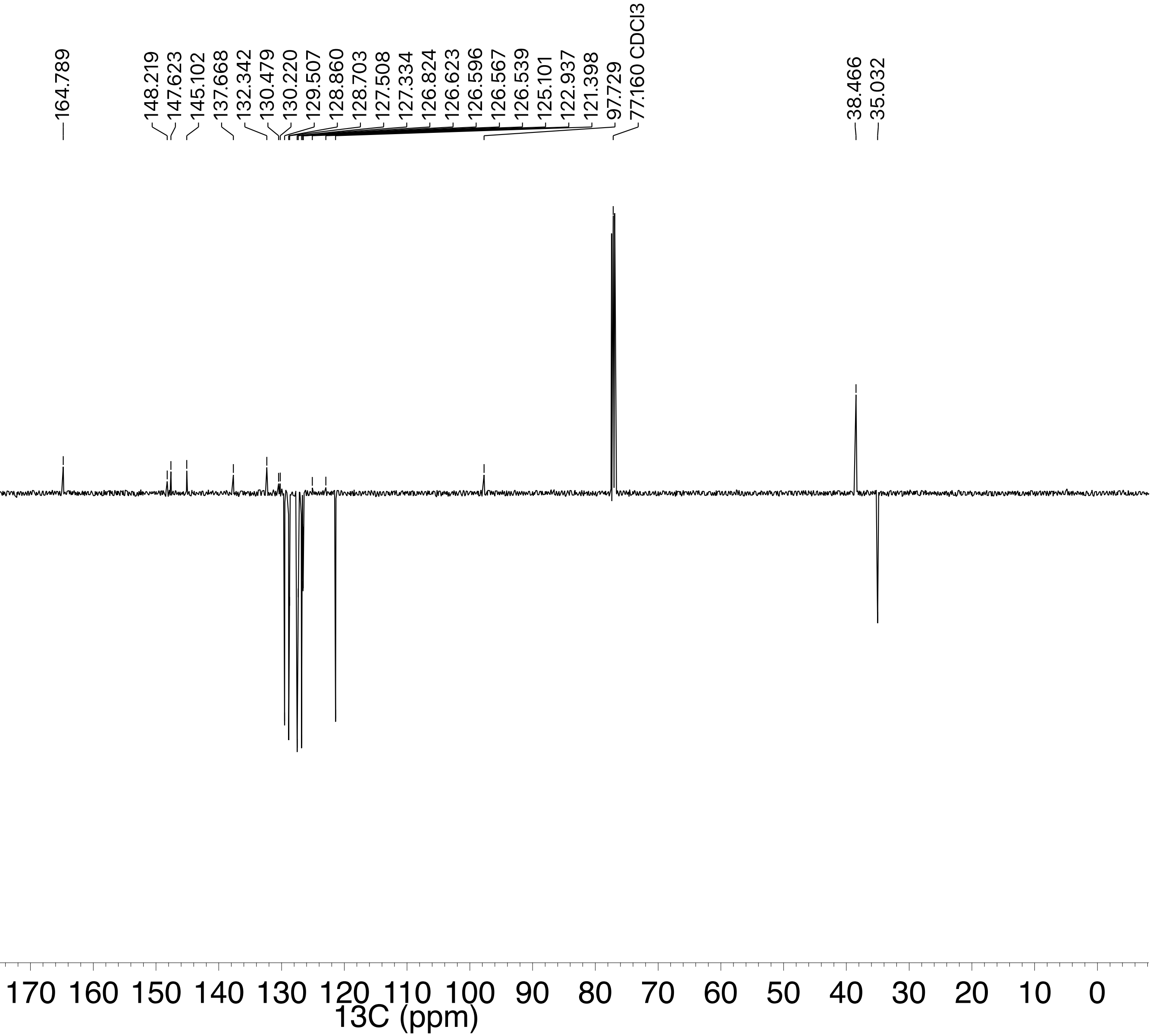

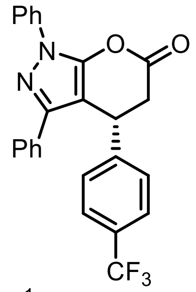

60, $^{19}F\{^{1}H\}$, $CDCl_3$, 470 MHz

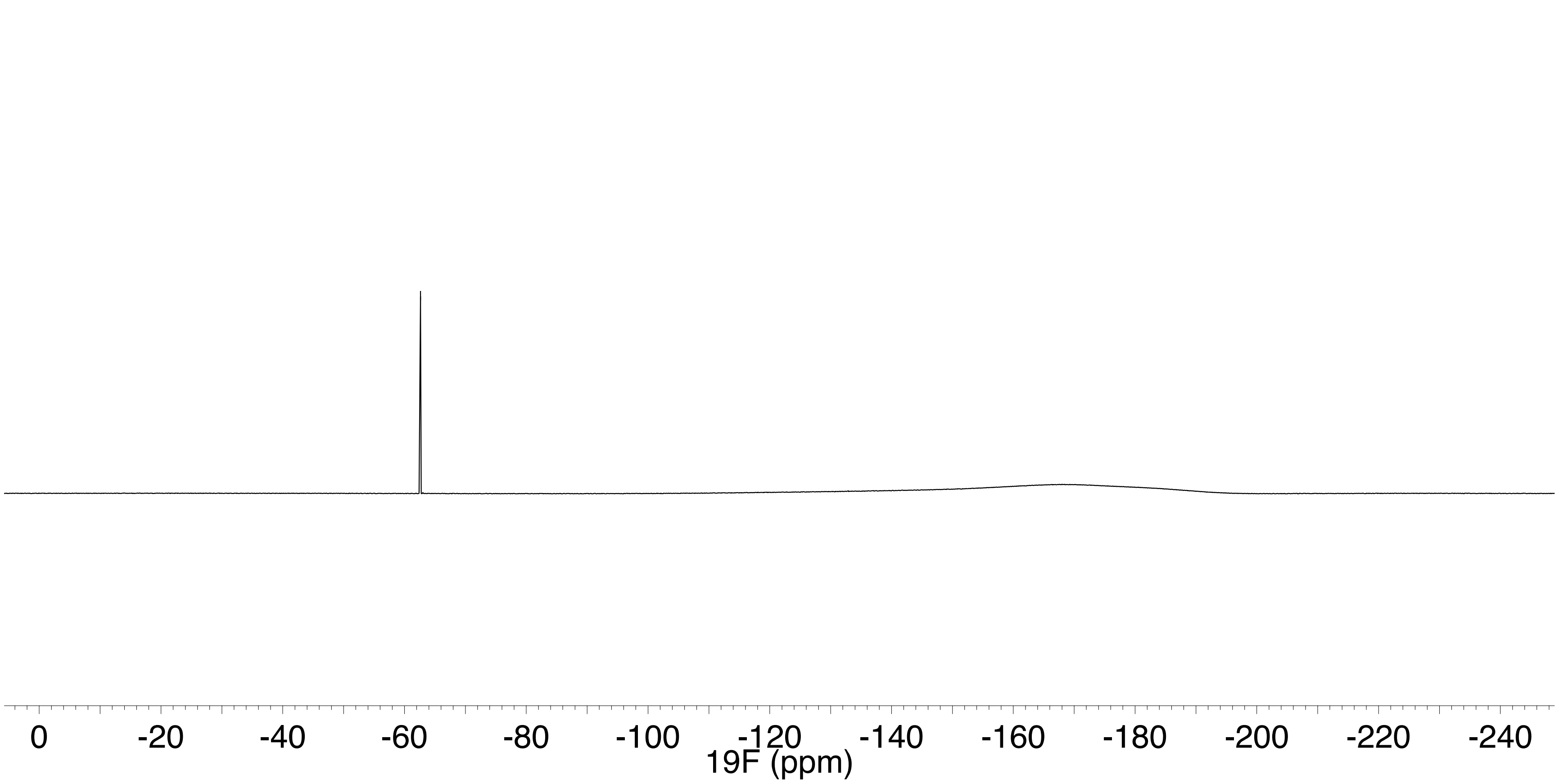

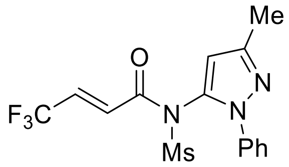

21, ^{1}H, $CDCl_3$, 400 MHz

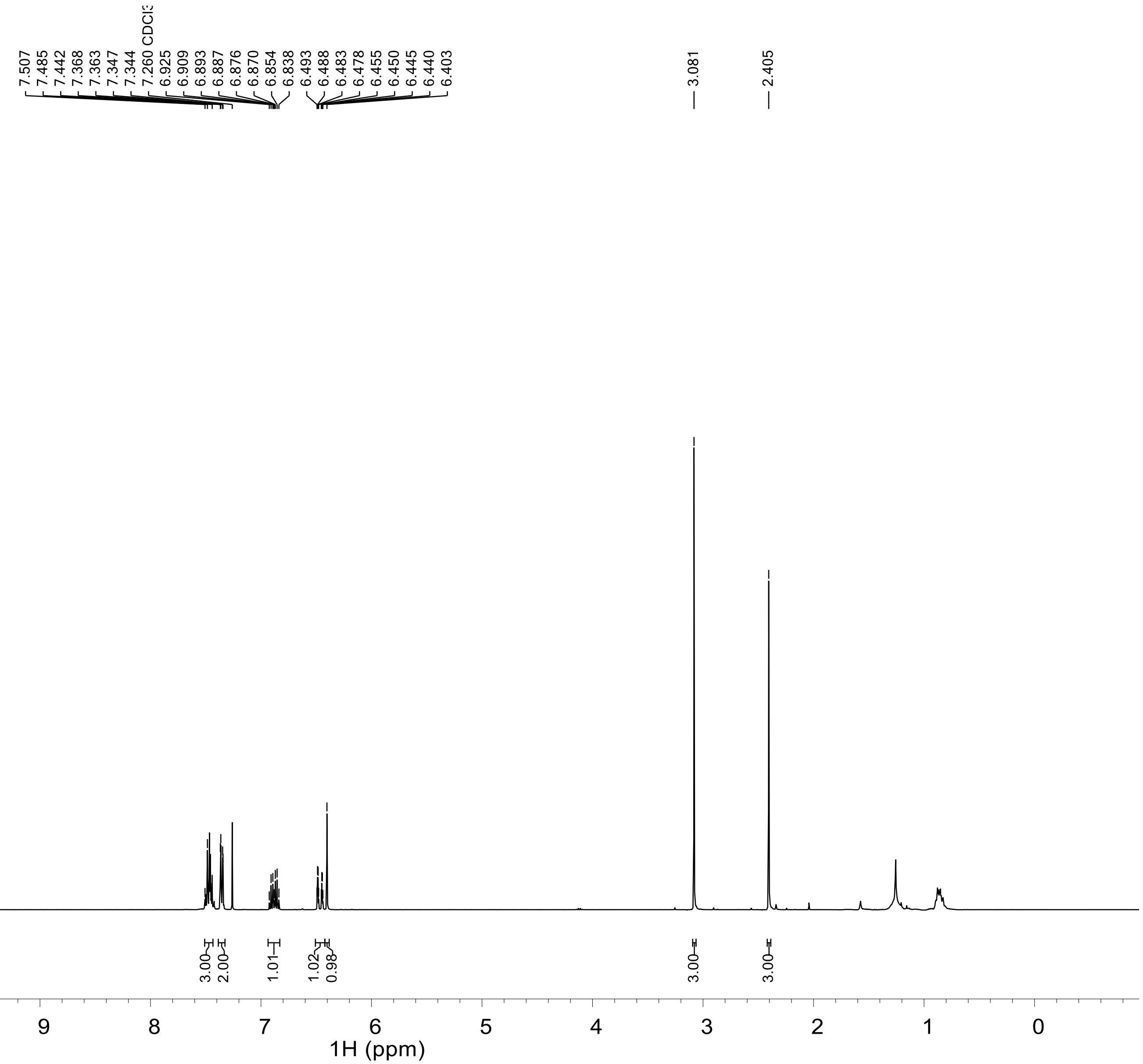

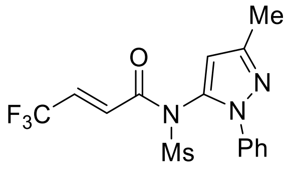

21, $^{13}C\{^{1}H\}$, $CDCl_3$, 126 MHz

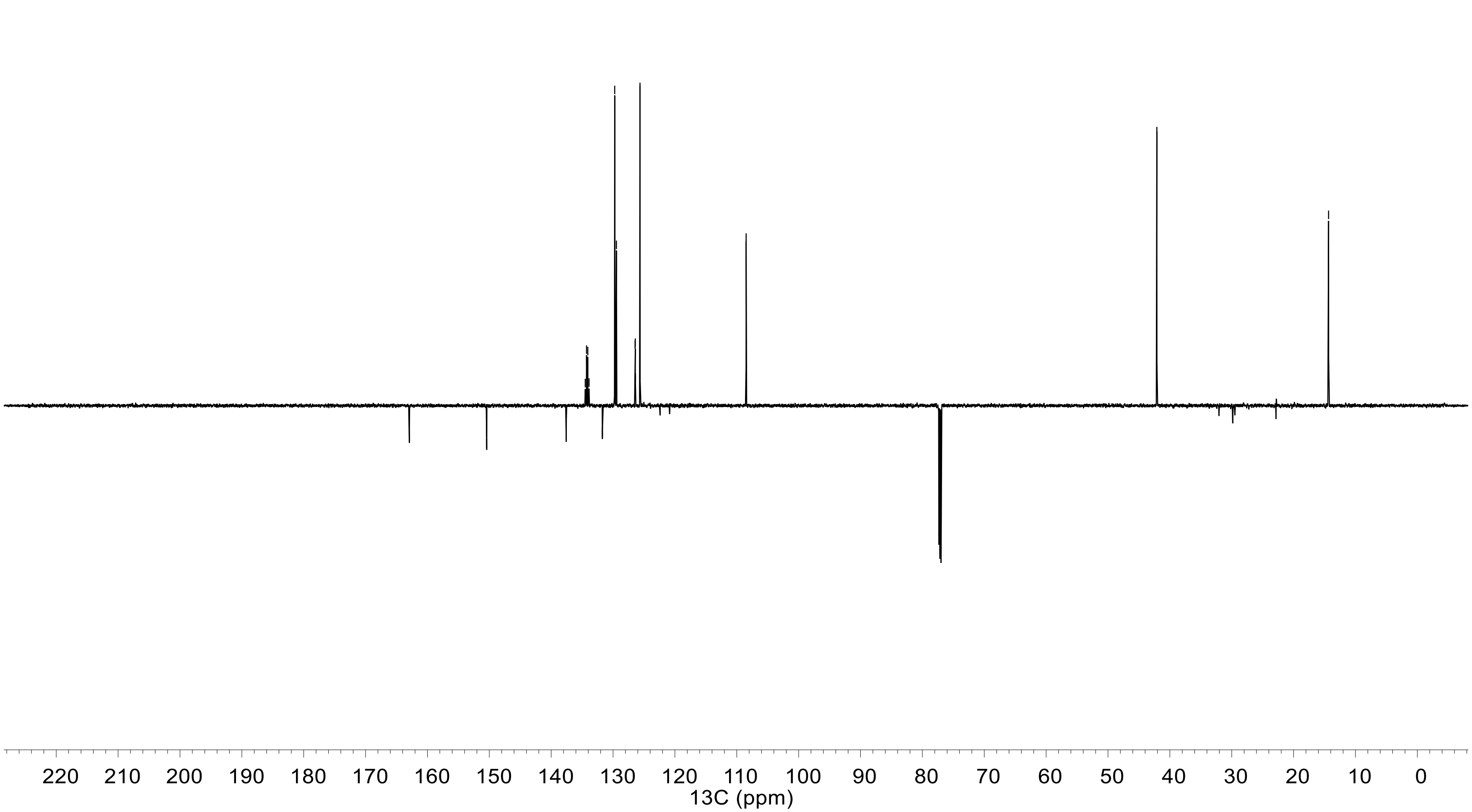

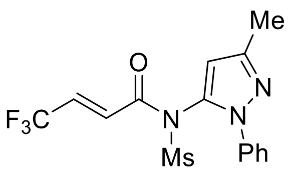

21, $^{19}F\{^1H\}$, $CDCl_3$, 377 MHz

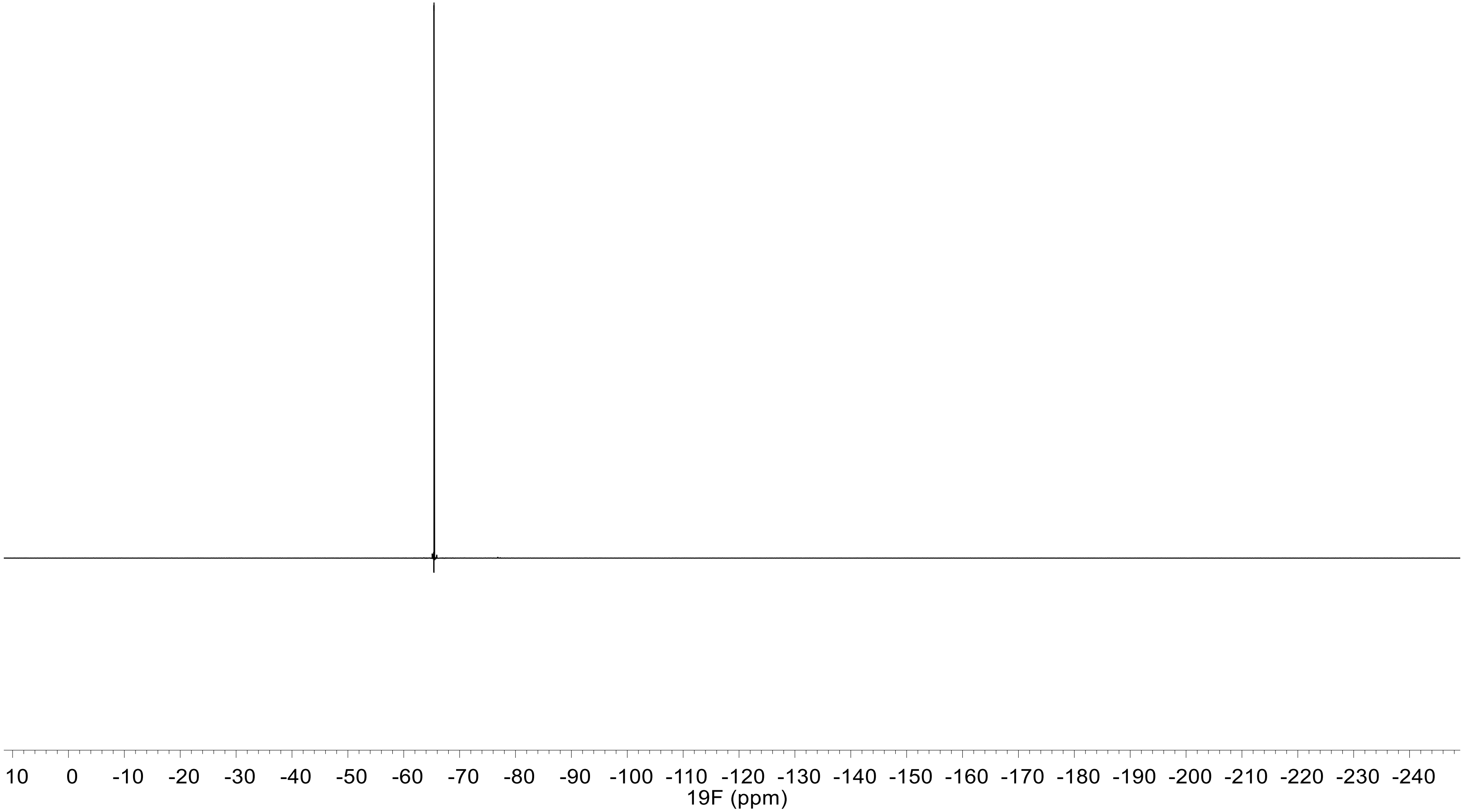

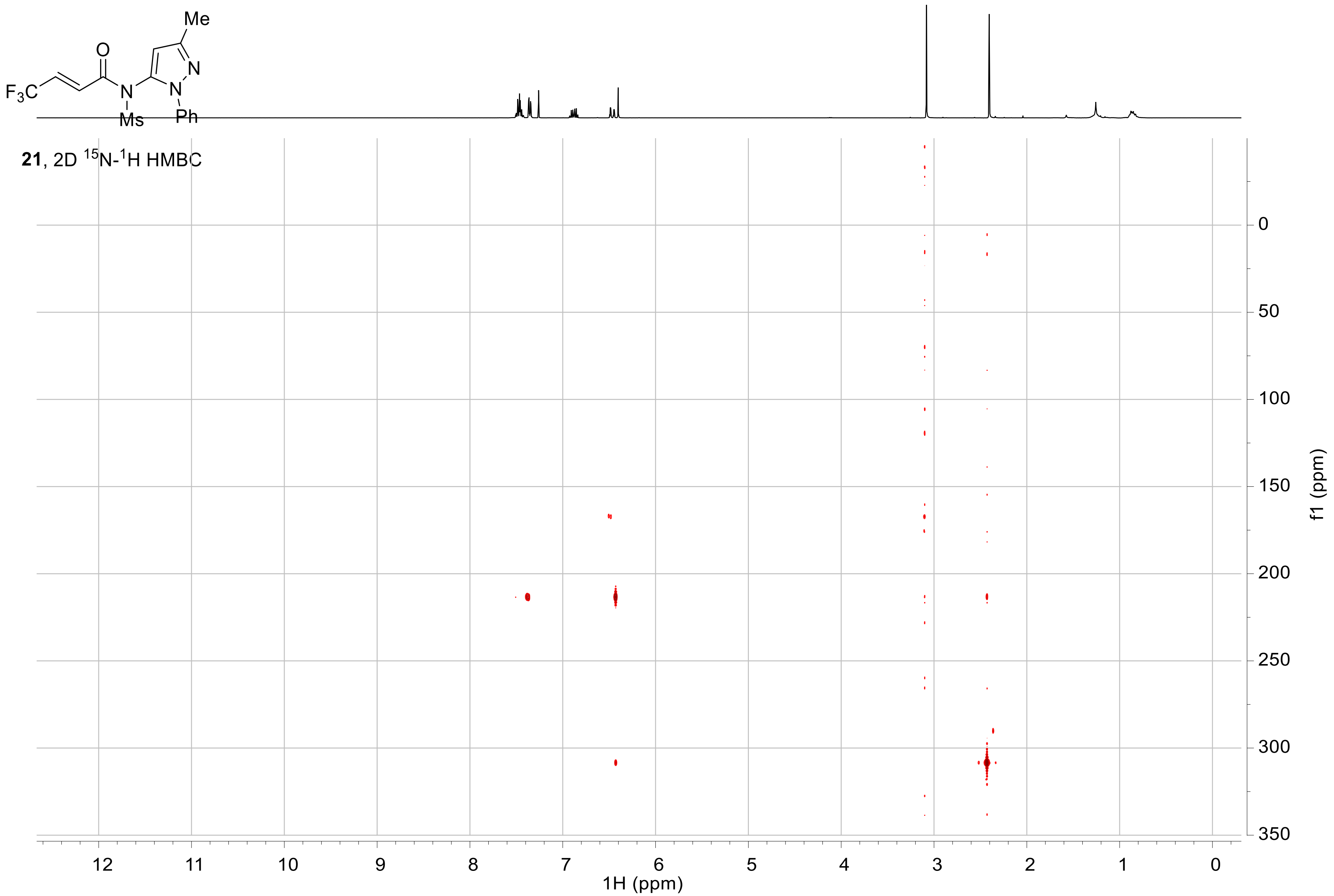

Me
O
N
F3C
N
N
Ms
Ph
21, 2D 15N-1H HMBC
0
50
100
150
200
250
300
350
f1 (ppm)
12
11
10
9
8
7
6
5
4
3
2
1
0
1H (ppm)

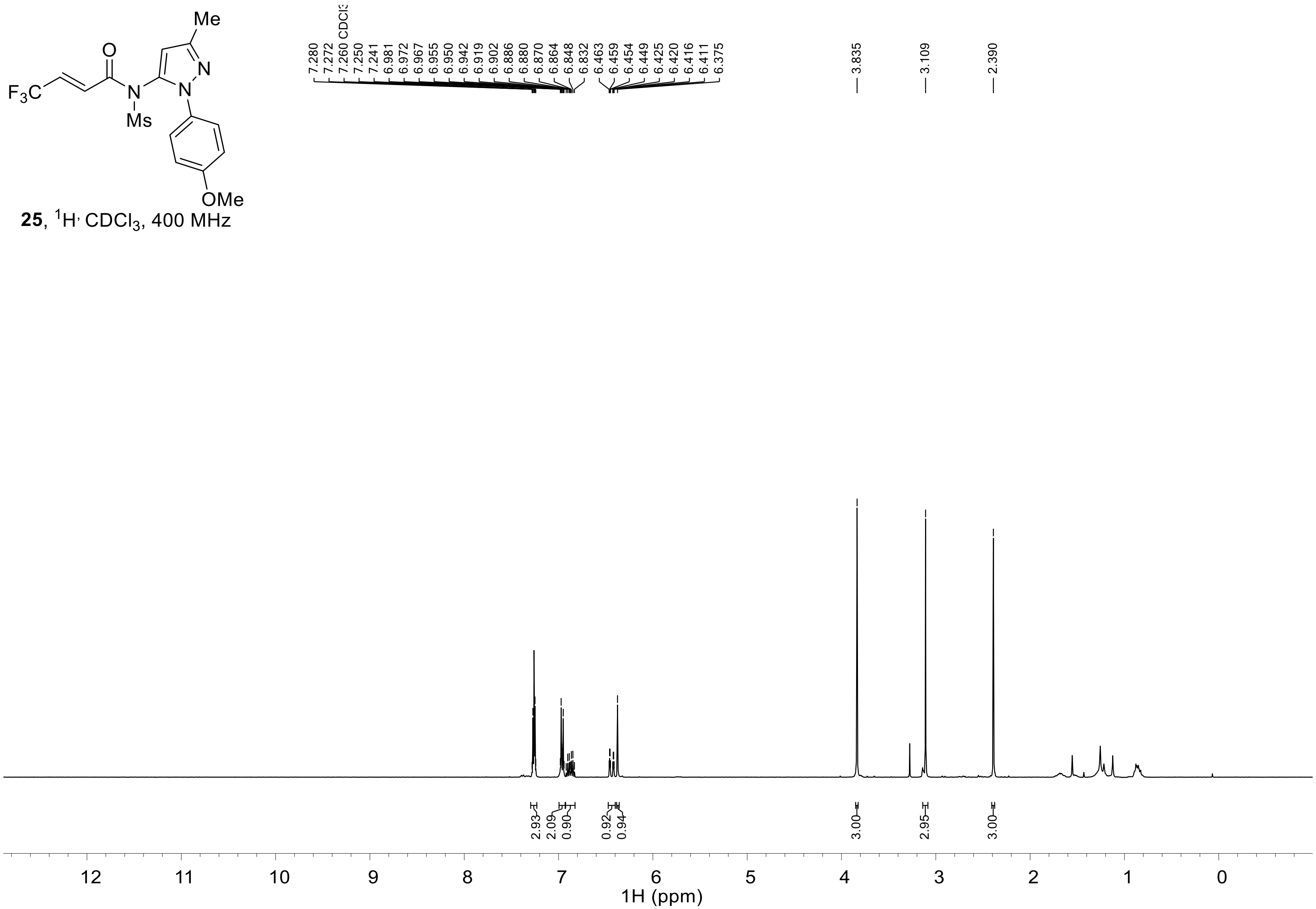

25, ^{1}H, $CDCl_3$, 400 MHz

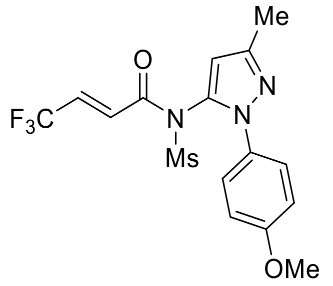

25, $^{13}C\{^1H\}$, $CDCl_3$, 126 MHz

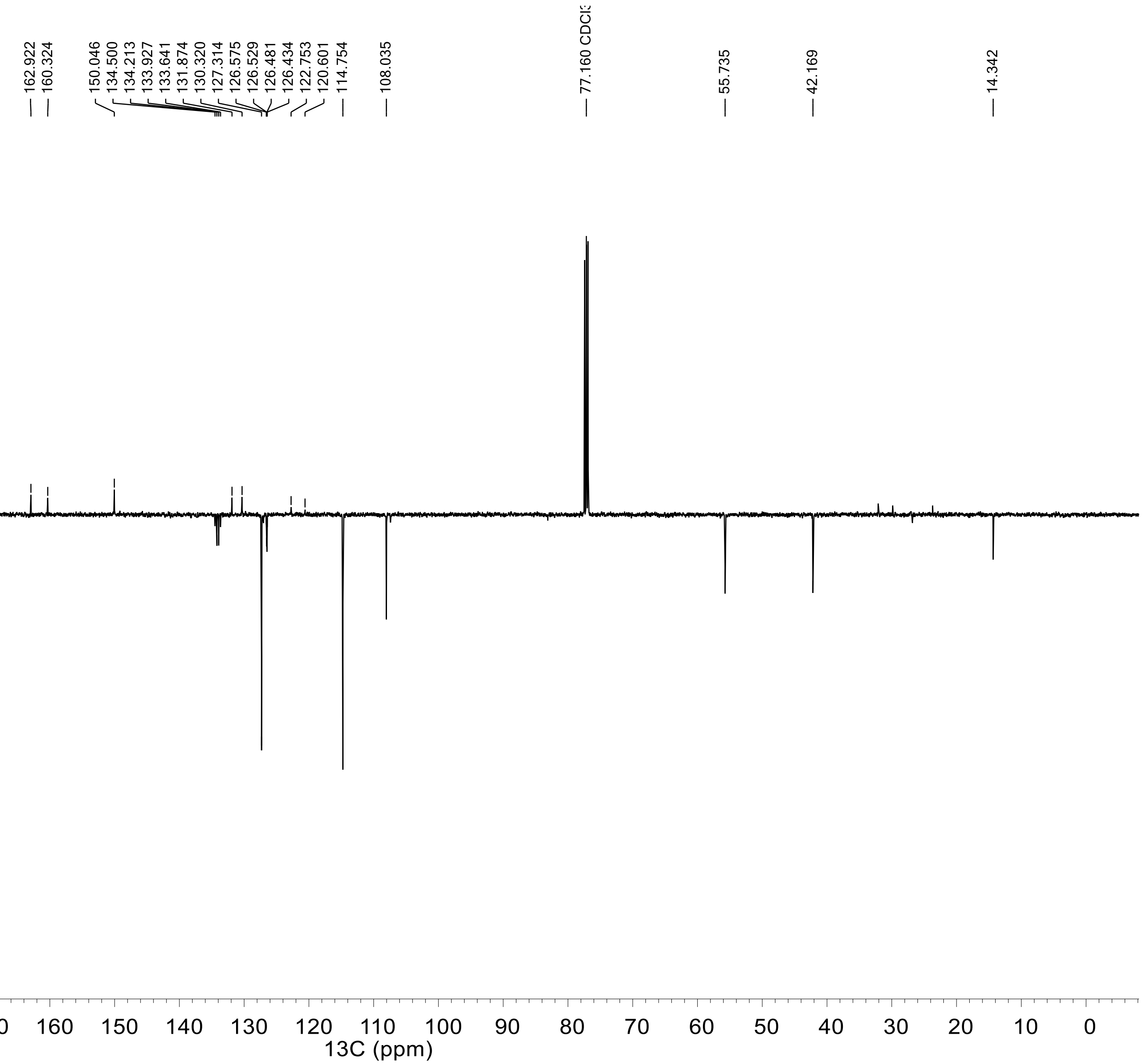

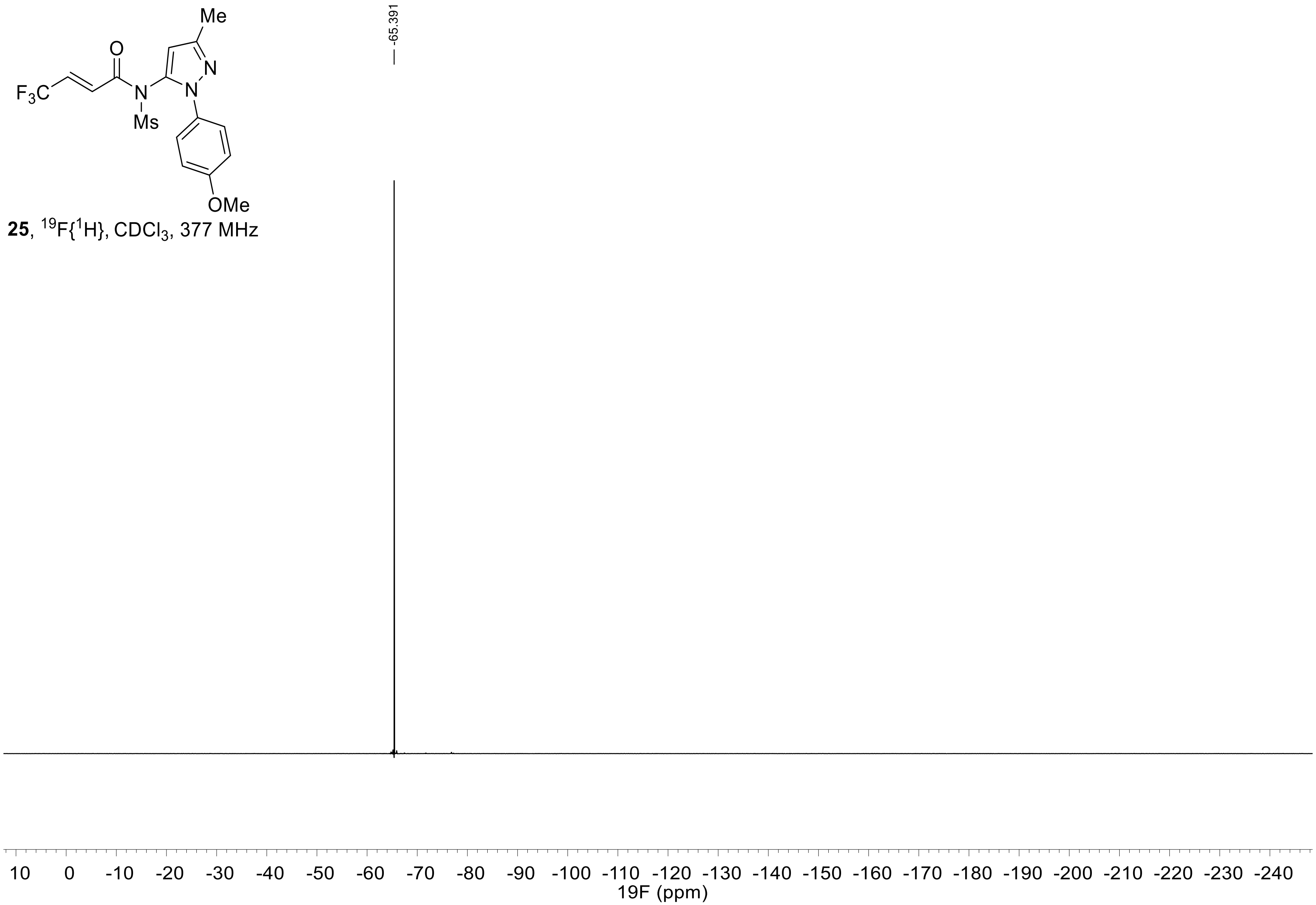

25, $^{19}F\{^{1}H\}$, $CDCl_3$, 377 MHz

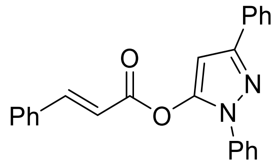

45, ^1H, $CDCl_3$. 400 MHz

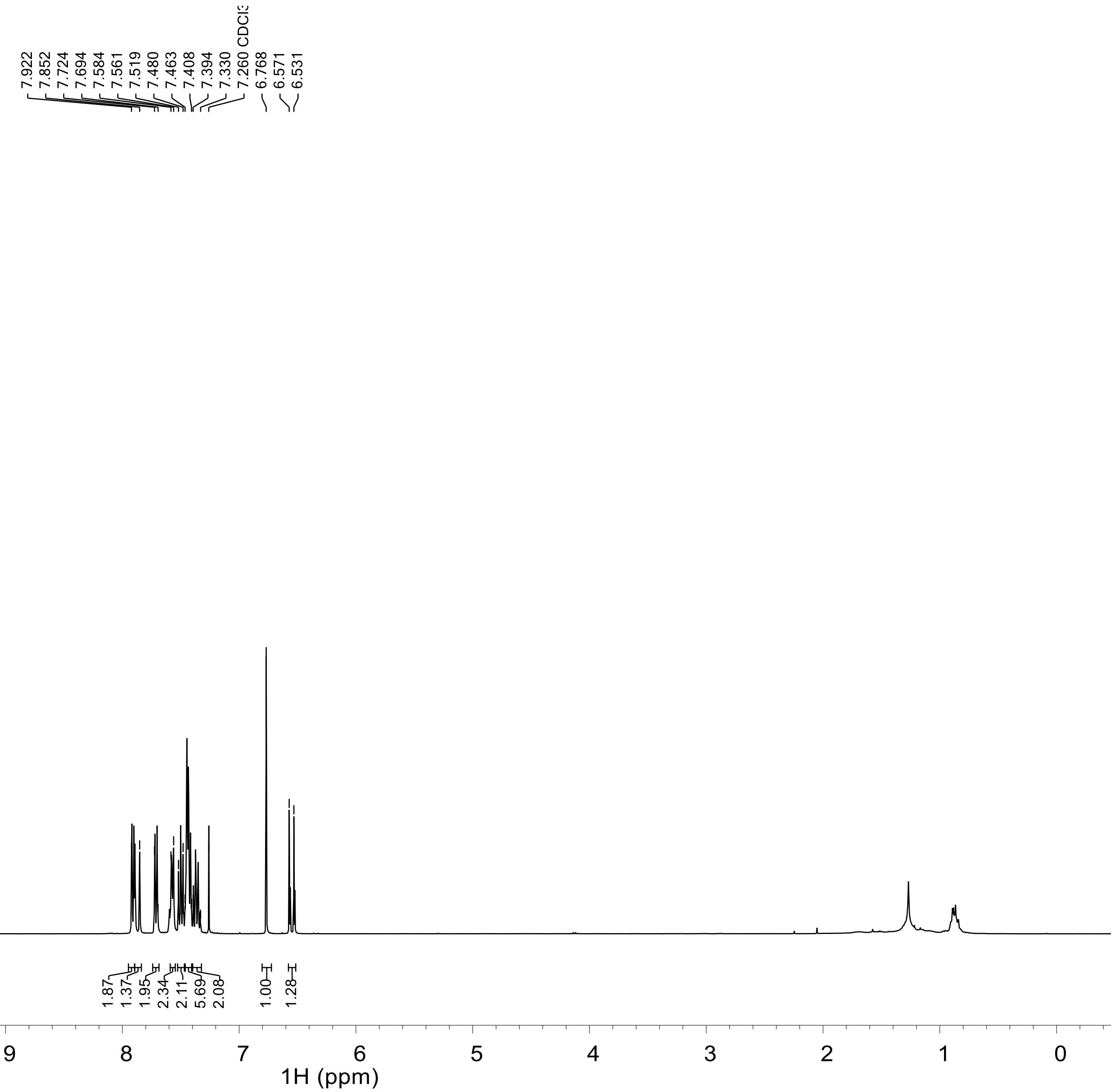

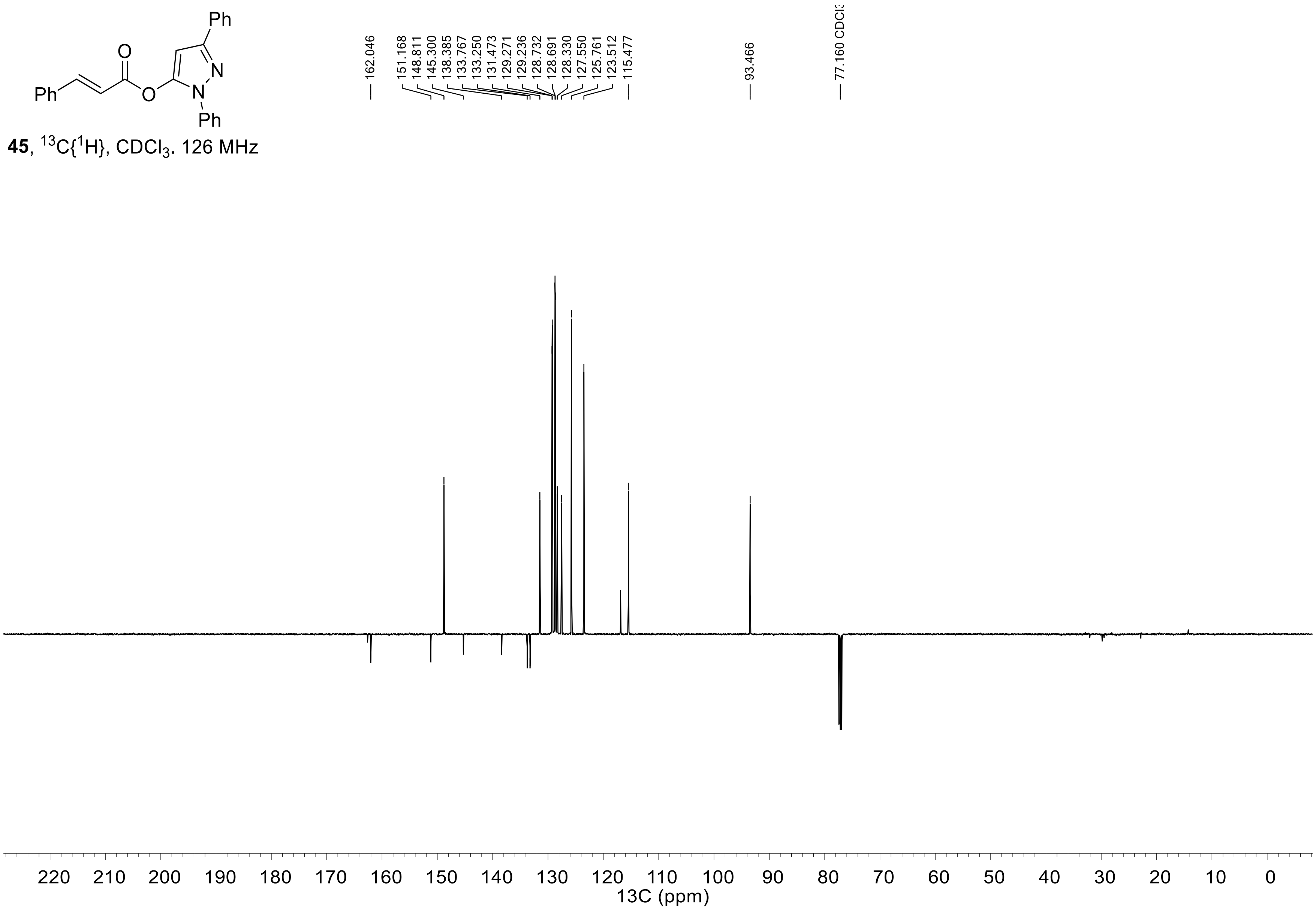

45, $^{13}C\{^1H\}$, $CDCl_3$. 126 MHz

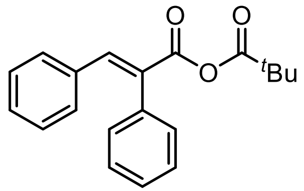

S56, ^{1}H, $CDCl_3$, 400 MHz

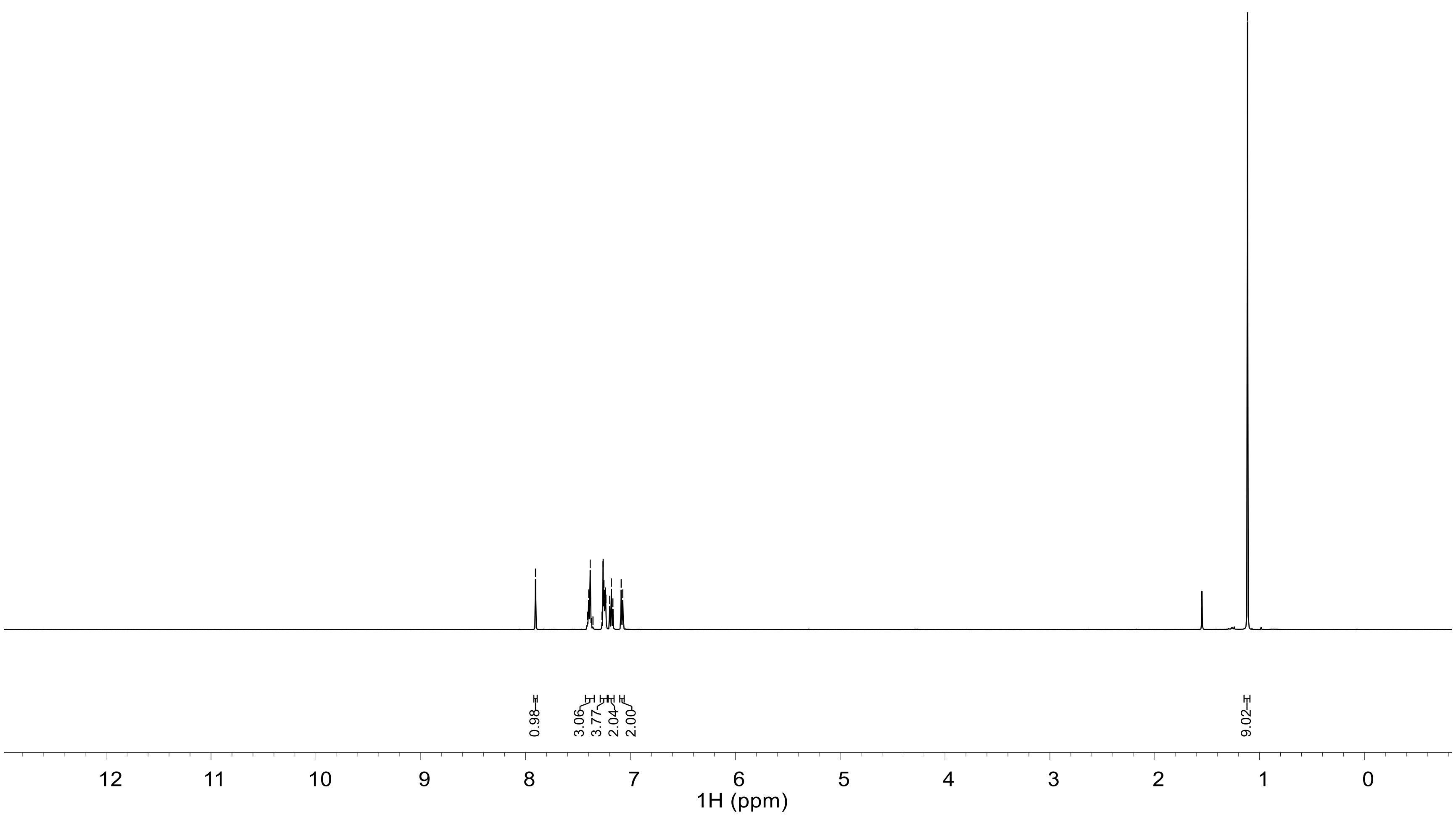

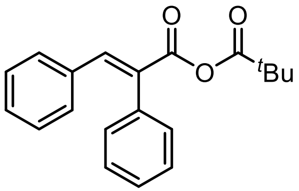

S56, $^{13}C\{^1H\}$, $CDCl_3$, 126 MHz

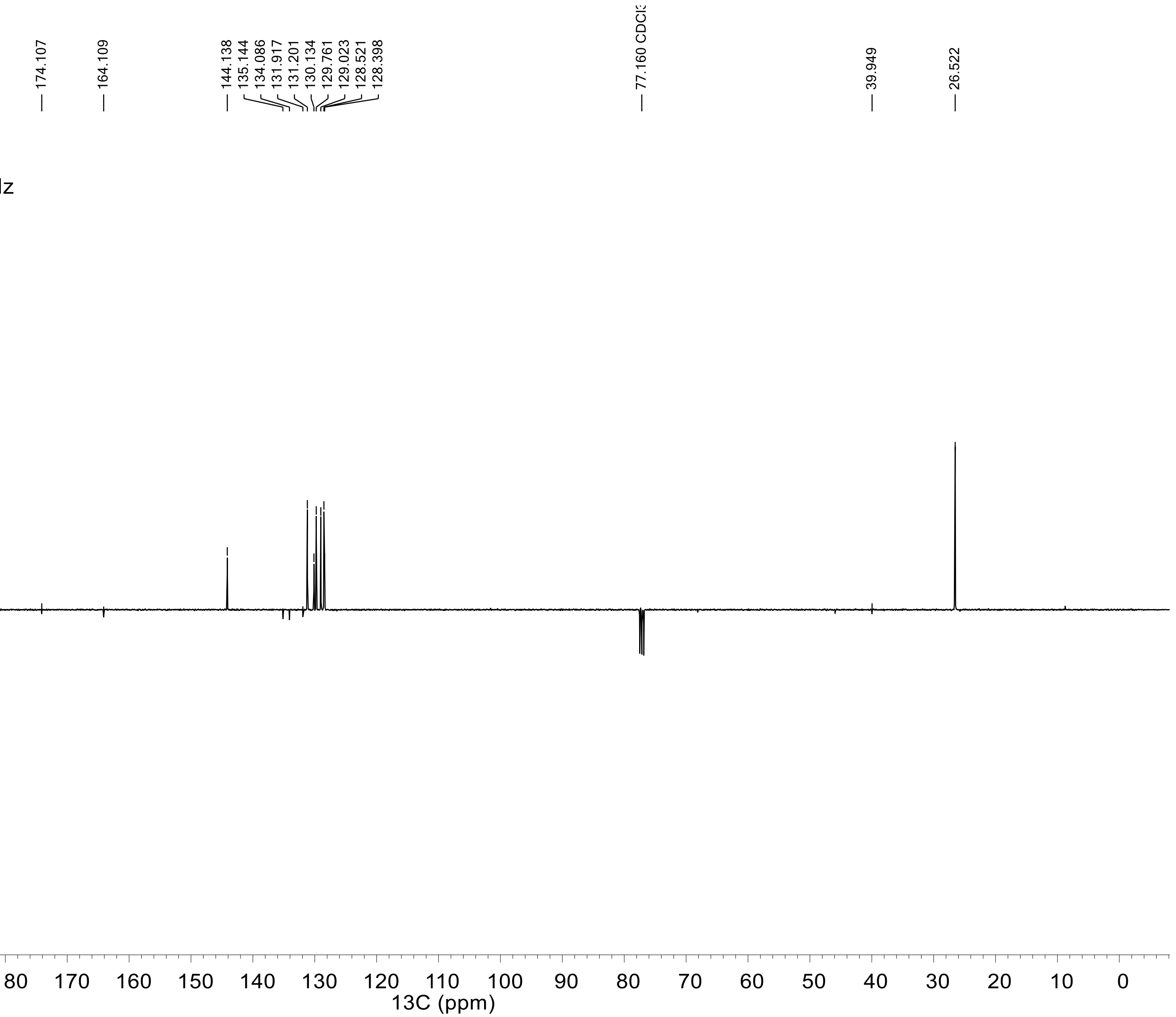

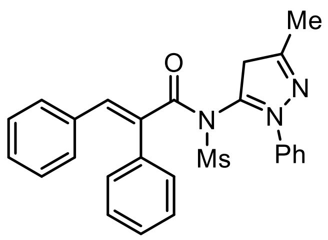

S57, ^1H, $CDCl_3$, 400 MHz

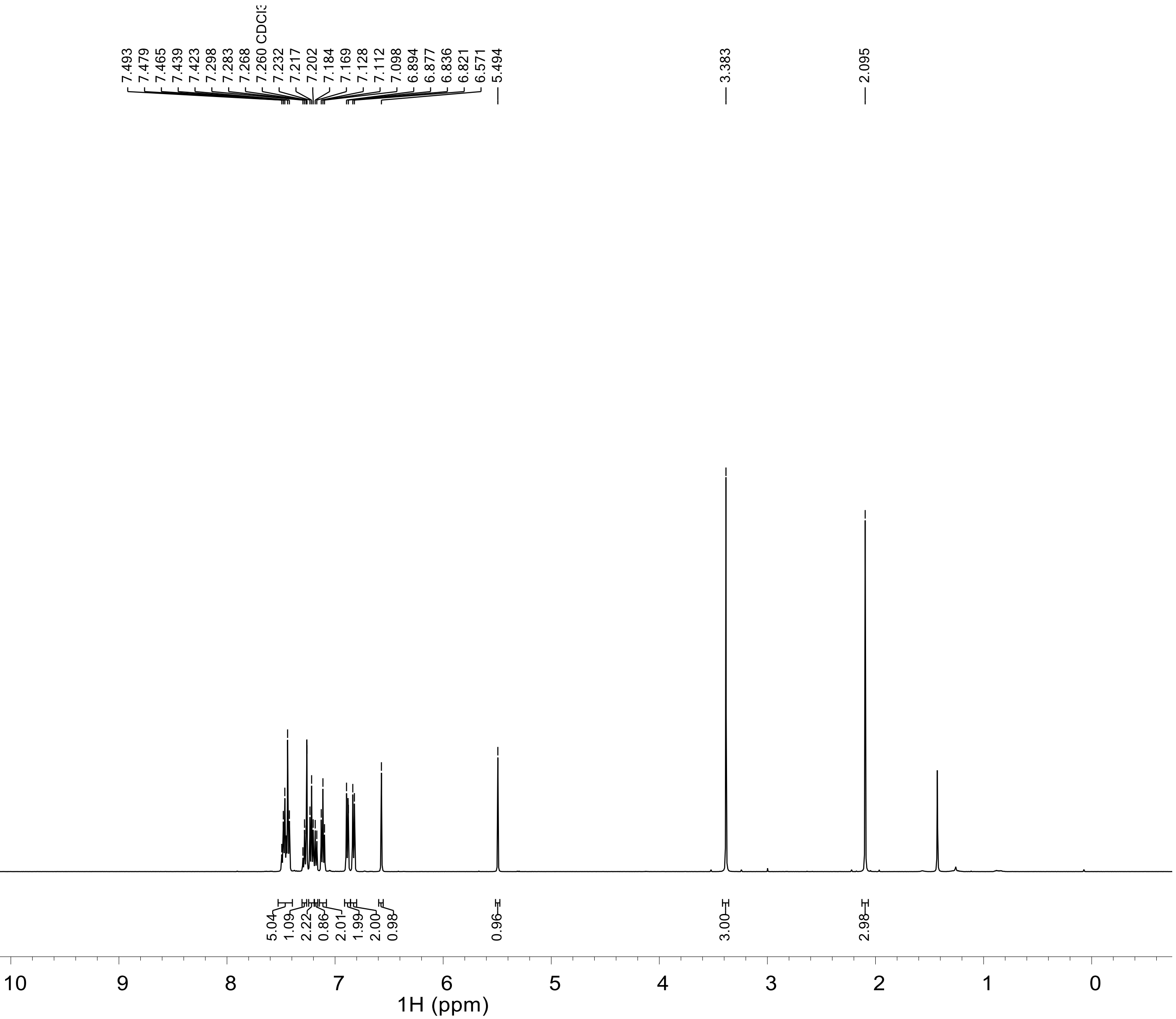

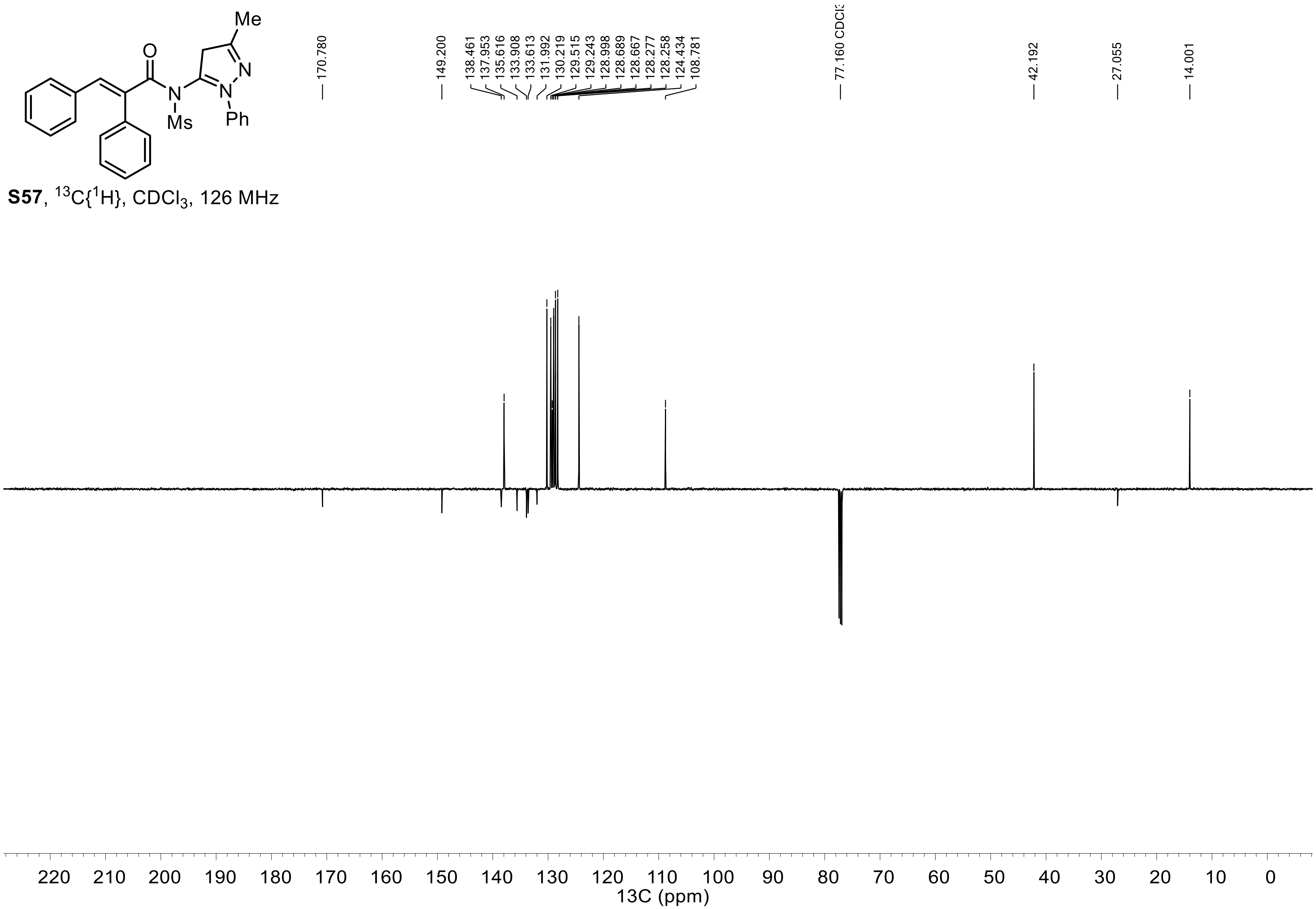

S57, $^{13}C\{^1H\}$, $CDCl_3$, 126 MHz

11. HPLC data

HPLC data for **19**, Chiralpak AD-H (90:10 hexane : IPA, flow rate 1mL.min^{-1}, 211 nm, 30 °C) t_R (4*S*): 18.1 min, t_R (4*R*): 22.5 min, 99:1 er

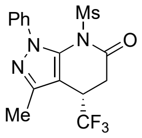

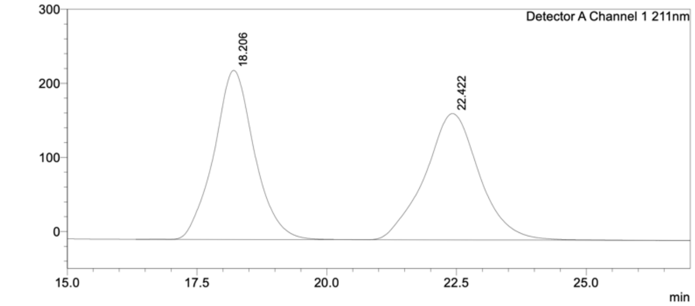

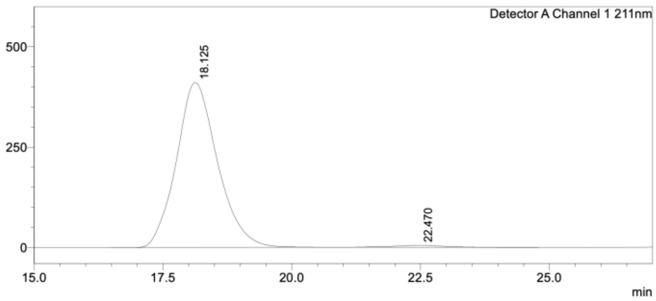

<Peak Table>

Detector A Channel 1 211nm

Peak#	Ret. Time	Area%
1	18.206	49.885
2	22.422	50.115
Total		100.000

<Peak Table>

Detector A Channel 1 211nm

Peak#	Ret. Time	Area%
1	18.125	98.556
2	22.470	1.444
Total		100.000

HPLC data for **28** (from (*E*)-**S18**), Chiralpak AD-H (90:10 hexane : IPA, flow rate 1mL.min^{-1}, 211 nm, 30 °C) t_R(4*S*): 24.7 min, t_R(4*R*): 30.1 min, 95:5 er

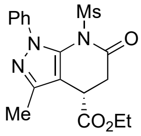

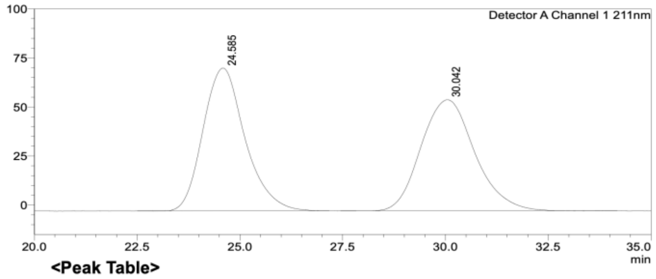

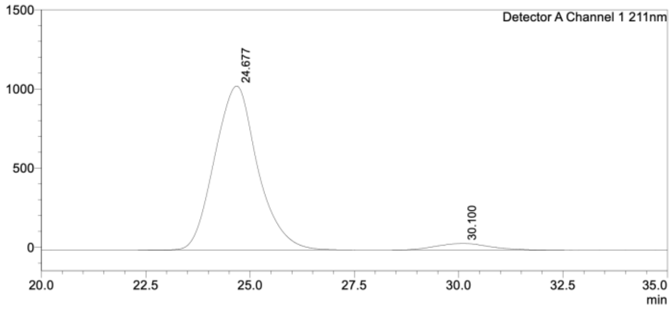

<Peak Table>

Detector A Channel 1 211nm

Peak#	Ret. Time	Area%
1	24.585	50.088
2	30.042	49.912
Total		100.000

<Peak Table>

Detector A Channel 1 211nm

Peak#	Ret. Time	Area%
1	24.677	95.209
2	30.100	4.791
Total		100.000

HPLC data for **28** (from (*Z*)-**S18**), Chiralak AD-H (90:10 hexane : IPA, flow rate 1mL.min^{-1}, 211 nm, 30 °C) t_R(4*S*): 25.2 min, t_R(4*R*): 30.6 min, 90:10 er

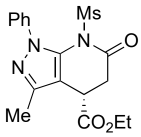

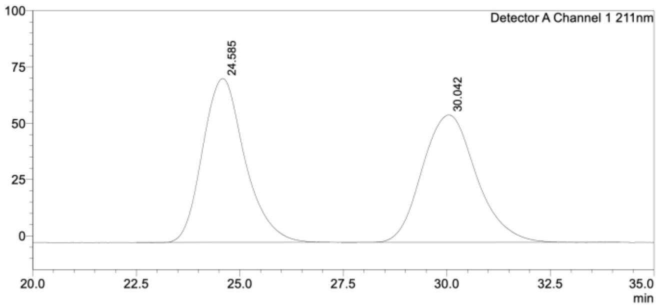

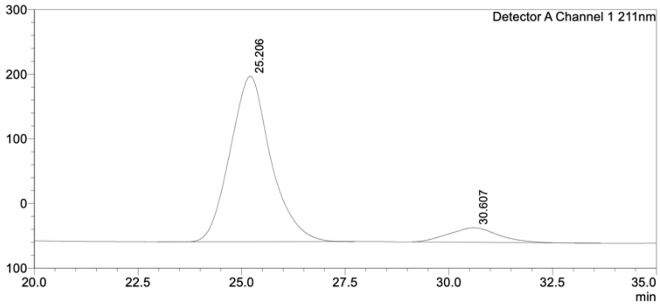

<Peak Table>

Peak#	Ret. Time	Area%
1	24.585	50.088
2	30.042	49.912
Total		100.000

<Peak Table>

Detector A Channel 1 211nm

Peak#	Ret. Time	Area%
1	25.206	90.266
2	30.607	9.734
Total		100.000

HPLC data for **29,** Chiralpak AD-H (90:10 hexane : IPA, flow rate 1mL.min^{-1}, 211 nm, 30 °C) t_R (4*S*): 14.9 min, t_R (4*R*): 18.8 min, 93:7 er

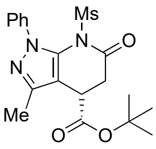

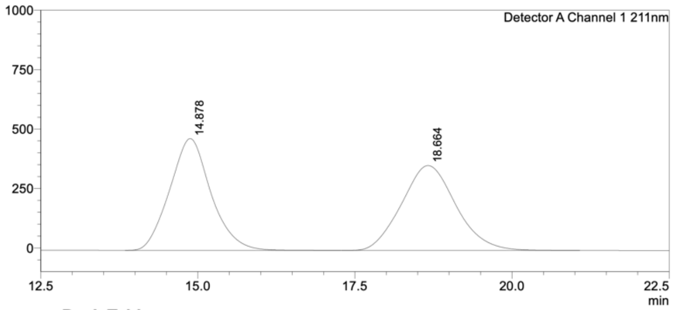

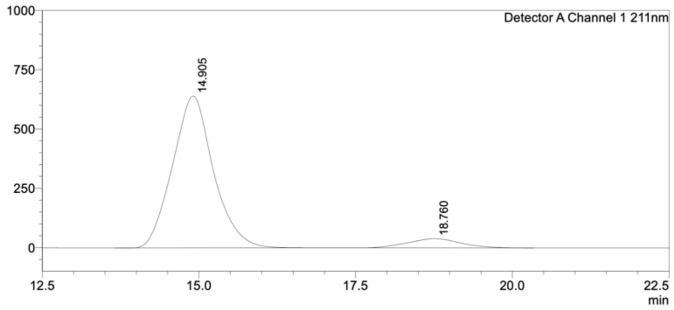

<Peak Table>

Detector A Channel 1 211nm

Peak#	Ret. Time	Area%
1	14.878	50.006
2	18.664	49.994
Total		100.000

<Peak Table>

Detector A Channel 1 211nm

Peak#	Ret. Time	Area%
1	14.905	92.738
2	18.760	7.262
Total		100.000

HPLC data for **30,** Chiralpak IC (75:25 hexane : IPA, flow rate 1mL.min^{-1}, 211 nm, 30 °C) t$_R$ (4*S*): 12.4 min, t$_R$ (4*R*): 19.6 min, 85:15 er

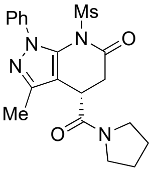

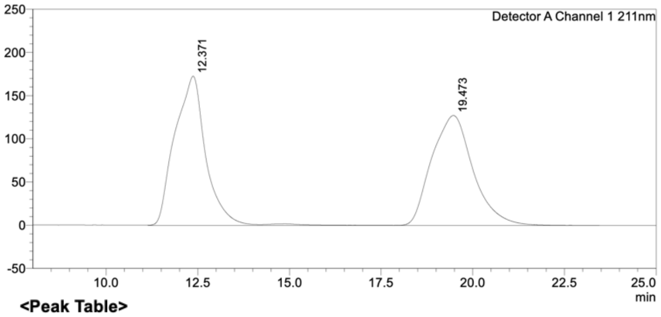

<Peak Table>

Detector A Channel 1 211nm

Peak#	Ret. Time	Area%
1	12.371	50.434
2	19.473	49.566
Total		100.000

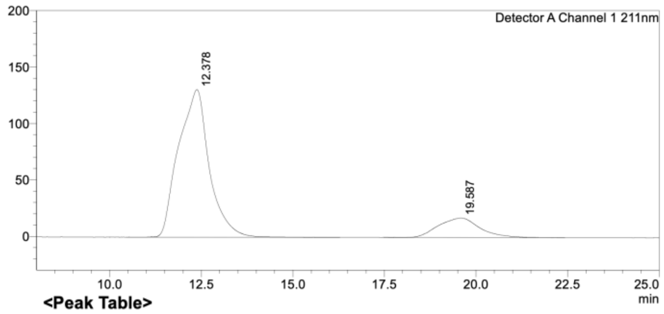

<Peak Table>

Detector A Channel 1 211nm

Peak#	Ret. Time	Area%
1	12.378	84.918
2	19.587	15.082
Total		100.000

HPLC data for **31**, Chiralpak AD-H (85:15 hexane : IPA, flow rate 1mL.min^{-1}, 211 nm, 30 °C) t$_R$ (4*S*): 23.0 min, t$_R$ (4*R*): 45.3 min, er 84:16

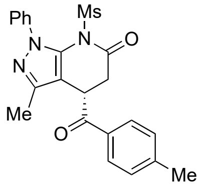

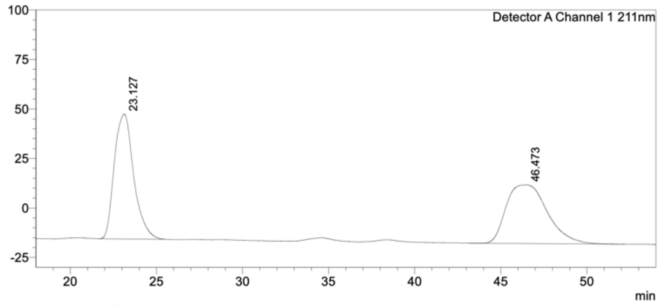

<Peak Table>

Detector A Channel 1 211nm

Peak#	Ret. Time	Area%
1	23.127	50.060
2	46.473	49.940
Total		100.000

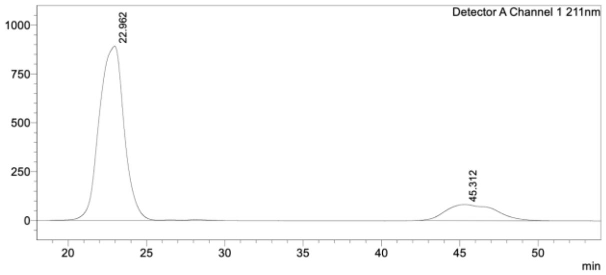

<Peak Table>

Detector A Channel 1 211nm

Peak#	Ret. Time	Area%
1	22.962	84.393
2	45.312	15.607
Total		100.000

HPLC data for **32,** Chiralpak AD-H (75:25 hexane : IPA, flow rate 1mL.min^{-1}, 211 nm, 30 °C) t$_R$ (4*S*): 12.3 min, t$_R$ (4*R*): 19.1 min, 79:21 er

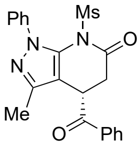

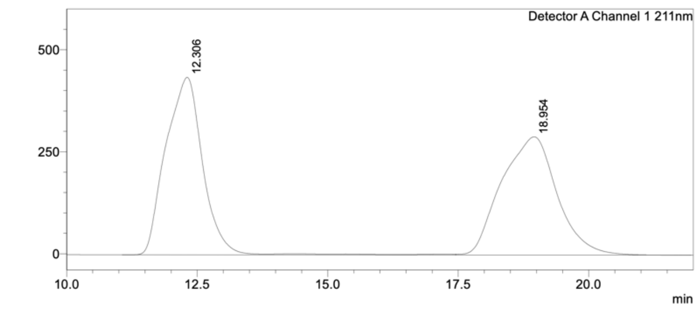

<Peak Table>

Detector A Channel 1 211nm

Peak#	Ret. Time	Area%
1	12.306	50.113
2	18.954	49.887
Total		100.000

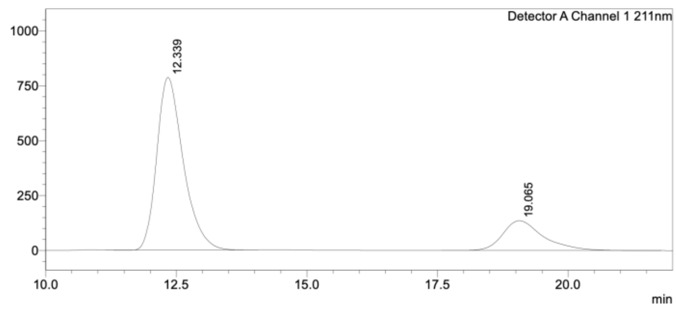

<Peak Table>

Detector A Channel 1 211nm

Peak#	Ret. Time	Area%
1	12.339	79.200
2	19.065	20.800
Total		100.000

HPLC data for **33**, Chiralpak ID (88:12 hexane : IPA, flow rate 1mL.min^{-1}, 211 nm, 30 °C) t$_R$ (4*R*): 16.4 min, t$_R$ (4*S*): 19.7 min, 4:96 er

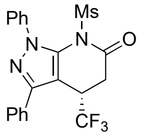

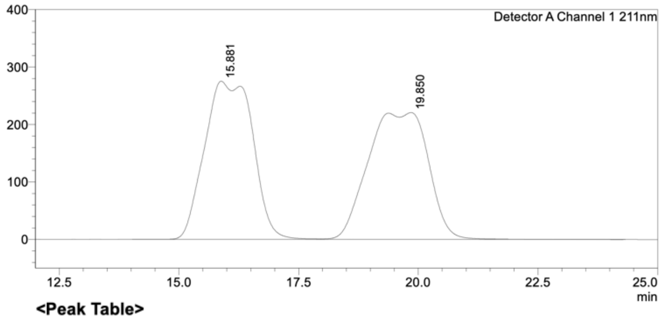

<Peak Table>

Detector A Channel 1 211nm

Peak#	Ret. Time	Area%
1	15.881	49.912
2	19.850	50.088
Total		100.000

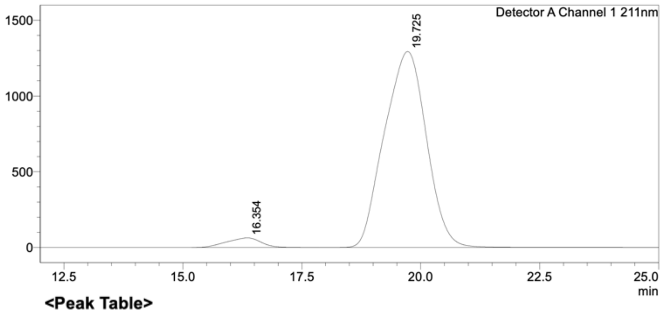

<Peak Table>

Detector A Channel 1 211nm

Peak#	Ret. Time	Area%
1	16.354	3.885
2	19.725	96.115
Total		100.000

HPLC data for **34**, Chiralpak ID (90:10 hexane : IPA, flow rate 1mL.min^{-1}, 254 nm, 30 °C) t_R (4*R*): 23.2 min, t_R (4*S*): 39.8 min, 3:97 er

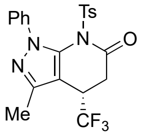

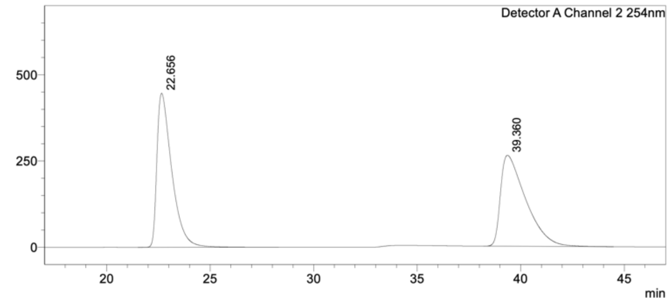

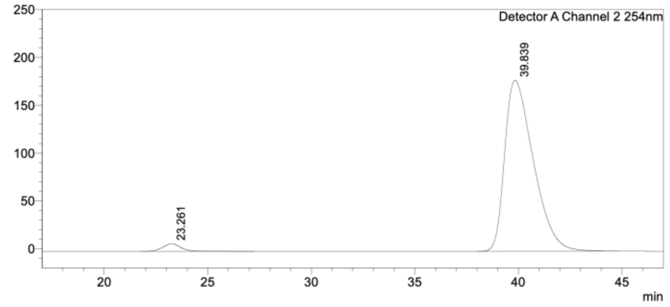

<Peak Table>

Detector A Channel 2 254nm

Peak#	Ret. Time	Area%
1	22.656	49.937
2	39.360	50.063
Total		100.000

<Peak Table>

Detector A Channel 2 254nm

Peak#	Ret. Time	Area%
1	23.261	2.842
2	39.839	97.158
Total		100.000

HPLC data for **27**, Chiralpak AD-H (88:12 hexane : IPA, flow rate 1mL.min^{-1}, 254 nm, 30 °C) t_R (4*R*): 21.6 min, t_R (4*S*): 29.5 min, 2:98 er

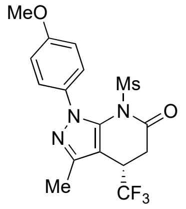

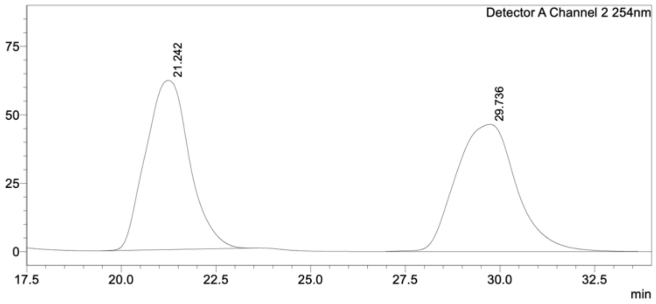

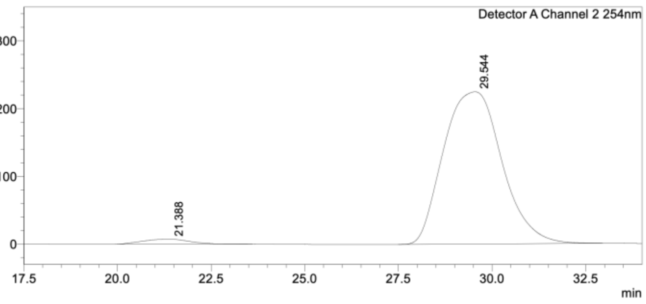

<Peak Table>

Detector A Channel 2 254nm

Peak#	Ret. Time	Area%
1	21.242	49.124
2	29.736	50.876
Total		100.000

<Peak Table>

Detector A Channel 2 254nm

Peak#	Ret. Time	Area%
1	21.388	2.446
2	29.544	97.554
Total		100.000

HPLC data for **35**, Chiralpak ID (99:1 hexane : IPA, flow rate 1mL.min^{-1}, 211 nm, 30 °C) t_R (4*R*): 14.9 min, t_R (4*S*): 18.1 min, 1:99 er

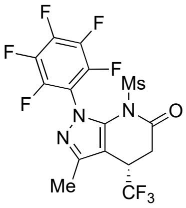

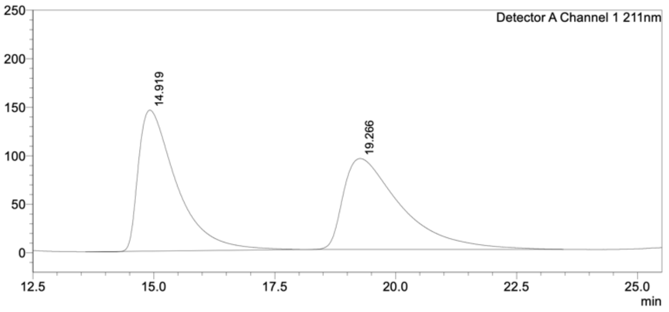

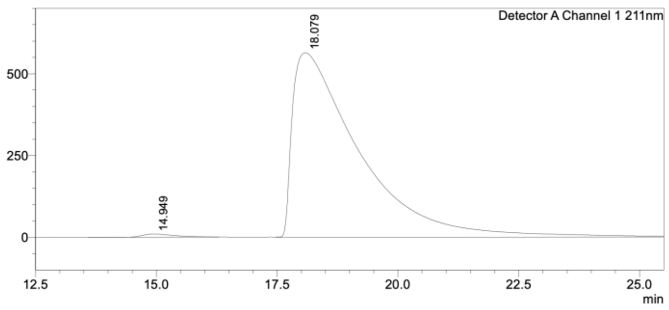

<Peak Table>

Detector A Channel 1 211nm

Peak#	Ret. Time	Area%
1	14.919	50.611
2	19.266	49.389
Total		100.000

<Peak Table>

Detector A Channel 1 211nm

Peak#	Ret. Time	Area%
1	14.949	0.822
2	18.079	99.178
Total		100.000

HPLC data for **36**, Chiralpak AD-H (90:10 hexane : IPA, flow rate 1mL.min^{-1}, 254 nm, 30 °C) t_R (4*R*): 20.3 min, t_R (*4S*): 26.2 min, 3:97 er

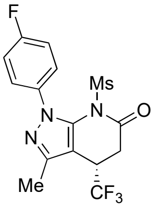

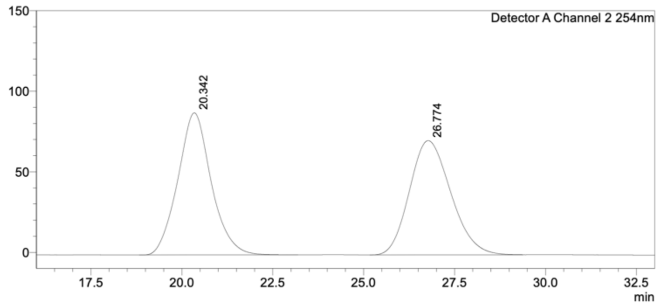

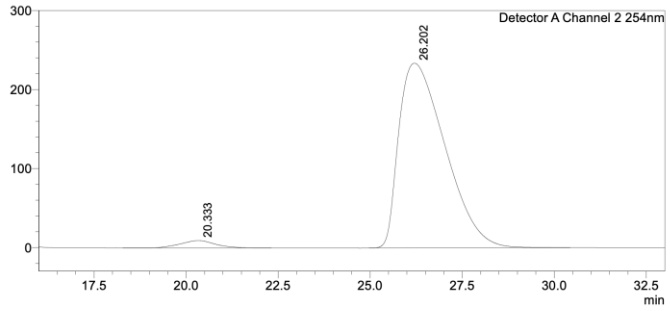

<Peak Table>

Detector A Channel 2 254nm

Peak#	Ret. Time	Area%
1	20.342	50.157
2	26.774	49.843
Total		100.000

<Peak Table>

Detector A Channel 2 254nm

Peak#	Ret. Time	Area%
1	20.333	2.924
2	26.202	97.076
Total		100.000

HPLC data for **37**, Chiralpak IC (85:15 hexane : IPA, flow rate 1mL.min^{-1}, 211 nm, 40 °C) t_R (4*S*): 10.8 min, t_R (4*R*): 15.9 min, 82:18 er

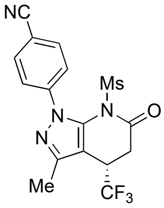

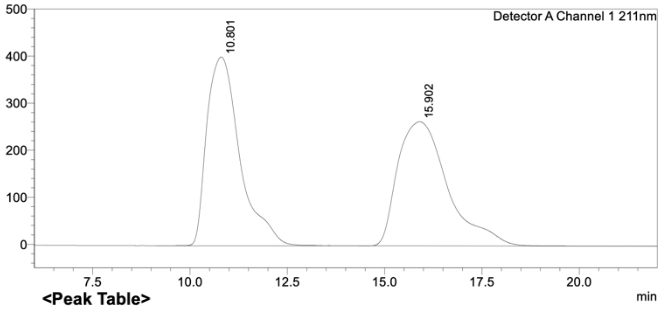

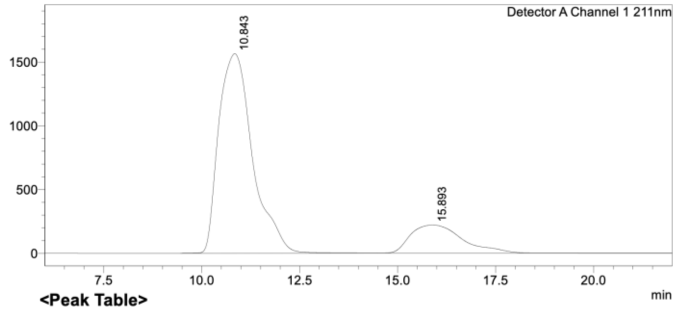

<Peak Table>

Detector A Channel 1 211nm

Peak#	Ret. Time	Area%
1	10.801	50.103
2	15.902	49.897
Total		100.000

<Peak Table>

Detector A Channel 1 211nm

Peak#	Ret. Time	Area%
1	10.843	82.060
2	15.893	17.940
Total		100.000

HPLC data for **38**, Chiralpak AD-H (90:10 hexane : IPA, flow rate 1mL.min^{-1}, 211 nm, 30 °C) t_R (4*S*):34.8 min, t_R (4*R*): 47.3 min, 81:9 er

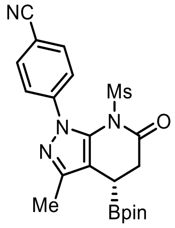

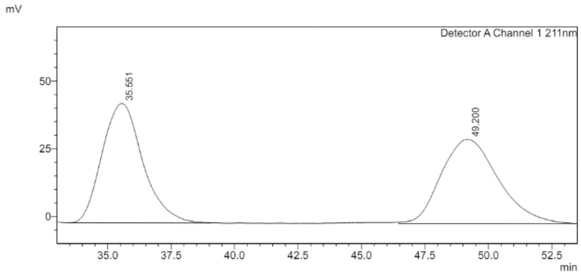

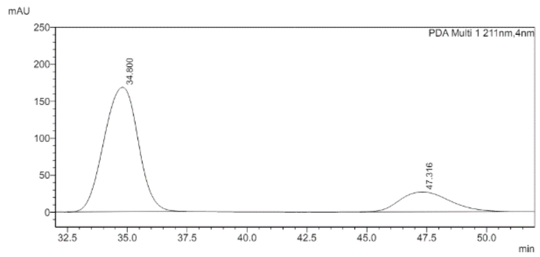

<Peak Table>

Detector A Channel 1 211nm

Peak#	Ret. Time	Area%
1	35.551	50.390
2	49.200	49.610
Total		100.000

<Peak Table>

PDA Ch1 211nm

Peak#	Ret. Time	Area%
1	34.800	80.924
2	47.316	19.076
Total		100.000

HPLC data for **39**, Chiralpak AD-H (92:8 hexane : IPA, flow rate 1mL.min^{-1}, 211 nm, 30 °C) t$_R$ (4*S*): 15.3 min, t$_R$ (4*R*): 20.3 min, >99:1 er

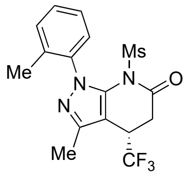

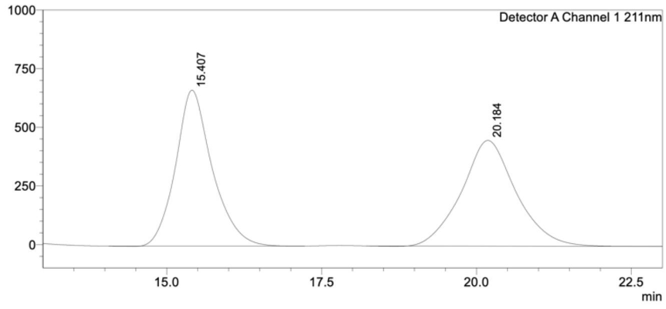

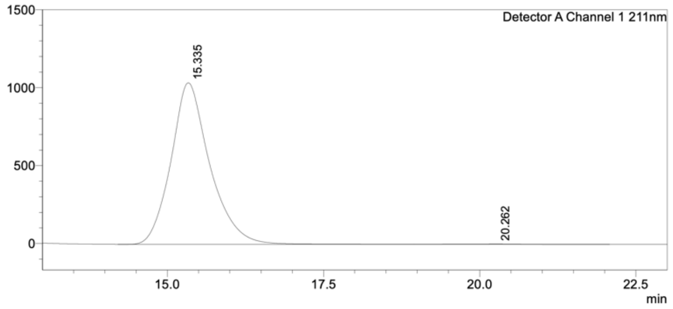

<Peak Table>

Detector A Channel 1 211nm

Peak#	Ret. Time	Area%
1	15.407	49.756
2	20.184	50.244
Total		100.000

<Peak Table>

Detector A Channel 1 211nm

Peak#	Ret. Time	Area%
1	15.335	99.638
2	20.262	0.362
Total		100.000

HPLC data for **41**, Chiralpak AD-H (92:8 hexane : IPA, flow rate 1mL.min^{-1}, 211 nm, 30 °C) t$_R$ (4*S*): 10.7 min, t$_R$ (4*R*): 13.8 min, 98:2 er

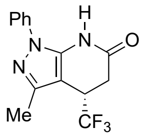

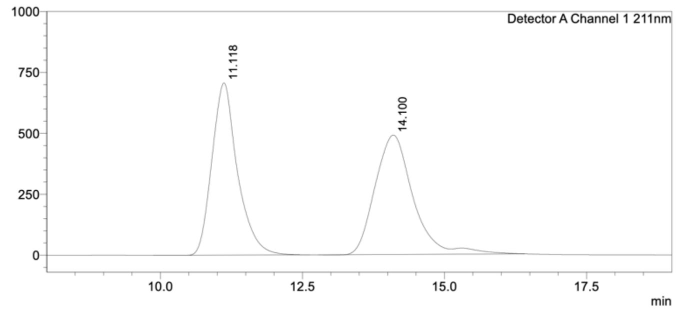

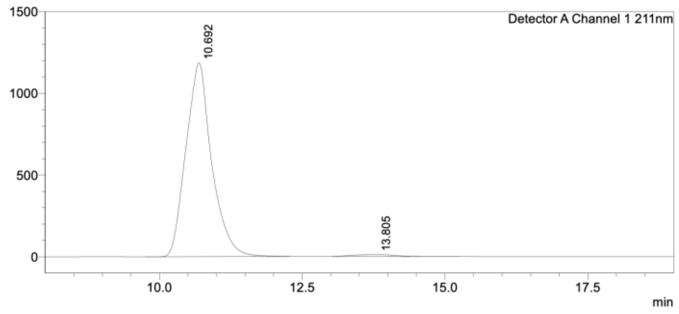

<Peak Table>

Detector A Channel 1 211nm

Peak#	Ret. Time	Area%
1	11.118	49.093
2	14.100	50.907
Total		100.000

<Peak Table>

Detector A Channel 1 211nm

Peak#	Ret. Time	Area%
1	10.692	98.486
2	13.805	1.514
Total		100.000

HPLC data for **40**, Chiralpak ID (90:10 hexane : IPA, flow rate 1mL.min^{-1}, 211 nm, 30 °C) t_R (4*S*): 14.6 min, t_R (4*R*): 17.9 min, 98:2 er

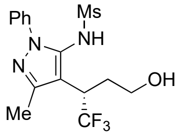

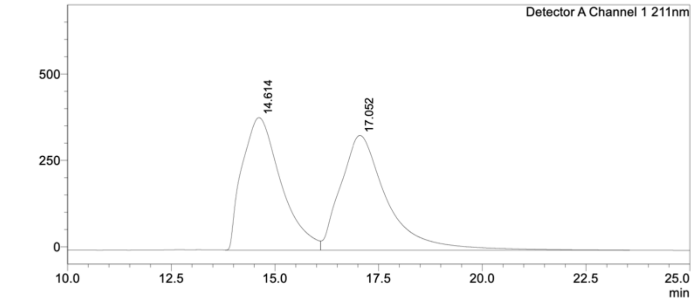

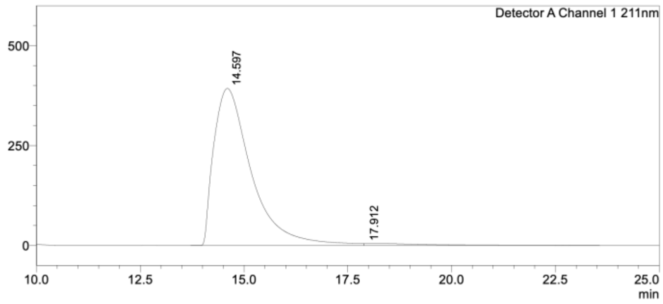

<Peak Table>

Detector A Channel 1 211nm

Peak#	Ret. Time	Area%
1	14.614	49.043
2	17.052	50.957
Total		100.000

<Peak Table>

Detector A Channel 1 211nm

Peak#	Ret. Time	Area%
1	14.597	97.920
2	17.912	2.080
Total		100.000

HPLC data for **44**, Chiralpak IA (98:2 hexane : IPA, flow rate 1.0 mL·min^{-1}, 254 nm, 30 °C) t_R (4*S*): 14.3 min, t_R (4*R*): 16.9 min, 2:98 er

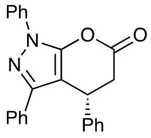

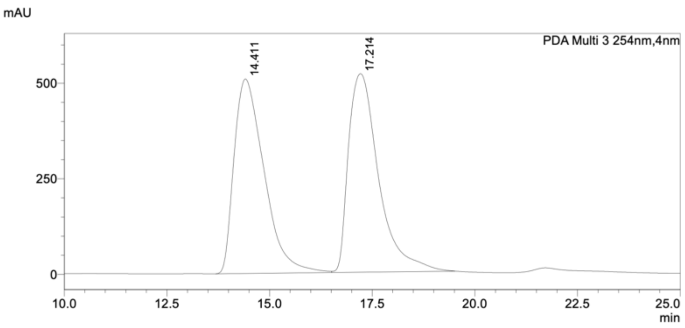

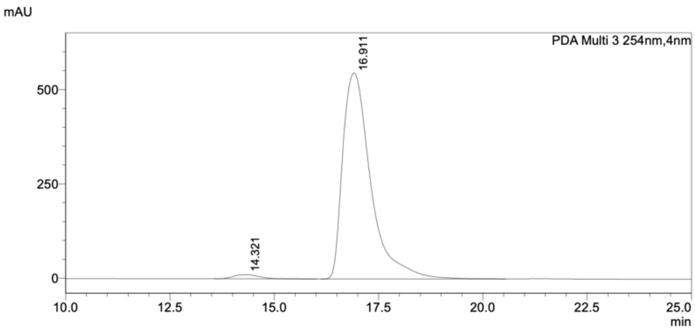

<Peak Table>

PDA Ch3 254nm

Peak#	Ret. Time	Area%
1	14.411	49.349
2	17.214	50.651
Total		100.000

<Peak Table>

PDA Ch3 254nm

Peak#	Ret. Time	Area%
1	14.321	1.889
2	16.911	98.111
Total		100.000

HPLC data for **47**, Chiralpak IA (98:2 hexane : IPA, flow rate 1.0 mL·min^{-1}, 220 nm, 30 °C) t_R (4*S*): 18.8 min, t_R (4*R*): 21.2 min, 3:97 er

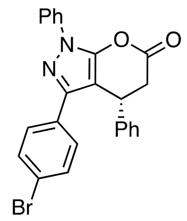

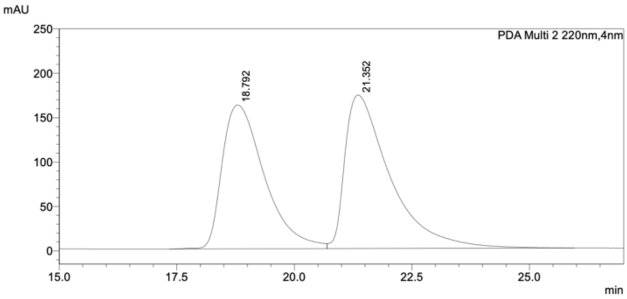

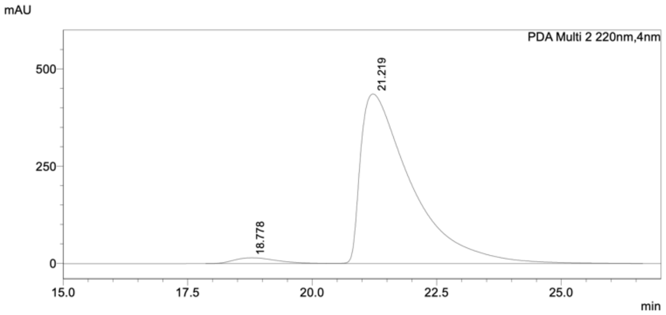

<Peak Table>

PDA Ch2 220nm

Peak#	Ret. Time	Area%
1	18.792	46.676
2	21.352	53.324
Total		100.000

<Peak Table>

PDA Ch2 220nm

Peak#	Ret. Time	Area%
1	18.778	2.742
2	21.219	97.258
Total		100.000

HPLC data for **48**, Chiralpak IA (95:5 hexane : IPA, flow rate 1.0 mL·min^{-1}, 270 nm, 30 °C) t_R (4*S*): 16.3 min, t_R (4*R*): 19.1 min, 2:98 er

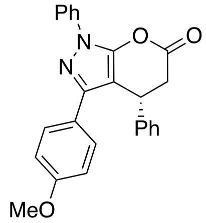

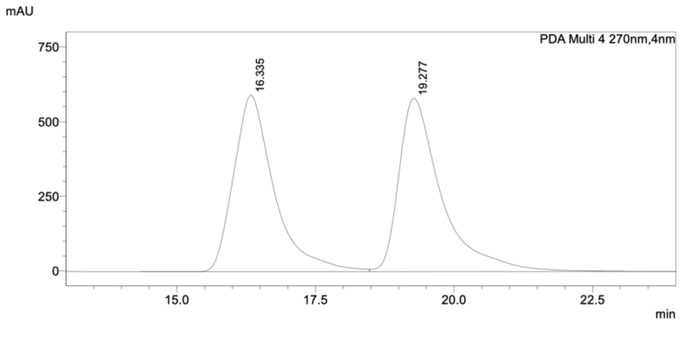

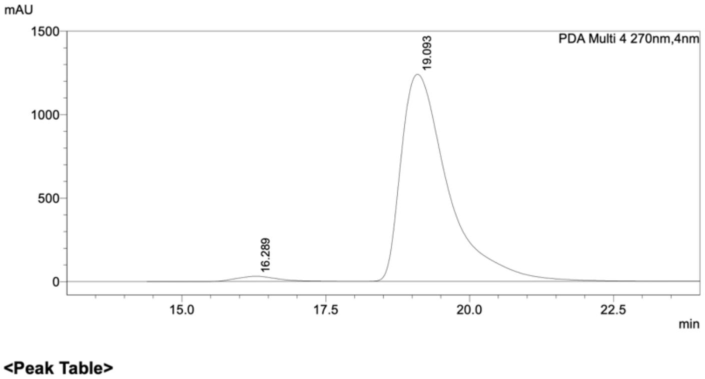

<Peak Table>

PDA Ch4 270nm

Peak#	Ret. Time	Area%
1	16.335	49.094
2	19.277	50.906
Total		100.000

<Peak Table>

PDA Ch4 270nm

Peak#	Ret. Time	Area%
1	16.289	2.169
2	19.093	97.831
Total		100.000

HPLC data for **49**, Chiralpak IB (98:2 hexane : IPA, flow rate 1.0 mL.min^{-1}, 211 nm, 30 °C) t_R (4*S*): 13.4 min, t_R (4*R*): 22.4 min, 4:96 er

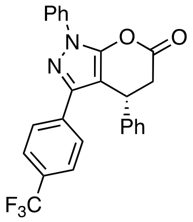

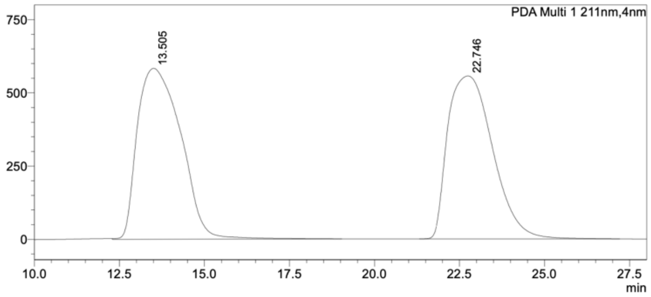

<Peak Table>

PDA Ch1 211nm

Peak#	Ret. Time	Area%
1	13.505	50.446
2	22.746	49.554
Total		100.000

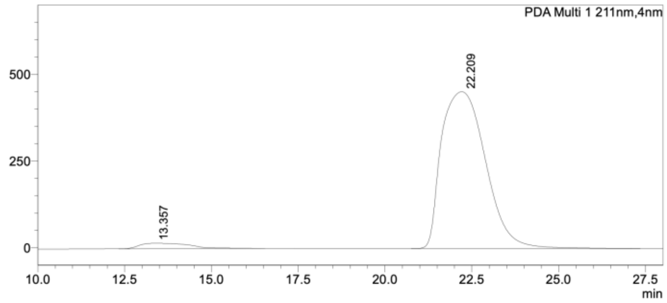

<Peak Table>

PDA Ch1 211nm

Peak#	Ret. Time	Area%
1	13.357	3.810
2	22.209	96.190
Total		100.000

HPLC data for **50**, Chiralpak IA (98:2 hexane : IPA, flow rate 1mL.min^{-1}, 220 nm, 30 °C) t_R (4*R*): 23.1 min, t_R (4*S*): 25.9 min, 3:97 er

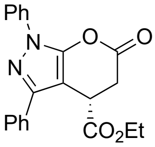

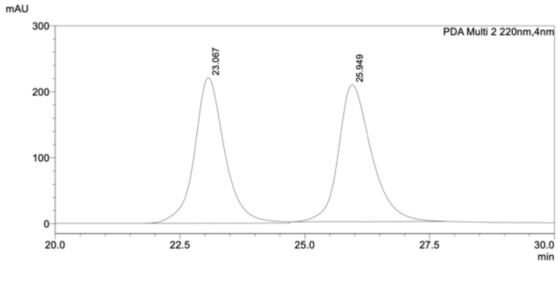

<Peak Table>

PDA Ch2 220nm

Peak#	Ret. Time	Area%
1	23.067	49.253
2	25.949	50.747
Total		100.000

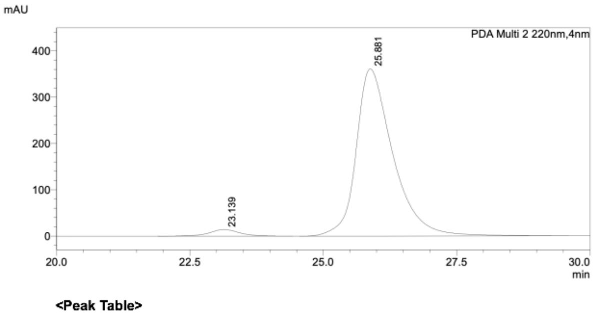

<Peak Table>

PDA Ch2 220nm

Peak#	Ret. Time	Area%
1	23.139	3.244
2	25.881	96.756
Total		100.000

HPLC data for **51**, Chiralpak IA (99:1 hexane : IPA, flow rate 1.0 mL·min^{-1}, 220 nm, 30 °C) t$_R$ (4*R*): 15.5 min, t$_R$ (4*S*): 17.9 min, 2:98 er

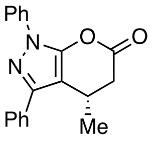

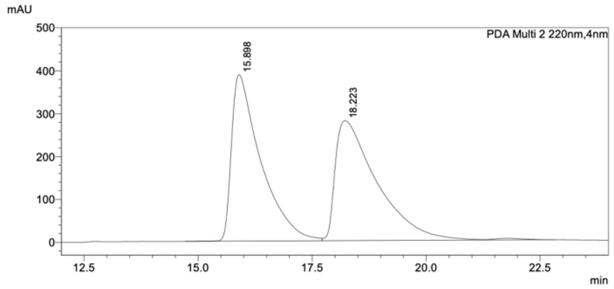

<Peak Table>

PDA Ch2 220nm

Peak#	Ret. Time	Area%
1	15.898	49.121
2	18.223	50.879
Total		100.000

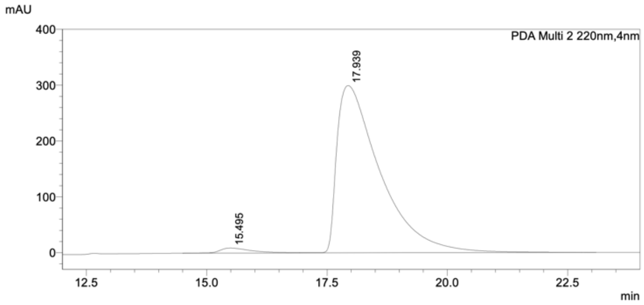

<Peak Table>

PDA Ch2 220nm

Peak#	Ret. Time	Area%
1	15.495	2.158
2	17.939	97.842
Total		100.000

HPLC data for **52**, Chiralcel OD-H (90:10 hexane : IPA, flow rate 1.0 mL.min^{-1}, 254 nm, 30 °C) t$_R$ (4*S*): 14.5 min, t$_R$ (4*R*): 17.0 min, 11:89 er

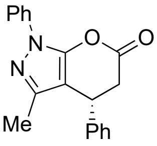

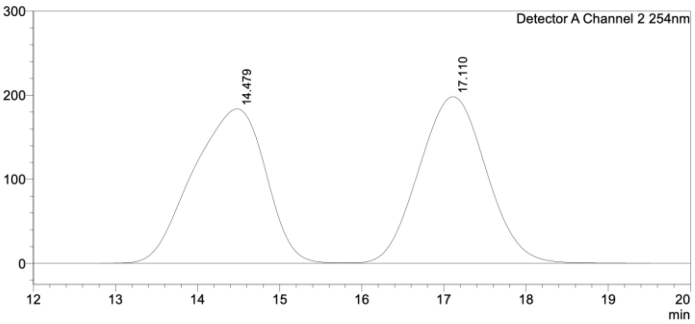

<Peak Table>

Detector A Channel 2 254nm

Peak#	Ret. Time	Area%
1	14.479	49.986
2	17.110	50.014
Total		100.000

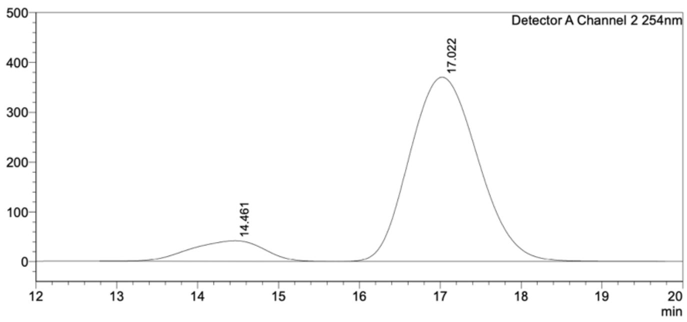

<Peak Table>

Detector A Channel 2 254nm

Peak#	Ret. Time	Area%
1	14.461	10.740
2	17.022	89.260
Total		100.000

HPLC data for **53**, Chiralpak IC (90.9:0.1 hexane : IPA, flow rate 1.0 mL·min^{-1}, 254 nm, 30 °C) t_R (4*S*): 11.3 min, t_R (4*R*): 12.8 min, 9:91 er

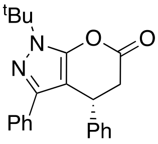

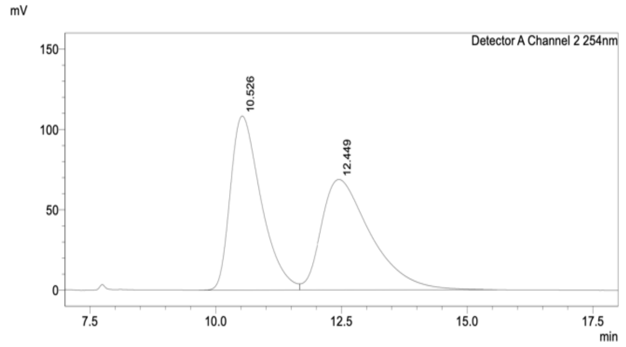

<Peak Table>

Detector A Channel 2 254nm

Peak#	Ret. Time	Area%
1	10.526	49.428
2	12.449	50.572
Total		100.000

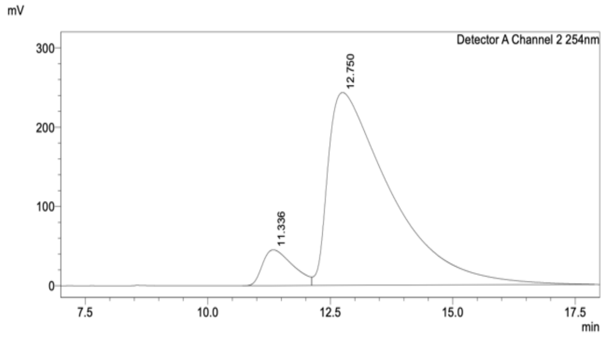

<Peak Table>

Detector A Channel 2 254nm

Peak#	Ret. Time	Area%
1	11.336	8.237
2	12.750	91.763
Total		100.000

HPLC data for **54**, Chiralpak IA (95:5 hexane : IPA, flow rate 1mL.min^{-1}, 220 nm, 30 °C) t_R (4*R*): 11.2 min, t_R (4*S*): 18.3 min, 7:93 er

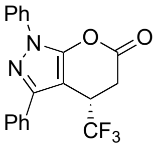

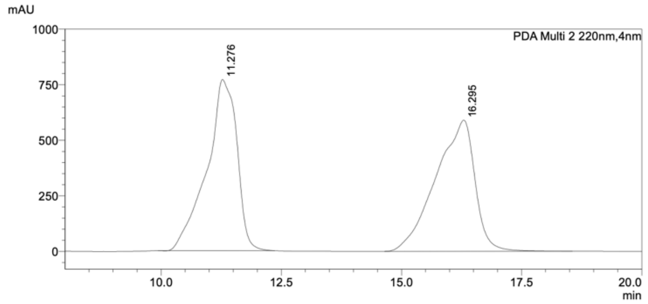

<Peak Table>

PDA Ch2 220nm

Peak#	Ret. Time	Area%
1	11.276	50.000
2	16.295	50.000
Total		100.000

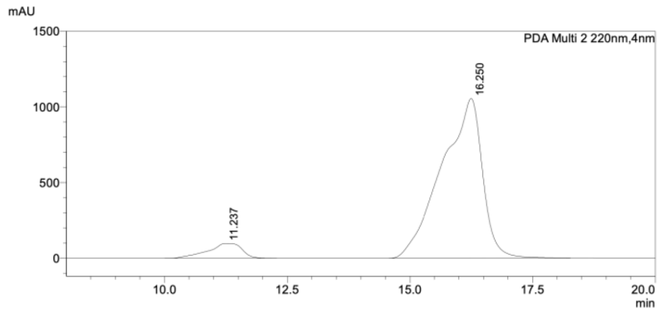

<Peak Table>

PDA Ch2 220nm

Peak#	Ret. Time	Area%
1	11.237	7.268
2	16.250	92.732
Total		100.000

HPLC data for **55**, Chiralpak IA (97:3 hexane : IPA, flow rate 1.0 mL·min^{-1}, 254 nm, 30 °C) t_R (4*S*): 17.2 min, t_R (4*R*): 22.5 min, 1:99 er

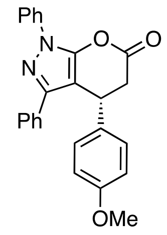

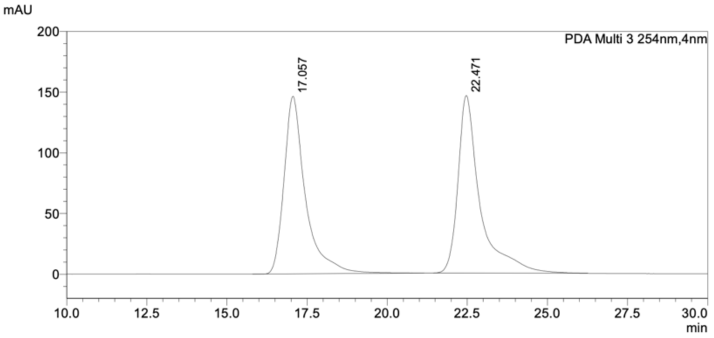

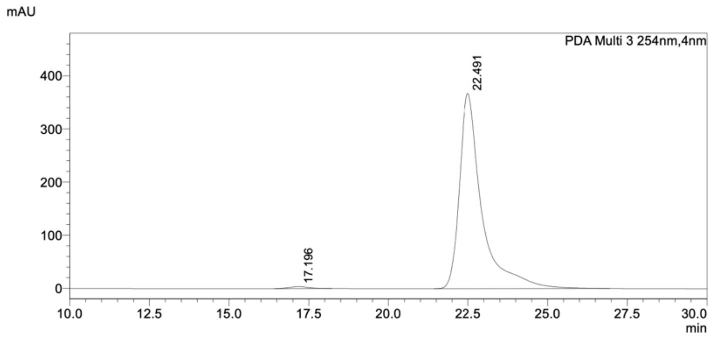

<Peak Table>

PDA Ch3 254nm

Peak#	Ret. Time	Area%
1	17.057	49.130
2	22.471	50.870
Total		100.000

<Peak Table>

PDA Ch3 254nm

Peak#	Ret. Time	Area%
1	17.196	0.841
2	22.491	99.159
Total		100.000

HPLC data for **56**, Chiralpak IC (99:1 hexane : IPA, flow rate 1.0 mL·min^{-1}, 2211 nm, 30 °C) t_R (4*S*): 37.9 min, t_R (4*R*): 46.5 min, 1:99 er

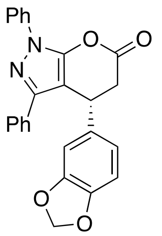

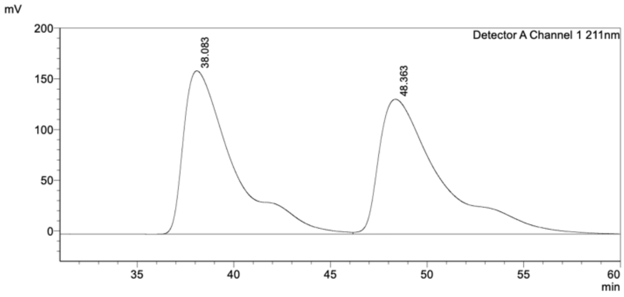

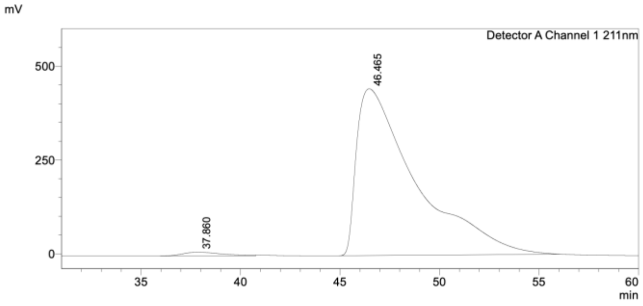

<Peak Table>

Detector A Channel 1 211nm

Peak#	Ret. Time	Area%
1	38.083	49.536
2	48.363	50.464
Total		100.000

<Peak Table>

Detector A Channel 1 211nm

Peak#	Ret. Time	Area%
1	37.860	1.497
2	46.465	98.503
Total		100.000

HPLC data for **57**, Chiralpak IA (97:3 hexane : IPA, flow rate 1.0 mL·min^{-1}, 220 nm, 30 °C) t_R (4*R*): 11.3 min, t_R (4*S*): 14.1 min, 95:5 er

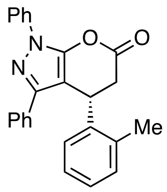

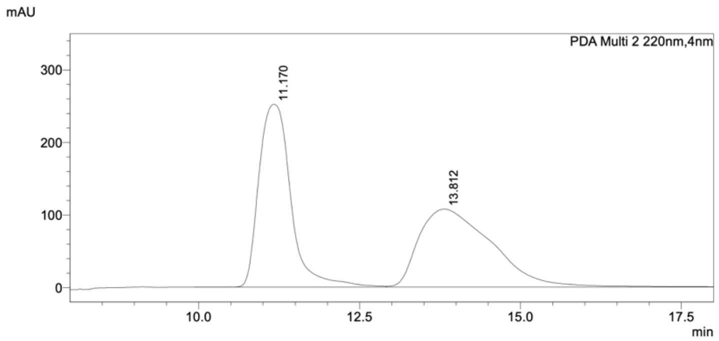

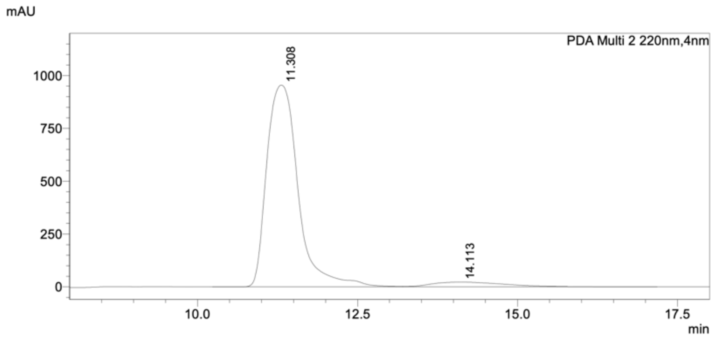

<Peak Table>

PDA Ch2 220nm

Peak#	Ret. Time	Area%
1	11.170	50.586
2	13.812	49.414
Total		100.000

<Peak Table>

PDA Ch2 220nm

Peak#	Ret. Time	Area%
1	11.308	94.871
2	14.113	5.129
Total		100.000

HPLC data for **58**, Chiralpak IA (90:10 hexane : IPA, flow rate 1.0 mL·min^{-1}, 270 nm, 30 °C) t_R (4*R*): 8.7 min, t_R (4*S*): 15.2 min, 97:3 er

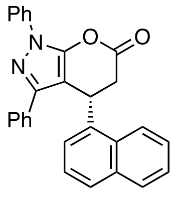

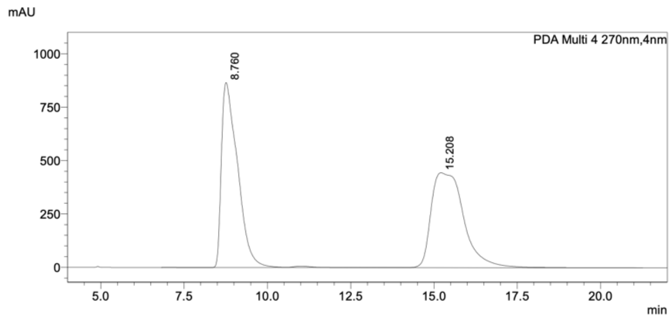

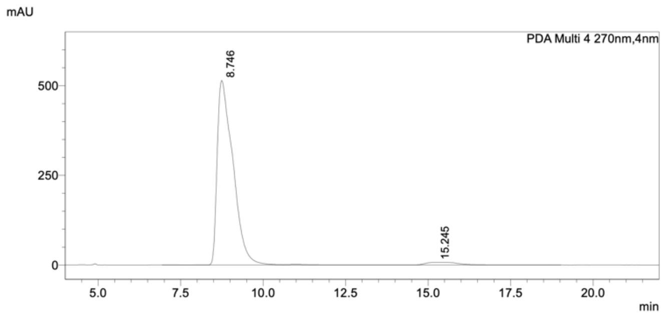

<Peak Table>

PDA Ch4 270nm

Peak#	Ret. Time	Area%
1	8.760	49.151
2	15.208	50.849
Total		100.000

<Peak Table>

PDA Ch4 270nm

Peak#	Ret. Time	Area%
1	8.746	96.999
2	15.245	3.001
Total		100.000

HPLC data for **59**, Chiralpak IA (97:3 hexane : IPA, flow rate 1.0 mL·min^{-1}, 220 nm, 30 °C) t_R (4*S*): 14.4 min, t_R (4*R*): 21.3 min, 2:98 er

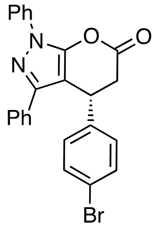

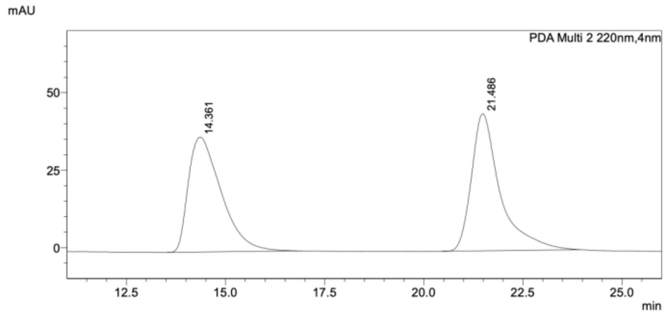

<Peak Table>

PDA Ch2 220nm

Peak#	Ret. Time	Area%
1	14.361	48.940
2	21.486	51.060
Total		100.000

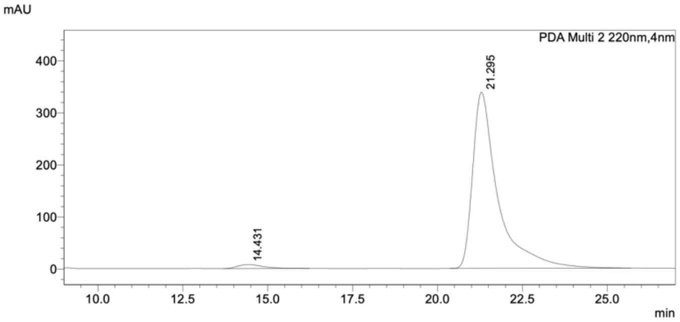

<Peak Table>

PDA Ch2 220nm

Peak#	Ret. Time	Area%
1	14.431	2.211
2	21.295	97.789
Total		100.000

HPLC data for **60**, Chiralpak IA (97:3 hexane : IPA, flow rate 1.0 mL·min^{-1}, 254 nm, 30 °C) t_R (4*S*): 12.4 min, t_R (4*R*): 17.9 min, 1:99 er

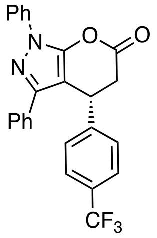

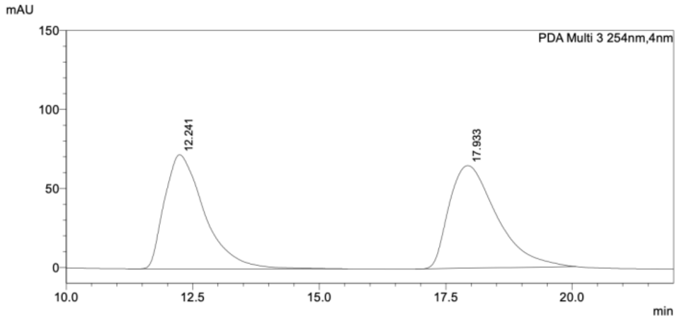

<Peak Table>

PDA Ch3 254nm

Peak#	Ret. Time	Area%
1	12.241	48.192
2	17.933	51.808
Total		100.000

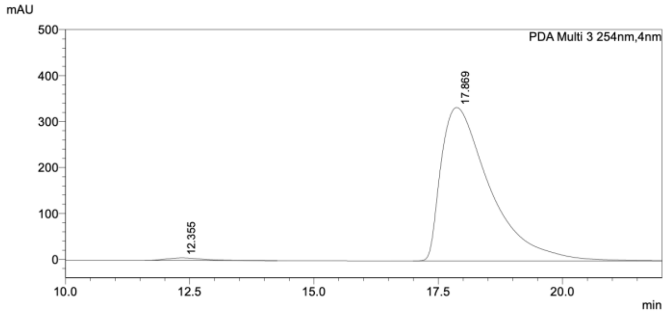

<Peak Table>

PDA Ch3 254nm

Peak#	Ret. Time	Area%
1	12.355	1.272
2	17.869	98.728
Total		100.000
